# RNA editing by the host ADAR system affects the molecular evolution of the Zika virus

**DOI:** 10.1002/ece3.3033

**Published:** 2017-05-15

**Authors:** Helen Piontkivska, Madeline Frederick, Michael M. Miyamoto, Marta L. Wayne

**Affiliations:** ^1^ Department of Biological Sciences and School of Biomedical SciencesKent State UniversityKentOHUSA; ^2^ Department of BiologyUniversity of FloridaGainesvilleFLUSA; ^3^ Emerging Pathogens InstituteUniversity of FloridaGainesvilleFLUSA

**Keywords:** ADAR, molecular evolution, RNA editing, RNA virus, Zika virus

## Abstract

Zika virus (ZIKV) is a mosquito‐transmitted flavivirus, linked to microcephaly and fetal death in humans. Here, we investigate whether host‐mediated RNA editing of adenosines (ADAR) plays a role in the molecular evolution of ZIKV. Using complete coding sequences for the ZIKV polyprotein, we show that potential ADAR substitutions are underrepresented at the ADAR‐resistant GA dinucleotides of both the positive and negative strands, that these changes are spatially and temporally clustered (as expected of ADAR editing) for three branches of the viral phylogeny, and that ADAR mutagenesis can be linked to its codon usage. Furthermore, resistant GA dinucleotides are enriched on the positive (but not negative) strand, indicating that the former is under stronger purifying selection than the latter. ADAR editing also affects the evolution of the rhabdovirus sigma. Our study now documents that host ADAR editing is a mutation and evolutionary force of positive‐ as well as negative‐strand RNA viruses.

## 1 Introduction

Zika virus (ZIKV) is a small (~10,800 base), single‐stranded, positive‐sense RNA virus from the genus *Flavivirus* (Flaviviridae) (Fields, Knipe, Howley, & Griffin, 2013). Other important members of the genus *Flavivirus* include the dipteran‐vectored human pathogens: West Nile virus (WNV), Japanese encephalitis (JEV), and Dengue virus (DENV) (Faye et al., 2014). The family Flaviviridae also includes the classical swine fever virus (Pestivirus) and hepatitis C virus (Hepacivirus) (Ryu, 2016). ZIKV is transmitted primarily by mosquitos, including members of the *Aedes* and *Anopheles* genera (Faye et al., 2014), although other modes of transmission, including sexual and maternal‐fetal, have been reported (Brasil et al., 2016; Musso et al., 2015; Venturi et al., 2016). There are additional concerns about the possibility of transmission through the blood supply due to infected donors (Marano, Pupella, Vaglio, Liumbruno, & Grazzini, 2016; Musso et al., 2014).

Discovered in 1947 in Uganda, ZIKV remained in relative obscurity (Dick, 1952; Simpson, 1964) until the outbreak in Brazil in 2015, when the initial link between ZIKV and microcephaly was first described (Mlakar et al., 2016). This association between ZIKV infection and subsequent increase in risk (of up to 13%; Johansson, Mier‐y‐Teran‐Romero, Reefhuis, Gilboa, & Hills, 2016) of infant neurodevelopmental abnormalities, including microcephaly and other severe brain defects, was soon confirmed with multiple follow‐up studies (Cauchemez et al., 2016; Franca et al., 2016 ). In adults, ZIKV infections are largely asymptomatic, although in rare cases, ZIKV was linked to an increased incidence of Guillain‐Barré syndrome (Cao‐Lormeau et al., 2016; Duffy et al., 2009; Focosi, Maggi, & Pistello, 2016). Robust and sustained efforts are necessary to delineate the fundamental mechanisms of ZIKV infection and associated health complications.

Innate immunity mechanisms, particularly those shared by dipterans and humans, may contribute useful insights into the evolution of ZIKV. For example, adenosine‐to‐inosine (A → I) RNA editing by ADAR (“adenosine deaminases acting on RNA”) enzymes is one of the mechanisms of gene regulation conserved throughout metazoans (Bass, 2002; Grice & Degnan, 2015; Jin, Zhang, & Li, 2009; Palladino, Keegan, O'Connell, & Reenan, 2009). Because A → I editing leads to A → G transitions, which in turn often results in amino acid substitution, ADAR action contributes to proteome diversification and is considered to be part of the host “editome” (Chen et al., 2014; Keegan, Gallo, & O'Connell, 2001). ADARs may also play a role in host antiviral response (Carpenter, Keegan, Wilfert, O'Connell, & Jiggins, 2009; Keegan et al., 2001; Kumar & Carmichael, 1997; Samuel, 2011; Scadden, 2005).

In this study, we examine the molecular evolution of publicly available sequences of ZIKV to determine whether or not these genomes exhibit the signatures of ADAR‐associated changes. Specifically, we assessed the differential variabilities and frequencies of weak and strong dinucleotide targets of ADAR on both the positive and negative strands of ZIKV; evaluated the temporal, as well as spatial, clustering of potential ADAR substitutions; and compared the usage of synonymous A‐ and G‐ending codons by ZIKV given expectations under ADAR editing, and relative to its human and mosquito hosts. Our study provides new evidence that ADAR editing is a mutational and potentially a selective force in ZIKV.

## 2 Methods

### 2.1 Zika virus genomes used and multiple sequence alignment

Initially, 115 ZIKV, complete or near‐complete, polyprotein‐encoding, nucleotide sequences were collected from GenBank (release 215.0, August, 2016). Of these, 107 sequences encoding complete polyproteins were collected from the ZIKV Variation Resource (http://www.ncbi.nlm.nih.gov/genome/viruses/variation/Zika/). Additionally, eight complete or partial ZIKV sequences were added following BLASTN searching against the nr database (the NC_012532 sequence was used as the query). Only those partial polyprotein‐encoding ZIKV sequence matches that had at least 95% query coverage were included. Of these, one sequence (KF383120) was annotated as a nonfunctional polyprotein gene (Faye et al., 2014) and therefore was excluded from further consideration. Thus, preliminary analyses were based upon 114 ZIKV (complete and almost complete) polyprotein‐encoding gene sequences.

Upon further examination of the sequence annotations, closely related sequences that were annotated as derived by multistep passaging (via multiple protocols) and/or identical were flagged and removed from the subsequent analyses. It should be noted that in a study that contrasted sequences derived through direct sequencing of a sample versus a single passage in Vero cells (Barzon et al., 2016), only a single synonymous nucleotide substitution was detected, suggesting a quite low error rate that may be directly attributed to passaging. Regardless, to minimize the possibility of mutations or adaptations to environments other than intact mosquitos or humans, we focused our analyses on ZIKV sequences whose GenBank annotations did not explicitly indicate that multiple passaging events had occurred, with the sole exception of the oldest available ZIKV sequences (including NC_012532, designated as the MR‐766 strain) (Kuno & Chang, 2007). Ultimately, 56 sequences remained.

Table S1 lists the GenBank accession numbers of the 56 ZIKV sequences used in the final analyses. Following the removal of their noncoding 5′ and 3′ termini, these 56 sequences were aligned to each other using ClustalW as implemented in MEGA6 (Tamura, Stecher, Peterson, Filipski, & Kumar, 2013). The final multiple sequence alignment for the subsequent comparative analyses included 10,272 positions of contiguous coding RNA for the ZIKV polyprotein (Figure S1).

### 2.2 Phylogenetic inference

Phylogenetic analysis of the aligned ZIKV genomes was performed under the maximum likelihood (ML) criterion with PHYML v3.0 (Guindon et al., 2010). This inference relied on the BIONJ and ten randomly selected trees as the starting phylogenies, on both NNI and SPR branch swapping, and on the GTR+Γ+F model that was chosen for these genomes by PHYML's Smart Model Selection. The reliability of the ML phylogeny was assessed with 1,000 bootstrap replicates. To root the ML phylogeny, the Spondweni virus genome (GenBank accession number DQ859064) was aligned as before to the 56 ZIKV sequences. To minimize the divergence between the outgroup and study group, the Spondweni and ZIKV genomes were translated into their inferred polypeptide sequences prior to their PHYML comparison. This outgroup analysis relied on the same run conditions as used before for the study group alone, except that the JTT + Γ + F model was chosen for these protein sequences by PHYML's Smart Model Selection. The assignment of the root by this protein‐based outgroup analysis was compared against those supported by available flavivirus phylogenies (e.g., Grard et al., 2010; Sironi, Forni, Clerici, & Cagliani, 2016) and by midpoint rooting. The original nucleotide‐based PHYML phylogeny was then rooted according to the congruence among these three complementary approaches.

### 2.3 Base and dinucleotide frequency calculations

Base and dinucleotide frequencies were first calculated for the two reference genomes, KF383118 from the Senegal (Faye et al., 2014) and KU744693 from a recent Chinese traveler to Venezuela (Liu et al., 2016). These two genomes were selected to represent the range of nucleotide diversity among the 56 ZIKV sequences and for our detailed statistical tests, because they were the most likely to be independent as they belonged to the two most distantly related phylogenetic groups (see below) and differed the most according to their proportional distance (*p* = .124) (Felsenstein, 2003). Still, for the sake of completeness, we also calculated the base and dinucleotide frequencies for the other 54 genomes and summarized the proportions for all 56 ZIKV sequences as their means and ranges (Table S2).

The base compositions at the first, second, third, and all codon positions of the ZIKV genomes were calculated with MEGA6 (Tamura et al., 2013). The dinucleotide frequencies of the genomes were then determined with SEQOOL v2.0 (Wang, 2006). The dinucleotide calculations focused on the NA and UN frequencies of the positive strand (i.e., on those dinucleotides with a 3′ A in their positive or negative complement, respectively). The CG frequencies of the two references were also calculated in light of the well‐known CG deficit among many viruses, bacteria, and eukaryotes (Karlin & Burge, 1995; Lobo et al., 2009).

### 2.4 Testing the frequencies of conserved and variable dinucleotides

GA and AA/CA/UA dinucleotides were classified as weak and strong targets of ADAR, respectively (Bass, 2002; Eggington, Greene, & Bass, 2011). Potential ADAR substitutions (A → G) on the positive strand were identified by reconstructing the character state changes of the 56 ZIKV genomes on their PHYML phylogeny under the ML criterion with PAUP* v4.0b (Swofford, 2003). These reconstructions relied on the GTR + Γ + F model with its relative substitution rates, gamma shape parameter, and equilibrium base frequencies fixed to their ML estimates from PHYML.

Subsequent analysis of potential ADAR substitutions focused on those at third codon positions. Specifically, third codon positions with one or more A → G on the PHYML tree and with an invariant 5′ neighbor (i.e., second codon positions) were scored as variable AA, CA, GA, and UA. Conversely, third codon positions with an unchanged A and an invariant 5′ neighbor were counted as conserved AA, CA, GA, and UA at the second and third codon positions. U → C and UN are the reverse complements of A → G and NA (IUPAC code N stands for any nucleotide). Accordingly, U → C at the third codon positions and their 3′ neighbors were scored in the same way as for A → G and NA (i.e., as conserved and variable UA, UC, UG, and UU at the third and first codon positions). These counts of conserved and variable UN at third and first codon positions allowed for comparable tests on the negative strand as well as on the positive sequence of weak and strong dinucleotides and their A → G variability.

Under the standard genetic code, all U → C and almost all A → G transitions at third codon positions are synonymous, except for isoleucine (AUA) → methionine (AUG) and STOP (UGA) → tryptophan (UGG). As the latter affects the normal termination of translation, and thus are usually strongly deleterious, UGA → UGG mutations are expected to make only a negligible contribution to the molecular evolution of ZIKV (Graur, 2016). In turn, the frequency of AUA was only ~1.5% for our 56 ZIKV genomes according to their codon usage analysis (see below). Thus, our set of potential ADAR substitutions at third codon positions almost entirely consisted of synonymous changes that are typically regarded as neutral (Graur, 2016).

### 2.5 Testing the frequencies of weak and strong dinucleotides

To test for significant dinucleotide excesses and deficits of NA, UN, and CG, 1,000 random sequences were separately simulated for the two references (KF383118 and KU744693) with the custom C++ program of Piontkivska et al. (2016). These simulations were carried out under a model of equal sequence length and underlying base frequencies at first, second, and third codon positions as present in the reference genome. The dinucleotide frequencies of the random sequences were calculated as before with SEQOOL and were then summarized as the null distributions for testing the observed NA, UN, and CG proportions of their references.

### 2.6 Testing for spatial clustering of potential ADAR substitutions

In DMelSV, ADAR‐introduced changes are observed to be spatially clustered, separated by up to a few hundred bases at most (Carpenter et al., 2009; Piontkivska et al., 2016). To test for such clustering, the alignment numbers (i.e., locations in the multiple sequence alignment) of all three codon positions (first and second as well as third) with A → G and U → C were separately compiled for each external branch and internal branch of the PHYML phylogeny. For each branch, the minimum absolute distance between every variable site and its immediate upstream or downstream counterpart (in bases) was calculated, and the median shortest interval between all such neighbors was determined as an estimate of spatial clustering between potential ADAR substitutions. To test for significant spatial clustering, the variable sites of each branch were randomly reassigned to unique locations of the multiple sequence alignment, and their median minimum absolute distance was then recalculated as before. For each branch, this procedure was repeated 1,000 times, and the median minimum absolute distances for all 1,000 permutations were summarized as the null distribution for testing its observed spatial clustering of potential ADAR substitutions.

Our current branch‐by‐branch test of spatial clustering is based on the premise that individual bouts of ADAR editing happen at particular points in time such that their A → G and U → C are temporally as well as spatially clumped (Eggington et al., 2011). Thus, our approach acknowledges that the substitutions of a single ADAR event should not be dispersed among different branches of the phylogeny, but will instead co‐occur on the same lineage. Furthermore, our current test relies on the median of the minimum absolute distances between adjacent sites with potential ADAR substitutions, rather than on the average of their intervals for all neighbor and nonneighbor pairwise comparisons (Carpenter et al., 2009; Piontkivska et al., 2016). Thus, our test offers a more sensitive approach as it most heavily weights the shorter distances that are the basis of spatial clustering.

### 2.7 Viral and host codon usage

Codon usage tables were generated for the two ZIKV references (KF383118 and KU744693) with MEGA6 (Tamura et al., 2013). Codon usage focused on the synonymous A‐ and G‐ending triplets of those amino acids with NNR redundancy according to the standard genetic code (IUPAC code R designates a purine, A or G). The usage of synonymous A‐ and G‐ending triplets by the two references was assessed in terms of the weak and strong dinucleotides at second and third codon positions and the well‐documented deficits of CG and UA among different viruses, bacteria, and eukaryotes (Karlin & Burge, 1995; Lobo et al., 2009).

The use of synonymous A‐ and G‐ending triplets by the two ZIKV references was compared to the codon usage tables for their human and mosquito hosts as obtained from Nakamura, Gojobori, and Ikemura (2000). The codon usage tables for the yellow fever mosquito (*Aedes aegypti*) and Asian tiger mosquito (*A. albopictus*) were chosen from all mosquitos with a known genome, because they are well‐documented carriers of ZIKV (Grard et al., 2014; Marchette, Garcia, & Rudnick, 1969). Comparisons of ZIKV and its hosts were conducted under the assumption that the codon preferences of humans and mosquitos are positively correlated with their relative abundances of cognate tRNAs, which thereby leads to enhanced translational accuracy and efficiency (Graur, 2016; Sharp, Emery, & Zeng, 2010). Given this assumption, these comparisons allowed for an assessment of how the usage of synonymous A‐ and G‐ending codons by ZIKV is structured when the availability of the host cognate tRNAs varies for the accurate and efficient translation of the virus.

## 3 Results

### 3.1 Maximum likelihood phylogeny

**Figure 1:**
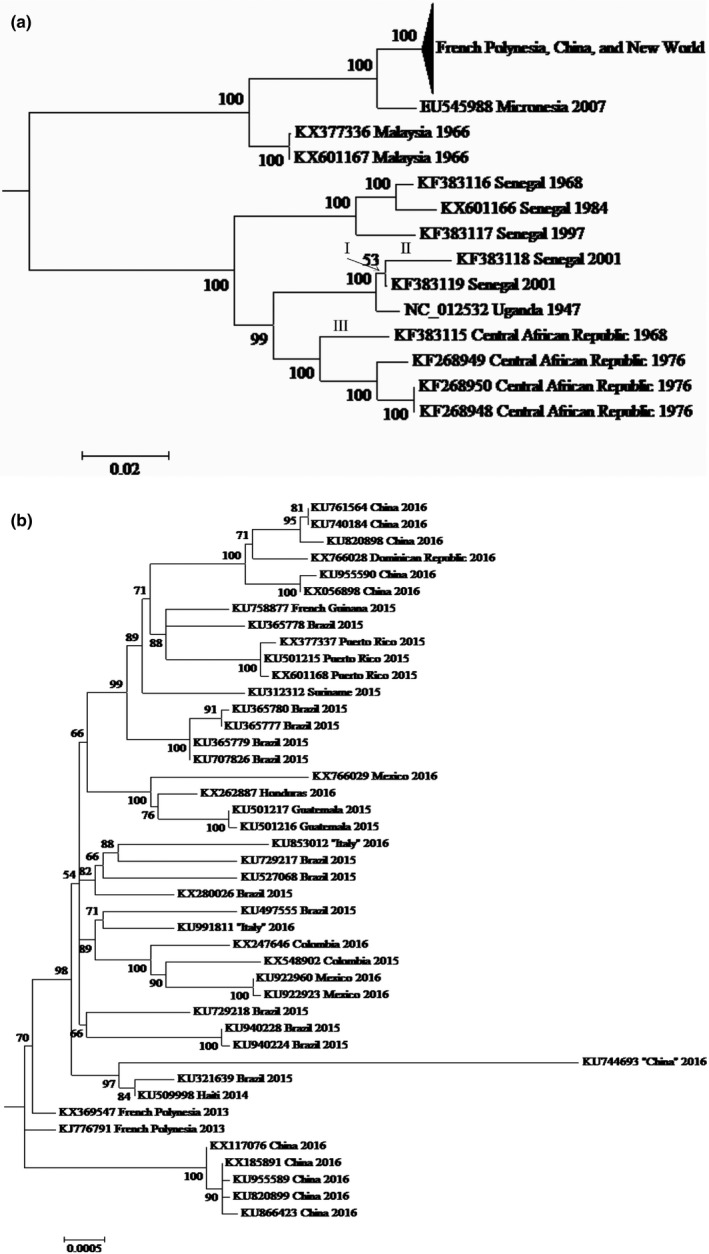
(A) ML phylogeny for the 56 ZIKV genomes with the French Polynesian, Chinese, and New World subtree compressed. (B) Expansion of the French Polynesian, Chinese, and New World subtree. For diagrammatic purposes, this ML phylogeny is drawn as midpoint rooted (i.e., although all three rooting approaches assign the root to the same internal branch, only midpoint rooting specifies an exact location of the root on this internode of the nucleotide‐based tree). Bootstrap scores are given next to internal internodes, whereas branch lengths are drawn proportional to their inferred amounts of evolutionary change [note the different scales in (A) and (B)]. Genomes are labeled by their GenBank accession numbers and their countries and dates of collection. The countries for KU744693, KU853012, and KU991811 are in parentheses, because their genomes are from recent travelers to Venezuela, the Dominican Republic, and Brazil, respectively. In (A), “I–III” mark the three branches with significant spatial clustering of their potential ADAR substitutions (Figure 3)

The PHYML protein comparison with the Spondweni virus as the outgroup, the available flavivirus phylogenies (e.g., Grard et al., 2010; Sironi et al., 2016), and our own midpoint rooting analysis all agree that the root for the ZIKV ML phylogeny lies somewhere along the internal branch that bipartitions the ten African genomes from the 46 Asian, Oceanian, and New World sequences (Figure 1). Within the Asian, Oceanian, and New World group, the two Malaysian genomes diverge first, followed by the split of the Micronesian sequence from the French Polynesian, Chinese, and American subtree (Faria et al., 2016; Wang et al., 2016). Within the latter, the 11 Chinese genomes are subdivided among three distinct lineages (Fajardo, Soñora, Moreno, Moratorio, & Cristina, 2016). Furthermore, the three Chinese and Italian genomes from recent visitors to Latin America (KU744693, KU853012, and KU991811) are most closely allied to Brazilian and/or Haitian sequences, which is consistent with their acquisition during travel rather than locally. These relationships of the ZIKV ML phylogeny are all moderately to strongly supported by bootstrap values of 88%–100%, with the sole exception of the 71% union of KU991811 to KU497555 from Brazil (Figure 1).

Although sufficient for polarizing the more recent substitutions, the rooting of the ML phylogeny on the internal branch that bipartitions the African genomes from the Asian, Oceanian, and New World sequences is insufficient to direct changes along the two basal branches that lead to these groups (Figure 1). This limitation stems from the fact that none of the three complementary approaches for rooting involves a character‐by‐character outgroup analysis at the nucleotide level. Instead, our outgroup analysis with the Spondweni virus is at the protein level, whereas midpoint rooting depends on the overall pattern of divergence rather than on the per‐character variation. Furthermore, little overlap exists between the ZIKV genomes of this study and those of previous investigations on flavivirus phylogeny, which thereby limits the use of the latter to polarize the current basal changes. For these reasons, changes along the two basal branches are not included in our following tests of A → G and U → C, because they necessarily remain undirected (i.e., as unpolarized A ↔ G and U ↔ C, respectively).

### 3.2 Base composition variation among the three codon positions

Overall, the genomes of the two ZIKV references (KF383118 and KU744693) are both purine‐rich (%R of 56.8% and 56.6%, respectively; Table 1) (van Hemert & Berkhout, 2016). This base composition bias is most evident at first codon positions (66.4% and 66.6%), and less pronounced, but still present at third positions (55.3% and 54.6%), respectively. For first and third codon positions, the A and G frequencies consistently exceed equal representation by all four nucleotides (i.e., 25%). However, at second codon positions, %R (sum of A and G) is less than the expected 50% (48.6% and 48.7% for KF383118 and KU744693), because of the decreased frequencies of G (0.222 and 0.223), respectively. Thus, unlike the other positions, second codon positions are not purine‐rich. Substitutions at second codon positions invariably result in amino acid changes, which can affect fitness, so this purine deficit is consistent with purifying selection to protect vulnerable second codon positions from attack by ADAR. These trends are consistent with those for the full set of 56 ZIKV genomes, whose base frequencies vary little (Table S2).

**Table 1:** Base compositions at first, second, third, and all codon positions for the two ZIKV references, KF383118 and KU744693. IUPAC code R designates a purine, A or G

Reference	First codon positions					Second codon positions					Third codon positions					All codon positions				
	U	C	A	G	%R	U	C	A	G	%R	U	C	A	G	%R	U	C	A	G	%R
KF383118	0.163	0.173	0.305	0.359	66.4%	0.286	0.228	0.264	0.222	48.6%	0.198	0.249	0.262	0.291	55.3%	0.215	0.217	0.277	0.291	56.8%
KU744693	0.163	0.171	0.305	0.361	66.6%	0.283	0.230	0.264	0.223	48.7%	0.197	0.257	0.251	0.295	54.6%	0.214	0.220	0.273	0.293	56.6%

### 3.3 GA and CA are conserved on both the positive and negative strands

The four NA dinucleotides at second and third codon positions (i.e., with ADAR targets at third positions) differ significantly in their frequencies of conserved and variable dinucleotides [Table 2; chi‐square test of heterogeneity with Yates’ correction ($\chi_{c}^{2}$) = 36.985, degrees of freedom (*df*) = 3, *p* < .001]. CA and GA are more frequently conserved than AA and UA (23.4% and 30.4% variable versus 49.5% and 47.0%, respectively), whereas CA and GA themselves are similarly conserved ($\chi_{c}^{2}$ = 2.380, *df* = 1, *p* = .123). Focusing on the complement, the four UN dinucleotides spanning the third and first codon positions (i.e., with ADAR targets corresponding to the third position, but their 5′ bases now at the first position) also differ in their conserved and variable frequencies (Table 2; $\chi_{c}^{2}$ = 115.431, *df* = 3, *p* < .001). UC (complement of GA) is again more frequently conserved than UA and UU (complementary to UA and AA; 71.8% variable versus 95.0% and 86.4%, respectively; $\chi_{c}^{2}$ = 17.101, *df* = 2, and *p* < .001). However, UC is now more variable than UG (complement of CA; 43.6% variable; $\chi_{c}^{2}$ = 18.967, *df* = 1, *p* < .001).

**Table 2:** Frequencies of conserved and variable NA and UN at second/third and third/first codon positions, respectively. UU, UG, UC, and UA at third/first codon positions are the complements of AA, CA, GA, and UA on the negative strand, respectively. “Conserved” refers to those NA and UN with unchanged A and U at their third codon positions, respectively. Conversely, “variable” refers to those NA and UN with at least one A → G and U → C at their third codon positions, respectively. IUPAC code N stands for any nucleotide

Positive‐strand ADAR targets				Negative‐strand ADAR targets			
NA dinucleotides at second/third codon positions	Conserved NA	Variable NA	Totals (% variable NA)	UN dinucleotides at third/first codon positions	Conserved UN	Variable UN	Totals (% variable UN)
AA	99	97	196 (49.5%)	UU	12	76	88 (86.4%)
CA	167	51	218 (23.4%)	UG	184	142	326 (43.6%)
GA	151	66	217 (30.4%)	UC	22	56	78 (71.8%)
UA	53	47	100 (47.0%)	UA	5	95	100 (95.0%)
Totals	470	261	731 (35.7%)	Totals	223	369	592 (62.3%)

One complication of the UN tests is that the total number of UG (326) exceeds the sum for all other UN dinucleotides (UA + UC + UU = 266; Table 2). The low total number of UA is attributable to its well‐documented deficit among viruses, bacteria, and eukaryotes (see below), whereas the low numbers of UC and UU are tied to the rapid rate of C ↔ U at the first codon positions of the synonymous CUN and UUR triplets for leucine. The C ↔ U rate is >3 times faster than those for any other substitution type and the amino acid composition of leucine is >9% for ZIKV proteins according to our PHYML and codon usage analyses. The counting of conserved and variable UC and UU at third and first codon positions relies on an invariant C and U at the first positions, respectively. Thus, frequent switching between the synonymous CUN and UUR codons for leucine (which is an abundant amino acid of ZIKV proteins) reduces the numbers of invariant C and U at first codon positions for UC scoring and UU scoring. Despite this complication, the NA and UN tests agree that GA and CA are conserved on both the positive and negative strands. This conservation is consistent with the status of GA as a weak ADAR target, but not with the designation of CA as a strong ADAR substrate.

### 3.4 Dinucleotide excesses and deficits

**Figure 2:**
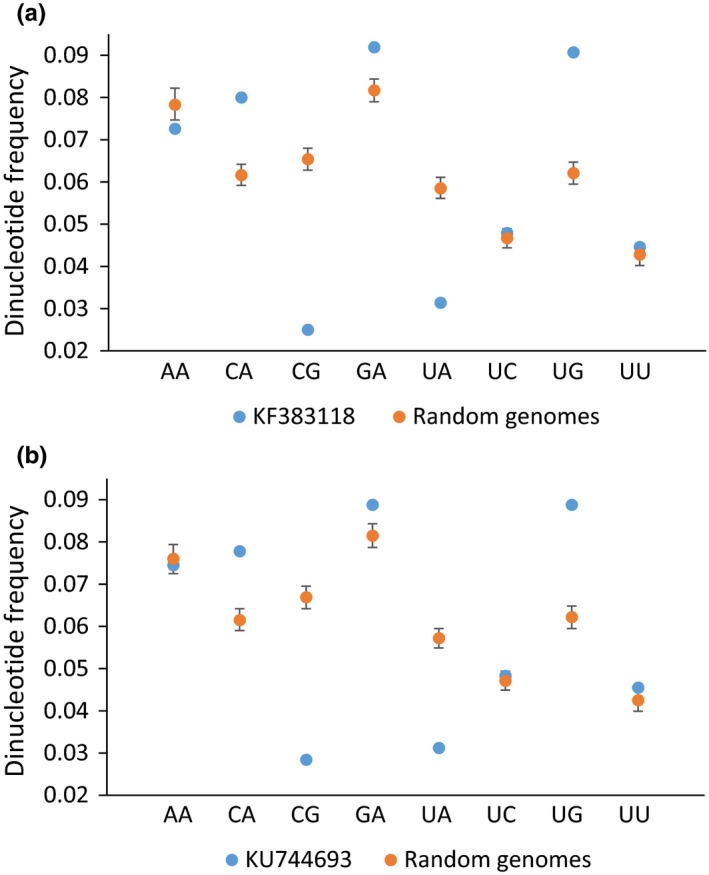
Dinucleotide frequencies for the two ZIKV references, KF383118 (a) and KU744693 (b). Blue dots refer to the observed frequencies for NA and UN, as well as for the widely deficient CG (Karlin & Burge, 1995; Lobo et al., 2009). Orange dots and their bars correspond to the means and 95% confidence intervals for their null distributions, respectively, as estimated with the 1,000 random genomes for each reference

The two ZIKV references (KF383118 and KU744693) are both enriched for GA (Figure 2). Their observed GA frequencies of 0.092 and 0.089, respectively, are consistently greater than those for all 1000 of their random sequences (thus, *p* < .002 in each case with two‐tailed testing). Conversely, neither is enriched for UC, which is the complement of GA (observed frequencies of 0.048 for both references; *p* = .284 and .316, respectively). KF383118 and KU744693 are also enriched for CA and its complement, UG (observed frequencies of 0.080/0.078 and 0.091/0.089), but deficient for CG and UA (0.025/0.028 and 0.031/0.031), respectively (*p* < .002 in every case). These results are consistent with the dinucleotide frequencies for all 56 ZIKV genomes, whose estimates vary within narrow ranges (Table S2). The dinucleotide frequency analyses document that weak GA is enriched on only the positive strand, whereas strong CA is overrepresented on both the positive and negative strands. Conversely, they show that the ZIKV genome is deficient for both CG and UA.

### 3.5 Spatial clustering of potential ADAR substitutions

The rooted ML phylogeny consists of 56 external and 52 internal branches (not counting the two basal internodes for which substitutions remain undirected; Figure 1). Of these 108 external and internal branches, 22 are associated with more than eight A → G and U → C for testing the spatial clustering of potential ADAR substitutions. Of these 22 branches, significant spatial clustering is found for three that correspond to one internal (I) and two external (II and III) internodes of the African group. The observed median minimum absolute distances for branches I, II, and III are 9, 14, and 24 bases, respectively, and these estimates are consistently less than those for all 1000 of their random permutations. Thus, *p* is <.002 for all three branches, which supports the significance of their spatial clustering of potential ADAR substitutions even after conservative Bonferroni correction (i.e., after α is reduced from .05 to .05/22 = .0023).

**Figure 3:**
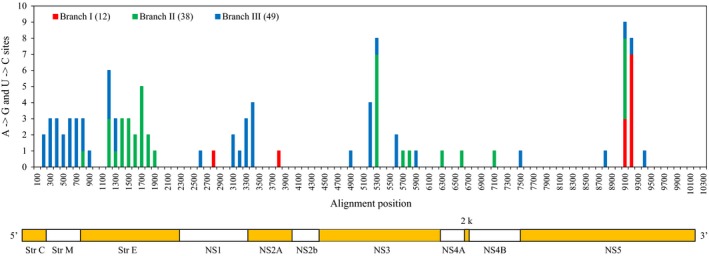
Locations of potential ADAR substitutions for the three branches (I–III) with significant spatial clustering (Figure 1a). These locations are summarized according to the multiple sequence alignment (Figure S1) and the gene structure of the ZIKV genome (Pickett et al., 2012). For each branch, the total number of potential ADAR substitutions is provided in parentheses

Two different sets of six potential ADAR substitutions for branch III are separately clumped among alignment positions 225–357 and 3063–3231 (133 and 169 sites) of the Str C and NS1 genes, respectively (Figure 3). All 12 of these potential ADAR substitutions, except for one Str C change, are at third codon positions, which thereby indicates that each of their two separate clusters is most likely due to some common mutational event (i.e., ADAR editing) rather than an episode of selection for correlated amino acid replacements in the encoded protein. Comparable cases of clustering are also evident for branches I and II [e.g., ten and six potential ADAR substitutions for I and II are clumped among alignment positions 9066–9147 and 1608–1698 (82 and 91 sites) of the NS5 and Str E genes, respectively, and all but two of these 16 changes (two in NS5) are at third codon positions]. Thus, all three branches offer unique and compelling cases of A → G and U → C spatial clustering, which are consistent with ADAR editing.

Internal branch I is supported by a weak bootstrap score of 53%, which thereby questions the reliability of its spatial clustering and that for its direct descendant II (Figure 1a). Critical, then, is our finding that the signals of spatial clumping for branches I and II remain even after the deletion of KF383119 from the substitution and clustering analyses. The deletion of KF383119 results in the removal of the weak internal branch I that unites this genome to KF383118. Crucially, as for the original branches I and II (Figure 3), the spatial clustering for the newly elongated external internode to KF383118 is also significant as documented by its *p* < .002 and its multiple clumps of potential ADAR substitutions in the NS5 and Str E genes.

### 3.6 ZIKV and host codon preferences

A codon set is defined as those triplets that share the same nucleotide and R (A/G) redundancy at their second and third codon positions, respectively. For example, UUR and CUR for leucine and GUR for valine are combined into the same codon set (NUR) on the basis of their shared U and A/G redundancy at their second and third codon positions, respectively (Table 3). Conversely, AUR is not included in this set, because it lacks A/G redundancy at its third codon positions (i.e., AUA codes for isoleucine, whereas AUG specifies methionine).

**Table 3:** Usage of synonymous A‐ and G‐ending codons by the two references, KF383118 (A) and KU744693 (B). A codon set is defined as those triplets that share the same base and R (A/G) redundancy at their second and third codon positions, respectively. A summary of synonymous A‐ and G‐ending codon usage by all 56 ZIKV genomes (which is consistent with the trends for KF383118 and KU744693) is provided in Table S2

	Encoded amino acids	Synonymous codons		Totals (%A‐ending)
		A‐ending	G‐ending	
(A) Codon sets for KF383118				
NUR	L and V	81	284	365 (22.2%)
NCR	S, P, T, and A	295	71	366 (80.6%)
NAR	Q, K, and E	217	269	486 (44.7%)
NGR	R and G	251	145	396 (63.4%)
Totals		844	769	1613 (52.3%)
(B) Codon sets for KU744693				
NUR	L and V	74	278	352 (21.0%)
NCR	S, P, T, and A	274	88	362 (75.7%)
NAR	Q, K, and E	228	256	484 (47.1%)
NGR	R and G	229	165	394 (58.1%)
Totals		805	787	1592 (50.6%)

For the two ZIKV references (KF383118 and KU744693), the usage of synonymous A‐ and G‐ending triplets differs significantly among the four codon sets (Table 3; $\chi_{c}^{2}$ = 277.970 and 223.02, respectively; *df* = 3, *p* < .001 for both). For both references, A‐ending codons are more frequently used than are G‐ending triplets by NCR (%A‐ending of 80.6% and 75.7%) and NGR (63.4% and 58.1%), whereas the opposite is true for NAR (44.7% and 47.1%) and NUR (22.2% and 21.0%), respectively. The greater usage of A‐ and G‐ending codons by NGR and NAR is consistent with the weak and strong preferences of ADAR for NGA and NAA, respectively. However, the greater usage of A‐ending codons by NCR is once again inconsistent with the status of its NCA as a strong ADAR target.

**Figure 4:**
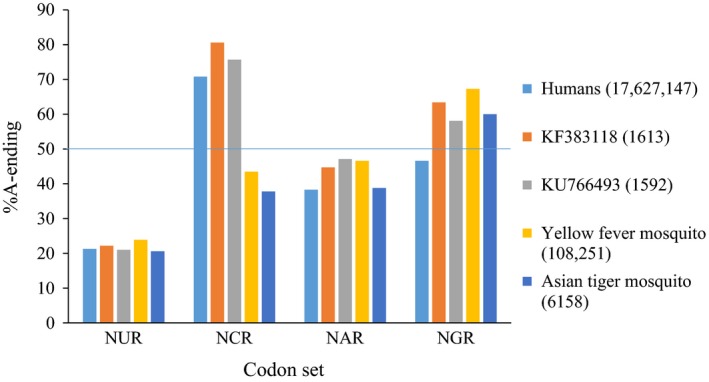
Percent usage of A‐ending codons by the two ZIKV references and their human and mosquito hosts. These percentages are presented on a per‐codon‐set basis (with %A‐ending and codon set defined as in Table 3). The thin blue horizontal line highlights the 50% breakpoint, whereby the relative usage of synonymous A‐ and G‐ending codons shifts from the latter to the former. The total number of synonymous A‐ and G‐ending codons is given in parentheses for each ZIKV and host

Similar to the two ZIKV references, the human and mosquito hosts also rely to a greater degree on G‐ending codons for NAR (%A‐ending of 38.3% and 38.8%–46.6%) and NUR (21.3% and 20.6%–23.9%), respectively (Figure 4). Conversely, humans (70.8%) depend to a greater extent on the A‐ending codons of NCR (as does ZIKV), but on the G‐ending triplets (46.6%) of NGR (unlike the virus). The reverse is true for the mosquitos, who prefer the G‐ending codons of NCR (37.8%–43.5%), but the A‐ending triplets of NGR (60.0%–67.3%). The preference of ZIKV for the A‐ending codons of both NCR and NGR illustrates an additional way by which the virus “compromises” between the different codon usages (and thereby, presumably the cognate tRNA pools) of its human and mosquito hosts [i.e., rather than lowering the bias for both NCR and NGR (Cristina, Fajardo, Soñora, Moratorio, & Musto, 2016; Jenkins & Holmes, 2003), ZIKV “splits the difference” such that its NCR usage matches that of one host, while its NGR preference overlaps with that of the other].

## 4 Discussion

### 4.1 ADAR editing is a factor of ZIKV mutagenesis and evolution

Recently, Cristina et al. (2016) and van Hemert and Berkhout (2016) separately concluded that the base, dinucleotide, and codon compositions of ZIKV are interrelated and that mutation pressure is the primary force underlying its codon usage. In light of their conclusions, we now hypothesize that host ADAR editing is one of the factors that contributes to this mutation pressure. Our hypothesis is premised on four different lines of evidence. (1) GA is conserved on both the positive and negative strands (Table 2). As GA is a weak target of ADAR (Eggington et al., 2011), this conservation is a signature of biased ADAR mutagenesis. (2) Weak GA is enriched on the positive strand, but not on the negative sequence (Figure 2). Coupled with the greater conservation of GA on the positive strand (Table 2), this inequality indicates that the positive sequence (which functions as both the genome and mRNA of ZIKV) is under greater purifying selection than the negative complement (Piontkivska et al., 2016). (3) A → G and U → C for three branches of the ML phylogeny are spatially clustered (Figures 1 and 3). Such spatial clustering and temporal clustering are diagnostic of how ADAR introduces mutations into double‐stranded RNA (Carpenter et al., 2009; Eggington et al., 2011). (4) The contradictory preferences of ZIKV for A‐ and G‐ending codons of NGR and NAR, respectively, are consistent with the opposing ADAR‐resistant and ‐susceptible status of their NGA and NAA, respectively (Table 3 and Figure 4).

The molecular evolution of biological sequences is a complex process that is driven by many different mutation and selection forces (Graur, 2016). Thus, it is not surprising that our comparisons of ZIKV genomes reveal the signatures of other factors in addition to ADAR editing. In particular, we find that CA is conserved and enriched on both the positive and negative strands and that A‐ending codons are preferred by NCR (Tables 2 and 3 and Figures 2 and 4). As a strong ADAR target, these trends cannot be explained by ADAR editing, but can be related instead to the widespread CG and UA deficits that exist among different viruses (including ZIKV), bacteria, and eukaryotes (Karlin & Burge, 1995; Lobo et al., 2009). Specifically, the fast transition rates of A → G (i.e., as introduced by ADAR) and C → U convert CA → CG and CA → UA, respectively (Graur, 2016). However, for viruses, both CG and UA can be deleterious as the former is recognized by the host innate immune system as a pathogen tag (Dorn & Kippenberger, 2008; Kawai & Akira, 2006), whereas the latter constitutes a potential source of spurious UA‐rich regulatory elements and premature stop codons (Cristina et al., 2016; Karlin & Burge, 1995). Thus, despite being a strong ADAR target, purifying selection opposes the fixation of CA → CG ADAR edits, which thereby leads to CA conservation, enrichment, and preference for NCA codons. These trends are then bolstered by additional purifying selection against the fixation of CA → CG and CA → UA transitions due to other mutation factors.

### 4.2 ADAR editing and positive‐ /negative‐sense RNA viruses

Recently, Piontkivska et al. (2016) provided comparable evidence that ADAR editing shapes the genomic evolution of DMelSV, which is a pathogen of the fruit fly (*Drosophila melanogaster*). DMelSV is a negative‐strand RNA virus (Rhabdoviridae), and thus, the two studies collectively document that ADAR editing can be a mutational and evolutionary factor of both positive and negative single‐stranded RNA viruses. However, one difference between the previous study and this study is that the positive strand of DMelSV is borderline significantly enriched for UC, whereas no such enrichment is found for ZIKV (Figure 2). Correspondingly, this difference means that contrary to ZIKV, the negative strand of DMelSV is also borderline enriched for resistant GA, which thereby suggests that purifying selection is operating to a greater extent on its negative complement. One possible explanation for this discrepancy is that the negative strand of DMelSV serves as its genome and is thereby under greater functional constraint than the negative strand of ZIKV.

Potential ADAR substitutions of DMelSV are significantly clustered for genes P and X, but not for G, M, and N (Piontkivska et al., 2016). Similarly, potential ADAR substitutions of ZIKV are significantly clumped for three branches of the African group, but not for 19 other internodes of its phylogeny (Figures 1 and 3). As posited for DMelSV, the lack of more widespread spatial clustering for ZIKV can now be attributed to the superimposition of the scattered transitions for other mutation factors, which thereby reduces the statistical power to detect the spatially clumped changes of ADAR editing. This argument serves as an additional reminder that molecular evolution is a complex process, which is influenced by many different interacting factors (Graur, 2016).

### 4.3 Future directions

A primary purpose of our hypothesis (i.e., host ADAR editing is a contributing factor of ZIKV mutagenesis and evolution) is to facilitate the development of novel ideas, to assist in the design of rigorous experiments, and to foster the generation of new comprehensive datasets. Toward these goals, our study now calls for mutation accumulation experiments that will allow for the direct observation of changes as they occur during the evolution of ZIKV (Barrick & Lenski, 2013; Duffy, Shackelton, & Holmes, 2008). These studies in experimental evolution will provide a critical reality check on our current overall trends of ZIKV change from in silico inferences. Both experimental and bioinformatic information will then be needed for additional Flaviviridae and other RNA (positive‐sense, negative‐strand, and double‐stranded) viruses. Such a comprehensive database of experimental and bioinformatic information for diverse RNA viruses will allow for rigorous tests about the limits of host ADAR editing and its interactions with different viral groups that vary in their genomic organizations and functions, life cycles, and modes of transmission.

At this time, we favor the argument that the primary substrate of ADAR editing is the double‐stranded RNA intermediates that form during viral replication and transcription rather than the double‐stranded stems of their folded RNA secondary structure (Carpenter et al., 2009; Khrustalev, Khrustaleva, Sharma, & Giri, 2017). This preference is based on the fact that GA is enriched to a greater degree than its complement (UC) on the positive strands of both ZIKV and DMelSV (Figure 2). This inequality indicates that the double‐stranded stems of the folded RNA are not the primary target of ADAR as a balanced overabundance of GA and UC is needed to maintain their internal base pairing (Piontkivska et al., 2016). Importantly, this bioinformatic finding is corroborated by experimental evidence, which shows that the adenosines of longer double‐stranded RNAs with perfect base pairing (i.e., as found during viral replication and transcription) are more likely to be extensively edited than are those of shorter duplex RNAs with mismatched and unmatched bases (e.g., as common for the stems of folded RNA) (Eggington et al., 2011). To rigorously test this argument for ZIKV, an experimentally determined secondary structure for its folded RNA genome is now needed to evaluate whether our current set of potential ADAR substitutions is concentrated (or not as we would predict) in its double‐stranded stems.

RNA methylation of the ZIKV genome by human host methyltransferases and demethylases was recently shown to regulate viral replication during infection (Lichinchi et al., 2016). However, as a consequence of these RNA edits, the functions of the host methyltransferases and demethylases were altered, which thereby led to modifications of the human host RNAs and target genes as well. Our findings that ADAR editing is a contributing factor of ZIKV mutagenesis and evolution highlight another mechanism by which the human host editome modifies the RNA of this pathogen. We now call for studies that investigate the intriguing possibility that host ADAR editing may also be related to the pathogenicity of ZIKV.

## Conflict of Interest

None declared.
