# Supplementary material for: RNA editing by the host ADAR system affects the molecular evolution of the Zika virus
Source: Ecol Evol. 2017 May 15;7(12):4475–85. doi: 10.1002/ece3.3033 (PMC5478085; doi:10.1002/ece3.3033)
Supplement: Supplementary file 2 [file ECE3-7-4475-s002.docx]

**Supplementary Table 2**

Base compositions at first, second, third, and all codon positions (A), dinucleotide frequencies of NA, UN, and CG (B), and codon usages (i.e., %A-ending synonymous codons) for all 56 ZIKV genomes. These estimates for all 56 ZIKV genomes are summarized as their means and ranges (in parentheses).

1. Base compositions

| First codon position | | | | | Second codon position | | | | | Third codon position | | | | | All codon positions | | | | |
| --- | --- | --- | --- | --- | --- | --- | --- | --- | --- | --- | --- | --- | --- | --- | --- | --- | --- | --- | --- |
| U | C | A | G | %R | U | C | A | G | %R | U | C | A | G | %R | U | C | A | G | %R |
| 0.163  (0.160-  0.164) | 0.172  (0.169-  0.173) | 0.305  (0.304-  0.307) | 0.360  (0.359-  0.364) | 66.5%  (66.4-  66.8%) | 0.285  (0.283-  0.287) | 0.229  (0.227-  0.230) | 0.265  (0.264-0.266) | 0.222  (0.220-  0.223) | 48.6%  (48.5-  48.7%) | 0.198  (0.195-  0.205) | 0.255  (0.244-0.258) | 0.254  (0.249-0.267) | 0.293  (0.282-0.298) | 54.7%  (54.5-55.3%) | 0.215 (0.214-  0.218) | 0.219 (0.214-  0.220) | 0.275 (0.273-  0.279) | 0.292 (0.288-  0.294) | 56.6%  (56.5-  56.9%) |

1. Dinucleotide frequencies

| AA | CA | CG | GA | UA | UC | UG | UU |
| --- | --- | --- | --- | --- | --- | --- | --- |
| 0.074 (0.071-0.075) | 0.078 (0.077-0.081) | 0.028 (0.023-0.029) | 0.089 (0.088-0.093) | 0.031 (0.030-0.032) | 0.048 (0.046-0.048) | 0.089 (0.088-0.092) | 0.046 (0.045-0.049) |

1. Codon usages

| Codon sets | %A-ending synonymous codons |
| --- | --- |
| NUR | 21.6% (20.5-22.8%) |
| NCR | 76.4% (73.2-81.9%) |
| NAR | 46.9% (42.1-48.2%) |
| NGR | 59.0% (57.2-66.7%) |
| Totals | 50.9% (49.7-53.7%) |
