# Supplementary material for: RNA editing by the host ADAR system affects the molecular evolution of the Zika virus
Source: Ecol Evol. 2017 May 15;7(12):4475–85. doi: 10.1002/ece3.3033 (PMC5478085; doi:10.1002/ece3.3033)
Supplement: Supplementary file 3 [file ECE3-7-4475-s003.pdf]

Supplementary Materials

Piontkivska, Frederick, Miyamoto and Wayne

RNA editing by the host ADAR system affects the molecular evolution of the Zika virus

Supplementary Table 1. GenBank accession numbers of ZIKV sequences used in the analyses, with collection and host information as per the GenBank, the Virus Pathogen Resource (viprbrc.org) and other available annotations.

Supplementary Table 2. Base compositions at first, second, third, and all codon positions (A), dinucleotide frequencies of NA, UN, and CG (B), and codon usages (i.e., %A-ending synonymous codons) for all 56 ZIKV genomes. These estimates for all 56 ZIKV genomes are summarized as their means and ranges (in parentheses).

Supplementary Figure 1. Multiple sequence alignment for the 56 ZIKV genomes. Sequences are identified with their respective GenBank accession numbers. Dashes ("-") refer to gaps, whereas "?" correspond to missing data.

Supplementary Table 1. GenBank accession numbers of ZIKV sequences used in the analyses, with collection and host information as per the GenBank, the Virus Pathogen Resource (viprbrc.org) and other available annotations.

Part A. 56 ZIKV sequences included in the analyses

GenBank accession number	Strain Name	Collection Date*	GenBank Host	Country	Notes
EU545988	FSM	06/2007	Homo sapiens	Micronesia	
KF268948	ARB13565	1976	Aedes africanus	Central African Republic	
KF268949	ARB15076	1980	Aedes opok	Central African Republic	**Per Berthet et al., 2014**
KF268950	ARB7701	1976	Aedes africanus	Central African Republic	**Per Berthet et al., 2014**
KF383115	ArB1362	1968	Aedes africanus	Central African Republic	**Per Faye et al., 2014 (Suppl.Table 1)**
KF383116	ArD7117	1968	Aedes luteocephalus	Senegal	**Per Faye et al., 2014 (Suppl.Table 1)**
KF383117	ArD128000	1997	Aedes luteocephalus	Senegal	**Per Faye et al., 2014 (Suppl.Table 1)**
KF383118	ArD157995	2001	Aedes dalzieli	Senegal	**Per Faye et al., 2014 (Suppl.Table 1)**
KF383119	ArD158084	2001	Aedes dalzieli	Senegal	**Per Faye et al., 2014 (Suppl.Table 1)**
KJ776791	H/PF/2013	11/28/2013	Homo sapiens	French Polynesia	
KU312312	Z1106033	10/02/2015	Homo sapiens	Suriname	
KU321639	ZikaSPH2015	03/2015	Homo sapiens	Brazil	
KU365777	BeH818995	2015	Homo sapiens	Brazil	
KU365778	BeH819015	2015	Homo sapiens	Brazil	
KU365779	BeH819966	2015	Homo sapiens	Brazil	
KU365780	BeH815744	2015	Homo sapiens	Brazil	
KU497555	Brazil-ZKV2015	11/30/2015	Homo sapiens	Brazil	
KU501215	PRVABC59	12/01/2015	Homo sapiens	Puerto Rico	
KU501216	103344	12/01/2015	Homo sapiens	Guatemala	
KU501217	8375	11/01/2015	Homo sapiens	Guatemala	
KU509998	Haiti/1225/201	12/12/2014	Homo sapiens	Haiti	
KU527068	Natal RGN	2015	Homo sapiens	Brazil	
KU707826	SSABR1	07/01/2015	Homo sapiens	Brazil	
KU729217	BeH823339	2015	Homo sapiens	Brazil	
KU729218	BeH828305	2015	Homo sapiens	Brazil	
KU740184	GD01	02/2016	Homo sapiens	China	
KU744693	VE_Ganxian	02/06/2016	Homo sapiens	China	
KU758877	17271	12/2015	Homo sapiens	French Guiana	
KU761564	GDZ16001	02/12/2016	Homo sapiens	China	
KU820898	GZ01	02/14/2016	Homo sapiens	China	
KU820899	ZJ03	02/17/2016	Homo sapiens	China	
KU853012	Dominican Rep	02/01/2016	Homo sapiens	Italy	
KU866423	Zika virus/SZ01	2016	Homo sapiens	China	
KU922923	MEX/InDRE/Lm	02/25/2016	Homo sapiens	Mexico	
KU922960	MEX/InDRE/Sm	02/25/2016	Homo sapiens; female	Mexico	
KU940224	Bahia09	08/01/2015	Homo sapiens	Brazil	
KU940228	Bahia07	07/01/2015	Homo sapiens	Brazil	
KU955589	Z16006	02/16/2016	Homo sapiens	China	
KU955590	Z16019	02/26/2016	Homo sapiens	China	
KU991811	Brazil/2016/INI	03/06/2016	Homo sapiens	Italy	
KX056898	Zika virus/GZ02	02/25/2016	Homo sapiens	China	
KX117076	Zhejiang04	02/17/2016	Homo sapiens	China	
KX185891	Zika virus/CN/S	02/17/2016	Homo sapiens	China	
KX247646	Zika virus/Hom	02/09/2016	Homo sapiens	Colombia	
KX262887	103451	01/06/2016	Homo sapiens	Honduras	
KX280026	Paraiba_01	2015	Homo sapiens	Brazil	
KX369547	PF13/251013-1	10/25/2013	Homo sapiens	French Polynesia	
KX377336	P6-740	07/1966	Aedes aegypti (mosqu	Malaysia	
KX377337	PRVABC-59	12/2015	Homo sapiens	Puerto Rico	
KX548902	ZIKV/COL/FCC0	10/07/2015	Homo sapiens	Colombia	
KX601166	ZIKV/Aedes afri	11/17/1984	Aedes africanus	Senegal	
KX601167	ZIKV/Aedes sp.,	07/14/1966	Aedes sp.	Malaysia	
KX601168	ZIKV/Homo sap	12/01/2015	Homo sapiens	Puerto Rico	
KX766028	R114916	06/06/2016	Homo sapiens	Dominican Republic	
KX766029	R116265	06/23/2016	Homo sapiens	Mexico	
NC_012532	MR 766	1947	sentinel monkey	Uganda	**Per Kuno et al. 2007**

Part B. Additional 59 ZIKV sequences used in the preliminary analyses

GenBank accession number	Strain Name	Collection	GenBank Host	Country	Notes
DQ859059	MR 766	1947	sentinel monkey	Uganda	**Per Kuno et al. 2007**
HQ234498	MR_766	1947	sentinel rhesus	Uganda	
HQ234499	P6-740	1966	Aedes aegypti	Malaysia	
HQ234500	IbH_30656	1968	Homo sapiens	Nigeria	
HQ234501	ArD_41519	1984	Aedes africanus	Senegal	
JN860885	FSS13025	2010	Homo sapiens	Cambodia	
KF383121	ArD158095	-N/A-	-N/A-	-N/A-	
KF993678	PLCal_ZV	02/19/201	Homo sapiens	Canada	
KU647676	MRS_OPY_Martinique_PaRi_2015	12/2015	Homo sapiens	Martinique	
KU681081	Zika virus/H.sapiens-tc/THA/2014/SV0127- 14	07/19/201	Homo sapiens	Thailand	
KU681082	Zika virus/H.sapiens-tc/PHL/2012/CPC-0740	05/09/201	Homo sapiens	Philippines	
KU720415	MR 766	1947	sentinel monkey	Uganda	**Per Kuno et al. 2007**
KU820897	FLR	12/2015	Homo sapiens	Colombia	
KU853013	Dominican Republic/2016/PD2	02/01/201	Homo sapiens	Italy	
KU870645	FB-GWUH-2016	02/02/201	Homo sapiens	USA	
KU926309	Rio-U1	01/14/201	Homo sapiens	Brazil	
KU926310	Rio-S1	01/29/201	Homo sapiens	Brazil	
KU937936	ZIKVNL00013	02/11/201	Homo sapiens	Suriname	
KU955591	Zika virus/A.africanus-tc/SEN/1984/41525-DAK	11/20/198	Aedes africanus	Senegal	
KU955592	Zika virus/A.taylori-tc/SEN/1984/41662-DAK	12/06/198	Aedes taylori	Senegal	
KU955593	Zika virus/H.sapiens-tc/KHM/2010/FSS13025	2010	Homo sapiens	Cambodia	
KU955594	Zika virus/M.mulatta-tc/UGA/1947/MR-766	04/1947	Macaca mulatta	Uganda	
KU955595	Zika virus/A.taylori-tc/SEN/1984/41671-DAK	12/14/198	Aedes taylori	Senegal	
KU963573	ZIKV/Macaca mulatta/UGA/MR-766_SM150-V8/1947	04/20/194	Macaca mulatta	Uganda	
KU963574	ZIKV/Homo sapiens/NGA/IbH-30656_SM21V1-V3/1968	09/09/196	Homo sapiens	Nigeria	
KU963796	SZ-WIV01	2016	Homo sapiens	China	
KX051563	Haiti/1/2016	02/05/201	Homo sapiens	USA	
KX087101	ZIKV/Homo sapiens/PRI/PRVABC59/2015	12/2015	Homo sapiens	Puerto Rico	
KX087102	ZIKV/Homo sapiens/COL/FLR/2015	12/2015	Homo sapiens	Colombia	
KX156774	ZIKV/Homo sapiens/PAN/CDC-259359_V1-V3/2015	12/18/201	Homo sapiens	Panama	
KX156775	ZIKV/Homo sapiens/PAN/CDC-259249_V1-V3/2015	12/11/201	Homo sapiens	Panama	
KX156776	ZIKV/Homo sapiens/PAN/CDC-259364_V1-V2/2015	12/18/201	Homo sapiens	Panama	
KX197192	ZIKV/H.sapiens/Brazil/PE243/2015	2015	Homo sapiens	Brazil	
KX198134	ZIKV/Aedes africanus/SEN/DAK-AR-41524_A1C1-V2/1984	11/17/198	Aedes africanus	Senegal	
KX198135	ZIKV/Homo sapiens/PAN/BEI-259634_V4/2016	2016	Homo sapiens	Panama	
KX247632	MEX_I_7	11/2015	Homo sapiens	Mexico	
KX253996	ZKC2/2016	02/16/201	Homo sapiens	China	
KX266255	ZIKV_SMGC-1	02/14/201	Homo sapiens	China	
KX377335	MR-766	04/1947	Macaca mulatta (monkey	Uganda	
KX446950	ZIKV/Aedes.sp/MEX/MEX_2-81/2016	01/01/201	Aedes sp.	Mexico	
KX446951	ZIKV/Aedes.sp/MEX/MEX_I-7/2016	01/01/201	Aedes sp.	Mexico	
KX447509	1_0087_PF	12/2013	Homo sapiens	French Polynesia	
KX447510	1_0049_PF	12/2013	Homo sapiens	French Polynesia	
KX447511	1_0015_PF	01/2014	Homo sapiens	French Polynesia	
KX447512	1_0181_PF	12/2013	Homo sapiens	French Polynesia	
KX447513	1_0134_PF	12/2013	Homo sapiens	French Polynesia	
KX447514	1_0035_PF	01/2014	Homo sapiens	French Polynesia	
KX447515	1_0030_PF	11/2013	Homo sapiens	French Polynesia	
KX447516	1_0111_PF	01/2014	Homo sapiens	French Polynesia	
KX447517	1_0038_PF	01/2014	Homo sapiens	French Polynesia	
KX520666	HS-2015-BA-01	08/2015	Homo sapiens; female	Brazil	
KX601169	ZIKV/Macaca mulatta/UGA/MR-766/1947	04/20/194	Macaca mulatta	Uganda	
KX673530	PHE_semen_Guadeloupe	04/21/201	Homo sapiens	United Kin	**Per Atkinson et al., 2016**
KX694532	ZIKV/Homo sapiens/THA/PLCal_ZV/2013	02/12/201	Homo sapiens; female	Thailand	
KX694533	ZIKV/Aedes aegypti/MYS/P6-740/1966	07/14/196	Aedes sp.	Malaysia	
KX694534	ZIKV/Homo sapiens/HND/R103451/2015	01/06/201	Homo sapiens	Honduras	
KX702400	Zika virus/Homo sapiens/VEN/UF-1/2016	03/25/201	Homo sapiens; lactating f	Venezuela	
LC002520	MR766-NIID	-N/A-	-N/A-	Uganda	
KF383120	ArD142623	2000	Anopheles coustani	Senegal	**Per Faye et al., 2014 (Suppl.Table 1)**

**** Collection date is given as per the respective GenBank record; and if no information is available, then other sources are used.***

Atkinson, B., V. Graham, R. W. Miles, K. Lewandowski, S. D. Dowall, S. T. Pullan and R. Hewson (2016). "Complete Genome Sequence of Zika Virus Isolated from Semen." Genome Announc 4(5).

Berthet, N., E. Nakoune, B. Kamgang, B. Selekon, S. Descorps-Declere, A. Gessain, J. C. Manuguerra and M. Kazanji (2014). "Molecular characterization of three Zika flaviviruses obtained from sylvatic mosquitoes in the Central African Republic." Vector Borne Zoonotic Dis 14(12): 862-865.

Faye, O., C. C. Freire, A. Iamarino, J. V. de Oliveira, M. Diallo, P. M. Zanotto and A. A. Sall (2014). "Molecular evolution of Zika virus during its emergence in the 20(th) century." PLoS Negl Trop Dis 8(1): e2636.

Kuno, G. and G. J. Chang (2007). "Full-length sequencing and genomic characterization of Bagaza, Kedougou, and Zika viruses." Arch Virol 152(4): 687-696.

Supplementary Table 2

Base compositions at first, second, third, and all codon positions (A), dinucleotide frequencies of NA, UN, and CG (B), and codon usages (i.e., %A-ending synonymous codons) for all 56 ZIKV genomes. These estimates for all 56 ZIKV genomes are summarized as their means and ranges (in parentheses).

A) Base compositions

First codon position					Second codon position					Third codon position					All codon positions				
U	C	A	G	%R	U	C	A	G	%R	U	C	A	G	%R	U	C	A	G	%R
0.163 (0.160-0.164)	0.172 (0.169-0.173)	0.305 (0.304-0.307)	0.360 (0.359-0.364)	66.5% (66.4-66.8%)	0.285 (0.283-0.287)	0.229 (0.227-0.230)	0.265 (0.264-0.266)	0.222 (0.220-0.223)	48.6% (48.5-48.7%)	0.198 (0.195-0.205)	0.255 (0.244-0.258)	0.254 (0.249-0.267)	0.293 (0.282-0.298)	54.7% (54.5-55.3%)	0.215 (0.214-0.218)	0.219 (0.214-0.220)	0.275 (0.273-0.279)	0.292 (0.288-0.294)	56.6% (56.5-56.9%)

B) Dinucleotide frequencies

AA	CA	CG	GA	UA	UC	UG	UU
0.074 (0.071-0.075)	0.078 (0.077-0.081)	0.028 (0.023-0.029)	0.089 (0.088-0.093)	0.031 (0.030-0.032)	0.048 (0.046-0.048)	0.089 (0.088-0.092)	0.046 (0.045-0.049)

C) Codon usages

Codon sets	%A-ending synonymous codons
NUR	21.6% (20.5-22.8%)
NCR	76.4% (73.2-81.9%)
NAR	46.9% (42.1-48.2%)
NGR	59.0% (57.2-66.7%)
Totals	50.9% (49.7-53.7%)

Supplementary Fig. 1. Multiple sequence alignment for the 56 ZIKV genomes. Sequences are identified with their respective GenBank accession numbers. Dashes ("-") refer to gaps, whereas "?" correspond to missing data.

```
                                                                                                               1111111111111111111111111111111111111111111111111111111111111111111111111111111111
                          1111111111222222222233333333334444444444555555555566666666667777777777888888888899999999990000000000111111111122222222223333333333444444444455555555556666666666777777777788
Sequence     1234567890123456789012345678901234567890123456789012345678901234567890123456789012345678901234567890123456789012345678901234567890123456789012345678901234567890123456789012345678901
-------------------------------------------------------------------------------------------------------------------------------------------------------------------------------------------------------

NC_012532    AUGAAAAACCCCAAAGAAGAAAUCCGGAGGAUCCGGAUUGUCAAUAUGCUAAAACGCGGAGUAGCCCGUGUAAACCCCUUGGGAGGUUUGAAGAGGUUGCCAGCCGGACUUCUGCUGGGUCAUGGACCCAUCAGAAUGGUUUUGGCGAUACUAGCCUUUUUGAGAUUUACAGCAAUCAAGC
KU922960     AUGAAAAACCCAAAAAAGAAAUCCGGAGGAUUCCGGAUUGUCAAUAUGCUAAAACGCGGAGUAGCCCGUGUGAGCCCCUUUGGGGGCUUGAAGAGGCUGCCAGCCGGACUUCUGCUGGGUCAUGGGCCCAUCAGGAUGGUCUUGGCGAUUCUAGCCUUUUUGAGAUUCACGGCAAUCAAGC
KU922923     AUGAAAAACCCAAAAAAGAAAUCCGGAGGAUUCCGGAUUGUCAAUAUGCUAAAACGCGGAGUAGCCCGUGUGAGCCCCUUUGGGGGCUUGAAGAGGCUGCCAGCCGGACUUCUGCUGGGUCAUGGGCCCAUCAGGAUGGUCUUGGCGAUUCUAGCCUUUUUGAGAUUCACGGCAAUCAAGC
KU820898     AUGAAAAACCCAAAAAAGAAAUCCGGAGGAUUCCGGAUUGUCAAUAUGCUAAAACGCGGAGUAGCCCGUGUGAGCCCCUUUGGGGGCUUGAAGAGGCUGCCAGCCGGACUUCUGCUGGGUCAUGGGCCCAUCAGGAUGGUCUUGGCGAUUCUAGCCUUUUUGAGAUUCACGGCAAUCAAGC
KU321639     AUGAAAAACCCAAAAAAGAAAUCCGGAGGAUUCCGGAUUGUCAAUAUGCUAAAACGCGGAGUAGCCCGUGUGAGCCCCUUUGGGGGCUUGAAGAGGCUGCCAGCCGGACUUCUGCUGGGUCAUGGGCCCAUCAGGAUGGUCUUGGCAAUUCUAGCCUUUUUGAGAUUCACGGCAAUCAAGC
KU820899     AUGAAAAACCCAAAAAAGAAAUCCGGAGGAUUCCGGAUUGUCAAUAUGCUAAAACGCGGAGUAGCCCGUGUGAGCCCCUUUGGGGGCUUGAAGAGGCUGCCAGCCGGACUUCUGCUGGGUCAUGGGCCCAUCAGGAUGGUCUUGGCGAUUCUAGCCUUCUUGAGAUUCACGGCAAUCAAGC
KU853012     AUGAAAAACCCAAAAAAGAAAUCCGGAGGAUUCCGGAUUGUCAAUAUGCUAAAACGCGGAGUAGCCCGUGUGAGCCCCUUUGGGGGCUUGAAGAGGCUGCCAGCCGGACUUCUGCUGGGUCAUGGGCCCAUCAGGAUGGUCUUGGCGAUUCUAGCCUUUUUGAGAUUCACGGCAAUCAAGC
KU729217     AUGAAAAACCCAAAAAAGAAAUCCGGAGGAUUCCGGAUUGUCAAUAUGCUAAAACGCGGAGUAGCCCGUGUGAGCCCCUUUGGGGGCUUGAAGAGGCUGCCAGCCGGACUUCUGCUGGGUCAUGGGCCCAUCAGGAUGGUCUUGGCGAUUCUAGCCUUUUUGAGAUUCACGGCAAUCAAGC
KU729218     AUGAAAAACCCAAAAAAGAAAUCCGGAGGAUUCCGGAUUGUCAAUAUGCUAAAACGCGGAGUAGCCCGUGUGAGCCCCUUUGGGGGCUUGAAGAGGCUGCCAGCCGGACUUCUGCUGGGUCAUGGGCCCAUCAGGAUGGUCUUGGCGAUUCUAGCCUUUUUGAGAUUCACGGCAAUCAAGC
KU761564     AUGAAAAACCCAAAAAAGAAAUCCGGAGGAUUCCGGAUUGUCAAUAUGCUAAAACGCGGAGUAGCCCGUGUGAGCCCCUUUGGGGGCUUGAAGAGGCUGCCAGCCGGACUUCUGCUGGGUCAUGGGCCCAUCAGGAUGGUCUUGGCGAUUCUAGCCUUCUUGAGAUUCACGGCAAUCAAGC
KU740184     AUGAAAAACCCAAAAAAGAAAUCCGGAGGAUUCCGGAUUGUCAAUAUGCUAAAACGCGGAGUAGCCCGUGUGAGCCCCUUUGGGGGCUUGAAGAGGCUGCCAGCCGGACUUCUGCUGGGUCAUGGGCCCAUCAGGAUGGUCUUGGCGAUUCUAGCCUUCUUGAGAUUCACGGCAAUCAAGC
KU744693     AUGAAAAACCCAAAAAAGAAAUCCGGAGGAUUCCGGAUUGUCAAUAUGCUAAAACGCGGAGUAGCCCGUGUGAGCCCCUUUGGGGGCUUGAAGAGGCUGCCAGCCGGACUUCUGCUGGGUCAUGGGCCCAUCAGGAUGGUCUUGGCAAUUCUAGCCUUUUUGAGAUUCACGGCAAUCAAGC
KU497555     AUGAAAAACCCAAAAAAGAAAUCCGGAGGAUUCCGGAUUGUCAAUAUGCUAAAACGCGGAGUAGCCCGUGUGAGCCCCUUUGGGGGCUUGAAGAGGCUGCCAGCCGGACUUCUGCUGGGUCAUGGGCCCAUCAGGAUGGUCUUGGCGAUUCUAGCCUUUUUGAGAUUCACGGCAAUCAAGC
KU527068     AUGAAAAACCCAAAAAAGAAAUCCGGAGGAUUCCGGAUUGUCAAUAUGCUAAAACGCGGAGUAGCCCGUGUGAGCCCCUUUGGGGGCUUGAAGAGGCUGCCAGCCGGACUUCUGCUGGGUCAUGGGCCCAUCAGGAUGGUCUUGGCAAUUCUAGCCUUUUUGAGAUUCACGGCAAUCAAGC
KU501217     AUGAAAAACCCAAAAAAGAAAUCCGGAGGAUUCCGGAUUGUCAAUAUGCUAAAACGCGGAGUAGCCCGUGUGAGCCCCUUUGGGGGCUUGAAGAGGCUGCCAGCCGGACUUCUGCUGGGUCAUGGGCCCAUCAGGAUGGUCUUGGCGAUUCUAGCCUUUUUGAGAUUCACGGCAAUCAAGC
KU501216     AUGAAAAACCCAAAAAAGAAAUCCGGAGGAUUCCGGAUUGUCAAUAUGCUAAAACGCGGAGUAGCCCGUGUGAGCCCCUUUGGGGGCUUGAAGAGGCUGCCAGCCGGACUUCUGCUGGGUCAUGGGCCCAUCAGGAUGGUCUUGGCGAUUCUAGCCUUUUUGAGAUUCACGGCAAUCAAGC
KU501215     AUGAAAAACCCAAAAAAGAAAUCCGGAGGAUUCCGGAUUGUCAAUAUGCUAAAACGCGGAGUAGCCCGUGUGAGCCCCUUUGGGGGCUUGAAGAGGCUGCCAGCCGGACUUCUGCUGGGUCAUGGGCCCAUCAGGAUGGUCUUGGCGAUUCUAGCCUUUUUGAGAUUCACGGCAAUCAAGC
KU365780     AUGAAAAACCCAAAAAAGAAAUCCGGAGGAUUCCGGAUUGUCAAUAUGCUAAAACGCGGAGUAGCCCGUGUGAGCCCCUUUGGGGGCUUGAAGAGGCUGCCAGCCGGACUUCUGCUGGGUCAUGGGCCCAUCAGGAUGGUCUUGGCGAUUCUAGCCUUUUUGAGAUUCACGGCAAUCAAGC
KU365779     AUGAAAAACCCAAAAAAGAAAUCCGGAGGAUUCCGGAUUGUCAAUAUGCUAAAACGCGGAGUAGCCCGUGUGAGCCCCUUUGGGGGCUUGAAGAGGCUGCCAGCCGGACUUCUGCUGGGUCAUGGGCCCAUCAGGAUGGUCUUGGCGAUUCUAGCCUUUUUGAGAUUCACGGCAAUCAAGC
KU707826     AUGAAAAACCCAAAAAAGAAAUCCGGAGGAUUCCGGAUUGUCAAUAUGCUAAAACGCGGAGUAGCCCGUGUGAGCCCCUUUGGGGGCUUGAAGAGGCUGCCAGCCGGACUUCUGCUGGGUCAUGGGCCCAUCAGGAUGGUCUUGGCGAUUCUAGCCUUUUUGAGAUUCACGGCAAUCAAGC
KU365778     AUGAAAAACCCAAAAAAGAAAUCCGGAGGAUUCCGGAUUGUCAAUAUGCUAAAACGCGGAGUAGCCCGUGUGAGCCCCUUUGGGGGCUUGAAGAGGCUGCCAGCCGGACUUCUGCUGGGUCAUGGGCCCAUCAGGAUGGUCUUGGCGAUUCUAGCCUUUUUGAGAUUCACGGCAAUCAAGC
KU365777     AUGAAAAACCCAAAAAAGAAAUCCGGAGGAUUCCGGAUUGUCAAUAUGCUAAAACGCGGAGUAGCCCGUGUGAGCCCCUUUGGGGGCUUGAAGAGGCUGCCAGCCGGACUUCUGCUGGGUCAUGGGCCCAUCAGGAUGGUCUUGGCGAUUCUAGCCUUUUUGAGAUUCACGGCAAUCAAGC
KU312312     AUGAAAAACCCAAAAAAGAAAUCCGGAGGAUUCCGGAUUGUCAAUAUGCUAAAACGCGGAGUAGCCCGUGUGAGCCCCUUUGGGGGCUUGAAGAGGCUGCCAGCCGGACUUCUGCUGGGUCAUGGGCCCAUCAGGAUGGUCUUGGCGAUUCUAGCCUUUUUGAGAUUCACGGCAAUCAAGC
KF268950     AUGAAAAACCCAAAGAAGAAAUCCGGAGGAUUCCGGAUUGUCAAUAUGCUAAAACGCGGAGUAGCCCGUGUAAACCCCUUGGGAGGUUUGAAGAGGCUGCCAGCCGGACUUCUGCUGGGUCAUGGACCCAUCAGAAUGGUUUUGGCAAUACUAGCCUUUUUGAGAUUUACAGCAAUCAAGC
KF268949     AUGAAAAACCCAAAGAAGAAAUCCGGAGGAUUCCGGAUUGUCAAUAUGCUAAAACGCGGAGUAGCCCGUGUAAACCCCUUGGGAGGUUUGAAGAGGCUGCCAGCCGGACUCCUGCUGGGUCAUGGACCCAUCAGAAUGGUUUUGGCAAUACUAGCCUUUUUGAGAUUUACAGCAAUCAAGC
KF268948     AUGAAAAACCCAAAGAAGAAAUCCGGAGGAUUCCGGAUUGUCAAUAUGCUAAAACGCGGAGUAGCCCGUGUAAACCCCUUGGGAGGUUUGAAGAGGCUGCCAGCCGGACUUCUGCUGGGUCAUGGACCCAUCAGAAUGGUUUUGGCAAUACUAGCCUUUUUGAGAUUUACAGCAAUCAAGC
KJ776791     AUGAAAAACCCAAAAAAGAAAUCCGGAGGAUUCCGGAUUGUCAAUAUGCUAAAACGCGGAGUAGCCCGUGUGAGCCCCUUUGGGGGCUUGAAGAGGCUGCCAGCCGGACUUCUGCUGGGUCAUGGGCCCAUCAGGAUGGUCUUGGCGAUUCUAGCCUUUUUGAGAUUCACGGCAAUCAAGC
KF383119     AUGAAAAACCCAAAGAAGAAAUCCGGAGGAUUCCGGAUUGUCAAUAUGCUAAAACGCGGAGUAGCCCGUGUAAACCCCUUGGGAGGUUUGAAGAGGUUGCCAGCCGGACUUCUGCUGGGUCAUGGACCCAUCAGAAUGGUUUUGGCGAUACUAGCCUUUUUGAGAUUUACAGCAAUCAAGC
KF383118     AUGAAAAACCCAAAGAAGAAAUCCGGAAGAUUCCGGAUUGUCAAUAUGCUAAAACGCGGAGUAGCCCGUGUAAACCCCUUGGGAGGUUUGAAGAGGUUGCCAGCCGGACUUCUGCUGGGUCAUGGACCCAUCAGAAUGGUUUUGGCGAUACUAGCCUUUUUGAGAUUUACAGCAAUCAAGC
KF383117     AUGAAAAACCCAAAGAGGAAAUCCGGAGGAUUCCGGAUUGUCAAUAUGCUAAAACGCGGAGUAGCCCGUGUAAACCCCUUGGGGGGUUUGAAGAGGCUGCCAGCCGGACUUCUGCUGGGUCAUGGACCCAUUAGAAUGGUUUUGGCGAUACUAGCCUUUUUGAGAUUUACAGCAAUCAAGC
KF383116     AUGAAAAACCCAAAGAAGAGAUCCGGAGGAUUCCGGAUUGUCAAUAUGCUAAAACGCGGAGUAGCCCGUGUAAACCCCUUGGGGGGUUUGAAGAGGCUGCCGGCCGGACUUCUGCUGGGCCAUGGACCCAUCAGAAUGGUUUUGGCGAUACUAGCCUUUUUGAGAUUCACAGCAAUCAAGC
KF383115     AUGAAAAACCCAAAGAAGAAAUCCGGAGGAUUCCGGAUUGUCAAUAUGCUAAAACGCGGAGUAGCCCGUGUAAACCCCUUGGGAGGUUUGAAGAGGCUGCCAGCCGGACUUCUGCUGGGUCAUGGACCCAUUAGAAUGGUUUUGGCGAUACUAGCCUUUUUGAGAUUUACAGCAAUCAAGC
EU545988     AUGAAAAACCCCAAAGAAGAAAUCCGGAGGAUCCGGAUUGUCAAUAUGCUAAAACGCGGAGUAGCCCGUGUGAGCCCCUUUGGGGGCUUGAAGAGGCUGCCAGCCGGACUUCUGCUGGGUCAUGGGCCCAUCAGGAUGGUCUUGGCGAUACUAGCCUUUUUGAGAUUCACGGCAAUCAAGC
KU955589     AUGAAAAACCCAAAAAAGAAAUCCGGAGGAUUCCGGAUUGUCAAUAUGCUAAAACGCGGAGUAGCCCGUGUGAGCCCCUUUGGGGGCUUGAAGAGGCUGCCAGCCGGACUUCUGCUGGGUCAUGGGCCCAUCAGGAUGGUCUUGGCGAUUCUAGCCUUCUUGAGAUUCACGGCAAUCAAGC
KU955590     AUGAAAAACCCAAAAAAGAAAUCCGGAGGAUUCCGGAUUGUCAAUAUGCUAAAACGCGGAGUAGCCCGUGUGAGCCCCUUUGGGGGCUUGAAGAGGCUGCCAGCCGGACUUCUGCUGGGUCAUGGGCCCAUCAGGAUGGUCUUGGCGAUUCUAGCCUUUUUGAGAUUCACGGCAAUCAAGC
KU940228     AUGAAAAACCCAAAAAAGAAAUCCGGAGGAUUCCGGAUUGUCAAUAUGCUAAAACGCGGAGUAGCCCGUGUGAGCCCCUUUGGGGGCUUGAAGAGGCUGCCAGCCGGACUUCUGCUGGGUCAUGGGCCCAUCAGGAUGGUCUUGGCGAUUCUAGCCUUUUUGAGAUUCACGGCAAUCAAGC
KU991811     AUGAAAAACCCAAAAAAGAAAUCCGGAGGAUUCCGGAUUGUCAAUAUGCUAAAACGCGGAGUAGCCCGUGUGAGCCCCUUUGGGGGCUUGAAGAGGCUGCCAGCCGGACUUCUGCUGGGUCAUGGGCCCAUCAGGAUGGUCUUGGCGAUUCUAGCCUUUUUGAGAUUCACGGCAAUCAAGC
KU509998     AUGAAAAACCCAAAAAAGAAAUCCGGAGGAUUCCGGAUUGUCAAUAUGCUAAAACGCGGAGUAGCCCGUGUGAGCCCCUUUGGGGGCUUGAAGAGGCUGCCAGCCGGACUUCUGCUGGGUCAUGGGCCCAUCAGGAUGGUCUUGGCAAUUCUAGCCUUUUUGAGAUUCACGGCAAUCAAGC
KX056898     AUGAAAAACCCAAAAAAGAAAUCCGGAGGAUUCCGGAUUGUCAAUAUGCUAAAACGCGGAGUAGCCCGUGUGAGCCCCUUUGGGGGCUUGAAGAGGCUGCCAGCCGGACUUCUGCUGGGUCAUGGGCCCAUCAGGAUGGUCUUGGCGAUUCUAGCCUUUUUGAGAUUCACGGCAAUCAAGC
KX117076     AUGAAAAACCCAAAAAAGAAAUCCGGAGGAUUCCGGAUUGUCAAUAUGCUAAAACGCGGAGUAGCCCGUGUGAGCCCCUUUGGGGGCUUGAAGAGGCUGCCAGCCGGACUUCUGCUGGGUCAUGGGCCCAUCAGGAUGGUCUUGGCGAUUCUAGCCUUCUUGAGAUUCACGGCAAUCAAGC
KX185891     AUGAAAAACCCAAAAAAGAAAUCCGGAGGAUUCCGGAUUGUCAAUAUGCUAAAACGCGGAGUAGCCCGUGUGAGCCCCUUUGGGGGCUUGAAGAGGCUGCCAGCCGGACUUCUGCUGGGUCAUGGGCCCAUCAGGAUGGUCUUGGCGAUUCUAGCCUUCUUGAGAUUCACGGCAAUCAAGC
KX247646     AUGAAAAACCCAAAAAAGAAAUCCGGAGGAUUCCGGAUUGUCAAUAUGCUAAAACGCGGAGUAGCCCGUGUGAGCCCCUUUGGGGGCUUGAAGAGGCUGCCAGCCGGACUUCUGCUGGGUCAUGGGCCCAUCAGGAUGGUCUUGGCGAUUCUAGCCUUUUUGAGAUUCACGGCAAUCAAGC
KX262887     AUGAAAAACCCAAAAAAGAAAUCCGGAGGAUUCCGGAUUGUCAAUAUGCUAAAACGCGGAGUAGCCCGUGUGAGCCCCUUUGGGGGCUUGAAGAGGCUGCCAGCCGGACUUCUGCUGGGUCAUGGGCCCAUCAGGAUGGUCUUGGCGAUUCUAGCCUUUUUGAGAUUCACGGCAAUCAAGC
KX280026     AUGAAAAACCCAAAAAAGAAAUCCGGAGGAUUCCGGAUUGUCAAUAUGCUAAAACGCGGAGUAGCCCGUGUGAGCCCCUUUGGGGGCUUGAAGAGGCUGCCAGCCGGACUUCUGCUGGGUCAUGGGCCCAUCAGGAUGGUCUUGGCGAUUCUAGCCUUUUUGAGAUUCACGGCAAUCAAGC
KU758877     AUGAAAAACCCAAAAAAGAAAUCCGGAGGAUUCCGGAUUGUCAAUAUGCUAAAACGCGGAGUAGCCCGUGUGAGCCCCUUUGGGGGCUUGAAGAGGCUGCCAGCCGGACUUCUGCUGGGUCAUGGGCCCAUCAGGAUGGUCUUGGCGAUUCUAGCCUUUUUGAGAUUCACGGCAAUCAAGC
KU866423     AUGAAAAACCCAAAAAAGAAAUCCGGAGGAUUCCGGAUUGUCAAUAUGCUAAAACGCGGAGUAGCCCGUGUGAGCCCCUUUGGGGGCUUGAAGAGGCUGCCAGCCGGACUUCUGCUGGGUCAUGGGCCCAUCAGGAUGGUCUUGGCGAUUCUAGCCUUCUUGAGAUUCACGGCAAUCAAGC
KX377336     AUGAAAAACCCAAAAAAGAAAUCCGGAGGAUUCCGGAUUGUCAAUAUGCUAAAACGCGGAGUAGCCCGUGUGAGCCCCUUUGGGGGCUUGAAGAGGCUACCAGCUGGACUUCUGCUGGGUCAUGGACCCAUCAGGAUGGUCUUGGCGAUACUAGCCUUCUUGAGAUUCACGGCAAUCAAGC
KX377337     AUGAAAAACCCAAAAAAGAAAUCCGGAGGAUUCCGGAUUGUCAAUAUGCUAAAACGCGGAGUAGCCCGUGUGAGCCCCUUUGGGGGCUUGAAGAGGCUGCCAGCCGGACUUCUGCUGGGUCAUGGGCCCAUCAGGAUGGUCUUGGCGAUUCUAGCCUUUUUGAGAUUCACGGCAAUCAAGC
KX369547     AUGAAAAACCCAAAAAAGAAAUCCGGAGGAUUCCGGAUUGUCAAUAUGCUAAAACGCGGAGUAGCCCGUGUGAGCCCCUUUGGGGGCUUGAAGAGGCUGCCAGCCGGACUUCUGCUGGGUCAUGGGCCCAUCAGGAUGGUCUUGGCGAUUCUAGCCUUUUUGAGAUUCACGGCAAUCAAGC
KX548902     AUGAAAAACCCAAAAAAGAAAUCCGGAGGAUUCCGGAUUGUCAAUAUGCUAAAACGCGGAGUAGCCCGUGUGAGCCCCUUUGGGGGCUUGAAGAGGCUGCCAGCCGGACUUCUGCUGGGUCAUGGGCCCAUCAGGAUGGUCUUGGCGAUUCUAGCCUUUUUGAGAUUCACGGCAAUCAAGC
KX601166     AUGAAAAACCCAAAGAAGAAAUCCGGAGGAUUCCGGAUUGUCAAUAUGCUAAAACGCGGAGUAGCCCGUGUAAACCCCUUGGGGGGUUUGAAGAGGCUGCCAGCCGGACUUCUGCUGGGCCAUGGACCCAUCAGAAUGGUUUUGGCGAUACUAGCCUUCUUGAGAUUCACAGCAAUCAAAC
KX601167     AUGAAAAACCCAAAAAAGAAAUCCGGAGGAUUCCGGAUUGUCAAUAUGCUAAAACGCGGAGUAGCCCGUGUGAGCCCCUUUGGGGGCUUGAAGAGGCUACCAGCUGGACUUCUGCUGGGUCAUGGACCCAUCAGGAUGGUCUUGGCGAUACUAGCCUUCUUGAGAUUCACGGCAAUCAAGC
KX601168     AUGAAAAACCCAAAAAAGAAAUCCGGAGGAUUCCGGAUUGUCAAUAUGCUAAAACGCGGAGUAGCCCGUGUGAGCCCCUUUGGGGGCUUGAAGAGGCUGCCAGCCGGACUUCUGCUGGGUCAUGGGCCCAUCAGGAUGGUCUUGGCGAUUCUAGCCUUUUUGAGAUUCACGGCAAUCAAGC
KX766028     AUGAAAAACCCAAAAAAGAAAUCCGGAGGAUUCCGGAUUGUCAAUAUGCUAAAACGCGGAGUAGCCCGUGUGAGCCCCUUUGGGGGCUUGAAGAGGCUGCCAGCCGGACUUCUGCUGGGUCAUGGGCCCAUCAGGAUGGUCUUGGCGAUUCUAGCCUUUUUGAGAUUCACGGCAAUCAAGC
KX766029     AUGAAAAACCCAAAAAAGAAAUCCGGAGGAUUCCGGAUUGUCAAUAUGCUAAAACGCGGAGUAGCCCGUGUGAGCCCCUUUGGGGGCUUGAAGAGGCUGCCAGCCGGACUUCUGCUGGGUCAUGGGCCCAUCAGGAUGGUCUUGGCGAUUCUAGCCUUUUUGAGAUUCACGGCAAUCAAGC
KU940224     ------------------------------------------------CUAAAACGCGGAGUAGCCCGUGUGAGCCCCUUUGGGGGCUUGAAGAGGCUGCCAGCCGGACUUCUGCUGGGUCAUGGGCCCAUCAGGAUGGUCUUGGCGAUUCUAGCCUUUUUGAGAUUCACGGCAAUCAAGC
```

```
             111111111111111111222222222222222222222222222222222222222222222222222222222222222222222222222222222222222222222222222223333333333333333333333333333333333333333333333333333333333333333
             888888889999999999000000000011111111112222222222333333333344444444445555555555666666666677777777778888888888999999999900000000001111111111222222222233333333334444444444555555555566 6
Sequence     2345678901234567890123456789012345678901234567890123456789012345678901234567890123456789012345678901234567890123456789012345678901234567890123456789012345678901234567890123456789012
-------------------------------------------------------------------------------------------------------------------------------------------------------------------------------------------------------

NC_012532    CAUCACUGGGCCUUAUCAACAGAUGGGGUUCCGUGGGGAAAAAAGAGGCUAUGGAAAUAAUAAAGAAGUUCAAGAAAGAUCUUGCUGCCAUGUUGAGAAUAAUCAAUGCUAGGAAAGAGAGGAAGAGACGUGGCGCAGACACCAGCAUCGGAAUCAUUGGCCUCCUGCUGACUACAGCCAU
KU922960     CAUCACUGGGUCUCAUCAAUAGAUGGGGUUCAGUGGGGAAAAAAGAGGCUAUGGAAAUAAUAAAGAAGUUCAAGAAAGAUCUGGCUGCCAUGCUGAGAAUAAUCAAUGCUAGGAAGGAGAAGAAGAGACGAGGCGCAGAAACUAGUGUCGGAAUUGUUGGCCUCCUGCUGACCACAGCUAU
KU922923     CAUCACUGGGUCUCAUCAAUAGAUGGGGUUCAGUGGGGAAAAAAGAGGCUAUGGAAAUAAUAAAGAAGUUCAAGAAAGAUCUGGCUGCCAUGCUGAGAAUAAUCAAUGCUAGGAAGGAGAAGAAGAGACGAGGCGCAGAAACUAGUGUCGGAAUUGUUGGCCUCCUGCUGACCACAGCUAU
KU820898     CAUCACUGGGUCUCAUCAAUAGAUGGGGUUCAGUGGGGAAAAAAGAGGCUAUGGAAAUAAUAAAGAAGUUCAAGAAAGAUCUGGCUGCCAUGCUGAGAAUAAUCAAUGCUAGGAAGGAGAAGAAGAGACGAGGCGCAGAAACUAGUGUCGGAAUUGUUGGCCUCCUGCUGACCACAGCUAU
KU321639     CAUCACUGGGUCUCAUCAAUAGAUGGGGUUCAGUGGGGAAAAAAGAGGCUAUGGAAAUAAUAAAGAAGUUCAAGAAAGAUCUGGCUGCCAUGCUGAGAAUAAUCAAUGCUAGGAAGGAGAAGAAGAGACGGGGCGCAGAUACUAGUGUCGGAAUUGUUGGCCUCCUGCUGACCACAGCUAU
KU820899     CAUCACUGGGUCUCAUCAAUAGAUGGGGUUCAGUGGGGAAAAAAGAGGCUAUGGAAAUAAUAAAGAAGUUCAAGAAAGAUCUGGCUGCCAUGCUGAGAAUAAUCAAUGCUAGGAAGGAGAAGAAGAGACGAGGCGCAGAUACUAAUGUCGGAAUUGUUGGCCUCCUGCUGACCACAGCUAU
KU853012     CAUCACUGGGUCUCAUCAAUAGAUGGGGUUCAGUGGGGAAAAAAGAGGCUAUGGAAAUAAUAAAGAAGUUCAAGAAAGAUCUGGCUGCCAUGCUGAGAAUAAUCAAUGCUAGGAAGGAGAAGAAGAGACGAGGCGCAGAUACUAGUGUCGGAAUUGUUGGCCUCCUGCUGACCACAGCUAU
KU729217     CAUCACUGGGUCUCAUCAAUAGAUGGGGUUCAGUGGGGAAAAAAGAGGCUAUGGAAAUAAUAAAGAAGUUCAAGAAAGAUCUGGCUGCCAUGCUGAGAAUAAUCAAUGCUAGGAAGGAGAAGAAGAGACGAGGCGCAGAUACUAGUGUCGGAAUUGUUGGCCUCCUGCUGACCACAGCUAU
KU729218     CAUCACUGGGUCUCAUCAAUAGAUGGGGUUCAGUGGGGAAAAAAGAGGCUAUGGAAAUAAUAAAGAAGUUCAAGAAAGAUCUGGCUGCCAUGCUGAGAAUAAUCAAUGCUAGGAAGGAGAAGAAGAGACGAAGCGCAGAUACUAGUGUCGGAAUUGUUGGCCUCCUGCUGACCACAGCUAU
```

```
KU761564    CAUCACUGGGUCUCAUCAAUAGAUGGGGUUCAGUGGGGAAAAAAGAGGCUAUGGAAAUAAUAAAGAAGUUCAAGAAAGAUCUGGCUGCCAUGCUGAGAAUAAUCAAUGCUAGGAAGGAGAAGAAGAGACGAGGCGCAGAUACUAGUGUCGGAAUUGUUGGCCUCCUGCUGACCACAGCUAU
KU740184    CAUCACUGGGUCUCAUCAAUAGAUGGGGUUCAGUGGGGAAAAAAGAGGCUAUGGAAAUAAUAAAGAAGUUCAAGAAAGAUCUGGCUGCCAUGCUGAGAAUAAUCAAUGCUAGGAAGGAGAAGAAGAGACGAGGCGCAGAUACUAGUGUCGGAAUUGUUGGCCUCCUGCUGACCACAGCUAU
KU744693    CAUCACUGGGUCUCAUCAAUAGAUGGGGUUCAGUGGGGAAAAAAGAUGCUAUGGAAAUAAUAAAGAAGUUCAAGAAAGAUCUGGCUGCCAUGCUGAGAAUAAUCAAUGCUAGGAAGGAGAAGAAGAGACGAGGCGCAGAUACUAGUGUCGGAAUUGUUGGCCUCCUGCUGACCACAGCUAU
KU497555    CAUCACUGGGUCUCAUCAAUAGAUGGGGUUCAGUGGGGAAAAAAGAGGCUAUGGAAAUAAUAAAGAAGUUCAAGAAAGAUCUGGCUGCCAUGCUGAGAAUAAUCAAUGCCAGGAAGGAGAAGAAGAGACGAGGCGCAGAUACUAGUGUCGGAAUCGUUGGCCUCCUGCUGACCACAGCUAU
KU527068    CAUCACUGGGUCUCAUCAAUAGAUGGGGUUCAGUGGGGAAAAAAGAGGCUAUGGAAAUAAUAAAGAAGUUCAAGAAAGAUCUGGCUGCCAUGCUGAGAAUAAUCAAUGCUAGGAAGGAGAAGAAGAGACGAGGCGCAGAUACUAGUGUCGGAAUUGUUGGCCUCCUGCUGACCACAGCUAU
KU501217    CAUCACUGGGUCUCAUCAAUAGAUGGGGUUCAGUGGGGAAAAAAGAGGCUAUGGAAAUAAUAAAGAAGUUCAAGAAAGAUCUGGCUGCCAUGCUGAGAAUAAUCAAUGCUAGGAAGGAGAAGAAGAGACGAGGCGCAGAUACUAGUGUCGGAAUUGUUGGCCUCCUGCUGACCACAGCUAU
KU501216    CAUCACUGGGUCUCAUCAAUAGAUGGGGUUCAGUGGGGAAAAAAGAGGCUAUGGAAAUAAUAAAGAAGUUCAAGAAAGAUCUGGCUGCCAUGCUGAGAAUAAUCAAUGCUAGGAAGGAGAAGAAGAGACGAGGCGCAGAUACUAGUGUCGGAAUUGUUGGCCUCCUGCUGACCACAGCUAU
KU501215    CAUCACUGGGUCUCAUCAAUAGAUGGGGUUCAGUGGGGAAAAAAGAGGCUAUGGAAACAAUAAAGAAGUUCAAGAAAGAUCUGGCUGCCAUGCUGAGAAUAAUCAAUGCUAGGAAGGAGAAGAAGAGACGAGGCGCAGAUACUAGUGUCGGAAUUGUUGGCCUCCUGCUGACCACAGCUAU
KU365780    CAUCACUGGGUCUCAUCAAUAGAUGGGGUUCAGUGGGGAAAAAAGAGGCUAUGGAAAUAAUAAAGAAGUUCAAGAAAGAUCUGGCUGCCAUGCUGAGAAUAAUCAAUGCUAGGAAGGAGAAGAAGAGACGAGGCGCAGAUACUAGUGUCGGAAUUGUUGGCCUCCUGCUGACCACAGCUAU
KU365779    CAUCACUGGGUCUCAUCAAUAGAUGGGGUUCAGUGGGGAAAAAAGAGGCUAUGGAAAUAAUAAAGAAGUUCAAGAAAGAUCUGGCUGCCAUGCUGAGAAUAAUCAAUGCUAGGAAGGAGAAGAAGAGACGAGGCGCAGAUACUAGUGUCGGAAUUGUUGGCCUCCUGCUGACCACAGCUAU
KU707826    CAUCACUGGGUCUCAUCAAUAGAUGGGGUUCAGUGGGGAAAAAAGAGGCUAUGGAAAUAAUAAAGAAGUUCAAGAAAGAUCUGGCUGCCAUGCUGAGAAUAAUCAAUGCUAGGAAGGAGAAGAAGAGACGAGGCGCAGAUACUAGUGUCGGAAUUGUUGGCCUCCUGCUGACCACAGCUAU
KU365778    CAUCACUGGGUCUCAUCAAUAGAUGGGGUUCAGUUGGGAAAAAAGAGGCUAUGGAAAUAAUAAAGAAGUUCAAGAAAGAUCUGGCUGCCAUGCUGAGAAUAAUCAAUGCUAGGAAGGAGAAGAAGAGACGAGGCGCAGAUACUAGUGUCGGAAUUGUUGGCCUCCUGCUGACCACAGCUAU
KU365777    CAUCACUGGGUCUCAUCAAUAGAUGGGGUUCAGUGGGGAAAAAAGAGGCUAUGGAAAUAAUAAAGAAGUUCAAGAAAGAUCUGGCUGCCAUGCUGAGAAUAAUCAAUGCUAGGAAGGAGAAGAAGAGACGAGGCGCAGAUACUAGUGUCGGAAUUGUUGGCCUCCUGCUGACCACAGCUAU
KU312312    CAUCACUGGGUCUCAUCAAUAGAUGGGGUUCAGUGGGGAAAAAAGAGGCUAUGGAAAUAAUAAAGAAGUUCAAGAAAGAUCUGGCUGCCAUGCUGAGAAUAAUCAAUGCUAGGAAGGAGAAGAAGAGACGAGGCGCAGAUACUAGUGUCGGAAUUGUUGGCCUCCUGCUGACCACAGCUAU
KF268950    CAUCACUGGGCCUCAUCAACAGAUGGGGUUCUGUGGGGAAAAAAGAGGCUAUGGAAAUAAUAAAGAAGUUUAAGAAAGAUCUUGCUGCCAUGUUGAGAAUAAUCAAUGCUAGGAAGGAGAGGAAGAGACGUGGUGCAGACACCAGCAUCGGAAUCAUUGGUCUCCUGCUGACUACAGCCAU
KF268949    CAUCACUGGGCCUCAUCAACAGAUGGGGUUCAGUGGGGAAAAAAGAGGCUAUGGAAAUAAUAAAGAAGUUUAAGAAAGAUCUUGCUGCCAUGUUGAGAAUAAUCAAUGCUAGGAAGGAGAGGAAGAGACGUGGUGCAGACACCAGCAUCGGAAUCAUUGGUCUCCUGCUGACUACAGCCAU
KF268948    CAUCACUGGGCCUCAUCAACAGAUGGGGUUCUGUGGGGAAAAAAGAGGCUAUGGAAAUAAUAAAGAAGUUUAAGAAAGAUCUUGCUGCCAUGUUGAGAAUAAUCAAUGCUAGGAAGGAGAGGAAGAGACGUGGUGCAGACACCAGCAUCGGAAUCAUUGGUCUCCUGCUGACUACAGCCAU
KJ776791    CAUCACUGGGUCUCAUCAAUAGAUGGGGUUCAGUGGGGAAAAAAGAGGCUAUGGAAAUAAUAAAGAAGUUCAAGAAAGAUCUGGCUGCCAUGCUGAGAAUAAUCAAUGCUAGGAAGGAGAAGAAGAGACGAGGCGCAGAUACUAGUGUCGGAAUUGUUGGCCUCCUGCUGACCACAGCUAU
KF383119    CAUCACUGGGCCUUAUCAACAGAUGGGGUUCCGUGGGGAAAAAAGAGGCUAUGGAAAUAAUAAAGAAGUUCAAGAAAGAUCUUGCUGCCAUGUUGAGAAUAAUCAAUGCUAGGAAAGAGAGGAAGAGACGUGGCGCAGACACCAGCAUCGGAAUCAUUGGCCUCCUGCUGACUACAGCCAU
KF383118    CAUCACUGGGCCUUAUCAACAGAUGGGGUUCCGUGGGGAAAAAAGAGGCUAUGGAAAUAAUAAAGAAGUUCAAGAAAGAUCUUGCUGCCAUGUUGAGAAUAAUCAAUGCUAGGAAAGAGAGGAAGAGACGUGGCGCAGACACCAGCAUCGGAAUCAUUGGCCUCCUGCUGACUACAGCCAU
KF383117    CAUCACUGGGCCUCAUCAAUAGAUGGGGUUCAGUGGGGAAAAAAGAGGCUAUGGAGAUAAUAAAGAAGUUUAAGAAAGAUCUUGCUGCCAUGUUGAGAAUAAUCAAUGCUAGGAAGGAGAGGAAGAGACGUGGUGCAGACACCAGUAUCGGAAUCAUUGGCCUCCUGCUGACUACAGCCAU
KF383116    CAUCACUGGGCCUCAUCAAUAGAUGGGGUUCCGUGGGGAAGAAGGAGGCUAUGGAAAUAAUAAAAAAGUUCAAGAAAGACCUUGCUGCCAUGUUGAGAAUUAUCAAUGCUAGGAAGGAGAGGAAGAGACGUGGCGCUGACACCAGCAUCGGAAUCGUCGGCCUCCUGCUGACCACAGCCAU
KF383115    CAUCACUGGGCCUCAUCAAUAGAUGGGGUUCAGUGGGGAAAAAAGAGGCUAUGGAGAUAAUAAAGAAGUUUAAGAAAGAUCUUGCUGCCAUGUUGAGAAUAAUCAAUGCUAGGAAGGAGAGGAAGAGACGUGGUGCAGACACCAGUAUCGGAAUCAUUGGCCUCCUGCUGACUACAGCCAU
EU545988    CAUCACUGGGUCUCAUCAAUAGAUGGGGUUCAGUGGGGAAAAAAGAGGCUAUGGAAAUAAUAAAGAAGUUCAAGAAAGAUCUGGCUGCCAUGCUGAGAAUAAUCAAUGCUAGGAAGGAGAAGAAGAGACGAGGCACAGAUACUAGUGUCGGAAUUGUUGGCCUCCUGCUGACCACAGCCAU
KU955589    CAUCACUGGGUCUCAUCAAUAGAUGGGGUUCAGUGGGGAAAAAAGAGGCUAUGGAAAUAAUAAAGAAGUUCAAGAAAGAUCUGGCUGCCAUGCUGAGAAUAAUCAAUGCUAGGAAGGAGAAGAAGAGACGAGGCGCAGAUACUAAUGUCGGAAUUGUUGGCCUCCUGCUGACCACAGCUAU
KU955590    CAUCACUGGGUCUCAUCAAUAGAUGGGGUUCAGUGGGGAAAAAAGAGGCUAUGGAAAUAAUAAAGAAGUUCAAGAAAGAUCUGGCUGCCAUGCUGAGAAUAAUCAAUGCUAGGAAGGAGAAGAAGAGACGAGGCGCAGAUACUAGUGUCGGAAUUGUUGGCCUCCUGCUGACCACAGCUAU
KU940228    CAUCACUGGGUCUCAUCAAUAGAUGGGGUUCAGUGGGGAAAAAAGAGGCUAUGGAAAUAAUAAAGAAGUUCAAGAAAGAUCUGGCUGCCAUGCUGAGAAUAAUCAAUGCUAGGAAGGAGAAGAAGAGACGAGGCGCAGAUACUAGUGUCGGAAUUGUUGGCCUCCUGCUGACCACAGCUAU
KU991811    CAUCACUGGGUCUCAUCAAUAGAUGGGGUUCAGUGGGGAAAAAAGAGGCUAUGGAAAUAAUAAAGAAGUUCAAGAAAGAUCUGGCUGCCAUGCUGAGAAUAAUCAAUGCUAGGAAGGAGAAGAAGAGACGAGGCGCAGAUACUAGUGUCGGAAUUGUUGGCCUCCUGCUGACCACAGCUAU
KU509998    CAUCACUGGGUCUCAUCAAUAGAUGGGGUUCAGUGGGGAAAAAAGAGGCUAUGGAAAUAAUAAAGAAGUUCAAGAAAGAUCUGGCUGCCAUGCUGAGAAUAAUCAAUGCUAGGAAGGAGAAGAAGAGACGAGGCGCAGAUACUAGUGUCGGAAUUGUUGGCCUCCUGCUGACCACAGCUAU
KX056898    CAUCACUGGGUCUCAUCAAUAGAUGGGGUUCAGUGGGGAAAAAAGAGGCUAUGGAAAUAAUAAAGAAGUUCAAGAAAGAUCUGGCUGCCAUGCUGAGAAUAAUCAAUGCUAGGAAGGAGAAGAAGAGACGAGGCGCAGAUACUAGUGUCGGAAUUGUUGGCCUCCUGCUGACCACAGCUAU
KX117076    CAUCACUGGGUCUCAUCAAUAGAUGGGGUUCAGUGGGGAAAAAAGAGGCUAUGGAAAUAAUAAAGAAGUUCAAGAAAGAUCUGGCUGCCAUGCUGAGAAUAAUCAAUGCUAGGAAGGAGAAGAAGAGACGAGGCGCAGAUACUAAUGUCGGAAUUGUUGGCCUCCUGCUGACCACAGCUAU
KX185891    CAUCACUGGGUCUCAUCAAUAGAUGGGGUUCAGUGGGGAAAAAAGAGGCUAUGGAAAUAAUAAAGAAGUUCAAGAAAGAUCUGGCUGCCAUGCUGAGAAUAAUCAAUGCUAGGAAGGAGAAGAAGAGACGAGGCGCAGAUACUAAUGUCGGAAUUGUUGGCCUCCUGCUGACCACAGCUAU
KX247646    CAUCACUGGGUCUCAUCAAUAGAUGGGGUUCAGUGGGGAAAAAAGAGGCUAUGGAAAUAAUAAAGAAGUUCAAGAAAGAUCUGGCUGCCAUGCUGAGAAUAAUCAAUGCUAGGAAGGAGAAGAAGAGACGAGGCGCAGAAACUAGUGUCGGAAUUGUUGGCCUCCUGCUGACCACAGCUAU
KX262887    CAUCACUGGGUCUCAUCAAUAGAUGGGGUUCAGUGGGGAAAAAAGAGGCUAUGGAAAUAAUAAAGAAGUUCAAGAAAGAUCUGGCUGCCAUGCUGAGAAUAAUCAAUGCUAGGAAGGAGAAGAAGAGACGAGGCGCAGAUACUAGUGUCGGAAUUGUUGGCCUCCUGCUGACCACAGCUAU
KX280026    CAUCACUGGGUCUCAUCAAUAGAUGGGGUUCAGUGGGGAAAAAAGAGGCUAUGGAAAUAAUAAAGAAGUUCAAGAAAGAUCUGGCUGCCAUGCUGAGAAUAAUCAAUGCUAGGAAGGAGAAGAAGAGACGAGGCGCAGAUACUAGUGUCGGAAUUGUUGGCCUCCUGCUGACCACAGCUAU
KU758877    CAUCACUGGGUCUCAUCAAUAGAUGGGGUUCAGUGGGGAAAAAAGAGGCUAUGGAAAUAAUAAAGAAGUUCAAGAAAGAUCUGGCUGCCAUGCUGAGAAUAAUCAAUGCUAGGAAGGAGAAGAAGAGACGAGGCGCAGAUACUAGUGUCGGAAUUGUUGGCCUCCUGCUGACCACAGCUAU
KU866423    CAUCACUGGGUCUCAUCAAUAGAUGGGGUUCAGUGGGGAAAAAAGAGGCUAUGGAAAUAAUAAAGAAGUUCAAGAAAGAUCUGGCUGCCAUGCUGAGAAUAAUCAAUGCUAGGAAGGAGAAGAAGAGACGAGGCGCAGAUACUAAUGUCGGAAUUGUUGGCCUCCUGCUGACCACAGCUAU
KX377336    CAUCACUGGGUCUCAUCAAUAGAUGGGGUUCCGUGGGGAAAAAAGAGGCUAUGGAAAUAAUAAAGAAGUUCAAGAAAGAUCUGGCUGCCAUGCUGAGAAUAAUCAAUGCUAGGAAGGAGAAGAAGAGACGUGGCGCAGACACCAGUGUCGGAAUUGUUGGCCUCCUGCUGACCACAGCCAU
KX377337    CAUCACUGGGUCUCAUCAAUAGAUGGGGUUCAGUGGGGAAAAAAGAGGCUAUGGAAACAAUAAAGAAGUUCAAGAAAGAUCUGGCUGCCAUGCUGAGAAUAAUCAAUGCUAGGAAGGAGAAGAAGAGACGAGGCGCAGAUACUAGUGUCGGAAUUGUUGGCCUCCUGCUGACCACAGCUAU
KX369547    CAUCACUGGGUCUCAUCAAUAGAUGGGGUUCAGUGGGGAAAAAAGAGGCUAUGGAAAUAAUAAAGAAGUUCAAGAAAGAUCUGGCUGCCAUGCUGAGAAUAAUCAAUGCUAGGAAGGAGAAGAAGAGACGAGGCGCAGAUACUAGUGUCGGAAUUGUUGGCCUCCUGCUGACCACAGCUAU
KX548902    CAUCACUGGGUCUCAUCAAUAGAUGGGGUUCAGUGGGGAAAAAAGAGGCUAUGGAAAUAAUAAAGAAGUUCAAGAAAGAUCUGGCUGCCAUGCUGAGAAUAAUCAAUGCUAGGAAGGAGAAGAAGAGACGAGGCGCAGAAACUAGUGUCGGAAUUGUUGGCCUCCUGCUGACCACAGCUAU
KX601166    CAUCACUGGGCCUCAUCAAUAGAUGGGGUUCCGUGGGGAAGAAGGAGGCUAUGGAAAUAAUAAAAAAGUUCAAGAAAGACCUUGCUGCCAUGUUGAGAAUUAUCAAUGCUAGGAAGGAGAGGAAGAGACGUGGCGCUGACACCAGCAUCGGAAUCGUCGGCCUCCUGCUGACCACAGCCAU
KX601167    CAUCACUGGGUCUCAUCAAUAGAUGGGGUUCCGUGGGGAAAAAAGAGGCUAUGGAAAUAAUAAAGAAGUUCAAGAAAGAUCUGGCUGCCAUGCUGAGAAUAAUCAAUGCUAGGAAGGAGAAGAAGAGACGUGGCGCAGACACCAGUGUCGGAAUUGUUGGCCUCCUGCUGACCACAGCCAU
KX601168    CAUCACUGGGUCUCAUCAAUAGAUGGGGUUCAGUGGGGAAAAAAGAGGCUAUGGAAACAAUAAAGAAGUUCAAGAAAGAUCUGGCUGCCAUGCUGAGAAUAAUCAAUGCUAGGAAGGAGAAGAAGAGACGAGGCGCAGAUACUAGUGUCGGAAUUGUUGGCCUCCUGCUGACCACAGCUAU
KX766028    CAUCACUGGGUCUCAUCAAUAGAUGGGGUUCAGUGGGGAAAAAAGAGGCUAUGGAAAUAAUAAAGAAGUUCAAGAAAGAUCUGGCUGCCAUGCUGAGAAUAAUCAAUGCUAGGAAGGAGAAGAAGAGACGAGGCACAGAUACUAGUGUCGGAAUUGUUGGCCUCCUGCUGACCACAGCUAU
KX766029    CAUCACUGGGUCUCAUCAAUAGAUGGGGUUCAGUGGGGAAAAAAGAGGCUAUGGAAAUAAUAAAGAAGUUCAAGAAAGAUCUGGCUGCCAUGCUGAGAAUAAUCAAUGCUAGGAAGGAGAAGAAGAGACGAGGCGCAGAUACUAGUGUCGGAAUUGUUGGCCUCCUGCUGACCACAGCUAU
KU940224    CAUCACUGGGUCUCAUCAAUAGAUGGGGUUCAGUGGGGAAAAAAGAGGCUAUGGAAAUAAUAAAGAAGUUCAAGAAAGAUCUGGCUGCCAUGCUGAGAAUAAUCAAUGCUAGGAAGGAGAAGAAGAGACGAGGCGCAGAUACUAGUGUCGGAAUUGUUGGCCUCCUGCUGACCACAGCUAU


            3333333333333333333333333333333333333444444444444444444444444444444444444444444444444444444444444444444444444444444444444444444444455555555555555555555555555555555555555555555
            6666666777777777788888888889999999999000000000011111111112222222222333333333344444444445555555555666666666677777777778888888888999999999900000000001111111111222222222233333333334444
Sequence    3456789012345678901234567890123456789012345678901234567890123456789012345678901234567890123456789012345678901234567890123456789012345678901234567890123456789012345678901234567890123
-------------------------------------------------------------------------------------------------------------------------------------------------------------------------------------------------
NC_012532   GGCAGCAGAGAUCACUAGACGCGGGAGUGCAUACUACAUGUACUUGGAUAGGAGCGAUGCCGGGAAGGCCAUUUCGUUUGCUACCACAUUGGGAGUGAACAAGUGCCACGUACAGAUCAUGGACCUCGGGCACAUGUGUGACGCCACCAUGAGUUAUGAGUGCCCUAUGCUGGAUGAGGGA
KU922960    GGCAGCGGAGGUCACUAGACGUGGGAGUGCAUACUAUAUGUACUUGGACAGAAACGAUGCUGGGGAGGCCAUAUCUUUUCCAACCACAUUGGGGAUGAAUAAGUGUUAUAUACAGAUCAUGGAUCUUGGACACAUGUGUGAUGCCACCAUGAGCUAUGAAUGCCCUAUGCUGGAUGAGGGG
KU922923    GGCAGCGGAGGUCACUAGACGUGGGAGUGCAUACUAUAUGUACUUGGACAGAAACGAUGCUGGGGAGGCCAUAUCUUUUCCAACCACAUUGGGGAUGAAUAAGUGUUAUAUACAGAUCAUGGAUCUUGGACACAUGUGUGAUGCCACCAUGAGCUAUGAAUGCCCUAUGCUGGAUGAGGGG
KU820898    GGCAGCGGAGGUCACUAGACGUGGGAGUGCAUACUAUAUGUACUUGGACAGAAACGAUGCUGGGGAGGCCAUAUCUUUUCCAACCACAUUGGGGAUGAAUAAGUGUUAUAUACAGAUCAUGGAUCUUGGACACAUGUGUGAUGCCACCAUGAGCUAUGAAUGCCCUAUGCUGGAUGAGGGG
KU321639    GGCAGCGGAGGUCACUAGACGUGGGAGUGCAUACUAUAUGUACUUGGACAGAAACGAUGCUGGGGAGGCCAUAUCUUUUCCAACCACAUUGGGGAUGAAUAAGUGUUAUAUACAGAUCAUGGAUCUUGGACACAUGUGUGAUGCCACCAUGAGCUAUGAAUGCCCUAUGCUGGAUGAGGGG
KU820899    GGCAGCGGAGGUCACUAGACGUGGGAGUGCAUACUAUAUGUACUUGGACAGAAACGAUGCUGGGGAGGCCAUAUCUUUUCCAACCACAUUGGGGAUGAAUAAGUGUUAUAUACAGAUCAUGGAUCUUGGACACAUGUGUGAUGCCACCAUGAGCUAUGAAUGCCCUAUGCUGGAUGAGGGG
KU853012    GGCAGCGGAGGUCACUAGACGUGGGAGUGCAUACUACAUGUACUUGGACAGAAACGAUGCUGGGGAGGCCAUAUCUUUUCCAACCACAUUGGGGAUGAAUAAGUGUUAUAUACAGAUCAUGGAUCUUGGACACAUGUGUGAUGCCACCAUGAGCUAUGAAUGCCCUAUGCUGGAUGAGGGG
KU729217    GGCAGCGGAGGUCACUAGACGUGGGAGUGCAUACUAUAUGUACUUGGACAGAAACGAUGCUGGGGAGGCCAUAUCUUUUCCAACCACAUUGGGGAUGAAUAAGUGUUAUAUACAGAUCAUGGAUCUUGGACACAUGUGUGAUGCCACCAUGAGCUAUGAAUGCCCUAUGCUGGAUGAGGGG
KU729218    GGCAGCGGAGGUCACUAGACGUGGGAGUGCAUACUAUAUGUACUUGGACAGAAACGAUGCUGGGGAGGCCAUAUCUUUUCCAACCACAUUGGGGAUGAAUAAGUGUUAUAUACAGAUCAUGGAUCUUGGACACAUGUGUGAUGCCACUAUGAGCUAUGAAUGCCCUAUGCUGGAUGAGGGG
KU761564    GGCAGCGGAGGUCACUAGACGUGGGAGUGCAUACUAUAUGUACUUGGACAGAAACGAUGCUGGGGAGGCCAUAUCUUUUCCAACCACAUUGGGGAUGAAUAAGUGUUAUAUACAGAUCAUGGAUCUUGGACACAUGUGUGAUGCCACCAUGAGCUAUGAAUGCCCUAUGCUGGAUGAGGGG
KU740184    GGCAGCGGAGGUCACUAGACGUGGGAGUGCAUACUAUAUGUACUUGGACAGAAACGAUGCUGGGGAGGCCAUAUCUUUUCCAACCACAUUGGGGAUGAAUAAGUGUUAUAUACAGAUCAUGGAUCUUGGACACAUGUGUGAUGCCACCAUGAGCUAUGAAUGCCCUAUGCUGGAUGAGGGG
KU744693    GGCAGCGGAGGUCACUAGACGUGGGAGUGCAUACUAUAUGUACUUGGACAGAAACGAUGCUGGGGAGGCCAUAUCUUUUCCAACCACAUUGGGGAUGAAUAAGUGUUAUAUACAGAUCAUGGAUCUUGGACACAUGUGUGAUGCCACCAUGAGCUAUGAAUGCCCUAUGCUGGAUGAGGGG
KU497555    GGCAGCGGAGGUCACUAGACGUGGGAGUGCAUACUAUAUGUACUUGGACAGAAACGAUGCUGGGGAGGCCAUAUCUUUUCCAACCACAUUGGGGAUGAAUAAGUGUUAUAUACAGAUCAUGGAUCUUGGACACAUGUGUGAUGCCACCAUGAGCUAUGAAUGCCCUAUGCUGGAUGAGGGG
KU527068    GGCAGCGGAGGUCACUAGACGUGGGAGUGCAUACUAUAUGUACUUGGACAGAAACGAUGCUGGGGAGGCCAUAUCUUUUCCAACCACAUUGGGGAUGAAUAAGUGUUAUAUACAGAUCAUGGAUCUUGGACACAUGUGUGAUGCCACCAUGAGCUAUGAAUGCCCUAUGCUGGAUGAGGGG
KU501217    GGCAGCGGAGGUCACUAGACGUGGGAGUGCAUACUAUAUGUACUUGGACAGAAACGAUGCUGGGGAGGCCAUAUCUUUUCCAACCACAUUGGGGAUGAAUAAGUGUUAUAUACAGAUCAUGGAUCUUGGACACAUGUGUGAUGCCACCAUGAGCUAUGAAUGCCCUAUGCUGGAUGAGGGG
KU501216    GGCAGCGGAGGUCACUAGACGUGGGAGUGCAUACUAUAUGUACUUGGACAGAAACGAUGCUGGGGAGGCCAUAUCUUUUCCAACCACAUUGGGGAUGAAUAAGUGUUAUAUACAGAUCAUGGAUCUUGGACACAUGUGUGAUGCCACCAUGAGCUAUGAAUGCCCUAUGCUGGAUGAGGGG
KU501215    GGCAGCGGAGGUCACUAGACGUGGGAGUGCAUACUAUAUGUACUUGGACAGAAACGAUGCUGGGGAGGCCAUAUCUUUUCCAACCACAUUGGGGAUGAAUAAGUGUUAUAUACAGAUCAUGGAUCUUGGACACAUGUGUGAUGCCACCAUGAGCUAUGAAUGCCCUAUGCUGGAUGAGGGG
KU365780    GGCAGCGGAGGUCACUAGACGUGGGAGUGCAUACUAUAUGUACUUGGACAGAAACGAUGCUGGGGAGGCCAUAUCUUUUCCAACCACAUUGGGGAUGAAUAAGUGUUAUAUACAGAUCAUGGAUCUUGGACACAUGUGUGAUGCCACCAUGAGCUAUGAAUGCCCUAUGCUGGAUGAGGGG
KU365779    GGCAGCGGAGGUCACUAGACGUGGGAGUGCAUACUAUAUGUACUUGGACAGAAACGAUGCUGGGGAGGCCAUAUCUUUUCCAACCACAUUGGGGAUGAAUAAGUGUUAUAUACAGAUCAUGGAUCUUGGACACAUGUGUGAUGCCACCAUGAGCUAUGAAUGCCCUAUGCUGGAUGAGGGG
KU707826    GGCAGCGGAGGUCACUAGACGUGGGAGUGCAUACUAUAUGUACUUGGACAGAAACGAUGCUGGGGAGGCCAUAUCUUUUCCAACCACAUUGGGGAUGAAUAAGUGUUAUAUACAGAUCAUGGAUCUUGGACACAUGUGUGAUGCCACCAUGAGCUAUGAAUGCCCUAUGCUGGAUGAGGGG
KU365778    GGCAGCGGAGGUCACUAGACGUGGGAGUGCAUACUAUAUGUACUUGGACAGAAACGAUGCUGGGGAGGCCAUAUCUUUUCCAACCACAUUGGGGAUGAAUAAGUGUUAUAUACAGAUCAUGGAUCUUGGACACAUGUGUGAUGCCACCAUGAGCUAUGAAUGCCCUAUGCUGGAUGAGGGG
KU365777    GGCAGCGGAGGUCACUAGACGUGGGAGUGCAUACUAUAUGUACUUGGACAGAAACGAUGCUGGGGAGGCCAUAUCUUUUCCAACCACAUUGGGGAUGAAUAAGUGUUAUAUACAGAUCAUGGAUCUUGGACACAUGUGUGAUGCCACCAUGAGCUAUGAAUGCCCUAUGCUGGAUGAGGGG
KU312312    GGCAGCGGAGGUCACUAGACGUGGGAGUGCAUACUAUAUGUACUUGGACAGAAACGAUGCUGGGGAGGCCAUAUCUUUUCCAACCACAUUGGGGAUGAAUAAGUGUUAUAUACAGAUCAUGGAUCUUGGACACACGUGUGAUGCCACCAUGAGCUAUGAAUGCCCUAUGCUGGAUGAGGGG
KF268950    GGCAGCGGAGAUUACCAGACGUGGAAGUGCAUACUACAUGUACUUGGACAGGAGCGAUGCUGGGAAGGCCAUCUCCUUUGCUACCAACUUGGGAGUUAACAAAUGCCAUGUACAGAUCAUGGAUCUCGGGCACAUGUGUGACGCCACCAUGAGUUAUGAGUGCCCUAUGUUGGACGAGGGA
KF268949    GGCAGCAGAGAUUACCAGACGUGGAAGUGCAUACUACAUGUACUUGGACAGGAGCGAUGCUGGGAAGGCCAUCUCCUUUGCUACCAAUUUGGGAGUUAACAAAUGCCAUGUACAGAUCAUGGAUCUCGGGCACAUGUGUGACGCCACCAUGAGUUAUGAGUGUCCUAUGUUGGACGAGGGA
```

```
KF268948    GGCAGCGGAGAUUACCAGACGUGGAAGUGCAUACUACAUGUACUUGGACAGGAGCGAUGCUGGGAAGGCCAUCUCCUUUGCUACCAACUUGGGAGUUAACAAAUGCCAUGUACAGAUCAUGGAUCUCGGGCACAUGUGUGACGCCACCAUGAGUUAUGAGUGCCCUAUGUUGGACGAGGGA
KJ776791    GGCAGCGGAGGUCACUAGACGUGGGAGUGCAUACUAUAUGUACUUGGACAGAAACGACGCUGGGGAGGCCAUAUCUUUUCCAACCACAUUGGGGAUGAAUAAGUGUUAUAUACAGAUCAUGGAUCUUGGACACAUGUGUGAUGCCACCAUGAGCUAUGAAUGCCCUAUGCUGGAUGAGGGG
KF383119    GGCAGCAGAGAUCACUAGACGCGGGAGUGCAUACUACAUGUACUUGGAUAGGAGCGAUGCCGGGAAGGCCAUUUCGUUUGCUACCACAUUGGGAGUGAACAAGUGCCACGUACAGAUCAUGGACCUCGGGCACAUGUGUGACGCCACCAUGAGUUAUGAGUGCCCUAUGCUGGAUGAGGGA
KF383118    GGCAGCAGAGAUCACUAGACGCGGGAGUGCAUACUACAUGUACUUGGAUAGGAGCGAUGCCGGGAAGGCCAUUUCGUUUGCUACCACAUUGGGAGUGAACAAGUGCCACGUACAGAUCAUGGACCUCGGGCACAUGUGUGACGCCACCAUGAGUUAUGAGUGCCCUAUGCUGGAUGAGGGA
KF383117    GGCAGCAGAGAUUACUAGACGAGGAAGUGCAUACUACAUGUACUUGGACAGGAGCGAUGCUGGGAAGGCCAUUUCCUUUGCUACCACAUUGGGAGUGAACAAAUGCCAUGUACAGAUCAUGGAUCUCGGGCACAUGUGUGACGCCACCAUGAGUUAUGAGUGCCCUAUGCUAGACGAAGGA
KF383116    GGCAGCCGAGAUCACCAGACGUGGGAGUGCAUACUACAUGUACUUGGACAGGAGCGAUGCUGGUAAGGCCAUUUCUUUUGCUACCACAUUGGGGGUGAACAAAUGCCAUGUACAGAUCAUGGACCUCGGGCACAUGUGUGACGCCACCAUGAGUUAUGAGUGCCCCAUGCUGGACGAGGGA
KF383115    GGCAGCAGAGAUUACUAGACGAGGAAGUGCAUACUACAUGUACUUGGACAGGAGCGAUGCUGGGAAGGCCAUUUCCUUUGCUACCACAUUGGGAGUGAACAAAUGCCAUGUACAGAUCAUGGAUCUCGGGCACAUGUGUGACGCCACCAUGAGUUAUGAGUGCCCUAUGCUAGACGAAGGA
EU545988    GGCAGUGGAGGUCACUAGACGUGGGAGUGCAUACUAUAUGUACUUGGACAGAAGCGAUGCUGGGGAGGCCAUAUCUUUUCCAACCACACUGGGGAUGAACAAGUGUUACAUACAGAUCAUGGAUCUUGGACACAUGUGUGAUGCCACCAUGAGCUAUGAAUGCCCUAUGUUGGAUGAGGGG
KU955589    GGCAGCGGAGGUCACUAGACGUGGGAGUGCAUACUAUAUGUACUUGGACAGAAACGAUGCUGGGGAGGCCAUAUCUUUUCCAACCACAUUGGGGAUGAAUAAGUGUUAUAUACAGAUCAUGGAUCUUGGACACAUGUGUGAUGCCACCAUGAGCUAUGAAUGCCCUAUGCUGGAUGAGGGG
KU955590    GGCAGCGGAGGUCACUAGACGUGGGAGUGCAUACUAUAUGUACUUGGACAGAAACGAUGCUGGGGAGGCCAUAUCUUUUCCAACCACAUUGGGGAUGAAUAAGUGUUAUAUACAGAUCAUGGAUCUUGGACACAUGUGUGAUGCCACCAUGAGCUAUGAAUGCCCUAUGCUGGAUGAGGGG
KU940228    GGCAGCGGAGGUCACUAGACGUGGGAGUGCAUACUAUAUGUACUUGGACAGAAACGAUGCUGGGGAGGCCAUAUCUUUUCCAACCACAUUGGGGAUGAAUAAGUGUUAUAUACAGAUCAUGGAUCUUGGACACAUGUGUGAUGCCACCAUGAGCUAUGAAUGCCCUAUGCUGGAUGAGGGG
KU991811    GGCAGCGGAGGUCACUAGACGUGGGAGUGCAUACUAUAUGUACUUGGACAGAAACGAUGCUGGGGAGGCCAUAUCUUUUCCAACCACAUUGGGGAUGAAUAAGUGUUAUAUACAGAUCAUGGAUCUUGGACACAUGUGUGAUGCCACCAUGAGCUAUGAAUGCCCUAUGCUGGAUGAGGGG
KU509998    GGCAGCGGAGGUCACUAGACGUGGGAGUGCAUACUAUAUGUACUUGGACAGAAACGAUGCUGGGGAGGCCAUAUCUUUUCCAACCACAUUGGGGAUGAAUAAGUGUUAUAUACAGAUCAUGGAUCUUGGACACAUGUGUGAUGCCACCAUGAGCUAUGAAUGCCCUAUGCUGGAUGAGGGG
KX056898    GGCAGCGGAGGUCACUAGACGUGGGAGUGCAUACUAUAUGUACUUGGACAGAAACGAUGCUGGGGAGGCCAUAUCUUUUCCAACCACAUUGGGGAUGAAUAAGUGUUAUAUACAGAUCAUGGAUCUUGGACACAUGUGUGAUGCCACCAUGAGCUAUGAAUGCCCUAUGCUGGAUGAGGGG
KX117076    GGCAGCGGAGGUCACUAGACGUGGGAGUGCAUACUAUAUGUACUUGGACAGAAACGAUGCUGGGGAGGCCAUAUCUUUUCCAACCACAUUGGGGAUGAAUAAGUGUUAUAUACAGAUCAUGGAUCUUGGACACAUGUGUGAUGCCACCAUGAGCUAUGAAUGCCCUAUGCUGGAUGAGGGG
KX185891    GGCAGCGGAGGUCACUAGACGUGGGAGUGCAUACUAUAUGUACUUGGACAGAAACGAUGCUGGGGAGGCCAUAUCUUUUCCAACCACAUUGGGGAUGAAUAAGUGUUAUAUACAGAUCAUGGAUCUUGGACACAUGUGUGAUGCCACCAUGAGCUAUGAAUGCCCUAUGCUGGAUGAGGGG
KX247646    GGCAGCGGAGGUCACUAGACGUGGGAGUGCAUACUAUAUGUACUUGGACAGAAACGAUGCUGGGGAGGCCAUAUCUUUUCCAACCACAUUGGGGAUGAAUAAGUGUUAUAUACAGAUCAUGGAUCUUGGACACAUGUGUGAUGCCACCAUGAGCUAUGAAUGCCCUAUGCUGGAUGAGGGG
KX262887    GGCAGCGGAGGUCACUAGACGUGGGAGUGCAUACUAUAUGUACUUGGACAGAAACGAUGCUGGGGAGGCCAUAUCUUUUCCAACCACAUUGGGGAUGAAUAAGUGUUAUAUACAGAUCAUGGAUCUUGGACACAUGUGUGAUGCCACCAUGAGCUAUGAAUGCCCUAUGCUGGAUGAGGGG
KX280026    GGCAGCGGAGGUCACUAGACGUGGGAGUGCAUACUAUAUGUACUUGGACAGAAACGAUGCUGGGGAGGCCAUAUCUUUUCCAACCACAUUGGGGAUGAAUAAGUGUUAUAUACAGAUCAUGGAUCUUGGACACAUGUGUGAUGCCACCAUGAGCUAUGAAUGCCCUAUGCUGGAUGAGGGG
KU758877    GGCAGCGGAGGUCACUAGACGUGGGAGUGCAUACUAUAUGUACUUGGACAGAAACGAUGCUGGGGAGGCCAUAUCUUUUCCAACCACAUUGGGGAUGAAUAAGUGUUAUAUACAGAUCAUGGAUCUUGGACACAUGUGUGAUGCCACCAUGAGCUAUGAAUGCCCUAUGCUGGAUGAGGGG
KU866423    GGCAGCGGAGGUCACUAGACGUGGGAGUGCAUACUAUAUGUACUUGGACAGAAACGAUGCUGGGGAGGCCAUAUCUUUUCCAACCACAUUGGGGAUGAAUAAGUGUUAUAUACAGAUCAUGGAUCUUGGACACAUGUGUGAUGCCACCAUGAGCUAUGAAUGCCCUAUGCUGGAUGAGGGG
KX377336    GGCAGUGGAGGUCACCAGACGUGGGAGUGCAUACUAUAUGUACUUAGACAGAAGCGAUGCUGGGAAGGCCAUAUCUUUUCCAACCACACUGGGGGUGAAUAAGUGUUACAUACAGAUCAUGGAUCUUGGACACAUGUGUGAUGCCACAAUGAGCUAUGAAUGCCCUAUGUUGGAUGAGGGG
KX377337    GGCAGCGGAGGUCACUAGACGUGGGAGUGCAUACUAUAUGUACUUGGACAGAAACGAUGCUGGGGAGGCCAUAUCUUUUCCAACCACAUUGGGGAUGAAUAAGUGUUAUAUACAGAUCAUGGAUCUUGGACACAUGUGUGAUGCCACCAUGAGCUAUGAAUGCCCUAUGCUGGAUGAGGGG
KX369547    GGCAGCGGAGGUCACUAGACGUGGGAGUGCAUACUAUAUGUACUUGGACAGAAACGAUGCUGGGGAGGCCAUAUCUUUUCCAACCACAUUGGGGAUGAAUAAGUGUUAUAUACAGAUCAUGGAUCUUGGACACAUGUGUGAUGCCACCAUGAGCUAUGAAUGCCCUAUGCUGGAUGAGGGG
KX548902    GGCAGCGGAGGUCACUAGACGUGGGAGUGCAUACUAUAUGUACUUGGACAGAAACGAUGCUGGGGAGGCCAUAUCUUUUCCAACCACAUUGGGGAUGAAUAAGUGUUAUAUACAGAUCAUGGAUCUUGGACACAUGUGUGAUGCCACCAUGAGCUAUGAAUGCCCUAUGCUGGAUGAGGGG
KX601166    GGCAGCCGAGAUCACUAGACGUGGGAGUGCAUACUACAUGUACUUGGACAGGAGCGAUGCUGGUAAGGCCAUUUCUUUUGCCACCACAUUGGGGGUGAACAAAUGCCAUGUACAGAUCAUGGACCUCGGGCACAUGUGUGACGCCACCAUGAGUUAUGAGUGCCCCAUGCUAGACGAGGGA
KX601167    GGCAGUGGAGGUCACCAGACGUGGGAGUGCAUACUAUAUGUACUUAGACAGAAGCGAUGCUGGGGAGGCCAUAUCUUUUCCAACCACACUGGGGGUGAAUAAGUGUUACAUACAGAUCAUGGAUCUUGGACACAUGUGUGAUGCCACAAUGAGCUAUGAAUGCCCUAUGUUGGAUGAGGGG
KX601168    GGCAGCGGAGGUCACUAGACGUGGGAGUGCAUACUAUAUGUACUUGGACAGAAACGAUGCUGGGGAGGCCAUAUCUUUUCCAACCACAUUGGGGAUGAAUAAGUGUUAUAUACAGAUCAUGGAUCUUGGACACAUGUGUGAUGCCACCAUGAGCUAUGAAUGCCCUAUGCUGGAUGAGGGG
KX766028    GGCAGCGGAGGUCACUAGACGUGGGAGUGCAUACUAUAUGUACUUGGACAGAAACGAUGCUGGGGAGGCCAUAUCUUUUCCAACCACAUUGGGGAUGAAUAAGUGUUAUAUACAGAUCAUGGAUCUUGGACACAUGUGUGAUGCCACCAUGAGCUAUGAAUGCCCUAUGCUGGAUGAGGGG
KX766029    GGCAGCGGAGGUCACUAGACGUGGGAGUGCAUACUAUAUGUACUUGGACAGAAACGAUGCUGGGGAGGCCAUAUCUUUUCCAACCACAUUGGGGAUGAAUAAGUGUUAUAUACAGAUCAUGGAUCUUGGACACAUGUGUGAUGCCACCAUGAGCUAUGAAUGCCCUAUGCUGGAUGAGGGG
KU940224    GGCAGCGGAGGUCACUAGACGUGGGAGUGCAUACUAUAUGUACUUGGACAGAAACGAUGCUGGGGAGGCCAUAUCUUUUCCAACCACAUUGGGGAUGAAUAAGUGUUAUAUACAGAUCAUGGAUCUUGGACACAUGUGUGAUGCCACCAUGAGCUAUGAAUGCCCUAUGCUGGAUGAGGGG


            5555555555555555555555555555555555555555555555555555555566666666666666666666666666666666666666666666666666666666666666666666666666666666666666666666666666666666666777777777777777777777777
            4444445555555555666666666677777777778888888888999999999900000000001111111111222222222233333333334444444444555555555566666666667777777777888888888899999999990000000000111111111122222
Sequence    4567890123456789012345678901234567890123456789012345678901234567890123456789012345678901234567890123456789012345678901234567890123456789012345678901234567890123456789012345678901234
---------------------------------------------------------------------------------------------------------------------------------------------------------------------------------------------------------
NC_012532   GUGGAACCAGAUGAUGUCGAUUGCUGGUGCAACACGACAUCAACUUGGGUUGUGUACGGAACCUGUCAUCACAAAAAAGGUGAGGCACGGCGAUCUAGAAGAGCCGUGACGCUCCCUUCUCACUCUACAAGGAAGUUGCAAACGCGGUCGCAGACCUGGUUAGAAUCAAGAGAAUACACGA
KU922960    GUGGAACCAGAUGACGUCGAUUGUUGGUGCAACACGACGUCAACUUGGGUUGUGUACGGAACCUGCCAUCACAAAAAAGGUGAAGCACGGAGAUCUAGAAGAGCCGUGACGCUCCCCUCCCAUUCCACUAGGAAGCUGCAAACGCGGUCGCAAACCUGGUUGGAAUCAAGAGAAUACACAA
KU922923    GUGGAACCAGAUGACGUCGAUUGUUGGUGCAACACGACGUCAACUUGGGUUGUGUACGGAACCUGCCAUCACAAAAAAGGUGAAGCACGGAGAUCUAGAAGAGCCGUGACGCUCCCCUCCCAUUCCACUAGGAAGCUGCAAACGCGGUCGCAAACCUGGUUGGAAUCAAGAGAAUACACAA
KU820898    GUGGAACCAGAUGACGUCGAUUGUUGGUGCAACACGACGUCAACUUGGGUUGUGUACGGAACCUGCCAUCACAAAAAAGGUGAAGCACGGAGAUCUAGAAGAGCUGUGACGCUCCCUUCCCAUUCCACUAGGAAGCUGCAAACGCGGUCGCAAACCUGGUUGGAAUCAAGAGAAUACACAA
KU321639    GUGGAACCAGAUGACGUCGAUUGUUGGUGCAACACGACGUCAACUUGGGUUGUGUACGGAACCUGCCAUCACAAAAAAGGUGAAGCACGGAGAUCUAGAAGAGCUGUGACGCUCCCCUCCCAUUCCACUAGGAAGCUGCAAACGCGGUCGCAAACCUGGUUGGAAUCAAGAGAAUACACAA
KU820899    GUGGAACCAGAUGACGUCGAUUGUUGGUGCAACACGACGUCAACUUGGGUUGUGUACGGAACCUGCCAUCACAAAAAAGGUGAAGCACGGAGAUCUAGAAGAGCUGUGACGCUCCCCUCCCAUUCCACUAGGAAGCUGCAAACGCGGUCGCAAACUUGGUUGGAAUCAAGAGAAUACACAA
KU853012    GUGGAACCAGAUGACGUCGAUUGUUGGUGCAACACGACGUCAACUUGGGUUGUGUACGGAACCUGCCAUCACAAAAAAGGUGAAGCACGGAGAUCUAGAAGAGCUGUGACGCUCCCCUCCCAUUCCACUAGGAAGCUGCAAACGCGGUCGCAAACCUGGUUGGAAUCAAGAGAAUACACAA
KU729217    GUGGAACCAGAUGACGUCGAUUGUUGGUGCAACACGACGUCAACUUGGGUUGUGUACGGAACCUGCCAUCACAAAAAAGGUGAAGCACGGAGAUCUAGAAGAGCUGUGACGCUCCCCUCCCAUUCCACUAGGAAGCUGCAAACGCGGUCGCAAACCUGGUUGGAAUCAAGAGAAUACACAA
KU729218    GUGGAACCAGAUGACGUCGAUUGUUGGUGCAACACGACGUCAACUUGGGUUGUGUACGGAACCUGCCAUCACAAAAAAGGUGAAGCACGGAGAUCUAGAAGAGCUGUGACGCUCCCCUCCCAUUCCACUAGGAAGCUGCAAACGCGGUCGCAAACCUGGUUGGAAUCAAGAGAAUACACAA
KU761564    GUGGAACCAGAUGACGUCGAUUGUUGGUGCAACACGACGUCAACUUGGGUUGUGUACGGAACCUGCCAUCACAAAAAAGGUGAAGCACGGAGAUCUAGAAGAGCUGUGACGCUCCCUUCCCAUUCCACUAGGAAGCUGCAAACGCGGUCGCAAACCUGGUUGGAAUCAAGAGAAUACACAA
KU740184    GUGGAACCAGAUGACGUCGAUUGUUGGUGCAACACGACGUCAACUUGGGUUGUGUACGGAACCUGCCAUCACAAAAAAGGUGAAGCACGGAGAUCUAGAAGAGCUGUGACGCUCCCUUCCCAUUCCACUAGGAAGCUGCAAACGCGGUCGCAAACCUGGUUGGAAUCAAGAGAAUACACAA
KU744693    GUGGAACCAGAUGACGUCGAUUGUUGGUGCAACACGACGUCAACUUGGGUUGUGUACGGAACCUGCCAUCACAAAAAAGGUGAAGCACGGAGAUCUAGAAGAGCUGUGACGCUCCCUUCCCAUUCCACUAGGAAGCUGCAAACGCGGUCGCAAACCUGGUUGGAAUCAAGAGAAUACACAA
KU497555    GUGGAACCAGAUGACGUCGAUUGUUGGUGCAACACGACGUCAACUUGGGUUGUGUACGGAACCUGCCAUCACAAAAAAGGUGAAGCACGGAGAUCUAGAAGAGCUGUGACGCUCCCCUCCCAUUCCACUAGGAAGCUGCAAACGCGGUCGCAAACCUGGUUGGAAUCAAGAGAAUACACAA
KU527068    GUGGAACCAGAUGACGUCGAUUGUUGGUGCAACACGACGUCAACUUGGGUUGUGUACGGAACCUGCCAUCACAAAAAAGGUGAAGCACGGAGAUCUAGAAGAGCUGUGACGCUCCCCUCCCAUUCCACUAGGAAGCUGCAAACGCGGUCGCAAACCUGGUUGGAAUCAAGAGAAUACACAA
KU501217    GUGGAACCAGAUGACGUCGAUUGUUGGUGCAACACGACGUCAACUUGGGUUGUGUACGGAACCUGCCAUCACAAAAAAGGUGAAGCACGGAGAUCUAGAAGAGCUGUGACGCUCCCCUCCCAUUCCACUAGGAAGCUGCAAACGCGGUCGCAAACCUGGUUGGAAUCAAGAGAAUACACAA
KU501216    GUGGAACCAGAUGACGUCGAUUGUUGGUGCAACACGACGUCAACUUGGGUUGUGUACGGAACCUGCCAUCACAAAAAAGGUGAAGCACGGAGAUCUAGAAGAGCUGUGACGCUCCCCUCCCAUUCCACUAGGAAGCUGCAAACGCGGUCGCAAACCUGGUUGGAAUCAAGAGAAUACACAA
KU501215    GUGGAACCAGAUGACGUCGAUUGUUGGUGCAACACGACGUCAACUUGGGUUGUGUACGGAACCUGCCAUCACAAAAAAGGUGAAGCACGGAGAUCUAGAAGAGCUGUGACGCUCCCCUCCCAUUCCACCAGGAAGCUGCAAACGCGGUCGCAAACCUGGUUGGAAUCAAGAGAAUACACAA
KU365780    GUGGAACCAGAUGACGUCGAUUGUUGGUGCAACACGACGUCAACUUGGGUUGUGUACGGAACCUGCCAUCACAAAAAAGGUGAAGCACGGAGAUCUAGAAGAGCUGUGACGCUCCCCUCCCAUUCCACUAGGAAGCUGCAAACGCGGUCGCAAACCUGGUUGGAAUCAAGAGAAUACACAA
KU365779    GUGGAACCAGAUGACGUCGAUUGUUGGUGCAACACGACGUCAACUUGGGUUGUGUACGGAACCUGCCAUCACAAAAAAGGUGAAGCACGGAGAUCUAGAAGAGCUGUGACGCUCCCCUCCCAUUCCACUAGGAAGCUGCAAACGCGGUCGCAAACCUGGUUGGAAUCAAGAGAAUACACAA
KU707826    GUGGAACCAGAUGACGUCGAUUGUUGGUGCAACACGACGUCAACUUGGGUUGUGUACGGAACCUGCCAUCACAAAAAAGGUGAAGCACGGAGAUCUAGAAGAGCUGUGACGCUCCCCUCCCAUUCCACUAGGAAGCUGCAAACGCGGUCGCAAACCUGGUUGGAAUCAAGAGAAUACACAA
KU365778    GUGGAACCAGAUGACGUCGAUUGUUGGUGCAACACGACGUCAACUUGGGUUGUGUACGGAACCUGCCAUCACAAAAAAGGUGAAGCACGGAGAUCUAGAAGAGCUGUGACGCUCCCCUCCCAUUCCACUAGGAAGCUGCAAACGCGGUCGCAAACCUGGUUGGAAUCAAGAGAAUACACAA
KU365777    GUGGAACCAGAUGACGUCGAUUGUUGGUGCAACACGACGUCAACUUGGGUUGUGUACGGAACCUGCCAUCACAAAAAAGGUGAAGCACGGAGAUCUAGAAGAGCUGUGACGCUCCCCUCCCAUUCCACUAGGAAGCUGCAAACGCGGUCGCAAACCUGGUUGGAAUCAAGAGAAUACACAA
KU312312    GUGGAACCAGAUGACGUCGAUUGUUGGUGCAACACGACGUCAACUUGGGUUGUGUACGGAACCUGCCAUCACAAAAAAGGUGAAGCACGGAGAUCUAGAAGAGCUGUGACGCUCCCCUCCCAUUCCACUAGGAAGCUGCAAACGCGGUCGCAAACCUGGUUGGAAUCAAGAGAAUACACAA
KF268950    GUGGAACCAGAUGAUGUCGAUUGCUGGUGCAACACGACAUCAACUUGGGUUGUGUACGGAACCUGUCAUCACAAAAAAGGUGAAGCACGGCGAUCUAGAAGAGCUGUGACGCUCCCUUCUCACUCUACAAGGAAGUUGCAAACGCGGUCGCAGACCUGGUUAGAAUCAAGAGAAUACACGA
KF268949    GUGGAACCAGAUGAUGUCGAUUGCUGGUGCAACACGACAUCAACUUGGGUUGUGUACGGAACCUGUCAUCACAAAAAAGGUGAAGCACGGCGAUCUAGAAGAGCUGUGACGCUCCCUUCUCACUCUACAAGGAAGCUGCAAACGCGGUCGCAGACUUGGUUAGAAUCAAGAGAAUACACGA
KF268948    GUGGAACCAGAUGAUGUCGAUUGCUGGUGCAACACGACAUCAACUUGGGUUGUGUACGGAACCUGUCAUCACAAAAAAGGUGAAGCACGGCGAUCUAGAAGAGCUGUGACGCUCCCUUCUCACUCUACAAGGAAGUUGCAAACGCGGUCGCAGACCUGGUUAGAAUCAAGAGAAUACACGA
KJ776791    GUGGAACCAGAUGACGUCGAUUGUUGGUGCAACACGACGUCAACUUGGGUUGUGUACGGAACCUGCCAUCACAAAAAAGGUGAAGCACGGAGAUCUAGAAGAGCUGUGACGCUCCCCUCCCAUUCCACUAGGAAGCUGCAAACGCGGUCGCAAACCUGGUUGGAAUCAAGAGAAUACACAA
KF383119    GUGGAACCAGAUGAUGUCGAUUGCUGGUGCAACACGACAUCAACUUGGGUUGUGUACGGAACCUGUCAUCACAAAAAAGGUGAGGCACGGCGAUCUAGAAGAGCCGUGACGCUCCCUUCUCACUCUACAAGGAAGUUGCAAACGCGGUCGCAGACCUGGUUAGAAUCAAGAGAAUACACGA
KF383118    GUGGAACCAGAUGAUGUCGAUUGCUGGUGCAACACGACAUCAACUUGGGUUGUGUACGGAACCUGUCAUCACAAAAAAGGUGAGACACGGCGAUCUAGAAGAUCUGUGUCGCUCCGUUAUCACUAUACAAGGAAGUUGCAAACGCGGUCGCAGACAUGGUUAGAAUCAAGAGAAUACAAGA
KF383117    GUGGAACCAGAUGAUGUCGAUUGCUGGUGCAACACGACAUCAACUUGGGUUGUGUACGGAACCUGUCAUCACAAAAAAGGUGAAGCACGGCGAUCUAGAAGAGCUGUGACGCUCCCUUCUCACUCCACAAGGAAGUUGCAAACGCGGUCGCAGACCUGGUUAGAAUCAAGAGAAUACACGA
KF383116    GUGGAGCCAGAUGACGUCGAUUGCUGGUGCAACACGACAUCAACUUGGGUUGUGUACGGAACCUGUCAACAUAAAAAAGGUGAAGCACGACGAUCCAGAAGAGCCGUGACGCUUCCUUCUCACUCCACAAGGAAGCUGCAAACGCGAUCACAGACUUGGCUAGAAUCAAGAGAAUACACAA
KF383115    GUGGAACCAGAUGAUGUCGAUUGCUGGUGCAACACGACAUCAACUUGGGUUGUGUACGGAACCUGUCAUCACAAAAAAGGUGAAGCACGGCGAUCUAGAAGAGCUGUGACGCUCCCUUCUCACUCCACAAGGAAGUUGCAAACGCGGUCGCAGACCUGGUUAGAAUCAAGAGAAUACACGA
EU545988    GUAGAACCAGAUGACGUCGAUUGUUGGUGCAACACGACAUCAACUUGGGUUGUGUACGGAACCUGCCACCACAAAAAAGGUGAAGCACGGAGAUCUAGAAGAGCUGUGACGCUCCCCUCCCAUUCCACUAGGAAGCUGCAAACGCGGUCGCAGACCUGGUUGGAAUCAAGAGAAUAUACAA
KU955589    GUGGAACCAGAUGACGUCGAUUGUUGGUGCAACACGACGUCAACUUGGGUUGUGUACGGAACCUGCCAUCACAAAAAAGGUGAAGCACGGAGAUCUAGAAGAGCUGUGACGCUCCCCUCCCAUUCCACUAGGAAGCUGCAAACGCGGUCGCAAACUUGGUUGGAAUCAAGAGAAUACACAA
KU955590    GUGGAACCAGAUGACGUCGAUUGUUGGUGCAACACGACGUCAACUUGGGUUGUGUACGGAACCUGCCAUCACAAAAAAGGUGAAGCACGGAGAUCUAGAAGAGCUGUGACGCUCCCUUCCCAUUCCACUAGGAAGCUGCAAACGCGGUCGCAAACCUGGUUGGAAUCAAGAGAAUACACAA
KU940228    GUGGAACCAGAUGACGUCGAUUGUUGGUGCAACACGACGUCAACUUGGGUUGUGUACGGAACCUGCCAUCACAAAAAAGGUGAAGCACGGAGAUCUAGAAGAGCUGUGACGCUCCCCUCCCAUUCCACUAGGAAGCUGCAAACGCGGUCGCAAACCUGGUUGGAAUCAAGAGAAUACACAA
KU991811    GUGGAACCAGAUGACGUCGAUUGUUGGUGCAACACGACGUCAACUUGGGUUGUGUACGGAACCUGCCAUCACAAAAAAGGUGAAGCACGGAGAUCUAGAAGAGCUGUGACGCUCCCCUCCCAUUCCACUAGGAAGCUGCAAACGCGGUCGCAAACCUGGUUGGAAUCAAGAGAAUACACAA
KU509998    GUGGAACCAGAUGACGUCGAUUGUUGGUGCAACACGACGUCAACUUGGGUUGUGUACGGAACCUGCCAUCACAAAAAAGGUGAAGCACGGAGAUCUAGAAGAGCUGUGACGCUCCCCUCCCAUUCCACUAGGAAGCUGCAAACGCGGUCGCAAACCUGGUUGGAAUCAAGAGAAUACACAA
KX056898    GUGGAACCAGAUGACGUCGAUUGUUGGUGCAACACGACGUCAACUUGGGUUGUGUACGGAACCUGCCAUCACAAAAAAGGUGAAGCACGGAGAUCUAGAAGAGCUGUGACGCUCCCUUCCCAUUCCACUAGGAAGCUGCAAACGCGGUCGCAAACCUGGUUGGAAUCAAGAGAAUACACAA
KX117076    GUGGAACCAGAUGACGUCGAUUGUUGGUGCAACACGACGUCAACUUGGGUUGUGUACGGAACCUGCCAUCACAAAAAAGGUGAAGCACGGAGAUCUAGAAGAGCUGUGACGCUCCCCUCCCAUUCCACUAGGAAGCUGCAAACGCGGUCGCAAACUUGGUUGGAAUCAAGAGAAUACACAA
KX185891    GUGGAACCAGAUGACGUCGAUUGUUGGUGCAACACGACGUCAACUUGGGUUGUGUACGGAACCUGCCAUCACAAAAAAGGUGAAGCACGGAGAUCUAGAAGAGCUGUGACGCUCCCCUCCCAUUCCACUAGGAAGCUGCAAACGCGGUCGCAAACUUGGUUGGAAUCAAGAGAAUACACAA
```

```
KX247646   GUGGAACCAGAUGACGUCGAUUGUUGGUGCAACACGACGUCAACUUGGGUUGUGUACGGAACCUGCCAUCACAAAAAAGGUGAAGCACGGAGAUCUAGAAGAGCCGUGACGCUCCCCUCCCAUUCCACUAGGAAGCUGCAAACGCGGUCGCAAACCUGGUUGGAAUCAAGAGAAUACACAA
KX262887   GUGGAACCAGAUGACGUCGAUUGUUGGUGCAACACGACGUCAACUUGGGUUGUGUACGGAACCUGCCAUCACAAAAAAGGUGAAGCACGGAGAUCUAGAAGAGCUGUGACGCUCCCCUCCCAUUCCACUAGGAAGCUGCAAACGCGGUCGCAAACCUGGUUGGAAUCAAGAGAAUACACAA
KX280026   GUGGAACCAGAUGACGUCGAUUGUUGGUGCAACACGACGUCAACUUGGGUUGUGUACGGAACCUGCCAUCACAAAAAAGGUGAAGCACGGAGAUCUAGAAGAGCUGUGACGCUCCCCUCCCAUUCCACUAGGAAGCUGCAAACGCGGUCGCAAACCUGGUUGGAAUCAAGAGAAUACACAA
KU758877   GUGGAACCAGAUGACGUCGAUUGUUGGUGCAACACGACGUCAACUUGGGUUGUGUACGGAACCUGCCAUCACAAAAAAGGUGAAGCACGGAGAUCUAGAAGAGCUGUGACGCUCCCCUCCCAUUCCACUAGGAAGCUGCAAACGCGGUCGCAAACCUGGUUGGAAUCAAGAGAAUACACAA
KU866423   GUGGAACCAGAUGACGUCGAUUGUUGGUGCAACACGACGUCAACUUGGGUUGUGUACGGAACCUGCCAUCACAAAAAAGGUGAAGCACGGAGAUCUAGAAGAGCUGUGACGCUCCCCUCCCAUUCCACUAGGAAGCUGCAAACGCGGUCGCAAACUUGGUUGGAAUCAAGAGAAUACACAA
KX377336   GUAGAACCAGAUGACGUCGAUUGCUGGUGCAACACGACAUCGACUUGGGUUGUGUACGGAACCUGCCAUCACAAAAAAGGUGAGGCACGGAGAUCUAGAAGAGCUGUGACGCUCCCCUCUCAUUCCACUAGGAAGCUGCAAACGCGGUCGCAGACCUGGUUGGAAUCAAGAGAAUACACAA
KX377337   GUGGAACCAGAUGACGUCGAUUGUUGGUGCAACACGACGUCAACUUGGGUUGUGUACGGAACCUGCCAUCACAAAAAAGGUGAAGCACGGAGAUCUAGAAGAGCUGUGACGCUCCCCUCCCAUUCCACCAGGAAGCUGCAAACGCGGUCGCAAACCUGGUUGGAAUCAAGAGAAUACACAA
KX369547   GUGGAACCAGAUGACGUCGAUUGUUGGUGCAACACGACGUCAACUUGGGUUGUGUACGGAACCUGCCAUCACAAAAAAGGUGAAGCACGGAGAUCUAGAAGAGCUGUGACGCUCCCCUCCCAUUCCACUAGAAAGCUGCAAACGCGGUCGCAAACCUGGUUGGAAUCAAGAGAAUACACAA
KX548902   GUGGAACCAGAUGACGUCGAUUGUUGGUGCAACACGACGUCAACUUGGGUUGUGUACGGAACCUGCCAUCACAAAAAAGGUGAAGCACGGAGAUCUAGAAGAGCCGUGACGCUCCCCUCCCAUUCCACUAGGAAGCUGCAAACGCGGUCGCAAACCUGGUUGGAAUCAAGAGAAUACACAA
KX601166   GUGGAGCCAGAUGACGUCGAUUGCUGGUGCAACACGACAUCGACUUGGGUUGUGUACGGAACCUGUCAUCAUAAAAAAGGUGAAGCACGACGAUCCAGAAGAGCCGUGACGCUUCCUUCUCACUCCACGAGGAAGCUGCAAACGCGAUCGCAGACUUGGCUAGAGUCAAGAGAAUACACAA
KX601167   GUAGAACCAGAUGACGUCGAUUGCUGGUGCAACACGACAUCGACUUGGGUUGUGUACGGAACCUGCCAUCACAAAAAAGGUGAGGCACGGAGAUCUAGAAGAGCUGUGACGCUCCCCUCUCAUUCCACUAGGAAGCUGCAAACGCGGUCGCAGACCUGGUUGGAAUCAAGAGAAUACACAA
KX601168   GUGGAACCAGAUGACGUCGAUUGUUGGUGCAACACGACGUCAACUUGGGUUGUGUACGGAACCUGCCAUCACAAAAAAGGUGAAGCACGGAGAUCUAGAAGAGCUGUGACGCUCCCCUCCCAUUCCACCAGGAAGCUGCAAACGCGGUCGCAAACCUGGUUGGAAUCAAGAGAAUACACAA
KX766028   GUGGAACCAGAUGACGUCGAUUGUUGGUGCAACACGACGUCAACUUGGGUUGUGUACGGAACCUGCCAUCACAAAAAAGGUGAAGCACGGAGAUCUAGAAGAGCUGUGACGCUCCCUUCCCAUUCCACUAGGAAGCUGCAAACGCGGUCGCAAACCUGGUUGGAAUCAAGAGAAUACACAA
KX766029   GUGGAACCAGAUGACGUCGAUUGUUGGUGCAACACGACGUCAACUUGGGUUGUGUACGGAACCUGCCAUCACAAAAAAGGUGAAGCACGGAGAUCUAGAAGAGCUGUGACGCUCCCCUCCCAUUCCACUAGGAAGCUGCAAACGCGGUCGCAAACCUGGUUGGAAUCAAGAGAAUACACAA
KU940224   GUGGAACCAGAUGACGUCGAUUGUUGGUGCAACACGACGUCAACUUGGGUUGUGUACGGAACCUGCCAUCACAAAAAAGGUGAAGCACGGAGAUCUAGAAGAGCUGUGACGCUCCCCUCCCAUUCCACUAGGAAGCUGCAAACGCGGUCGCAAACCUGGUUGGAAUCAAGAGAAUACACAA


           7777777777777777777777777777777777777777777777777777777777777777777777777778888888888888888888888888888888888888888888888888888888888888888888888888888888888888888888888888888888888999999
           2222233333333334444444444555555555566666666667777777777888888888899999999990000000000111111111122222222223333333333444444444455555555556666666666777777777788888888889999999999000000
Sequence   5678901234567890123456789012345678901234567890123456789012345678901234567890123456789012345678901234567890123456789012345678901234567890123456789012345678901234567890123456789012345
---------------------------------------------------------------------------------------------------------------------------------------------------------------------------------------------------------
NC_012532  AGCACUUGAUCAAGGUUGAAAACUGGAUAUUCAGGAACCCCGGGUUUGCGCUAGUGGCCGUUGCCAUUGCCUGGCUUUUGGGAAGCUCGACGAGCCAAAAAGUCAUAUACUUGGUCAUGAUACUGCUGAUUGCCCCGGCAUACAGUAUCAGGUGCAUUGGAGUCAGCAAUAGAGACUUCGU
KU922960   AGCACUUGAUUAGAGUCGAAAAUUGGAUAUUCAGGAACCCUGGUUUCGCUUUAGCAGCAGCUGCCAUCGCGUGGCUUUUGGGAAGCUCAACGAGCCAAAAAGUCAUAUACUUGGUCAUGAUACUGCUGAUUGCCCCGGCAUACAGCAUCAGGUGCAUAGGAGUCAGCAAUAGGGACUUUGU
KU922923   AGCACUUGAUUAGAGUCGAAAAUUGGAUAUUCAGGAACCCUGGUUUCGCUUUAGCAGCAGCUGCCAUCGCGUGGCUUUUGGGAAGCUCAACGAGCCAAAAAGUCAUAUACUUGGUCAUGAUACUGCUGAUUGCCCUGGCAUACAGCAUCAGGUGCAUAGGAGUCAGCAAUAGGGACUUUGU
KU820898   AGCACUUGAUUAGAGUCGAAAAUUGGAUAUUCAGGAACCCUGGCUUCGCGUUAGCAGCAGCUGCUAUCGCUUGGCUUUUGGGAAGCUCAACGAGCCAAAAAGUCAUAUACUUGGUCAUGAUACUGCUGAUUGCCCCGGCAUACAGCAUCAGGUGCAUAGGAGUCAGCAAUAGGGACUUUGU
KU321639   AGCACUUGAUUAGAGUCGAAAAUUGGAUAUUCAGGAACCCUGGCUUCGCGUUAGCAGCAGCUGCCAUCGCUUGGCUUUUGGGAAGCUCAACGAGCCAAAAAGUCAUAUACUUGGUCAUGAUACUGCUGAUUGCCCCGGCAUACAGCAUCAGGUGCAUAGGAGUCAGCAAUAGGGACUUUGU
KU820899   AGCACUUGAUUAGAGUCGAAAAUUGGAUAUUCAGGAACCCUGGCUUCGCGUUAGCAGCAGCUGCCAUCGCUUGGCUUUUGGGAAGCUCAACGAGCCAAAAAGUCAUAUACUUGGUCAUGAUACUGCUGAUUGCCCCGGCAUACAGCAUCAGGUGCAUAGGAGUCAGCAAUAGGGACUUUGU
KU853012   AGCACUUGAUUAGAGUCGAAAAUUGGAUAUUCAGGAACCCUGGCUUCGCGUUAGCAGCAGCUGCCAUCGCUUGGCUUUUGGGAAGCUCAACGAGCCAAAAAGUCAUAUACUUGGUCAUGAUACUGCUGAUUGCCCCGGCAUACAGCAUCAGGUGCAUAGGAGUCAGCAAUAGGGACUUUGU
KU729217   AGCACUUGAUUAGAGUCGAAAAUUGGAUAUUCAGGAACCCUGGCUUCGCGUUAGCAGCAGCUGCCAUCGCUUGGCUUUUGGGAAGCUCAACGAGCCAAAAAGUCAUAUACUUGGUCAUGAUACUGCUGAUUGCCCCGGCAUACAGCAUCAGGUGCAUAGGAGUCAGCAAUAGGGACUUUGU
KU729218   AGCACUUGAUUAGAGUCGAAAAUUGGAUAUUCAGGAACCCUGGCUUCGCGUUAGCAGCAGCUGCCAUCGCUUGGCUUUUGGGAAGCUCAACGAGCCAAAAAGUCAUAUACUUGGUCAUGAUACUGCUGAUUGCCCCGGCAUACAGCAUCAGGUGCAUAGGAGUCAGCAAUAGGGACUUUGU
KU761564   AGCACUUGAUUAGAGUCGAAAAUUGGAUAUUCAGGAACCCUGGCUUCGCGUUAGCAGCAGCUGCUAUCGCUUGGCUUUUGGGAAGCUCAACGAGCCAAAAAGUCAUAUACUUGGUCAUGAUACUGCUGAUUGCCCCGGCAUACAGCAUCAGGUGCAUAGGAGUCAGCAAUAGGGACUUUGU
KU740184   AGCACUUGAUUAGAGUCGAAAAUUGGAUAUUCAGGAACCCUGGCUUCGCGUUAGCAGCAGCUGCUAUCGCUUGGCUUUUGGGAAGCUCAACGAGCCAAAAAGUCAUAUACUUGGUCAUGAUACUGCUGAUUGCCCCGGCAUACAGCAUCAGGUGCAUAGGAGUCAGCAAUAGGGACUUUGU
KU744693   AGCACUUGAUUAGAGUCGAAAAUUGGAUAUUCAGGAACCCUGGCUUCGCGUUAGCAGCAGCUGCCAUCGCUUGGCUUUUGGGAAGCUCAACGAGCCAAAAAGUCAUAUACUUGGUCAUGAUACUGCUGAUUGCCCCGGCAUACAGCAUCAGGUGCAUAGGAGUCAGCAAUAGGGACUUUGU
KU497555   AGCACUUGAUUAGAGUCGAAAAUUGGAUAUUCAGGAACCCUGGCUUCGCGUUAGCAGCAGCUGCCAUCGCUUGGCUUUUGGGAAGCUCAACGAGCCAAAAAGUCAUAUACUUGGUCAUGAUACUGCUGAUUGCCCCGGCAUACAGCAUCAGGUGCAUAGGAGUCAGCAAUAGGGACUUUGU
KU527068   AGCACUUGAUUAGAGUCGAAAAUUGGAUAUUCAGGAACCCUGGCUUCGCGUUAGCAGCAGCUGCCAUCGCUUGGCUUUUGGGAAGCUCAACGAGCCAAAAAGUCAUAUACUUGGUCAUGAUACUGCUGAUUGCCCCGGCAUACAGCAUCAGGUGCAUAGGAGUCAGCAAUAGGGACUUUGU
KU501217   AGCACUUGAUUAGAGUCGAAAAUUGGAUAUUCAGGAACCCUGGCUUCGCGUUAGCAGCAGCUGCCAUCGCUUGGCUUUUGGGAAGCUCAACGAGCCAAAAAGUCAUAUACUUGGUCAUGAUACUGCUGAUUGCCCCGGCAUACAGCAUCAGGUGCAUAGGAGUCAGCAAUAGGGACUUUGU
KU501216   AGCACUUGAUUAGAGUCGAAAAUUGGAUAUUCAGGAACCCUGGCUUCGCGUUAGCAGCAGCUGCCAUCGCUUGGCUUUUGGGAAGCUCAACGAGCCAAAAAGUCAUAUACUUGGUCAUGAUACUGCUGAUUGCCCCGGCAUACAGCAUCAGGUGCAUAGGAGUCAGCAAUAGGGACUUUGU
KU501215   AGCACUUGAUUAGAGUCGAAAAUUGGAUAUUCAGGAACCCUGGCUUCGCGUUAGCAGCAGCUGCCAUCGCUUGGCUUUUGGGAAGCUCAACGAGCCAAAAAGUCAUAUACUUGGUCAUGAUACUGCUGAUUGCCCCGGCAUACAGCAUCAGGUGCAUAGGAGUCAGCAAUAGGGACUUUGU
KU365780   AGCACUUGAUUAGAGUCGAAAAUUGGAUAUUUAGGAACCCUGGCUUCGCGUUAGCAGCAGCUGCCAUCGCUUGGCUUUUGGGAAGCUCAACGAGCCAAAAAGUCAUAUACUUGGUCAUGAUACUGCUGAUUGCCCCGGCAUACAGCAUCAGGUGCAUAGGAGUCAGCAAUAGGGACUUUGU
KU365779   AGCACUUGAUUAGAGUCGAAAAUUGGAUAUUCAGGAACCCUGGCUUCGCGUUAGCAGCAGCUGCCAUCGCUUGGCUUUUGGGAAGCUCAACGAGCCAAAAAGUCAUAUACUUGGUCAUGAUACUGCUGAUUGCCCCGGCAUACAGCAUCAGGUGCAUAGGAGUCAGCAAUAGGGACUUUGU
KU707826   AGCACUUGAUUAGAGUCGAAAAUUGGAUAUUCAGGAACCCUGGCUUCGCGUUAGCAGCAGCUGCCAUCGCUUGGCUUUUGGGAAGCUCAACGAGCCAAAAAGUCAUAUACUUGGUCAUGAUACUGCUGAUUGCCCCGGCAUACAGCAUCAGGUGCAUAGGAGUCAGCAAUAGGGACUUUGU
KU365778   AGCACUUGAUUAGAGUCGAAAAUUGGAUAUUCAGGAACCCUGGCUUCGCGUUAGCAGCAGCUGCCAUCGCUUGGCUUUUGGGAAGCUCAACGAGCCAAAAAGUCAUAUACUUGGUCAUGAUACUGCUGAUUGCCCCGGCAUACAGCAUCAGGUGCAUAGGAGUCAGCAAUAGGGACUUUGU
KU365777   AGCACUUGAUUAGAGUCGAAAAUUGGAUAUUUAGGAACCCUGGCUUCGCGUUAGCAGCAGCUGCCAUCGCUUGGCUUUUGGGAAGCUCAACGAGCCAAAAAGUCAUAUACUUGGUCAUGAUACUGCUGAUUGCCCCGGCAUACAGCAUCAGGUGCAUAGGAGUCAGCAAUAGGGACUUUGU
KU312312   AGCACUUGAUUAGAGUCGAAAAUUGGAUAUUCAGGAACCCUGGCUUCGCGUUAGCAGCAGCUGCCAUCGCUUGGCUUUUGGGAAGCUCAACGAGCCAAAAAGUCAUAUACUUGGUCAUGAUACUGCUGAUUGCCCCGGCAUACAGCAUCAGGUGCAUAGGAGUCAGCAAUAGGGACUUUGU
KF268950   AGCACCUGAUCAAGGUUGAAAACUGGAUAUUUAGGAACCCCGGGUUUGCGCUCGCAGCUGUUGCCAUUGCCUGGCUUUUGGGAAGCUCGACGAGCCAAAAAGUCAUAUACUUGGUUAUGAUACUGCUGAUUGCCCCGGCAUACAGCAUCAGGUGCAUUGGAGUCAGCAAUAGAGACUUCGU
KF268949   AGCACUUGAUCAAGGUUGAAAACUGGAUAUUCAGGAACCCCGGGUUUGCGCUCGCAGCUGUUGCCAUUGCCUGGCUUUUGGGAAGCUCGACGAGCCAAAAAGUCAUAUACUUGGUCAUGAUACUGCUGAUUGCCCCGGCAUACAGCAUCAGGUGCAUUGGAGUCAGCAAUAGAGACUUCGU
KF268948   AGCACCUGAUCAAGGUUGAAAACUGGAUAUUUAGGAACCCCGGGUUUGCGCUCGCAGCUGUUGCCAUUGCCUGGCUUUUGGGAAGCUCGACGAGCCAAAAAGUCAUAUACUUGGUUAUGAUACUGCUGAUUGCCCCGGCAUACAGCAUCAGGUGCAUUGGAGUCAGCAAUAGAGACUUCGU
KJ776791   AGCACUUGAUUAGAGUCGAAAAUUGGAUAUUCAGGAACCCUGGCUUCGCGUUAGCAGCAGCUGCCAUCGCUUGGCUUUUGGGAAGCUCAACGAGCCAAAAAGUCAUAUACUUGGUCAUGAUACUGCUGAUUGCCCCGGCAUACAGCAUCAGGUGCAUAGGAGUCAGCAAUAGGGACUUUGU
KF383119   AGCACUUGAUCAAGGUUGAAAACUGGAUAUUCAGGAACCCCGGGUUUGCGCUAGUGGCCGUUGCCAUUGCCUGGCUUUUGGGAAGCUCGACGAGCCAAAAAGUCAUAUACUUGGUCAUGAUACUGCUGAUUGCCCCGGCAUACAGUAUCAGGUGCAUUGGAGUCAGCAAUAGAGACUUCGU
KF383118   AGCACUUGAUCAUGGUCGAAAACUGGAUAUUCAGGAACCCCGGGUUUGCCAUAGUGUCCGUUGCCAUUACCUGGCUGAUGGGAAGCUUGACGAGCCAAAAAGUCAUAUACUUGGUCAUGAUAGUGUUGAUUGUCCCGGCAUACAGUAUCAGCUGCAUUGGAGUCAGCAAUAGAGACUUAGU
KF383117   AGCACUUGAUCAAGGUUGAAAACUGGAUAUUCAGGAACCCCGGGUUUGCGCUAGCGGCUGUUGCCAUUGCUUGGCUUCUGGGAAGCUCGACGAGCCAAAAAGUCAUAUACUUGGUCAUGAUAUUGUUGAUUGCCCCGGCAUACAGCAUCAGGUGCAUAGGAGUUAGCAAUAGAGAUUUCGU
KF383116   AGCACCUGAUCAAGGUUGAGAAUUGGAUAUUCAGGAACCCCGGGUUUGCGCUAGUGGCUGUAGCUAUUGCCUGGCUCCUGGGAAGCUCGACGAGCCAAAAAGUCAUAUACUUGGUAAUGAUAUUGUUGAUUGCCCCGGCAUACAGCAUCAGGUGCAUAGGAGUUAGCAAUAGAGACUUCGU
KF383115   AGCACUUGAUCAAGGUUGAAAACUGGAUAUUCAGGAACCCCGGGUUUGCGCUAGCGGCUGUUGCCAUUGCUUGGCUUCUGGGAAGUUCGACGAGCCAAAAAGUCAUAUACUUGAUCAUGAUACUGCUGAUUGCCCCGGCAUACAGCAUCAGGUGCAUUGGAGUCAGCAACAGAGACUUCGU
EU545988   AGCACCUGAUUAGAGUCGAAAAUUGGAUAUUCAGGAACCCUGGCUUCGCGUUAGCAGCAGCUGCCAUCGCCUGGCUUUUGGGAAGUUCAACGAGCCAAAAAGUCAUAUACUUGGUCAUGAUACUGCUGAUUGCCCCGGCAUACAGCAUCAGGUGCAUAGGAGUCAGCAAUAGGGACUUUGU
KU955589   AGCACUUGAUUAGAGUCGAAAAUUGGAUAUUCAGGAACCCUGGCUUCGCGUUAGCAGCAGCUGCCAUCGCUUGGCUUUUGGGAAGCUCAACGAGCCAAAAAGUCAUAUACUUGGUCAUGAUACUGCUGAUUGCCCCGGCAUACAGCAUCAGGUGCAUAGGAGUCAGCAAUAGGGACUUUGU
KU955590   AGCACUUGAUUAGAGUCGAAAAUUGGAUAUUCAGGAACCCUGGCUUCGCGUUAGCAGCAGCUGCCAUCGCUUGGCUUUUGGGAAGCUCAACGAGCCAAAAAGUCAUAUACUUGGUCAUGAUACUGCUGAUUGCCCCGGCAUACAGCAUCAGGUGCAUAGGAGUCAGCAAUAGGGACUUUGU
KU940228   AGCACUUGAUUAGAGUCGAAAAUUGGAUAUUCAGGAACCCUGGCUUCGCGUUAGCAGCAGCUGCCAUCGCUUGGCUUUUGGGAAGCUCAACGAGCCAAAAAGUCAUAUACUUGGUCAUGAUACUGCUGAUUGCCCCGGCAUACAGCAUCAGGUGCAUAGGAGUCAGCAAUAGGGACUUUGU
KU991811   AGCACUUGAUUAGAGUCGAAAAUUGGAUAUUCAGGAACCCUGGCUUCGCGUUAGCAGCAGCUGCCAUCGCUUGGCUUUUGGGAAGCUCAACGAGCCAAAAAGUCAUAUACUUGGUCAUGAUACUGCUGAUUGCCCCGGCAUACAGCAUCAGGUGCAUAGGAGUCAGCAAUAGGGACUUUGU
KU509998   AGCACUUGAUUAGAGUCGAAAAUUGGAUAUUCAGGAACCCUGGCUUCGCGUUAGCAGCAGCUGCCAUCGCUUGGCUUUUGGGAAGCUCAACGAGCCAAAAAGUCAUAUACUUGGUCAUGAUACUGCUGAUUGCCCCGGCAUACAGCAUCAGGUGCAUAGGAGUCAGCAAUAGGGACUUUGU
KX056898   AGCACUUGAUUAGAGUCGAAAAUUGGAUAUUCAGGAACCCUGGCUUCGCGUUAGCAGCAGCUGCCAUCGCUUGGCUUUUGGGAAGCUCAACGAGCCAAAAAGUCAUAUACUUGGUCAUGAUACUGCUGAUUGCCCCGGCAUACAGCAUCAGGUGCAUAGGAGUCAGCAAUAGGGACUUUGU
KX117076   AGCACUUGAUUAGAGUCGAAAAUUGGAUAUUCAGGAACCCUGGCUUCGCGUUAGCAGCAGCUGCCAUCGCUUGGCUUUUGGGAAGCUCAACGAGCCAAAAAGUCAUAUACUUGGUCAUGAUACUGCUGAUUGCCCCGGCAUACAGCAUCAGGUGCAUAGGAGUCAGCAAUAGGGACUUUGU
KX185891   AGCACUUGAUUAGAGUCGAAAAUUGGAUAUUCAGGAACCCUGGCUUCGCGUUAGCAGCAGCUGCCAUCGCUUGGCUUUUGGGAAGCUCAACGAGCCAAAAAGUCAUAUACUUGGUCAUGAUACUGCUGAUUGCCCCGGCAUACAGCAUCAGGUGCAUAGGAGUCAGCAAUAGGGACUUUGU
KX247646   AGCACUUGAUUAGAGUCGAAAAUUGGAUAUUCAGGAACCCUGGUUUCGCUUUAGCAGCAGCUGCCAUCGCUUGGCUUUUGGGAAGCUCAACGAGCCAAAAAGUCAUAUACUUGGUCAUGAUACUGCUGAUUGCCCCGGCAUACAGCAUCAGGUGCAUAGGAGUCAGCAAUAGGGACUUUGU
KX262887   AGCACUUGAUUAGAGUCGAAAAUUGGAUAUUCAGGAACCCUGGCUUCGCGUUAGCAGCAGCUGCCAUCGCUUGGCUUUUGGGAAGCUCAACGAGCCAAAAAGUCAUAUACUUGGUCAUGAUACUGCUGAUUGCCCCGGCAUACAGCAUCAGGUGCAUAGGAGUCAGCAAUAGGGACUUUGU
KX280026   AGCAUUUGAUUAGAGUCGAAAAUUGGAUAUUCAGGAACCCUGGCUUCGCGUUAGCAGCAGCUGCCAUCGCUUGGCUUUUGGGAAGCUCAACAAGCCAAAAAGUCAUAUACUUGGUCAUGAUACUGCUGAUUGCCCCGGCAUACAGCAUCAGGUGCAUAGGAGUCAGCAAUAGGGACUUUGU
KU758877   AGCACUUGAUUAGAGUCGAAAAUUGGAUAUUCAGGAACCCUGGCUUCGCGUUAGCAGCAGCUGCCAUCGCUUGGCUUUUGGGAAGCUCAACGAGCCAAAAAGUCAUAUACUUGGUCAUGAUACUGCUGAUUGCCCCGGCAUACAGCAUCAGGUGCAUAGGAGUCAGCAAUAGGGACUUUGU
KU866423   AGCACUUGAUUAGAGUCGAAAAUUGGAUAUUCAGGAACCCUGGCUUCGCGUUAGCAGCAGCUGCCAUCGCUUGGCUUUUGGGAAGCUCAACGAGCCAAAAAGUCAUAUACUUGGUCAUGAUACUGCUGAUUGCCCCGGCAUACAGCAUCAGGUGCAUAGGAGUCAGCAAUAGGGACUUUGU
KX377336   AGCACUUGAUCAGAGUCGAAAAUUGGAUAUUCAGGAACCCUGGCUUUGCGUUGGCAGCAGCUGCCAUUGCUUGGCUUUUGGGAAGCUCAACGAGCCAAAAAGUCAUAUACUUGGUCAUGAUACUGUUGAUUGCCCCGGCAUACAGUAUCAGGUGCAUAGGAGUCAGCAAUAGGGAUUUUGU
KX377337   AGCACUUGAUUAGAGUCGAAAAUUGGAUAUUCAGGAACCCUGGCUUCGCGUUAGCAGCAGCUGCCAUCGCUUGGCUUUUGGGAAGCUCAACGAGCCAAAAAGUCAUAUACUUGGUCAUGAUACUGCUGAUUGCCCCGGCAUACAGCAUCAGGUGCAUAGGAGUCAGCAAUAGGGACUUUGU
KX369547   AGCACUUGAUUAGAGUCGAAAAUUGGAUAUUCAGGAACCCUGGCCUCGCGUUAGCAGCAGCUGCCAUCGCUUGGCUUUUGGGAAGCUCAACGAGCCAAAAAGUCAUAUACUUGGUCAUGAUACUGCUGAUUGCCCCGGCAUACAGCAUCAGGUGCAUAGGAGUCAGCAAUAGGGACUUUGU
KX548902   AGCACUUGAUUAGAGUCGAAAAUUGGAUAUUCAGGAACCCUGGUUUCGCUUUAGCAGCAGCUGCCAUCGCUUGGCUUUUGGGAAGCUCAACGAGCCAAAAAGUCAUAUACUUGGUCAUGAUACUGCUGAUUGCCCCGGCAUACAGCAUCAGGUGCAUAGGAGUCAGCAAUAGGGACUUUGU
KX601166   AGCACCUGAUCAAGGUUGAAAAUUGGAUAUUCAGGAACCCCGGGUUUGCGCUAGUGGCUGUAGCUAUAGCCUGGCUCCUGGGAAGCUCGACGAGCCAAAAAGUUAUAUACUUGGUCAUGAUAUUGUUGAUUGCCCCGGCAUACAGCAUUAGGUGCAUAGGAGUUAGCAAUAGAGACUUCGU
KX601167   AGCACUUGAUCAGAGUCGAAAAUUGGAUAUUCAGGAACCCUGGCUUUGCGUUGGCAGCAGCUGCCAUUGCUUGGCUUUUGGGAAGCUCAACGAGCCAAAAAGUCAUAUACUUGGUCAUGAUACUGUUGAUUGCCCCGGCAUACAGUAUCAGGUGCAUAGGAGUCAGCAAUAGGGAUUUUGU
KX601168   AGCACUUGAUUAGAGUCGAAAAUUGGAUAUUCAGGAACCCUGGCUUCGCGUUAGCAGCAGCUGCCAUCGCUUGGCUUUUGGGAAGCUCAACGAGCCAAAAAGUCAUAUACUUGGUCAUGAUACUGCUGAUUGCCCCGGCAUACAGCAUCAGGUGCAUAGGAGUCAGCAAUAGGGACUUUGU
KX766028   AGCACUUGAUUAGAGUCGAAAAUUGGAUAUUCAGGAACCCUGGCUUCGCGUUAGCAGCAGCUGCCAUCGCUUGGCUUUUGGGAAGCUCAACGAGCCAAAAAGUCAUAUACUUGGUCAUGAUACUGCUGAUUGCCCCGGCAUACAGCAUCAGGUGCAUAGGAGUCAGCAAUAGGGACUUUGU
KX766029   AGCACUUGAUUAGAGUCGAAAAUUGGAUAUUCAGGAACCCUGGCUUCGCGUUAGCAGCAGCUGCCAUCGCUUGGCUUUUGGGAAGCUCAACGAGCCAAAAAGUCAUAUACUUGGUCAUGAUACUGCUGAUUGCCCCGGCAUACAGCAUCAGGUGCAUAGGAGUCAGCAAUAGGGACUUUGU
KU940224   AGCACUUGAUUAGAGUCGAAAAUUGGAUAUUCAGGAACCCUGGCUUCGCGUUAGCAGCAGCUGCCAUCGCUUGGCUUUUGGGAAGCUCAACGAGCCAAAAAGUCAUAUACUUGGUCAUGAUACUGCUGAUUGCCCCGGCAUACAGCAUCAGGUGCAUAGGAGUCAGCAAUAGGGACUUUGU
```

```
                                                                                                                                                                                                                                                               111111111111111111111111111111111111111111111111111111111111111111111111111111111111111
             9999999999999999999999999999999999999999999999999999999999999999999999999999999999999999999999000000000000000000000000000000000000000000000000000000000000000000000000000000000000000
             0000111111111122222222223333333333444444444455555555556666666666777777777788888888889999999999000000000011111111112222222222333333333344444444445555555555666666666677777777778888888
Sequence     6789012345678901234567890123456789012345678901234567890123456789012345678901234567890123456789012345678901234567890123456789012345678901234567890123456789012345678901234567890123456
--------------------------------------------------------------------------------------------------------------------------------------------------------------------------------------------------

NC_012532    GGAGGGCAUGUCAGGUGGGACCUGGGUUGAUGUUGUCUUGGAACAUGGAGGCUGCGUUACCGUGAUGGCACAGGACAAGCCAACAGUCGACAUAGAGUUGGUCACGACGACGGUUAGUAACAUGGCCGAGGUAAGAUCCUAUUGCUACGAGGCAUCGAUAUCGGACAUGGCUUCGGACAGU
KU922960     GGAAGGUAUGUCAGGUGGGACUUGGGUUGAUGUUGUCUUGGAACAUGGAGGUUGUGUCACCGUAAUGGCACAGGACAAACCGACUGUCGACAUAGAGCUGGUUACAACAACAGUCAGCAACAUGGCGGAGGUAAGAUCCUACUGCUAUGAGGCAUCAAUAUCAGACAUGGCUUCGGACAGC
KU922923     GGAAGGUAUGUCAGGUGGGACUUGGGUUGAUGUUGUCUUGGAACAUGGAGGUUGUGUCACCGUAAUGGCACAGGACAAACCGACUGUCGACAUAGAGCUGGUUACAACAACAGUCAGCAACAUGGCGGAGGUAAGAUCCUACUGCUAUGAGGCAUCAAUAUCAGACAUGGCUUCGGACAGC
KU820898     GGAAGGUAUGUCAGGUGGGACUUGGGUUGAUGUUGUCUUGGAACAUGGAGGUUGUGUCACCGUAAUGGCACAGGACAAACCGACUGUCGACAUAGAGCUGGUUACAACAACAGUCAGCAACAUGGCGGAGGUAAGAUCCUACUGCUAUGAGGCAUCAAUAUCAGACAUGGCUUCGGACAGC
KU321639     GGAAGGUAUGUCAGGUGGGACUUGGGUUGAUAUUGUCUUGGAACAUGGAGGUUGUGUCACCGUAAUGGCACAGGACAAACCGACUGUCGACAUAGAGCUGGUUACAACAACAGUCAGCAACAUGGCGGAGGUAAGAUCCUACUGCUAUGAGGCAUCAAUAUCAGACAUGGCUUCGGACAGC
KU820899     GGAAGGUAUGUCAGGUGGGACUUGGGUUGAUGUUGUCUUGGAACAUGGAGGUUGUGUCACCGUAAUGGCACAGGACAAACCGACUGUCGACAUAGAGCUGGUUACAACAACAGUCAGCAACAUGGCGGAGGUAAGAUCCUACUGCUAUGAGGCAUCAAUAUCGGACAUGGCUUCGGACAGC
KU853012     GGAAGGUAUGUCAGGUGGGACUUGGGUUGAUGUUGUCUUGGAACAUGGAGGUUGUGUCACCGUAAUGGCACAGGACAAACCGACUGUCGACAUAGAGCUGGUUACAACAACAGUCAGCAACAUGGCGGAGGUAAGAUCCUACUGCUAUGAGGCAUCAAUAUCAGACAUGGCUUCGGACAGC
KU729217     GGAAGGUAUGUCAGGUGGGACUUGGGUUGAUGUUGUCUUGGAACAUGGAGGUUGUGUCACCGUAAUGGCACAGGACAAACCGACUGUCGACAUAGAGCUGGUUUCAACAACAGUCAGCAACAUGGCGGAGGUAAGAUCCUACUGCUAUGAGGCAACAAUAUCAGACAUAGCUUCGGACAGC
KU729218     GGAAGGUAUGUCAGGUGGGACUUGGGUUGAUGUUGUCUUGGAACAUGGAGGUUGUGUCACCGUAAUGGCACAGGACAAACCGACUGUCGACAUAGAGCUGGUUACAACAACAGUCAGCAACAUGGCGGAGGUAAGAUCCUACUGCUAUGAGGCAUCAAUAUCAGACAUGGCUUCGGACAGC
KU761564     GGAAGGUAUGUCAGGUGGGACUUGGGUUGAUGUUGUCUUGGAACAUGGAGGUUGUGUCACCGUAAUGGCACAGGACAAACCGACUGUCGACAUAGAGCUGGUUACAACAACAGUCAGCAACAUGGCGGAGGUAAGAUCCUACUGCUAUGAGGCAUCAAUAUCAGACAUGGCUUCGGACAGC
KU740184     GGAAGGUAUGUCAGGUGGGACUUGGGUUGAUGUUGUCUUGGAACAUGGAGGUUGUGUCACCGUAAUGGCACAGGACAAACCGACUGUCGACAUAGAGCUGGUUACAACAACAGUCAGCAACAUGGCGGAGGUAAGAUCCUACUGCUAUGAGGCAUCAAUAUCAGACAUGGCUUCGGACAGC
KU744693     GGAAGGUAUGUCAGGUGGGACUUGGGUUGAUGUUGUCUUGGAACAUGGAGGUUGUGUCACCGCAAUGGCACAGGACAAACCGACUGUCGACAUAGAGCUGGUUACAACAACAGUCAGCAACAUGGCGGAGGUAAGAUCCUACUGCUAUGAGGCAUCAAUAUCAGACAUGGCUUCGGACAGC
KU497555     GGAAGGUAUGUCAGGUGGGACUUGGGUUGAUGUUGUCUUGGAACAUGGGGGUUGUGUCACCGUAAUGGCACAGGACAAACCGACUGUCGACAUAGAGCUGGUUACAACAACAGUCAGCAACAUGGCGGAGGUAAGAUCCUACUGCUAUGAGGCAUCAAUAUCAGACAUGGCUUCGGACAGC
KU527068     GGAAGGUAUGUCAGGUGGGACUUGGGUUGAUGUUGUCUUGGAACAUGGAGGUUGUGUCACCGUAAUGGCACAGGACAAACCGACUGUCGACAUAGAGCUGGUUACAACAACAGUCAGCAACAUGGCGGAGGUAAGAUCCUACUGCUAUGAGGCAUCAAUAUCAGACAUGGCUUCGGACAGC
KU501217     GGAAGGUAUGUCAGGUGGGACUUGGGUUGAUGUUGUCUUGGAACAUGGAGGUUGUGUCACCGUAAUGGCACAGGACAAACCGACUGUCGACAUAGAGCUGGUUACAACAACAGUCAGCAACAUGGCGGAGAUAAGAUCCUACUGCUAUGAGGCAUCAAUAUCAGACAUGGCUUCGGACAGC
KU501216     GGAAGGUAUGUCAGGUGGGACUUGGGUUGAUGUUGUCUUGGAACAUGGAGGUUGUGUCACCGUAAUGGCACAGGACAAACCGACUGUCGACAUAGAGCUGGUUACAACAACAGUCAGCAACAUGGCGGAGAUAAGAUCCUACUGCUAUGAGGCAUCAAUAUCAGACAUGGCUUCGGACAGC
KU501215     GGAAGGUAUGUCAGGUGGGACUUGGGUUGAUGUUGUCUUGGAACAUGGAGGUUGUGUCACCGUAAUGGCACAGGACAAACCGACUGUCGACAUAGAGCUGGUUACAACAACAGUCAGCAACAUGGCGGAGGUAAGAUCCUACUGCUAUGAGGCAUCAAUAUCAGACAUGGCUUCUGACAGC
KU365780     GGAAGGUAUGUCAGGUGGGACCUGGGUUGAUGUUGUCUUGGAACAUGGAGGUUGUGUCACCGUAAUGGCACAGGACAAACCGACUGUCGACAUAGAGCUGGUUACAACAACAGUCAGCAACAUGGCGGAGGUAAGAUCCUACUGCUAUGAGGCAUCAAUAUCAGACAUGGCUUCGGACAGC
KU365779     GGAAGGUAUGUCAGGUGGGACCUGGGUUGAUGUUGUCUUGGAACAUGGAGGUUGUGUCACCGUAAUGGCACAGGACAAACCGACUGUCGACAUAGAGCUGGUUACAACAACAGUCAGCAACAUGGCGGAGGUAAGAUCCUACUGCUAUGAGGCAUCAAUAUCAGACAUGGCUUCGGACAGC
KU707826     GGAAGGUAUGUCAGGUGGGACCUGGGUUGAUGUUGUCUUGGAACAUGGAGGUUGUGUCACCGUAAUGGCACAGGACAAACCGACUGUCGACAUAGAGCUGGUUACAACAACAGUCAGCAACAUGGCGGAGGUAAGAUCCUACUGCUAUGAGGCAUCAAUAUCAGACAUGGCUUCGGACAGC
KU365778     GGAAGGUAUGUCAGGUGGGACUUGGGUUGAUGUUGUCUUGGAACAUGGAGGUUGUGUCACCGUAAUGGCACAGGACAAACCGACUGUCGACAUAGAGCUGGUUACAACAACAGUCAGCAACAUGGCGGAGGUAAGAUCCUACUGCUAUGAGGCAUCAAUAUCAGACAUGGCUUCGGACAGC
KU365777     GGAAGGUAUGUCAGGUGGGACCUGGGUUGAUGUUGUCUUGGAACAUGGAGGUUGUGUCACCGUAAUGGCACAGGACAAACCGACUGUCGACAUAGAGCUGGUUACAACAACAGUCAGCAACAUGGCGGAGGUAAGAUCCUACUGCUAUGAGGCAUCAAUAUCAGACAUGGCUUCGGACAGC
KU312312     GGAAGGUAUGUCAGGUGGGACUUGGGUUGAUGUUGUCUUGGAACAUGGAGGUUGUGUCACUGUAAUGGCACAGGACAAACCGACUGUCGACAUAGAGCUGGUUACAACAACAGUCAGCAACAUGGCGGAGGUAAGAUCCUACUGCUAUGAGGCAUCAAUAUCAGACAUGGCUUCGGACAGC
KF268950     GGAAGGCAUGUCAGGUGGGACCUGGGUUGAUGUCGUCUUGGAACAUGGAGGUUGCGUCACCGUUAUGGCACAGGACAAGCCAACAGUUGACAUAGAGUUGGUCACGACAACGGUUAGUAACAUGGCCGAGGUAAGAUCCUACUGUUAUGAGGCAUCAAUAUCAGACAUGGCUUCGGACAGU
KF268949     GGAAGGCAUGUCAGGUGGGACCUGGGUUGAUGUCGUCUUGGAACAUGGAGGUUGCGUCACCGUAAUGGCACAGGACAAGCCAACAGUUGACAUAGAGUUGGUCACGACAACGGUCAGUAACAUGGCCGAGGUAAGAUCCUACUGUUAUGAGGCAUCAAUAUCGGACAUGGCUUCGGACAGU
KF268948     GGAAGGCAUGUCAGGUGGGACCUGGGUUGAUGUCGUCUUGGAACAUGGAGGUUGCGUCACCGUUAUGGCACAGGACAAGCCAACAGUUGACAUAGAGUUGGUCACGACAACGGUUAGUAACAUGGCCGAGGUAAGAUCCUACUGUUAUGAGGCAUCAAUAUCAGACAUGGCUUCGGACAGU
KJ776791     GGAAGGUAUGUCAGGUGGGACUUGGGUUGAUGUUGUCUUGGAACAUGGAGGUUGUGUCACCGUAAUGGCACAGGACAAACCGACUGUCGACAUAGAGCUGGUUACAACAACAGUCAGCAACAUGGCGGAGGUAAGAUCCUACUGCUAUGAGGCAUCAAUAUCGGACAUGGCUUCGGACAGC
KF383119     GGAGGGCAUGUCAGGUGGGACCUGGGUUGAUGUUGUCUUGGAACAUGGAGGCUGCGUUACCGUGAUGGCACAGGACAAGCCAACAGUUGACAUAGAGUUGGUCACGACGACGGUUAGUAACAUGGCCGAGGUAAGAUCCUAUUGCUACGAGGCAUCGAUAUCGGACAUGGCUUCGGACAGU
KF383118     GGAGGGCAUGUCAGGUGGGACCUGGGUUGAUGUUGUCUUGGAACAUGGAGGGUGCGUUACCGAGAUGGCACAGGACAAGCCAACAGUUGACAUAGAGUUGGUCACGAUGACGGUUAGUAACAUGGCCGAGGUAAGAUCCUAUUGCUACGAGGCAUCGUUAUCCGACAUGGCUUCGGCCAGU
KF383117     AGAGGGCAUGUCAGGUGGGACCUGGGUUGAUGUCGUCUUGGAACAUGGGGGUUGCGUCACCGUGAUGGCACAGGACAAGCCAACAGUUGACAUCGAGUUGGUCACGACAACGGUUAGCAACAUGGCCGAGGUAAGAUCCUACUGCUAUGAGGCAUCAAUAUCGGACAUGGCCUCGGACAGC
KF383116     GGAGGGCAUGUCAGGUGGGACCUGGGUUGAUGUUGUCUUGGAACAUGGGGGUUGCGUCACCGUGAUGGCACAGGACAAGCCAACAGUUGACAUCGAGUUGGUCACGACAACGGUUAGCAACAUGGCCGAGGUAAGAUCCUACUGCUAUGAGGCAUCAAUAUCGGACAUGGCUUCGGACAGC
KF383115     GGAAGGCAUGUCAGGUGGGACCUGGGUUGAUGUCGUCUUGGAACAUGGAGGUUGCGUCACCGUAAUGGCACAGGACAAGCCAACAGUUGACAUAGAGUUGGUCACGACAACGGUUAGCAACAUGGCUGAGGUAAGAUCCUACUGCUACGAGGCAUCAAUAUCGGACAUGGCUUCGGAUAGU
EU545988     GGAAGGUAUGUCAGGUGGGACUUGGGUUGAUGUUGUCUUGGAACAUGGAGGUUGUGUUACCGUAAUGGCACAGGACAAACCGGCUGUCGACAUAGAGCUGGUUACAACAACAGUCAGCAACAUGGCGGAGGUAAGAUCCUAUUGCUAUGAGGCAUCAAUAUCGGACAUGGCUUCGGACAGC
KU955589     GGAAGGUAUGUCAGGUGGGACUUGGGUUGAUGUUGUCUUGGAACAUGGAGGUUGUGUCACCGUAAUGGCACAGGACAAACCGACUGUCGACAUAGAGCUGGUUACAACAACAGUCAGCAACAUGGCGGAGGUAAGAUCCUACUGCUAUGAGGCAUCAAUAUCGGACAUGGCUUCGGACAGC
KU955590     GGAAGGUAUGUCAGGUGGGACUUGGGUCGAUGUUGUCUUGGAACAUGGAGGUUGUGUCACCGUAAUGGCACAGGACAAACCGACUGUCGACAUAGAGCUGGUUACAACAACAGUCAGCAACAUGGCGGAGGUAAGAUCCUACUGCUAUGAGGCAUCAAUAUCAGACAUGGCUUCGGACAGC
KU940228     GGAAGGUAUGUCAGGUGGGACUUGGGUUGAUGUUGUCUUGGAACAUGGAGGUUGUGUCACCGUAAUGGCACAGGACAAACCGACUGUCGACAUAGAGCUGGUUACAACAACAGUCAGCAACAUGGCGGAGGUAAGAUCCUACUGCUAUGAGGCAUCAAUAUCAGACAUGGCUUCGGACAGC
KU991811     GGAAGGUAUGUCAGGUGGGACUUGGGUUGAUGUUGUCUUGGAACAUGGGGGUUGUGUCACCGUAAUGGCACAGGACAAACCGACUGUCGACAUAGAGCUGGUUACAACAACAGUCAGCAACAUGGCGGAGGUAAGAUCCUACUGCUAUGAGGCAUCAAUAUCAGACAUGGCUUCGGACAGC
KU509998     GGAAGGUAUGUCAGGUGGGACUUGGGUUGAUGUUGUCUUGGAACAUGGAGGUUGUGUCACCGUAAUGGCACAGGACAAACCGACUGUCGACAUAGAGCUGGUUACAACAACAGUCAGCAACAUGGCGGAGGUAAGAUCCUACUGCUAUGAGGCAUCAAUAUCAGACAUGGCUUCGGACAGC
KX056898     GGAAGGUAUGUCAGGUGGGACUUGGGUCGAUGUUGUCUUGGAACAUGGAGGUUGUGUCACCGUAAUGGCACAGGACAAACCGACUGUCGACAUAGAGCUGGUUACAACAACAGUCAGCAACAUGGCGGAGGUAAGAUCCUACUGCUAUGAGGCAUCAAUAUCAGACAUGGCUUCGGACAGC
KX117076     GGAAGGUAUGUCAGGUGGGACUUGGGUUGAUGUUGUCUUGGAACAUGGAGGUUGUGUCACCGUAAUGGCACAGGACAAACCGACUGUCGACAUAGAGCUGGUUACAACAACAGUCAGCAACAUGGCGGAGGUAAGAUCCUACUGCUAUGAGGCAUCAAUAUCGGACAUGGCUUCGGACAGC
KX185891     GGAAGGUAUGUCAGGUGGGACUUGGGUUGAUGUUGUCUUGGAACAUGGAGGUUGUGUCACCGUAAUGGCACAGGACAAACCGACUGUCGACAUAGAGCUGGUUACAACAACAGUCAGCAACAUGGCGGAGGUAAGAUCCUACUGCUAUGAGGCAUCAAUAUCGGACAUGGCUUCGGACAGC
KX247646     GGAAGGUAUGUCAGGUGGGACUUGGGUUGAUGUCGUCUUGGAACAUGGAGGUUGUGUCACCGUAAUGGCACAGGACAAACCGACUGUCGACAUAGAGCUGGUUACAACAACAGUCAGCAACAUGGCGGAGGUAAGAUCCUACUGCUAUGAGGCAUCAAUAUCAGACAUGGCUUCGGACAGC
KX262887     GGAAGGUAUGUCAGGUGGGACUUGGGUUGAUGUUGUCUUGGAACAUGGAGGUUGUGUCACCGUAAUGGCACAGGACAAACCGACUGUCGACAUAGAGCUGGUUACAACAACAGUCAGCAACAUGGCGGAGGUAAGAUCCUACUGCUAUGAGGCAUCAAUAUCAGACAUGGCUUCGGACAGC
KX280026     GGAAGGUAUGUCAGGUGGGACUUGGGUUGAUGUUGUCUUGGAACAUGGAGGUUGUGUCACCGUAAUGGCACAGGACAAACCGACUGUCGACAUAGAGCUGGUUACAACAACAGUCAGCAACAUGGCGGAGGUAAGAUCCUACUGCUAUGAGGCAUCAAUAUCAGACAUGGCUUCGGACAGC
KU758877     GGAAGGUAUGUCAGGUGGGACUUGGGUUGAUGUUGUCUUGGAACAUGGAGGUUGUGUCACCGUAAUGGCACAGGACAAACCGACUGUCGACAUAGAGCUGGUUACAACAACAGUCAGCAACAUGGCGGAGGUAAGAUCCUACUGCUAUGAGGCAUCAAUAUCAGACAUGGCUUCGGACAGC
KU866423     GGAAGGUAUGUCAGGUGGGACUUGGGUUGAUGUUGUCUUGGAACAUGGAGGUUGUGUCACCGUAAUGGCACAGGACAAACCGACUGUCGACAUAGAGCUGGUUACAACAACAGUCAGCAACAUGGCGGAGGUAAGAUCCUACUGCUAUGAGGCAUCAAUAUCGGACAUGGCUUCGGACAGC
KX377336     GGAAGGUAUGUCAGGUGGGACCUGGGUUGAUGUUGUCUUGGAACAUGGAGGUUGUGUUACCGUAAUGGCACAGGACAAGCCAACUGUUGAUAUAGAGUUGGUCACAACAACGGUUAGCAACAUGGCGGAGGUAAGAUCCUACUGCUACGAGGCAUCAAUAUCGGACAUGGCUUCGGACAGC
KX377337     GGAAGGUAUGUCAGGUGGGACUUGGGUUGAUGUUGUCUUGGAACAUGGAGGUUGUGUCACCGUAAUGGCACAGGACAAACCGACUGUCGACAUAGAGCUGGUUACAACAACAGUCAGCAACAUGGCGGAGGUAAGAUCCUACUGCUAUGAGGCAUCAAUAUCAGACAUGGCUUCUGACAGC
KX369547     GGAAGGUAUGUCAGGUGGGACUUGGGUUGAUGUUGUCUUGGAACAUGGAGGUUGUGUCACCGUAAUGGCACAGGACAAACCGACUGUCGACAUAGAGCUGGUUACAACAACAGUCAGCAACAUGGCGGAGGUAAGAUCCUACUGCUAUGAGGCAUCAAUAUCAGACAUGGCUUCGGACAGC
KX548902     GGAAGGUAUGUCAGGUGGGACUUGGGUUGAUGUUGUCUUGGAACAUGGAGGUUGUGUCACCGUAAUGGCACAGGACAAACCGACUGUCGACAUAGAGCUGGUUACAACAACAGUCAGCAACAUGGCGGAGGUAAGAUCCUACUGCUAUGAGGCAUCAAUAUCAGACAUGGCUUCGGACAGC
KX601166     GGAGGGCAUGUCAGGUGGGACCUGGGUUGAUGUUGUCUUGGAACAUGGGGGUUGCGUCACCGUGAUGGCACAGGACAAGCCAACAGUUGAUAUCGAGUUGGUCACGACAACGGUUAGCAACAUGGCCGAGGUAAGAUCCUACUGCUAUGAGGCAUCAAUAUCGGACAUGGCUUCGGACAGC
KX601167     GGAAGGUAUGUCAGGUGGGACCUGGGUUGAUGUUGUCUUGGAACAUGGAGGUUGUGUUACCGUAAUGGCACAGGACAAGCCAACUGUUGAUAUAGAGUUGGUCACAACAACGGUUAGCAACAUGGCGGAGGUAAGAUCCUACUGCUACGAGGCAUCAAUAUCGGACAUGGCUUCGGACAGC
KX601168     GGAAGGUAUGUCAGGUGGGACUUGGGUUGAUGUUGUCUUGGAACAUGGAGGUUGUGUCACCGUAAUGGCACAGGACAAACCGACUGUCGACAUAGAGCUGGUUACAACAACAGUCAGCAACAUGGCGGAGGUAAGAUCCUACUGCUAUGAGGCAUCAAUAUCAGACAUGGCUUCUGACAGC
KX766028     GGAAGGUAUGUCAGGUGGGACUUGGGUUGAUGUUGUCUUGGAACAUGGAGGUUGUGUCACCGUAAUGGCACAGGACAAACCGACUGUCGACAUAGAGCUGGUUACAACAACAGUCAGCAACAUGGCGGAGGUAAGAUCCUACUGCUAUGAGGCAUCAAUAUCAGACAUGGCUUCGGACAGC
KX766029     GGAAGGUAUGUCAGGUGGGACUUGGGUUGAUGUUGUCUUGGAACAUGGAGGUUGUGUCACCGUAAUGGCACAGGACAAACCGACUGUCGACAUAGAGCUGGUCACAACAACAGUCAGCAACAUGGCGGAGGUAAGAUCCUACUGCUAUGAGGCAUCAAUAUCAGACAUGGCUUCGGACAGC
KU940224     GGAAGGUAUGUCAGGUGGGACUUGGGUUGAUGUUGUCUUGGAACAUGGAGGUUGUGUCACCGUAAUGGCACAGGACAAACCGACUGUCGACAUAGAGCUGGUUACAACAACAGUCAGCAACAUGGCGGAGGUAAGAUCCUACUGCUAUGAGGCAUCAAUAUCAGACAUGGCUUCGGACAGC


             1111111111111111111111111111111111111111111111111111111111111111111111111111111111111111111111111111111111111111111111111111111111111111111111111111111111111111111111111111111111111
             0000000000000111111111111111111111111111111111111111111111111111111111111111111111111111111111111111111111111222222222222222222222222222222222222222222222222222222222222222222222
             8889999999999000000000011111111112222222222333333333344444444445555555555666666666677777777778888888888999999999900000000001111111111222222222233333333334444444444555555555566666666
Sequence     7890123456789012345678901234567890123456789012345678901234567890123456789012345678901234567890123456789012345678901234567890123456789012345678901234567890123456789012345678901234567
--------------------------------------------------------------------------------------------------------------------------------------------------------------------------------------------------

NC_012532    CGUUGCCCAACACAAGGUGAAGCCUACCUUGACAAGCAAUCAGACACUCAAUAUGUCUGCAAAAGAACAUUAGUGGACAGAGGUUGGGGAAACGGUUGUGGACUUUUUGGCAAAGGGAGCUUGGUGACAUGUGCCAAGUUUACGUGUUCUAAGAAGAUGACCGGGAAGAGCAUUCAACCGG
KU922960     CGCUGCCCAACACAAGGUGAAGCCUACCUUGACAAGCAAUCAGACACUCAAUAUGUCUGCAAAAGAACGUUAGUGGACAGAGGCUGGGGAAAUGGAUGUGGACUUUUUGGCAAAGGGAGCCUGGUGACAUGCGCUAAGUUUGCAUGCUCCAAGAAAAUGACCGGGAAGAGCAUCCAGCCAG
KU922923     CGCUGCCCAACACAAGGUGAAGCCUACCUUGACAAGCAAUCAGACACUCAAUAUGUCUGCAAAAGAACGUUAGUGGACAGAGGCUGGGGAAAUGGAUGUGGACUUUUUGGCAAAGGGAGCCUGGUGACAUGCGCUAAGUUUGCAUGCUCCAAGAAAAUGACCGGGAAGAGCAUCCAGCCAG
KU820898     CGCUGCCCAACACAAGGUGAAGCCUACCUUGACAAGCAAUCAGACACUCAAUAUGUCUGCAAAAGAACGUUAGUGGACAGAGGCUGGGGAAAUGGAUGUGGACUUUUUGGCAAAGGGAGCCUGGUGACAUGCGCUAAGUUUGCAUGCUCCAAGAAAAUGACCGGGAAAAGCAUCCAGCCAG
KU321639     CGCUGCCCAACACAAGGUGAAGCCUACCUUGACAAGCAAUCAGACACUCAAUAUGUCUGCAAAAGAACGUUAGUGGACAGAGGCUGGGGAAAUGGAUGUGGACUUUUUGGCAAAGGGAGUCUGGUGACAUGCGCUAAGUUUGCAUGCUCCAAGAAAAUGACCGGGAAGAGCAUCCAGCCAG
KU820899     CGCUGCCCAACACAAGGUGAAGCCUACCUUGACAAGCAAUCAGACACUCAAUAUGUCUGCAAAAGAACGUUAGUGGACAGAGGCUGGGGAAAUGGAUGUGGACUUUUUGGCAAAGGGAGCCUGGUGACAUGCGCUAAGUUUGCAUGCUCCAAGAAAAUGACCGGGAAGAGCAUCCAGCCAG
KU853012     CGCUGCCCAACACAAGGUGAAGCCUACCUUGACAAGCAAUCAGACACUCAAUAUGUCUGCAAAAGAACGUUAGUGGACAGAGGCUGGGGAAAUGGAUGUGGACUUUUUGGCAAAGGGAGCCUGGUGACAUGCGCUAAGUUUGCAUGCUCCAAGAAAAUGACCGGGAAGAGCAUCCAGCCAG
KU729217     CGCUGCCCAACACAAGGUGAAGCCUACCUUGACAAGCAAUCAGACACUCAAUAUGUCUGCAAAAGAACGUUAGUGGACAGAGGCUGGGGAAAUGGAUGUGGACUUUUUGGCAAAGGGAGCCUGGUGACAUGCGCUAAGUUUGCAUGCUCCAAGAAAAUGACCGGGAAGAGCAUCCAGCCAG
```

```
KU729218    CGCUGCCCAACACAAGGUGAAGCCUACCUUGACAAGCAAUCAGACACUCAAUAUGUCUGCAAAAGAACGUUAGUGGACAGAGGCUGGGGAAAUGGAUGUGGACUUUUUGGCAAAGGGAGCCUGGUGACAUGCGCUAAGUUUGCAUGCUCCAAGAAAAUGACCGGGAAGAGCAUCCAGCCAG
KU761564    CGCUGCCCAACACAAGGUGAAGCCUACCUUGACAAGCAAUCAGACACUCAAUAUGUCUGCAAAAGAACGUUAGUGGACAGAGGCUGGGGAAAUGGAUGUGGACUUUUUGGCAAAGGGAGCCUGGUGACAUGCGCUAAGUUUGCAUGCUCCAAGAAAAUGACCGGGAAAAGCAUCCAGCCAG
KU740184    CGCUGCCCAACACAAGGUGAAGCCUACCUUGACAAGCAAUCAGACACUCAAUAUGUCUGCAAAAGAACGUUAGUGGACAGAGGCUGGGGAAAUGGAUGUGGACUUUUUGGCAAAGGGAGCCUGGUGACAUGCGCUAAGUUUGCAUGCUCCAAGAAAAUGACCGGGAAAAGCAUCCAGCCAG
KU744693    CGCUGCCCAACACAAGGUGAAGCCUACCUUGACAAGCAAUCAGACACUCAAUAUGUUUGCAAAAGAACGUUAGUGGACAGAGGCUGGGGAAAUGGAUGUGGACUUUUUGGCAAAGGGAGUCUGGUGACAUGCGCUAAGUUUGCAUGCUCCAAGAAAAUGACCGGGAAGAGCAUCCAGCCAG
KU497555    CGCUGCCCAACACAAGGUGAAGCCUACCUUGACAAGCAAUCAGACACUCAAUAUGUCUGCAAAAGAACGUUAGUGGACAGAGGCUGGGGAAAUGGAUGUGGACUUUUUGGCAAAGGGAGCCUGGUGACAUGCGCUAAGUUUGCAUGCUCCAAGAAAAUGACCGGGAAGAGCAUCCAGCCAG
KU527068    CGCUGCCCAACACAAGGUGAAGCCUACCUUGACAAGCAAUCAGACACUCAAUAUGUCUGCAAAAGAACGUUAGUGGACAGAGGCUGGGGAAAUGGAUGUGGACUUUUUGGCAAAGGGAGCCUGGUGACAUGCGCUAAGUUUGCAUGCUCCAAGAAAAUGACCGGGAAGAGCAUCCAGCCAG
KU501217    CGCUGCCCAACACAAGGUGAAGCCUACCUUGACAAGCAAUCAGACACUCAAUAUGUCUGCAAAAGAACGUUAGUGGACAGAGGCUGGGGAAAUGGAUGUGGACUUUUUGGCAAAGGGAGCCUGGUGACAUGCGCUAAGUUUGCAUGCUCCAAGAAAAUGACCGGGAAGAGCAUCCAGCCAG
KU501216    CGCUGCCCAACACAAGGUGAAGCCUACCUUGACAAGCAAUCAGACACUCAAUAUGUCUGCAAAAGAACGUUAGUGGACAGAGGCUGGGGAAAUGGAUGUGGACUUUUUGGCAAAGGGAGCCUGGUGACAUGCGCUAAGUUUGCAUGCUCCAAGAAAAUGACCGGGAAGAGCAUCCAGCCAG
KU501215    CGCUGCCCAACACAAGGUGAAGCCUACCUUGACAAGCAAUCAGACACUCAAUAUGUCUGCAAAAGAACGUUAGUGGACAGAGGCUGGGGAAAUGGAUGUGGACUUUUUGGCAAAGGGAGCCUGGUGACAUGCGCUAAGUUUGCAUGCUCCAAGAAAAUGACCGGGAAGAGCAUCCAGCCAG
KU365780    CGCUGCCCAACACAAGGUGAAGCCUACCUUGACAAGCAAUCAGACACUCAAUAUGUCUGCAAAAGAACGUUAGUGGACAGAGGCUGGGGAAAUGGAUGUGGACUUUUUGGCAAAGGGAGCCUGGUGACAUGCGCUAAGUUUGCAUGCUCCAAGAAAAUGACCGGGAAGAGCAUCCAGCCAG
KU365779    CGCUGCCCAACACAAGGUGAAGCCUACCUUGACAAGCAAUCAGACACUCAAUAUGUCUGCAAAAGAACGUUAGUGGACAGAGGCUGGGGAAAUGGAUGUGGACUUUUUGGCAAAGGGAGCCUGGUGACAUGCGCUAAGUUUGCAUGCUCCAAGAAAAUGACCGGGAAGAGCAUCCAGCCAG
KU707826    CGCUGCCCAACACAAGGUGAAGCCUACCUUGACAAGCAAUCAGACACUCAAUAUGUCUGCAAAAGAACGUUAGUGGACAGAGGCUGGGGAAAUGGAUGUGGACUUUUUGGCAAAGGGAGCCUGGUGACAUGCGCUAAGUUUGCAUGCUCCAAGAAAAUGACCGGGAAGAGCAUCCAGCCAG
KU365778    CGCUGCCCAACACAAGGUGAAGCCUACCUUGACAAGCAAUCAGACACUCAAUAUGUCUGCAAAAGAACGUUAGUGGACAGAGGCUGGGGAAAUGGAUGUGGACUUUUUGGCAAAGGGAGCCUGGUGACAUGCGCUAAGUUUGCAUGCUCCAAGAAAAUGACCGGGAAGAGCAUCCAGCCAG
KU365777    CGCUGCCCAACACAAGGUGAAGCCUACCUUGACAAGCAAUCAGACACUCAAUAUGUCUGCAAAAGAACGUUAGUGGACAGAGGCUGGGGAAAUGGAUGUGGACUUUUUGGCAAAGGGAGCCUGGUGACAUGCGCUAAGUUUGCAUGCUCCAAGAAAAUGACCGGGAAGAGCAUCCAGCCAG
KU312312    CGCUGCCCAACACAAGGUGAAGCCUACCUUGACAAGCAAUCAGACACUCAAUAUGUCUGCAAAAGAACGUUAGUGGACAGAGGCUGGGGAAAUGGAUGUGGACUUUUUGGCAAAGGGAGCCUGGUGACAUGCGCUAAGUUUGCAUGCUCCAAGAAAAUGACCGGGAAGAGCAUCCAGCCAG
KF268950    CGCUGCCCAACACAAGGUGAAGCCUACCUUGAUAAGCAAUCAGACACCCAAUAUGUGUGCAAAAGAACAUUGGUGGACAGAGGUUGGGGAAAUGGGUGUGGACUUUUUGGCAAAGGGAGCCUGGUGACAUGUGCCAAGUUCACGUGUUCCAAGAAGAUGACUGGGAAGAGCAUUCAGCCGG
KF268949    CGCUGCCCAACACAAGGUGAAGCCUACCUUGACAAGCAAUCAGACACUCAAUAUGUAUGCAAAAGAACAUUGGUGGACAGAGGUUGGGGAAAUGGGUGUGGACUUUUUGGCAAAGGGAGCCUGGUGACAUGUGCCAAGUUCACGUGUUCCAAGAAGAUGACCGGGAAGAGCAUUCAGCCGG
KF268948    CGCUGCCCAACACAAGGUGAAGCCUACCUUGAUAAGCAAUCAGACACCCAAUAUGUGUGCAAAAGAACAUUGGUGGACAGAGGUUGGGGAAAUGGGUGUGGACUUUUUGGCAAAGGGAGCCUGGUGACAUGUGCCAAGUUCACGUGUUCCAAGAAGAUGACUGGGAAGAGCAUUCAGCCGG
KJ776791    CGCUGCCCAACACAAGGUGAAGCCUACCUUGACAAGCAAUCAGACACUCAAUAUGUCUGCAAAAGAACGUUAGUGGACAGAGGCUGGGGAAAUGGAUGUGGACUUUUUGGCAAAGGGAGCCUGGUGACAUGCGCUAAGUUUGCAUGCUCCAAGAAAAUGACCGGGAAGAGCAUCCAGCCAG
KF383119    CGUUGCCCAACACAAGGUGAAGCCUACCUUGACAAGCAAUCAGACACUCAAUAUGUCUGCAAAAGAACAUUAGUGGACAGAGGUUGGGGAAAUGGUUGUGGACUUUUUGGCAAAGGGAGCUUGGUGACAUGUGCCAAGUUCACGUGUUCUAAGAAGAUGACCGGGAAGAGCAUUCAACCGG
KF383118    CGUUGCCCAACACAAGGCGAACCCUCCCUCGACAAGCAAUCAGACACUCAAUCUGUAUGCAAAAGAACAUUAGGAGACAGAGGUUGGGGAAAUGGUUGUGGAUUUUUGGCAAAGGGAGCUUGGUGACAUGUUCCAAGUUCACGUGUUGUAAGAAGAUGCCCGGGAAGAGCAUUCAACCGG
KF383117    CGCUGUCCAACACAAGGUGAAGCCUACCUUGACAAGCAAUCAGACACUCAAUAUGUCUGCAAAAGAACAUUAGUGGACAGAGGUUGGGGAAACGGUUGUGGACUUUUUGGCAAAGGGAGCUUGGUGACAUGUGCCAAGUUUACGUGUUCUAAGAAGAUGACCGGGAAGAGCAUUCAGCCGG
KF383116    CGCUGUCCAACACAAGGUGAAGCCUACCUUGACAAGCAGUCAGACACUCAAUAUGUCUGCAAGAGAACAUUGGUGGAUAGAGGUUGGGGAAAUGGGUGUGGACUUUUUGGCAAAGGGAGCUUGGUGACAUGUGCCAAGUUUACGUGCUCCAAGAAAAUGACAGGCAAGAGCAUCCAGCCGG
KF383115    CGCUGCCCAACACAAGGUGAAGCCUACCUUGACAAGCAAUCAGACACUCAAUAUGUUUGCAAAAGAACAUUGGUGGACAGAGGUUGGGGAAAUGGGUGUGGACUUUUUGGCAAAGGGAGCCUGGUGACAUGUGCCAAGUUCACGUGUUCCAAGAAGAUGACCGGGAAGAGCAUCCAGCCGG
EU545988    CGCUGCCCAACACAAGGUGAAGCCUACCUUGACAAGCAGUCAGACACUCAAUAUGUCUGCAAAAGAACGUUAGUGGACAGAGGCUGGGGAAAUGGAUGUGGACUUUUUGGCAAAGGGAGCCUGGUGACAUGCGCUAAGUUUGCAUGCUCCAAGAAAAUGACCGGGAAGAGCAUCCAGCCAG
KU955589    CGCUGCCCAACACAAGGUGAAGCCUACCUUGACAAGCAAUCAGACACUCAAUAUGUCUGCAAAAGAACGUUAGUGGACAGAGGCUGGGGAAAUGGAUGUGGACUUUUUGGCAAAGGGAGCCUGGUGACAUGCGCUAAGUUUGCAUGCUCCAAGAAAAUGACCGGGAAGAGCAUCCAGCCAG
KU955590    CGCUGCCCAACACAAGGUGAAGCCUACCUUGACAAGCAAUCAGACACUCAAUAUGUCUGCAAAAGAACGUUAGUGGACAGAGGCUGGGGAAAUGGAUGUGGACUUUUUGGCAAAGGGAGCCUGGUGACAUGCGCUAAGUUUGCAUGCUCCAAGAAAAUGACCGGGAAGAGCAUCCAGCCAG
KU940228    CGCUGCCCAACACAAGGUGAAGCCUACCUUGACAAGCAAUCAGACACUCAAUAUGUCUGCAAAAGAACGUUAGUGGACAGAGGCUGGGGAAAUGGAUGUGGACUUUUUGGCAAAGGGAGCCUGGUGACAUGCGCUAAGUUUGCAUGCUCCAAGAAAAUGACCGGGAAGAGCAUCCAGCCAG
KU991811    CGCUGCCCAACACAAGGUGAAGCCUACCUUGACAAGCAAUCAGACACUCAAUAUGUCUGCAAAAGAACGUUAGUGGACAGAGGCUGGGGAAAUGGAUGUGGACUUUUUGGCAAAGGGAGCCUGGUGACAUGCGCUAAGUUUGCAUGCUCCAAGAAAAUGACCGGGAAGAGCAUCCAGCCAG
KU509998    CGCUGCCCAACACAAGGUGAAGCCUACCUUGACAAGCAAUCAGACACUCAAUAUGUCUGCAAAAGAACGUUAGUGGACAGAGGCUGGGGAAAUGGAUGUGGACUUUUUGGCAAAGGGAGUCUGGUGACAUGCGCUAAGUUUGCAUGCUCCAAGAAAAUGACCGGGAAGAGCAUCCAGCCAG
KX056898    CGCUGCCCAACACAAGGUGAAGCCUACCUUGACAAGCAAUCAGACACUCAAUAUGUCUGCAAAAGAACGUUAGUGGACAGAGGCUGGGGAAAUGGAUGUGGACUUUUUGGCAAAGGGAGCCUGGUGACAUGCGCUAAGUUUGCAUGCUCCAAGAAAAUGACCGGGAAGAGCAUCCAGCCAG
KX117076    CGCUGCCCAACACAAGGUGAAGCCUACCUUGACAAGCAAUCAGACACUCAAUAUGUCUGCAAAAGAACGUUAGUGGACAGAGGCUGGGGAAAUGGAUGUGGACUUUUUGGCAAAGGGAGCCUGGUGACAUGCGCUAAGUUUGCAUGCUCCAAGAAAAUGACCGGGAAGAGCAUCCAGCCAG
KX185891    CGCUGCCCAACACAAGGUGAAGCCUACCUUGACAAGCAAUCAGACACUCAAUAUGUCUGCAAAAGAACGUUAGUGGACAGAGGCUGGGGAAAUGGAUGUGGACUUUUUGGCAAAGGGAGCCUGGUGACAUGCGCUAAGUUUGCAUGCUCCAAGAAAAUGACCGGGAAGAGCAUCCAGCCAG
KX247646    CGCUGCCCAACACAAGGUGAAGCCUACCUUGACAAGCAAUCAGACACUCAAUAUGUCUGCAAAAGAACGUUAGUGGACAGAGGCUGGGGAAAUGGAUGUGGACUUUUUGGCAAAGGGAGCCUGGUGACAUGCGCUAAGUUUGCAUGCUCCAAGAAAAUGACCGGGAAGAGCAUCCAGCCAG
KX262887    CGCUGCCCAACACAAGGUGAAGCCUACCUUGACAAGCAAUCAGACACUCAAUAUGUCUGCAAAAGAACGUUAGUGGACAGAGGCUGGGGAAAUGGAUGUGGACUUUUUGGCAAAGGGAGCCUGGUGACAUGCGCUAAGUUUGCAUGCUCCAAGAAAAUGACCGGGAAGAGCAUCCAGCCAG
KX280026    CGCUGCCCAACACAAGGUGAAGCCUACCUUGACAAGCAAUCAGACACUCAAUAUGUCUGCAAAAGAACGUUAGUGGACAGAGGCUGGGGAAAUGGAUGUGGACUUUUUGGCAAAGGGAGCCUGGUGACAUGCGCUAAGUUUGCAUGCUCCAAGAAAAUGACCGGGAAGAGCAUCCAGCCAG
KU758877    CGCUGCCCAACACAAGGUGAAGCCUACCUUGACAAGCAAUCAGACACUCAAUAUGUCUGCAAAAGAACGUUAGUGGACAGAGGCUGGGGAAAUGGAUGUGGACUUUUUGGCAAAGGGAGCCUGGUGACAUGCGCUAAGUUUGCAUGCUCCAAGAAAAUGACCGGGAAGAGCAUCCAGCCAG
KU866423    CGCUGCCCAACACAAGGUGAAGCCUACCUUGACAAGCAAUCAGACACUCAAUAUGUCUGCAAAAGAACGUUAGUGGACAGAGGCUGGGGAAAUGGAUGUGGACUUUUUGGCAAAGGGAGCCUGGUGACAUGCGCUAAGUUUGCAUGCUCCAAGAAAAUGACCGGGAAGAGCAUCCAGCCAG
KX377336    CGCUGCCCAACACAAGGUGAAGCCUACCUUGACAAGCAGUCAGACACUCAAUAUGUUUGCAAAAGAACGUUAGUGGACAGAGGUUGGGGAAAUGGAUGUGGACUCUUUGGCAAAGGGAGCCUGGUGACAUGCGCCAAGUUUGCAUGCUCCAAGAAAAUGACUGGGAAGAGCAUCCAGCCAG
KX377337    CGCUGCCCAACACAAGGUGAAGCCUACCUUGACAAGCAAUCAGACACUCAAUAUGUCUGCAAAAGAACGUUAGUGGACAGAGGCUGGGGAAAUGGAUGUGGACUUUUUGGCAAAGGGAGCCUGGUGACAUGCGCUAAGUUUGCAUGCUCCAAGAAAAUGACCGGGAAGAGCAUCCAGCCAG
KX369547    CGCUGCCCAACACAAGGUGAAGCCUACCUUGACAAGCAAUCAGACACUCAAUAUGUCUGCAAAAGAACGUUAGUGGACAGAGGCUGGGGAAAUGGAUGUGGACUUUUUGGCAAAGGGAGCCUGGUGACAUGCGCUAAGUUUGCAUGCUCCAAGAAAAUGACCGGGAAGAGCAUCCAGCCAG
KX548902    CGCUGCCCAACACAAGGUGAAGCCUACCUUGACAAGCAAUCAGACACUCAAUAUGUCUGCAAAAGAACGUUAGUGGACAGAGGCUGGGGAAAUGGAUGUGGACUUUUUGGCAAAGGGAGCCUGGUGACAUGCGCUAAGUUUGCAUGCUCCAAGAAAAUGACCGGGAAGAGCAUCCAGCCAG
KX601166    CGUUGUCCAACACAAGGUGAAGCCUACCUUGACAAGCAGUCAGACACUCAAUAUGUCUGCAAGAGAACAUUGGUGGAUAGAGGUUGGGGAAAUGGGUGUGGACUUUUUGGCAAAGGGAGCUUGGUGACAUGUGCCAAGUUUACGUGCUCCAAGAAAAUGACAGGCAAGAGCAUCCAGCCGG
KX601167    CGCUGCCCAACACAAGGUGAAGCCUACCUUGACAAGCAGUCAGACACUCAAUAUGUUUGCAAAAGAACGUUAGUGGACAGAGGUUGGGGAAAUGGAUGUGGACUCUUUGGCAAAGGGAGCCUGGUGACAUGCGCCAAGUUUGCAUGCUCCAAGAAAAUGACUGGGAAGAGCAUCCAGCCAG
KX601168    CGCUGCCCAACACAAGGUGAAGCCUACCUUGACAAGCAAUCAGACACUCAAUAUGUCUGCAAAAGAACGUUAGUGGACAGAGGCUGGGGAAAUGGAUGUGGACUUUUUGGCAAAGGGAGCCUGGUGACAUGCGCUAAGUUUGCAUGCUCCAAGAAAAUGACCGGGAAGAGCAUCCAGCCAG
KX766028    CGCUGCCCAACACAAGGUGAAGCCUACCUUGACAAGCAAUCAGACACUCAAUAUGUCUGCAAAAGAACGUUAGUGGACAGAGGCUGGGGAAAUGGAUGUGGACUUUUUGGCAAAGGGAGCCUGGUGACAUGCGCUAAGUUUGCAUGCUCCAAGAAAAUGACCGGGAAGAGCAUCCAGCCAG
KX766029    CGCUGCCCAACACAAGGUGAAGCCUACCUUGACAAGCAAUCAGACACUCAAUAUGUCUGUAAAAGAACGUUAGUGGACAGAGGCUGGGGAAAUGGAUGUGGACUUUUUGGCAAAGGGAGCCUGGUGACAUGCGCUAAGUUUGCUUGCUCCAAGAAAAUGACCGGGAAGAGCAUCCAGCCAG
KU940224    CGCUGCCCAACACAAGGUGAAGCCUACCUUGACAAGCAAUCAGACACUCAAUAUGUCUGCAAAAGAACGUUAGUGGACAGAGGCUGGGGAAAUGGAUGUGGACUUUUUGGCAAAGGGAGCCUGGUGACAUGCGCUAAGUUUGCAUGCUCCAAGAAAAUGACCGGGAAGAGCAUCCAGCCAG


            1111111111111111111111111111111111111111111111111111111111111111111111111111111111111111111111111111111111111111111111111111111111111111111111111111111111111111111111111111111111111
            2222222222222222222222222222222233333333333333333333333333333333333333333333333333333333333333333333333333333333333333333333333334444444444444444444444444444444444444444444444444
            6677777777778888888888999999999900000000001111111111222222222233333333334444444444555555555566666666667777777777888888888899999999990000000000111111111122222222223333333333444444444
Sequence    8901234567890123456789012345678901234567890123456789012345678901234567890123456789012345678901234567890123456789012345678901234567890123456789012345678901234567890123456789012345678
-------------------------------------------------------------------------------------------------------------------------------------------------------------------------------------------------------------
NC_012532   AAAAUCUGGAGUAUCGGAUAAUGCUAUCAGUGCAUGGCUCCCAGCAUAGCGGGAUG------------AUUGGAUAUGAAACUGACGAAGAUAGAGCGAAAGUCGAGGUUACGCCUAAUUCACCAAGAGCGGAAGCAACCUUGGGAGGCUUUGGAAGCUUAGGACUUGACUGUGAACCAAG
KU922960    AGAAUCUGGAGUACCGGAUAAUGUUGUCAGUUCAUGGCUCCCAGCACAGUGGGAUGAUCGUUAAUGACACAGGACAUGAAACUGAUGAGAAUAGAGCGAAGGUUGAGAUAACGCCCAAUUCACCAAGAGCCGAAGCCACCCUGGGGGGUUUUGGAAGCCUAGGACUUGAUUGUGAACCGAG
KU922923    AGAAUCUGGAGUACCGGAUAAUGUUGUCAGUUCAUGGCUCCCAGCACAGUGGGAUGAUCGUUAAUGACACAGGACAUGAAACUGAUGAGAAUAGAGCGAAGGUUGAGAUAACGCCCAAUUCACCAAGAGCCGAAGCCACCCUGGGGGGUUUUGGAAGCCUAGGACUUGAUUGUGAACCGAG
KU820898    AGAAUCUGGAGUACCGGAUAAUGCUGUCAGUUCAUGGCUCCCAGCACAGUGGGAUGAUCGUUAAUGACACAGGACAUGAAACUGAUGAGAAUAGAGCGAAAGUUGAGAUAACGCCCAAUUCACCAAGAGCCGAAGCCACCCUGGGGGGUUUUGGAAGCCUAGGACUUGAUUGUGAACCGAG
KU321639    AGAAUCUGGAGUACCGGAUAAUGCUGUCAGUUCAUGGCUCCCAGCACAGUGGGAUGAUCGUUAAUGACACAGGACAUGAAACUGAUGAGAAUAGAGCGAAGGUUGAGAUAACGCCCAAUUCACCAAGAGCCGAAGCCACCCUGGGGGGUUUUGGAAGCCUAGGACUUGAUUGUGAACCGAG
KU820899    AGAAUCUGGAGUACCGGAUAAUGCUGUCAGUUCAUGGCUCCCAGCACAGUGGGAUGAUCGUUAAUGACACAGGACAUGAAACUGAUGAGAAUAGAGCGAAGGUUGAGAUAACGCCCAAUUCACCAAGAGCCGAAGCCACCCUGGGGGGUUUUGGAAGCCUAGGACUUGAUUGUGAACCGAG
KU853012    AGAAUCUGGAGUACCGGAUAAUGCUGUCAGUUCAUGGCUCCCAGCACAGUGGGAUGAUCGUUAAUGACACUGGACAUGAAACUGAUGAGAAUAGAGCGAAGGUUGAGAUAACGCCCAAUUCACCAAGAGCCGAAGCCACCCUGGGGGGUUUUGGAAGCCUAGGACUUGAUUGUGAACCGAG
KU729217    AGAAUCUGGAGUACCGGAUAAUGCUGUCAGUUCAUGGCUCCCAGCACAGUGGGAUGAUCGUUAAUGACACAGGACAUGAAACUGAUGAGAAUAGAGCGAAGGUUGAGAUAACGCCCAAUUCACCAAGAGCCGAAGCCACCCUGGGGGGUUUUGGAAGCCUAGGACUUGAUUGUGAACCGAG
KU729218    AGAAUCUGGAGUACCGGAUAAUGCUGUCAGUUCAUGGCUCCCAGCACAGUGGGAUGAUCGUUAAUGACACAGGACAUGAAACUGAUGAGAAUAGAGCGAAGGUUGAGAUAACGCCCAAUUCACCAAGAGCCGAAGCCACCCUGGGGGGUUUUGGAAGCCUAGGACUUGAUUGUGAACCGAG
KU761564    AGAAUCUGGAGUACCGGAUAAUGCUGUCAGUUCAUGGCUCCCAGCACAGUGGGAUGAUCGUUAAUGACACAGGACAUGAAACUGAUGAGAAUAGAGCGAAAGUUGAGAUAACGCCCAAUUCACCAAGAGCCGAAGCCACCCUGGGGGGUUUUGGAAGCCUAGGACUUGAUUGUGAACCGAG
KU740184    AGAAUCUGGAGUACCGGAUAAUGCUGUCAGUUCAUGGCUCCCAGCACAGUGGGAUGAUCGUUAAUGACACAGGACAUGAAACUGAUGAGAAUAGAGCGAAAGUUGAGAUAACGCCCAAUUCACCAAGAGCCGAAGCCACCCUGGGGGGUUUUGGAAGCCUAGGACUUGAUUGUGAACCGAG
KU744693    AGAAUCUGGAGUACCGGAUAAUGCUGUCAGUUCAUGGCUCCCAGCACAGUGGGAUGCUCGUUAAUGACACAGGACAUGAAACUGAUGAGAAUAGAGCGAAGGUUGAGAUAACGCCCAAUUCACCAAGAGCCGAAGCCACCCUGGGGGGUUUUGGAAGCCUAGGACUUGAUUGUGAACCGAG
KU497555    AGAAUCUGGAGUACCGGAUAAUGCUGUCAGUUCAUGGCUCCCAGCACAGUGGGAUGAUCGUUAAUGACACAGGACAUGAAACUGAUGAGAAUAGAGCGAAGGUUGAGAUAACGCCCAAUUCACCAAGAGCCGAAGCCACCCUGGGGGGUUUUGGAAGCUUAGGACUUGAUUGUGAACCGAG
KU527068    AGAAUCUGGAGUACCGGAUAAUGCUGUCAGUUCAUGGCUCCCAGCACAGUGGGAUGAUCGUUAAUGACACAGGACAUGAAACUGAUGAGAAUAGAGCGAAGGUUGAGAUAACGCCCAAUUCACCAAGAGCCGAAGCCACCCUGGGGGGUUUUGGAAGCCUAGGACUUGAUUGUGAACCGAG
KU501217    AGAAUCUGGAGUACCGGAUAAUGCUGUCAGUUCAUGGCUCCCAGCACAGUGGGAUGAUCGUUAAUGACACAGGACAUGAAACUGAUGAGAAUAGAGCGAAGGUUGAGAUAACGCCCAAUUCACCAAGAGCCGAAGCCACCCUGGGGGGUUUUGGAAGCCUAGGACUUGAUUGUGAACCGAG
KU501216    AGAAUCUGGAGUACCGGAUAAUGCUGUCAGUUCAUGGCUCCCAGCACAGUGGGAUGAUCGUUAAUGACACAGGACAUGAAACUGAUGAGAAUAGAGCGAAGGUUGAGAUAACGCCCAAUUCACCAAGAGCCGAAGCCACCCUGGGGGGUUUUGGAAGCCUAGGACUUGAUUGUGAACCGAG
KU501215    AGAAUCUGGAGUACCGGAUAAUGCUGUCAGUUCAUGGCUCCCAGCACAGUGGGAUGAUCGUUAAUGACACAGGACAUGAAACUGAUGAGAAUAGAGCGAAAGUUGAGAUAACGCCCAAUUCACCGAGAGCCGAAGCCACCCUGGGGGGUUUUGGAAGCCUAGGACUUGAUUGUGAACCGAG
KU365780    AGAAUCUGGAGUACCGGAUAAUGCUGUCAGUUCAUGGCUCCCAGCACAGUGGGAUGAUUGUUAAUGACACAGGACAUGAAACUGAUGAGAAUAGAGCGAAAGUUGAGAUAACGCCCAAUUCACCAAGAGCCGAAGCCACCCUGGGGGGUUUUGGAAGCCUAGGACUUGAUUGUGAACCGAG
KU365779    AGAAUCUGGAGUACCGGAUAAUGCUGUCAGUUCAUGGCUCCCAGCACAGUGGGAUGAUUGUUAAUGACACAGGACAUGAAACUGAUGAGAAUAGAGCGAAAGUUGAGAUAACGCCCAAUUCACCAAGAGCCGAAGCCACCCUGGGGGGUUUUGGAAGCCUAGGACUUGAUUGUGAACCGAG
KU707826    AGAAUCUGGAGUACCGGAUAAUGCUGUCAGUUCAUGGCUCCCAGCACAGUGGGAUGAUUGUUAAUGACACAGGACAUGAAACUGAUGAGAAUAGAGCGAAAGUUGAGAUAACGCCCAAUUCACCAAGAGCCGAAGCCACCCUGGGGGGUUUUGGAAGCCUAGGACUUGAUUGUGAACCGAG
KU365778    AGAAUCUGGAGUACCGGAUAAUGCUGUCAGUUCAUGGCUCCCAGCACAGUGGGAUGAUCGUUAAUGACACAGGACAUGAAACUGAUGAGAAUAGAGCGAAAGUUGAGAUAACGCCCAAUUCACCAAGAGCCGAAGCCACCCUGGGGGGUUUUGGAAGCCUAGGACUUGAUUGUGAACCGAG
KU365777    AGAAUCUGGAGUACCGGAUAAUGCUGUCAGUUCAUGGCUCCCAGCACAGUGGGAUGAUUGUUAAUGACACAGGACAUGAAACUGAUGAGAAUAGAGCGAAAGUUGAGAUAACGCCCAAUUCACCAAGAGCCGAAGCCACCCUGGGGGGUUUUGGAAGCCUAGGACUUGAUUGUGAACCGAG
KU312312    AGAAUCUGGAGUACCGGAUAAUGCUGUCAGUUCAUGGCUCCCAGCACAGUGGGAUGAUCGUUAAUGACACAGGACAUGAAACUGAUGAGAAUAGAGCGAAAGUUGAGAUAACGCCCAAUUCACCAAGAGCCGAAGCCACCCUGGGGGGUUUUGGAAGCCUAGGACUUGAUUGUGAACCGAG
```

```
KF268950    AGAAUCUGGAGUAUCGAAUAAUGCUAUCAGUGCAUGGCUCCCAGCACAGCGGGAUGAUUGUUAAUGACAUAGGACAUGAAACUGACGAAAACAGAGCGAAAGUCGAGGUCACGCCCAAUUCACCAAGAGCAGAAGCAACCUUGGGAGGCUUUGGAAGCUUAGGACUUGAUUGUGAACCAAG
KF268949    AGAAUCUGGAGUAUCGAAUAAUGCUAUCAGUGCAUGGCUCCCAGCACAGCGGGAUGAUUGUUAAU------------------GACGAAAAUAGGGCGAAAGUCGAGGUCACGCCCAAUUCACCAAGAGCAGAAGCAACCUUGGGAGGCUUUGGAAGCUUAGGACUUGAUUGUGAACCAAG
KF268948    AGAAUCUGGAGUAUCGAAUAAUGCUAUCAGUGCAUGGCUCCCAGCACAGCGGGAUGAUUGUUAAUGACAUAGGACAUGAAACUGACGAAAACAGAGCGAAAGUCGAGGUCACGCCCAAUUCACCAAGAGCAGAAGCAACCUUGGGAGGCUUUGGAAGCUUAGGACUUGAUUGUGAACCAAG
KJ776791    AGAAUCUGGAGUACCGGAUAAUGCUGUCAGUUCAUGGCUCCCAGCACAGUGGGAUGAUCGUUAAUGACACAGGACAUGAAACUGAUGAGAAUAGAGCGAAGGUUGAGAUAACGCCCAAUUCACCAAGAGCCGAAGCCACCCUGGGGGGUUUUGGAAGCCUAGGACUUGAUUGUGAACCGAG
KF383119    AAAAUCUGGAGUAUCGGAUAAUGCUAUCAGUGCAUGGCUCCCAGCAUAGCGGGAUGAUUGUCAAUGAUAUAGGACAUGAAACUGACGAAAACAGAGCGAAAGUCGAGGUUACGCCUAAUUCACCAAGAGCGGAAGCAACCUUGGGAGGCUUUGGAAGCUUAGGACUUGACUGUGAACCAAG
KF383118    AAAAUCUGGAGUAUCGGAUAAUGCUCCCAGUGCAUGGCUCCCAGCAUAGCGGGAUGAUUGUGAAUGACAUAGGACAUGAAACUGACGAAAACAGAGCGAAAGUCGAGGUCACACCCAAUUCACCAAGAGCAGAAGCAACCUUGGGAGGUUUUGGAAGCUUGGGACUUGACUGUGAACCAAG
KF383117    AGAACUUGGAGUACCGGAUAAUGCUAUCAGUGCAUGGAUCCCAGCACAGUGGGAUG???????????????GGACAUGAAACUGACGAAAACAGAGCAAAGGUCGAGGUCACACCCAAUUCACCAAGAGCAGAAGCAACCUUGGGAGGUUUUGGAAGCUUGGGACUUGACUGUGAACCAAG
KF383116    AGAACUUGGAGUACCGGAUAAUGCUAUCAGUGCAUGGAUCCCAGCACAGUGGGAUGAUUGUGAAUGACAUAGGACAUGAAACUGACGAAAACAGAGCAAAAGUCGAGGUCACACCCAAUUCACCAAGAGCAGAAGCAACCUUGGGAGGUUUUGGAAGCUUGGGACUUGACUGUGAACCAAG
KF383115    AGAAUCUGGAGUAUCGAAUAAUGCUAUCGGUGCAUGGCUCCCAGCACAGCGGGAUGAUUGUUAAUGAC?????????????????????AACAGAGCGGAAGUCGAGGUCACGCCUAAUUCACCAAGAGCAGAAGCAACCUUGGGAGGCUUUGGAAGUUUAGGACUUGAUUGUGAACCAAG
EU545988    AGAAUCUGGAGUACCGGAUAAUGCUGUCAGUUCAUGGCUCCCAGCACAGUGGGAUGAUCGUUAAUGACACAGGACAUGAAACUGAUGAGAAUAGAGCGAAGGUUGAGAUAACGCCCAAUUCACCAAGAGCUGAAGCCACCCUGGGGGGUUUUGGAAGCCUAGGACUUGAUUGUGAACCGAG
KU955589    AGAAUCUGGAGUACCGGAUAAUGCUGUCAGUUCAUGGCUCCCAGCACAGUGGGAUGAUCGUUAAUGACACAGGACAUGAAACUGAUGAGAAUAGAGCGAAGGUUGAGAUAACGCCCAAUUCACCAAGAGCCGAAGCCACCCUGGGGGGUUUUGGAAGCCUAGGACUUGAUUGUGAACCGAG
KU955590    AGAAUCUGGAGUACCGGAUAAUGCUGUCAGUUCAUGGCUCCCAGCACAGUGGGAUGAUCGUUAAUGACACAGGACAUGAAACUGAUGAGAAUAGAGCGAAAGUUGAGAUAACGCCCAAUUCACCAAGAGCCGAAGCCACCCUGGGGGGUUUUGGAAGCCUAGGACUUGAUUGUGAACCGAG
KU940228    AGAAUCUGGAGUACCGGAUAAUGCUGUCAGUUCAUGGCUCCCAGCACAGUGGGAUGAUCGUUAAUGACACAGGACAUGAAACUGAUGAGAAUAGAGCGAAGGUUGAGAUAACGCCCAAUUCACCAAGAGCCGAAGCCACCCUGGGGGGUUUUGGAAGCCUAGGACUUGAUUGUGAACCGAG
KU991811    AGAAUCUGGAGUACCGGAUAAUGCUGUCAGUUCAUGGCUCCCAGCACAGUGGGAUGAUCGUUAAUGACACAGGACAUGAAACUGAUGAGAAUAGAGCGAAGGUUGAGAUAACGCCCAAUUCACCAAGAGCCGAAGCCACCCUGGGGGGUUUUGGAAGCCUAGGACUUGAUUGUGAACCGAG
KU509998    AGAAUCUGGAGUACCGGAUAAUGCUGUCAGUUCAUGGCUCCCAGCACAGUGGGAUGAUCGUUAAUGACACAGGACAUGAAACUGAUGAGAAUAGAGCGAAGGUUGAGAUAACGCCCAAUUCACCAAGAGCCGAAGCCACCCUGGGGGGUUUUGGAAGCCUAGGACUUGAUUGUGAACCGAG
KX056898    AGAAUCUGGAGUACCGGAUAAUGCUGUCAGUUCAUGGCUCCCAGCACAGUGGGAUGAUCGUUAAUGACACAGGACAUGAAACUGAUGAGAAUAGAGCGAAAGUUGAGAUAACGCCCAAUUCACCAAGAGCCGAAGCCACCCUGGGGGGUUUUGGAAGCCUAGGACUUGAUUGUGAACCGAG
KX117076    AGAAUCUGGAGUACCGGAUAAUGCUGUCAGUUCAUGGCUCCCAGCACAGUGGGAUGAUCGUUAAUGACACAGGACAUGAAACUGAUGAGAAUAGAGCGAAGGUUGAGAUAACGCCCAAUUCACCAAGAGCCGAAGCCACCCUGGGGGGUUUUGGAAGCCUAGGACUUGAUUGUGAACCGAG
KX185891    AGAAUCUGGAGUACCGGAUAAUGCUGUCAGUUCAUGGCUCCCAGCACAGUGGGAUGAUCGUUAAUGACACAGGACAUGAAACUGAUGAGAAUAGAGCGAAGGUUGAGAUAACGCCCAAUUCACCAAGAGCCGAAGCCACCCUGGGGGGUUUUGGAAGCCUAGGACUUGAUUGUGAACCGAG
KX247646    AGAAUCUGGAGUACCGGAUAAUGUUGUCAGUUCAUGGCUCCCAGCACAGUGGGAUGAUCGUUAAUGACACAGGACAUGAAACUGAUGAGAAUAGAGCGAAGGUUGAGAUAACGCCCAAUUCACCAAGAGCCGAAGCCACCCUGGGGGGUUUUGGAAGCCUAGGACUUGAUUGUGAACCGAG
KX262887    AGAAUCUGGAGUACCGGAUAAUGCUGUCAGUUCAUGGCUCCCAGCACAGUGGGAUGAUCGUUAAUGACACAGGACAUGAAACUGAUGAGAAUAGAGCGAAGGUUGAGAUAACGCCCAAUUCACCAAGAGCCGAAGCCACCCUGGGGGGUUUUGGAAGCCUAGGACUUGAUUGUGAACCGAG
KX280026    AGAAUCUGGAGUACCGGAUAAUGCUGUCAGUUCAUGGCUCCCAGCACAGUGGGAUGAUCGUUAAUGACACAGGACAUGAAACUGAUGAGAAUAGAGCGAAGGUUGAGAUAACGCCCAAUUCACCAAGAGCCGAAGCCACCCUGGGGGGUUUUGGAAGCCUAGGACUUGAUUGUGAACCGAG
KU758877    AGAAUCUGGAGUACCGGAUAAUGCUGUCAGUUCAUGGCUCCCAGCACAGUGGGAUGAUCGUUAAUGACACAGGACAUGAAACUGAUGAGAAUAGAGCGAAAGUUGAGAUAACGCCCAACUCACCAAGAGCCGAAGCCACCCUGGGGGGUUUUGGAAGCCUAGGACUUGAUUGUGAACCGAG
KU866423    AGAAUCUGGAGUACCGGAUAAUGCUGUCAGUUCAUGGCUCCCAGCACAGUGGGAUGAUCGUUAAUGACACAGGACAUGAAACUGAUGAGAAUAGAGCGAAGGUUGAGAUAACGCCCAAUUCACCAAGAGCCGAAGCCACCCUGGGGGGUUUUGGAAGCCUAGGACUUGAUUGUGAACCGAG
KX377336    AGAACCUGGAGUACCGGAUAAUGCUGUCAGUUCAUGGCUCCCAGCACAGUGGGAUGAUUGUUAAUGACAUAGGACAUGAAACUGAUGAGAAUAGAGCGAAGGUUGAGAUAACGCCCAAUUCACCAAGAGCCGAAGCCACCCUGGGAGGUUUUGGAAGCCUAGGACUUGAUUGUGAACCGAG
KX377337    AGAAUCUGGAGUACCGGAUAAUGCUGUCAGUUCAUGGCUCCCAGCACAGUGGGAUGAUCGUUAAUGACACAGGACAUGAAACUGAUGAGAAUAGAGCGAAAGUUGAGAUAACGCCCAAUUCACCGAGAGCCGAAGCCACCCUGGGGGGUUUUGGAAGCCUAGGACUUGAUUGUGAACCGAG
KX369547    AGAAUCUGGAGUACCGGAUAAUGCUGUCAGUUCAUGGCUCCCAGCACAGUGGGAUGAUCGUUAAUGACACAGGACAUGAAACUGAUGAGAAUAGAGCGAAGGUUGAGAUAACGCCCAAUUCACCAAGAGCCGAAGCCACCCUGGGGGGUUUUGGAAGCCUAGGACUUGAUUGUGAACCGAG
KX548902    AGAAUCUGGAGUACCGGAUAAUGUUGUCAGUUCAUGGCUCCCAGCACAGUGGGAUGAUCGUUAAUGACACAGGACAUGAAACUGAUGAGAAUAGAGCGAAGGUUGAGAUAACGCCCAAUUCACCAAGAGCCGAAGCCACCCUGGGGGGUUUUGGAAGCCUAGGACUUGAUUGUGAACCGAG
KX601166    AGAACUUGGAGUACCGGAUAAUGCUAUCAGUGCAUGGAUCCCAGCACAGUGGGAUGAUUGUGAAU------------------GACGAAAACAGAGCAAAAGUCGAGGUCACACCCAAUUCACCAAGAGCAGAAGCAACCUUGGGAGGUUUUGGAAGCUUGGGACUUGACUGUGAACCAAG
KX601167    AGAACCUGGAGUACCGGAUAAUGCUGUCAGUUCAUGGCUCCCAGCACAGUGGGAUGAUUGUUAAUGACACAGGACAUGAAACUGAUGAGAAUAGAGCGAAGGUUGAGAUAACGCCCAAUUCACCAAGAGCCGAAGCCACCCUGGGAGGUUUUGGAAGCCUAGGACUUGAUUGUGAACCGAG
KX601168    AGAAUCUGGAGUACCGGAUAAUGCUGUCAGUUCAUGGCUCCCAGCACAGUGGGAUGAUCGUUAAUGACACAGGACAUGAAACUGAUGAGAAUAGAGCGAAAGUUGAGAUAACGCCCAAUUCACCGAGAGCCGAAGCCACCCUGGGGGGUUUUGGAAGCCUAGGACUUGAUUGUGAACCGAG
KX766028    AGAAUCUGGAGUACCGGAUAAUGCUGUCAGUUCAUGGCUCCCAGCACAGUGGGAUGAUCGUUAAUGACACAGGACAUGAAACUGAUGAGAAUAGAGCGAARGUUGAGAUAACGCCCAAUUCACCAAGAGCCGAAGCCACCCUGGGGGGUUUUGGAAGCCUAGGACUUGAUUGUGAACCGAG
KX766029    AGAAUCUGGAGUACCGGAUAAUGCUGUCAGUUCAUGGCUCCCAGCACAGUGGGAUGAUCGUUAAUGACACAGGACAUGAAACUGAUGAGAAUAGAGCGAAGGUUGAGAUAACGCCCAAUUCACCAAGAGCCGAAGCCACCCUGGGGGGUUUUGGAAGCCUAGGACUUGAUUGUGAACCGAG
KU940224    AGAAUCUGGAGUACCGGAUAAUGCUGUCAGUUCAUGGCUCCCAGCACAGUGGGAUGAUCGUUAAUGACACAGGACAUGAAACUGAUGAGAAUAGAGCGAAGGUUGAGAUAACGCCCAAUUCACCAAGAGCCGAAGCCACCCUGGGGGGUUUUGGAAGCCUAGGACUUGAUUGUGAACCGAG


            1111111111111111111111111111111111111111111111111111111111111111111111111111111111111111111111111111111111111111111111111111111111111111111111111111111111111111111111111111111111111
            4444444444444444444444444444444444444444444444444445555555555555555555555555555555555555555555555555555555555555555555555555555555555555555555555555555555556666666666666666666666666666666
            4555555555666666666677777777778888888888999999999900000000001111111111222222222233333333334444444444555555555566666666667777777777888888888899999999990000000000111111111122222222222
Sequence    9012345678901234567890123456789012345678901234567890123456789012345678901234567890123456789012345678901234567890123456789012345678901234567890123456789012345678901234567890123456789
-------------------------------------------------------------------------------------------------------------------------------------------------------------------------------------------------
NC_012532   GACAGGCCUUGACUUUUCAGAUCUGUAUUACCUGACCAUGAACAAUAAGCAUUGGUUGGUGCACAAAGAGUGGUUUCAUGACAUCCCAUUGCCUUGGCAUGCUGGGGCAGACACCGGAACUCCACACUGGAACAACAAAGAGGCAUUGGUAGAAUUCAAGGAUGCCCACGCCAAGAGGCAA
KU922960    GACAGGCCUUGACUUUUCAGAUUUGUAUUACUUGACUAUGAAUAACAAGCACUGGUUGGUUCACAAGGAGUGGUUCCACGACAUUCCAUUACCUUGGCACGCUGGGGCAGACACCGGAACUCCACACUGGAACAACAAAGAAGCACUGGUAGAGUUCAAGGACGCACAUGCCAAAAGGCAA
KU922923    GACAGGCCUUGACUUUUCAGAUUUGUAUUACUUGACUAUGAAUAACAAGCACUGGUUGGUUCACAAGGAGUGGUUCCACGACAUUCCAUUACCUUGGCACGCUGGGGCAGACACCGGAACUCCACACUGGAACAACAAAGAAGCACUGGUAGAGUUCAAGGACGCACAUGCCAAAAGGCAA
KU820898    GACAGGCCUUGACUUUUCAGAUUUGUAUUACUUGACUAUGAAUAACAAGCACUGGUUGGUUCACAAGGAGUGGUUCCACGACAUUCCAUUACCUUGGCACGCUGGGGCAGACACCGGAACUCCACACUGGAACAACAAAGAAGCACUGGUAGAGUUCAAGGACGCACAUGCCAAAAGGCAA
KU321639    GACAGGCCUUGACUUUUCAGAUUUGUAUUACUUGACUAUGAAUAACAAGCACUGGUUGGUUCACAAGGAGUGGUUCCACGACAUUCCAUUACCUUGGCACGCUGGGGCAGACACCGGAACUCCACACUGGAACAACAAAGAAGCACUGGUAGAGUUCAAGGACGCACAUGCCAAAAGGCAA
KU820899    GACAGGCCUUGACUUUUCAGAUUUGUAUUACUUGACUAUGAAUAACAAGCACUGGUUGGUUCACAAGGAGUGGUUCCACGACAUUCCAUUACCUUGGCACGCUGGGGCAGACACCGGAACUCCACACUGGAACAACAAAGAAGCACUGGUAGAGUUCAAGGACGCACAUGCCAAAAGGCAA
KU853012    GACAGGCCUUGACUUUUCAGAUUUGUAUUACUUGACUAUGAAUAACAAGCACUGGUUGGUUCACAAGGAGUGGUUCCACGACAUUCCAUUACCUUGGCACGCUGGGGCAGACACCGGAACUCCACACUGGAACAACAAAGAAGCACUGGUAGAGUUCAAGGACGCACAUGCCAAAAGGCAA
KU729217    GACAGGCCUUGACUUUUCAGAUUUGUAUUACUUGACUAUGAAUAACAAGCACUGGUUGGUUCACAAGGAGUGGUUCCACGACAUUCCAUUACCUUGGCACGCUGGGGCAGACACCGGAACUCCACACUGGAACAACAAAGAAGCACUGGUAGAGUUCAAGGACGCACAUGCCAAAAGGCAA
KU729218    GACAGGCCUUGACUUUUCAGAUUUGUAUUACUUGACUAUGAAUAACAAGCACUGGUUGGUUCACAAGGAGUGGUUCCACGACAUUCCAUUACCUUGGCACGCUGGGGCAGACACCGGAACUCCACACUGGAACAAUAAAGAAGCACUGGUAGAGUUCAAGGACGCACAUGCCAAAAGGCAA
KU761564    GACAGGCCUUGACUUUUCAGAUUUGUAUUACUUGACUAUGAAUAACAAGCACUGGUUGGUUCACAAGGAGUGGUUCCACGACAUUCCAUUACCUUGGCACGCUGGGGCAGACACCGGAACUCCACACUGGAACAACAAAGAAGCACUGGUAGAGUUCAAGGACGCACAUGCCAAAAGGCAA
KU740184    GACAGGCCUUGACUUUUCAGAUUUGUAUUACUUGACUAUGAAUAACAAGCACUGGUUGGUUCACAAGGAGUGGUUCCACGACAUUCCAUUACCUUGGCACGCUGGGGCAGACACCGGAACUCCACACUGGAACAACAAAGAAGCACUGGUAGAGUUCAAGGACGCACAUGCCAAAAGGCAA
KU744693    GACAGGCCUUGACUUUUCAGAUUUGUAUUACUUGACUAUGAAUAACAAGCACUGGUUGGCUCACAAGGAGUGGUUCCACGACAUUCCAUUACCUUGGCACGCUGGGGCAGCCACCGGAACUCCACACUGGAACAACAAAGAAGCACUGGUAGAGUUCAAGGACGCACAUGCCAAAAGGCAA
KU497555    GACAGGCCUUGACUUUUCAGAUUUGUAUUACUUGACUAUGAAUAACAAGCACUGGUUGGUUCACAAGGAGUGGUUCCACGACAUUCCAUUACCUUGGCACGCUGGGGCAGACACCGGAACUCCACACUGGAACAACAAAGAAGCACUGGUAGAGUUCAAGGACGCACAUGCCAAAAGGCAA
KU527068    GACAGGCCUUGACUUUUCAGAUUUGUAUUACUUGACUAUGAAUAACAAGCACUGGUUGGUCCACAAGGAGUGGUUCCACGACAUUCCAUUACCUUGGCACGCUGGGGCAGACACCGGAACUCCACACUGGAACAACAAAGAAGCACUGGUAGAGUUCAAGGACGCACAUGCCAAAAGGCAA
KU501217    GACAGGCCUUGACUUUUCAGAUUUGUAUUACUUGACUAUGAAUAACAAGCACUGGUUGGUUCACAAGGAGUGGUUCCACGACAUUCCAUUACCUUGGCACGCUGGGGCAGACACCGGAACUCCACACUGGAACAACAAAGAAGCACUGGUGGAGUUCAAGGACGCACAUGCCAAAAGGCAA
KU501216    GACAGGCCUUGACUUUUCAGAUUUGUAUUACUUGACUAUGAAUAACAAGCACUGGUUGGUUCACAAGGAGUGGUUCCACGACAUUCCAUUACCUUGGCACGCUGGGGCAGACACCGGAACUCCACACUGGAACAACAAAGAAGCACUGGUGGAGUUCAAGGACGCACAUGCCAAAAGGCAA
KU501215    GACAGGCCUUGACUUUUCAGAUUUGUAUUACUUGACUAUGAAUAACAAGCACUGGUUGGUUCACAAGGAGUGGUUCCACGACAUUCCAUUACCUUGGCACGCUGGGGCAGACACCGGAACUCCACACUGGAACAACAAAGAAGCACUGGUAGAGUUCAAGGACGCACAUGCCAAAAGGCAA
KU365780    GACAGGCCUUGACUUUUCAGAUUUGUAUUACUUGACUAUGAAUAACAAGCACUGGUUGGUUCACAAGGAGUGGUUCCACGACAUUCCAUUACCUUGGCACGCUGGGGCAGACACCGGAACUCCACACUGGAACAACAAAGAAGCACUGGUAGAGUUCAAGGACGCACAUGCCAAAAGGCAA
KU365779    GACAGGCCUUGACUUUUCAGAUUUGUAUUACUUGACUAUGAAUAACAAGCACUGGUUGGUUCACAAGGAGUGGUUCCACGACAUUCCAUUACCUUGGCACGCUGGGGCAGACACCGGAACUCCACACUGGAACAACAAAGAAGCACUGGUAGAGUUCAAGGACGCACAUGCCAAAAGGCAA
KU707826    GACAGGCCUUGACUUUUCAGAUUUGUAUUACUUGACUAUGAAUAACAAGCACUGGUUGGUUCACAAGGAGUGGUUCCACGACAUUCCAUUACCUUGGCACGCUGGGGCAGACACCGGAACUCCACACUGGAACAACAAAGAAGCACUGGUAGAGUUCAAGGACGCACAUGCCAAAAGGCAA
KU365778    GACAGGCCUUGACUUUUCAGAUUUGUAUUACUUGACUAUGAAUAACAAGCACUGGUUGGUUCACAAGGAGUGGUUCCACGACAUUCCAUUACCUUGGCACGCUGGGGCAGACACCGGAACUCCACACUGGAACAACAAAGAAGCACUGGUAGAGUUCAAGGACGCACAUGCCAAAAGGCAA
KU365777    GACAGGCCUUGACUUUUCAGAUUUGUAUUACUUGACUAUGAAUAACAAGCACUGGUUGGUUCACAAGGAGUGGUUCCACGACAUUCCAUUACCUUGGCACGCUGGGGCAGACACCGGAACUCCACACUGGAACAACAAAGAAGCACUGGUAGAGUUCAAGGACGCACAUGCCAAAAGGCAA
KU312312    GACAGGCCUUGACUUUUCAGAUUUGUAUUACUUGACUAUGAAUAACAAGCACUGGCUGGUUCACAAGGAGUGGUUCCACGACAUUCCAUUACCUUGGCACGCUGGGGCAGACACCGGAACUCCACACUGGAACAACAAAGAAGCACUGGUAGAGUUCAAGGACGCACAUGCCAAAAGGCAA
KF268950    GACAGGCCUGGACUUUUCAGAUUUGUACUACUUGACCAUGAACAACAAGCACUGGUUGGUGCACAAAGAGUGGUUUCAUGACAUCCCAUUGCCUUGGCAUGCUGGGGCAGAUACCGGAACUCCACACUGGAACAACAAGGAGGCAUUGGUAGAGUUCAAAGAUGCCCACGCCAAGAGGCAA
KF268949    GACAGGCCUGGACUUUUCAGAUUUGUACUACUUGACCAUGAACAAUAAGCACUGGUUAGUGCACAAAGAGUGGUUUCAUGACAUCCCAUUGCCCUGGCAUGCUGGGGCAGACACCGGAACUCCACAUUGGAACAACAAGGAGGCAUUGGUAGAGUUCAAAGAUGCCCACGCUAAGAGGCAA
KF268948    GACAGGCCUGGACUUUUCAGAUUUGUACUACUUGACCAUGAACAACAAGCACUGGUUGGUGCACAAAGAGUGGUUUCAUGACAUCCCAUUGCCUUGGCAUGCUGGGGCAGAUACCGGAACUCCACACUGGAACAACAAGGAGGCAUUGGUAGAGUUCAAAGAUGCCCACGCCAAGAGGCAA
KJ776791    GACAGGCCUUGACUUUUCAGAUUUGUAUUACUUGACUAUGAAUAACAAGCACUGGUUGGUUCACAAGGAGUGGUUCCACGACAUUCCAUUACCUUGGCACGCUGGGGCAGACACCGGAACUCCACACUGGAACAACAAAGAAGCACUGGUAGAGUUCAAGGACGCACAUGCCAAAAGGCAA
KF383119    GACAGGCCUUGACUUUUCAGAUCUGUAUUACCUGACCAUGAACAAUAAGCAUUGGUUGGUGCACAAAGAGUGGUUUCAUGACAUCCCAUUGCCUUGGCAUGCUGGGGCAGACACCGGAACUCCACACUGGAACAACAAAGAGGCAUUGGUAGAAUUCAAGGAUGCCCACGCCAAGAGGCAA
KF383118    GACAGGCCUUGACUUCUCAGAUCUGUAUUAUCUGACCAUGAACAACAAGCAUUGGUUGGUGCACAAGGAGUGGUUUCAUGACAUCCCAUUACCUUGGCAUGCUGGUGCAGACACUGGAACUCCACACUGGAACAACAAAGAGGCAUUGGUGGAGUUCAAGGACGCCCACGCCAAGAGGCAA
KF383117    GACAGGCCUUGACUUCUCAGAUCUGUAUUAUCUGACCAUGAACAACAAGCAUCGGUUGGUGCGCAAGGAGUGGUUUCAUGACAUCCCAUUACCUUGGCAUGCUGGUGCAGACACUGGAACUCCACACUGGAACAACAAAGAGGCAUUGGUGGAGUUCAAGGACGCUCACGCCAAGAGGCAA
KF383116    GACAGGCCUUGACUUCUCAGAUCUGUAUUACCUGACCAUGAACAAUAAGCAUUGGUUGGUGCACAAGGAGUGGUUUCAUGACAUCCCAUUACCUUGGCAUGCUGGUGCAGACACUGGAACUCCACACUGGAACAACAAAGAGGCAUUGGUGGAGUUUAAGGACGCCCACGCCAAGAGGCAA
KF383115    GACAGGCCUUGACUUUUCAGAUUUGUAUUACCUGACCAUGAACAAUAAGCACUGGUUAGUCCACAAAGAGUGGUUUCAUGACAUCCCAUUGCCUUGGCAUGCUGGGGCAGACACCGGAACUCCACACUGGAACAACAAAGAGGCAUUGGUAGAGUUCAAGGAUGCCCACGCCAAGAGGCAA
EU545988    GACAGGCCUUGACUUUUCAGAUUUGUAUUACUUGACUAUGAAUAACAAGCACUGGUUGGUUCACAAGGAGUGGUUCCACGACAUUCCAUUACCUUGGCAUGCUGGGGCAGACACCGGAACUCCACAUUGGAACAACAAAGAAGCAUUGGUAGAGUUCAAGGACGCACAUGCCAAAAGGCAA
KU955589    GACAGGCCUUGACUUUUCAGAUUUGUAUUACUUGACUAUGAAUAACAAGCACUGGUUGGUUCACAAGGAGUGGUUCCACGACAUUCCAUUACCUUGGCACGCUGGGGCAGACACCGGAACUCCACACUGGAACAACAAAGAAGCACUGGUAGAGUUCAAGGACGCACAUGCCAAAAGGCAA
KU955590    GACAGGCCUUGACUUUUCAGAUUUGUAUUACUUGACUAUGAAUAACAAGCACUGGUUGGUUCACAAGGAGUGGUUCCACGACAUUCCAUUACCUUGGCACGCAGGGGCAGACACCGGAACUCCACACUGGAACAACAAAGAAGCACUGGUAGAGUUCAAGGACGCACAUGCCAAAAGGCAA
KU940228    GACAGGCCUUGACUUUUCAGAUUUGUAUUACUUGACUAUGAAUAACAAGCACUGGUUGGUUCACAAGGAGUGGUUCCACGACAUUCCAUUACCUUGGCACGCUGGGGCAGACACCGGAACUCCACACUGGAACAACAAAGAAGCACUGGUAGAGUUCAAGGACGCACAUGCCAAAAGGCAA
KU991811    GACAGGCCUUGACUUUUCAGAUUUGUAUUACUUGACUAUGAAUAACAAGCACUGGUUGGUUCACAAGGAGUGGUUCCACGACAUUCCAUUACCUUGGCACGCUGGGGCAGACACCGGAACUCCACACUGGAACAACAAAGAAGCACUGGUAGAGUUCAAGGACGCACAUGCCAAAAGGCAA
KU509998    GACAGGCCUUGACUUUUCAGAUUUGUAUUACUUGACUAUGAAUAACAAGCACUGGUUGGUUCACAAGGAGUGGUUCCACGACAUUCCAUUACCUUGGCACGCUGGGGCAGACACCGGAACUCCACACUGGAACAACAAAGAAGCACUGGUAGAGUUCAAGGACGCACAUGCCAAAAGGCAA
```

```
KX056898    GACAGGCCUUGACUUUUCAGAUUUGUAUUACUUGACUAUGAAUAACAAGCACUGGUUGGUUCACAAGGAGUGGUUCCACGACAUUCCAUUACCUUGGCACGCAGGGGCAGACACCGGAACUCCACACUGGAACAACAAAGAAGCACUGGUAGAGUUCAAGGACGCACAUGCCAAAAGGCAA
KX117076    GACAGGCCUUGACUUUUCAGAUUUGUAUUACUUGACUAUGAAUAACAAGCACUGGUUGGUUCACAAGGAGUGGUUCCACGACAUUCCAUUACCUUGGCACGCUGGGGCAGACACCGGAACUCCACACUGGAACAACAAAGAAGCACUGGUAGAGUUCAAGGACGCACAUGCCAAAAGGCAA
KX185891    GACAGGCCUUGACUUUUCAGAUUUGUAUUACUUGACUAUGAAUAACAAGCACUGGUUGGUUCACAAGGAGUGGUUCCACGACAUUCCAUUACCUUGGCACGCUGGGGCAGACACCGGAACUCCACACUGGAACAACAAAGAAGCACUGGUAGAGUUCAAGGACGCACAUGCCAAAAGGCAA
KX247646    GACAGGCCUUGACUUUUCAGAUUUGUAUUACUUGACUAUGAAUAACAAGCACUGGUUGGUUCACAAGGAGUGGUUCCACGACAUUCCAUUACCUUGGCACGCUGGGGCAGACACCGGAACUCCACACUGGAACAACAAAGAAGCACUGGUAGAGUUCAAGGACGCACAUGCCAAAAGGCAA
KX262887    GACAGGCCUUGACUUUUCAGAUUUGUAUUACUUGACUAUGAAUAACAAGCACUGGUUGGUUCACAAGGAGUGGUUCCACGACAUUCCAUUACCUUGGCACGCUGGGGCAGACACCGGAACUCCACACUGGAACAACAAAGAAGCACUGGUAGAGUUCAAGGACGCACAUGCCAAAAGGCAA
KX280026    GACAGGCCUUGACUUUUCAGAUUUGUAUUACUUGACUAUGAAUAACAAGCACUGGUUGGUUCACAAGGAGUGGUUCCACGACAUUCCAUUACCUUGGCACGCUGGGGCAGACACCGGAACUCCACACUGGAACAACAAAGAAGCACUGGUAGAGUUCAAGGACGCACAUGCCAAAAGGCAA
KU758877    GACAGGCCUUGACUUUUCAGAUUUGUAUUACUUGACUAUGAAUAACAAGCACUGGUUGGUUCACAAGGAGUGGUUCCACGACAUUCCAUUACCUUGGCACGCUGGGGCAGACACCGGAACUCCACACUGGAACAACAAAGAAGCACUGGUAGAGUUCAAGGACGCACAUGCCAAAAGGCAA
KU866423    GACAGGCCUUGACUUUUCAGAUUUGUAUUACUUGACUAUGAAUAACAAGCACUGGUUGGUUCACAAGGAGUGGUUCCACGACAUUCCAUUACCUUGGCACGCUGGGGCAGACACCGGAACUCCACACUGGAACAACAAAGAAGCACUGGUAGAGUUCAAGGACGCACAUGCCAAAAGGCAA
KX377336    GACAGGCCUUGACUUUUCAGAUUUGUAUUACUUGACUAUGAAUAACAAGCAUUGGUUGGUGCACAAGGAGUGGUUCCAUGACAUUCCACUACCUUGGCAUGCUGGGGCAGACACCGGAACUCCACAUUGGAACAACAAAGAAGCAUUGGUAGAGUUCAAGGACGCACAUGCCAAAAGGCAA
KX377337    GACAGGCCUUGACUUUUCAGAUUUGUAUUACUUGACUAUGAAUAACAAGCACUGGUUGGUUCACAAGGAGUGGUUCCACGACAUUCCAUUACCUUGGCACGCUGGGGCAGACACCGGAACUCCACACUGGAACAACAAAGAAGCACUGGUAGAGUUCAAGGACGCACAUGCCAAAAGGCAA
KX369547    GACAGGCCUUGACUUUUCAGAUUUGUAUUACUUGACUAUGAAUAACAAGCACUGGUUGGUUCACAAGGAGUGGUUCCACGACAUUCCAUUACCUUGGCACGCUGGGGCAGACACCGGAACUCCACACUGGAACAACAAAGAAGCACUGGUAGAGUUCAAGGACGCACAUGCCAAAAGGCAA
KX548902    GACAGGCCUUGACUUUUCAGAUUUGUAUUACUUGACUAUGAAUAACAAGCACUGGUUGGUUCACAAGGAGUGGUUCCACGACAUUCCAUUACCUUGGCACGCUGGGGCAGACACCGGAACUCCACACUGGAACAACAAAGAAGCACUGGUAGAGUUCAAGGACGCACAUGCCAAAAGGCAA
KX601166    GACAGGCCUUGACUUCUCAGAUCUGUAUUACCUGACCAUGAACAAUAAGCAUUGGUUGGUGCACAAGGAGUGGUUUCAUGACAUCCCAUUACCUUGGCAUGCUGGUGCAGACACUGGACUCCACACUGGAACAACAAAGAGGCAUUGGUGGAGUUCAAGGACGCCCACGCCAAGAGGCAA
KX601167    GACAGGCCUUGACUUUUCAGAUUUGUAUUACUUGACUAUGAAUAACAAGCAUUGGUUGGUGCACAAGGAGUGGUUCCAUGACAUUCCACUACCUUGGCAUGCUGGGGCAGACACCGGAACUCCACAUUGGAACAACAAAGAAGCAUUGGUAGAGUUCAAGGACGCACAUGCCAAAAGGCAA
KX601168    GACAGGCCUUGACUUUUCAGAUUUGUAUUACUUGACUAUGAAUAACAAGCACUGGUUGGUUCACAAGGAGUGGUUCCACGACAUUCCAUUACCUUGGCACGCUGGGGCAGACACCGGAACUCCACACUGGAACAACAAAGAAGCACUGGUAGAGUUCAAGGACGCACAUGCCAAAAGGCAA
KX766028    GACAGGCCUUGACUUUUCAGAUUUGUAUUACUUGACUAUGAAUAACAAGCACUGGUUGGUUCACAAGGAGUGGUUCCACGACAUUCCAUUACCUUGGCACGCUGGGGCAGACACCGGAACUCCACACUGGAACAACAAAGAAGCACUGGUAGAGUUCAAGGACGCACAUGCCAAAAGGCAA
KX766029    GACAGGCCUUGACUUUUCAGAUUUGUAUUACUUGACUAUGAAUAACAAGCACUGGUUGGUUCACAAGGAGUGGUUCCACGACAUUCCAUUACCUUGGCACGCUGGGGCAGACACCGGAACUCCACACUGGAACAACAAAGAAGCACUGGUAGAGUUCAAGGACGCACAUGCCAAAAGGCAA
KU940224    GACAGGCCUUGACUUUUCAGAUUUGUAUUACUUGA??AUGAAUAACAAGCACUGGUUGGUUCACAAGGAGUGGUUCCACGACAUUCCAUUACCUUGGCACGCUGGGGCAGACACCGGAACUCCACACUGGAACAACAAAGAAGCACUGGUAGAGUUCAAGGACGCACAUGCCAAAAGGCAA


            1111111111111111111111111111111111111111111111111111111111111111111111111111111111111111111111111111111111111111111111111111111111111111111111111111111111111111111111111111111111111
            6666666666666666666666666666666666666666666666666666666666666666666666777777777777777777777777777777777777777777777777777777777777777777777777777777777777777777777777788888888888
            3333333333444444444455555555556666666666777777777788888888889999999999000000000011111111112222222222333333333344444444445555555555666666666677777777778888888888999999999900000000001
Sequence    0123456789012345678901234567890123456789012345678901234567890123456789012345678901234567890123456789012345678901234567890123456789012345678901234567890123456789012345678901234567890
---------------------------------------------------------------------------------------------------------------------------------------------------------------------------------------------------------
NC_012532   ACCGUCGUCGUUCUGGGGAGCCAGGAAGGAGCCGUUCACACGGCUCUCGCUGGAGCUCUAGAGGCUGAGAUGGAUGGUGCAAAGGGAAGGCUGUUCUCUGGCCAUUUGAAAUGCCGCCUAAAAAUGGACAAGCUUAGAUUGAAGGGCGUGUCAUAUUCCUUGUGCACUGCGGCAUUCACAU
KU922960    ACUGUCGUGGUUCUAGGGAGUCAAGAAGGAGCAGUUCACACGGCCCUUGCUGGAGCUCUGGAGGCUGAGAUGGAUGGUGCAAAGGGAAGGCUGUCCUCUGGCCACUUGAAAUGUCGCCUGAAAAUGGAUAAACUUAGAUUGAAGGGCGUGUCAUACUCCUUGUGUACCGCAGCGUUCACAU
KU922923    ACUGUCGUGGUUCUAGGGAGUCAAGAAGGAGCAGUUCACACGGCCCUUGCUGGAGCUCUGGAGGCUGAGAUGGAUGGUGCAAAGGGAAGGCUGUCCUCUGGCCACUUGAAAUGUCGCCUGAAAAUGGAUAAACUUAGAUUGAAGGGCGUGUCAUACUCCUUGUGUACCGCAGCGUUCACAU
KU820898    ACUGUCGUGGUUCUAGGGAGUCAAGAAGGAGCAGUUCACACGGCCCUUGCUGGAGCUCUGGAGGCUGAGAUGGAUGGAGCAAAGGGAAGGCUGUCCUCUGGCCACUUGAAAUGUCGCCUGAAAAUGGAUAAACUUAGAUUGAAGGGCGUGUCAUACUCCUUGUGUACUGCAGCGUUCACAU
KU321639    ACUGUCGUGGUUCUAGGGAGUCAAGAAGGAGCAGUUCACACGGCCCUUGCUGGAGCUCUGGAGGCUGAGAUGGAUGGUGCAAAGGGAAGGCUGUCCUCUGGCCACUUGAAAUGUCGCCUGAAAAUGGAUAAACUUAGAUUGAAGGGCGUGUCAUACUCCUUGUGUACCGCAGCGUUCACAU
KU820899    ACUGUCGUGGUUCUAGGGAGUCAAGAAGGAGCAGUUCACACGGCCCUUGCUGGAGCUCUGGAGGCUGAGAUGGAUGGUGCAAAGGGAAGGCUGUCCUCUGGCCACUUGAAAUGUCGCCUGAAAAUGGAUAAACUUAGAUUGAAGGGCGUGUCAUACUCCUUGUGUACCGCAGCGUUCACAU
KU853012    ACUGUCGUGGUUCUAGGGAGUCAAGAAGGAGCAGUUCACACGGCCCUUGCUGGAGCUCUGGAGGCUGAGAUGGAUGGUGCAAAGGGAAGGCUGUCCUCUGGCCACUUGAAAUGUCGCCUGAAAAUGGAUAAACUUAGAUUGAAGGGCGUGUCAUACUCCUUGUGUACCGCAGCGUUCACAU
KU729217    ACUGCCGUGGUUCUAGGGAGUCAAGAAGGAGCAGUUCACACGGCCCUUGCUGGAGCUCUGGAGGCUGAGAUGGAUGGUGCAAAGGGAAGGCUGUCCUCUGGCCACUUGAAAUGUCGCCUGAAAAUGGAUAAACUUAGAUUGAAGGGCGUGUCAUACUCCUUGUGUACCGCAGCGUUCACAU
KU729218    ACUGUCGUGGUUCUAGGGAGUCAAGAAGGAGCAGUUCACACGGCCCUUGCUGGAGCUCUGGAGGCUGAGAUGGAUGGUGCAAAGGGAAGGCUGUCCUCUGGCCACUUGAAAUGUCGCCUGAAAAUGGAUAAACUUAGAUUGAAGGGCGUGUCAUACUCCUUGUGUACCGCAGCGUUCACAU
KU761564    ACUGUCGUGGUUCUAGGGAGUCAAGAAGGAGCAGUUCACACGGCCCUUGCUGGAGCUCUGGAGGCUGAGAUGGAUGGAGCAAAGGGAAGGCUGUCCUCUGGCCACUUGAAAUGUCGCCUGAAAAUGGAUAAACUUAGAUUGAAGGGCGUGUCAUACUCCUUGUGUACUGCAGCGUUCACAU
KU740184    ACUGUCGUGGUUCUAGGGAGUCAAGAAGGAGCAGUUCACACGGCCCUUGCUGGAGCUCUGGAGGCUGAGAUGGAUGGAGCAAAGGGAAGGCUGUCCUCUGGCCACUUGAAAUGUCGCCUGAAAAUGGAUAAACUUAGAUUGAAGGGCGUGUCAUACUCCUUGUGUACUGCAGCGUUCACAU
KU744693    ACUGUCGUGGUUCUAGGGAGUCAAGAAGGAGCAGUUCACACGGCCCUUGCUGGAGCUCUGGAGGCUGAGAUGGAUGGUGCAAAGGGAAGGCUGUCCUCUGGCCACUUGAAAUGUCGCCUGAAAAUGGAUAAACUUAGAUUGAAGGGCGUGUCAUACUCCUUGUGUACCGCAGCGUUCACAU
KU497555    ACUGUCGUGGUUCUAGGGACUCAAGAAGGAGCAGUUCACACGGCCCUUGCUGGAGCUCUGGAGGCUGAGAUGGAUGGUGCAAAGGGAAGGCUGUCCUCUGGCCACUUGAAAUGUCGCCUGAAAAUGGAUAAACUUAGAUUGAAGGGCGUGUCAUACUCCUUGUGUACCGCAGCGUUCACAU
KU527068    ACUGUCGUGGUUCUAGGGAGUCAAGAAGGAGCAGUUCACACGGCCCUUGCUGGAGCUCUGGAGGCUGAGAUGGAUGGUGCAAAGGGAAGGCUGUCCUCUGGCCACUUGAAAUGUCGCCUGAAAAUGGAUAAACUUAGAUUGAAGGGCGUGUCAUACUCCUUGUGUACCGCAGCGUUCACAU
KU501217    ACUGUCGUGGUUCUAGGGAGUCAAGAAGGAGCAGUUCACACGGCCCUUGCUGGAGCUCUGGAGGCUGAGAUGGAUGGUGCAAAGGGAAGGCUGUCCUCUGGCCACUUGAAAUGUCGCCUGAAAAUGGAUAAACUUAGAUUGAAGGGCGUGUCAUACUCCUUGUGUACCGCAGCGUUCACAU
KU501216    ACUGUCGUGGUUCUAGGGAGUCAAGAAGGAGCAGUUCACACGGCCCUUGCUGGAGCUCUGGAGGCUGAGAUGGAUGGUGCAAAGGGAAGGCUGUCCUCUGGCCACUUGAAAUGUCGCCUGAAAAUGGAUAAACUUAGAUUGAAGGGCGUGUCAUACUCCUUGUGUACCGCAGCGUUCACAU
KU501215    ACUGUCGUGGUUCUAGGGAGUCAAGAAGGAGCAGUUCACACGGCCCUUGCUGGAGCUCUGGAGGCUGAGAUGGAUGGUGCAAAGGGAAGGCUGUCCUCUGGCCACUUGAAAUGUCGCCUGAAAAUGGAUAAACUUAGAUUGAAGGGCGUGUCAUACUCCUUGUGUACUGCAGCGUUCACAU
KU365780    ACUGUCGUGGUUCUAGGGAGUCAAGAAGGAGCAGUUCACACGGCCCUUGCUGGAGCUCUGGAGGCUGAGAUGGAUGGUGCAAAGGGAAGGCUGUCCUCUGGCCACUUGAAAUGUCGCCUGAAAAUGGAUAAACUUAGAUUGAAGGGCGUGUCAUACUCCUUGUGUACUGCAGCGUUCACAU
KU365779    ACUGUCGUGGUUCUAGGGAGUCAAGAAGGAGCAGUUCACACGGCCCUUGCUGGAGCUCUGGAGGCUGAGAUGGAUGGUGCAAAGGGAAGGCUGUCCUCUGGCCACUUGAAAUGUCGCCUGAAAAUGGAUAAACUUAGAUUGAAGGGCGUGUCAUACUCCUUGUGUACUGCAGCGUUCACAU
KU707826    ACUGUCGUGGUUCUAGGGAGUCAAGAAGGAGCAGUUCACACGGCCCUUGCUGGAGCUCUGGAGGCUGAGAUGGAUGGUGCAAAGGGAAGGCUGUCCUCUGGCCACUUGAAAUGUCGCCUGAAAAUGGAUAAACUUAGAUUGAAGGGCGUGUCAUACUCCUUGUGUACUGCAGCGUUCACAU
KU365778    ACUGUCGUGGUUCUAGGGAGUCAAGAAGGAGCAGUUCACACGGCCCUUGCUGGAGCUCUGGAGGCUGAGAUGGAUGGUGCAAAGGGAAGGCUGUCCUCUGGCCACUUGAAAUGUCGCCUGAAAAUGGAUAAACUUAGAUUGAAGGGCGUGUCAUACUCCUUGUGUACUGCAGCGUUCACAU
KU365777    ACUGUCGUGGUUCUAGGGAGUCAAGAAGGAGCAGUUCACACGGCCCUUGCUGGAGCUCUGGAGGCUGAGAUGGAUGGUGCAAAGGGAAGGCUGUCCUCUGGCCACUUGAAAUGUCGCCUGAAAAUGGAUAAACUUAGAUUGAAGGGCGUGUCAUACUCCUUGUGUACUGCAGCGUUCACAU
KU312312    ACUGUCGUGGUUCUAGGGAGUCAAGAAGGAGCAGUUCACACGGCCCUUGCUGGAGCUCUGGAGGCUGAGAUGGAUGGUGCAAAGGGAAGGCUGUCCUCUGGCCACUUGAAAUGUCGCCUGAAAAUGGAUAAACUUAGAUUGAAGGGCGUGUCAUACUCCUUGUGUACUGCAGCGUUCACAU
KF268950    ACCGUCGUUGUUCUAGGGAGCCAGGAAGGAGCCGUUCACACGGCUCUUGCUGGAGCUCUGGAGGCUGAGAUGGAUGGUGCAAAAGGAAGGCUGUUCUCCGGCCAUUUGAAAUGCCGCUUAAAAAUGGACAAGCUUAGAUUGAAGGGCGUGUCAUAUUCCUUGUGCACCGCAGCGUUCACAU
KF268949    ACCGUCGUUGUUCUAGGGAGCCAGGAAGGAGCCGUUCACACGGCUCUUGCUGGAGCUCUGGAGGCUGAGAUGGAUGGUGCAAAAGGAAGGUUGUUCUCCGGCCAUUUGAAAUGCCGCUUAAAAAUGGACAAGCUUAGAUUGAAGGGCGUGUCAUAUUCCUUGUGCACCGCAGCGUUCACAU
KF268948    ACCGUCGUUGUUCUAGGGAGCCAGGAAGGAGCCGUUCACACGGCUCUUGCUGGAGCUCUGGAGGCUGAGAUGGAUGGUGCAAAAGGAAGGCUGUUCUCCGGCCAUUUGAAAUGCCGCUUAAAAAUGGACAAGCUUAGAUUGAAGGGCGUGUCAUAUUCCUUGUGCACCGCAGCGUUCACAU
KJ776791    ACUGUCGUGGUUCUAGGGAGUCAAGAAGGAGCAGUUCACACGGCCCUUGCUGGAGCUCUGGAGGCUGAGAUGGAUGGUGCAAAGGGAAGGCUGUCCUCUGGCCACUUGAAAUGUCGCCUGAAAAUGGAUAAACUUAGAUUGAAGGGCGUGUCAUACUCCUUGUGUACCGCAGCGUUCACAU
KF383119    ACCGUCGUCGUUCUGGGGAGCCAGGAAGGAGCCGUUCACACGGCUCUCGCUGGAGCUCUAGAGGCUGAGAUGGAUGGUGCAAAGGGAAGGCUGUUCUCUGGCCAUUUGAAAUGCCGCCUAAAAAUGGACAAGCUUAGAUUGAAGGGCGUGUCAUAUUCCUUGUGCACUGCGGCAUUCACAU
KF383118    ACUGUUGUGGUUCUGGGGAGCCAAGAGGGAGCUGUUCACACGGCCCUCGCUGGAGCUCUGGAGGCUGAGAUGGAUGGUGCAAAGGGAAGGCUAUUCUCUGGCCAUUUGAAAUGCCGCCUAAAAAUGGACAAGCUUAGGUUGAAGGGUGUGUCAUAUUCCCUGUGUACUGCAGCGUUCACAU
KF383117    ACUGUUGUGGUUCUGGGGAGCCAAGAGGGAGCUGUUCACACGGCCCUCGCUGGAGCUCUGGAGGCUGAGAUGGAUGGUGCAAAGGGAAGGCUAUUCUCUGGCCAUUUGAAAUGCCGCCUAAAAAUGGACAAGCUUAGGUUGAAGGGUGUGUCAUAUUCCCUGUGUACUGCAGCGUUCACAU
KF383116    ACUGUUGUGGUUCUGGGGAGCCAAGAGGGAGCUGUUCACACGGCCCUCGCUGGAGCUUUGGAGGCUGAGAUGGAUGGUGCAAAGGGAAGGCUAUUCUCUGGCCAUUUGAAAUGCCGCCUAAAAAUGGACAAGCUUAGGUUGAAGGGUGUGUCAUAUUCCCUGUGUACCGCAGUGUGUACCG
KF383115    ACCGUCGUCGUUCUGGGGAGCCAGGAAGGAGCUGUUCACACGGCUCUUGCUGGAGCUUUGGAGGCUGAGAUGGAUGGUGCAAAAGGAAGGCUGUUCUCUGGCCAUUUGAAAUGCCGCCUAAAAAUGGACAAGCUUAGAUUGAAGGGCGUGUCAUAUUCCUUGUGCACCGCAGCGUUCACAU
EU545988    ACUGUCGUGGUUCUAGGGAGUCAAGAAGGAGCAGUUCACACGGCCCUUGCUGGAGCUCUGGAGGCUGAGAUGGAUGGUGCAAAGGGAAGGCUGUCCUCUGGCCACUUGAAAUGUCGCCUGAAAAUGGAUAAACUUAGAUUGAAGGGCGUGUCAUACUCCUUGUGUACCGCAGCGUUCACAU
KU955589    ACUGUCGUGGUUCUAGGGAGUCAAGAAGGAGCAGUUCACACGGCCCUUGCUGGAGCUCUGGAGGCUGAGAUGGAUGGUGCAAAGGGAAGGCUGUCCUCUGGCCACUUGAAAUGUCGCCUGAAAAUGGAUAAACUUAGAUUGAAGGGCGUGUCAUACUCCUUGUGUACCGCAGCGUUCACAU
KU955590    ACUGUCGUGGUUCUAGGGAGUCAAGAAGGAGCAGUUCACACGGCCCUUGCUGGAGCUCUGGAGGCUGAGAUGGAUGGAGCAAAGGGAAGGCUGUCCUCUGGCCACUUGAAAUGUCGCCUGAAAAUGGAUAAACUUAGAUUGAAGGGCGUGUCAUACUCCUUGUGUACUGCAGCGUUCACAU
KU940228    ACUGUCGUGGUUCUAGGGAGUCAAGAAGGAGCAGUUCACACGGCCCUUGCUGGAGCUCUAGAGGCUGAGAUGGAUGGUGCAAAGGGAAGGCUGUCCUCUGGCCACUUGAAAUGUCGCCUGAAAAUGGAUAAACUUAGAUUGAAGGGCGUGUCAUACUCCUUGUGUACCGCAGCGUUCACAU
KU991811    ACUGUCGUGGUUCUAGGGAGUCAAGAAGGAGCAGUUCACACGGCCCUUGCUGGAGCUCUGGAGGCUGAGAUGGAUGGUGCAAAGGGAAGGCUGUCCUCUGGCCACUUGAAAUGUCGCCUGAAAAUGGAUAAACUUAGAUUGAAGGGCGUGUCAUACUCCUUGUGUACCGCAGCGUUCACAU
KU509998    ACUGUCGUGGUUCUAGGGAGUCAAGAAGGAGCAGUUCACACGGCCCUUGCUGGAGCUCUGGAGGCUGAGAUGGAUGGUGCAAAGGGAAGGCUGUCCUCUGGCCACUUGAAAUGUCGCCUGAAAAUGGAUAAACUUAGAUUGAAGGGCGUGUCAUACUCCUUGUGUACCGCAGCGUUCACAU
KX056898    ACUGUCGUGGUUCUAGGGAGUCAAGAAGGAGCAGUUCACACGGCCCUUGCUGGAGCUCUGGAGGCUGAGAUGGAUGGAGCAAAGGGAAGGCUGUCCUCUGGCCACUUGAAAUGUCGCCUGAAAAUGGAUAAACUUAGAUUGAAGGGCGUGUCAUACUCCUUGUGUACUGCAGCGUUCACAU
KX117076    ACUGUCGUGGUUCUAGGGAGUCAAGAAGGAGCAGUUCACACGGCCCUUGCUGGAGCUCUGGAGGCUGAGAUGGAUGGUGCAAAGGGAAGGCUGUCCUCUGGCCACUUGAAAUGUCGCCUGAAAAUGGAUAAACUUAGAUUGAAGGGCGUGUCAUACUCCUUGUGUACCGCAGCGUUCACAU
KX185891    ACUGUCGUGGUUCUAGGGAGUCAAGAAGGAGCAGUUCACACGGCCCUUGCUGGAGCUCUGGAGGCUGAGAUGGAUGGUGCAAAGGGAAGGCUGUCCUCUGGCCACUUGAAAUGUCGCCUGAAAAUGGAUAAACUUAGAUUGAAGGGCGUGUCAUACUCCUUGUGUACCGCAGCGUUCACAU
KX247646    ACUGUCGUGGUUCUAGGGAGUCAAGAAGGAGCAGUUCACACGGCCCUUGCUGGAGCUCUGGAGGCUGAGAUGGAUGGUGCAAAGGGAAGGCUGUCCUCUGGCCACUUGAAAUGUCGCCUGAAAAUGGAUAAACUUAGAUUGAAGGGCGUGUCAUACUCCUUGUGUACCGCAGCGUUCACAU
KX262887    ACUGUCGUGGUUCUAGGGAGUCAAGAAGGAGCAGUUCACACGGCCCUUGCUGGAGCUCUGGAGGCUGAGAUGGAUGGUGCAAAGGGAAGGCUGUCCUCUGGCCACUUGAAAUGUCGCCUGAAAAUGGAUAAACUUAGAUUGAAGGGCGUGUCAUACUCCUUGUGUACCGCAGCGUUCACAU
KX280026    ACUGUCGUGGUUCUAGGGAGUCAAGAAGGAGCAGUUCACACGGCCCUUGCUGGAGCUCUGGAGGCUGAGAUGGAUGGUGCAAAGGGAAGGCUGUCCUCUGGCCACUUGAAAUGUCGCCUGAAAAUGGAUAAACUUAGAUUGAAGGGCGUGUCAUACUCCUUGUGUACCGCAGCGUUCACAU
KU758877    ACUGUCGUGGUUCUAGGGAGUCAAGAAGGAGCAGUUCACACGGCCCUUGCUGGAGCUCUGGAGGCUGAGAUGGAUGGUGCAAAGGGAAGGCUGUCCUCUGGCCACUUGAAAUGUCGCCUGAAAAUGGAUAAACUUAGAUUGAAGGGCGUGUCAUACUCCUUGUGUACUGCAGCGUUCACAU
KU866423    ACUGUCGUGGUUCUAGGGAGUCAAGAAGGAGCAGUUCACACGGCCCUUGCUGGAGCUCUGGAGGCUGAGAUGGAUGGUGCAAAGGGAAGGCUGUCCUCUGGCCACUUGAAAUGUCGCCUGAAAAUGGAUAAACUUAGAUUGAAGGGCGUGUCAUACUCCUUGUGUACCGCAGCGUUCACAU
KX377336    ACUGUCGUGGUUCUAGGGAGUCAAGAAGGAGCCGUUCACACGGCUCUUGCUGGAGCCCUGGAGGCUGAGAUGGAUGGUGCAAAGGGAAGGCUGUCCUCUGGCCACUUGAAAUGUCGCUUGAAAAUGGACAAACUUAGAUUGAAGGGCGUGUCAUACUCCUUAUGUACCGCGGCGUUCACAU
KX377337    ACUGUCGUGGUUCUAGGGAGUCAAGAAGGAGCAGUUCACACGGCCCUUGCUGGAGCUCUGGAGGCUGAGAUGGAUGGUGCAAAGGGAAGGCUGUCCUCUGGCCACUUGAAAUGUCGCCUGAAAAUGGAUAAACUUAGAUUGAAGGGCGUGUCAUACUCCUUGUGUACUGCAGCGUUCACAU
KX369547    ACUGUCGUGGUUCUAGGGAGUCAAGAAGGAGCAGUUCACACGGCCCUUGCUGGAGCUCUGGAGGCUGAGAUGGAUGGUGCAAAGGGAAGGCUGUCCUCUGGCCACUUGAAAUGUCGCCUGAAAAUGGAUAAACUUAGAUUGAAGGGCGUGUCAUACUCCUUGUGUACCGCAGCGUUCACAU
KX548902    ACUGUCGUGGUUCUAGGGAGUCAAGAAGGAGCAGUUCACACGGCCCUUGCUGGAGCUCUGGAGGCUGAGAUGGAUGGUGCAAAGGGAAGGCUGUCCUCUGGCCACUUGAAAUGUCGCCUGAAAAUGGAUAAACUUAGAUUGAAGGGCGUGUCAUACUCCUUGUGUACCGCAGCGUUCACAU
KX601166    ACUGUUGUGGUUCUGGGGAGCCAAGAGGGAGCUGUUCAUACGGCCCUCGCUGGAGCUUUGGAGGCUGAGAUGGAUGGUGCAAAGGGAAGGCUAUUCUCUGGCCAUUUGAAAUGCCGCCUAAAAAUGGACAAGCUUAGGUUGAAGGGUGUGUCAUAUUCCCUGUGUACCGCAGCGUUCACAU
KX601167    ACUGUCGUGGUUCUAGGGAGUCAAGAAGGAGCCGUUCACACGGCUCUUGCUGGAGCCCUGGAGGCUGAGAUGGAUGGUGCAAAGGGAAGGCUGUCCUCUGGCCACUUGAAAUGUCGCUUGAAAAUGGACAAACUUAGAUUGAAGGGCGUGUCAUACUCCUUAUGUACCGCGGCGUUCACAU
KX601168    ACUGUCGUGGUUCUAGGGAGUCAAGAAGGAGCAGUUCACACGGCCCUUGCUGGAGCUCUGGAGGCUGAGAUGGAUGGUGCAAAGGGAAGGCUGUCCUCUGGCCACUUGAAAUGUCGCCUGAAAAUGGAUAAACUUAGAUUGAAGGGCGUGUCAUACUCCUUGUGUACUGCAGCGUUCACAU
```

```
KX766028    ACUGUCGUGGUUCUAGGGAGUCAAGAAGGAGCAGUUCACACGGCCCUUGCUGGAGCUCUGGAGGCUGAGAUGGAUGGAGCAAAGGGAAGGCUGUCCUCUGGCCACUUGAAAUGUCGCCUGAAAAUGGAUAAACUUAGAUUGAAGGGCGUGUCAUACUCCUUGUGUACUGCAGCGUUCACAU
KX766029    ACUGUCGUGGUUCUAGGGAGUCAAGAAGGAGCAGUUCACACGGCCCUUGCUGGAGCUCUGGAGGCUGAGAUGGAUGGUGCAAAGGGAAGGCUGUCCUCUGGCCACUUGAAAUGUCGCCUGAAAAUGGAUAAACUUAGAUUGAAGGGCGUGUCAUACUCCUUGUGUACCGCAGCGUUCACAU
KU940224    ACUGUCGUGGUUCUAGGGAGUCAAGAAGGAGCAGUUCACACGGCCCUUGCUGGAGCUCUAGAGGCUGAGAUGGAUGGUGCAAAGGGAAGGCUGUCCUCUGGCCACUUGAAAUGUCGCCUGAAAAUGGAUAAACUUAGAUUGAAGGGCGUGUCAUACUCCUUGUGUACCGCAGCGUUCACAU


            1111111111111111111111111111111111111111111111111111111111111111111111111111111111111111111111111111111111111111111111111111111111111111111111111111111111111111111111111111111
            8888888888888888888888888888888888888888888888888888888888888888888888888888888889999999999999999999999999999999999999999999999999999999999999999999999999999999999999999999
            1111111112222222222333333333344444444445555555555666666666677777777778888888888999999999900000000001111111111222222222233333333334444444444555555555566666666667777777777888888888899
Sequence    1234567890123456789012345678901234567890123456789012345678901234567890123456789012345678901234567890123456789012345678901234567890123456789012345678901234567890123456789012345678901
-------------------------------------------------------------------------------------------------------------------------------------------------------------------------------------------------
NC_012532   UCACCAAGGUCCCAGCUGAAACACUGCAUGGAACAGUCACAGUGGAGGUGCAGUAUGCAGGGACAGAUGGACCCUGCAAGAUCCCAGUCCAGAUGGCGGUGGACAUGCAGACCCUGACCCCAGUUGGAAGGCUGAUAACCGCCAACCCCGUGAUUACUGAAAGCACUGAGAACUCAAAGAU
KU922960    UCACCAAGAUCCCGGCUGAAACACUGCACGGGACAGUCACAGUGGAGGUACAGUACGCAGGGACAGAUGGACCUUGCAAGGUUCCAGCCCAGAUGGCGGUGGACAUGCAAACUCUGACCCCAGUUGGGAGGUUGAUAACCGCUAACCCCGUAAUCACUGAAAGCACUGAGAACUCUAAGAU
KU922923    UCACCAAGAUCCCGGCUGAAACACUGCACGGGACAGUCACAGUGGAGGUACAGUACGCAGGGACAGAUGGACCUUGCAAGGUUCCAGCCCAGAUGGCGGUGGACAUGCAAACUCUGACCCCAGUUGGGAGGUUGAUAACCGCUAACCCCGUAAUCACUGAAAGCACUGAGAACUCUAAGAU
KU820898    UCACCAAGAUCCCGGCUGAAACACUGCACGGGACAGUCACAGUGGAGGUACAGUACGCAGGGACAGAUGGACCUUGCAAGGUUCCAGCUCAGAUGGCGGUGGACAUGCAAACUCUGACCCCAGUUGGGAGGUUGAUAACCGCUAACCCCGUAAUCACUGAAAGCACUGAGAACUCUAAGAU
KU321639    UCACCAAGAUCCCGGCUGAAACACUGCACGGGACAGUCACAGUGGAGGUACAGUACGCAGGGACAGAUGGACCUUGCAAGGUUCCAGCUCAGAUGGCGGUGGACAUGCAAACUCUGACCCCAGUUGGGAGGUUGAUAACCGCUAACCCCGUAAUCACUGAAAGCACUGAGAACUCUAAGAU
KU820899    UCACCAAGAUCCCGGCUGAAACACUGCACGGGACAGUCACAGUGGAGGUACAGUACGCAGGGACAGAUGGACCUUGCAAGGUUCCAGCUCAGAUGGCGGUGGACAUGCAAACUCUGACCCCAGUUGGGAGGCUGAUAACCGCUAACCCCGUAAUCACUGAAAGCACUGAGAACUCCAAGAU
KU853012    UCACCAAGAUCCCGGCUGAAACACUGCACGGGACAGUCACAGUGGAGGUACAGUACGCAGGGACAGAUGGACCUUGCAAGGUUCCAGCUCAGAUGGCGGUGGACAUGCAAACUCUGACCCCAGUUGGGAGGUUGAUAACCGCCAACCCCGUAAUCACUGAAAGCACUGAGAACUCUAAGAU
KU729217    UCACCAAGAUCCCGGCUGAAACACUGCACGGGACAGUCACAGUGGAGGUACAGUACGCAGGGACAGAUGGACCUUGCAAGGUUCCAGCUCAGAUGGCGGUGGACAUGCAAACUCUGACCCCAGUUGGGAGGUUGAUAACCGCUAACCCCGUAAUCACUGAAAGCACUGAGAACUCUAAGAU
KU729218    UCACCAAGAUCCCGGCUGAAACACUGCACGGGACAGUCACAGUGGAGGUACAGUACGCAGGGACAGAUGGACCUUGCAAGGUUCCAGCUCAGAUGGCGGUGGACACGCAAACUCUGACCCCAGUUGGGAGGUUGAUAACCGCUAACCCCGUAAUCACUGAAAGCACUGAGAACUCUAAGAU
KU761564    UCACCAAGAUCCCGGCUGAAACACUGCACGGGACAGUCACAGUGGAGGUACAGUACGCAGGGACAGAUGGACCUUGCAAGGUUCCAGCUCAGAUGGCGGUGGACAUGCAAACUCUGACCCCAGUUGGGAGGUUGAUAACCGCUAACCCCGUAAUCACUGAAAGCACUGAGAACUCUAAGAU
KU740184    UCACCAAGAUCCCGGCUGAAACACUGCACGGGACAGUCACAGUGGAGGUACAGUACGCAGGGACAGAUGGACCUUGCAAGGUUCCAGCUCAGAUGGCGGUGGACAUGCAAACUCUGACCCCAGUUGGGAGGUUGAUAACCGCUAACCCCGUAAUCACUGAAAGCACUGAGAACUCUAAGAU
KU744693    UCACCAAGAUCCCGGCUGAAACAGUGGACGGGACAGUCACAGUGGAGGGACAGUACGGAGGGACAGAUGGACCUUGCAAGGUUCCAGCUCAGAUGGCGGUGGACAUGCAGACUCUGACCCCAGUUGGGAGGUUGAUAACCGCUAACCCCGUAAUCACUGAAAGCACUGAGAACUCUAAGAU
KU497555    UCACCAAGAUCCCGGCUGAAACACUGCACGGGACAGUCACAGUGGAGGUACAGUACGCAGGGACAGAUGGACCUUGCAAGGUUCCAGCUCAGAUGGCGGUGGACAUGCAAACUCUGACCCCAGUUGGGAGGUUGAUAACCGCUAACCCCGUAAUCACUGAAAGCACUGAGAACUCUAAGAU
KU527068    UCACCAAGAUCCCGGCUGAAACACUGCACGGGACAGUCACAGUGGAGGUACAGUACGCAGGGACAGAUGGACCUUGCAAGGUUCCAGCUCAGAUGGCGGUGGACAUGCAAACUCUGACCCCAGUUGGGAGGUUGAUAACCGCUAACCCCGUAAUCACUGAAAGCACUGAGAACUCUAAGAU
KU501217    UCACCAAGAUCCCGGCUGAAACACUGCACGGGACAGUCACAGUGGAGGUACAGUACGCAGGGACAGAUGGACCUUGCAAGGUUCCAGCUCAGAUGGCGGUGGACAUGCAAACUCUGACCCCAGUUGGGAGGUUGAUAACCGCUAACCCCGUAAUCACUGAAAGCACUGAGAACUCUAAGAU
KU501216    UCACCAAGAUCCCGGCUGAAACACUGCACGGGACAGUCACAGUGGAGGUACAGUACGCAGGGACAGAUGGACCUUGCAAGGUUCCAGCUCAGAUGGCGGUGGACAUGCAAACUCUGACCCCAGUUGGGAGGUUGAUAACCGCUAACCCCGUAAUCACUGAAAGCACUGAGAACUCUAAGAU
KU501215    UCACCAAGAUCCCGGCUGAAACACUGCACGGGACAGUCACAGUGGAGGUACAGUACGCAGGGACAGAUGGACCUUGCAAGGUUCCAGCUCAGAUGGCGGUGGACAUGCAAACUCUGACCCCAGUUGGGAGGUUGAUAACCGCUAACCCCGUAAUCACUGAAAGCACUGAGAACUCUAAGAU
KU365780    UCACCAAGAUCCCGGCUGAAACACUGCACGGGACAGUCACAGUGGAGGUACAGUACGCAGGGACAGAUGGACCUUGCAAGGUUCCAGCUCAGAUGGCGGUGGACAUGCAAACUCUGACCCCAGUUGGGAGGUUGAUAACCGCUAACCCCGUAAUCACUGAAAGCACUGAGAACUCUAAGAU
KU365779    UCACCAAGAUCCCGGCUGAAACACUGCACGGGACAGUCACAGUGGAGGUACAGUACGCAGGGACAGAUGGACCUUGCAAGGUUCCAGCUCAGAUGGCGGUGGACAUGCAAACUCUGACCCCAGUUGGGAGGUUGAUAACCGCUAACCCCGUAAUCACUGAAAGCACUGAGAACUCUAAGAU
KU707826    UCACCAAGAUCCCGGCUGAAACACUGCACGGGACAGUCACAGUGGAGGUACAGUACGCAGGGACAGAUGGACCUUGCAAGGUUCCAGCUCAGAUGGCGGUGGACAUGCAAACUCUGACCCCAGUUGGGAGGUUGAUAACCGCUAACCCCGUAAUCACUGAAAGCACUGAGAACUCUAAGAU
KU365778    UCACCAAGAUCCCGGCUGAAACACUGCACGGGACAGUCACAGUGGAGGUACAGUACGCAGGGACAGAUGGACCUUGCAAGGUUCCAGCUCAGAUGGCGGUGGACAUGCAAACUCUGACCCCAGUUGGGAGGUUGAUAACCGCUAACCCCGUAAUCACUGAAAGCACUGAGAACUCUAAGAU
KU365777    UCACCAAGAUCCCGGCUGAAACACUGCACGGGACAGUCACAGUGGAGGUACAGUACGCAGGGACAGAUGGACCUUGCAAGGUUCCAGCUCAGAUGGCGGUGGACAUGCAAACUCUGACCCCAGUUGGGAGGUUGAUAACCGCUAACCCCGUAAUCACUGAAAGCACUGAGAACUCUAAGAU
KU312312    UCACCAAGAUCCCGGCUGAAACACUGCACGGGACAGUCACAGUGGAGGUACAGUACGCAGGGACAGAUGGACCUUGCAAGGUUCCAGCUCAGAUGGCGGUGGACAUGCAAACUCUGACCCCAGUUGGGAGGUUGAUAACCGCUAACCCCGUAAUCACUGAAAGCACUGAGAACUCUAAGAU
KF268950    UUACCAAGGUCCCAGCUGAAACAUUGCAUGGAACAGUUACAGUGGAGGUGCAGUAUGCAGGGACAGAUGGACCCUGCAAGGUCCCAGCCCAGAUGGCGGUGGACAUGCAGACCCUGACCCCAGUCGGGAGGCUGAUAACCGCCAACCCUGUGAUUACUGAAAGCACUGAGAAUUCAAAGAU
KF268949    UCACCAAGGUCCCAGCUGAAACAUUGCAUGGAACAGUUACAGUGGAGGUGCAGUAUGCAGGGACAGAUGGACCCUGCAAGGUCCCAGCUCAGAUGGCGGUGGACAUGCAGACCCUGACCCCAGUCGGAAGGCUGAUAACCGCCAACCCCGUGAUUACUGAAAGCACUGAGAAUUCAAAGAU
KF268948    UUACCAAGGUCCCAGCUGAAACAUUGCAUGGAACAGUUACAGUGGAGGUGCAGUAUGCAGGGACAGAUGGACCCUGCAAGGUCCCAGCCCAGAUGGCGGUGGACAUGCAGACCCUGACCCCAGUCGGGAGGCUGAUAACCGCCAACCCUGUGAUUACUGAAAGCACUGAGAAUUCAAAGAU
KJ776791    UCACCAAGAUCCCGGCUGAAACACUGCACGGGACAGUCACAGUGGAGGUACAGUACGCAGGGACAGAUGGACCUUGCAAGGUUCCAGCUCAGAUGGCGGUGGACAUGCAAACUCUGACCCCAGUUGGGAGGUUGAUAACCGCUAACCCCGUAAUCACUGAAAGCACUGAGAACUCUAAGAU
KF383119    UCACCAAGGUCCCAGCUGAAACACUGCAUGGAACAGUCACAGUGGAGGUGCAGUAUGCAGGGACAGAUGGACCCUGCAAGGUCCCAGCCCAGAUGGCGGUGGACAUGCAGACCCUGACCCCAGUUGGAAGGCUGAUAACCGCCAACCCCGUGAUUACUGAAAGCACUGAGAACUCAAAGAU
KF383118    UUACCAAGGUCCCAGCUGAAACAUUGCAUGGAACAGUUACAGUGGAGGUGCAGUCUGCAGGGACAGAUGGACCCUGCAAGGUCCCAGCCCAGAUGGCGGUGGACAUGCAGACCCUGACCCCAGUUGGAAGGCUGAUAACCGCCAACCCCGUGAUUACUGAAAGCACUGAGAACUCAAAGAU
KF383117    UUACCAAGGUCCCAGCUGAAACAUUGCAUGGAACAGUUACAGUGGAGGUGCAGUAUGCAGGGACAGAUGGACCUUGCAAGGUCCCAGCCCAGAUGGCGGUGGACAUGCAGACUCUGACCCCAGUUGGAAGGCUGAUAACCGCCAACCCUGUGAUCACUGAAAGUACUGAGAAUUCAAAGAU
KF383116    CAGCGAAGGUCCCAGCUGAAACAUUGCAUGGAACAGUCACAGUGGAGGUGCAGUAUGCAGGGACAGAUGGACCCUGCAAGGUCCCAGCCCAGAUGGCGGUAGACAUGCAGACCCUGACCCCAGUUGGAAGGCUGAUAACCGCCAACCCUGUGAUCACUGAAAGUACUGAGAAUUCAAAGAU
KF383115    UCACCAAGGUCCCAGCUGAAACACUGCAUGGAACAGUCACAGUGGAGGUGCAGUAUGCAGGGACAGAUGGACCCUGCAAGGUCCCAGCCCAGAUGGCGGUGGACAUGCAGACUUUGACCCCAGUCGGAAGGCUGAUAACUGCCAACCCCGUGAUUACUGAAAGCACUGAAAAUUCAAAGAU
EU545988    UCACCAAGAUCCCGGCUGAAACACUGCACGGGACAGUCACAGUGGAGGUACAGUACGCAGGGACAGAUGGACCCUGCAAGGUUCCAGCUCAGAUGGCGGUGGACAUGCAAACUCUGACCCCAGUUGGGAGGCUGAUAACCGCUAACCCCUGUAAUCACUGAAAGCACUGAGAACUCUAAGAU
KU955589    UCACCAAGAUCCCGGCUGAAACACUGCACGGGACAGUCACAGUGGAGGUACAGUACGCAGGGACAGAUGGACCUUGCAAGGUUCCAGCUCAGAUGGCGGUGGACAUGCAAACUCUGACCCCAGUUGGGAGGCUGAUAACCGCUAACCCCGUAAUCACUGAAAGCACUGAGAACUCCAAGAU
KU955590    UCACCAAGAUCCCGGCUGAAACACUGCACGGGACAGUCACAGUGGAGGUACAGUACGCAGGGACAGAUGGACCUUGCAAGGUUCCAGCUCAGAUGGCGGUGGACAUGCAAACUCUGACCCCAGUUGGGAGGUUGAUAACCGCUAACCCCGUAAUCACUGAAAGCACUGAGAACUCUAAGAU
KU940228    UCACCAAGAUCCCGGCUGAAACACUGCACGGGACAGUCACAGUGGAGGUACAGUACGCAGGGACUGAUGGACCUUGCAAGGUUCCAGCUCAGAUGGCGGUGGACAUGCAAACUCUGACCCCAGUUGGGAGGUUGAUAACCGCUAACCCCGUAAUCACUGAAAGCACUGAGAACUCUAAGAU
KU991811    UCACCAAGAUCCCGGCUGAAACACUGCACGGGACAGUCACAGUGGAGGUACAGUACGCAGGGACAGAUGGACCUUGCAAGGUUCCAGCUCAGAUGGCGGUGGACAUGCAAACUCUGACCCCAGUUGGGAGGUUGAUAACCGCUAACCCCGUAAUCACUGAAAGCACUGAGAACUCUAAGAU
KU509998    UCACCAAGAUCCCGGCUGAAACACUGCACGGGACAGUCACAGUGGAGGUACAGUACGCAGGGACAGAUGGACCUUGCAAGGUUCCAGCUCAGAUGGCGGUGGACAUGCAAACUCUGACCCCAGUUGGGAGGUUGAUAACCGCUAACCCCGUAAUCACUGAAAGCACUGAGAACUCUAAGAU
KX056898    UCACCAAGAUCCCGGCUGAAACACUGCACGGGACAGUCACAGUGGAGGUACAGUACGCAGGGACAGAUGGACCUUGCAAGGUUCCAGCUCAGAUGGCGGUGGACAUGCAAACUCUGACCCCAGUUGGGAGGUUGAUAACCGCUAACCCCGUAAUCACUGAAAGCACUGAGAACUCUAAGAU
KX117076    UCACCAAGAUCCCGGCUGAAACACUGCACGGGACAGUCACAGUGGAGGUACAGUACGCAGGGACAGAUGGACCUUGCAAGGUUCCAGCUCAGAUGGCGGUGGACAUGCAAACUCUGACCCCAGUUGGGAGGCUGAUAACCGCUAACCCCGUAAUCACUGAAAGCACUGAGAACUCCAAGAU
KX185891    UCACCAAGAUCCCGGCUGAAACACUGCACGGGACAGUCACAGUGGAGGUACAGUACGCAGGGACAGAUGGACCUUGCAAGGUUCCAGCUCAGAUGGCGGUGGACAUGCAAACUCUGACCCCAGUUGGGAGGCUGAUAACCGCUAACCCCGUAAUCACUGAAAGCACUGAGAACUCCAAGAU
KX247646    UCACCAAGAUCCCGGCUGAAACACUGCACGGGACAGUCACAGUGGAGGUACAGUACGCAGGGACAGAUGGACCUUGCAAGGUUCCAGCUCAGAUGGCGGUGGACAUGCAAACUCUGACCCCAGUUGGGAGGUUGAUAACCGCUAACCCCGUAAUCACUGAAAGCACUGAGAACUCUAAGAU
KX262887    UCACCAAGAUCCCGGCUGAAACACUGCACGGGACAGUCACAGUGGAGGUACAGUACGCAGGGACAGAUGGACCUUGCAAGGUUCCAGCUCAGAUGGCGGUGGACAUGCAAACUCUGACCCCAGUUGGGAGGUUGAUAACCGCUAACCCCGUAAUCACUGAAAGCACUGAGAACUCUAAGAU
KX280026    UCACCAAGAUCCCGGCUGAAACACUGCACGGGACAGUCACAGUGGAGGUACAGUACGCAGGGACAGAUGGACCUUGCAAGGUUCCAGCUCAGAUGGCGGUGGACAUGCAAACUCUGACCCCAGUUGGGAGGUUGAUAACCGCUAACCCCGUAAUCACUGAAAGCACUGAGAACUCUAAGAU
KU758877    UCACCAAGAUCCCGGCUGAAACACUGCACGGGACAGUCACAGUGGAGGUACAGUACGCAGGGACAGAUGGACCUUGCAAGGUUCCAGCUCAGAUGGCGGUGGACAUGCAAACUCUGACCCCAGUUGGGAGGUUGAUAACCGCUAACCCCGUAAUCACUGAAAGCACUGAGAACUCUAAGAU
KU866423    UCACCAAGAUCCCGGCUGAAACACUGCACGGGACAGUCACAGUGGAGGUACAGUACGCAGGGACAGAUGGACCUUGCAAGGUUCCAGCUCAGAUGGCGGUGGACAUGCAAACUCUGACCCCAGUUGGGAGGCUGAUAACCGCUAACCCCGUAAUCACUGAAAGCACUGAGAACUCCAAGAU
KX377336    UCACCAAGAUCCCGGCUGAAACGCUGCAUGGGACAGUCACAGUGGAGGUACAGUAUGCAGGGACAGAUGGACCCUGCAAGGUUCCAGCUCAGAUGGCGGUGGAUAUGCAAACUCUGACCCCAGUUGGGAGGUUGAUAACCGCUAACCCUGUGAUCACUGAAAGCACUGAGAAUUCAAAGAU
KX377337    UCACCAAGAUCCCGGCUGAAACACUGCACGGGACAGUCACAGUGGAGGUACAGUACGCAGGGACAGAUGGACCUUGCAAGGUUCCAGCUCAGAUGGCGGUGGACAUGCAAACUCUGACCCCAGUUGGGAGGUUGAUAACCGCUAACCCCGUAAUCACUGAAAGCACUGAGAACUCUAAGAU
KX369547    UCACCAAGAUCCCGGCUGAAACACUGCACGGGACAGUCACAGUGGAGGUACAGUACGCAGGGACAGAUGGACCUUGCAAGGUUCCAGCUCAGAUGGCGGUGGACAUGCAAACUCUGACCCCAGUUGGGAGGUUGAUAACCGCUAACCCCGUAAUCACUGAAAGCACUGAGAACUCUAAGAU
KX548902    UCACCAAGAUCCCGGCUGAAACACUGCACGGGACAGUCACAGUGGAGGUACAGUACGCAGGGACAGAUGGACCUUGCAAGGUUCCAGCUCAGAUGGCGGUGGACAUGCAAACUCUGACCCCAGUUGGGAGGUUGAUAACCGCUAACCCCGUAAUCACUGAAAGCACUGAGAACUCUAAGAU
KX601166    UUACCAAGGUCCCAGCUGAAACAUUGCAUGGAACAGUCACAGUGGAGGUGCAGUAUGCAGGGACAGACGGACCCUGCAAAGUCCCAGCCCAGAUGGCGGUGGACAUGCAGACCCUGACCCCAGUUGGAAGGCUGAUAACCGCCAAUCCUGUGAUCACUGAAAGUACUGAGAAUUCAAAGAU
KX601167    UCACCAAGAUCCCGGCUGAAACGCUGCAUGGGACAGUCACAGUGGAGGUACAGUAUGCAGGGACAGAUGGACCCUGCAAGGUUCCAGCUCAGAUGGCGGUGGAUAUGCAAACUCUGACCCCAGUUGGGAGGUUGAUAACCGCUAACCCUGUGAUCACUGAAAGCACUGAGAAUUCAAAGAU
KX601168    UCACCAAGAUCCCGGCUGAAACACUGCACGGGACAGUCACAGUGGAGUUACAGUACGCAGGGACAGAUGGACCUUGCAAGGUUCCAGCUCAGAUGGCGGUGGACAUGCAAACUCUGACCCCAGUUGGGAGGUUGAUAACCGCUAACCCCGUAAUCACUGAAAGCACUGAGAACUCUAAGAU
KX766028    UCACCAAGAUCCCGGCUGAAACACUGCACGGGACAGUCACAGUGGAGGUACAGUACGCAGGGACAGAUGGACCUUGCAAGGUUCCAGCUCAGAUGGCGGUGGACAUGCAAACUCUGACCCCAGUUGGGAGGUUGAUAACCGCUAACCCCGUAAUCACUGAAAGCACUGAGAACUCUAAGAU
KX766029    UCACCAAGAUCCCGGCUGAAACACUGCACGGGACAGUCACAGUGGAGGUACAGUACGCAGGGACAGAUGGACCUUGCAAGGUUCCAGCUCAGAUGGCGGUGGACAUGCAAACUCUGACCCCAGUUGGGAGGUUGAUAACCGCUAACCCCGUAAUCACUGAAAGCACUGAGAACUCUAAGAU
KU940224    UCACCAAGAUCCCGGCUGAAACACUGCACGGGACAGUCACAGUGGAGGUACAGUACGCAGGGACUGAUGGACCUUGCAAGGUUCCAGCUCAGAUGGCGGUGGACAUGCAAACUCUGACCCCAGUUGGGAGGUUGAUAACCGCUAACCCCGUAAUCACUGAAAGCACUGAGAACUCUAAGAU


            1111111122222222222222222222222222222222222222222222222222222222222222222222222222222222222222222222222222222222222222222222222222222222222222222222222222222222222222222222222
            9999999900000000000000000000000000000000000000000000000000000000000000000000000000000000000000000000000001111111111111111111111111111111111111111111111111111111111111111111111111
            9999999900000000001111111111222222222233333333334444444444555555555566666666667777777777888888888899999999990000000000111111111122222222223333333333444444444455555555556666666666777
Sequence    2345678901234567890123456789012345678901234567890123456789012345678901234567890123456789012345678901234567890123456789012345678901234567890123456789012345678901234567890123456789012
-------------------------------------------------------------------------------------------------------------------------------------------------------------------------------------------------
NC_012532   GAUGUUGGAGCUUGACCCACCAUUUGGGGAUUCUUACAUUGUCAUAGGAGUUGGGGACAAGAAAAUCACCCACCACUGGCAUAGGAGUGGUAGCACCAUCGGAAAGGCAUUUGAGGCCACUGUGAGAGGCGCCAAGAGAAUGGCAGUCCUGGGGGAUACAGCCUGGGACUUCGGAUCAGUC
KU922960    GAUGCUGGAACUUGAUCCACCAUUUGGGGACUCUUACAUUGUCAUAGGAGUCGGGGAGAAGAAGAUCACCCACCACUGGCACAGGAGUGGCAGCACCAUUGGAAAAGCAUUUGAAGCCACUGUGAGAGGUGCCAAGAGAAUGGCAGUCUUGGGAGACACAGCCUGGGACUUUGGAUCAGUU
KU922923    GAUGCUGGAACUUGAUCCACCAUUUGGGGACUCUUACAUUGUCAUAGGAGUCGGGGAGAAGAAGAUCACCCACCACUGGCACAGGAGUGGCAGCACCAUUGGAAAAGCAUUUGAAGCCACUGUGAGAGGUGCCAAGAGAAUGGCAGUCUUGGGAGACACAGCCUGGGACUUUGGAUCAGUU
KU820898    GAUGCUGGAACUUGAUCCACCAUUUGGGGACUCUUACAUUGUCAUAGGAGUCGGGGAGAAGAAGAUCACCCACCACUGGCACAGGAGUGGCAGCACCAUUGGAAAAGCAUUUGAAGCCACUGUGAGAGGUGCCAAGAGAAUGGCAGUCUUGGGAGACACAGCCUGGGACUUUGGAUCAGUU
```

```
KU321639    GAUGCUGGAACUUGAUCCACCAUUUGGGGACUCUUACAUUGUCAUAGGAGUCGGGGAGAAGAAGAUCACCCACCACUGGCACAGGAGUGGCAGCACCAUUGGAAAAGCAUUUGAAGCCACUGUGAGAGGUGCCAAGAGAAUGGCAGUCUUGGGAGACACAGCCUGGGACUUUGGAUCAGUU
KU820899    GAUGCUGGAACUUGAUCCACCAUUUGGGGACUCUUACAUUGUCAUAGGAGUCGGGGAGAAGAAGAUCACCCACCACUGGCACAGGAGUGGCAGCACCAUUGGAAAAGCAUUUGAAGCCACUGUGAGAGGUGCCAGGAGAAUGGCAGUCUUGGGAGACACAGCCUGGGACUUUGGAUCAGUU
KU853012    GAUGCUGGAACUUGAUCCACCAUUUGGGGACUCUUACAUUGUCAUAGGAGUCGGGGAGAAGAAGAUCACCCACCACUGGCACAGGAGUGGCAGCACCAUUGGAAAAGCAUUUGAAGCCACUGUGAGAGGUGCCAAGAGAAUGGCAGUCUUGGGAGACACAGCCUGGGACUUUGGAUCAGUU
KU729217    GAUGCUGGAACUUGAUCCACCAUUUGGGGACUCUUACAUUGUCAUAGGAGUCGGGGAGAAGAAGAUCACCCACCACUGGCACAGGAGUGGCAGCACCAUUGGAAAAGCAUUUGAAGCCACUGUGAGAGGUGCCAAGAGAAUGGCAGUCUUGGGAGACACAGCCUGGGACUUUGGAUCAGUU
KU729218    GAUGCUGGAACUUGAUCCACCAUUUGGGGACUCUUACAUUGUCAUAGGAGUCGGGGAGAAGAAGAUCACCCACCACUGGCACAGGAGUGGCAGCACCAUUGGAAAAGCAUUUGAAGCCACUGUGAGAGGUGCCAAGAGAAUGGCAGUCUUGGGAGACACAGCCUGGGACUUUGGAUCAGUU
KU761564    GAUGCUGGAACUUGAUCCACCAUUUGGGGACUCUUACAUUGUCAUAGGAGUCGGGGAGAAGAAGAUCACCCACCACUGGCACAGGAGUGGCAGCACCAUUGGAAAAGCAUUUGAAGCCACUGUGAGAGGUGCCAAGAGAAUGGCAGUCUUGGGAGACACAGCCUGGGACUUUGGAUCAGUU
KU740184    GAUGCUGGAACUUGAUCCACCAUUUGGGGACUCUUACAUUGUCAUAGGAGUCGGGGAGAAGAAGAUCACCCACCACUGGCACAGGAGUGGCAGCACCAUUGGAAAAGCAUUUGAAGCCACUGUGAGAGGUGCCAAGAGAAUGGCAGUCUUGGGAGACACAGCCUGGGACUUUGGAUCAGUU
KU744693    GAUGCUGGAACUUGAUCCACCAUUUGGGGACUCUUACAUUGUCAUAGGAGUCGGGGAGAAGAAGAUCACCCACCACUGGCACAGGAGUGGCAGCACCAUUGGAAAAGCAUUUGAAGCCACUGUGAGAGGUGCCAAGAGAAUGGCAGUCUUGGGAGACACAGCCUGGGACUUUGGAUCAGUU
KU497555    GAUGCUGGAACUUGAUCCACCAUUUGGGGACUCUUACAUUGUCAUAGGAGUCGGGGAGAAGAAGAUCACCCACCACUGGCACAGGAGUGGCAGCACCAUUGGAAAAGCAUUUGAAGCCACUGUGAGAGGUGCCAAGAGAAUGGCAGUCUUGGGAGACACAGCCUGGGACUUUGGAUCAGUU
KU527068    GAUGCUGGAACUUGAUCCACCAUUUGGGGACUCUUACAUUGUCAUAGGAGUCGGGGAGAAGAAGAUCACCCACCACUGGCACAGGAGUGGCAGCACCAUUGGAAAAGCAUUUGAAGCCACUGUGAGAGGUGCCAAGAGAAUGGCAGUCUUGGGAGACACAGCCUGGGACUUUGGAUCAGUU
KU501217    GAUGCUGGAACUUGAUCCACCAUUUGGGGACUCUUACAUUGUCAUAGGAGUCGGGGAGAAGAAGAUCACCCACCACUGGCACAGGAGUGGCAGCACCAUUGGAAAAGCAUUUGAAGCCACUGUGAGAGGUGCCAAGAGAAUGGCAGUCUUGGGAGACACAGCCUGGGACUUUGGAUCAGUU
KU501216    GAUGCUGGAACUUGAUCCACCAUUUGGGGACUCUUACAUUGUCAUAGGAGUCGGGGAGAAGAAGAUCACCCACCACUGGCACAGGAGUGGCAGCACCAUUGGAAAAGCAUUUGAAGCCACUGUGAGAGGUGCCAAGAGAAUGGCAGUCUUGGGAGACACAGCCUGGGACUUUGGAUCAGUU
KU501215    GAUGCUGGAACUUGAUCCACCAUUUGGGGACUCUUACAUUGUCAUAGGAGUCGGGGAGAAGAAGAUCACCCACCACUGGCACAGGAGUGGCAGCACCAUUGGAAAAGCAUUUGAAGCCACUGUGAGAGGUGCCAAGAGAAUGGCAGUCUUGGGAGACACAGCCUGGGACUUUGGAUCAGUU
KU365780    GAUGCUGGAACUUGAUCCACCAUUUGGGGACUCUUACAUUGUCAUAGGAGUCGGGGAGAAGAAGAUCACCCACCACUGGCACAGGAGUGGCAGCACCAUUGGAAAAGCAUUUGAAGCCACUGUGAGAGGUGCCAAGAGAAUGGCAGUCUUGGGAGACACAGCCUGGGACUUUGGAUCAGUU
KU365779    GAUGCUGGAACUUGAUCCACCAUUUGGGGACUCUUACAUUGUCAUAGGAGUCGGGGAGAAGAAGAUCACCCACCACUGGCACAGGAGUGGCAGCACCAUUGGAAAAGCAUUUGAAGCCACUGUGAGAGGUGCCAAGAGAAUGGCAGUCUUGGGAGACACAGCCUGGGACUUUGGAUCAGUU
KU707826    GAUGCUGGAACUUGAUCCACCAUUUGGGGACUCUUACAUUGUCAUAGGAGUCGGGGAGAAGAAGAUCACCCACCACUGGCACAGGAGUGGCAGCACCAUUGGAAAAGCAUUUGAAGCCACUGUGAGAGGUGCCAAGAGAAUGGCAGUCUUGGGAGACACAGCCUGGGACUUUGGAUCAGUU
KU365778    GAUGCUGGAACUUGAUCCACCAUUUGGGGACUCUUACAUUGUCAUAGGAGUCGGGGAGAAGAAGAUCACCCACCACUGGCACAGGAGUGGCAGCACCAUUGGAAAAGCAUUUGAAGCCACUGUGAGAGGUGCCAAGAGAAUGGCAGUCUUGGGAGACACAGCCUGGGACUUUGGAUCAGUU
KU365777    GAUGCUGGAACUUGAUCCACCAUUUGGGGACUCUUACAUUGUCAUAGGAGUCGGGGAGAAGAAGAUCACCCACCACUGGCACAGGAGUGGCAGCACCAUUGGAAAAGCAUUUGAAGCCACUGUGAGAGGUGCCAAGAGAAUGGCAGUCUUGGGAGACACAGCCUGGGACUUUGGAUCAGUU
KU312312    GAUGCUGGAACUUGAUCCACCAUUUGGGGACUCUUACAUUGUCAUAGGAGUCGGGGAGAAGAAGAUCACCCACCACUGGCACAGGAGUGGCAGCACCAUUGGAAAAGCAUUUGAAGCCACUGUGAGAGGUGCCAAGAGAAUGGCAGUCUUGGGAGACACAGCCUGGGACUUUGGAUCAGUU
KF268950    GAUGUUGGAGCUCGACCCACCAUUUGGGGAUUCUUACAUUGUCAUAGGAGUUGGGGACAAGAAAAUCACCCAUCACUGGCAUAGGAGUGGUAGCACCAUCGGAAAAGCAUUUGAGGCCACUGUGAGAGGUGCCAAGAGAAUGGCAGUUCUGGGGGAUACAGCCUGGGACUUCGGAUCAGUC
KF268949    GAUGUUGGAGCUCGACCCACCAUUUGGGGAUUCUUACAUUGUCAUAGGAGUUGGGGACAAGAAAAUUACCCACCACUGGCAUAGGAGUGGUAGCACCAUCGGAAAAGCAUUUGAGGCCACUGUGAGAGGUGCCAAGAGAAUGGCAGUUCUGGGGGACACAGCCUGGGACUUUGGAUCAGUC
KF268948    GAUGUUGGAGCUCGACCCACCAUUUGGGGAUUCUUACAUUGUCAUAGGAGUUGGGGACAAGAAAAUCACCCAUCACUGGCAUAGGAGUGGUAGCACCAUCGGAAAAGCAUUUGAGGCCACUGUGAGAGGUGCCAAGAGAAUGGCAGUUCUGGGGGAUACAGCCUGGGACUUCGGAUCAGUC
KJ776791    GAUGCUGGAACUUGAUCCACCAUUUGGGGACUCUUACAUUGUCAUAGGAGUCGGGGAGAAGAAGAUCACCCACCACUGGCACAGGAGUGGCAGCACCAUUGGAAAAGCAUUUGAAGCCACUGUGAGAGGUGCCAAGAGAAUGGCAGUCUUGGGAGACACAGCCUGGGACUUUGGAUCAGUU
KF383119    GAUGUUGGAGCUUGACCCACCAUUUGGGGAUUCUUACAUUGUCAUAGGAGUUGGGGACAAGAAAAUCACCCACCACUGGCAUAGGAGUGGUAGCACCAUCGGAAAGGCAUUUGAAGCCACUGUGAGAGGCGCCAAGAGAAUGGCAGUCCUGGGGGAUACAGCCUGGGACUUCGGAUCAGUC
KF383118    GAUGUUGGAGCUUGACCCACCAUUUGGGGAUUCUUACAUUGUCAUAGGAGUUGGGGACAAGAAAAUCACCCACCACUGGCAUAGGAGUGGUAGCACCAUCGGAAAGGCAUUUGAAGCCACUGUGAGAGGCGCCAAGAGAAUGGCAGUCCUGGGGGAUACAGCCUGGGACUUCGGAUCAGUC
KF383117    GAUGUUGGAACUUGACCCACCAUUUGGGGAUUCUUACAUUGUCAUAGGAGUCGGGGACAAGAAAAUCACCCAUCACUGGCUCAAGAAAGGGAGCUCCAUCGGAAAGGCAUUUGAAGCCACUGUGAGAGGUGCCAAGAGAAUGGCAGUCUUGGGGGACACAGCCUGGGACUUUGGAUCAGUU
KF383116    GAUGUUGGAGCUCGACCCACCAUUUGGGGAUUCUUACAUUGUCAUAGGAGUCGGGGACAAGAAAAUCACCCAUCACUGGCAUCGGAGUGGUAGCACCAUCGGAAAGGCAUUUGAAGCCACUGUGAGAGGUGCCAAGAGAAUGGCAGUCUUGGGGGACACAGCCUGGGACUUUGGAUCAGUU
KF383115    GAUGUUGGAGCUCGACCCACCAUUUGGGGAUUCUUACAUUGUCAUAGGAGUUGGAGAUAAGAAAAUCACCCACCACUGGCAUAGGAGUGGUAGCACCAUCGGAAAAGCAUUUGAGGCCACUGUGAGAGGUGCCAAGAGAAUGGCAGUUUUGGGGGACACAGCUUGGGACUUUGGAUCCGUC
EU545988    GAUGCUGGAACUUGAUCCACCAUUUGGGGACUCUUACAUUGUCAUAGGAGUCGGGGAGAAGAAGAUCACCCAUCACUGGCACAGGAGUGGCAGCACCAUUGGAAAAGCAUUUGAAGCCACUGUGAGAGGUGCCAAGAGAAUGGCAGUCUUGGGAGACACAGCCUGGGAUUUUGGAUCAGUU
KU955589    GAUGCUGGAACUUGAUCCACCAUUUGGGGACUCUUACAUUGUCAUAGGAGUCGGGGAGAAGAAGAUCACCCACCACUGGCACAGGAGUGGCAGCACCAUUGGAAAAGCAUUUGAAGCCACUGUGAGAGGUGCCAGGAGAAUGGCAGUCUUGGGAGACACAGCCUGGGACUUUGGAUCAGUU
KU955590    GAUGCUGGAACUUGAUCCACCAUUUGGGGACUCUUACAUUGUCAUAGGAGUCGGGGAGAAGAAGAUCACCCACCACUGGCACAGGAGUGGCAGCACCAUUGGAAAAGCAUUUGAAGCCACUGUGAGAGGUGCCAAGAGAAUGGCAGUCUUGGGAGACACAGCCUGGGACUUUGGAUCAGUU
KU940228    GAUGCUGGAACUUGAUCCACCAUUUGGGGACUCCUACAUUGUCAUAGGAGUCGGGGAGAAGAAGAUCACCCACCACUGGCACAGGAGUGGCAGCACCAUUGGAAAAGCAUUUGAAGCCACUGUGAGAGGUGCCAAGAGAAUGGCAGUCUUGGGAGACACAGCCUGGGACUUUGGAUCAGUU
KU991811    GAUGCUGGAACUUGAUCCACCAUUUGGGGACUCUUACAUUGUCAUAGGAGUCGGGGAGAAGAAGAUCACCCACCACUGGCACAGGAGUGGCAGCACCAUUGGAAAAGCAUUUGAAGCCACUGUGAGAGGUGCCAAGAGAAUGGCAGUCUUGGGAGACACAGCCUGGGACUUUGGAUCAGUU
KU509998    GAUGCUGGAACUUGAUCCACCAUUUGGGGACUCUUACAUUGUCAUAGGAGUCGGGGAGAAGAAGAUCACCCACCACUGGCACAGGAGUGGCAGCACCAUUGGAAAAGCAUUUGAAGCCACUGUGAGAGGUGCCAAGAGAAUGGCAGUCUUGGGAGACACAGCCUGGGACUUUGGAUCAGUU
KX056898    GAUGCUGGAACUUGAUCCACCAUUUGGGGACUCUUACAUUGUCAUAGGAGUCGGGGAGAAGAAGAUCACCCACCACUGGCACAGGAGUGGCAGCACCAUUGGAAAAGCAUUUGAAGCCACUGUGAGAGGUGCCAAGAGAAUGGCAGUCUUGGGAGACACAGCCUGGGACUUUGGAUCAGUU
KX117076    GAUGCUGGAACUUGAUCCACCAUUUGGGGACUCUUACAUUGUCAUAGGAGUCGGGGAGAAGAAGAUCACCCACCACUGGCACAGGAGUGGCAGCACCAUUGGAAAAGCAUUUGAAGCCACUGUGAGAGGUGCCAGGAGAAUGGCAGUCUUGGGAGACACAGCCUGGGACUUUGGAUCAGUU
KX185891    GAUGCUGGAACUUGAUCCACCAUUUGGGGACUCUUACAUUGUCAUAGGAGUCGGGGAGAAGAAGAUCACCCACCACUGGCACAGGAGUGGCAGCACCAUUGGAAAAGCAUUUGAAGCCACUGUGAGAGGUGCCAGGAGAAUGGCAGUCUUGGGAGACACAGCCUGGGACUUUGGAUCAGUU
KX247646    GAUGCUGGAACUUGAUCCACCAUUUGGGGACUCUUACAUUGUCAUAGGAGUCGGGGAGAAGAAGAUCACCCACCACUGGCACAGGAGUGGCAGCACCAUUGGAAAAGCAUUUGAAGCCACUGUGAGAGGUGCCAAGAGAAUGGCAGUCUUGGGAGACACAGCCUGGGACUUUGGAUCAGUU
KX262887    GAUGCUGGAACUUGAUCCACCAUUUGGGGACUCUUACAUUGUCAUAGGAGUCGGGGAGAAGAAGAUCACCCACCACUGGCACAGGAGUGGCAGCACCAUUGGAAAAGCAUUUGAAGCCACUGUGAGAGGUGCCAAGAGAAUGGCAGUCUUGGGAGACACAGCCUGGGACUUUGGAUCAGUU
KX280026    GAUGCUGGAACUUGAUCCACCAUUUGGGGACUCUUACAUUGUCAUAGGAGUCGGGGAGAAGAAGAUCACCCACCACUGGCACAGGAGUGGCAGCACCAUUGGAAAAGCAUUUGAAGCCACUGUGAGAGGUGCCAAGAGAAUGGCAGUCUUGGGAGACACAGCCUGGGACUUUGGAUCAGUU
KU758877    GAUGCUGGAACUUGAUCCACCAUUUGGGGACUCUUACAUUGUCAUAGGAGUCGGGGAGAAGAAGAUCACCCACCACUGGCACAGGAGUGGCAGCACCAUUGGAAAAGCAUUUGAAGCCACUGUGAGAGGUGCCAAGAGAAUGGCAGUCUUGGGAGACACAGCCUGGGACUUUGGAUCAGUU
KU866423    GAUGCUGGAACUUGAUCCACCAUUUGGGGACUCUUACAUUGUCAUAGGAGUCGGGGAGAAGAAGAUCACCCACCACUGGCACAGGAGUGGCAGCACCAUUGGAAAAGCAUUUGAAGCCACUGUGAGAGGUGCCAGGAGAAUGGCAGUCUUGGGAGACACAGCCUGGGACUUUGGAUCAGUU
KX377336    GAUGUUGGAACUUGACCCACCAUUUGGGGAUUCUUACAUUGUCAUAGGAGUUGGGGAUAAGAAGAUCACCCACCACUGGCACAGGAGUGGCAGCACCAUCGGAAAAGCAUUUGAAGCCACUGUGAGAGGCGCCAAGAGAAUGGCAGUCUUGGGAGACACAGCCUGGGACUUUGGAUCAGUC
KX377337    GAUGCUGGAACUUGAUCCACCAUUUGGGGACUCUUACAUUGUCAUAGGAGUCGGGGAGAAGAAGAUCACCCACCACUGGCACAGGAGUGGCAGCACCAUUGGAAAAGCAUUUGAAGCCACUGUGAGAGGUGCCAAGAGAAUGGCAGUCUUGGGAGACACAGCCUGGGACUUUGGAUCAGUU
KX369547    GAUGCUGGAACUUGAUCCACCAUUUGGGGACUCUUACAUUGUCAUAGGAGUCGGGGAGAAGAAGAUCACCCACCACUGGCACAGGAGUGGCAGCACCAUUGGAAAAGCAUUUGAAGCCACUGUGAGAGGUGCCAAGAGAAUGGCAGUCUUGGGAGACACAGCCUGGGACUUUGGAUCAGUU
KX548902    GAUGCUGGAACUUGAUCCACCAUUUGGGGACUCUUACAUUGUCAUAGGAGUCGGGGAGAAGAAGAUCACCCACCACUGGCACAGGAGUGGCAGCACCAUUGGAAAAGCAUUUGAAGCCACUGUGAGAGGUGCCAAGAGAAUGGCAGUCUUGGGAGACACAGCCUGGGACUUUGGAUCAGUU
KX601166    GAUGUUGGAGCUCGACCCACCAUUUGGGGAUUCUUACAUUGUCAUAGGAGUCGGGGACAAGAAAAUCACCCAUCACUGGCAUCGGAGUGGUAGCACCAUCGGAAAGGCAUUUGAAGCCACUGUGAGAGGUGCCAAGAGAAUGGCAGUCUUGGGGGACACAGCCUGGGACUUUGGAUCAGUU
KX601167    GAUGUUGGAACUUGACCCACCAUUUGGGGAUUCUUACAUUGUCAUAGGAGUUGGGGAUAAGAAGAUCACCCACCACUGGUACAGGAGUGGCAGCACCAUCGGAAAAGCAUUUGAAGCCACUGUGAGAGGCGCCAAGAGAAUGGCAGUCUUGGGAGACACAGCCUGGGACUUUGGAUCAGUC
KX601168    GAUGCUGGAACUUGAUCCACCAUUUGGGGACUCUUACAUUGUCAUAGGAGUCGGGGAGAAGAAGAUCACCCACCACUGGCACAGGAGUGGCAGCACCAUUGGAAAAGCAUUUGAAGCCACUGUGAGAGGUGCCAAGAGAAUGGCAGUCUUGGGAGACACAGCCUGGGACUUUGGAUCAGUU
KX766028    GAUGCUGGAACUUGAUCCACCAUUUGGGGACUCUUACAUUGUCAUAGGAGUCGGGGAGAAGAAGAUCACCCACCACUGGCACAGGAGUGGCAGCACCAUUGGAAAAGCAUUUGAAGCCACUGUGAGAGGUGCCAAGAGAAUGGCAGUCUUGGGAGACACAGCCUGGGACUUUGGAUCAGUU
KX766029    GAUGCUGGAACUUGAUCCACCAUUUGGGGACUCUUACAUUGUCAUAGGAGUCGGGGAGAAGAAGAUCACCCACCACUGGCACAGGAGUGGCAGCACCAUUGGAAAAGCAUUUGAAGCCACUGUGAGAGGUGCCAAGAGAAUGGCAGUCUUGGGAGACACAGCCUGGGACUUUGGAUCAGUU
KU940224    GAUGCUGGAACUUGAUCCACCAUUUGGGGACUCCUACAUUGUCAUAGGAGUCGGGGAGAAGAAGAUCACCCACCACUGGCACAGGAGUGGCAGCACCAUUGGAAAAGCAUUUGAAGCCACUGUGAGAGGUGCCAAGAGAAUGGCAGUCUUGGGAGACACAGCCUGGGACUUUGGAUCAGUU


            2222222222222222222222222222222222222222222222222222222222222222222222222222222222222222222222222222222222222222222222222222222222222222222222222222222222222222222222222222222222222
            1111111111111111111111111112222222222222222222222222222222222222222222222222222222222222222222222222222222222222222222222222222223333333333333333333333333333333333333333333333333333333
            7777777888888888899999999990000000000111111111122222222223333333333444444444455555555556666666666777777777788888888889999999999000000000011111111112222222222333333333344444444445555
Sequence    3456789012345678901234567890123456789012345678901234567890123456789012345678901234567890123456789012345678901234567890123456789012345678901234567890123456789012345678901234567890123
-----------------------------------------------------------------------------------------------------------------------------------------------------------------------------------------------------------
NC_012532   GGGGGUGUGUUCAACUCACUGGGUAAGGGCAUUCACCAGAUUUUUGGAGCAGCCUUCAAAUCACUGUUUGGAGGAAUGUCCUGGUUCUCACAGAUCCUCAUAGGCACGCUGCUAGUGUGGUUAGGUUUGAACACAAAGAAUGGAUCUAUCUCCCUCACAUGCUUGGCCCUGGGGGGAGUGA
KU922960    GGAGGCGCUCUCAACUCAUUGGGCAAGGGCAUCCAUCAAAUUUUUGGAGCAGCUUUCAAAUCAUUGUUUGGAGGAAUGUCCUGGUUCUCACAAAUUCUCAUUGGAACGUUGCUGAUGUGGUUGGGUCUGAACACAAAGAAUGGAUCUAUUUCCCUUAUGUGCUUGGCCUUAGGGGGAGUGU
KU922923    GGAGGCGCUCUCAACUCAUUGGGCAAGGGCAUCCAUCAAAUUUUUGGAGCAGCUUUCAAAUCAUUGUUUGGAGGAAUGUCCUGGUUCUCACAAAUUCUCAUUGGAACGUUGCUGAUGUGGUUGGGUCUGAACACAAAGAAUGGAUCUAUUUCCCUUAUGUGCUUGGCCUUAGGGGGAGUGU
KU820898    GGAGGCGCUCUCAACUCAUUGGGCAAGGGCAUCCAUCAAAUUUUUGGAGCAGCUUUCAAAUCAUUGUUUGGAGGAAUGUCCUGGUUCUCACAAAUUCUCAUUGGAACGUUGCUGAUGUGGUUGGGUCUGAACACAAAGAAUGGAUCUAUUUCCCUUAUGUGCUUGGCCUUAGGGGGAGUGU
KU321639    GGAGGCGCUCUCAACUCAUUGGGCAAGGGCAUCCAUCAAAUUUUUGGAGCAGCUUUCAAAUCAUUGUUUGGAGGAAUGUCCUGGUUCUCACAAAUUCUCAUUGGAACGUUGCUGAUGUGGUUGGGUCUGAACACAAAGAAUGGAUCUAUUUCCCUUAUGUGCUUGGCCUUAGGGGGAGUGU
KU820899    GGAGGCGCUCUCAACUCAUUGGGCAAGGGCAUCCAUCAAAUUUUUGGAGCAGCUUUCAAAUCAUUGUUUGGAGGAAUGUCCUGGUUCUCACAAAUUCUCAUUGGAACGUUGCUGAUGUGGUUGGGUCUGAACACAAAGAAUGGAUCUAUUUCCCUUAUGUGCUUGGCCUUAGGGGGAGUGU
KU853012    GGAGGCGCUCUCAACUCAUUGGGCAAGGGCAUCCAUCAAAUUUUUGGAGCAGCUUUCAAAUCAUUGUUUGGAGGAAUGUCCUGGUUCUCACAAAUCCUCAUUGGAACGUUGCUGAUGUGGUUGGGUCUGAACACAAAGAAUGGAUCUAUUUCCCUCAUGUGCUUGGCCUUAGGGGGAGUGU
KU729217    GGAGGCGCUCUCAACUCAUUGGGCAAGGGCAUCCAUCAAAUUUUUGGAGCAGCUUUCAAAUCAUUGUUUGGAGGAAUGUCCUGGUUCUCACAAAUCCUCAUUGGAACGUUGCUGAUGUGGUUGGGUCUGAACACAAAGAAUGGAUCUAUUUCCCUUAUGUGCUUGGCCUUAGGGGGAGUGU
KU729218    GGAGGCGCUCUCAACUCAUUGGGCAAGGGCAUCCAUCAAAUUUUUGGAGCAGCUUUCAAAUCAUUGUUUGGAGGAAUGUCCUGGUUCUCACAAAUUCUCAUUGGAACGUUGCUGAUGUGGUUGGGUCUGAACACAAAGAAUGGAUCUAUUUCCCUUAUGUGCUUGGCCUUAGGGGGAGUGU
KU761564    GGAGGCGCUCUCAACUCAUUGGGCAAGGGCAUCCAUCAAAUUUUUGGAGCAGCUUUCAAAUCAUUGUUUGGAGGAAUGUCCUGGUUCUCACAAAUUCUCAUUGGAACGUUGCUGAUGUGGUUGGGUCUGAACACAAAGAAUGGAUCUAUUUCCCUUAUGUGCUUGGCCUUAGGGGGAGUGU
KU740184    GGAGGCGCUCUCAACUCAUUGGGCAAGGGCAUCCAUCAAAUUUUUGGAGCAGCUUUCAAAUCAUUGUUUGGAGGAAUGUCCUGGUUCUCACAAAUUCUCAUUGGAACGUUGCUGAUGUGGUUGGGUCUGAACACAAAGAAUGGAUCUAUUUCCCUUAUGUGCUUGGCCUUAGGGGGAGUGU
KU744693    GGAGGCGCUCUCAACUCAUUGGGCAAGGGCAUCCAUCAAAUUAUUGGAGCAGCUUUCAAAUCAUUGUUUGGAGGAAUGUCCUGGUUCUCACAAAUUCUCAUUGGGACGUUGCUGAUGUGGUUGGGUCUGAACACAAAGAAUGGAUCUAUUUCCCUUAUGUGCUUGGCCUUAGGGGGAGUGU
KU497555    GGAGGCGCUCUCAACUCAUUGGGCAAGGGCAUCCAUCAAAUUUUUGGAGCAGCUUUCAAAUCAUUGUUUGGAGGAAUGUCCUGGUUCUCACAAAUUCUCAUUGGAACGUUGCUGAUGUGGUUGGGUCUGAACACAAAGAAUGGAUCUAUUUCCCUUAUGUGCUUGGCCUUAGGGGGAGUGU
KU527068    GGAGGCGCUCUCAACUCAUUGGGCAAGGGCAUCCAUCAAAUUUUUGGAGCAGCUUUCAAAUCAUUGUUUGGAGGAAUGUCCUGGUUCUCACAAAUCCUCAUUGGAACGUUGCUGAUGUGGUUGGGUCUGAACACAAAGAAUGGAUCUAUUUCCCUUAUGUGCUUGGCCUUAGGGGGAGUGU
KU501217    GGAGGCGCUCUCAACUCAUUGGGCAAGGGCAUCCAUCAAAUUUUUGGAGCUGCUUUCAAAUCAUUGUUUGGAGGAAUGUCCUGGUUCUCACAAAUUCUCAUUGGAACGUUGCUGAUGUGGUUGGGUCUGAACACAAAGAAUGGAUCUAUUUCCCUUAUGUGCUUGGCCUUAGGGGGAGUGU
KU501216    GGAGGCGCUCUCAACUCAUUGGGCAAGGGCAUCCAUCAAAUUUUUGGAGCUGCUUUCAAAUCAUUGUUUGGAGGAAUGUCCUGGUUCUCACAAAUUCUCAUUGGAACGUUGCUGAUGUGGUUGGGUCUGAACACAAAGAAUGGAUCUAUUUCCCUUAUGUGCUUGGCCUUAGGGGGAGUGU
KU501215    GGAGGCGCUCUCAACUCAUUGGGCAAGGGCAUCCAUCAAAUUUUUGGAGCAGCUUUCAAAUCAUUGUUUGGAGGAAUGUCCUGGUUCUCACAAAUUCUCAUUGGAACGUUGCUGAUGUGGUUGGGUCUGAACACAAAGAAUGGAUCUAUUUCCCUUAUGUGCUUGGCCUUAGGGGGAGUGU
KU365780    GGAGGCGCUCUCAACUCAUUGGGCAAGGGCAUCCAUCAAAUUUUUGGAGCAGCUUUCAAAUCAUUGUUUGGAGGAAUGUCCUGGUUCUCACAAAUUCUCAUUGGAACGUUGCUGAUGUGGUUGGGUCUGAACACAAAGAAUGGAUCUAUUUCCCUUAUGUGCUUGGCCUUAGGGGGAGUGU
KU365779    GGAGGCGCUCUCAACUCAUUGGGCAAGGGCAUCCAUCAAAUUUUUGGAGCAGCUUUCAAAUCAUUGUUUGGAGGAAUGUCCUGGUUCUCACAAAUUCUCAUUGGAACGUUGCUGAUGUGGUUGGGUCUGAACACAAAGAAUGGAUCUAUUUCCCUUAUGUGCUUGGCCUUAGGGGGAGUGU
```

```
KU707826    GGAGGCGCUCUCAACUCAUUGGGCAAGGGCAUCCAUCAAAUUUUUGGAGCAGCUUUCAAAUCAUUGUUUGGAGGAAUGUCCUGGUUCUCACAAAUUCUCAUUGGAACGUUGCUGAUGUGGUUGGGUCUGAACACAAAGAAUGGAUCUAUUUCCCUUAUGUGCUUGGCCUUAGGGGGAGUGU
KU365778    GGAGGCGCUCUCAACUCAUUGGGCAAGGGCAUCCAUCAAAUUUUUGGAGCAGCUUUCAAAUCAUUGUUUGGAGGAAUGUCCUGGUUCUCACAAAUUCUCAUUGGAACGUUGCUGAUGUGGUUGGGUCUGAACACAAAGAAUGGAUCUAUUUCCCUUAUGUGCUUGGCCUUAGGGGGGGUGU
KU365777    GGAGGCGCUCUCAACUCAUUGGGCAAGGGCAUCCAUCAAAUUUUUGGAGCAGCUUUCAAAUCAUUGUUUGGAGGAAUGUCCUGGUUCUCACAAAUUCUCAUUGGAACGUUGCUGAUGUGGUUGGGUCUGAACACAAAGAAUGGAUCUAUUUCCCUUAUGUGCUUGGCCUUAGGGGGAGUGU
KU312312    GGAGGCGCUCUCAACUCAUUGGGCAAGGGCAUCCAUCAAAUCUUUGGAGCAGCUUUCAAAUCAUUGUUUGGAGGAAUGUCCUGGUUCUCACAAAUUCUCAUUGGAACGUUGCUGAUGUGGUUGGGUCUGAACGCAAAGAAUGGAUCUAUUUCCCUUAUGUGCUUGGCCUUAGGGGGAGUGU
KF268950    GGGGGCGUGUUCAACUCAUUGGGCAAGGGUGUUCACCAGAUUUUUGGAGCAGCUUUCAAAUCACUGUUUGGAGGAAUGUCCUGGUUCUCACAGAUCCUCAUAGGCACGCUGCUGGUGUGGUUGGGUUUGAACACAAAGAAUGGAUCCAUCUCCCUCACAUGCUUGGCCCUGGGGGGAGUGA
KF268949    GGGGGCGUGUUUAACUCAUUGGGCAAGGGCAUCCACCAGAUUUUUGGAGCAGCCUUCAAAUCACUGUUUGGAGGAAUGUCCUGGUUCUCACAGAUCCUCAUAGGCACGCUGCUGGUGUGGUUGGGUUUGAACACAAAGAAUGGAUCCAUCUCCCUCACAUGCUUGGCCCUGGGGGGAGUGA
KF268948    GGGGGCGUGUUCAACUCAUUGGGCAAGGGUGUUCACCAGAUUUUUGGAGCAGCUUUCAAAUCACUGUUUGGAGGAAUGUCCUGGUUCUCACAGAUCCUCAUAGGCACGCUGCUGGUGUGGUUGGGUUUGAACACAAAGAAUGGAUCCAUCUCCCUCACAUGCUUGGCCCUGGGGGGAGUGA
KJ776791    GGAGGCGCUCUCAACUCAUUGGGCAAGGGCAUCCAUCAAAUUUUUGGAGCAGCUUUCAAAUCAUUGUUUGGAGGAAUGUCCUGGUUCUCACAAAUUCUCAUUGGAACGUUGCUGAUGUGGUUGGGUCUGAACACAAAGAAUGGAUCUAUUUCCCUUAUGUGCUUGGCCUUAGGGGGAGUGU
KF383119    GGGGGUGUGUUCAACUCACUGGGUAAGGGCAUUCACCAGAUUUUUGGAGCAGCCUUCAAAUCACUGUUUGGAGGAAUGUCCUGGUUCUCACAGAUCCUCAUAGGCACGCUGCUAGUGUGGUUAGGUUUGAACACAAAGAAUGGAUCUAUCUCCCUCACAUGCUUGGCCCUGGGGGGAGUGA
KF383118    GGGGGUGUGUUCAACUCACUGGGUAAGGGCAUUCACCAGAUUUUUGGAGCAGCCUUCAAAUCACUGUUUGGAGGAAUGUCCUGGUUCUCACAGAUCCUCAUAGGCACGCUGCUAGUGUGGUUAGGUUUGAACACAAAGAAUGGAUCUAUCUCCCUCACAUGCUUGGCCCUGGGGGGAGUGA
KF383117    GGAGGUGUGUUUAAUUCAUUGGGUAAGGGUGUUCAUCAGAUCUUUGGAGCAGCUUUCAAAUCACUGUUUGGAGGAAUGUCCUGGUUCUCACAGAUCCUCAUAGGCACACUGUUGGUGUGGUUGGGUCUGAACACAAAGAAUGGAUCUAUCUCCCUCACAUGCUUAGCCCUGGGGGGAGUGA
KF383116    GGGGGUGUGUUUAAUUCAUUGGGUAAGGGUAUUCACCAGAUCUUUGGAGCAGCUUUUAAAUCACUGUUUGGAGGAAUGUCCUGGUUCUCACAGAUCCUCAUAGGCACACUGUUGGUGUGGUUAGGUCUGAACACAAAGAAUGGAUCUAUCUCCCUCACAUGCUUAGCCCUGGGGGGAGUGA
KF383115    GGGGGUGUGUUCAACUCAUUGGGCAAGGGCAUCCACCAGAUCUUUGGAGCAGCCUUCAAAUCACUGUUUGGAGGAAUGUCCUGGUUCUCACAGAUCCUCAUAGGCACGCUGCUAGUGUGGUUGGGCUUGAACACAAAGAAUGGAUCCAUCUCCCUCACAUGCUUGGCCCUGGGGGGAGUGA
EU545988    GGAGGUGCUCUCAACUCAUUGGGCAAGGGCAUCCAUCAAAUUUUUGGAGCAGCUUUCAAAUCAUUGUUUGGAGGAAUGUCCUGGUUCUCACAAAUUCUCAUUGGAACGUUGCUGGUGUGGUUGGGUCUGAAUACAAAGAAUGGAUCUAUUUCCCUUACGUGCUUGGCCUUAGGGGGAGUGU
KU955589    GGAGGCGCUCUCAACUCAUUGGGCAAGGGCAUCCAUCAAAUUUUUGGAGCAGCUUUCAAAUCAUUGUUUGGAGGAAUGUCCUGGUUCUCACAAAUUCUCAUUGGAACGUUGCUGAUGUGGUUGGGUCUGAACACAAAGAAUGGAUCUAUUUCCCUUAUGUGCUUGGCCUUAGGGGGAGUGU
KU955590    GGAGGCGCUCUCAACUCAUUGGGCAAGGGCAUCCAUCAAAUUUUUGGAGCAGCUUUCAAAUCAUUGUUUGGAGGAAUGUCCUGGUUCUCACAAAUUCUCAUUGGAACGUUGCUGAUGUGGUUGGGUCUGAACACAAAGAAUGGAUCUAUUUCCCUUAUGUGCUUGGCCUUAGGGGGAGUGU
KU940228    GGAGGCGCUCUCAACUCAUUGGGCAAGGGCAUCCAUCAAAUUUUUGGAGCAGCUUUCAAAUCAUUGUUUGGAGGAAUGUCCUGGUUCUCACAAAUUCUCAUUGGAACGUUGCUGAUGUGGUUGGGUCUGAACACAAAGAAUGGAUCUAUUUCCCUUAUGUGCUUGGCCUUAGGGGGAGUGU
KU991811    GGAGGCGCUCUCAACUCAUUGGGCAAGGGCAUCCAUCAAAUUUUUGGAGCAGCUUUCAAAUCAUUGUUUGGAGGAAUGUCCUGGUUCUCACAAAUUCUCAUUGGAACGUUGCUGAUGUGGUUGGGUCUGAACACAAAGAAUGGAUCUAUUUCCCUUAUGUGCUUGGCCUUAGGGGGAGUGU
KU509998    GGAGGCGCUCUCAACUCAUUGGGCAAGGGCAUCCAUCAAAUUUUUGGAGCAGCUUUCAAAUCAUUGUUUGGAGGAAUGUCCUGGUUCUCACAAAUUCUCAUUGGAACGUUGCUGAUGUGGUUGGGUCUGAACACAAAGAAUGGAUCUAUUUCCCUUAUGUGCUUGGCCUUAGGGGGAGUGU
KX056898    GGAGGCGCUCUCAACUCAUUGGGCAAGGGCAUCCAUCAAAUUUUUGGAGCAGCUUUCAAAUCAUUGUUUGGAGGAAUGUCCUGGUUCUCACAAAUUCUCAUUGGAACGUUGCUGAUGUGGUUGGGUCUGAACACAAAGAAUGGAUCUAUUUCCCUUAUGUGCUUGGCCUUAGGGGGAGUGU
KX117076    GGAGGCGCUCUCAACUCAUUGGGCAAGGGCAUCCAUCAAAUUUUUGGAGCAGCUUUCAAAUCAUUGUUUGGAGGAAUGUCCUGGUUCUCACAAAUUCUCAUUGGAACGUUGCUGAUGUGGUUGGGUCUGAACACAAAGAAUGGAUCUAUUUCCCUUAUGUGCUUGGCCUUAGGGGGAGUGU
KX185891    GGAGGCGCUCUCAACUCAUUGGGCAAGGGCAUCCAUCAAAUUUUUGGAGCAGCUUUCAAAUCAUUGUUUGGAGGAAUGUCCUGGUUCUCACAAAUUCUCAUUGGAACGUUGCUGAUGUGGUUGGGUCUGAACACAAAGAAUGGAUCUAUUUCCCUUAUGUGCUUGGCCUUAGGGGGAGUGU
KX247646    GGAGGCGCUCUCAACUCAUUGGGCAAGGGCAUCCAUCAAAUUUUUGGAGCAGCUUUCAAAUCAUUGUUUGGAGGAAUGUCCUGGUUCUCACAAAUUCUCAUUGGAACGUUGCUGAUGUGGUUGGGUCUGAACACAAAGAAUGGAUCUAUUUCCCUUAUGUGCUUGGCCUUAGGGGGAGUGU
KX262887    GGAGGCGCUCUCAACUCAUUGGGCAAGGGCAUCCAUCAAAUUUUUGGAGCAGCUUUCAAAUCAUUGUUUGGAGGAAUGUCCUGGUUCUCACAAAUUCUCAUUGGAACGUUGCUGAUGUGGUUGGGUCUGAACACAAAGAAUGGAUCUAUUUCCCUUAUGUGCUUGGCCUUAGGGGGAGUGU
KX280026    GGAGGCGCUCUCAACUCAUUGGGCAAGGGCAUCCAUCAAAUUUUUGGAGCAGCUUUCAAAUCAUUGUUUGGAGGAAUGUCCUGGUUCUCACAAAUUCUCAUUGGAACGUUGCUGAUGUGGUUGGGUCUGAACACAAAGAAUGGAUCUAUUUCCCUUAUGUGCUUGGCCUUAGGGGGAGUGU
KU758877    GGAGGCGCUCUCAACUCAUUGGGCAAGGGCAUCCAUCAAAUUUUUGGAGCAGCUUUCAAAUCAUUGUUUGGAGGAAUGUCCUGGUUCUCACAAAUUCUCAUUGGAACGUUGCUGAUGUGGUUGGGUCUGAACACAAAGAAUGGAUCUAUUUCCCUUAUGUGCUUGGCCUUAGGGGGAGUGU
KU866423    GGAGGCGCUCUCAACUCAUUGGGCAAGGGCAUCCAUCAAAUUUUUGGAGCAGCUUUCAAAUCAUUGUUUGGAGGAAUGUCCUGGUUCUCACAAAUUCUCAUUGGAACGUUGCUGAUGUGGUUGGGUCUGAACACAAAGAAUGGAUCUAUUUCCCUUAUGUGCUUGGCCUUAGGGGGAGUGU
KX377336    GGAGGUGCUCUCAACUCAUUGGGCAAGGGCAUCCAUCAAAUUUUUGGAGCAGCUUUCAAAUCAUUGUUUGGAGGAAUGUCCUGGUUCUCACAAAUCCUCAUAGGAACGUUGCUGGUGUGGUUGGGUCUGAACACAAAGAAUGGAUCUAUUUCCCUUACGUGCUUGGCCUUAGGGGGAGUGU
KX377337    GGAGGCGCUCUCAACUCAUUGGGCAAGGGCAUCCAUCAAAUUUUUGGAGCAGCUUUCAAAUCAUUGUUUGGAGGAAUGUCCUGGUUCUCACAAAUUCUCAUUGGAACGUUGCUGAUGUGGUUGGGUCUGAACACAAAGAAUGGAUCUAUUUCCCUUAUGUGCUUGGCCUUAGGGGGAGUGU
KX369547    GGAGGCGCUCUCAACUCAUUGGGCAAGGGCAUCCAUCAAAUUUUUGGAGCAGCUUUCAAAUCAUUGUUUGGAGGAAUGUCCUGGUUCUCACAAAUUCUCAUUGGAACGUUGCUGAUGUGGUUGGGUCUGAACACAAAGAAUGGAUCUAUUUCCCUUAUGUGCUUGGCCUUAGGGGGAGUGU
KX548902    GGAGGCGCUCUUAACUCAUUGGGCAAGGGCAUCCAUCAAAUUUUUGGAGCAGCUUUCAAAUCAUUGUUUGGAGGAAUGUCCUGGUUCUCACAAAUUCUUAUUGGAACGUUGCUGAUGUGGUUGGGUCUGAACACAAAGAAUGGAUCUAUUUCCCUUAUGUGCUUGGCCUUGGGGGGAGUGU
KX601166    GGGGGUGUGUUUAAUUCAUUGGGUAAGGGUAUUCACCAGAUCUUUGGAGCAGCUUUCAAAUCACUGUUUGGAGGAAUGUCCUGGUUCUCACAGAUCCUCAUAGGCACACUGUUGGUGUGGUUAGGUUUGAACACAAAGAAUGGAUCUAUCUCCCUCACAUGCUUAGCCCUGGGGGGAGUGA
KX601167    GGAGGUGCUCUCAACUCAUUGGGCAAGGGCAUCCAUCAAAUUUUUGGAGCAGCUUUCAAAUCAUUGUUUGGAGGAAUGUCCUGGUUCUCACAAAUCCUCAUAGGAACGUUGCUGGUGUGGUUGGGUCUGAACACAAAGAAUGGAUCUAUUUCCCUUACGUGCUUGGCCUUAGGGGGAGUGU
KX601168    GGAGGCGCUCUCAACUCAUUGGGCAAGGGCAUCCAUCAAAUUUUUGGAGCAGCUUUCAAAUCAUUGUUUGGAGGAAUGUCCUGGUUCUCACAAAUUCUCAUUGGAACGUUGCUGAUGUGGUUGGGUCUGAACACAAAGAAUGGAUCUAUUUCCCUUAUGUGCUUGGCCUUAGGGGGAGUGU
KX766028    GGAGGCGCUCUCAACUCAUUGGGCAAGGGCAUCCAUCAAAUUUUUGGAGCAGCUUUCAAAUCAUUGUUUGGAGGAAUGUCCUGGUUCUCACAAAUUCUCAUUGGAACGUUGCUGAUGUGGUUGGGUCUGAACACAAAGAAUGGAUCUAUUUCCCUUAUGUGCUUGGCCUUAGGGGGAGUGU
KX766029    GGAGGCGCUCUCAACUCAUUGGGCAAGGGCAUCCAUCAAAUUUUUGGAGCAGCUUUCAAAUCAUUGUUUGGAGGAAUGUCCUGGUUCUCACAAAUUCUCAUUGGAACGUUGCUGAUGUGGUUGGGUCUGAACACAAAGAAUGGAUCUAUUUCCCUUAUGUGCUUGGCCUUAGGGGGAGUGU
KU940224    GGAGGCGCUCUCAACUCAUUGGGCAAGGGCAUCCAUCAAAUUUUUGGAGCAGCUUUCAAAUCAUUGUUUGGAGGAAUGUCCUGGUUCUCACAAAUUCUCAUUGGAACGUUGCUGAUGUGGUUGGGUCUGAACACAAAGAAUGGAUCUAUUUCCCUUAUGUGCUUGGCCUUAGGGGGAGUGU


            2222222222222222222222222222222222222222222222222222222222222222222222222222222222222222222222222222222222222222222222222222222222222222222222222222222222222222222222222222222222222
            3333333333333333333333333333333333333333333333444444444444444444444444444444444444444444444444444444444444444444444444444444444444444444444444444444445555555555555555555555555555555555
            5555556666666666777777777788888888889999999999000000000011111111112222222222333333333344444444445555555555666666666677777777778888888888999999999900000000001111111111222222222233333
Sequence    4567890123456789012345678901234567890123456789012345678901234567890123456789012345678901234567890123456789012345678901234567890123456789012345678901234567890123456789012345678901234
--------------------------------------------------------------------------------------------------------------------------------------------------------------------------------------------------------
NC_012532   UGAUCUUCCUCUCCACGGCUGUUUCUGCUGACGUGGGGUGCUCAGUGGACUUCUCAAAAAAGGAAACGAGAUGUGGCACGGGGGUAUUCAUCUAUAAUGAUGUUGAAGCCUGGAGGGACCGGUACAAGUACCAUCCUGACUCCCCCCGCAGAUUGGCAGCAGCAGUCAAGCAGGCCUGGGA
KU922960    UGAUCUUCUUAUCCACAGCCGUCUCUGCUGAUGUGGGGUGCUCGGUGGACUUCUCAAAGAAGGAGACGAGAUGUGGUACAGGGGUGUUCGUCUAUAACGACGUUGAAGCCUGGAGGGACAGGUACAAGUACCAUCCUGACUCCCCCCGUAGAUUGGCAGCAGCAGUCAAGCAAGCCUGGGA
KU922923    UGAUCUUCUUAUCCACAGCCGUCUCUGCUGAUGUGGGGUGCUCGGUGGACUUCUCAAAGAAGGAGACGAGAUGUGGUACAGGGGUGUUCGUCUAUAACGACGUUGAAGCCUGGAGGGACAGGUACAAGUACCAUCCUGACUCCCCCCGUAGAUUGGCAGCAGCAGUCAAGCAAGCCUGGGA
KU820898    UGAUCUUCUUAUCCACAGCCGUCUCUGCUGAUGUGGGGUGCUCGGUGGACUUCUCAAAGAAGGAGACGAGAUGCGGUACAGGGGUGUUUGUCUAUAACGACGUUGAAGCCUGGAGGGACAGGUACAAGUACCAUCCUGACUCCCCCCGUAGAUUGGCAGCAGCAGUCAAGCAAGCCUGGGA
KU321639    UGAUCUUCUUAUCCACAGCCGUCUCUGCUGAUGUGGGGUGCUCGGUGGACUUCUCAAAGAAGGAGACGAGAUGCGGUACAGGGGUGUUCGUCUAUAACGACGUUGAAGCCUGGAGGGACAGGUACAAGUACCAUCCUGACUCCCCCCGUAGAUUGGCAGCAGCAGUCAAGCAAGCCUGGGA
KU820899    UGAUCUUCUUAUCCACAGCCGUCUCUGCUGAUGUGGGGUGCUCGGUGGACUUCUCAAAGAAGGAGACGAGAUGCGGUACAGGGGUGUUCGUCUAUAACGACGUUGAAGCCUGGAGGGACAGGUACAAGUACCAUCCUGACUCCCCCCGUAGAUUGGCAGCAGCAGUCAAGCAAGCCUGGGA
KU853012    UGAUCUUCUUAUCCACAGCCGUCUCUGCUGAUGUGGGGUGCUCGGUGGACUUCUCAAAGAAGGAGACGAGAUGCGGUACAGGGGUGUUCGUCUAUAACGACGUUGAAGCCUGGAGGGACAGGUACAAGUACCAUCCUGACUCCCCCCGUAGAUUGGCAGCAGCAGUCAAGCAAGCCUGGGA
KU729217    UGAUCUUCUUAUCCACAGCCGUCUCUGCUGAUGUGGGGUGCUCGGUGGACUUCUCAAAGAAGGAGACGAGAUGCGGUACAGGGGUGUUCGUCUAUAACGACGUUGAAGCCUGGAGGGACAGGUACAAGUACCAUCCUGACUCCCCCCGUAGAUUGGCAGCAGCAGUCAAGCAAGCCUGGGA
KU729218    UGAUCUUCUUAUCCACAGCCGUCUCUGCUGAUGUGGGGUGCUCGGUGGACUUCUCAAAGAAGGAGACGAGAUGCGGUACAGGGGUGUUCGUCUAUAACGACGUUGAAGCCUGGAGGGACAGGUACAAGUACCAUCCUGACUCCCCCCGUAGAUUGGCAGCAGCAGUCAAGCAAGCCUGGGA
KU761564    UGAUCUUCUUAUCCACAGCCGUCUCUGCUGAUGUGGGGUGCUCGGUGGACUUCUCAAAGAAGGAGACGAGAUGCGGUACAGGGGUGUUUGUCUAUAACGACGUUGAAGCCUGGAGGGACAGGUACAAGUACCAUCCUGACUCCCCCCGUAGAUUGGCAGCAGCAGUCAAGCAAGCCUGGGA
KU740184    UGAUCUUCUUAUCCACAGCCGUCUCUGCUGAUGUGGGGUGCUCGGUGGACUUCUCAAAGAAGGAGACGAGAUGCGGUACAGGGGUGUUUGUCUAUAACGACGUUGAAGCCUGGAGGGACAGGUACAAGUACCAUCCUGACUCCCCCCGUAGAUUGGCAGCAGCAGUCAAGCAAGCCUGGGA
KU744693    UGAUCUUCUUAUCCACAGCCGUCUCAGGUGGUGUGGGGUGCUCGGUGGACUUCUCAAAGAAGGAGACGAGAUGCGGUACAGGGGUGUUCGUCUAUAACGAUGUUGAAGCCUGGAGGGACAGGUACAAGUACCAUCCUGACUCCCCCCGUAGAUUGGCAGCAGCAGUCAAGCAAGCCUGGGA
KU497555    UGAUCUUCUUAUCCACAGCCGUCUCUGCUGAUGUGGGGUGCUCGGUGGACUUCUCAAAGAAGGAGACGAGAUGUGGUACAGGGGUGUUCGUCUAUAACGACGUUGAAGCCUGGAGGGACAGGUACAAGUACCAUCCUGACUCUCCCCGUAGAUUGGCAGCAGCAGUCAAGCAAGCCUGGGA
KU527068    UGAUCUUCUUAUCCACAGCCGUCUCUGCUGAUGUGGGGUGCUCGGUGGACUUCUCAAAGAAGGAGACGAGAUGCGGUACAGGGGUGUUCGUCUAUAACGACGUUGAAGCCUGGAGGGACAGGUACAAGUACCAUCCUGACUCCCCCCGUAGAUUGGCAGCAGCAGUCAAGCAAGCCUGGGA
KU501217    UGAUCUUCUUAUCCACAGCCGUCUCUGCUGAUGUGGGGUGCUCGGUGGACUUCUCAAAGAAGGAGACGAGAUGCGGUACAGGGGUGUUCGUCUAUAACGACGUUGAAGCCUGGAGGGACAGGUACAAGUACCAUCCUGACUCCCCCCGUAGAUUGGCAGCAGCAGUCAAGCAAGCCUGGGA
KU501216    UGAUCUUCUUAUCCACAGCCGUCUCUGCUGAUGUGGGGUGCUCGGUGGACUUCUCAAAGAAGGAGACGAGAUGCGGUACAGGGGUGUUCGUCUAUAACGACGUUGAAGCCUGGAGGGACAGGUACAAGUACCAUCCUGACUCCCCCCGUAGAUUGGCAGCAGCAGUCAAGCAAGCCUGGGA
KU501215    UGAUCUUCUUAUCCACAGCCGUCUCUGCUGAUGUGGGGUGCUCGGUGGACUUCUCAAAGAAGGAGACGAGAUGCGGUACAGGGGUGUUCGUCUAUAACGACGUUGAAGCCUGGAGGGACAGGUACAAGUACCAUCCUGACUCCCCCCGUAGAUUGGCAGCAGCAGUCAAGCAAGCCUGGGA
KU365780    UGAUCUUCUUAUCCACAGCCGUCUCUGCUGAUGUGGGGUGCUCGGUGGACUUCUCAAAGAAGGAGACGAGAUGCGGUACAGGGGUGUUCGUCUAUAACGACGUUGAAGCCUGGAGGGACAGGUACAAGUACCAUCCUGACUCCCCCCGUAGAUUGGCAGCAGCAGUCAAGCAAGCCUGGGA
KU365779    UGAUCUUCUUAUCCACAGCCGUCUCUGCUGAUGUGGGGUGCUCGGUGGACUUCUCAAAGAAGGAGACGAGAUGCGGUACAGGGGUGUUCGUCUAUAACGACGUUGAAGCCUGGAGGGACAGGUACAAGUACCAUCCUGACUCCCCCCGUAGAUUGGCAGCAGCAGUCAAGCAAGCCUGGGA
KU707826    UGAUCUUCUUAUCCACAGCCGUCUCUGCUGAUGUGGGGUGCUCGGUGGACUUCUCAAAGAAGGAGACGAGAUGCGGUACAGGGGUGUUCGUCUAUAACGACGUUGAAGCCUGGAGGGACAGGUACAAGUACCAUCCUGACUCCCCCCGUAGAUUGGCAGCAGCAGUCAAGCAAGCCUGGGA
KU365778    UGAUCUUCUUAUCCACAGCCGUCUCUGCUGAUGUGGGGUGCUCGGUGGACUUCUCAAAGAAGGAGACGAGAUGCGGUACAGGGGUGUUCGUCUAUAACGACGUUGAAGCCUGGAGGGACAGGUACAAGUACCAUCCUGACUCCCCCCGUAGAUUGGCAGCAGCAGUCAAGCAAGCCUGGGA
KU365777    UGAUCUUCUUAUCCACAGCCGUCUCUGCUGAUGUGGGGUGCUCGGUGGACUUCUCAAAGAAGGAGACGAGAUGCGGUACAGGGGUGUUCGUCUAUAACGACGUUGAAGCCUGGAGGGACAGGUACAAGUACCAUCCUGACUCCCCCCGUAGAUUGGCAGCAGCAGUCAAGCAAGCCUGGGA
KU312312    UGAUCUUCUUAUCCACAGCCGUCUCUGCUGAUGUGGGGUGCUCGGUGGACUUCUCAAAGAAGGAGACGAGAUGCGGUACAGGGGUGUUCGUCUAUAACGACGUUGAAGCCUGGAGGGACAGGUACAAGUACCAUCCUGACUCCCCCCGUAGAUUGGCAGCAGCAGUCAAGCAAGCCUGGGA
KF268950    UGAUCUUCCUCUCCACGGCUGUCUCUGCUGACGUGGGGUGCUCGGUGGACUUCUCAAAAAGAGAAACGAGAUGUGGCACGGGGGUGUUCGUCUACAAUGAUGUUGAAGCCUGGAGGGACAGGUACAAGUACCAUCCUGACUCCCCCCGCAGAUUGGCAGCAGCAGUCAAGCAGGCUUGGGA
KF268949    UGAUCUUCCUCUCCACGGCUGUCUCUGCUGACGUGGGGUGCUCGGUGGACUUCUCAAAAAGAGAAACGAGAUGUGGCACGGGGGUGUUCGUCUACAAUGAUGUUGAAGCCUGGAGGGACAGGUACAAGUACCAUCCUGACUCCCCCCGCAGAUUGGCAGCAGCAGUCAAGCAGGCUUGGGA
KF268948    UGAUCUUCCUCUCCACGGCUGUCUCUGCUGACGUGGGGUGCUCGGUGGACUUCUCAAAAAGAGAAACGAGAUGUGGCACGGGGGUGUUCGUCUACAAUGAUGUUGAAGCCUGGAGGGACAGGUACAAGUACCAUCCUGACUCCCCCCGCAGAUUGGCAGCAGCAGUCAAGCAGGCUUGGGA
KJ776791    UGAUCUUCUUAUCCACAGCUGUCUCUGCUGAUGUGGGGUGCUCGGUGGACUUCUCAAAGAAGGAGACGAGAUGCGGUACAGGGGUGUUCGUCUAUAACGACGUUGAAGCCUGGAGGGACAGGUACAAGUACCAUCCUGACUCCCCCCGUAGAUUGGCAGCAGCAGUCAAGCAAGCCUGGGA
KF383119    UGAUCUUCCUCUCCACGGCUGUUUCUGCUGACGUGGGGUGCUCAGUGGACUUCUCAAAAAGAGAAACGAGAUGUGGCACGGGGGUAUUCAUCUAUAAUGAUGUUGAAGCCUGGAGGGACCGGUACAAGUACCAUCCUGACUCCCCCCGCAGAUUGGCAGCAGCAGUCAAGCAGGCCUGGGA
KF383118    UGAUCUUCCUCUCCACGGCUGUUUCUGCUGACGUGGGGUGCUCAGUGGACUUCUCAAAAAAGGAAACGAGAUGUGGCACGGGGGUAUUCAUCUAUAAUGAUGUUGAAGCCUGGAGGGACCGGUACAAGUACCAUCCUGACUCCCCCCGCAGAUUGGCAGCAGCAGUCAAGCAGGCCUGGGA
KF383117    UGAUUUUCCUUUCCACGGCUGUUUCUGCUGAUGUUGGGUGCUCGGUGGACUUCUCAAAAAAGGAAACGAGAUGUGGCACGGGGGUGUUCGUCUACAAUGACGUUGAAGCCUGGAGGGACCGUUACAAGUAUCAUCCUGACUCCCCCCGCAGAUUGGCAGCAGCUGUUAAGCAGGCUUGGGA
KF383116    UGAUCUUCCUUUCCACGGCUGUUUCUGCUGAUGUUGGGUGCUCGGUGGACUUCUCAAAAAAGGAAACGAGAUGUGGCACGGGGGUGUUCGUCUACAAUGACGUUGAAGCCUGGAGGGACCGGUACAAGUACCAUCCUGACUCCCCCCGCAGAUUGGCAGCAGCUGUCAAGCAGGCUUGGGA
KF383115    UGAUCUUCCUCUCCACGGCUGUUUCUGCUGAUGUGGGGUGCUCGGUGGACUUCUCAAAAAGAGAAACGAGAUGUGGCACGGGGGUGUUCGUCUACAAUGACGUUGAAGCCUGGAGGGAUAGGUACAAGUACCAUCCUGACUCCCCCCGCAGAUUGGCAGCAGCAGUCAAGCAGGCUUGGGA
EU545988    UGAUCUUUUUAUCCACAGCCGUCUCUGCUGAUGUGGGGUGCUCGGUGGACUUCUCAAAGAAGGAAACGAGAUGCGGUACGGGGGUGUUCGUCUAUAACGACGUUGAUGCCUGGAGGGACAGGUACAAGUACCAUCCUGACUCCCCUCGUAGAUUAGCAGCAGCAGUCAAGCAAGCCUGGGR
KU955589    UGAUCUUCUUAUCCACAGCCGUCUCUGCUGAUGUGGGGUGCUCGGUGGACUUCUCAAAGAAGGAGACGAGAUGCGGUACAGGGGUGUUCGUCUAUAACGACGUUGAAGCCUGGAGGGACAGGUACAAGUACCAUCCUGACUCCCCCCGUAGAUUGGCAGCAGCAGUCAAGCAAGCCUGGGA
```

```
KU955590    UGAUCUUCUUAUCCACAGCCGUCUCUGCUGAUGUGGGGUGCUCGGUGGACUUCUCAAAGAAGGAGACGAGAUGCGGUACAGGGGUGUUUGUCUAUAACGACGUUGAAGCCUGGAGGGACAGGUACAAGUACCAUCCUGACUCCCCCCGUAGAUUGGCAGCAGCAGUCAAGCAAGCCUGGGA
KU940228    UGAUCUUCUUAUCCACAGCCGUCUCUGCUGAUGUGGGGUGCUCGGUGGACUUCUCAAAGAAGGAGACGAGAUGCGGUACAGGGGUGUUCGUCUAUAACGACGUUGAAGCCUGGAGGGACAGGUACAAGUACCAUCCUGACUCCCCCCGUAGAUUGGCAGCAGCAGUCAAGCAAGCCUGGGA
KU991811    UGAUCUUCUUAUCCACAGCCGUCUCUGCUGAUGUGGGGUGUUCGGUGGACUUCUCAAAGAAGGAGACGAGAUGUGGUACAGGGGUGUUCGUCUAUAACGACGUUGAAGCCUGGAGGGACAGGUACAAGUACCAUCCUGACUCCCCCCGUAGAUUGGCAGCAGCAGUCAAGCAAGCCUGGGA
KU509998    UGAUCUUCUUAUCCACAGCCGUCUCUGCUGAUGUGGGGUGCUCGGUGGACUUCUCAAAGAAGGAGACGAGAUGCGGUACAGGGGUGUUCGUCUAUAACGACGUUGAAGCCUGGAGGGACAGGUACAAGUACCAUCCUGACUCCCCCCGUAGAUUGGCAGCAGCAGUCAAGCAAGCCUGGGA
KX056898    UGAUCUUCUUAUCCACAGCCGUCUCUGCUGAUGUGGGGUGCUCGGUGGACUUCUCAAAGAAGGAGACGAGAUGCGGUACAGGGGUGUUUGUCUAUAACGACGUUGAAGCCUGGAGGGACAGGUACAAGUACCAUCCUGACUCCCCCCGUAGAUUGGCAGCAGCAGUCAAGCAAGCCUGGGA
KX117076    UGAUCUUCUUAUCCACAGCCGUCUCUGCUGAUGUGGGGUGCUCGGUGGACUUCUCAAAGAAGGAGACGAGAUGCGGUACAGGGGUGUUCGUCUAUAACGACGUUGAAGCCUGGAGGGACAGGUACAAGUACCAUCCUGACUCCCCCCGUAGAUUGGCAGCAGCAGUCAAGCAAGCCUGGGA
KX185891    UGAUCUUCUUAUCCACAGCCGUCUCUGCUGAUGUGGGGUGCUCGGUGGACUUCUCAAAGAAGGAGACGAGAUGCGGUACAGGGGUGUUCGUCUAUAACGACGUUGAAGCCUGGAGGGACAGGUACAAGUACCAUCCUGACUCCCCCCGUAGAUUGGCAGCAGCAGUCAAGCAAGCCUGGGA
KX247646    UGAUCUUCUUAUCCACAGCCGUCUCUGCUGAUGUGGGGUGCUCGGUGGACUUCUCAAAGAAGGAGACGAGAUGUGGUACAGGGGUGUUCGUCUAUAACGACGUUGAAGCCUGGAGGGACAGGUACAAGUACCAUCCUGACUCCCCCCGUAGAUUGGCAGCAGCAGUCAAGCAAGCCUGGGA
KX262887    UGAUCUUCUUAUCCACAGCCGUCUCUGCUGAUGUGGGGUGCUCGGUGGACUUCUCAAAGAAGGAGACGAGAUGCGGUACAGGGGUGUUCGUCUAUAACGACGUUGAAGCCUGGAGGGACAGGUACAAGUACCAUCCUGACUCCCCCCGUAGAUUGGCAGCAGCAGUCAAGCAAGCCUGGGA
KX280026    UGAUCUUCUUAUCCACAGCCGUCUCUGCUGAUGUGGGGUGCUCGGUGGACUUCUCAAAGAAGGAGACGAGAUGCGGUACAGGGGUGUUCGUCUAUAACGACGUUGAAGCCUGGAGGGACAGGUACAAGUACCAUCCUGACUCCCCCCGUAGAUUGGCAGCAGCAGUCAAGCAAGCCUGGGA
KU758877    UGAUCUUCUUAUCCACAGCCGUCUCUGCUGAUGUGGGGUGCUCGGUGGACUUCUCAAAGAAGGAAACGAGAUGCGGUACAGGGGUGUUCGUCUAUAACGACGUUGAAGCCUGGAGGGACAGGUACAAGUACCAUCCUGACUCCCCCCGUAGAUUGGCAGCGGCAGUCAAGCAAGCCUGGGA
KU866423    UGAUCUUCUUAUCCACAGCCGUCUCUGCUGAUGUGGGGUGCUCGGUGGACUUCUCAAAGAAGGAGACGAGAUGCGGUACAGGGGUGUUCGUCUAUAACGACGUUGAAGCCUGGAGGGACAGGUACAAGUACCAUCCUGACUCCCCCCGUAGAUUGGCAGCAGCAGUCAAGCAAGCCUGGGA
KX377336    UGAUCUUCCUAUCUACAGCCGUCUCUGCUGAUGUGGGGUGUUCGGUGGACUUCUCAAAGAAGGAAACGAGAUGCGGUACGGGGGUGUUCGUCUAUAACGACGUUGAAGCCUGGAGGGACAGGUACAAGUACCAUCCUGACUCCCCUCGUAGAUUGGCAGCAGCAGUCAAGCAGGCCUGGGA
KX377337    UGAUCUUCUUAUCCACAGCCGUCUCUGCUGAUGUGGGGUGCUCGGUGGACUUCUCAAAGAAGGAGACGAGAUGCGGUACAGGGGUGUUCGUCUAUAACGACGUUGAAGCCUGGAGGGACAGGUACAAGUACCAUCCUGACUCCCCCCGUAGAUUGGCAGCAGCAGUCAAGCAAGCCUGGGA
KX369547    UGAUCUUCUUAUCCACAGCCGUCUCUGCUGAUGUGGGGUGCUCGGUGGACUUCUCAAAGAAGGAGACGAGAUGCGGUACAGGGGUGUUCGUCUAUAACGACGUUGAAGCCUGGAGGGACAGGUACAAGUACCAUCCUGACUCCCCCCGUAGAUUGGCAGCAGCAGUCAAGCAAGCCUGGGA
KX548902    UGAUCUUCUUAUCCACAGCCGUCUCUGCUGAUGUGGGGUGCUCGGUGGACUUCUCAAAGAAGGAGACGAGAUGUGGUACAGGGGUGUUCGUCUAUAACGACGUUGAAGCCUGGAGGGACAGGUACAAGUACCAUCCUGACUCCCCCCGUAGAUUGGCAGCAGCAGUCAAGCAAGCCUGGGA
KX601166    UGAUCUUCCUUUCCACGGCUGUUUCUGCUGAUGUUGGGUGCUCGGUGGACUUCUCAAAAAAGGAAACGAGAUGUGGCACGGGGGUGUUCGUCUACAAUGACGUUGAAGCCUGGAGGGACCGGUACAAGUACCAUCCUGACUCCCCCCGCAGAUUGGCAGCAGCUGUUAAGCAGGCUUGGGA
KX601167    UGAUCUUCCUAUCUACAGCCGUCUCUGCUGAUGUGGGGUGUUCGGUGGACUUCUCAAAGAAGGAAACGAGAUGCGGUACGGGGGUGUUCGUCUAUAACGACGUUGAAGCCUGGAGGGACAGGUACAAGUACCAUCCUGACUCCCCUCGUAGAUUGGCAGCAGCAGUCAAGCAGGCCUGGGA
KX601168    UGAUCUUCUUAUCCACAGCCGUCUCUGCUGAUGUGGGGUGCUCGGUGGACUUCUCAAAGAAGGAGACGAGAUGCGGUACAGGGGUGUUCGUCUAUAACGACGUUGAAGCCUGGAGGGACAGGUACAAGUACCAUCCUGACUCCCCCCGUAGAUUGGCAGCAGCAGUCAAGCAAGCCUGGGA
KX766028    UGAUCUUCUUAUCCACAGCCGUCUCUGCUGAUGUGGGGUGCUCGGUGGACUUCUCAAAGAAGGAGACGAGAUGCGGUACAGGGGUGUUUGUCUAUAACGACGUUGAAGCCUGGAGGGACAGGUACAAGUACCAUCCUGACUCCCCCCGUAGAUUGGCAGCAGCAGUCAAGCAAGCCUGGGA
KX766029    UGAUCUUCUUAUCCACAGCCGUCUCUGCUGAUGUGGGGUGCUCGGUGGACUUCUCAAAGAAGGAGACGAGAUGCGGUACAGGGGUGUUCGUCUAUAACGACGUUGAAGCCUGGAGGGACAGGUAUAAGUACCAUCCUGACUCCCCCCGUAGAUUGGCAGCAGCAGUCAAGCAAGCCUGGGA
KU940224    UGAUCUUCUUAUCCACAGCCGUCUCUGCUGAUGUGGGGUGCUCGGUGGACUUCUCAAAGAAGGAGACGAGAUGCGGUACAGGGGUGUUCGUCUAUAACGACGUUGAAGCCUGGAGGGACAGGUACAAGUACCAUCCUGACUCCCCCCGUAGAUUGGCAGCAGCAGUCAAGCAAGCCUGGGA


            2222222222222222222222222222222222222222222222222222222222222222222222222222222222222222222222222222222222222222222222222222222222222222222222222222222222222222222222222222222
            5555555555555555555555555555555555555555555555555555555555555556666666666666666666666666666666666666666666666666666666666666666666666666666666666666666666666666666667777777777777777
            333344444444445555555555666666666677777777778888888888999999999900000000001111111111222222222233333333334444444444555555555566666666667777777777888888888899999999990000000000111111
Sequence    5678901234567890123456789012345678901234567890123456789012345678901234567890123456789012345678901234567890123456789012345678901234567890123456789012345678901234567890123456789012345
-------------------------------------------------------------------------------------------------------------------------------------------------------------------------------------------------------
NC_012532   AGAGGGGAUCUGUGGGAUCUCAUCCGUUUCAAGAAUGGAAAACAUCAUGUGGAAAUCAGUAGAAGGGGAGCUCAAUGCUAUCCUAGAGGAGAAUGGAGUUCAACUGACAGUUGUUGUGGGAUCUGUAAAAAACCCCAUGUGGAGAGGUCCACAAAGAUUGCCAGUGCCUGUGAAUGAGCUG
KU922960    AGAUGGUAUCUGCGGGAUCUCCCUCUGUUUCAAGAAUGGAAAACAUCAUGUGGAGAUCAGUAGAAGGGGAGCUCAACGCAAUCCUGGAAGAGAAUGGAGUUCAACUGACGGUCGUUGUGGGAUCUGUAAAAAACCCCAUGUGGAGAGGUCCACAGAGAUUGCCCGUGCCUGUGAACGAGCUG
KU922923    AGAUGGUAUCUGCGGGAUCUCCCUCUGUUUCAAGAAUGGAAAACAUCAUGUGGAGAUCAGUAGAAGGGGAGCUCAACGCAAUCCUGGAAGAGAAUGGAGUUCAACUGACGGUCGUUGUGGGAUCUGUAAAAAACCCCAUGUGGAGAGGUCCACAGAGAUUGCCCGUGCCUGUGAACGAGCUG
KU820898    AGAUGGUAUCUGCGGGAUCUCCCUCUGUUUCAAGAAUGGAAAACAUCAUGUGGAGAUCAGUAGAAGGGGAGCUCAACGCAAUCCUGGAAGAGAAUGGAGUUCAACUGACGGUCGUUGUGGGAUCUGUAAAAAACCCCAUGUGGAGAGGUCCACAGAGAUUGCCCGUGCCUGUGAACGAGCUG
KU321639    AGAUGGUAUCUGCGGGAUCUCCCUCUGUUUCAAGAAUGGAAAACAUCAUGUGGAGAUCAGUAGAAGGGGAGCUCAACGCAAUCCUGGAAGAGAAUGGAGUUCAACUGACGGUCGUUGUGGGAUCUGUAAAAAACCCCAUGUGGAGAGGUCCACAGAGAUUGCCCGUGCCUGUGAACGAGCUG
KU820899    AGAUGGUAUCUGUGGGAUCUCCCUCUGUUUCAAGAAUGGAAAACAUCAUGUGGAGAUCAGUAGAAGGGGAGCUCAACGCAAUCCUGGAAGAGAAUGGAGUUCAACUGACGGUCGUUGUGGGAUCUGUAAAAAACCCCAUGUGGAGAGGUCCACAGAGAUUGCCCGUGCCUGUGAACGAGCUG
KU853012    AGAUGGUAUCUGCGGGAUCUCCCUCUGUUUCAAGAAUGGAAAACAUCAUGUGGAGAUCAGUAGAAGGGGAGCUCAAUGCAAUCCUGGAAGAGAAUGGAGUUCAACUGACGGUCGUUGUGGGAUCUGUAAAAAACCCCAUGUGGAGAGGUCCACAGAGAUUGCCCGUGCCUGUGAACGAGCUG
KU729217    AGAUGGUAUCUGCGGGAUCUCCCUCUGUUUCAAGAAUGGAAAACAUCAUGUGGAGAUCAGUAGAAGGGGAGCUCAAUGCAAUCCUGGAAGAGAAUGGAGUUCAACUGACGGUCGUUGUGGGAUCUGUGAAAAACCCCAUGUGGAGAGGUCCACAGAGAUUGCCCGUGCCUGUGAACGAGCUG
KU729218    AGAUGGUAUCUGCGGGAUCUCCCUCUGUUUCAAGAAUGGAAAACAUCAUGUGGAGAUCAGUAGAAGGGGAGCUCAACGCAAUCCUGGAAGAGAAUGGAGUUCAACUGACGGUCGUUGUGGGAUCUGUAAAAAACCCCAUGUGGAGAGGUCCACAGAGAUUGCCCGUGCCUGUGAACGAGCUG
KU761564    AGAUGGUAUCUGCGGGAUCUCCCUCUGUUUCAAGAAUGGAAAACAUCAUGUGGAGAUCAGUAGAAGGGGAGCUCAACGCAAUCCUGGAAGAGAAUGGAGUUCAACUGACGGUCGUUGUGGGAUCUGUAAAAAACCCCAUGUGGAGAGGUCCACAGAGAUUGCCCGUGCCUGUGAACGAGCUG
KU740184    AGAUGGUAUCUGCGGGAUCUCCCUCUGUUUCAAGAAUGGAAAACAUCAUGUGGAGAUCAGUAGAAGGGGAGCUCAACGCAAUCCUGGAAGAGAAUGGAGUUCAACUGACGGUCGUUGUGGGAUCUGUAAAAAACCCCAUGUGGAGAGGUCCACAGAGAUUGCCCGUGCCUGUGAACGAGCUG
KU744693    AGAUGGUAUCUGCGGGAUCUCCCUCUGUUUCAAGAAUGGAAAACAUCAUGUGGAGAUCAGUAGAAGGGGAGCUCAACGCAAUCCUGGAAGAGAAUGGAGUUCAACUGACGGUCGUUGUGGGAUCUGUAAAAAACCCCAUGUGGAGAGGUCCACAGAGAUUGCCCGUGCCUGUGAACGAGCUG
KU497555    AGAUGGUAUCUGCGGGAUCUCCCUCUGUUUCAAGAAUGGAAAACAUCAUGUGGAGAUCAGUAGAAGGGGAGCUUAACGCAAUCCUGGAAGAGAAUGGAGUUCAACUGACGGUCGUUGUGGGAUCUGUAAAAAACCCCAUGUGGAGAGGUCCACAGAGAUUGCCCGUGCCUGUGAACGAGCUG
KU527068    AGAUGGUAUCUGCGGGAUCUCCCUCUGUUUCAAGAAUGGAGAACAUCAUGUGGAGAUCAGUAGAAGGGGAGCUCAACGCAAUCUUGGAAGAGAAUGGAGUUCAACUGACGGUCGUUGUGGGAUCUGUAAAAAACCCCAUGUGGAGAGGUCCACAGAGAUUGCCCGUGCCUGUGAACGAGCUG
KU501217    AGAUGGUAUCUGCGGGAUCUCCCUCUGUUUCAAGAAUGGAAAACAUCAUGUGGAGAUCAGUAGAAGGGGAGCUCAACGCAAUCCUGGAAGAGAAUGGAGUUCAACUGACGGUCGUUGUGGGAUCUGUAAAAAACCCCAUGUGGAGAGCUCCACAGAGAUUGCCCGUGCCUGUGAACGAGCUG
KU501216    AGAUGGUAUCUGCGGGAUCUCCCUCUGUUUCAAGAAUGGAAAACAUCAUGUGGAGAUCAGUAGAAGGGGAGCUCAACGCAAUCCUGGAAGAGAAUGGAGUUCAACUGACGGUCGUUGUGGGAUCUGUAAAAAACCCCAUGUGGAGAGCUCCACAGAGAUUGCCCGUGCCUGUGAACGAGCUG
KU501215    AGAUGGUAUCUGCGGGAUCUCCCUCUGUUUCAAGAAUGGAAAACAUCAUGUGGAGAUCAGUAGAAGGGGAGCUCAACGCAAUCCUGGAAGAGAAUGGAGUUCAACUGACGGUCGUUGUGGGAUCUGUAAAAAACCCCAUGUGGAGAGGUCCACAGAGAUUGCCCGUGCCUGUGAACGAGCUG
KU365780    AGAUGGUAUCUGCGGGAUCUCCCUCUGUUUCAAGAAUGGAAAACAUCAUGUGGAGAUCAGUAGAAGGGGAGCUCAACGCAAUCCUGGAAGAGAAUGGAGUUCAACUGACGGUCGUUGUGGGAUCUGUAAAAAACCCCAUGUGGAGAGGUCCACAGAGAUUGCCCGUGCCUGUGAACGAGCUG
KU365779    AGAUGGUAUCUGCGGGAUCUCCCUCUGUUUCAAGAAUGGAAAACAUCAUGUGGAGAUCAGUAGAAGGGGAGCUCAACGCAAUCCUGGAAGAGAAUGGAGUUCAACUGACGGUCGUUGUGGGAUCUGUAAAAAACCCCAUGUGGAGAGGUCCACAGAGAUUGCCCGUGCCUGUGAACGAGCUG
KU707826    AGAUGGUAUCUGCGGGAUCUCCCUCUGUUUCAAGAAUGGAAAACAUCAUGUGGAGAUCAGUAGAAGGGGAGCUCAACGCAAUCCUGGAAGAGAAUGGAGUUCAACUGACGGUCGUUGUGGGAUCUGUAAAAAACCCCAUGUGGAGAGGUCCACAGAGAUUGCCCGUGCCUGUGAACGAGCUG
KU365778    AGAUGGUAUCUGCGGGAUCUCCCUCUGUUUCAAGAAUGGAAAACAUCAUGUGGAGAUCAGUAGAAGGGGAGCUCAACGCAAUCCUGGAAGAGAAUGGAGUUCAACUGACGGUCGUUGUGGGAUCUGUAAAAAACCCCAUGUGGAGAGGUCCACAGAGAUUGCCCGUGCCUGUGAACGAGCUG
KU365777    AGAUGGUAUCUGCGGGAUCUCCCUCUGUUUCAAGAAUGGAAAACAUCAUGUGGAGAUCAGUAGAAGGGGAGCUCAACGCAAUCCUGGAAGAGAAUGGAGUUCAACUGACGGUCGUUGUGGGAUCUGUAAAAAACCCCAUGUGGAGAGGUCCACAGAGAUUGCCCGUGCCUGUGAACGAGCUG
KU312312    AGAUGGUAUCUGCGGGAUCUCCCUCUGUUUCAAGAAUGGAAAACAUCAUGUGGAGAUCAGUAGAAGGGGAGCUCAACGCAAUCCUGGAAGAGAAUGGAGUUCAACUGACGGUCGUUGUGGGAUCUGUAAAAAACCCCAUGUGGAGAGGUCCACAGAGAUUGCCCGUGCCUGUGAACGAGCUG
KF268950    AGAGGGGAUUUGUGGGAUCUCCCUCCGUUUCAAGAAUGGAAAACAUCAUGUGGAAAUCAGUGGAAGGGGAGCUCAAUGCUAUCUUAGAGGAGAAUGGAGUUCAACUGACAGUUGUUGUAGGAUCUGUAAAAAACCCCAUGUGGAGAGGCCCACAAAGAUUGCCAGUGCCUGUGAAUGAGCUG
KF268949    AGAGGGAAUUUGUGGGAUCUCCCUCCGUUUCAAGAAUGGAAAACAUCAUGUGGAAAUCAGUGGAAGGGGAGCUCAAUGCUAUCUUAGAGGAGAAUGGAGUUCAACUGACAGUUGUUGUAGGAUCUGUAAAAAACCCCAUGUGGAGAGGUCCACAAAGAUUGCCAGUGCCUGUGAAUGAGCUG
KF268948    AGAGGGGAUUUGUGGGAUCUCCCUCCGUUUCAAGAAUGGAAAACAUCAUGUGGAAAUCAGUGGAAGGGGAGCUCAAUGCUAUCUUAGAGGAGAAUGGAGUUCAACUGACAGUUGUUGUAGGAUCUGUAAAAAACCCCAUGUGGAGAGGCCCACAAAGAUUGCCAGUGCCUGUGAAUGAGCUG
KJ776791    AGAUGGUAUCUGUGGGAUCUCCCUCUGUUUCAAGAAUGGAAAACAUCAUGUGGAGAUCAGUAGAAGGGGAGCUCAACGCAAUCCUGGAAGAGAAUGGAGUUCAACUGACGGUCGUUGUGGGAUCUGUAAAAAACCCCAUGUGGAGAGGUCCACAGAGAUUGCCCGUGCCUGUGAACGAGCUG
KF383119    AGAGGGGAUCUGUGGGAUCUCAUCCGUUUCAAGAAUGGAAAACAUCAUGUGGAAAUCAGUAGAAGGGGAGCUCAAUGCUAUCCUAGAGGAGAAUGGAGUUCAACUGACAGUUGUUGUGGGAUCUGUAAAAAACCCCAUGUGGAGAGGUCCACAAAGAUUGCCAGUGCCUGUGAAUGGGCUG
KF383118    AGAGGGGAUCUGUGGGAUCUCAUCCGUUUCAAGAAUGGAAAACAUCAUGUGGAAAUCAGUAGAAGGGGAGCUCAAUGCUAUCCUAGAGGAGAAUGGAGUUCAACUGACAGUUGUUGUGGGAUCUGUAAAAAACCCCAUGUGGAGAGGUCCACAAAGAUUGCCAGUGCCUGUGAAUG?GCUG
KF383117    AGAGGGGAUUUGUGGGAUCUCCCUCCGUUUCGAGAAUGGAAAACAUCAUGUGGAAAUCAGUGGAAGGGGAGCUUAAUGCAAUCCUAGAGGAGAAUGGAGUCCAACUGACAGUUGUAGUGGGGUCUGUAAAAAACCCCAUGUGGAGAGGUCCACAAAGAUUGCCAGUGCCCGUGAAUGAGCUG
KF383116    AGAGGGGAUUUGUGGGAUCUCCCUCCGUUUCGAGAAUGGAAAACAUCAUGUGGAAAUCAGUGGAAGGGGAGCUUAAUGCAAUCCUAGAGGAGAAUGGAGUCCAACUGACAGUUGUAGUGGGGUCUGUAAAAAACCCCUAUGUGGAGAGGUCCACAAAGAUUGCCAGUGCCCGUGAAUGAGCUG
KF383115    AGAGGGGAUUUGUGGGAUCUCCCUCCGUUUCAAGAAUGGAAAACAUCAUGUGGAAAUCAGUGGAAGGGGAGCUCAAUGCUAUCUUAGAGGAGAAUGGAGUUCAACUGACAGUUGUUGUGGGAUCUGUAAAAAACCCCAUGUGGAGAGGUCCACAAAGAUUGCCAGUGCCUGUAAAUGAGCUG
EU545988    AGAUGGGAUCUGUGGGAUCUCCCUCUGUUUCAAGAAUGGAAAACAUCAUGUGGAGAUCAGUAGAAGGGGAGCUCAACGCAAUCCUGGAAGAGAAUGGAGUUCAACUGACGGUCGUUGUGGGAUCUGUAAAAAACCCCAUGUGGAGAGGUCCACAGAGAUUGCCCGUGCCUGUGAACGAGCUG
KU955589    AGAUGGUAUCUGUGGGAUCUCCCUCUGUUUCAAGAAUGGAAAACAUCAUGUGGAGAUCAGUAGAAGGGGAGCUCAACGCAAUCCUGGAAGAGAAUGGAGUUCAACUGACGGUCGUUGUGGGAUCUGUAAAAAACCCCAUGUGGAGAGGUCCACAGAGAUUGCCCGUGCCUGUGAACGAGCUG
KU955590    AGAUGGUAUCUGCGGGAUCUCCCUCUGUUUCAAGAAUGGAAAACAUCAUGUGGAGAUCAGUAGAAGGGGAGCUCAACGCAAUCCUGGAAGAGAAUGGAGUUCAACUGACGGUCGUUGUGGGAUCUGUAAAAAACCCCAUGUGGAGAGGUCCACAGAGAUUGCCCGUGCCUGUGAACGAGCUG
KU940228    AGAUGGUAUCUGCGGGAUCUCCCUCUGUUUCAAGAAUGGAAAACAUCAUGUGGAGAUCAGUAGAAGGGGAGCUCAACGCAAUCCUGGAAGAGAAUGGAGUUCAACUGACGGUCGUUGUGGGAUCUGUAAAAAACCCCAUGUGGAGAGGUCCACAGAGAUUGCCCGUGCCUGUGAACGAGCUG
KU991811    AGAUGGUAUCUGCGGGAUCUCCCUCUGUUUCAAGAAUGGAAAAUAUCAUGUGGAGAUCAGUAGAAGGGGAGCUCAACGCAAUCCUGGAAGAGAAUGGAGUUCAACUGACGGUCGUUGUGGGAUCUGUAAAAAACCCCAUGUGGAGAGGUCCACAGAGAUUGCCCGUGCCUGUGAACGAGCUG
KU509998    AGAUGGUAUCUGCGGGAUCUCCCUCUGUUUCAAGAAUGGAAAACAUCAUGUGGAGAUCAGUAGAAGGGGAGCUCAACGCAAUCCUGGAAGAGAAUGGAGUUCAACUGACGGUCGUUGUGGGAUCUGUAAAAAACCCCAUGUGGAGAGGUCCACAGAGAUUGCCCGUGCCUGUGAACGAGCUG
KX056898    AGAUGGUAUCUGCGGGAUCUCCCUCUGUUUCAAGAAUGGAAAACAUCAUGUGGAGAUCAGUAGAAGGGGAGCUCAACGCAAUCCUGGAAGAGAAUGGAGUUCAACUGACGGUCGUUGUGGGAUCUGUAAAAAACCCCAUGUGGAGAGGUCCACAGAGAUUGCCCGUGCCUGUGAACGAGCUG
KX117076    AGAUGGUAUCUGUGGGAUCUCCCUCUGUUUCAAGAAUGGAAAACAUCAUGUGGAGAUCAGUAGAAGGGGAGCUCAACGCAAUCCUGGAAGAGAAUGGAGUUCAACUGACGGUCGUUGUGGGAUCUGUAAAAAACCCCAUGUGGAGAGGUCCACAGAGAUUGCCCGUGCCUGUGAACGAGCUG
KX185891    AGAUGGUAUCUGUGGGAUCUCCCUCUGUUUCAAGAAUGGAAAACAUCAUGUGGAGAUCAGUAGAAGGGGAGCUCAACGCAAUCCUGGAAGAGAAUGGAGUUCAACUGACGGUCGUUGUGGGAUCUGUAAAAAACCCCAUGUGGAGAGGUCCACAGAGAUUGCCCGUGCCUGUGAACGAGCUG
KX247646    AGAUGGUAUCUGCGGGAUCUCCCUCUGUUUCAAGAAUGGAAAACAUCAUGUGGAGAUCAGUAGAAGGGGAGCUCAACGCAAUCCUGGAAGAGAAUGGAGUUCAACUGACGGUCGUUGUGGGAUCUGUAAAAAACCCCAUGUGGAGAGGUCCACAGAGAUUGCCCGUGCCUGUGAACGAGCUG
KX262887    AGAUGGUAUCUGCGGGAUCUCCCUCUGUUUCAAGAAUGGAAAACAUCAUGUGGAGAUCAGUAGAAGGGGAGCUCAACGCAAUCCUGGAAGAGAAUGGAGUUCAACUGACGGUCGUUGUGGGAUCUGUAAAAAACCCCAUGUGGAGAGCUCCACAGAGAUUGCCCGUGCCUGUGAACGAGCUG
KX280026    AGAUGGUAUCUGCGGGAUCUCCCUCUGUUUCAAGAAUGGAAAACAUCAUGUGGAGAUCAGUAGAAGGGGAGCUCAACGCAAUCCUGGAAGAGAAUGGAGUUCAACUGACGGUCGUUGUGGGAUCUGUAAAAAACCCCAUGUGGAGAGGUCCACAGAGAUUGCCCGUGCCUGUGAACGAGCUG
KU758877    AGAUGGUAUCUGCGGGAUCUCCCUCUGUUUCAAGAAUGGAAAACAUCAUGUGGAGAUCAGUAGAAGGGGAGCUCAACGCAAUCCUGGAAGAGAAUGGAGUUCAACUGACGGUCGUUGUGGGAUCUGUAAAAAACCCCAUGUGGAGAGGUCCACAGAGAUUGCCCGUGCCUGUGAACGAGCUG
KU866423    AGAUGGUAUCUGUGGGAUCUCCCUCUGUUUCAAGAAUGGAAAACAUCAUGUGGAGAUCAGUAGAAGGGGAGCUCAACGCAAUCCUGGAAGAGAAUGGAGUUCAACUGACGGUCGUUGUGGGAUCUGUAAAAAACCCCAUGUGGAGAGGUCCACAGAGAUUGCCCGUGCCUGUGAACGAGCUG
KX377336    AGAUGGGAUCUGUGGGAUCUCCCUCUGUUUCAAGAAUGGAAAACAUUAUGUGGAGAUCAGUAGAAGGGGAGCUCAACGCAAUUCUGGAAGAGAAUGGAGUUCAACUGACGGUCGUUGUGGGAUCUGUAAAAAACCCCAUGUGGAGAGGUCCGCAGAGGUUGCCUGUGCCUGUGAAUGAGCUG
KX377337    AGAUGGUAUCUGCGGGAUCUCCCUCUGUUUCAAGAAUGGAAAACAUCAUGUGGAGAUCAGUAGAAGGGGAGCUCAACGCAAUCCUGGAAGAGAAUGGAGUUCAACUGACGGUCGUUGUGGGAUCUGUAAAAAACCCCAUGGGGAGAGGUCCACAGAGAUUGCCCGUGCCUGUGAACGAGCUG
KX369547    AGAUGGUAUCUGUGGGAUCUCCCUCUGUUUCAAGAAUGGAAAACAUCAUGUGGAGAUCAGUAGAAGGGGAGCUCAACGCAAUCCUGGAAGAGAAUGGAGUUCAACUGACGGUCGUUGUGGGAUCUGUAAAAAACCCCAUGUGGAGAGGUCCACAGAGAUUGCCCGUGCCUGUGAACGAGCUG
```

```
KX548902    AGAUGGUAUCUGCGGGAUCUCCUCUGUUUCAAGAAUGGAAAACAUCAUGUGGAGAUCAGUAGAAGGGGAGCUCAACGCAAUCCUGGAAGAGAAUGGAGUUCAACUGACGGUCGUUGUGGGAUCUGUAAAAAACCCCAUGUGGAGAGGUCCACAGAGAUUGCCCGUGCCUGUGAACGAGCUG
KX601166    AGAGGGGAUUUGUGGGAUCUCCUCCGUUUCGAGAAUGGAAAACAUCAUGUGGAAAUCAGUGGAAGGGGAGCUUAAUGCAAUCCUAGAGGAGAAUGGAGUUCAACUGACAGUUGUAGUGGGGUCUGUAAAAAACCCUAUGUGGAGAGGUCCACAGAGAUUGCCAGUGCCUGUGAAUGAGCUG
KX601167    AGAUGGGAUCUGUGGGAUCUCCUCUGUUUCAAGAAUGGAAAACAUUAUGUGGAGAUCAGUAGAAGGGGAGCUCAACGCAAUUCUGGAAGAGAAUGGAGUUCAACUGACGGUCGUUGUGGGAUCUGUAAAAAACCCCAUGUGGAGAGGUCCGCAGAGGUUGCCUGUGCCUGUGAAUGAGCUG
KX601168    AGAUGGUAUCUGCGGGAUCUCCUCUGUUUCAAGAAUGGAAAACAUCAUGUGGAGAUCAGUAGAAGGGGAGCUCAACGCAAUCCUGGAAGAGAAUGGAGUUCAACUGACGGUCGUUGUGGGAUCUGUAAAAAACCCCAUGUGGAGAGGUCCACAGAGAUUGCCCGUGCCUGUGAACGAGCUG
KX766028    AGAUGGUAUCUGCGGGAUCUCCUCUGUUUCAAGAAUGGAAAACAUCAUGUGGAGAUCAGUAGAAGGGGAGCUCAACGCAAUCCUGGAAGAGAAUGGAGUUCAACUGACGGUCGUUGUGGGAUCUGUAAAAAACCCCAUGUGGAGAGGUCCACAGAGAUUGCCCGUGCCUGUGAACGAGCUG
KX766029    AGAUGGUAUCUGCGGGAUCUCCUCUGUUUCAAGAAUGGAAAACAUCAUGUGGAGAUCAGUAGAAGGGGAGCUCAACGCAAUCCUGGAAGAGAAUGGAGUUCAACUGACGGUCGUUGUGGGAUCUGUAAAAAACCCCAUGUGGAGAGCUCCACAGAGAUUGCCCGUGCCUGUGAACGAGCUG
KU940224    AGAUGGUAUCUGCGGGAUCUCCUCUGUUUCAAGAAUGGAAAACAUCAUGUGGAGAUCAGUAGAAGGGGAGCUCAACGCAAUCCUGGAAGAGAAUGGAGUUCAACUGACGGUCGUUGUGGGAUCUGUAAAAAACCCCAUGUGGAGAGGUCCACAGAGAUUGCCCGUGCCUGUGAACGAGCUG


            2222222222222222222222222222222222222222222222222222222222222222222222222222222222222222222222222222222222222222222222222222222222222222222222222222222222222222222222222222222222222
            7777777777777777777777777777777777777777777777777777777777777777777777777777777777778888888888888888888888888888888888888888888888888888888888888888888888888888888888888888888888888
            1111222222222233333333334444444444555555555566666666667777777777888888888899999999990000000000111111111122222222223333333333444444444455555555556666666666777777777788888888889999999
Sequence    6789012345678901234567890123456789012345678901234567890123456789012345678901234567890123456789012345678901234567890123456789012345678901234567890123456789012345678901234567890123456
-------------------------------------------------------------------------------------------------------------------------------------------------------------------------------------------------------
NC_012532   CCCCAUGGCUGGAAAGCCUGGGGGAAAUCGUAUUUUGUUAGGGCGGCAAAGACCAACAACAGUUUUGUUGUCGACGGUGACACACUGAAGGAAUGUCCGCUUGAGCACAGAGCAUGGAAUAGUUUUCUUGUGGAGGAUCACGGGUUUGGAGUCUUCCACACCAGUGUCUGGCUUAAGGUCA
KU922960    CCCCACGGCUGGAAGGCUUGGGGGAAAUCGUACUUCGUCAGAGCAGCAAAGACAAAUAACAGCUUUGUCGUGGAUGGUGACACACUGAAGGAAUGCCCACUCAAACAUAGAGCAUGGAACAGCUUUCUUGUGGAGGAUCAUGGGUUCGGGGUAUUUCACACUAGUGUCUGGCUCAAGGUUA
KU922923    CCCCACGGCUGGAAGGCUUGGGGGAAAUCGUACUUCGUCAGAGCAGCAAAGACAAAUAACAGCUUUGUCGUGGAUGGUGACACACUGAAGGAAUGCCCACUCAAACAUAGAGCAUGGAACAGCUUUCUUGUGGAGGAUCAUGGGUUCGGGGUAUUUCACACUAGUGUCUGGCUCAAGGUUA
KU820898    CCCCACGGCUGGAAGGCUUGGGGGAAAUCGUACUUCGUCAGAGCAGCAAAGACAAAUAACAGCUUUGUCGUGGAUGGUGACACACUGAAGGAAUGCCCACUCAAACAUAGAGCAUGGAACAGCUUUCUUGUGGAGGAUCAUGGGUUCGGGGUAUUUCACACUAGUGUCUGGCUCAAGGUUA
KU321639    CCCCACGGCUGGAAGGCUUGGGGGAAAUCGCACUUCGUCAGAGCAGCAAAGACAAAUAACAGCUUUGUCGUGGAUGGUGACACACUGAAGGAAUGCCCACUCAAACAUAGAGCAUGGAACAGCUUUCUUGUGGAGGAUCAUGGGUUCGGGGUAUUUCACACUAGUGUCUGGCUCAAGGUUA
KU820899    CCCCACGGCUGGAAGGCUUGGGGGAAAUCGUACUUCGUCAGAGCAGCAAAGACAAAUAACAGCUUUGUCGUGGAUGGUGACACACUGAAGGAAUGCCCACUCAAACAUAGAGCAUGGAACAGCUUUCUUGUGGAGGAUCAUGGGUUCGGGGUAUUUCACACUAGUGUCUGGCUCAAGGUUA
KU853012    CCCCACGGCUGGAAGGCUUGGGGGAAAUCGUACUUCGUUAGAGCAGCAAAGACAAAUAACAGCUUUGUCGUGGAUGGUGACACACUGAAGGAAUGCCCACUCAAACAUAGAGCAUGGAACAGCUUUCUUGUGGAGGAUCAUGGGUUCGGGGUAUUUCACACUAGUGUCUGGCUCAAGGUUA
KU729217    CCCCACGGCUGGAAGGCUUGGGGGAAAUCGUACUUCGUCAGAGCAGCAAAGACAAAUAACAGCUUUGUCGUGGAUGGUGACACACUGAAGGAAUGCCCACUCAAACAUAGAGCAUGGAACAGCUUUCUUGUGGAGGAUCAUGGGUUCGGGGUAUUUCACACUAGUGUCUGGCUCAAGGUUA
KU729218    CCCCACGGCUGGAAGGCUUGGGGGAAAUCGUACUUCGUCAGAGCAGCAAAGACAAAUAACAGCUUUGUCGUGGAUGGUGACACACUGAAGGAAUGCCCACUCAAACAUAGAGCAUGGAACAGCUUUCUUGUGGAGGAUCAUGGGUUCGGGGUAUUUCACACUAGUGUCUGGCUCAAGGUUA
KU761564    CCCCACGGCUGGAAGGCUUGGGGGAAAUCGUACUUCGUCAGAGCAGCAAAGACAAAUAACAGCUUUGUCGUGGAUGGUGACACACUGAAGGAAUGCCCACUCAAACAUAGAGCAUGGAACAGCUUUCUUGUGGAGGAUCAUGGGUUCGGGGUAUUUCACACUAGUGUCUGGCUCAAGGUUA
KU740184    CCCCACGGCUGGAAGGCUUGGGGGAAAUCGUACUUCGUCAGAGCAGCAAAGACAAAUAACAGCUUUGUCGUGGAUGGUGACACACUGAAGGAAUGCCCACUCAAACAUAGAGCAUGGAACAGCUUUCUUGUGGAGGAUCAUGGGUUCGGGGUAUUUCACACUAGUGUCUGGCUCAAGGUUA
KU744693    CCCCACGGCUGGAAGGCUUGGGGGAAAUCGUACUUCGUCAGAGCAGCAAAGACAAAUAACAGCUUUGUCGUGGAUGGUGACACACUGAAGGAAUGCCCACUCAAACAUAGAGCAUGGAACAGCUUUCUUGUGGAGGAUCAUGGGUUCGGGGUAUUUCACACUAGUGUCUGGCUCAAGGUUA
KU497555    CCCCACGGCUGGAAGGCUUGGGGGAAAUCGUACUUCGUCAGAGCAGCAAAGACAAAUAACAGCUUUGUCGUGGAUGGUGACACACUGAAGGAAUGCCCACUCAAACAUAGAGCAUGGAACAGCUUUCUUGUGGAGGAUCAUGGGUUCGGGGUAUUUCACACUAGUGUCUGGCUCAAGGUUA
KU527068    CCCCACGGCUGGAAGGCUUGGGGGAAAUCGUACUUCGUCAGAGCAGCAAAGACAAAUAACAGCUUUGUCGUGGAUGGUGACACACUGAAGGAAUGCCCACUCGAACAUAGAGCAUGGAACAGCUUUCUUGUGGAGGAUCAUGGGUUCGGGGUAUUUCACACUAGUGUCUGGCUCAAGGUUA
KU501217    CCCCACGGCUGGAAGGCUUGGGGGAAAUCGUACUUCGUCAGAGCAGCAAAGACAAAUAACAGCUUUGUCGUGGAUGGUGACACACUGAAGGAAUGCCCACUCAAACAUAGAGCAUGGAACAGCUUUCUUGUGGAGGAUCAUGGGUUCGGGGUAUUUCACACUAGUGUCUGGCUCAAGGUUA
KU501216    CCCCACGGCUGGAAGGCUUGGGGGAAAUCGUACUUCGUCAGAGCAGCAAAGACAAAUAACAGCUUUGUCGUGGAUGGUGACACACUGAAGGAAUGCCCACUCAAACAUAGAGCAUGGAACAGCUUUCUUGUGGAGGAUCAUGGGUUCGGGGUAUUUCACACUAGUGUCUGGCUCAAGGUUA
KU501215    CCCCACGGCUGGAAGGCUUGGGGGAAAUCGUAUUUCGUCAGAGCAGCAAAGACAAAUAACAGCUUUGUCGUGGAUGGUGACACACUGAAGGAAUGCCCACUCAAACAUAGAGCAUGGAACAGCUUUCUUGUGGAGGAUCAUGGGUUCGGGGUAUUUCACACUAGUGUCUGGCUCAAGGUUA
KU365780    CCCCACGGCUGGAAGGCUUGGGGGAAAUCGUACUUCGUCAGAGCAGCAAAGACAAAUAACAGCUUUGUCGUGGAUGGUGACACACUGAAGGAAUGCCCACUCAAACAUAGAGCAUGGAACAGCUUUCUUGUGGAGGAUCAUGGGUUCGGGGUAUUUCACACUAGUGUCUGGCUCAAGGUUA
KU365779    CCCCACGGCUGGAAGGCUUGGGGGAAAUCGUACUUCGUCAGAGCAGCAAAGACAAAUAACAGCUUUGUCGUGGAUGGUGACACACUGAAGGAAUGCCCACUCAAACAUAGAGCAUGGAACAGCUUUCUUGUGGAGGAUCAUGGGUUCGGGGUAUUUCACACUAGUGUCUGGCUCAAGGUUA
KU707826    CCCCACGGCUGGAAGGCUUGGGGGAAAUCGUACUUCGUCAGAGCAGCAAAGACAAAUAACAGCUUUGUCGUGGAUGGUGACACACUGAAGGAAUGCCCACUCAAACAUAGAGCAUGGAACAGCUUUCUUGUGGAGGAUCAUGGGUUCGGGGUAUUUCACACUAGUGUCUGGCUCAAGGUUA
KU365778    CCCCACGGCUGGAAGGCUUGGGGGAAAUCGUACUUCGUCAGAGCAGCAAAGACAAAUAACAGCUUUGUCGUGGAUGGUGACACACUGAAGGAAUGCCCACUCAAACAUAGAGCAUGGAACAGCUUUCUUGUGGAGGAUCAUGGGUUCGGGGUAUUUCACACUAGUGUCUGGCUCAAGGUUA
KU365777    CCCCACGGCUGGAAGGCUUGGGGGAAAUCGUACUUCGUCAGAGCAGCAAAGACAAAUAACAGCUUUGUCGUGGAUGGUGACACACUGAAGGAAUGCCCACUCAAACAUAGAGCAUGGAACAGCUUUCUUGUGGAGGAUCAUGGGUUCGGGGUAUUUCACACUAGUGUCUGGCUCAAGGUUA
KU312312    CCCCACGGCUGGAAGGCUUGGGGGAAAUCGUACUUCGUCAGAGCAGCAAAGACAAAUAACAGCUUUGUCGUGGAUGGUGACACACUGAAGGAAUGCCCACUCAAACAUAGAGCAUGGAACAGCUUUCUUGUGGAGGAUCAUGGGUUCGGGGUAUUUCACACUAGUGUCUGGCUCAAGGUUA
KF268950    CCCCAUGGCUGGAAAGCCUGGGGGAAAUCAUAUUUUGUCAGAGCGGCAAAGACCAACAACAGUUUUGUUGUCGAUGGUGACACACUGAAGGAAUGUCCGCUCAAACACAGAGCAUGGAAUAGUUUCCUUGUGGAGGAUCAUGGGUUUGGGGUCUUCCACACCAGUGUUUGGCUCAAGGUUA
KF268949    CCCCAUGGCUGGAAAGCCUGGGGGAAAUCAUAUUUUGUCAGAGCGGCAAAGACCAACAACAGGUUUGUUGUCGAUGGUGACACACUGAAGGAAUGUCCGCUCAAACACAGAGCAUGGAAUAGUUUCCUUGUGGAGGAUCAUGGGUUUGGGGUCUUCCACACCAGUGUUUGGCUUAAGGUUA
KF268948    CCCCAUGGCUGGAAAGCCUGGGGGAAAUCAUAUUUUGUCAGAGCGGCAAAGACCAACAACAGUUUUGUUGUCGAUGGUGACACACUGAAGGAAUGUCCGCUCAAACACAGAGCAUGGAAUAGUUUCCUUGUGGAGGAUCAUGGGUUUGGGGUCUUCCACACCAGUGUUUGGCUCAAGGUUA
KJ776791    CCCCACGGCUGGAAGGCUUGGGGGAAAUCGUACUUCGUCAGAGCAGCAAAGACAAAUAACAGCUUUGUCGUGGAUGGUGACACACUGAAGGAAUGCCCACUCAAACAUAGAGCAUGGAACAGCUUUCUUGUGGAGGAUCAUGGGUUCGGGGUAUUUCACACUAGUGUCUGGCUCAAGGUUA
KF383119    CCCCAUGGCUGGAAAGCCUGGGGGAAAUCGUAUUUUGUUAGGGCGGCAAAGACCAACAACAGUUUUGUUGUCGACGGUGACACACUGAAGGAAUGUCCGCUUAAGCACAGAGCAUGGAAUAGUUUUCUUGUGGAGGAUCACGGGUUUGGAGUCUUCCACACCAGUGUUUGGCUUAAGGUCA
KF383118    CCCCAUGGCUGGAAAGCCUGGGGGAAAUCGUAUUUUGUUAGGGCGGCAAAGACCAACAACAGUUUUGUUGUCGACGGUGACACACUGAAGGAAUGUCCGCUUAAGCACAGAGCAUGGAAUAGUUUUCUUGUGGAGGAUCACGGGUUUGGAGUCUUCCACACCAGUGUUUGGCUUAAGGUCA
KF383117    CCCCAUGGCUGGAAAGCCUGGGGGAAAUCGUACUUUGUCAGAGCGGCAAAGACCAACAACAGUUUUGUUGUCGACGGAGACACACUGAAGGAGUGUCCGCUCAAACAUAGAGCAUGGAAUAGCUUCCUUGUGGAGGAUCACGGGUUUGGGAUUUUCCACACCAGUGUUUGGCUGAAGGUCA
KF383116    CCCCAUGGCUGGAAAGCCUGGGGGAAAUCGUACUUUGUCAGAGCGGCAAAGACCAACAACAGUUUUGUUGUCGACGGUGACACACUGAAGGAGUGUCCGCUCAAACAUAGAGCAUGGAAUAGCUUCCUUGUGGAGGAUCACGGGUUUGGGAUCUUCCACACCAGUGUUUGGCUGAAGGUCA
KF383115    CCCCAUGGCUGGAAAGCCUGGGGGAAAUCAUAUUUUGUCAGAGCGGCAAAGACCAACAACAGUUUUGUUGUCGACGGUGACACACUGAAGGAAUGUCCGCUCAAACACAGAGCAUGGAACAGUUUCCUUGUGGAGGAUCACGGGUUUGGGGUCUUCCACACUAGUGUUUGGCUUAAGGUUA
EU545988    CCCCACGGCUGGAAGGCUUGGGGGAAAUCGUACUUCGUCAGAGCAGCAAAGACAAAUAACAGCUUUGUCGUGGAUGGUGACACACUGAAGGAAUGCCCACUCAAACAUAGAGCAUGGAACAGCUUUCUUGUGGAGGAUCAUGGGUUCGGGGUAUUUCACACUAGUGUCUGGCUCAAGGUUA
KU955589    CCCCACGGCUGGAAGGCUUGGGGGAAAUCGUACUUCGUCAGAGCAGCAAAGACAAAUAACAGCUUUGUCGUGGAUGGUGACACACUGAAGGAAUGCCCACUCAAACAUAGAGCAUGGAACAGCUUUCUUGUGGAGGAUCAUGGGUUCGGGGUAUUUCACACUAGUGUCUGGCUCAAGGUUA
KU955590    CCCCACGGCUGGAAGGCUUGGGGGAAAUCGUACUUCGUCAGAGCAGCAAAGACAAAUAACAGCUUUGUCGUGGAUGGUGACACACUGAAGGAAUGCCCACUCAAACAUAGAGCAUGGAACAGCUUUCUUGUGGAGGAUCAUGGGUUCGGGGUAUUUCACACUAGUGUCUGGCUCAAGGUUA
KU940228    CCCCACGGCUGGAAGGCUUGGGGGAAAUCGUACUUCGUCAGAGCAGCAAAGACAAAUAACAGCUUUGUCGUGGAUGGUGACACACUGAAGGAAUGCCCACUCAAACAUAGAGCAUGGAACAGCUUUCUUGUGGAGGAUCAUGGGUUCGGGGUAUUUCACACUAGUGUCUGGCUCAAGGUUA
KU991811    CCCCACGGCUGGAAGGCUUGGGGGAAAUCGUACUUCGUCAGAGCAGCAAAGACAAAUAACAGCUUUGUCGUGGAUGGUGACACACUGAAGGAAUGCCCACUCAAACAUAGAGCAUGGAACAGCUUUCUUGUGGAGGAUCAUGGGUUCGGGGUAUUUCACACUAGUGUCUGGCUCAAGGUUA
KU509998    CCCCACGGCUGGAAGGCUUGGGGGAAAUCGCACUUCGUCAGAGCAGCAAAGACAAAUAACAGCUUUGUCGUGGAUGGUGACACACUGAAGGAAUGCCCACUCAAACAUAGAGCAUGGAACAGCUUUCUUGUGGAGGAUCAUGGGUUCGGGGUAUUUCACACUAGUGUCUGGCUCAAGGUUA
KX056898    CCCCACGGCUGGAAGGCUUGGGGGAAAUCGUACUUCGUCAGAGCAGCAAAGACAAAUAACAGCUUUGUCGUGGAUGGUGACACACUGAAGGAAUGCCCACUCAAACAUAGAGCAUGGAACAGCUUUCUUGUGGAGGAUCAUGGGUUCGGGGUAUUUCACACUAGUGUCUGGCUCAAGGUUA
KX117076    CCCCACGGCUGGAAGGCUUGGGGGAAAUCGUACUUCGUCAGAGCAGCAAAGACAAAUAACAGCUUUGUCGUGGAUGGUGACACACUGAAGGAAUGCCCACUCAAACAUAGAGCAUGGAACAGCUUUCUUGUGGAGGAUCAUGGGUUCGGGGUAUUUCACACUAGUGUCUGGCUCAAGGUUA
KX185891    CCCCACGGCUGGAAGGCUUGGGGGAAAUCGUACUUCGUCAGAGCAGCAAAGACAAAUAACAGCUUUGUCGUGGAUGGUGACACACUGAAGGAAUGCCCACUCAAACAUAGAGCAUGGAACAGCUUUCUUGUGGAGGAUCAUGGGUUCGGGGUAUUUCACACUAGUGUCUGGCUCAAGGUUA
KX247646    CCCCACGGCUGGAAGGCUUGGGGGAAAUCGUACUUCGUCAGAGCAGCAAAGACAAAUAACAGCUUUGUCGUGGAUGGUGACACACUGAAAGAAUGCCCACUCAAACAUAGAGCAUGGAACAGCUUUCUUGUGGAGGAUCAUGGGUUCGGGGUAUUUCACACUAGUGUCUGGCUCAAGGUUA
KX262887    CCCCACGGCUGGAAGGCUUGGGGGAAAUCGUACUUCGUCAGAGCAGCAAAGACAAAUAACAGCUUUGUCGUGGAUGGUGACACACUGAAGGAAUGCCCACUCAAACAUAGAGCAUGGAACAGCUUUCUUGUGGAGGAUCAUGGGUUCGGGGUAUUUCACACUAGUGUCUGGCUCAAGGUUA
KX280026    CCCCACGGCUGGAAGGCUUGGGGGAAAUCGUACUUCGUCAGAGCAGCAAAGACAAAUAACAGCUUUGUCGUGGAUGGUGACACACUGAAGGAAUGCCCACUCAAACAUAGAGCAUGGAACAGCUUUCUUGUGGAGGAUCAUGGGUUCGGGGUAUUUCACACUAGUGUCUGGCUCAAGGUUA
KU758877    CCCCACGGCUGGAAGGCUUGGGGGAAAUCGUACUUCGUCAGAGCAGCAAAGACAAAUAACAGCUUUGUCGUGGAUGGUGACACACUGAAGGAAUGCCCACUCAAACAUAGAGCAUGGAACAGCUUUCUUGUGGAGGAUCAUGGGUUCGGGGUAUUUCACACUAGUGUCUGGCUCAAGGUUA
KU866423    CCCCACGGCUGGAAGGCUUGGGGGAAAUCGUACUUCGUCAGAGCAGCAAAGACAAAUAACAGCUUUGUCGUGGAUGGUGACACACUGAAGGAAUGCCCACUCAAACAUAGAGCAUGGAACAGCUUUCUUGUGGAGGAUCAUGGGUUCGGGGUAUUUCACACUAGUGUCUGGCUCAAGGUUA
KX377336    CCCCACGGUUGGAAGGCCUGGGGGAAAUCGUACUUUGUCAGGGCAGCAAAGACCAACAACAGCUUUGUUGUGGAUGGUGACACACUGAAGGAAUGCCCGCUCAAACACAGAGCAUGGAACAGCUUUCUUGUGGAGGAUCACGGGUUCGGGGUAUUUCACACUAGUGUCUGGCUUAAAGUCA
KX377337    CCCCACGGCUGGAAGGCUUGGGGGAAAUCGUAUUUCGUCAGAGCAGCAAAGACAAAUAACAGCUUUGUCGUGGAUGGUGACACACUGAAGGAAUGCCCACUCAAACAUAGAGCAUGGAACAGCUUUCUUGUGGAGGAUCAUGGGUUCGGGGUAUUUCACACUAGUGUCUGGCUCAAGGUUA
KX369547    CCCCACGGCUGGAAGGCUUGGGGGAAAUCGUACUUCGUCAGAGCAGCAAAGACAAAUAACAGCUUUGUCGUGGAUGGUGACACACUGAAGGAAUGCCCACUCGAACAUAGAGCAUGGAACAGCUUUCUUGUGGAGGAUCAUGGGUUCGGGGUAUUUCACACUAGUGUCUGGCUCAAGGUUA
KX548902    CCCCACGGCUGGAAGGCUUGGGGGAAAUCGUACUUCGUCAGAGCAGCAAAGACAAAUAACAGCUUUGUCGUGGAUGGUGACACACUGAAGGAAUGCCCACUCAAACAUAGAGCAUGGAACAGCUUUCUUGUGGAGGAUCAUGGGUUCGGGGUAUUUCACACUAGUGUCUGGCUCAAGGUUA
KX601166    CCCCAUGGCUGGAAAGCCUGGGGGAAAUCGUACUUUGUCAGAGCAGCAAAGACCAACAACAGUUUUGUUGUCGACGGUGACACACUGAAGGAGUGUCCGCUCAAACAUAGAGCAUGGAAUAGCUUCCUUGUGGAGGAUCACGGGUUUGGGAUCUUCCACACCAGUGUUUGGCUGAAGGUCA
KX601167    CCCCACGGUUGGAAGGCCUGGGGGAAAUCGUACUUUGUCAGGGCAGCAAAGACCAACAACAGCUUUGUUGUGGAUGGUGACACACUGAAGGAAUGCCCGCUCAAACACAGAGCAUGGAACAGCUUUCUUGUGGAGGAUCACGGGUUCGGGGUAUUUCACACUAGUGUCUGGCUUAAAGUCA
KX601168    CCCCACGGCUGGAAGGCUUGGGGGAAAUCGUAUUUCGUCAGAGCAGCAAAGACAAAUAACAGCUUUGUCGUGGAUGGUGACACACUGAAGGAAUGCCCACUCAAACAUAGAGCAUGGAACAGCUUUCUUGUGGAGGAUCAUGGGUUCGGGGUAUUUCACACUAGUGUCUGGCUCAAGGUUA
KX766028    CCCCACGGCUGGAAGGCUUGGGGGAAAUCGUACUUCGUCAGAGCAGCAAAGACAAAUAACAGCUUUGUCGUGGAUGGUGACACACUGAAGGAAUGCCCACUCAAACAUAGAGCAUGGAACAGCUUUCUUGUGGAGGAUCAUGGGUUCGGGGUAUUUCACACUAGUGUCUGGCUCAAGGUUA
KX766029    CCCCACGGCUGGAAGGCUUGGGGGAAAUCGUACUUCGUUAGAGCAGCAAAGACAAAUAACAGCUUUGUCGUGGAUGGUGACACACUGAAGGAAUGCCCACUCAAACAUAGAGCAUGGAACAGCUUUCUUGUGGAGGAUCAUGGGUUCGGGGUAUUCCACACUAGUGUCUGGCUCAAGGUUA
KU940224    CCCCACGGCUGGAAGGCUUGGGGGAAAUCGUACUUCGUCAGAGCAGCAAAGACAAAUAACAGCUUUGUCGUGGAUGGUGACACACUGAAGGAAUGCCCACUCAAACAUAGAGCAUGGAACAGCUUUCUUGUGGAGGAUCAUGGGUUCGGGGUAUUUCACACUAGUGUCUGGCUCAAGGUUA


            2222222222222222222222222222222222222222222222222222222222222222222222222222222222222222222222222222222333333333333333333333333333333333333333333333333333333333333333333333333333333
            8889999999999999999999999999999999999999999999999999999999999999999999999999999999999999999999999999999000000000000000000000000000000000000000000000000000000000000000000000000000000
            9990000000000111111111122222222223333333333444444444455555555556666666666777777777788888888889999999999000000000011111111112222222222333333333344444444445555555555666666666677777777
Sequence    7890123456789012345678901234567890123456789012345678901234567890123456789012345678901234567890123456789012345678901234567890123456789012345678901234567890123456789012345678901234567
-------------------------------------------------------------------------------------------------------------------------------------------------------------------------------------------------------
```

```
NC_012532   GAGAAGAUUACUCAUUAGAAUGUGACCCAGCCGUCAUAGGAACAGCUGUUAAGGGAAGGGAGGCCGCGCACAGUGAUCUGGGCUAUUGGAUUGAAAGUGAAAAGAAUGACACAUGGAGGCUGAAGAGGGCCCACCUGAUUGAGAUGAAAACAUGUGAAUGGCCAAAGUCUCACACAUUGUG
KU922960    GAGAAGAUUAUUCAUUAGAGUGUGAUCCAGCCGUUAUUGGAACAGCUGUUAAGGGAAAGGAGGCUGUACACAGUGAUCUAGGCUACUGGAUUGAGAGUGAGAAGAAUGACACAUGGAGGCUGAAGAGGGCCCAUCUGAUCGAGAUGAAAACAUGUGAAUGGCCAAAAUCCCACACAUUGUG
KU922923    GAGAAGAUUAUUCAUUAGAGUGUGAUCCAGCCGUUAUUGGAACAGCUGUUAAGGGAAAGGAGGCUGUACACAGUGAUCUAGGCUACUGGAUUGAGAGUGAGAAGAAUGACACAUGGAGGCUGAAGAGGGCCCAUCUGAUCGAGAUGAAAACAUGUGAAUGGCCAAAAUCCCACACAUUGUG
KU820898    GAGAAGACUAUUCAUUAGAGUGUGAUCCAGCCGUUAUUGGAACAGCUGUUAAGGGAAAGGAGGCUGUACACAGUGAUCUAGGCUACUGGAUUGAGAGUGAGAAGAAUGACACAUGGAGGCUGAAGAGGGCCCAUCUGAUCGAGAUGAAAACAUGUGAAUGGCCAAAGUCCCACACAUUGUG
KU321639    GAGAAGAUUAUUCAUUAGAGUGUGAUCCAGCCGUUAUUGGAACAGCUGUUAAGGGAAAGGAGGCUGUACACAGUGAUCUAGGCUACUGGAUUGAGAGUGAGAAGAAUGACACAUGGAGGCUGAAGAGGGCCCAUCUGAUCGAGAUGAAAACAUGUGAAUGGCCAAAGUCCCACACAUUGUG
KU820899    GAGAAGAUUAUUCAUUAGAGUGUGAUCCAGCCGUUAUUGGAACAGCUGUUAAGGGAAAGGAGGCUGUACACAGUGAUCUAGGCUACUGGAUUGAGAGUGAGAAGAAUGACACAUGGAGGCUGAAGAGGGCCCAUCUGAUCGAGAUGAAAACAUGUGAAUGGCCAAAGUCCCACACAUUGUG
KU853012    GAGAAGAUUAUUCAUUAGAGUGUGAUCCAGCCGUUAUUGGAACAGCUGUUAAGGGAAAGGAGGCUGUACACAGUGAUCUAGGCUACUGGAUUGAGAGUGAGAAGAAUGACACAUGGAGGCUGAAGAGGGCCCAUCUGAUCGAGAUGAAAACAUGUGAAUGGCCAAAGUCCCACACAUUGUG
KU729217    GAGAAGAUUAUUCAUUAGAGUGUGAUCCAGCCGUUAUUGGAACAGCUGUUAAGGAAAAGGAGGCUGUACACAGUGAUCUAGGCUACUGGAUUGAGAGUGAGAAGAAUGACACAUGGAGGCUGAAGAGGGCCCAUCUGAUCGAGAUGAAAACAUGUGAAUGGCCAAAGUCCCACACAUUGUG
KU729218    GAGAAGAUUAUUCAUUAGAGUGUGAUCCAGCCGUUAUUGGAACAGCUGUUAAGGGAAAGGAGGCUGUACACAGUGAUCUAGGCUACUGGAUUGAGAGUGAGAAGAAUGACACAUGGAGGCUGAAGAGGGCCCAUCUGAUCGAGAUGAAAACAUGUGAAUGGCCAAAGUCCCACACAUUGUG
KU761564    GAGAAGACUAUUCAUUAGAGUGUGAUCCAGCCGUUAUUGGAACAGCUGUUAAGGGAAAGGAGGCUGUACACAGUGAUCUAGGCUACUGGAUUGAGAGUGAGAAGAAUGACACAUGGAGGCUGAAGAGGGCCCAUCUGAUCGAGAUGAAAACAUGUGAAUGGCCAAAGUCCCACACAUUGUG
KU740184    GAGAAGACUAUUCAUUAGAGUGUGAUCCAGCCGUUAUUGGAACAGCUGUUAAGGGAAAGGAGGCUGUACACAGUGAUCUAGGCUACUGGAUUGAGAGUGAGAAGAAUGACACAUGGAGGCUGAAGAGGGCCCAUCUGAUCGAGAUGAAAACAUGUGAAUGGCCAAAGUCCCACACAUUGUG
KU744693    GAGAAGACUAUUGGUUAGAGUGUGAUCCAGCCGUUAUUGGAACAGCUGUUAAGGGAAAGGAGGCUGUACACAGUGAUCUAGGCUACUGGAUUGAGAGUGAGAAGAAUGACACAUGGUGGCUGAAGAGGGCCCAUCUGAUCGAGAUGAAAACAUGUGAAUGGCCAAAGUCCCACACAUUGUG
KU497555    GAGAAGAUUAUUCAUUAGAGUGUGAUCCAGCCGUUAUUGGAACAGCUGUUAAGGGAAAGGAGGCUGUACACAGUGAUCUAGGCUACUGGAUUGAGAGUGAGAAGAAUGACACAUGGAGGCUGAAGAGGGCCCAUCUGAUCGAGAUGAAAACAUGUGAAUGGCCAAAGUCCCACACAUUGUG
KU527068    GAGAAGAUUAUUCAUUAGAGUGUGAUCCAGCCGUUAUUGGAACAGCUGUUAAGGGGAAGGAGGCUGUACACAGUGAUCUAGGCUACUGGAUUGAGAGUGAGAAGAAUGACACAUGGAGGCUGAAGAGGGCCCAUCUAAUCGAGAUGAAAACAUGUGAAUGGCCAAAGUCCCACACAUUGUG
KU501217    GAGAAGAUUAUUCAUUAGAGUGUGAUCCAGCCGUUAUUGGAACAGCUGUUAAGGGAAAGGAGGCUGUACACAGUGAUCUAGGCUACUGGAUUGAGAGUGAGAAGAAUGACACAUGGAGGCUGAAGAGGGCCCAUCUGAUCGAGAUGAAAACAUGUGAAUGGCCAAAGUCCCACACAUUGUG
KU501216    GAGAAGAUUAUUCAUUAGAGUGUGAUCCAGCCGUUAUUGGAACAGCUGUUAAGGGAAAGGAGGCUGUACACAGUGAUCUAGGCUACUGGAUUGAGAGUGAGAAGAAUGACACAUGGAGGCUGAAGAGGGCCCAUCUGAUCGAGAUGAAAACAUGUGAAUGGCCAAAGUCCCACACAUUGUG
KU501215    GAGAAGAUUAUUCAUUAGAGUGUGAUCCAGCCGUUAUUGGAACAGCUGUUAAGGGAAAGGAGGCUGUACACAGUGAUCUAGGCUACUGGAUUGAGAGUGAGAAGAAUGACACAUGGAGGCUGAAGAGGGCCCAUCUGAUCGAGAUGAAAACAUGUGAAUGGCCAAAGUCCCACACAUUGUG
KU365780    GAGAAGAUUAUUCAUUAGAGUGUGAUCCAGCCGUUAUUGGAACAGCUGUUAAGGGAAAGGAGGCUGUACACAGUGAUCUAGGCUACUGGAUUGAGAGUGAGAAGAAUGACACAUGGAGGCUGAAGAGGGCCCAUCUGAUCGAGAUGAAAACAUGUGAAUGGCCAAAGUCCCACACAUUGUG
KU365779    GAGAAGAUUAUUCAUUAGAGUGUGAUCCAGCCGUUAUUGGAACAGCUGUUAAGGGAAAGGAGGCUGUACACAGUGAUCUAGGCUACUGGAUUGAGAGUGAGAAGAAUGACACAUGGAGGCUGAAGAGGGCCCAUCUGAUCGAGAUGAAAACAUGUGAAUGGCCAAAGUCCCACACAUUGUG
KU707826    GAGAAGAUUAUUCAUUAGAGUGUGAUCCAGCCGUUAUUGGAACAGCUGUUAAGGGAAAGGAGGCUGUACACAGUGAUCUAGGCUACUGGAUUGAGAGUGAGAAGAAUGACACAUGGAGGCUGAAGAGGGCCCAUCUGAUCGAGAUGAAAACAUGUGAAUGGCCAAAGUCCCACACAUUGUG
KU365778    GAGAAGAUUAUUCAUUAGAGUGUGAUCCAGCCGUUAUUGGAACAGCUGUUAAGGGAAAGGAGGCUGUACACAGUGAUCUAGGCUACUGGAUUGAGAGUGAGAAGAAUGACACAUGGAGGCUGAAGAGGGCCCAUCUGAUCGAGAUGAAAACAUGUGAAUGGCCAAAGUCCCACACAUUGUG
KU365777    GAGAAGAUUAUUCAUUAGAGUGUGAUCCAGCCGUUAUUGGAACAGCUGUUAAGGGAAAGGAGGCUGUACACAGUGAUCUAGGCUACUGGAUUGAGAGUGAGAAGAAUGACACAUGGAGGCUGAAGAGGGCCCAUCUGAUCGAGAUGAAAACAUGUGAAUGGCCAAAGUCCCACACAUUGUG
KU312312    GAGAAGAUUAUUCAUUAGAGUGUGAUCCAGCCGUUAUUGGAACAGCUGUUAAGGGAAAGGAGGCUGUACACAGUGAUCUAGGCUACUGGAUUGAGAGUGAGAAGAAUGACACAUGGAGGCUGAAGAGGGCCCAUCUGAUCGAGAUGAAAACAUGUGAAUGGCCAAAGUCCCACACAUUGUG
KF268950    GAGAAGACUACUCACUAGAAUGUGACCCAGCCGUCAUAGGAACAGCUGUUAAGGGAAAGGAGGCCGCCCACAGUGACCUGGGCUACUGGAUUGAAAGUGAAAAGAAUGACACAUGGAGGCUGAAGAGGGCCCAUCUGAUUGAGAUGAAAACAUGUGAGUGGCCAAAGUCUCACACUCUGUG
KF268949    GAGAAGAUUACUCACUAGAAUGUGACCCAGCCGUCAUAGGAACAGCUGUUAAGGGAAAGGAGGCCGCGCACAGUGACCUAGGCUACUGGAUUGAAAGUGAAAAGAAUGACACAUGGAGGCUGAAGAGGGCCCACCUGAUUGAGAUGAAAACAUGUGAGUGGCCAAAGUCUCACACUCUGUG
KF268948    GAGAAGACUACUCACUAGAAUGUGACCCAGCCGUCAUAGGAACAGCUGUUAAGGGAAAGGAGGCCGCCCACAGUGACCUGGGCUACUGGAUUGAAAGUGAAAAGAAUGACACAUGGAGGCUGAAGAGGGCCCAUCUGAUUGAGAUGAAAACAUGUGAGUGGCCAAAGUCUCACACUCUGUG
KJ776791    GAGAAGAUUAUUCAUUAGAGUGUGAUCCAGCCGUUAUUGGAACAGCUGUUAAGGGAAAGGAGGCUGUACACAGUGAUCUAGGCUACUGGAUUGAGAGUGAGAAGAAUGACACAUGGAGGCUGAAGAGGGCCCAUCUGAUCGAGAUGAAAACAUGUGAAUGGCCAAAGUCCCACACAUUGUG
KF383119    GAGAAGAUUACUCAUUAGAAUGUGACCCAGCCGUCAUAGGAACAGCUGUUAAGGGAAGGGAGGCCGCGCACAGUGAUCUGGGCUAUUGGAUUGAAAGUGAAAAGAAUGACACAUGGAGGCUGAAGAGGGCCCACCUGAUUGAGAUGAAAACAUGUGAAUGGCCAAAGUCUCACACAUUGUG
KF383118    GAGAAGAUUACUCAUUAGAAUGUGACCCAGCCGUCAUAGGAACAGCUGUUAAGGGAAGGGAGGCCGCGCACAGUGAUCUGGGCUAUUGGAUUGAAAGUGAAAAGAAUGACACAUGGAGGCUGAAGAGGGCCCACCUGAUUGAGAUGAAAACAUGUGAAUGGCCAAAGUCUCACACAUUGUG
KF383117    GAGAGGACUACUCAUUAGAGUGUGACCCAGCCGUCAUAGGAACAGCUGUCAAGGGAAAGGAAGCUGCACACAGUGAUCUAUGCUAUUGGAUUGAGAGUGAAAAGAAUGACACAUGGAGGCUGAGGAGGGCCCACCUGAUUGAGAUGAAAACAUGUGAAUGGCCAAAGUCUCACACAUUGUG
KF383116    GAGAGGACUACUCAUUAGAGUGUGACCCAGCCGUCAUAGGAACAGCUGUCAAGGGAAAGGAAGCUGCACACAGUGAUCUAGGCUAUUGGAUUGAGAGUGAAAAGAAUGACACAUGGAGGCUGAGGAGGGCUCAUCUGAUUGAGAUGAAAACAUGUGAGUGGCCAAAGUCUCACACACUGUG
KF383115    GAGAAGAUUACUCACUAGAGUGUGACCCAGCCGUCAUAGGAACAGCUGUUAAGGGAAAGGAGGCCGCGCACAGUGAUCUAGGCUACUGGAUUGAAAGUGAAAAGAAUGACACAUGGAGGUUGAAGAGGGCCCACCUGAUUGAGAUGAAAACAUGUGAGUGGCCAAAGUCUCACACACUGUG
EU545988    GAGAAGAUUAUUCAUUAGAGUGUGAUCCAGCCGUUAUUGGAACAGCUGCUAAGGGAAAGGAGGCUGUGCACAGUGAUCUAGGCUACUGGAUUGAGAGUGAGAAGAAUGACACAUGGAGGCUGAAGAGGGCCCACCUGAUCGAGAUGAAAACAUGUGAAUGGCCAAAGUCCCACACAUUGUG
KU955589    GAGAAGAUUAUUCAUUAGAGUGUGAUCCAGCCGUUAUUGGAACAGCUGUUAAGGGAAAGGAGGCUGUACACAGUGAUCUAGGCUACUGGAUUGAGAGUGAGAAGAAUGACACAUGGAGGCUGAAGAGGGCCCAUCUGAUCGAGAUGAAAACAUGUGAAUGGCCAAAGUCCCACACAUUGUG
KU955590    GAGAAGAUUAUUCAUUAGAGUGUGAUCCAGCCGUUAUUGGAACAGCUGUUAAGGGAAAGGAGGCUGUACACAGUGAUCUAGGCUACUGGAUUGAGAGUGAGAAGAAUGACACAUGGAGGCUGAAGAGGGCCCAUCUGAUCGAGAUGAAAACAUGUGAAUGGCCAAAGUCCCACACAUUGUG
KU940228    GAGAAGAUUAUUCAUUAGAGUGUGAUCCAGCCGUUAUUGGAACAGCUGUUAAGGGAAAGGAGGCUGUACACAGUGAUCUAGGCUACUGGAUUGAGAGUGAGAAGAAUGACACAUGGAGGCUGAAGAGGGCCCAUCUGAUCGAGAUGAAAACAUGUGAAUGGCCAAAGUCCCACACAUUGUG
KU991811    GAGAAGAUUAUUCAUUAGAGUGUGAUCCAGCCGUUAUUGGAACAGCUGUUAAGGGAAAGGAGGCUGUACACAGUGAUCUAGGCUACUGGAUUGAGAGUGAGAAGAAUGACACAUGGAGGCUGAAGAGGGCCCAUCUGAUCGAGAUGAAAACAUGUGAAUGGCCAAAGUCCCACACAUUGUG
KU509998    GAGAAGAUUAUUCAUUAGAGUGUGAUCCAGCCGUUAUUGGAACAGCUGUUAAGGGAAAGGAGGCUGUACACAGUGAUCUAGGCUACUGGAUUGAGAGUGAGAAGAAUGACACAUGGAGGCUGAAGAGGGCCCAUCUGAUCGAGAUGAAAACAUGUGAAUGGCCAAAGUCCCACACAUUGUG
KX056898    GAGAAGAUUAUUCAUUAGAGUGUGAUCCAGCCGUUAUUGGAACAGCUGUUAAGGGAAAGGAGGCUGUACACAGUGAUCUAGGCUACUGGAUUGAGAGUGAGAAGAAUGACACAUGGAGGCUGAAGAGGGCCCAUCUGAUCGAGAUGAAAACAUGUGAAUGGCCAAAGUCCCACACAUUGUG
KX117076    GAGAAGAUUAUUCAUUAGAGUGUGAUCCAGCCGUUAUUGGAACAGCUGUUAAGGGAAAGGAGGCUGUACACAGUGAUCUAGGCUACUGGAUUGAGAGUGAGAAGAAUGACACAUGGAGGCUGAAGAGGGCCCAUCUGAUCGAGAUGAAAACAUGUGAAUGGCCAAAGUCCCACACAUUGUG
KX185891    GAGAAGAUUAUUCAUUAGAGUGUGAUCCAGCCGUUAUUGGAACAGCUGUUAAGGGAAAGGAGGCUGUACACAGUGAUCUAGGCUACUGGAUUGAGAGUGAGAAGAAUGACACAUGGAGGCUGAAGAGGGCCCAUCUGAUCGAGAUGAAAACAUGUGAAUGGCCAAAGUCCCACACAUUGUG
KX247646    GAGAAGAUUAUUCAUUAGAGUGUGAUCCAGCCGUUAUUGGAACAGCUGUUAAGGGAAAGGAGGCUGUACACAGUGAUCUAGGCUACUGGAUUGAGAGUGAGAAGAAUGACACAUGGAGGCUGAAGAGGGCCCAUCUGAUCGAGAUGAAAACAUGUGAAUGGCCAAAGUCCCACACAUUGUG
KX262887    GAGAAGAUUAUUCAUUAGAGUGUGAUCCAGCCGUUAUUGGAACAGCUGUUAAGGGAAAGGAGGCUGUACACAGUGAUCUAGGCUACUGGAUUGAGAGUGAGAAGAAUGACACAUGGAGGCUGAAGAGGGCCCAUCUGAUCGAGAUGAAAACAUGUGAAUGGCCAAAGUCCCACACAUUGUG
KX280026    GAGAAGAUUAUUCAUUAGAGUGUGAUCCAGCCGUUAUUGGAACAGCUGUUAAGGGAAAGGAGGCUGUACACAGUGAUCUAGGCUACUGGAUUGAGAGUGAGAAGAAUGACACAUGGAGGCUGAAGAGGGCCCACCUGAUCGAGAUGAAAACAUGUGAAUGGCCAAAGUCCCACACAUUGUG
KU758877    GAGAAGAUUAUUCAUUAGAGUGUGAUCCAGCCGUUAUUGGAACAGCUGUUAAGGGAAAGGAGGCUGUACACAGUGAUCUAGGCUACUGGAUUGAGAGUGAGAAGAAUGACACAUGGAGGCUGAAGAGGGCCCAUCUGAUCGAGAUGAAAACAUGUGAAUGGCCAAAGUCCCACACAUUGUG
KU866423    GAGAAGAUUAUUCAUUAGAGUGUGAUCCAGCCGUUAUUGGAACAGCUGUUAAGGGAAAGGAGGCUGUACACAGUGAUCUAGGCUACUGGAUUGAGAGUGAGAAGAAUGACACAUGGAGGCUGAAGAGGGCCCAUCUGAUCGAGAUGAAAACAUGUGAAUGGCCAAAGUCCCACACAUUGUG
KX377336    GAGAGGAUUACUCAUUAGAGUGUGAUCCAGCCGUCAUAGGAACAGCUGCUAAGGGAAAGGAGGCCGUGCACAGUGAUCUAGGCUACUGGAUUGAGAGUGAAAAGAACGACACAUGGAGGCUGAAGAGGGCUCACCUGAUCGAGAUGAAAACAUGUGAAUGGCCAAAGUCCCACACACUGUG
KX377337    GAGAAGAUUAUUCAUUAGAGUGUGAUCCAGCCGUUAUUGGAACAGCUGUUAAGGGAAAGGAGGCUGUACACAGUGAUCUAGGCUACUGGAUUGAGAGUGAGAAGAAUGACACAUGGAGGCUGAAGAGGGCCCAUCUGAUCGAGAUGAAAACAUGUGAAUGGCCAAAGUCCCACACAUUGUG
KX369547    GAGAAGAUUAUUCAUUAGAGUGUGAUCCAGCCGUUAUUGGAACAGCUGUUAAGGGAAAGGAGGCUGUACACAGUGAUCUAGGCUACUGGAUUGAGAGUGAGAAGAAUGACACAUGGAGGCUGAAGAGGGCCCAUCUGAUCGAGAUGAAAACAUGUGAAUGGCCAAAGUCCCACACAUUGUG
KX548902    GAGAAGAUUAUUCAUUAGAGUGUGAUCCAGCCGUUAUUGGAACAGCUGUUAAGGGAAAGGAGGCUGUACACAGUGAUCUAGGCUACUGGAUUGAGAGUGAGAAGAAUGACACAUGGAGGCUGAAGAGGGCCCAUCUGAUCGAGAUGAAAACAUGUGAAUGGCCAAAGUCCCACACAUUGUG
KX601166    GAGAGGACUACUCACUAGAGUGUGACCCAGCCGUCAUAGGAACAGCUGUCAAGGGAAAGGAAGCUGCACACAGUGAUCUAGGCUAUUGGAUUGAGAGUGAAAAGAAUGACACAUGGAGGCUGAGGAGGGCUCAUCUGAUUGAGAUGAAAACAUGUGAGUGGCCAAAGUCUCACACACUGUG
KX601167    GAGAGGAUUACUCAUUAGAGUGUGAUCCAGCCGUCAUAGGAACAGCUGCUAAGGGAAAGGAGGCCGUGCACAGUGAUCUAGGCUACUGGAUUGAGAGUGAAAAGAACGACACAUGGAGGCUGAAGAGGGCUCACCUGAUCGAGAUGAAAACAUGUGAAUGGCCAAAGUCCCACACACUGUG
KX601168    GAGAAGAUUAUUCAUUAGAGUGUGAUCCAGCCGUUAUUGGAACAGCUGUUAAGGGAAAGGAGGCUGUACACAGUGAUCUAGGCUACUGGAUUGAGAGUGAGAAGAAUGACACAUGGAGGCUGAAGAGGGCCCAUCUGAUCGAGAUGAAAACAUGUGAAUGGCCAAAGUCCCACACAUUGUG
KX766028    GAGAAGAUUAUUCAUUAGAGUGUGAUCCAGCCGUUAUUGGAACAGCUGUUAAGGGAAAGGAGGCUGUACACAGUGAUCUAGGCUACUGGAUUGAGAGUGAGAAGAAUGACACAUGGAGGCUGAAGAGGGCCCAUCUGAUCGAGAUGAAAACAUGUGAAUGGCCAAAGUCCCACACAUUGUG
KX766029    GAGAAGAUUAUUCAUUAGAGUGUGAUCCAGCCGUUAUUGGAACAGCUGUCAAGGGAAAGGAGGCUGUACACAGUGAUCUAGGCUACUGGAUUGAGAGUGAGAAGAAUGACACAUGGAGGCUGAAGAGGGCCCAUCUGAUCGAGAUGAAAACAUGUGAAUGGCCAAAGUCCCACACAUUGUG
KU940224    GAGAAGAUUAUUCAUUAGAGUGUGAUCCAGCCGUUAUUGGAACAGCUGUUAAGGGAAAGGAGGCUGUACACAGUGAUCUAGGCUACUGGAUUGAGAGUGAGAAGAAUGACACAUGGAGGCUGAAGAGGGCCCAUCUGAUCGAGAUGAAAACAUGUGAAUGGCCAAAGUCCCACACAUUGUG


            3333333333333333333333333333333333333333333333333333333333333333333333333333333333333333333333333333333333333333333333333333333333333333333333333333333333333333333333333333333
            0000000000000000000000111111111111111111111111111111111111111111111111111111111111111111111111111111111111111111111111111111122222222222222222222222222222222222222222222222222222222222
            7788888888889999999999000000000011111111112222222222333333333344444444445555555555666666666677777777778888888888999999999900000000001111111111222222222233333333334444444444555555555
Sequence    8901234567890123456789012345678901234567890123456789012345678901234567890123456789012345678901234567890123456789012345678901234567890123456789012345678901234567890123456789012345678
-----------------------------------------------------------------------------------------------------------------------------------------------------------------------------------------------------------
NC_012532   GACAGAUGGAGUAGAAGAAAGUGAUCUUAUCAUACCCAAGUCUUUAGCUGGUCCACUCAGCCACCACAACACCAGAGAGGGUUACAGAACCCAAGUGAAAGGGCCAUGGCACAGUGAAGAGCUUGAAAUCCGGUUUGAGGAAUGUCCAGGCACCAAGGUUUACGUGGAGGAGACAUGCGGA
KU922960    GACAGAUGGAAUAGAAGAGAGUGAUCUGAUCAUACCCAAGUCUUUAGCUGGGCCACUCAGCCAUCACAAUACCAGAGAGGGCUACAGGACCCAAAUGAAAGGGCCAUGGCACAGUGAAGAGCUUGAAAUUCGGUUUGAGGAAUGCCCAGGCACUAAGGUCCACGUGGAGGAAACAUGUGGA
KU922923    GACAGAUGGAAUAGAAGAGAGUGAUCUGAUCAUACCCAAGUCUUUAGCUGGGCCACUCAGCCAUCACAAUACCAGAGAGGGCUACAGGACCCAAAUGAAAGGGCCAUGGCACAGUGAAGAGCUUGAAAUUCGGUUUGAGGAAUGCCCAGGCACUAAGGUCCACGUGGAGGAAACAUGUGGA
KU820898    GACAGAUGGAAUAGAAGAGAGUGAUCUGAUCAUACCCAAGUCUUUAGCUGGGCCACUCAGCCAUCACAAUACCAGAGAGGGCUACAGGACCCAAAUGAAAGGGCCAUGGCACAGUGAGGAGCUUGAAAUUCGGUUUGAGGAAUGCCCAGGCACUAAGGUCCACGUGGAGGAAACAUGUGGA
KU321639    GACAGAUGGAAUAGAAGAGAGUGAUCUGAUCAUACCCAAGUCUUUAGCUGGGCCACUCAGCCAUCACAAUACCAGAGAGGGCUACAGGACCCAAAUGAAAGGGCCAUGGCACAGUGAAGAGCUUGAAAUUCGGUUUGAGGAAUGCCCAGGCACUAAGGUCCACGUGGAGGAAACAUGUGGA
KU820899    GACAGAUGGAAUAGAAGAGAGUGAUCUGAUCAUACCCAAGUCUUUAGCUGGGCCACUCAGCCAUCACAAUACCAGAGAGGGCUACAGGACCCAAAUGAAAGGGCCAUGGCACAGUGAAGAGCUUGAAAUUCGGUUUGAGGAAUGCCCAGGCACCAAGGUCCACGUGGAGGAAACAUGUGGA
KU853012    GACAGAUGGAAUAGAAGAGAGUGAUCUGAUCAUACCCAAGUCUUUAGCUGGGCCACUCAGCCAUCACAAUACCAGAGAGGGCUACAGGACCCAAAUGAAAGGGCCAUGGCACAGUGAAGAGCUUGAAAUUCGGUUUGAGGAAUGCCCAGGCACUAAGGUCCACGUGGAGGAAACAUGUGGA
KU729217    GACAGAUGGAAUAGAAGAGAGUGAUCUGAUCAUACCCAAGUCUUUAGCUGGGCCACUCAGCCAUCACAAUACCAGAGAGGGCUACAGGACCCAAAUGAAAGGGCCAUGGCACAGUGAAGAGCUUGAAAUUCGGUUUGAGGAAUGCCCAGGCACUAAGGUCCACGUGGAGGAAACAUGUGGA
KU729218    GACAGAUGGAAUAGAAGAGAGUGAUCUGAUCAUACCCAAGUCUUUAGCUGGGCCACUCAGCCAUCACAAUACCAGAGAGGGCUACAGGACCCAAAUGAAAGGGCCAUGGCACAGUGAAGAGCUUGAAAUUCGGUUUGAGGAAUGCCCAGGCACUAAGGUCCACGUGGAGGAAACAUGUGGA
KU761564    GACAGAUGGAAUAGAAGAGAGUGAUCUGAUCAUACCCAAGUCUUUAGCUGGGCCACUCAGCCAUCACAAUACCAGAGAGGGCUACAGGACCCAAAUGAAAGGGCCAUGGCACAGUGAGGAGCUUGAAAUUCGGUUUGAGGAAUGCCCAGGCACUAAGGUCCACGUGGAGGAAACAUGUGGA
KU740184    GACAGAUGGAAUAGAAGAGAGUGAUCUGAUCAUACCCAAGUCUUUAGCUGGGCCACUCAGCCAUCACAAUACCAGAGAGGGCUACAGGACCCAAAUGAAAGGGCCAUGGCACAGUGAGGAGCUUGAAAUUCGGUUUGAGGAAUGCCCAGGCACUAAGGUCCACGUGGAGGAAACAUGUGGA
KU744693    GACAGAUGGAAUAGAAGAGAGUGAUCUGAUCAUACCCAAGUCUUUAGCUGGGCCACUCAGCCAUCACAAUGCCAGAGAGGGCUACAGGACCCAAAUGAAAGGGCCAUGGCACAGUGAAGAGCUUGAAAUUCGGUUUGAGGAAUGCCCAGGCACUAAGGUCCACGUGGAGGAAACAUGUGGA
KU497555    GACAGAUGGAAUAGAAGAGAGUGAUCUGAUCAUACCCAAGUCUUUAGCUGGGCCACUCAGCCAUCACAAUACCAGAGAGGGCUACAGGACCCAAAUGAAAGGGCCAUGGCACAGUGAAGAGCUUGAAAUUCGGUUUGAGGAAUGCCCAGGCACUAAGGUCCACGUGGAGGAAACAUGUGGA
KU527068    GGCAGAUGGAAUAGAAGAGAGUGAUCUGAUCAUUCCCAAGUCUUUAGCUGGGCCACUCAGCCAUCACAAUACCAGAGAGGGCUACAGGACCCAAAUGAAAGGGCCAUGGCACAGUGAAGAGCUUGAAAUUCGGUUUGAGGAAUGCCCGGGCACUAAGGUCCACGUGGAGGAAACAUGUGGA
KU501217    GACAGAUGGAAUAGAAGAGAGUGAUCUGAUCAUACCCAAGUCUUUAGCUGGGCCACUCAGCCAUCACAAUACCAGAGAGGGCUACAGGACCCAAAUGAAAGGGCCAUGGCACAGUGAAGAGCUUGAAAUUCGGUUUGAGGAAUGCCCAGGCACUAAGGUCCACGUGGAGGAAACAUGUGGA
```

```
KU501216    GACAGAUGGAAUAGAAGAGAGUGAUCUGAUCAUACCCAAGUCUUUAGCUGGGCCACUCUCAGCCAUCACAAUACCAGAGAGGGCUACAGGACCCAAAUGAAAGGGCCAUGGCACAGUGAAGAGCUUGAAAUUCGGUUUGAGGAAUGCCCAGGCACUAAGGUCCACGUGGAGGAAACAUGUGGA
KU501215    GACAGAUGGAAUAGAAGAGAGUGAUCUGAUCAUACCCAAGUCUUUAGCUGGGCCACUCUCAGCCAUCACAAUACCAGAGAGGGCUACAGGACCCAAAUGAAAGGGCCAUGGCACAGUGAAGAGCUUGAAAUUCGGUUUGAGGAAUGCCCAGGCACUAAGGUCCACGUGGAGGAAACAUGUGGA
KU365780    GACAGAUGGAAUAGAAGAGAGUGAUCUGAUCAUACCCAAGUCUUUAGCUGGGCCACUCUCAGCCAUCACAAUACCAGAGAGGGCUACAGGACCCAAAUGAAAGGGCCAUGGCACAGUGAAGAGCUUGAAAUUCGGUUUGAGGAAUGCCCAGGCACUAAGGUCCACGUGGAGGAAACAUGUGGA
KU365779    GACAGAUGGAAUAGAAGAGAGUGAUCUGAUCAUACCCAAGUCUUUAGCUGGGCCACUCUCAGCCAUCACAAUACCAGAGAGGGCUACAGGACCCAAAUGAAAGGGCCAUGGCACAGUGAAGAGCUUGAAAUUCGGUUUGAGGAAUGCCCAGGCACUAAGGUCCACGUGGAGGAAACAUGUGGA
KU707826    GACAGAUGGAAUAGAAGAGAGUGAUCUGAUCAUACCCAAGUCUUUAGCUGGGCCACUCUCAGCCAUCACAAUACCAGAGAGGGCUACAGGACCCAAAUGAAAGGGCCAUGGCACAGUGAAGAGCUUGAAAUUCGGUUUGAGGAAUGCCCAGGCACUAAGGUCCACGUGGAGGAAACAUGUGGA
KU365778    GACAGAUGGAAUAGAAGAGAGUGAUCUGAUCAUACCCAAGUCUUUAGCUGGGCCACUCUCAGCCAUCACAAUACCAGAGAGGGCUACAGGACCCAAAUGAAAGGGCCAUGGCACAGUGAAGAGCUUGAAAUUCGGUUUGAGGAAUGCCCAGGCACUAAGGUCCACGUGGAGGAAACAUGUGGA
KU365777    GACAGAUGGAAUAGAAGAGAGUGAUCUGAUCAUACCCAAGUCUUUAGCUGGGCCACUCUCAGCCAUCACAAUACCAGAGAGGGCUACAGGACCCAAAUGAAAGGGCCAUGGCACAGUGAAGAGCUUGAAAUUCGGUUUGAGGAAUGCCCAGGCACUAAGGUCCACGUGGAGGAAACAUGUGGA
KU312312    GACAGAUGGAAUAGAAGAGAGUGAUCUGAUCAUACCCAAGUCUUUAGCUGGGCCACUCUCAGCCAUCACAAUACCAGAGAGGGCUACAGGACCCAAAUGAAAGGGCCAUGGCACAGUGAAGAGCUUGAAAUUCGGUUUGAGGAAUGCCCAGGCACUAAGGUCCACGUGGAGGAAACAUGUGGA
KF268950    GACAGAUGGAGUAGAAGAAAGUGAUCUGAUCAUACCUAAAUCUUUAGCUGGUCCACUCUCAGCCACCACAACACUAGAGAGGGUUACAGAACUCAAGCAAAAGGGCCAUGGCAUAGUGAAGAGCUUGAAAUCCGGUUUGAGGAAUGUCCAGGCACCAAGGUUCACGUGGAGGAGACAUGCGGA
KF268949    GACAGAUGGAGUAGAAGAAAGUGAUCUGAUCAUACCUAAAUCUUUAGCUGGUCCACUCUCAGCCACCACAACACUAGAGAGGGUUACAGAACUCAAGCAAAAGGGCCAUGGCAUAGUGAAGAGCUUGAAAUCCGGUUUGAGGAAUGUCCAGGCACCAAGGUUCAUGUGGAGGAGACAUGCGGA
KF268948    GACAGAUGGAGUAGAAGAAAGUGAUCUGAUCAUACCUAAAUCUUUAGCUGGUCCACUCUCAGCCACCACAACACUAGAGAGGGUUACAGAACUCAAGCAAAAGGGCCAUGGCAUAGUGAAGAGCUUGAAAUCCGGUUUGAGGAAUGUCCAGGCACCAAGGUUCACGUGGAGGAGACAUGCGGA
KJ776791    GACAGAUGGAAUAGAAGAGAGUGAUCUGAUCAUACCCAAGUCUUUAGCUGGGCCACUCUCAGCCAUCACAAUACCAGAGAGGGCUACAGGACCCAAAUGAAAGGGCCAUGGCACAGUGAAGAGCUUGAAAUUCGGUUUGAGGAAUGCCCAGGCACUAAGGUCCACGUGGAGGAAACAUGUGGA
KF383119    GACAGAUGGAGUAGAAGAAAGUGAUCUUAUCAUACCCAAGUCUUUAGCUGGUCCACUCUCAGCCACCACAACACCAGAGAGGGUUACAGAACCCAAGUGAAAGGGCCAUGGCACAGUGAAGAGCUUGAAAUCCGGUUUGAGGAAUGUCCAGGCACCAAGGUUCACGUGGAGGAGACAUGCGGA
KF383118    GACAGAUGGAGUAGAAGAAAGUGAUCUUAUCAUACCCAAGUCUUUAGCUGGUCCACUCUCAGCCACCACAACACCAGAGAGGGUUACAGAACCCAAGUGAAAGGGCCAUGGCACAGUGAAGAGCUUGAAAUCCGGUUUGAGGAAUGUCCAGGCACCAAGGUUCACGUGGAGGAGACAUGCGGA
KF383117    GACAGAUGGAGUAGAAGAAAGUGAUCUUAUCAUACCCAAGUCUUUAGCUGGUCCACUCUCAGCCACCACAACACCAGAGAGGGUUACAGAACCCAAGUGAAAGGGCCAUGGCACAGUGAAGAGCUUGAAAUCCGGUUUGAGGAAUGUCCAGGCACCAAGGUUUACGUGGAGGAGACAUGCGGA
KF383116    GACAGAUGGAGUGGAAGAAAGUGAUCUGAUCAUACCCAAGUCCUUAGCUGGUCCACUCUCAGCCACCACAACACCAGAGAGGGUUACAGAACUCAAGUGAAAGGGCCAUGGCAUAGUGAAGAGCUCGAAAUCCGGUUUGAGGAGUGCCCAGGAACCAAGGUUCACGUGGAGGAGACAUGCGGA
KF383115    GACAGAUGGAGUAGAAGAAAGUGAUCUGAUCAUACCCAAGUCUUUAGCUGGUCCACUCUCAGCCACCACAACACCAGAGAGGGUUACAGAACUCAGGCAAAAGGGCCAUGGCAUAGUGAAGAGCUUGAAAUCCGGUUUGAGGAAUGUCCAGGCACCAAGGUUCACGUGGAGGAGACAUGCGGA
EU545988    GACAGAUGGAAUAGAAGAAAGUGAUCUGAUCAUACCCAAGUCUUUAGCUGGGCCACUCUCAGCCAUCACAACACCAGAGAGGGCUACAGGACUCAAAUGAAAGGGCCAUGGCACAGUGAAGAGCUUGAAAUUCGGUUUGAGGAAUGCCCAGGCACUAAGGUCCACGUGGAGGAAACAUGUGGA
KU955589    GACAGAUGGAAUAGAAGAGAGUGAUCUGAUCAUACCCAAGUCUUUAGCUGGGCCACUCUCAGCCAUCACAAUACCAGAGAGGGCUACAGGACCCAAAUGAAAGGGCCAUGGCACAGUGAAGAGCUUGAAAUUCGGUUUGAGGAAUGCCCAGGCACCAAGGUCCACGUGGAGGAAACAUGUGGA
KU955590    GACAGAUGGAAUAGAAGAGAGUGAUCUGAUCAUACCCAAGUCUUUAGCUGGGCCACUCUCAGCCAUCACAAUACCAGAGAGGGCUACAGGACCCAAAUGAAAGGGCCAUGGCACAGUGAAGAGCUUGAAAUUCGGUUUGAGGAAUGCCCAGGCACUAAGGUCCACGUGGAGGAAACAUGUGGA
KU940228    GACAGAUGGAAUAGAAGAGAGUGAUCUGAUCAUACCCAAGUCUUUAGCUGGGCCACUCUCAGCCAUCACAAUACCAGAGAGGGCUACAGGACCCAAAUGAAAGGGCCAUGGCACAGUGAAGAGCUUGAAAUUCGGUUUGAGGAAUGCCCAGGCACUAAGGUCCACGUGGAGGAAACAUGUGGA
KU991811    GACAGAUGGAAUAGAAGAGAGUGAUCUGAUCAUACCCAAGUCUUUAGCUGGGCCACUCUCAGCCAUCACAAUACCAGAGAGGGCUACAGGACCCAAAUGGAAGGGCCAUGGCACAGUGAAGAGCUUGAAAUUCGGUUUGAGGAAUGCCCAGGCACUAAGGUCCACGUGGAGGAAACAUGUGGA
KU509998    GACAGAUGGAAUAGAAGAGAGUGAUCUGAUCAUACCCAAGUCUUUAGCUGGGCCACUCUCAGCCAUCACAAUACCAGAGAGGGCUACAGGACCCAAAUGAAAGGGCCAUGGCACAGUGAAGAGCUUGAAAUUCGGUUUGAGGAAUGCCCAGGCACUAAGGUCCACGUGGAGGAAACAUGUGGA
KX056898    GACAGAUGGAAUAGAAGAGAGUGAUCUGAUCAUACCCAAGUCUUUAGCUGGGCCACUCUCAGCCAUCACAAUACCAGAGAGGGCUACAGGACCCAAAUGAAAGGGCCAUGGCACAGUGAAGAGCUUGAAAUUCGGUUUGAGGAAUGCCCAGGCACUAAGGUCCACGUGGAGGAAACAUGUGGA
KX117076    GACAGAUGGAAUAGAAGAGAGUGAUCUGAUCAUACCCAAGUCUUUAGCUGGGCCACUCUCAGCCAUCACAAUACCAGAGAGGGCUACAGGACCCAAAUGAAAGGGCCAUGGCACAGUGAAGAGCUUGAAAUUCGGUUUGAGGAAUGCCCAGGCACCAAGGUCCACGUGGAGGAAACAUGUGGA
KX185891    GACAGAUGGAAUAGAAGAGAGUGAUCUGAUCAUACCCAAGUCUUUAGCUGGGCCACUCUCAGCCAUCACAAUACCAGAGAGGGCUACAGGACCCAAAUGAAAGGGCCAUGGCACAGUGAAGAGCUUGAAAUUCGGUUUGAGGAAUGCCCAGGCACCAAGGUCCACGUGGAGGAAACAUGUGGA
KX247646    GACAGAUGGAAUAGAAGAGAGUGAUCUGAUCAUACCCAAGUCUUUAGCUGGGCCACUCUCAGCCAUCACAAUACCAGAGAGGGCUACAGGACCCAAAUGAAAGGGCCAUGGCACAGUGAAGAGCUUGAAAUUCGGUUUGAGGAAUGCCCAGGCACUAAGGUCCACGUGGAGGAAACAUGUGGA
KX262887    GACAGAUGGAAUAGAAGAGAGUGAUCUGAUCAUACCCAAGUCUUUAGCUGGGCCACUCUCAGCCAUCACAAUACCAGAGAGGGCUACAGGACCCAAAUGAAAGGGCCAUGGCACAGUGAAGAGCUUGAAAUUCGGUUUGAGGAAUGCCCAGGCACUAAGGUCCACGUGGAGGAAACAUGUGGA
KX280026    GACAGAUGGAAUAGAAGAGAGUGAUCUGAUCAUACCCAAGUCUUUAGCUGGGCCACUCUCAGCCAUCACAAUACCAGAGAGGGCUACAGGACCCAAAUGAAAGGGCCAUGGCACAGUGAAGAGCUUGAAAUUCGGUUUGAGGAAUGCCCAGGCACUAAGGUCCACGUGGAGGAAACAUGUGGA
KU758877    GACAGAUGGAAUAGAAGAGAGUGAUCUGAUCAUACCCAAGUCUUUAGCUGGGCCACUCUCAGCCAUCACAAUACCAGAGAGGGCUACAGGACCCAAAUGAAAGGGCCAUGGCACAGUGAAGAGCUUGAAAUUCGGUUUGAGGAAUGCCCAGGCACUAAGGUCCACGUGGAGGAAACAUGUGGA
KU866423    GACAGAUGGAAUAGAAGAGAGUGAUCUGAUCAUACCCAAGUCUUUAGCUGGGCCACUCUCAGCCAUCACAAUACCAGAGAGGGCUACAGGACCCAAAUGAAAGGGCCAUGGCACAGUGAAGAGCUUGAAAUUCGGUUUGAGGAAUGCCCAGGCACCAAGGUCCACGUGGAGGAAACAUGUGGA
KX377336    GACAGAUGGAAUAGAAGAAAGUGAUCUGAUCAUACCUAAGUCUUUAGCUGGGCCACUCUCAGCCACCACAACACCAGAGAGGGCUACAGGACUCAAGUGAAAGGGCCGUGGCAUAGUGAAGAGCUUGAAAUCCGGUUUGAGGAAUGUCCAGGCACCAAGGUCCACGUGGAGGAAACAUGUGGA
KX377337    GACAGAUGGAAUAGAAGAGAGUGAUCUGAUCAUACCCAAGUCUUUAGCUGGGCCACUCUCAGCCAUCACAAUACCAGAGAGGGCUACAGGACCCAAAUGAAAGGGCCAUGGCACAGUGAAGAGCUUGAAAUUCGGUUUGAGGAAUGCCCAGGCACUAAGGUCCACGUGGAGGAAACAUGUGGA
KX369547    GACAGAUGGAAUAGAAGAGAGUGAUCUGAUCAUACCCAAGUCUUUAGCUGGGCCACUCUCAGCCAUCACAAUACCAGAGAGGGCUACAGGACCCAAAUGAAAGGGCCAUGGCACAGUGAAGAGCUUGAAAUUCGGUUUGAGGAAUGCCCAGGCACUAAGGUCCACGUGGAGGAAACAUGUGGA
KX548902    GACAGAUGGAAUAGAAGAGAGUGAUCUGAUCAUACCCAAGUCUUUAGCUGGGCCACUCUCAGCCAUCACAAUACCAGAGAGGGCUACAGGACCCAAAUGAAAGGGCCAUGGCACAGUGAAGAGCUUGAAAUUCGGUUUGAGGAAUGCCCAGGCACUAAGGUCUACGUGGAGGAAACAUGUGGA
KX601166    GACAGAUGGAGUGGAAGAAAGUGAUCUGAUCAUACCCAAGUCCUUAGCUGGUCCACUCUCAGCCACCACAACACCAGAGAGGGUUACAGAACUCAAGUGAAAGGGCCAUGGCAUAGUGAAGAGCUCGAAAUCCGGUUUGAGGAGUGCCCAGGAACCAAGGUUCACGUGGAGGAGACAUGCGGA
KX601167    GACAGAUGGAAUAGAAGAAAGUGAUCUGAUCAUACCUAAGUCUUUAGCUGGGCCACUCUCAGCCACCACAACACCAGAGAGGGCUACAGGACUCAAGUGAAAGGGCCGUGGCAUAGUGAAGAGCUUGAAAUCCGGUUUGAGGAAUGUCCAGGCACCAAGGUCCACGUGGAGGAAACAUGUGGA
KX601168    GACAGAUGGAAUAGAAGAGAGUGAUCUGAUCAUACCCAAGUCUUUAGCUGGGCCACUCUCAGCCAUCACAAUACCAGAGAGGGCUACAGGACCCAAAUGAAAGGGCCAUGGCACAGUGAAGAGCUUGAAAUUCGGUUUGAGGAAUGCCCAGGCACUAAGGUCCACGUGGAGGAAACAUGUGGA
KX766028    GACAGAUGGAAUAGAAGAGAGUGAUCUGAUCAUACCCAAGUCUUUAGCUGGGCCACUCUCAGCCAUCACAAUACCAGAGAGGGCUACAGGACCCAAAUGAAAGGGCCAUGGCACAGUGAAGAGCUUGAAAUUCGGUUUGAGGAAUGCCCAGGCACUAAGGUCCACGUGGAGGAAACAUGUGGA
KX766029    GACAGAUGGAAUAGAAGAGAGUGAUCUGAUCAUACCCAAGUCUUUAGCUGGGCCACUCUCAGCCAUCACAAUACCAGAGAGGGCUACAGGACCCAAAUGAAAGGGCCAUGGCACAGUGAAGAGCUUGAAAUUCGGUUUGAGGAAUGCCCAGGCACUAAGGUCCACGUGGAGGAAACAUGUGGA
KU940224    GACAGAUGGAAUAGAAGAGAGUGAUCUGAUCAUACCCAAGUCUUUAGCUGGGCCACUCUCAGCCAUCACAAUACCAGAGAGGGCUACAGGACCCAAAUGAAAGGGCCAUGGCACAGUGAAGAGCUUGAAAUUCGGUUUGAGGAAUGCCCAGGCACUAAGGUCCACGUGGAGGAAACAUGUGGA
```

```
            3333333333333333333333333333333333333333333333333333333333333333333333333333333333333333333333333333333333333333333333333333333333333333333333333333333333333333333333333333333333333
            2222222222222222222222222222222222222222233333333333333333333333333333333333333333333333333333333333333333333333333333333333333333333333333333334444444444444444444444444444444444444444
            5666666666677777777778888888888999999999900000000001111111111222222222233333333334444444444555555555566666666667777777777888888888899999999990000000000111111111122222222223333333333
Sequence    9012345678901234567890123456789012345678901234567890123456789012345678901234567890123456789012345678901234567890123456789012345678901234567890123456789012345678901234567890123456789
-------------------------------------------------------------------------------------------------------------------------------------------------------------------------------------------------------------
NC_012532   ACUAGAGGACCAUCUCUGAGAUCAACUACUGCAAGUGGAAGGGUCAUUGAGGAAUGGUGCUGUAGGGAAUGCACAAUGCCCCCACUAUCGUUUCGAGCAAAAGACGGCUGCUGGUAUGGAAUGGAGAUAAGGCCCAGGAAAGAACCAGAGAGCAACUUAGUGAGGUCAAUGGUGACAGCGG
KU922960    ACAAGAGGACCAUCUCUGAGAUCAACCACUGCAAGCGGAAGGGUGAUCGAGGAAUGGUGCUGCAGGGAGUGCACAAUGCCCCCACUGUCGUUCUGGGCUAAAGAUGGCUGUUGGUAUGGAAUGGAGAUAAGGCCCAGGAAAGAACCAGAAAGCAACUUAGUAAGGUCAAUGGUGACUGCAG
KU922923    ACAAGAGGACCAUCUCUGAGAUCAACCACUGCAAGCGGAAGGGUGAUCGAGGAAUGGUGCUGCAGGGAGUGCACAAUGCCCCCACUGUCGUUCUGGGCUAAAGAUGGCUGUUGGUAUGGAAUGGAGAUAAGGCCCAGGAAAGAACCAGAAAGCAACUUAGUAAGGUCAAUGGUGACUGCAG
KU820898    ACAAGAGGACCAUCUCUGAGAUCAACCACUGCAAGCGGAAGGGUGAUCGAGGAAUGGUGCUGCAGGGAGUGCACAAUGCCCCCACUGUCGUUCCGGGCUAAAGAUGGCUGUUGGUAUGGAAUGGAGAUAAGGCCCAGGAAAGAACCAGAAAGCAACUUAGUAAGGUCAAUGGUGACUGCAG
KU321639    ACAAGAGGACCAUCUCUGAGAUCAACCACUGCAAGCGGAAGGGUGAUCGAGGAAUGGUGCUGCAGGGAGUGCACAAUGCCCCCACUGUCGUUCCGGGCUAAAGAUGGCUGUUGGUAUGGAAUGGAGAUAAGGCCCAGGAAAGAACCAGAAAGCAACUUAGUAAGGUCAAUGGUGACUGCAG
KU820899    ACAAGAGGACCAUCUCUGAGAUCAACCACUGCAAGCGGAAGGGUGAUCGAGGAAUGGUGCUGCAGGGAGUGCACAAUGCCCCCACUGUCGUUCCAGGCUAAAGAUGGCUGUUGGUAUGGAAUGGAGAUAAGGCCCAGGAAAGAACCAGAAAGUAACUUAGUAAGGUCAAUGGUGACUGCAG
KU853012    ACAAGAGGACCAUCUCUGAGAUCAACCACUGCAAGCGGAAGGGUGAUCGAGGAAUGGUGCUGCAGGGAGUGCACAAUGCCCCCACUGUCGUUCCGGGCUAAAGAUGGCUGUUGGUAUGGAAUGGAGAUAAGGCCCAGGAAAGAACCAGAAAGCAACUUAGUAAGGUCAGUGGUGACUGCAG
KU729217    ACAAGAGGACCAUCUCUGAGAUCAACCACUGCAAGCGGAAGGGUGAUCGAGGAAUGGUGCUGCAGGGAGUGCACAAUGCCCCCACUGUCGUUCCGGGCUAAAGAUGGCUGUUGGUAUGGAAUGGAGAUAAGACCCAGGAAAGAACCAGAAAGCAACUUAGUAAGGUCAGUGGUGACUGCAG
KU729218    ACAAGAGGACCAUCUCUGAGAUCAACCACUGCAAGCGGAAGGGUGAUCGAGGAAUGGUGCUGCAGGGAGUGCACAAUGCCCCCACUGUCGUUCCGGGCUAAAGAUGGCUGUUGGUAUGGAAUGGAGAUAAGGCCCAGGAAAGAACCAGAAAGCAACUUAGUAAGGUCAAUGGUGACUGCAG
KU761564    ACAAGAGGACCAUCUCUGAGAUCAACCACUGCAAGCGGAAGGGUGAUCGAGGAAUGGUGCUGCAGGGAGUGCACAAUGCCCCCACUGUCGUUCCGGGCUAAAGAUGGCUGUUGGUAUGGAAUGGAGAUAAGGCCCAGGAAAGAACCAGAAAGCAACUUAGUAAGGUCAAUGGUGACUGCAG
KU740184    ACAAGAGGACCAUCUCUGAGAUCAACCACUGCAAGCGGAAGGGUGAUCGAGGAAUGGUGCUGCAGGGAGUGCACAAUGCCCCCACUGUCGUUCCGGGCUAAAGAUGGCUGUUGGUAUGGAAUGGAGAUAAGGCCCAGGAAAGAACCAGAAAGCAACUUAGUAAGGUCAAUGGUGACUGCAG
KU744693    ACAAGAGGACCAUCUCUGAGAUCAACCACUGCAAGCGGAAGGGUGAUCGAGGAAUGGUGCUCCAGGGAGUGCACAAUGCCCCCACUGUCCUUCCAGGCUAAAGAUGGCUGUUGGUAUGGAAUGGAGAUAAGGCCCAGGAAAGAACCAGAAAGCAACUUAGUAAGGUCAAUGGUGACUGCAG
KU497555    ACAAGAGGACCAUCUCUGAGAUCAACCACUGCAAGCGGAAGGGUGAUCGAGGAAUGGUGCUGCAGGGAGUGCACAAUGCCCCCACUGUCGUUCCGGGCUAAAGAUGGCUGUUGGUAUGGAAUGGAGAUAAGGCCCAGGAAAGAACCAGAAAGCAACUUAGUAAGGUCAAUGGUGACUGCAG
KU527068    ACAAGAGGACCAUCUCUGAGAUCAACCACUGCAAGCGGAAGGGUGAUCGAGGAAUGGUGCUGCAGGGAGUGCACAAUGCCCCCACUGUCGUUCCGGGCUAAAGAUGGCUGUUGGUAUGGAAUGGAGAUAAGGCCCAGGAAAGAACCAGAAAGCAACUUAGUAAGGUCAGUGGUGACUGCAG
KU501217    ACAAGAGGACCAUCUCUGAGAUCAACCACUGCAAGCGGAAGGGUGAUCGAGGAAUGGUGCUGCAGGGAGUGCACAAUGCCCCCACUGUCGUUCCGGGCUAAAGAUGGCUGUUGGUAUGGAAUGGAGAUAAGGCCCAGGAAAGAACCAGAAAGCAACUUAGUAAGGUCAAUGGUGACUGCAG
KU501216    ACAAGAGGACCAUCUCUGAGAUCAACCACUGCAAGCGGAAGGGUGAUCGAGGAAUGGUGCUGCAGGGAGUGCACAAUGCCCCCACUGUCGUUCCGGGCUAAAGAUGGCUGUUGGUAUGGAAUGGAGAUAAGGCCCAGGAAAGAACCAGAAAGCAACUUAGUAAGGUCAAUGGUGACUGCAG
KU501215    ACAAGAGGACCAUCUCUGAGAUCAACCACUGCAAGCGGAAGGGUGAUCGAGGAAUGGUGCUGCAGGGAGUGCACAAUGCCCCCACUGUCGUUCCGGGCUAAAGAUGGCUGUUGGUAUGGAAUGGAGAUAAGGCCCAGGAAAGAACCAGAAAGCAACUUAGUAAGGUCAAUGGUGACUGCAG
KU365780    ACAAGAGGACCAUCUCUGAGAUCAACCACUGCAAGCGGAAGGGUGAUCGAGGAAUGGUGCUGCAGGGAGUGCACAAUGCCCCCACUGUCGUUCCGGGCUAAAGAUGGCUGUUGGUAUGGAAUGGAGAUAAGGCCCAGGAAAGAACCAGAAAGCAACUUAGUAAGGUCAAUGGUGACUGCAG
KU365779    ACAAGAGGACCAUCUCUGAGAUCAACCACUGCAAGCGGAAGGGUGAUCGAGGAAUGGUGCUGCAGGGAGUGCACAAUGCCCCCACUGUCGUUCCGGGCUAAAGAUGGCUGUUGGUAUGGAAUGGAGAUAAGGCCCAGGAAAGAACCAGAAAGCAACUUAGUAAGGUCAAUGGUGACUGCAG
KU707826    ACAAGAGGACCAUCUCUGAGAUCAACCACUGCAAGCGGAAGGGUGAUCGAGGAAUGGUGCUGCAGGGAGUGCACAAUGCCCCCACUGUCGUUCCGGGCUAAAGAUGGCUGUUGGUAUGGAAUGGAGAUAAGGCCCAGGAAAGAACCAGAAAGCAACUUAGUAAGGUCAAUGGUGACUGCAG
KU365778    ACAAGAGGACCAUCUCUGAGAUCAACCACUGCAAGCGGAAGGGUGAUCGAGGAAUGGUGCUGCAGGGAGUGCACAAUGCCCCCACUGUCGUUCCGGGCUAAAGAUGGCUGUUGGUAUGGAAUGGAGAUAAGGCCCAGGAAAGAACCAGAAAGCAACUUAGUAAGGUCAAUGGUGACUGCAG
KU365777    ACAAGAGGACCAUCUCUGAGAUCAACCACUGCAAGCGGAAGGGUGAUCGAGGAAUGGUGCUGCAGGGAGUGCACAAUGCCCCCACUGUCGUUCCGGGCUAAAGAUGGCUGUUGGUAUGGAAUGGAGAUAAGGCCCAGGAAAGAACCAGAAAGCAACUUAGUAAGGUCAAUGGUGACUGCAG
KU312312    ACGAGAGGACCAUCUCUGAGAUCAACCACUGCAAGCGGAAGGGUGAUCGAGGAAUGGUGCUGCAGGGAGUGCACAAUGCCCCCACUGUCGUUCCGGGCUAAAGAUGGCUGUUGGUAUGGAAUGGAGAUAAGGCCCAGGAAAGAACCAGAAAGCAACUUAGUAAGGUCAAUGGUGACUGCAG
KF268950    ACUAGAGGACCAUCUCUGAGAUCAACCACUGCAAGUGGAAGGGUCAUUGAGGAAUGGUGCUGUAGAGAAUGCACAAUGCCCCCACUAUCGUUUCGAGCAAAAGACGGCUGCUGGUAUGGAAUGGAGAUAAGGCCCAGGAAAGAACCAGAGAGCAACUUAGUGAGGUCAAUGGUGACAGCGG
KF268949    ACUAGAGGACCAUCUCUGAGAUCAACCACUGCAAGUGGAAGGGUCAUUGAGGAAUGGUGCUGUAGAGAAUGCACAAUGCCCCCACUAUCGUUUCGAGCAAAAGAUGGCUGCUGGUAUGGAAUGGAGAUAAGGCCCAGGAAAGAACCAGAGAGCAACUUAGUGAGGUCAAUGGUGACAGCGG
KF268948    ACUAGAGGACCAUCUCUGAGAUCAACCACUGCAAGUGGAAGGGUCAUUGAGGAAUGGUGCUGUAGAGAAUGCACAAUGCCCCCACUAUCGUUUCGAGCAAAAGACGGCUGCUGGUAUGGAAUGGAGAUAAGGCCCAGGAAAGAACCAGAGAGCAACUUAGUGAGGUCAAUGGUGACAGCGG
KJ776791    ACAAGAGGACCAUCUCUGAGAUCAACCACUGCAAGCGGAAGGGUGAUCGAGGAAUGGUGCUGCAGGGAGUGCACAAUGCCCCCACUGUCGUUCCGGGCUAAAGAUGGCUGUUGGUAUGGAAUGGAGAUAAGGCCCAGGAAAGAACCAGAAAGUAACUUAGUAAGGUCAAUGGUGACUGCAG
KF383119    ACUAGAGGACCAUCUCUGAGAUCAACUACUGCAAGUGGAAGGGUCAUUGAGGAAUGGUGCUGUAGGGAAUGCACAAUGCCCCCACUAUCGUUUCGAGCAAAAGACGGCUGCUGGUAUGGAAUGGAGAUAAGGCCCAGGAAAGAACCAGAGAGCAACUUAGUGAGGUCAAUGGUGACAGCGG
KF383118    ACUAGAGGACCAUCUCUGAGAUCAACUACUGCAAGUGGAAGGGUCAUUGAGGAAUGGUGCUGUAGGGAAUGCACAAUGCCCCCACUAUCGUUUCGAGCAAAAGACGGCUGCUGGUAUGGAAUGGAGAUAAGGCCCAGGAAAGAACCAGAGAGCAACUUAGUGAGGUCAAUGGUGACAGCGG
KF383117    ACUAGAGGACCAUCUCUGAGAUCAACUACUGCAAGUGGAAGGGUCAUUGAGGAAUGGUGCUGUAGGGAAUGCACAAUGCCCCCACUAUCGUUUCGAGCAAAAGACGGCUGCUGGUAUGGAAUGGAGAUAAGGCCCAGGAAAGAACCAGAGAGCAACUUAGUGAGGUCAAUGGUGACAGCGG
```

```
KF383116      ACUAGAGGACCAUCUCUGAGAUCAACUACUGCAAGUGGAAGGGUCAUAGAGGAAUGGUGCUGUAGGGAAUGCACAAUGCCUCCACUAUCGUUCCGGGCGAAAGACGGCUGCUGGUAUGGAAUGGAGAUAAGGCCCAGAAAGGAACCAGAGAGCAACUUAGUAAGGUCUAUGGUGACAGCAG
KF383115      ACUAGAGGACCAUCUCUGAGAUCAACCACUGCAAGUGGAAGGGUCAUUGAGGAAUGGUGCUGUAGAGAAUGCACAAUGCCCCCACUAUCAUUUCGAGCGAAAGACGGCUGCUGGUAUGGAAUGGAGAUAAGGCCCAGGAAAGAACCAGAGAGCAAUUUAGUGAGGUCAAUGGUGACAGCGG
EU545988      ACAAGAGGACCAUCUCUGAGAUCAACCACUGCAAGCGGAAGGGUGAUCGAGGAAUGGUGCUGCAGGGAAUGCACAAUGCCCCCACUGUCGUUCCGGGCUAAAGAUGGCUGUUGGUAUGGAAUGGAGAUAAGGCCCAGGAAAGAACCAGAAAGUAACUUAGUAAGGUCAAUGGUGACUGCAG
KU955589      ACAAGAGGACCAUCUCUGAGAUCAACCACUGCAAGCGGAAGGGUGAUCGAGGAAUGGUGCUGCAGGGAGUGCACAAUGCCCCCACUGUCGUUCCAGGCUAAAGAUGGCUGUUGGUAUGGAAUGGAGAUAAGGCCCAGGAAAGAACCAGAAAGUAACUUAGUAAGGUCAAUGGUGACUGCAG
KU955590      ACAAGAGGACCAUCUCUGAGAUCAACCACUGCAAGCGGAAGGGUGAUCGAGGAAUGGUGCUGCAGGGAGUGCACAAUGCCCCCACUGUCGUUCCGGGCUAAAGAUGGCUGUUGGUAUGGAAUGGAGAUAAGGCCCAGGAAAGAACCAGAAAGCAACUUAGUAAGGUCAAUGGUGACUGCAG
KU940228      ACAAGAGGACCAUCUCUGAGAUCAACCACUGCAAGCGGAAGGGUGAUCGAGGAAUGGUGCUGCAGGGAGUGCACAAUGCCCCCACUGUCGUUCCGGGCUAAAGAUGGCUGUUGGUAUGGAAUGGAGAUAAGGCCCAGGAAAGAACCAGAAAGCAACUUAGUAAGGUCAAUGGUGACUGCAG
KU991811      ACAAGAGGACCAUCUCUGAGAUCAACCACUGCAAGCGGAAGGGUGAUCGAGGAAUGGUGCUGCAGGGAGUGCACAAUGCCCCCACUGUCGUUCCGGGCUAAAGAUGGCUGUUGGUAUGGAAUGGAGAUAAGGCCCAGGAAAGAACCAGAAAGCAACUUAGUAAGGUCAAUGGUGACUGCAG
KU509998      ACAAGAGGACCAUCUCUGAGAUCAACCACUGCAAGCGGAAGGGUGAUCGAGGAAUGGUGCUGCAGGGAGUGCACAAUGCCCCCACUGUCGUUCCGGGCUAAAGAUGGCUGUUGGUAUGGAAUGGAGAUAAGGCCCAGGAAAGAACCAGAAAGCAACUUAGUAAGGUCAAUGGUGACUGCAG
KX056898      ACAAGAGGACCAUCUCUGAGAUCAACCACUGCAAGCGGAAGGGUGAUCGAGGAAUGGUGCUGCAGGGAGUGCACAAUGCCCCCACUGUCGUUCCGGGCUAAAGAUGGCUGUUGGUAUGGAAUGGAGAUAAGGCCCAGGAAAGAACCAGAAAGCAACUUAGUAAGGUCAAUGGUGACUGCAG
KX117076      ACAAGAGGACCAUCUCUGAGAUCAACCACUGCAAGCGGAAGGGUGAUCGAGGAAUGGUGCUGCAGGGAGUGCACAAUGCCCCCACUGUCGUUCCAGGCUAAAGAUGGCUGUUGGUAUGGAAUGGAGAUAAGGCCCAGGAAAGAACCAGAAAGUAACUUAGUAAGGUCAAUGGUGACUGCAG
KX185891      ACAAGAGGACCAUCUCUGAGAUCAACCACUGCAAGCGGAAGGGUGAUCGAGGAAUGGUGCUGCAGGGAGUGCACAAUGCCCCCACUGUCGUUCCAGGCUAAAGAUGGCUGUUGGUAUGGAAUGGAGAUAAGGCCCAGGAAAGAACCAGAAAGUAACUUAGUAAGGUCAAUGGUGACUGCAG
KX247646      ACAAGAGGACCAUCUCUGAGAUCAACCACUGCAAGCGGAAGGGUGAUCGAGGAAUGGUGCUGCAGGGAGUGCACAAUGCCCCCACUGUCGUUCUGGGCUAAAGAUGGCUGUUGGUAUGGAAUGGAGAUAAGGCCCAGGAAAGAACCAGAAAGCAACUUAGUAAGGUCAAUGGUGACUGCAG
KX262887      ACAAGAGGACCAUCUCUGAGAUCAACCACUGCAAGCGGAAGGGUGAUCGAGGAAUGGUGCUGCAGGGAGUGCACAAUGCCCCCACUGUCGUUCCGGGCUAAAGAUGGCUGUUGGUAUGGAAUGGAGAUAAGGCCCAGGAAAGAACCAGAAAGCAACUUAGUAAGGUCAAUGGUGACUGCAG
KX280026      ACAAGAGGACCAUCUCUGAGAUCAACCACUGCAAGCGGAAGGGUGAUCGAGGAAUGGUGCUGCAGGGAGUGCACAAUGCCCCCACUGUCGUUCCGGGCUAAAGAUGGCUGUUGGUAUGGAAUGGAGAUAAGGCCCAGGAAAGAACCAGAAAGCAACUUAGUAAGGUCAGUGGUGACUGCAG
KU758877      ACAAGAGGACCAUCUCUGAGAUCAACCACUGCAAGCGGAAGGGUGAUCGAGGAAUGGUGCUGCAGGGAGUGCACAAUGCCCCCACUGUCGUUCCGGGCUAAAGAUGGCUGUUGGUAUGGAAUGGAGAUAAGGCCCAGGAAAGAACCAGAAAGCAACUUAGUAAGGUCAAUGGUGACUGCAG
KU866423      ACAAGAGGACCAUCUCUGAGAUCAACCACAGCAAGCGGAAGGGUGAUCGAGGAAUGGUGCUGCAGGGAGUGCACAAUGCCCCCACUGUCGUUCCAGGCUAAAGAUGGCUGUUGGUAUGGAAUGGAGAUAAGGCCCAGGAAAGAACCAGAAAGUAACUUAGUAAGGUCAAUGGUGACUGCAG
KX377336      ACGAGAGGACCGUCCCUGAGAUCAACCACUGCAAGCGGAAGGGUGAUCGAGGAAUGGUGCUGCAGGGAAUGCACAAUGCCCCCAUUGUCGUUCCGGGCAAAAGAUGGCUGUUGGUAUGGAAUGGAGAUAAGGCCCAGGAAGGAACCAGAGAGUAACCUAGUAAGGUCAAUGGUGACUGCAG
KX377337      ACAAGAGGACCAUCUCUGAGAUCAACCACUGCAAGCGGAAGGGUGAUCGAGGAAUGGUGCUGCAGGGAGUGCACAAUGCCCCCACUGUCGUUCCGGGCUAAAGAUGGCUGUUGGUAUGGAAUGGAGAUAAGGCCCAGGAAAGAACCAGAAAGCAACUUAGUAAGGUCAAUGGUGACUGCAG
KX369547      ACAAGAGGACCAUCUCUGAGAUCAACCACUGCAAGCGGAAGGGUGAUCGAGGAAUGGUGCUGCAGGGAGUGCACAAUGCCCCCACUGUCGUUCCGGGCUAAAGAUGGCUGUUGGUAUGGAAUGGAGAUAAGGCCCAGGAAAGAACCAGAAAGUAACUUAGUAAGGUCAAUGGUGACUGCAG
KX548902      ACAAGAGGACCAUCUCUGAGAUCACUCACUGCAAGCGGAAGGGUGAUCGAGGAAUGGUGCUGCAGGGAGUGCACAAUGCCCCCACUGUCGUUCUGGGCUAAAGAUGGCUGUUGGUAUGGAAUGGAGAUAAGGCCCAGGAAAGAACCAGAAAGCAACUUAGUAAGGUCAAUGGUGACUGCAG
KX601166      ACUAGAGGACCAUCUCUGAGAUCAACUACUGCUAGUGGAAGGGUCAUAGAGGAAUGGUGCUGUAGGGAAUGCACAAUGCCUCCACUAUCGUUCCGGGCGAAAGACGGCUGCUGGUAUGGAAUGGAGAUAAGGCCCAGAAAGGAACCAGAGAGCAACUUAGUGAGGUCCAUGGUGACAGCAG
KX601167      ACGAGAGGACCGUCCCUGAGAUCAACCACUGCAAGCGGAAGGGUGAUCGAGGAAUGGUGCUGCAGGGAAUGCACAAUGCCCCCAUUGUCGUUCCGGGCAAAAGAUGGCUGUUGGUAUGGAAUGGAGAUAAGGCCCAGGAAGGAACCAGAGAGUAACCUAGUAAGGUCAAUGGUGACUGCAG
KX601168      ACAAGAGGACCAUCUCUGAGAUCAACCACUGCAAGCGGAAGGGUGAUCGAGGAAUGGUGCUGCAGGGAGUGCACAAUGCCCCCACUGUCGUUCCGGGCUAAAGAUGGCUGUUGGUAUGGAAUGGAGAUAAGGCCCAGGAAAGAACCAGAAAGCAACUUAGUAAGGUCAAUGGUGACUGCAG
KX766028      ACAAGAGGACCAUCUCUGAGAUCAACCACUGCAAGCGGAAGGGUGAUCGAGGAAUGGUGCUGCAGGGAGUGCACAAUGCCCCCACUGUCGUUCCGGGCUAAAGAUGGCUGUUGGUAUGGAAUGGAGAUAAGGCCCAGGAAAGAACCAGAAAGCAACUUAGUAAGGUCAAUGGUGACUGCAG
KX766029      ACAAGAGGACCAUCUCUGAGAUCAACCACUGCAAGCGGAAGGGUGAUCGAGGAAUGGUGCUGCAGGGAGUGCACAAUGCCCCCACUGUCGUUCCGGGCUGAAGAUGGCUGUUGGUAUGGAAUGGAGAUAAGGCCCAGGAAAGAACCAGAAAGCAACUUAGUAAGGUCAAUGGUGACUGCAG
KU940224      ACAAGAGGACCAUCUCUGAGAUCAACCACUGCAAGCGGAAGGGUGAUCGAGGAAUGGUGCUGCAGGGAGUGCACAAUGCCCCCACUGUCGUUCCGGGCUAAAGAUGGCUGUUGGUAUGGAAUGGAGAUAAGGCCCAGGAAAGAACCAGAAAGCAACUUAGUAAGGUCAAUGGUGACUGCAG


              3333333333333333333333333333333333333333333333333333333333333333333333333333333333333333333333333333333333333333333333333333333333333333333333333333333333333333333333333333333333333
              4444444444444444444444444444444444444444444444444444444444445555555555555555555555555555555555555555555555555555555555555555555555555555555555555555555555555555666666666666666666666
              4444444444555555555566666666667777777777888888888899999999990000000000111111111122222222223333333333444444444455555555556666666666777777777788888888889999999999000000000011111111112
Sequence      0123456789012345678901234567890123456789012345678901234567890123456789012345678901234567890123456789012345678901234567890123456789012345678901234567890123456789012345678901234567890
-------------------------------------------------------------------------------------------------------------------------------------------------------------------------------------------------
NC_012532     GGUCAACCGAUCAUAUGGACCACUUCUCUCUUGGAGUGCUUGUGAUUCUACUCAUGGUGCAGGAGGGGUUGAAGAAGAGAAUGACCACAAAGAUCAUCAUGAGCACAUCAAUGGCAGUGCUGGUAGUCAUGAUCUUGGGAGGAUUUUCAAUGAGUGACCUGGCCAAGCUUGUGAUCCUGAU
KU922960      GAUCAACUGAUCACAUGGAUCACUUCUCCCUUGGAGUGCUUGUGAUUCUGCUCAUGGUGCAGGAAGGGCUGAAGAAGAGAAUGACCACAAAGAUCAUCAUAAGCACAUCAAUGGCAGUGCUGGUAGCUAUGAUCCUGGGAGGAUUUUCAAUGAGUGACCUGGCUAAGCUUGCAAUUUUGAU
KU922923      GAUCAACUGAUCACAUGGAUCACUUCUCCCUUGGAGUGCUUGUGAUUCUGCUCAUGGUGCAGGAAGGGCUGAAGAAGAGAAUGACCACAAAGAUCAUCAUAAGCACAUCAAUGGCAGUGCUGGUAGCUAUGAUCCUGGGAGGAUUUUCAAUGAGUGACCUGGCUAAGCUUGCAAUUUUGAU
KU820898      GAUCAACUGAUCACAUGGACCACUUCUCCCUUGGAGUGCUUGUGAUCCUGCUCAUGGUGCAGGAAGGGCUGAAGAAGAGAAUGACCACAAAGAUCAUCAUAAGCACAUCAAUGGCAGUGCUGGUAGCUAUGAUCCUGGGAGGAUUUUCAAUGAGUGACCUGGCUAAGCUUGCAAUUUUGAU
KU321639      GAUCAACUGAUCACAUGGAUCACUUCUCCCUUGGAGUGCUUGUGAUUCUGCUCAUGGUGCAGGAAGGGCUGAAGAAGAGAAUGACCACAAAGAUCAUCAUAAGCACAUCAAUGGCAGUGCUGGUAGCUAUGAUCCUGGGAGGAUUUUCAAUGAGUGACCUGGCUAAGCUUGCAAUUUUGAU
KU820899      GAUCAACUGAUCACAUGGAUCACUUCUCCCUUGGAGUGCUUGUGAUUCUGCUCAUGGUGCAGGAAGGGCUGAAGAAGAGAAUGACCACAAAGAUCAUCAUAAGCACAUCAAUGGCAGUGCUGGUAGCUAUGAUCCUGGGAGGAUUUUCAAUGAGUGACCUGGCUAAGCUUGCAAUUUUGAU
KU853012      GAUCAACUGAUCACAUGGAUCACUUCUCCCUUGGAGUGCUUGUGAUUCUGCUCAUGGUGCAGGAAGGGCUGAAGAAGAGAAUGACCACAAAGAUCAUCAUAAGCACAUCRAUGGCAGUGCUGGUAGCUAUGAUCCUGGGAGGAUUUUCAAUGAGUGACCUGGCUAAGCUUGCAAUUUUGAU
KU729217      GAUCAACUGAUCACAUGGAUCACUUCUCCCUUGGAGUGCUUGUGAUUCUGCUCAUGGUGCAGGAAGGGCUGAAGAAGAGAAUGACCACAAAGAUCAUCAUAAGCACAUCAAUGGCAGUGCUGGUAGCUAUGAUCCUGGGAGGAUUUUCAAUGAGUGACCUGGCUAAGCUUGCAAUUUUGAU
KU729218      GAUCAACUGAUCACAUGGAUCACUUCUCCCUUGGAGUGCUUGUGAUUCUGCUCAUGGUGCAGGAAGGGCUGAAGAAGAGAAUGACCACAAAGAUCAUCAUAAGCACAUCAAUGGCAGUGCUGGUAGCUAUGAUCCUGGGAGGAUUUUCAAUGAGUGACCUGGCUAAGCUUGCAAUUUUGAU
KU761564      GAUCAACUGAUCACAUGGACCACUUCUCCCUUGGAGUGCUUGUGAUCCUGCUCAUGGUGCAGGAAGGGCUGAAGAAGAGAAUGACCACAAAGAUCAUCAUAAGCACAUCAAUGGCAGUGCUGGUAGCUAUGAUCCUGGGAGGAUUUUCAAUGAGUGACCUGGCUAAGCUUGCAAUUUUGAU
KU740184      GAUCAACUGAUCACAUGGACCACUUCUCCCUUGGAGUGCUUGUGAUCCUGCUCAUGGUGCAGGAAGGGCUGAAGAAGAGAAUGACCACAAAGAUCAUCAUAAGCACAUCAAUGGCAGUGCUGGUAGCUAUGAUCCUGGGAGGAUUUUCAAUGAGUGACCUGGCUAAGCUUGCAAUUUUGAU
KU744693      GAUCAACUGAUCACAUGGAUCACUUCUCCCUUGGAGUGCUUGUGAUUCUGCUCAUGGUGCAGGAAGGGCUGAAGAAGAGAAUGACCACAAAGAUCAUCAUAAGCACAUCAAUGGCAGUGCUGGUAGCUAUGAUCCUGGGAGGAUUUUCAAUGAGUGACCUGGCUAAGCUUGCAAUUUUGAU
KU497555      GAUCAACUGAUCACAUGGAUCACUUCUCCCUUGGAGUGCUUGUGAUUCUGCUCAUGGUGCAGGAAGGGCUGAAGAAGAGAAUGACCACAAAGAUCAUCAUAAGCACAUCAAUGGCAGUGCUGGUAGCUAUGAUCCUGGGAGGAUUUUCAAUGAGUGACCUGGCUAAGCUUGCAAUUUUGAU
KU527068      GAUCAACUGAUCACAUGGAUCACUUCUCCCUUGGAGUGCUUGUGAUUCUGCUCAUGGUGCAGGAAGGGCUGAAGAAGAGAAUGACCACAAAGAUCAUCAUAAGCACAUCAAUGGCAGUGCUGGUAGCUAUGAUCCUGGGAGGAUUUUCAAUGAGUGACCUGGCUAAGCUUGCAAUUUUGAU
KU501217      GAUCAACUGAUCACAUGGAUCACUUCUCCCUUGGAGUGCUUGUGAUUCUGCUCAUGGUGCAGGAAGGGCUAAAGAAGAGAAUGACCACAAAGAUCAUCAUAAGCACAUCAAUGGCAGUGCUGGUAGCUAUGAUCCUGGGAGGAUUUUCAAUGAGUGACCUGGCUAAGCUUGCAAUUUUGAU
KU501216      GAUCAACUGAUCACAUGGAUCACUUCUCCCUUGGAGUGCUUGUGAUUCUGCUCAUGGUGCAGGAAGGGCUAAAGAAGAGAAUGACCACAAAGAUCAUCAUAAGCACAUCAAUGGCAGUGCUGGUAGCUAUGAUCCUGGGAGGAUUUUCAAUGAGUGACCUGGCUAAGCUUGCAAUUUUGAU
KU501215      GAUCAACUGAUCACAUGGACCACUUCUCCCUUGGAGUGCUUGUGAUCCUGCUCAUGGUGCAGGAAGGGCUGAAGAAGAGAAUGACCACAAAGAUCAUCAUAAGCACAUCAAUGGCAGUGCUGGUAGCUAUGAUCCUGGGAGGAUUUUCAAUGAGUGACCUGGCUAAGCUUGCAAUUUUGAU
KU365780      GAUCAACUGAUCACAUGGACCACUUCUCCCUUGGAGUGCUUGUGAUUCUGCUCAUGGUGCAGGAAGGGCUGAAGAAGAGAAUGACCACAAAGAUCAUCAUAAGCACAUCAAUGGCAGUGCUGGUAGCUAUGAUCCUGGGAGGAUUUUCAAUGAGUGACCUGGCUAAGCUUGCAAUUUUGAU
KU365779      GAUCAACUGAUCACAUGGACCACUUCUCCCUUGGAGUGCUUGUGAUUCUGCUCAUGGUGCAGGAAGGGCUGAAGAAGAGAAUGACCACAAAGAUCAUCAUAAGCACAUCAAUGGCAGUGCUGGUAGCUAUGAUCCUGGGAGGAUUUUCAAUGAGUGACCUGGCUAAGCUUGCAAUUUUGAU
KU707826      GAUCAACUGAUCACAUGGACCACUUCUCCCUUGGAGUGCUUGUGAUUCUGCUCAUGGUGCAGGAAGGGCUGAAGAAGAGAAUGACCACAAAGAUCAUCAUAAGCACAUCAAUGGCAGUGCUGGUAGCUAUGAUCCUGGGAGGAUUUUCAAUGAGUGACCUGGCUAAGCUUGCAAUUUUGAU
KU365778      GAUCAACUGAUCACAUGGACCACUUCUCCCUUGGAGUGCUUGUGAUCCUGCUCAUGGUGCAGGAAGGGCUGAAGAAGAGAAUGACCACAAAGAUCAUCAUAAGCACAUCAAUGGCAGUGCUGGUAGCUAUGAUCCUGGGAGGAUUUUCAAUGAGUGACCUGGCUAAGCUUGCAAUUUUGAU
KU365777      GAUCAACUGAUCACAUGGACCACUUCUCCCUUGGAGUGCUUGUGAUUCUGCUCAUGGUGCAGGAAGGGCUGAAGAAGAGAAUGACCACAAAGAUCAUCAUAAGCACAUCAAUGGCAGUGCUGGUAGCUAUGAUCCUGGGAGGAUUUUCAAUGAGUGACCUGGCUAAGCUUGCAAUUUUGAU
KU312312      GAUCAACUGAUCACAUGGACCACUUCUCCCUUGGAGUGCUUGUGAUUCUGCUCAUGGUGCAGGAAGGGUUGAAGAAGAGAAUGACCACAAAGAUCAUCAUAAGCACAUCAAUGGCAGUGCUGGUAGCUAUGAUCCUGGGAGGAUUUUCAAUGAGUGACCUGGCUAAGCUUGCAAUUUUGAU
KF268950      GAUCAACCGAUCACAUGGACCACUUCUCUCUUGGAGUGCUUGUGAUUCUACUCAUGGUGC??GAAGGGCUGAAGAAGAGAAUGACCACAAAGAUCAUUAUGAGCACAUCAAUGGCAGUGCUGGUGGCCAUGAUCCUGGGAGGAUUUUUAAUGAGUGACUUGGCUAAGCUUGUGAUCCUGAU
KF268949      GAUCAACCGAUCACAUGGACCACUUCUCUCUUGGAGUGCUUGUGAUUCUACUCAUGGUGCAGGAAGGGUUGAAGAAGAGAAUGACCACGAAGAUCAUUAUGAGCACAUCAAUGGCAGUGCUGGUAGCCAUGAUCCUGGGAGGAUUUUCAAUGAGUGACCUGGCUAAGCUUGUGAUCCUGAU
KF268948      GAUCAACCGAUCACAUGGACCACUUCUCUCUUGGAGUGCUUGUGAUUCUACUCAUGGUGCAGGAAGGGCUGAAGAAGAGAAUGACCACAAAGAUCAUUAUGAGCACAUCAAUGGCAGUGCUGGUGGCCAUGAUCCUGGGAGGAUUUUUAAUGAGUGACUUGGCUAAGCUUGUGAUCCUGAU
KJ776791      GAUCAACUGAUCACAUGGAUCACUUCUCCCUUGGAGUGCUUGUGAUUCUGCUCAUGGUGCAGGAAGGGCUGAAGAAGAGAAUGACCACAAAGAUCAUCAUAAGCACAUCGAUGGCAGUGCUGGUAGCUAUGAUCCUGGGAGGAUUUUCAAUGAGUGACCUGGCUAAGCUUGCAAUUUUGAU
KF383119      GGUCAACCGAUCAUAUGGACCACUUCUCUCUUGGAGUGCUUGUGAUUCUACUCAUGGUGCAGGAGGGGUUGAAGAAGAGAAUGACCACAAAGAUCAUCAUGAGCACAUCAAUGGCAGUGCUGGUAGUCAUGAUCUUGGGAGGAUUUUCAAUGAGUGACCUGGCCAAGCUUGUGAUCCUGAU
KF383118      GGUCAACCGAUCAUAUGGACCACUUCUCUCUUGGAGUGCUUGUGAUUCUACUCAUGGUGCAGGAGGGGUUGAAGAAGAGAAUGACCACAAAGAUCAUCAUGAGCACAUCAAUGGCAGUGCUGGUAGUCAUGAUCUUGGGAGGAUUUUCAAUGAGUGACCUGGCCAAGCUUGUGAUCCUGAU
KF383117      GGUCAACCGAUCAUAUGGACCACUUCUCUCUUGGAGUGCUUGUGAUUCUACUCAUGGUGCAGGAGGGGUUGAAGAAGAGAAUGACCACAAAGAUCAUCAUGAGCACAUCAAUGGCAGUGCUGGUAGCCAUGGUCUUGGGAGGAUUCUCAAUGAGUGAUCUGGCUAAGCUUGUGAUCCUGAU
KF383116      GAUCAACCGAUCAUAUGGAUCACUUCUCUCUUGGAGUGCUUGUGAUUCUACUCAUGGUGCAGGAAGGUUUGAAGAAGAGAAUGACCACAAAGAUCAUAAUGAGCACAUCAAUGGCAGUGCUGGUAGCCAUGGUCUUGGGAGGAUUCUCAAUGAGUGACCUGGCUAAGCUUGUGAUCCUGAU
KF383115      GAUCAACCGAUCACAUGGACCACUUCUCUCUUGGAGUGCUUGUGAUUCUACUCAUGGUGCAGGAAGGGCUGAAGAAGAGAAUGACCACGAAGAUCAUUGUGAGCACAUCAAUGGCAGUGCUGGUAGCCAUGAUCUUGGGAGGAUUCUCAAUGAGUGACCUGGCUAAGCUUGUGAUCCUGAU
EU545988      GAUCAACUGAUCACAUGGAUCACUUCUCCCUUGGAGUGCUUGUGAUUCUGCUCAUGGUGCAGGAAGGGCUGAAGAAGAGAAUGACCACAAAGAUCAUCAUAAGCACAUCAAUGGCAGUGCUGGUAGCUAUGAUCCUGGGAGGAUUUUCAAUGAGUGACCUGGCCAAGCUUGCAAUUUUGAU
KU955589      GAUCAACUGAUCACAUGGAUCACUUCUCCCUUGGAGUGCUUGUGAUUCUGCUCAUGGUGCAGGAAGGGCUGAAGAAGAGAAUGACCACAAAGAUCAUCAUAAGCACAUCAAUGGCAGUGCUGGUAGCUAUGAUCCUGGGAGGAUUUUCAAUGAGUGACCUGGCUAAGCUUGCAAUUUUGAU
KU955590      GAUCAACUGAUCACAUGGACCACUUCUCCCUUGGAGUGCUUGUGAUUCUGCUCAUGGUGCAGGAAGGGCUGAAGAAGAGAAUGACCACAAAGAUCAUCAUAAGCACAUCAAUGGCAGUGCUGGUAGCUAUGAUCCUGGGAGGAUUUUCAAUGAGUGACCUGGCUAAGCUUGCAAUUUUGAU
KU940228      GAUCAACUGAUCACAUGGAUCACUUCUCCCUUGGAGUGCUUGUGAUUCUGCUCAUGGUGCAGGAAGGGCUGAAGAAGAGAAUGACCACAAAGAUCAUCAUAAGCACAUCAAUGGCAGUGCUGGUAGCUAUGAUCCUGGGAGGAUUUUCAAUGAGUGACCUGGCUAAGCUUGCAAUUUUGAU
KU991811      GAUCAACUGAUCACAUGGAUCACUUCUCCCUUGGAGUGCUUGUGAUUCUGCUCAUGGUGCAGGAAGGGCUGAAGAAGAGAAUGACCACAAAGAUCAUCAUAAGCACAUCAAUGGCAGUGCUGGUAGCUAUGAUCCUGGGAGGAUUUUCAAUGAGUGACCUGGCUAAGCUUGCAAUUUUGAU
KU509998      GAUCAACUGAUCACAUGGAUCACUUCUCCCUUGGAGUGCUUGUGAUUCUGCUCAUGGUGCAGGAAGGGCUGAAGAAGAGAAUGACCACAAAGAUCAUCAUAAGCACAUCAAUGGCAGUGCUGGUAGCUAUGAUCCUGGGAGGAUUUUCAAUGAGUGACCUGGCUAAGCUUGCAAUUUUGAU
KX056898      GAUCAACUGAUCACAUGGACCACUUCUCCCUUGGAGUGCUUGUGAUCCUGCUCAUGGUGCAGGAAGGGCUGAAGAAGAGAAUGACCACAAAGAUCAUCAUAAGCACAUCAAUGGCAGUGCUGGUAGCUAUGAUCCUGGGAGGAUUUUCAAUGAGUGACCUGGCUAAGCUUGCAAUUUUGAU
KX117076      GAUCAACUGAUCACAUGGAUCACUUCUCCCUUGGAGUGCUUGUGAUUCUGCUCAUGGUGCAGGAAGGGCUGAAGAAGAGAAUGACCACAAAGAUCAUCAUAAGCACAUCAAUGGCAGUGCUGGUAGCUAUGAUCCUGGGAGGAUUUUCAAUGAGUGACCUGGCUAAGCUUGCAAUUUUGAU
KX185891      GAUCAACUGAUCACAUGGAUCACUUCUCCCUUGGAGUGCUUGUGAUUCUGCUCAUGGUGCAGGAAGGGCUGAAGAAGAGAAUGACCACAAAGAUCAUCAUAAGCACAUCAAUGGCAGUGCUGGUAGCUAUGAUCCUGGGAGGAUUUUCAAUGAGUGACCUGGCUAAGCUUGCAAUUUUGAU
KX247646      GAUCAACUGAUCACAUGGAUCACUUCUCCCUUGGAGUGCUUGUGAUUCUGCUCAUGGUGCAGGAAGGGCUGAAGAAGAGAAUGACCACAAAGAUCAUCAUAAGCACAUCAAUGGCAGUGCUGGUAGCUAUGAUCCUGGGAGGAUUUUCAAUGAGUGACCUGGCUAAGCUUGCAAUCUUGAU
KX262887      GAUCAACUGAUCACAUGGAUCACUUCUCCCUUGGAGUGCUUGUGAUUCUGCUCAUGGUGCAGGAAGGGCUAAAGAAGAGAAUGACCACAAAGAUCAUCAUAAGCACAUCAAUGGCAGUGCUGGUAGCUAUGAUCCUGGGAGGAUUUUCAAUGAGUGACCUGGCUAAGCUUGCAAUUUUGAU
KX280026      GAUCAACUGAUCACAUGGAUCACUUCUCCCUUGGAGUGCUUGUGAUUCUGCUCAUGGUGCAGGAAGGGCUGAAGAAGAGAAUGACCACAAAGAUCAUCAUAAGCACAUCAAUGGCAGUGCUGGUAGCUAUGAUCCUGGGAGGAUUUUCAAUGAGUGACCUGGCUAAGCUUGCAAUUUUGAU
KU758877      GAUCAACUGAUCACAUGGACCACUUCUCCCUUGGAGUGCUUGUGAUCCUGCUCAUGGUGCAGGAAGGGCUGAGGAAGAGAAUGACCACAAAGAUCAUCAUAAGCACAUCAAUGGCAGUGCUGGUAGCUAUGAUCCUGGGAGGAUUUUCAAUGAGUGACCUGGCUAAGCUUGCAAUUUUGAU
```

```
KU866423   GAUCAACUGAUCACAUGGAUCACUUCUCCCUUGGAGUGCUUGUGAUUCUGCUCAUGGUGCAGGAAGGGCUGAAGAAGAGAAUGACCACAAAGAUCAUCAUAAGCACAUCAAUGGCAGUGCUGGUAGCUAUGAUCCUGGGAGGAUUUUCAAUGAGUGACCUGGCUAAGCUUGCAAUUUUGAU
KX377336   GAUCAACUGAUCACAUGGAUCACUUCUCCCUUGGAGUGCUUGUGAUUCUGCUCAUGGUGCAGGAAGGGCUGAAGAAGAGAAUGACCACAAAGAUCAUCAUAAGCACAUCAAUGGCAGUGUUGGUAGCUAUGAUCCUGGGAGGAUUUUCAAUGAGUGACUUGGCUAAGCUUGCAAUUCUGAU
KX377337   GAUCAACUGAUCACAUGGACCACUUCUCCCUUGGAGUGCUUGUGAUCCUGCUCAUGGUGCAGGAAGGGCUGAAGAAGAGAAUGACCACAAAGAUCAUCAUAAGCACAUCAAUGGCAGUGCUGGUAGCUAUGAUCCUGGGAGGAUUUUCAAUGAGUGACCUGGCUAAGCUUGCAAUUUUGAU
KX369547   GAUCAACUGAUCACAUGGAUCACUUCUCCCUUGGAGUGCUUGUGAUUCUGCUCAUGGUGCAGGAAGGGCUGAAGAAGAGAAUGACCACAAAGAUCAUCAUAAGCACAUCAAUGGCAGUGCUGGUAGCUAUGAUCCUGGGAGGAUUUUCAAUGAGUGACCUGGCUAAGCUUGCAAUUUUGAU
KX548902   GAUCAACUGAUCACAUGGAUCACUUCUCCCUUGGAGUGCUUGUGAUUCUGCUCAUGGUGCAGGAAGGGCUGAAGAAGAGAAUGACCACAAAGAUCAUCAUAAGCACAUCAAUGGCAGUGCUGGUAGCUAUGAUCCUGGGAGGAUUUUCAAUGAGUGACCUGGCUAAGCUUGCAAUUUUGAU
KX601166   GAUCAACCGAUCAUAUGGAUCACUUCUCUCUUGGAGUGCUUGUGAUUCUACUCAUGGUGCAGGAAGGUUUGAAGAAGAGAAUGACCACAAAGAUCAUAAUGAGCACAUCAAUGGCAGUGCUGGUAGCCAUGGUCUUGGGAGGAUUCUCAAUGAGUGACCUGGCUAAGCUUGUGAUCCUGAU
KX601167   GAUCAACUGAUCACAUGGAUCACUUCUCCCUUGGAGUGCUUGUGAUUCUGCUCAUGGUGCAGGAAGGGCUGAAGAAGAGAAUGACCACAAAGAUCAUCAUAAGCACAUCAAUGGCAGUGUUGGUAGCUAUGAUCCUGGGAGGAUUUUCAAUGAGUGACUUGGCUAAGCUUGCAAUUCUGAU
KX601168   GAUCAACUGAUCACAUGGACCACUUCUCCCUUGGAGUGCUUGUGAUCCUGCUCAUGGUGCAGGAAGGGCUGAAGAAGAGAAUGACCACAAAGAUCAUCAUAAGCACAUCAAUGGCAGUGCUGGUAGCUAUGAUCCUGGGAGGAUUUUCAAUGAGUGACCUGGCUAAGCUUGCAAUUUUGAU
KX766028   GAUCAACUGAUCACAUGGACCACUUCUCCCUUGGAGUGCUUGUGAUCCUGCUCAUGGUGCAGGAAGGGCUGAAGAAGAGAAUGACCACAAAGAUCAUCAUAAGCACAUCAAUGGCAGUGCUGGUAGCUAUGAUCCUGGGAGGAUUUUCAAUGAGUGACCUGGCUAAGCUUGCAAUUUUGAU
KX766029   GAUCAACUGAUCACAUGGAUCACUUCUCCCUUGGAGUGCUUGUGAUUCUGCUCAUGGUGCAGGAAGGGCUAAAGAAGAGAAUGACCACAAAGAUCAUCAUAAGCACAUCAAUGGCAGUGCUGGUAGCUAUGAUCCUGGGAGGAUUUUCAAUGAGUGACCUGGCUAAGCUUGCAAUUUUGAU
KU940224   GAUCAACUGAUCACAUGGAUCACUUCUCCCUUGGAGUGCUUGUGAUUCUGCUCAUGGUGCAGGAAGGGCUGAAGAAGAGAAUGACCACAAAGAUCAUCAUAAGCACAUCAAUGGCAGUGCUGGUAGCUAUGAUCCUGGGAGGAUUUUCAAUGAGUGACCUGGCUAAGCUUGCAAUUUUGAU


           3333333333333333333333333333333333333333333333333333333333333333333333333333333333333333333333333333333333333333333333333333333333333333333333333333333333333333333333333333333333333
           6666666666666666666666666666666666666666666666666666666666666666666666666666666777777777777777777777777777777777777777777777777777777777777777777777777777777777777777777777777777788
           2222222223333333333444444444455555555556666666666777777777788888888889999999999000000000011111111112222222222333333333344444444445555555555666666666677777777778888888888999999999900
Sequence   1234567890123456789012345678901234567890123456789012345678901234567890123456789012345678901234567890123456789012345678901234567890123456789012345678901234567890123456789012345678901
----------------------------------------------------------------------------------------------------------------------------------------------------------------------------------------------------
NC_012532  GGGUGCUACUUUCGCAGAAAUGAACACUGGAGGAGAUGUAGCUCACUUGGCAUUGGUAGCGGCAUUUAAAGUCAGACCAGCCUUGCUGGUCUCCUUCAUUUUCAGAGCCAAUUGGACACCCCGUGAGAGCAUGCUGCUAGCCCUGGCUUCGUGUCUUCUGCAAACUGCGAUCUCUGCUCUU
KU922960   GGGUGCCACCUUCGCGGAAAUGAACACUGGAGGAGAUGUAGCUCAUCUGGCGCUGACAGCGGCAUUCAAAGUCAGACCAGCGUUGCUGGUAUCUUUCAUCUUCAGAGCUAAUUGGACACCCCGUGAAAGCAUGCUGCUGGCCUUGGCCUCGUGUCUUUUGCAAACUGCGAUCUCUGCCUUG
KU922923   GGGUGCCACCUUCGCGGAAAUGAACACUGGAGGAGAUGUAGCUCAUCUGGCGCUGACAGCGGCAUUCAAAGUCAGACCAGCGUUGCUGGUAUCUUUCAUCUUCAGAGCUAAUUGGACACCCCGUGAAAGCAUGCUGCUGGCCUUGGCCUCGUGUCUUUUGCAAACUGCGAUCUCUGCCUUG
KU820898   GGGUGCCACCUUCGCGGAAAUGAACACUGGAGGAGAUGUAGCUCAUCUGGCGCUGAUAGCGGCAUUCAAAGUCAGACCAGCGUUGCUGGUAUCUUUCAUCUUCAGAGCUAAUUGGACACCCCGUGAAAGCAUGCUGCUGGCCUUGGCCUCGUGUCUUUUGCAAACUGCGAUCUCCGCCUUG
KU321639   GGGUGCCACCUUCGCGGAAAUGAACACUGGAGGAGAUGUAGCUCAUCUGGCGCUGAUAGCGGCAUUCAAAGUCAGACCAGCGUUGCUGGUAUCUUUCAUCUUCAGAGCUAAUUGGACACCCCGUGAAAGCAUGCUGCUGGCCUUGGCCUCGUGUCUUUUGCAAACUGCGAUCUCCGCCUUG
KU820899   GGGUGCCACCUUCGCGGAAAUGAACACUGGAGGAGAUGUAGCUCAUCUGGCGCUGAUAGCGGCAUUCAAAGUCAGACCAGCGUUGCUGGUAUCUUUCAUCUUCAGAGCUAAUUGGACACCCCGUGAAAGCAUGCUGCUGGCCUUGGCCUCGUGUCUUUUGCAAACUGCGAUCUCCGCCUUG
KU853012   GGGUGCCACCUUCGCGGAAAUGAACACUGGAGGAGAUGUAGCUCAUCUGGCGCUGAUAGCGGCAUUCAAAGUCAGACCAGCGUUGCUGGUAUCUUUCAUCUUCAGAGCUAAUUGGACACCCCGUGAAAGCAUGCUGCUGGCCUUGGCCUCGUGUCUUUUGCAAACUGCGAUCUCCGCCUUG
KU729217   GGGUGCCACCUUCGCGGAAAUGAACACUGGAGGAGAUGUAGCUCAUCUGGCGCUGAUAGCGGCAUUCAAAGUCAGACCAGCGUUGCUGGUAUCUUUCAUCUUCAGAGCUAAUUGGACACCCCGUGAAAGCAUGCUGCUGGCCUUGGCCUCGUGUCUUUUGCAAACUGCGAUCUCCGCCUUG
KU729218   GGGUGCCACCUUCGCGGAAAUGAACACUGGAGGAGAUGUAGCUCAUCUGGCGCUGAUAGCGGCAUUCAAAGUCAGACCAGCGUUGCUGGUAUCUUUCAUCUUCAGAGCUAAUUGGACACCCCGUGAAAGCAUGCUGCUGGCCUUGGCCUCGUGUCUUUUGCAAACUGCGAUCUCCGCCUUG
KU761564   GGGUGCCACCUUCGCGGAAAUGAACACUGGAGGAGAUGUAGCUCAUCUGGCGCUGAUAGCGGCAUUCAAAGUCAGACCAGCGUUGCUGGUAUCUUUCAUCUUCAGAGCUAAUUGGACACCCCGUGAAAGCAUGCUGCUGGCCUUGGCCUCGUGUCUUUUGCAAACUGCGAUCUCCGCCUUG
KU740184   GGGUGCCACCUUCGCGGAAAUGAACACUGGAGGAGAUGUAGCUCAUCUGGCGCUGAUAGCGGCAUUCAAAGUCAGACCAGCGUUGCUGGUAUCUUUCAUCUUCAGAGCUAAUUGGACACCCCGUGAAAGCAUGCUGCUGGCCUUGGCCUCGUGUCUUUUGCAAACUGCGAUCUCCGCCUUG
KU744693   GGGUGCCACCUUCGCGGAAAUGAACACUGGAGGAGAUGUAGCUCAUCUGGCGCUGAUAGCGGCAUUCAAAGUCAGACCAGCGUUGCUGGUAUCUUUCAUCUUCAGAGCUAAUUGGACACCCCGUGAAAGCAUGCUGCUGGCCUUGGCCUCGUGUCUUUUGCAAACUGCGAUCUCCGCCUUG
KU497555   GGGUGCCACCUUCGCGGAAAUGAACACUGGAGGAGAUGUAGCUCAUCUGGCGCUGAUAGCGGCAUUCAAAGUCAGACCAGCGUUGCUGGUAUCUUUCAUCUUCAGAGCUAAUUGGACACCCCGUGAAAGCAUGCUGCUGGCCUUGGCCUCGUGUUUUUGCAAACUGCGAUCUCCGCCUUG
KU527068   GGGCGCCACCUUCGCGGAAAUGAACACUGGAGGAGAUGUAGCUCAUCUGGCGCUGAUAGCGGCAUUCAAAGUCAGACCAGCGUUGCUGGUAUCUUUCAUCUUCAGAGCUAAUUGGACACCCCGUGAAAGCAUGCUGCUGGCCUUGGCCUCGUGUCUUUUGCAAACUGCGAUCUCCGCCUUG
KU501217   GGGUGCCACCUUCGCGGAAAUGAACACUGGAGGAGAUGUAGCUCAUCUGGCGCUGAUAGCGGCAUUCAAAGUCAGACCAGCGUUGCUGGUAUCUUUCAUCUUCAGAGCUAAUUGGACACCCCGUGAAAGCAUGCUACUGGCCUUGGCCUCGUGUCUUUUGCAAACUGCGAUCUCCGCCUUG
KU501216   GGGUGCCACCUUCGCGGAAAUGAACACUGGAGGAGAUGUAGCUCAUCUGGCGCUGAUAGCGGCAUUCAAAGUCAGACCAGCGUUGCUGGUAUCUUUCAUCUUCAGAGCUAAUUGGACACCCCGUGAAAGCAUGCUACUGGCCUUGGCCUCGUGUCUUUUGCAAACUGCGAUCUCCGCCUUG
KU501215   GGGUGCCACCUUCGCGGAAAUGAACACUGGAGGAGAUGUAGCUCAUCUGGCGCUGAUAGCGGCAUUCAAAGUCAGACCAGCGUUGCUGGUAUCUUUCAUCUUCAGAGCUAAUUGGACACCCCGUGAAAGCAUGCUGCUGGCCUUGGCCUCGUGUCUUUUGCAAACUGCGAUCUCCGCCUUG
KU365780   GGGUGCCACCUUCGCGGAAAUGAACACUGGAGGAGAUGUAGCUCAUCUGGCGCUGAUAGCGGCAUUCAAAGUCAGACCAGCGUUGCUGGUAUCUUUCAUCUUCAGAGCUAAUUGGACACCCCGUGAAAGCAUGCUGCUGGCCUUGGCCUCGUGUCUUUUGCAAACUGCGAUCUCCGCCUUG
KU365779   GGGUGCCACCUUCGCGGAAAUGAACACUGGAGGAGAUGUAGCUCAUCUGGCGCUGAUAGCGGCAUUCAAAGUCAGACCAGCGUUGCUGGUAUCUUUCAUCUUCAGAGCUAAUUGGACACCCCGUGAAAGCAUGCUGCUGGCCUUGGCCUCGUGUCUUUUGCAAACUGCGAUCUCCGCCUUG
KU707826   GGGUGCCACCUUCGCGGAAAUGAACACUGGAGGAGAUGUAGCUCAUCUGGCGCUGAUAGCGGCAUUCAAAGUCAGACCAGCGUUGCUGGUAUCUUUCAUCUUCAGAGCUAAUUGGACACCCCGUGAAAGCAUGCUGCUGGCCUUGGCCUCGUGUCUUUUGCAAACUGCGAUCUCCGCCUUG
KU365778   GGGUGCCACCUUCGCGGAAAUGAACACUGGAGGAGAUGUAGCUCAUCUGGCGCUGAUAGCGGCAUUCAAAGUCAGACCAGCGUUGCUGGUAUCUUUCAUCUUCAGAGCUAAUUGGACACCCCGUGAAAGCAUGCUGCUGGCCUUGGCCUCGUGUCUUUUGCAAACUGCGAUCUCCGCCUUG
KU365777   GGGUGCCACCUUCGCGGAAAUGAACACUGGAGGAGAUGUAGCUCAUCUGGCGCUGAUAGCGGCAUUCAAAGUCAGACCAGCGUUGCUGGUAUCUUUCAUCUUCAGAGCUAAUUGGACACCCCGUGAAAGCAUGCUGCUGGCCUUGGCCUCGUGUCUUUUGCAAACUGCGAUCUCCGCCUUG
KU312312   GGGUGCCACCUUCGCGGAAAUGAACACUGGAGGAGAUGUAGCUCAUCUGGCGCUGAUAGCGGCAUUCAAAGUCAGACCAGCGUUGCUGGUAUCUUUCAUCUUCAGAGCUAAUUGGACACCCCGUGAAAGCAUGCUGCUGGCCUUGGCCUCGUGUCUUUUGCAAACUGCGAUCUCCGCCUUG
KF268950   GGGUGCCACCUUCGCAGAAAUGAACACUGGAGGAGAUGUGGCUCACUUGGCAUUGGUUGCGGCAUUUAAAGUCAGACCAGCCUUGCUGGUCUCCUUUAUUUUCAGAGCUAAUUGGACACCUCGUGAGAGCAUGCUGCUAGCCCUGGCUUCGUGUCUUUUGCAAACUGCAAUCUCUGCUCUC
KF268949   GGGUGCCACCUUCGCAGAAAUGAACACUGGAGGAGAUGUGGCUCACUUGGCAUUGGUUGCGGCAUUUAAAGUCAGACCAGCCUUGCUGGUCUCCUUUAUUUUCAGAGCUAAUUGGACACCUCGUGAGAGCAUGCUGCUAGCCCUGGCUUCGUGUCUUUUACAAACUGCAAUCUCUGCUCUU
KF268948   GGGUGCCACCUUCGCAGAAAUGAACACUGGAGGAGAUGUGGCUCACUUGGCAUUGGUUGCGGCAUUUAAAGUCAGACCAGCCUUGCUGGUCUCCUUUAUUUUCAGAGCUAAUUGGACACCUCGUGAGAGCAUGCUGCUAGCCCUGGCUUCGUGUCUUUUGCAAACUGCAAUCUCUGCUCUC
KJ776791   GGGUGCCACCUUCGCGGAAAUGAACACUGGAGGAGAUGUAGCUCAUCUGGCGCUGAUAGCGGCAUUCAAAGUCAGACCAGCGUUGCUGGUAUCUUUCAUCUUCAGAGCUAAUUGGACACCCCGUGAAAGCAUGCUGCUGGCCUUGGCCUCGUGUCUUUUGCAAACUGCGAUCUCCGCCUUG
KF383119   GGGUGCUACUUUCGCAGAAAUGAACACUGGAGGAGAUGUAGCUCACUUGGCAUUGGUAGCGGCAUUUAAAGUCAGACCAGCCUUGCUGGUCUCCUUCAUUCUCAGAGCCAAUUGGACACCCCGUGAGAGCAUGCUGCUAGCCCUGGCUUCGUGUCUUCUGCAAACUGCGAUCUCUGCUCUU
KF383118   GGGUGCUACUUUCGCAGAAAUGAACACUGGAGGAGAUGUAGCUCACUUGGCAUUGGUAGCGGCAUUUAAAGUCAGACCAGCCUUGCUGGUCUCCUUCAUUCUCAGAGCCAAUUGGACACCCCGUGAGAGCAUGCUGCUAGCCCUGGCUUCGUGUCUUCUGCAAACUGCGAUCUCUGCUCUU
KF383117   GGGUGCCACUUUCGCAGAAAUGAACACUGGAGGAGAUGUGGCUCACUUGGCAUUGGUUGCGGCAUUUAAAGUCAGACCAGCCUUGUUGGUUUCCUUUAUCUUCAGAGCCAACUGGACACCCCGUGAGAGCAUGCUGCUAGCCCUGGCUUCGUGUCUCCUGCAGACUGCGAUCUCCGCUCUU
KF383116   GGGUGCCACUUUCGCAGAAAUGAACACUGGAGGAGAUGUGGCUCACUUGGCAUUGGUUGCGGCAUUUAAAGUCAGACCAGCCUUGUUGGUUUCCUUCAUCUUCAGAGCCAACUGGACACCCCGUGAGAGCAUGCUGCUAGCCCUGGCUUCGUGUCUCCUGCAGACUGCGAUCUCCGCUCUU
KF383115   GGGUGCUACUUUCGCAGAAAUGAAUACUGGAGGAGAUGUGGCUCACUUGGCAUUGGUCGCGGCAUUCAAAGUCAGACCAGCCUUGCUGGUCUCCUUCAUUUUCAGAGCCAAUUGGACACCCCGUGAGAGCAUGCUGCUAGCCCUGGCUUCGUGUCUUUUGCAAACUGCAAUUUCAGCUCUU
EU545988   GGGUGCCACCUUUGCGGAAAUGAACACUGGAGGAGAUGUAGCUCAUCUGGCGCUGAUAGCGGCAUUCAAAGUCAGACCUGCGUUGCUGGUAUCUUUCAUCUUCAGAGCUAAUUGGACACCCCGUGAGAGCAUGCUGCUGGCCCUGGCUUCGUGUCUUCUGCAAACUGCGAUCUCCGCCUUG
KU955589   GGGUGCCACCUUCGCGGAAAUGAACACUGGAGGAGAUGUAGCUCAUCUGGCGCUGAUAGCGGCAUUCAAAGUCAGACCAGCGUUGCUGGUAUCUUUCAUCUUCAGAGCUAAUUGGACACCCCGUGAAAGCAUGCUGCUGGCCUUGGCCUCGUGUCUUUUGCAAACUGCGAUCUCCGCCUUG
KU955590   GGGUGCCACCUUCGCGGAAAUGAACACUGGAGGAGAUGUAGCUCAUCUGGCGCUGAUAGCGGCAUUCAAAGUCAGACCAGCGUUGCUGGUAUCUUUCAUCUUCAGAGCUAAUUGGACACCCCGUGAAAGCAUGCUGCUGGCCUUGGCCUCGUGUCUUUUGCAAACUGCGAUCUCCGCCUUG
KU940228   GGGUGCCACCUUCGCGGAAAUGAACACUGGAGGAGAUGUAGCUCAUCUGGCGCUGAUAGCGGCAUUCAAAGUCAGACCAGCGUUGCUGGUAUCUUUCAUCUUCAGAGCUAAUUGGACACCCCGUGAAAGCAUGCUGCUGGCCUUGGCCUCGUGUCUUUUGCAAACUGCGAUCUCCGCCUUG
KU991811   GGGUGCCACCUUCGCGGAAAUGAACACUGGAGGAGAUGUAGCUCAUCUGGCGCUGAUAGCGGCAUUCAAAGUCAGACCAGCGUUGCUGGUAUCUUUCAUCUUCAGAGCUAAUUGGACACCCCGUGAAAGCAUGCUGCUGGCCUUGGCCUCGUGUCUUUUGCAAACUGCGAUCUCCGCCUUG
KU509998   GGGUGCCACCUUCGCGGAAAUGAACACUGGAGGAGAUGUAGCUCAUCUGGCGCUGAUAGCGGCAUUCAAAGUCAGACCAGCGUUGCUGGUAUCUUUCAUCUUCAGAGCUAAUUGGACACCCCGUGAAAGCAUGCUGCUGGCCUUGGCCUCGUGUCUUUUGCAAACUGCGAUCUCCGCCUUG
KX056898   GGGUGCCACCUUCGCGGAAAUGAACACUGGAGGAGAUGUAGCUCAUCUGGCGCUGAUAGCGGCAUUCAAAGUCAGACCAGCGUUGCUGGUAUCUUUCAUCUUCAGAGCUAAUUGGACACCCCGUGAAAGCAUGCUGCUGGCCUUGGCCUCGUGUCUUUUGCAAACUGCGAUCUCCGCCUUG
KX117076   GGGUGCCACCUUCGCGGAAAUGAACACUGGAGGAGAUGUAGCUCAUCUGGCGCUGAUAGCGGCAUUCAAAGUCAGACCAGCGUUGCUGGUAUCUUUCAUCUUCAGAGCUAAUUGGACACCCCGUGAAAGCAUGCUGCUGGCCUUGGCCUCGUGUCUUUUGCAAACUGCGAUCUCCGCCUUG
KX185891   GGGUGCCACCUUCGCGGAAAUGAACACUGGAGGAGAUGUAGCUCAUCUGGCGCUGAUAGCGGCAUUCAAAGUCAGACCAGCGUUGCUGGUAUCUUUCAUCUUCAGAGCUAAUUGGACACCCCGUGAAAGCAUGCUGCUGGCCUUGGCCUCGUGUCUUUUGCAAACUGCGAUCUCCGCCUUG
KX247646   GGGUGCCACCUUCGCGGAAAUGAACACUGGAGGAGAUGUAGCUCAUCUGGCGCUGAUAGCGGCAUUCAAAGUCAGACCAGCGUUGCUGGUAUCCUUCAUCUUCAGAGCUAAUUGGACACCCCGUGAAAGCAUGCUGCUGGCCUUGGCCUCGUGUCUUUUGCAAACUGCGAUCUCCGCCUUG
KX262887   GGGUGCCACCUUCGCGGAAAUGAACACUGGAGGAGAUGUAGCUCAUCUGGCGCUGAUAGCGGCAUUCAAAGUCAGACCAGCGUUGCUGGUAUCUUUCAUCUUCAGAGCUAAUUGGACACCCCGUGAAAGCAUGCUACUGGCCUUGGCCUCGUGUCUUUUGCAAACUGCGAUCUCCGCCUUG
KX280026   GGGUGCCACCUUCGCGGAAAUGAACACUGGAGGAGAUGUAGCUCAUCUGGCGCUGAUAGCGGCAUUCAAAGUCAGACCAGCGUUGCUGGUAUCUUUCAUCUUCAGAGCUAAUUGGACACCCCGUGAAAGCAUGCUGCUGGCCUUGGCCUCGUGUCUUUUGCAAACUGCGAUCUCCGCCUUG
KU758877   GGGUGCCACCUUCGCGGAAAUGAACACUGGAGGAGAUGUAGCUCAUCUGGCGCUGAUAGCGGCAUUCAAAGUCAGACCAGCGUUGCUGGUAUCUUUCAUCUUCAGAGCUAAUUGGACACCCCGUGAAAGCAUGCUGCUGGCCUUGGCCUCGUGUCUUUUGCAAACUGCGAUCUCCGCCUUG
KU866423   GGGUGCCACCUUCGCGGAAAUGAACACUGGAGGAGAUGUAGCUCAUCUGGCGCUGAUAGCGGCAUUCAAAGUCAGACCAGCGUUGCUGGUAUCUUUCAUCUUCAGAGCUAAUUGGACACCCCGUGAAAGCAUGCUGCUGGCCUUGGCCUCGUGUCUUUUGCAAACUGCGAUCUCCGCCUUG
KX377336   GGGUGCCACCUUCGCGGAAAUGAACACUGGAGGAGAUGUAGCUCAUCUGGCGCUGAUAGCGGCAUUCAAAGUCAGACCCGCGUUGCUGGUCUCUUUCAUCUUCAGAGCCAAUUGGACACCCCGUGAGAGCAUGCUGCUGGCCUUGGCCUCGUGCCUUCUGCAAACUGUGAUCUCCGCCCUG
KX377337   GGGUGCCACCUUCGCGGAAAUGAACACUGGAGGAGAUGUAGCUCAUCUGGCGCUGAUAGCGGCAUUCAAAGUCAGACCAGCGUUGCUGGUAUCUUUCAUCUUCAGAGCUAAUUGGACACCCCGUGAAAGCAUGCUGCUGGCCUUGGCCUCGUGUCUUUUGCAAACUGCGAUCUCCGCCUUG
KX369547   GGGUGCCACCUUCGCGGAAAUGAACACUGGAGGAGAUGUAGCUCAUCUGGCGCUGAUAGCGGCAUUCAAAGUCAGACCAGCGUUGCUGGUAUCUUUCAUCUUCAGAGCUAAUUGGACACCCCGUGAAAGCAUGCUGCUGGCCUUGGCCUCGUGUCUUUUGCAAACUGCGAUCUCCGCCUUG
KX548902   GGGUGCCACCUUCGCGGAAAUGAACACUGGAGGAGAUGUAGCUCAUCUGGCGCUGAUAGCGGCAUUCAAAGUCAAACCAGCGUUGCUGGUAUCUUUCAUCUUCAGAGCUAAUUGGACACCCCGUGAAAGCAUGCUGUUGGCCUUGGCCUCGUGUCUUUUGCAAACUGCGAUCUCUGCCUUG
KX601166   GGGUGCUACUUUCGCAGAAAUGAACACUGGAGGAGAUGUAGCUCACUUGGCAUUGGUUGCGGCAUUUAAAGUCAGACCAGCCUUGUUGGUUUCCUUCAUCUUCAGAGCCAACUGGACACCCCGUGAGAGCAUGCUGCUAGCCCUGGCUUCGUGCCUCCUGCAGACUGCGAUCUCUGCUCUU
KX601167   GGGUGCCACCUUCGCGGAAAUGAACACUGGAGGAGAUGUAGCUCAUCUGGCGCUGAUAGCGGCAUUCAAAGUCAGACCCGCGUUGCUGGUCUCUUUCAUCUUCAGAGCCAAUUGGACACCCCGUGAGAGCAUGCUGCUGGCCUUGGCCUCGUGCCUUCUGCAAACUGCGAUCUCCGCCCUG
KX601168   GGGUGCCACCUUCGCGGAAAUGAACACUGGAGGAGAUGUAGCUCAUCUGGCGCUGAUAGCGGCAUUCAAAGUCAGACCAGCGUUGCUGGUAUCUUUCAUCUUCAGAGCUAAUUGGACACCCCGUGAAAGCAUGCUGCUGGCCUUGGCCUCGUGUCUUUUGCAAACUGCGAUCUCCGCCUUG
KX766028   GGGUGCCACCUUCGCGGAAAUGAACACUGGAGGAGAUGUAGCUCAUCUGGCGCUGAUAGCGGCAUUCAAAGUCAGACCAGCGUUGCUGGUAUCCUUCAUCUUCAGAGCUAAUUGGACACCCCGUGAAAGCAUGCUGCUGGCCUUGGCCUCGUGUCUUUUGCAAACUGCGAUCUCCGCCUUG
KX766029   GGGUGCCACCUUCGCGGAAAUGAACACUGGAGGAGAUGUAGCUCAUCUGGCGCUGAUAGCGGCAUUCAAAGUCAGACCAGCGUUGCUGGUAUCUUUCAUCUUCAGAGCUAAUUGGACACCCCGUGAAAGCAUGCUACUGGCCUUGGCCUCGUGUCUUUUGCAAACUGCGAUCUCCGCCUUG
KU940224   GGGUGCCACCUUCGCGGAAAUGAACACUGGAGGAGAUGUAGCUCAUCUGGCGCUGAUAGCGGCAUUCAAAGUCAGACCAGCGUUGCUGGUAUCUUUCAUCUUCAGAGCUAAUUGGACACCCCGUGAAAGCAUGCUGCUGGCCUUGGCCUCGUGUCUUUUGCAAACUGCGAUCUCCGCCUUG


           3333333333333333333333333333333333333333333333333333333333333333333333333333333333333333333333333333333333333333333333333333333333333333333333333333333333333333333333333333333333333
```

```
           8888888888888888888888888888888888888888888888888888888888888888888888888888888888888888888888888899999999999999999999999999999999999999999999999999999999999999999999999999999999999
           0000000011111111112222222222333333333344444444445555555555666666666677777777778888888888999999999900000000001111111111222222222233333333334444444444555555555566666666667777777777888
Sequence   2345678901234567890123456789012345678901234567890123456789012345678901234567890123456789012345678901234567890123456789012345678901234567890123456789012345678901234567890123456789012
-----------------------------------------------------------------------------------------------------------------------------------------------------------------------------------------------------------
NC_012532  GAAGGUGACUUGAUGGUCCUCAUUAAUGGAUUUGCUUUGGCCUGGUUGGCAAUUCGAGCAAUGGCCGUGCCACGCACUGACAACAUCGCUCUACCAAUCUUGGCUGCUCUAACACCACUAGCUCGAGGCACACUGCUCGUGGCAUGGAGAGCGGCCUGGCUACUUGUGGAGGGAUCAUGC
KU922960   GAAGGCGACCUGAUGGUUCUCAUCAAUGGUUUUGCUUUGGCCUGGUUGGCAAUACGAGCGAUGGUUGUUCCACGCACUGACAACAUCACCUUGGCAAUCCUGGCUGCUCUGACACCACUGGCCCGGGGCACACUGCUUGUGGCGUGGAGAGCAGGCCUUGCUACUUGCGGGGGUUUAUGC
KU922923   GAAGGCGACCUGAUGGUUCUCAUCAAUGGUUUUGCUUUGGCCUGGUUGGCAAUACGAGCGAUGGUUGUUCCACGCACUGACAACAUCACCUUGGCAAUCCUGGCUGCUCUGACACCACUGGCCCGGGGCACACUGCUUGUGGCGUGGAGAGCAGGCCUUGCUACUUGCGGGGGUUUAUGC
KU820898   GAAGGCGACCUGAUGGUUCUCAUCAAUGGUUUUGCUUUGGCCUGGUUGGCAGUACGAGCGAUGGUUGUUCCACGCACUGAUAACAUCACCUUAGCAAUCCUGGCUGCUCUGACACCACUGGCCCGGGGCACACUGCUUGUGGCGUGGAGAGCAGGCCUUGCUACUUGCGGGGGUUUAUGC
KU321639   GAAGGCGACCUGAUGGUUCUCAUCAAUGGUUUUGCUUUGGCCUGGUUGGCAAUACGAGCGAUGGUUGUUCCACGCACUGAUAACAUCACCUUGGCAAUCCUGGCUGCUCUGACACCACUGGCCCGGGGCACACUGCUUGUGGCGUGGAGAGCAGGCCUUGCUACUUGCGGGGGUUUAUGC
KU820899   GAAGGCGACCUGAUGGUUCUCAUCAAUGGUUUUGCUUUGGCCUGGUUGGCAAUACGAGCGAUGGUUGUUCCACGCACUGAUAACAUCACCUUGGCAAUCCUGGCUGCUCUGACACCACUGGCCCGGGGCACACUGCUUGUGGCGUGGAGAGCAGGCCUUGCUACUUGCGGGGGUUUAUGC
KU853012   GAAGGCGACCUGAUGGUUCUCAUCAAUGGUUUUGCUUUGGCCUGGUUGGCAAUACGAGCGAUGGUUGUUCCACGCACUGAUAACAUCACCUUGGCAAUCCUGGCUGCUCUGACACCACUGGCCCGGGGCACACUGCUUGUGGCGUGGAGAGCAGGCCUUGCUACUUGCGGGGGUUUAUGC
KU729217   GAAGGCGACCUGAUGGUUCUCAUCAAUGGUUUUGCUUUGGCCUGGUUGGCAAUACGAGCGAUGGUUGUUCCACGCACUGAUAACAUCACCUUGGCAAUCCUGGCUGCUCUGACACCACUGGCCCGGGGCACACUGCUUGUGGCGUGGAGAGCAGGCCUUGCUACUUGCGGGGGUUUAUGC
KU729218   GAAGGCGACCUGAUGGUUCUCAUCAAUGGUUUUGCUUUGGCCUGGUUGGCAAUACGAGCGAUGGUUGUUCCACGCACUGAUAACAUCACCUUGGCAAUCCUGGCUGCUCUGACACCACUGGCCCGGGGCACACUGCUUGUGGCGUGGAGAGCAGGCCUUGCUACUUGCGGGGGUUUAUGC
KU761564   GAAGGCGACCUGAUGGUUCUCAUCAAUGGUUUUGCUUUGGCCUGGUUGGCAGUACGAGCGAUGGUUGUUCCACGCACUGAUAACAUCACCUUAGCAAUCCUGGCUGCUCUGACACCACUGGCCCGGGGCACACUGCUUGUGGCGUGGAGAGCAGGCCUUGCUACUUGCGGGGGUUUAUGC
KU740184   GAAGGCGACCUGAUGGUUCUCAUCAAUGGUUUUGCUUUGGCCUGGUUGGCAGUACGAGCGAUGGUUGUUCCACGCACUGAUAACAUCACCUUAGCAAUCCUGGCUGCUCUGACACCACUGGCCCGGGGCACACUGCUUGUGGCGUGGAGAGCAGGCCUUGCUACUUGCGGGGGUUUAUGC
KU744693   GAAGGCGACCUGAUGGUUCUCAUCAAUGGUUUUGCUUUGGCCUGGUUGGCAAUACGAGCGAUGGUUGUUCCACGCACUGAUAACAUCACCUUAGCAAUCCUGGCUGCUCUGACACCACUGGCCCGGGGCACACUGCUUGUGGCGUGGAGAGCAGGCCUUGCUACUUGCGGGGGUUUAUGC
KU497555   GAAGGCGACCUGAUGGUUCUCAUCAAUGGUUUUGCUUUGGCCUGGUUGGCAAUACGAGCGAUGGUUGUUCCACGCACUGACAACAUCACCUUGGCAAUCCUGGCUGCUCUGACACCACUGGCCCGGGGCACACUGCUUGUGGCGUGGAGAGCAGGCCUUGCUACUUGCGGGGGUUUAUGC
KU527068   GAAGGCGACCUGAUGGUUCUCAUCAAUGGUUUUGCUUUGGCCUGGUUGGCAAUACGAGCGAUGGUUGUUCCACGCACUGAUAACAUCACCUUGGCAAUCCUGGCUGCUCUGACACCACUGGCCCGGGGCACACUGCUUGUGGCGUGGAGAGCAGGCCUUGCUACUUGCGGGGGUUUAUGC
KU501217   GAAGGCGACCUGAUGGUUCUCAUCAAUGGUUUUGCUUUGGCCUGGUUGGCAAUACGAGCGAUGGUUGUUCCACGCACUGAUAACAUCACCUUGGCAAUCCUGGCUGCUCUGACACCACUGGCCCGGGGCACACUGCUUGUGGCGUGGAGAGCAGGCCUUGCUACUUGCGGGGGUUUAUGC
KU501216   GAAGGCGACCUGAUGGUUCUCAUCAAUGGUUUUGCUUUGGCCUGGUUGGCAAUACGAGCGAUGGUUGUUCCACGCACUGAUAACAUCACCUUGGCAAUCCUGGCUGCUCUGACACCACUGGCCCGGGGCACACUGCUUGUGGCGUGGAGAGCAGGCCUUGCUACUUGCGGGGGUUUAUGC
KU501215   GAAGGCGACCUGAUGGUUCUCAUCAAUGGUUUUGCUUUGGCCUGGUUGGCAAUACGAGCGAUGGUUGUUCCACGCACUGAUAACAUCACCUUGGCAAUCCUGGCUGCUCUGACACCACUGGCCCGGGGCACACUGCUUGUGGCGUGGAGAGCAGGCCUUGCUACUUGCGGGGGUUUAUGC
KU365780   GAAGGCGACCUGAUGGUUCUCAUCAAUGGUUUUGCUUUGGCCUGGUUGGCAAUACGAGCGAUGGUUGUUCCACGCACUGAUAACAUCACCUUGGCAAUCCUGGCUGCUCUGACACCACUGGCCCGGGGCACACUGCUUGUGGCGUGGAGAGCAGGCCUUGCUACUUGCGGGGGUUUAUGC
KU365779   GAAGGCGACCUGAUGGUUCUCAUCAAUGGUUUUGCUUUGGCCUGGUUGGCAAUACGAGCGAUGGUUGUUCCACGCACUGAUAACAUCACCUUGGCAAUCCUGGCUGCUCUGACACCACUGGCCCGGGGCACACUGCUUGUGGCGUGGAGAGCAGGCCUUGCUACUUGCGGGGGUUUAUGC
KU707826   GAAGGCGACCUGAUGGUUCUCAUCAAUGGUUUUGCUUUGGCCUGGUUGGCAAUACGAGCGAUGGUUGUUCCACGCACUGAUAACAUCACCUUGGCAAUCCUGGCUGCUCUGACACCACUGGCCCGGGGCACACUGCUUGUGGCGUGGAGAGCAGGCCUUGCUACUUGCGGGGGUUUAUGC
KU365778   GAAGGCGACCUGAUGGUUCUCAUCAAUGGUUUUGCUUUGGCCUGGUUGGCAAUACGAGCGAUGGUUGUUCCACGCACUGAUAACAUCACCUUGGCAAUCCUGGCUGCUCUGACACCACUGGCCCGGGGCACACUGCUUGUGGCGUGGAGAGCAGGCCUUGCUACUUGCGGGGGUUUAUGC
KU365777   GAAGGCGACCUGAUGGUUCUCAUCAAUGGUUUUGCUUUGGCCUGGUUGGCAAUACGAGCGAUGGUUGUUCCACGCACUGAUAACAUCACCUUGGCAAUCCUGGCUGCUCUGACACCACUGGCCCGGGGCACACUGCUUGUGGCGUGGAGAGCAGGCCUUGCUACUUGCGGGGGUUUAUGC
KU312312   GAAGGCGACCUGAUGGUUCUCAUCAAUGGUUUUGCUUUGGCCUGGUUGGCAAUACGAGCGAUGGUUGUUCCACGCACUGAUAACAUCACCUUGGCAAUCCUGGCUGCUCUGACACCACUGGCCCGGGGCACACUGCUUGUGGCGUGGAGAGCAGGCCUUGCUACUUGCGGGGGUUUAUGC
KF268950   GAAGGUGACUUGAUGGUCCUCGUCAAUGGAUUUGCUUUGGCCUGGUUGGCAAUACGAGCAAUGGCCGUGCCACGCACUGACAACAUUGCUCUGGCAAUCCUGGCUGCUCUGACACCACUAGCCCGAGGUACACUGCUCGUGGCAUGGAGAGCGGGCCUCGCUACUUGUGGAGGGUUUAUGC
KF268949   GAAGGUGACUUGAUGGUCCUCGUCAAUGGAUUUGCUUUGGCCUGGUUGGCAAUACGAGCAAUGGCCGUGCCACGCACUGACAACAUUGCUCUAGCAGUCCUGGCUGCUCUGACACCACUAGCCCGAGGCACACUGCUCGUGGCAUGGAGAGCGGGCCUCGCUACUUGUGGAGGGUUCAUGC
KF268948   GAAGGUGACUUGAUGGUCCUCGUCAAUGGAUUUGCUUUGGCCUGGUUGGCAAUACGAGCAAUGGCCGUGCCACGCACUGACAACAUUGCUCUGGCAAUCCUGGCUGCUCUGACACCACUAGCCCGAGGUACACUGCUCGUGGCAUGGAGAGCGGGCCUCGCUACUUGUGGAGGGUUUAUGC
KJ776791   GAAGGCGACCUGAUGGUUCUCAUCAAUGGUUUUGCUUUGGCCUGGUUGGCAAUACGAGCGAUGGUUGUUCCACGCACUGAUAACAUCACCUUGGCAAUCCUGGCUGCUCUGACACCACUGGCCCGGGGCACACUGCUUGUGGCGUGGAGAGCAGGCCUUGCUACUUGCGGGGGUUUAUGC
KF383119   GAAGGUGACUUGAUGGUCCUCGUUAAUGGAUUUGCUUUGGCCUGGUUGGCAAUUCGAGCAAUGGCCGUGCCACGCACUGACAACAUCGCUCUAGCAAUCUUGGCUGCUCUAACACCACUAGCUCGAGGCACACUGCUCGUGGCAUGGAGAGCGGGCCUGGCUACUUGUGGAGGGUUCAUGC
KF383118   GAAGGUGACUUGAUGGUCCUCGUUAAUGGAUUUGCUUUGGCCUGGUUGGCAAUUCGAGCAAUGGCCGUGCCACGCACUGACAACAUCGCUCUAGCAAUCUUGGCUGCUCUAACACCACUAGCUCGAGGCACACUGCUCGUGGCAUGGAGAGCGGGCCUGGCUACUUGUGGAGGGUUCAUGC
KF383117   GAAGGCGAGCUGAUGGUCCUCGUUAAUGGAUUUGCUUUGGCCUGGUUGGCAAUACGAGCAAUGGCCGUGCCACGCACUGACAAUAUCGCUCUAGCAACUCUGGCCGCUUUAACACCAUUGGCCAGAGGCACACUGCUCGUGGCAUGGAGAGCGGGCCUCGCCACUUGUGGAGGGUUCAUGC
KF383116   GAAGGCGAGCUGAUGGUCCUCGUUAAUGGAUUUGCUUUGGCCUGGUUGGCAAUACGAGCAAUGGCCGUGCCACGCACUGACAAUAUCGCUCUAGCAAUUCUGGCUGCUCUAACACCAUUAGCCAGAGGCACACUGCUCGUGGCAUGGAGAGCGGGCCUCGCCACUUGUGGAGGGUUCAUGC
KF383115   GAAGGUGACUUGAUGGUCCUCGUUAAUGGAUUUGCUUUGGCCUGGUUGGCAAUACGAGCAAUGGCCGUGCCACGCACUGACAACAUUGCUCUAGCAAUCCUGGCUGCUCUGACACCACUAGCCCGAGGCACACUGCUCGUGGCAUGGAGAGCGGGCCUCGCCACUUGUGGAGGGUUCAUGC
EU545988   GAAGGCGACCUGAUGGUUCUCAUCAAUGGUUUUGCUUUGGCCUGGUUGGCAAUACGAGCGAUGGUUGUUCCACGCACUGACAACAUCACCUUGGCAAUCCUGACUGCGCUGACACCACUGGCCCGGGGCACGCUGCUUGUGGCGUGGAGAGCAGGCCUUGCUACUUGCGGGGGUUCAUGC
KU955589   GAAGGCGACCUGAUGGUUCUCAUCAAUGGUUUUGCUUUGGCCUGGUUGGCAAUACGAGCGAUGGUUGUUCCACGCACUGAUAACAUCACCUUGGCAAUCCUGGCUGCUCUGACACCACUGGCCCGGGGCACACUGCUUGUGGCGUGGAGAGCAGGCCUUGCUACUUGCGGGGGUUUAUGC
KU955590   GAAGGCGACCUGAUGGUUCUCAUCAAUGGUUUUGCUUUGGCCUGGUUGGCAGUACGAGCGAUGGUUGUUCCACGCACUGAUAACAUCACCCUAGCAAUCCUGGCUGCUCUGACACCACUGGCCCGGGGCACACUGCUUGUGGCGUGGAGAGCAGGCCUUGCUACUUGCGGGGGUUUAUGC
KU940228   GAAGGCGACCUGAUGGUUCUCAUCAAUGGUUUUGCUUUGGCCUGGUUGGCAAUACGAGCGAUGGUUGUUCCACGCACUGAUAACAUCACCUUGGCAAUCUUGGCUGCUCUGACACCACUGGCCCGGGGCACACUGCUUGUGGCGUGGAGAGCAGGCCUUGCUACUUGCGGGGGUUUAUGC
KU991811   GAAGGCGACCUGAUGGUUCUCAUCAAUGGUUUUGCUUUGGCCUGGUUGGCAAUACGAGCGAUGGUUGUUCCACGCACUGACAACAUCACCUUGGCAAUCCUGGCUGCUCUGACACCACUGGCCCGGGGCACACUGCUUGUGGCGUGGAGAGCAGGCCUUGCUACUUGCGGGGGUUUAUGC
KU509998   GAAGGCGACCUGAUGGUUCUCAUCAAUGGUUUUGCUUUGGCCUGGUUGGCAAUACGAGCGAUGGUUGUUCCACGCACUGAUAACAUCACCUUGGCAAUCCUGGCUGCUCUGACACCACUGGCCCGGGGCACACUGCUUGUGGCGUGGAGAGCAGGCCUUGCUACUUGCGGGGGUUUAUGC
KX056898   GAAGGCGACCUGAUGGUUCUCAUCAAUGGUUUUGCUUUGGCCUGGUUGGCAGUACGAGCGAUGGUUGUUCCACGCACUGAUAACAUCACCCUAGCAAUCCUGGCUGCUCUGACACCACUGGCCCGGGGCACACUGCUUGUGGCGUGGAGAGCAGGCCUUGCUACUUGCGGGGGUUUAUGC
KX117076   GAAGGCGACCUGAUGGUUCUCAUCAAUGGUUUUGCUUUGGCCUGGUUGGCAAUACGAGCGAUGGUUGUUCCACGCACUGAUAACAUCACCUUGGCAAUCCUGGCUGCUCUGACACCACUGGCCCGGGGCACACUGCUUGUGGCGUGGAGAGCAGGCCUUGCUACUUGCGGGGGUUUAUGC
KX185891   GAAGGCGACCUGAUGGUUCUCAUCAAUGGUUUUGCUUUGGCCUGGUUGGCAAUACGAGCGAUGGUUGUUCCACGCACUGAUAACAUCACCUUGGCAAUCCUGGCUGCUCUGACACCACUGGCCCGGGGCACACUGCUUGUGGCGUGGAGAGCAGGCCUUGCUACUUGCGGGGGUUUAUGC
KX247646   GAGGGCGACCUGAUGGUUCUCAUCAAUGGUUUUGCUUUGGCCUGGUUGGCAAUACGAGCGAUGGUUGUUCCACGCACUGACAACAUCACCUUGGCAAUCCUGGCUGCUCUGACACCACUGGCCCGGGGCACACUGCUUGUGGCGUGGAGAGCAGGCCUUGCUACUUGCGGGGGUUUAUGC
KX262887   GAAGGCGACCUGAUGGUUCUCAUCAAUGGUUUUGCUUUGGCCUGGUUGGCAAUACGAGCGAUGGUUGUUCCACGCACUGAUAACAUCACCUUGGCAAUCCUGGCUGCUCUGACACCACUGGCCCGGGGCACACUGCUUGUGGCGUGGAGAGCAGGCCUUGCUACUUGCGGGGGUUUAUGC
KX280026   GAAGGCGACCUGAUGGUUCUCAUCAAUGGUUUUGCUUUGGCCUGGUUGGCAAUACGAGCGAUGGUUGUUCCACGCACUGAUAACAUCACCUUGGCAAUCCUGGCUGCUCUGACACCACUGGCCCGGGGCACACUGCUUGUGGCGUGGAGAGCAGGCCUUGCUACUUGCGGGGGUUUAUGC
KU758877   GAAGGCGACCUGAUGGUUCUCAUCAAUGGUUUUGCUUUGGCCUGGUUGGCAAUACGAGCGAUGGUUGUUCCACGCACUGAUAACAUCACCUUGGCAAUCCUGGCUGCUCUGACACCACUGGCCCGGGGCACACUGCUUGUGGCGUGGAGAGCAGGCCUUGCUACUUGCGGGGGUUUAUGC
KU866423   GAAGGCGACCUGAUGGUUCUCAUCAAUGGUUUUGCUUUGGCCUGGUUGGCAAUACGAGCGAUGGUUGUUCCACGCACUGAUAACAUCACCUUGGCAAUCCUGGCUGCUCUGACACCACUGGCCCGGGGCACACUGCUUGUGGCGUGGAGAGCAGGCCUUGCUACUUGCGGGGGUUUAUGC
KX377336   GAAGGCGACCUGAUGGUUCUCAUCAAUGGUUUUGCUUUGGCCUGGUUGGCAAUACGAGCGAUGGCUGUUCCACGCACUGACAACAUCACCUUGGCAAUCCUGGCUGCUCUGACACCACUGGCCCGAGGCACACUGCUUGUAGCGUGGAGAGCAGGCCUUGCUACUUGUGGGGGGUUCAUGC
KX377337   GAAGGCGACCUGAUGGUUCUCAUCAAUGGUUUUGCUUUGGCCUGGUUGGCAAUACGAGCGAUGGUUGUUCCACGCACUGAUAACAUCACCUUGGCAAUCCUGGCUGCUCUGACACCACUGGCCCGGGGCACACUGCUUGUGGCGUGGAGAGCAGGCCUUGCUACUUGCGGGGGUUUAUGC
KX369547   GAAGGCGACCUGAUGGUUCUCAUCAAUGGUUUUGCUUUGGCCUGGUUGGCAAUACGAGCGAUGGUUGUUCCACGCACUGAUAACAUCACCUUGGCAAUCCUGGCUGCUCUGACACCACUGGCCCGGGGCACACUGCUUGUGGCGUGGAGAGCAGGCCUUGCUACUUGCGGGGGUUUAUGC
KX548902   GAAGGCGACCUGAUGGUUCUCAUCAAUGGUUUUGCUUUGGCCUGGUUGGCAAUACGAGCGAUGGUUGUUCCACGCACUGACAACAUCACCUUGGCAAUCCUGGCUGCUCUGACACCACUGGCCCGGGGCACACUGCUUGUGGCGUGGAGAGCAGGCCUUGCUACUUGCGGGGGUUUAUGC
KX601166   GAAGGCGAGCUGAUGGUCCUCGUUAAUGGAUUUGCUUUGGCCUGGUUGGCAAUACGAGCAAUGGCCGUGCCACGCACUGACAAUAUCGCUCUGGCAAUUCUGGCUGCUCUAACACCAUUAGCCAGAGGCACACUGCUCGUGGCAUGGAGAGCGGGCCUCGCCACUUGUGGAGGGUUCAUGC
KX601167   GAAGGCGACCUGAUGGUUCUCAUCAAUGGUUUUGCUUUGGCCUGGUUGGCAAUACGAGCGAUGGCUGUUCCACGCACUGACAACAUCACCUUGGCAAUCCUGGCUGCUCUGACACCACUGGCCCGAGGCACACUGCUUGUAGCGUGGAGAGCAGGCCUUGCUACUUGUGGGGGGUUCAUGC
KX601168   GAAGGCGACCUGAUGGUUCUCAUCAAUGGUUUUGCUUUGGCCUGGUUGGCAAUACGAGCGAUGGUUGUUCCACGCACUGAUAACAUCACCUUGGCAAUCCUGGCUGCUCUGACACCACUGGCCCGGGGCACACUGCUUGUGGCGUGGAGAGCAGGCCUUGCUACUUGCGGGGGUUUAUGC
KX766028   GAAGGCGACCUGAUGGUUCUCAUCAAUGGUUUUGCUUUGGCCUGGUUGGCAGUACGAGCGAUGGUUGUUCCACGCACUGAUAACAUCACCUUAGCAAUCCUGGCUGCUCUGACACCACUGGCCCGGGGCACACUGCUUGUGGCGUGGAGAGCAGGCCUUGCCACUUGCGGGGGUUUAUGC
KX766029   GAAGGCGACCUGAUGGUUCUCAUCAAUGGUUUUGCUUUGGCCUGGUUGGCAAUACGAGCGAUGGUUGUUCCACGCACUGAUAACAUCACCUUGGCAAUCCUGGCUGCUCUGACACCACUGGCCCGGGGCACACUGCUUGUGGCGUGGAGAGCAGGCCUUGCUACUUGCGGGGGUUUAUGC
KU940224   GAAGGCGACCUGAUGGUUCUCAUCAAUGGUUUUGCUUUGGCCUGGUUGGCAAUACGAGCGAUGGUUGUUCCACGCACUGAUAACAUCACCUUGGCAAUCUUGGCUGCUCUGACACCACUGGCCCGGGGCACACUGCUUGUGGCGUGGAGAGCAGGCCUUGCUACUUGCGGGGGUUUAUGC


           3333333333333333344444444444444444444444444444444444444444444444444444444444444444444444444444444444444444444444444444444444444444444444444444444444444444444444444444444444444
           9999999999999999900000000000000000000000000000000000000000000000000000000000000000000000000000000000000000000000000001111111111111111111111111111111111111111111111111111111111111111
           8888888999999999900000000001111111111222222222233333333334444444444555555555566666666667777777777888888888899999999990000000000111111111122222222223333333333444444444455555555556666
Sequence   3456789012345678901234567890123456789012345678901234567890123456789012345678901234567890123456789012345678901234567890123456789012345678901234567890123456789012345678901234567890123
-----------------------------------------------------------------------------------------------------------------------------------------------------------------------------------------------------------
NC_012532  UCCUCUCCCUGAAAGGGAAAGGUAGUGUGAAGAAGAACCUGCCAUUUGUCAUGGCCCUGGGAUUGACAGCUGUGAGGGUAGUAGACCCUAUUAAUGUGGUAGGACUACUGUUACUCACAAGGAGUGGGAAGCGGAGCUGGCCCCCUAGUGAAGUUCUCACAGCCGUUGGCCUGAUAUGUGC
KU922960   UCCUCUCUCUGAAGGGAAAAGGCAGUGUGAAGAAGAACUUACCAUUUGUCAUGGCCCUGGGACUAACCGCUGUGAGGCUGGUCGACCCCAUCAACGUGGUGGGACUGCUGUUGCUCACAAGGAGUGGGAAGCGGAGCUGGCCCCCUAGCGAAGUACUCACAGCUGUUGGCCUGAUAUGUGC
KU922923   UCCUCUCUCUGAAGGGAAAAGGCAGUGUGAAGAAGAACUUACCAUUUGUCAUGGCCCUGGGACUAACCGCUGUGAGGCUGGUCGACCCCAUCAACGUGGUGGGACUGCUGUUGCUCACAAGGAGUGGGAAGCGGAGCUGGCCCCCUAGCGAAGUACUCACAGCUGUUGGCCUGAUAUGUGC
KU820898   UCCUCUCUCUGAAGGGAAAAGGCAGUGUGAAGAAGAACUUACCAUUUGUCAUGGCCCUGGGACUAACCGCUGUGAGGCUGGUCGACCCCAUCAACGUGGUGGGACUGCUGUUGCUCACAAGGAGUGGGAAGCGGAGCUGGCCCCCUAGCGAAGUACUCACAGCUGUUGGCCUGAUAUGCGC
KU321639   UCCUCUCUCUGAAGGGAAAAGGCAGUGUGAAGAAGAACUUACCAUUUGUCAUGGCCCUGGGACUAACCGCUGUGAGGCUGGUCGACCCCAUCAACGUGGUGGGGCUGCUGUUGCUCACAAGGAGUGGGAAGCGGAGCUGGCCCCCUAGCGAAGUACUCACAGCUGUUGGCCUGAUAUGCGC
KU820899   UCCUCUCUCUGAAGGGAAAAGGCAGUGUGAAGAAGAACUUACCAUUUGUCAUGGCCCUGGGACUAACCGCUGUGAGGCUGGUCGACCCCAUCAACGUGGUGGGACUGCUGUUGCUCACAAGGAGUGGGAAGCGGAGCUGGCCCCCUAGCGAAGUACUCACAGCUGUUGGCCUGAUAUGCGC
KU853012   UCCUCUCUCUGAAGGGAAAAGGCAGUGUGAAGAAGAACUUACCAUUUGUCAUGGCCCUGGGACUAACCGCUGUGAGGCUGGUCGACCCCAUCAACGUGGUGGGACUGCUGUUGCUCACAAGGAGUGGGAAGCGGAGCUGGCCCCCUAGCGAAGUACUCACAGCUGUUGGCCUGAUAUGCGC
KU729217   UCCUCUCUCUGAAGGGAAAAGGCAGUGUGAAGAAGAACUUACCAUUUGUCAUGGCCCUGGGACUAACCGCUGUGAGGCUGGUCGACCCCAUCAACGUGGUGGGACUGCUGUUGCUCACAAGGAGUGGGAAGCGGAGCUGGCCCCCUAGCGAAGUACUCACAGCUGUUGGCCUGAUAUGCGC
KU729218   UCCUCUCUCUGAAGGGAAAAGGCAGUGUGAAGAAGAACUUACCAUUUGUCAUGGCCCUGGGACUAACCGCUGUGAGGCUGGUCGACCCCAUCAACGUGGUGGGACUGCUGUUGCUCACAAGGAGUGGGAAGCGGAGCUGGCCCCCUAGCGAAGUACUCACAGCUGUUGGCCUGAUAUGCGC
KU761564   UCCUCUCUCUGAAGGGAAAAGGCAGUGUGAAGAAGAACUUACCAUUUGUCAUGGCCCUGGGACUAACCGCUGUGAGGCUGGUCGACCCCAUCAACGUGGUGGGACUGCUGUUGCUCACAAGGAGUGGGAAGCGGAGCUGGCCCCCUAGCGAAGUACUCACAGCUGUUGGCCUGAUAUGCGC
KU740184   UCCUCUCUCUGAAGGGAAAAGGCAGUGUGAAGAAGAACUUACCAUUUGUCAUGGCCCUGGGACUAACCGCUGUGAGGCUGGUCGACCCCAUCAACGUGGUGGGACUGCUGUUGCUCACAAGGAGUGGGAAGCGGAGCUGGCCCCCUAGCGAAGUACUCACAGCUGUUGGCCUGAUAUGCGC
```

```
KU744693    UCCUCUCUCUGAAGGGAAAAGGCAGUGUGAAGAAGAACUUACCAUUUGUCAUGGCCCUGGGACUAACCGCUGUGAGGCUGGUCGACCCCAUCAACGUGGUGGGACUGCUGUUGCUCACAAGGAGUGGGAAGCGGAGCUGGCCCCCUAGCGAAGUACUCACAGCUGUUGGCCUGAUAUGCGC
KU497555    UCCUCUCUCUGAAGGGAAAAGGCAGUGUGAAGAAGAACUUACCAUUUGUCAUGGCCCUGGGACUAACCGCUGUGAGGCUGGUCGACCCCAUCAACGUGGUGGGACUGCUGUUGCUCACAAGGAGUGGGAAGCGGAGCUGGCCCCCUAGCGAAGUACUCACAGCUGUUGGCCUGAUAUGCGC
KU527068    UCCUCUCUCUGAAGGGAAAAGGCAGUGUGAAGAAGAACUUACCAUUUGUCAUGGCCCUGGGACUAACCGCUGUGAGGCUGGUCGACCCCAUCAACGUGGUGGGACUGCUGUUGCUCACAAGGAGUGGGAAGCGGAGCUGGCCCCCUAGCGAAGUACUCACAGCUGUUGGCCUGAUAUGCGC
KU501217    UCCUCUCUCUGAAGGGAAAAGGCAGUGUGAAGAAGAACUUACCAUUUGUCAUGGCCCUGGGACUAACCGCUGUGAGGCUGGUCGACCCCAUCAACGUGGUGGGACUGCUGUUGCUCACAAGGAGUGGGAAGCGGAGCUGGCCCCCUAGCGAAGUACUCACAGCUGUUGGCCUGAUAUGCGC
KU501216    UCCUCUCUCUGAAGGGAAAAGGCAGUGUGAAGAAGAACUUACCAUUUGUCAUGGCCCUGGGACUAACCGCUGUGAGGCUGGUCGACCCCAUCAACGUGGUGGGACUGCUGUUGCUCACAAGGAGUGGGAAGCGGAGCUGGCCCCCUAGCGAAGUACUCACAGCUGUUGGCCUGAUAUGCGC
KU501215    UCCUCUCUCUGAAGGGAAAAGGCAGUGUGAAGAAGAACUUACCAUUUGUCAUGGCCCUGGGACUAACCGCUGUGAGGCUGGUCGACCCCAUCAACGUGGUGGGACUGCUGUUGCUCACAAGGAGUGGGAAGCGGAGCUGGCCCCCUAGCGAAGUACUCACAGCUGUUGGCCUGAUAUGCGC
KU365780    UCCUCUCUCUGAAGGGAAAAGGCAGUGUGAAGAAGAACUUACCAUUUGUCAUGGCCCUGGGACUAACCGCUGUGAGGCUGGUCGACCCCAUCAACGUGGUGGGACUGCUGUUGCUCACAAGGAGUGGGAAGCGGAGCUGGCCCCCUAGCGAAGUACUCACAGCUGUUGGCCUGAUAUGCGC
KU365779    UCCUCUCUCUGAAGGGAAAAGGCAGUGUGAAGAAGAACUUACCAUUUGUCAUGGCCCUGGGACUAACCGCUGUGAGGCUGGUCGACCCCAUCAACGUGGUGGGACUGCUGUUGCUCACAAGGAGUGGGAAGCGGAGCUGGCCCCCUAGCGAAGUACUCACAGCUGUUGGCCUGAUAUGCGC
KU707826    UCCUCUCUCUGAAGGGAAAAGGCAGUGUGAAGAAGAACUUACCAUUUGUCAUGGCCCUGGGACUAACCGCUGUGAGGCUGGUCGACCCCAUCAACGUGGUGGGACUGCUGUUGCUCACAAGGAGUGGGAAGCGGAGCUGGCCCCCUAGCGAAGUACUCACAGCUGUUGGCCUGAUAUGCGC
KU365778    UCCUCUCUCUGAAGGGAAAAGGCAGUGUGAAGAAGAACUUACCAUUUGUCAUGGCCCUGGGACUAACCGCUGUGAGGCUGGUCGACCCCAUCAACGUGGUGGGACUGCUGUUACUCACAAGGAGUGGGAAGCGGAGCUGGCCCCCUAGCGAAGUACUCACAGCUGUUGGCCUGAUAUGCGC
KU365777    UCCUCUCUCUGAAGGGAAAAGGCAGUGUGAAGAAGAACUUACCAUUUGUCAUGGCCCUGGGACUAACCGCUGUGAGGCUGGUCGACCCCAUCAACGUGGUGGGACUGCUGUUGCUCACAAGGAGUGGGAAGCGGAGCUGGCCCCCUAGCGAAGUACUCACAGCUGUUGGCCUGAUAUGCGC
KU312312    UCCUCUCUCUGAAGGGAAAAGGCAGUGUGAAGAAGAACUUACCAUUUGUCAUGGCCCUGGGACUAACCGCUGUGAGGCUGGUCGACCCCAUCAACGUGGUGGGACUGCUGUUGCUCACAAGGAGUGGGAAGCGGAGCUGGCCCCCUAGCGAAGUACUCACAGCUGUUGGCCUGAUAUGCGC
KF268950    UCCUCUCCCUGAAAGGGAAAGGUAGUGUGAAGAAGAACCUGCCAUUUGUCAUGGCCUUGGGAUUGACUGCUGUGAGGAUAGUAGACCCCAUUAAUGUGGUGGGACUACUGUUACUCACAAGGAGUGGGAAGCGGAGCUGGCCCCCUAGUGAAGUGCUUACAGCUGUUGGCCUGAUAUGUGC
KF268949    UCCUUUCCCUGAAAGGGGAAAGGUAGUGUGAAGAAGAACCUGCCAUUUGUCAUGGCCUUGGGAUUGACCGCUGUGAGGAUAGUAGACCCCAUUAAUGUGGUAGGACUACUGUUACUCACAAGGAGCGGGAAGCGGAGCUGGCCCCCUAGUGAAGUGCUUACAGCUGUUGGCCUGAUAUGUGC
KF268948    UCCUCUCCCUGAAAGGGAAAGGUAGUGUGAAGAAGAACCUGCCAUUUGUCAUGGCCUUGGGAUUGACUGCUGUGAGGAUAGUAGACCCCAUUAAUGUGGUGGGACUACUGUUACUCACAAGGAGUGGGAAGCGGAGCUGGCCCCCUAGUGAAGUGCUUACAGCUGUUGGCCUGAUAUGUGC
KJ776791    UCCUCUCUCUGAAGGGAAAAGGCAGUGUGAAGAAGAACUUACCAUUUGUCAUGGCCCUGGGACUAACCGCUGUGAGGCUGGUCGACCCCAUCAACGUGGUGGGACUGCUGUUGCUCACAAGGAGUGGGAAGCGGAGCUGGCCCCCUAGCGAAGUACUCACAGCUGUUGGCCUGAUAUGCGC
KF383119    UCCUCUCCCUGAAAGGGAAAGGUAGUGUGAAGAAGAACCUGCCAUUUGUCAUGGCCCUGGGAUUGACAGCUGUGAGGGUAGUAGACCCUAUUAAUGUGGUAGGACUACUGUUACUCACAAGGAGUGGGAAGCGGAGCUGGCCCCCUAGUGAAGUUCUCACAGCCGUUGGCCUGAUAUGUGC
KF383118    UCCUCUCCCUGAAAGGGAAAGGUAGUGUGAAGAAGAACCUGCCAUUUGUCAUGGCCCUGGGAUUGACAGCUGUGAGGGUAGUAGACCCUAUUAAUGUGGUAGGACUACUGUUACUCACAAGGAGUGGGAAGCGGAGCUGGCCCCCUAGUGAAGUUCUCACAGCCGUUGGCCUGAUAUGUGC
KF383117    UUCUUUCCCUGAAAGGGAAAGGUAGUGUGAAGAAGAACCUGCCUUUUGUCAUGGCCUUGGGGUUGGCCGCUGUGAGGAUAGUGGACCCCAUCAAUGUGGUAGGACUACUGUUACUCACAAGGAGUGGAAAACGGAGCUGGCCCCCAGUGAAGUGCUUACAGCUGUUGGCCUGAUAUGUGC
KF383116    UCCUUUCCCUGAAAGGGAAAGGUAGUGUGAAGAAGAACCUGCCUUUUGUCAUGGCCUUGGGGUUGACCGCUGUGAGGAUAGUGGACCCCAUCAAUGUGGUAGGACUACUGUUACUCACACGGAGUGGAAAACGGAGCUGGCCCCCUAGUGAAGUGCUUACAGCUGUUGGCCUGAUAUGUGC
KF383115    UCCUCUCCUUGAAAGGAAAAGGUAGUGUGAAGAAGAACCUGCCAUUUGUCAUGGCCUUGGGAAUGACCGCUGUGAGGAUAGUGGACCCCAUUAACGUGGUGGGACUACUACUACUCACAAGGAGUGGGAAGCGGAGCUGGCCCCCAGUGAAGUGCUUACAGCUGUUGGCCUGAUAUGUGC
EU545988    UUCUCUCUCUGAAGGGGAAGGGCAGUGUGAAGAAGAACCUACCAUUUGUCAUGGCCUUGGGACUCACCGCUGUGAGGCUGGUCGACCCCAUCAACGUGGUGGGACUGCUGUUGCUCACAAGGAGUGGGAAGCGGAGCUGGCCCCCUAGUGAAGUACUCACAGCUGUUGGUCUGAUAUGCGC
KU955589    UCCUCUCUCUGAAGGGAAAAGGCAGUGUGAAGAAGAACUUACCAUUUGUCAUGGCCCUGGGACUAACCGCUGUGAGGCUGGUCGACCCCAUCAACGUGGUGGGACUGCUGUUGCUCACAAGGAGUGGGAAGCGGAGCUGGCCCCCUAGCGAAGUACUCACAGCUGUUGGCCUGAUAUGCGC
KU955590    UCCUCUCUCUGAAGGGAAAAGGCAGUGUGAAGAAGAACUUACCAUUUGUCAUGGCCCUGGGACUAACCGCUGUGAGGCUGGUCGACCCCAUCAACGUGGUGGGACUGCUGUUGCUCACAAGGAGUGGGAAGCGGAGCUGGCCCCCUAGCGAAGUACUCACAGCUGUUGGCCUGAUAUGCGC
KU940228    UCCUCUCUCUGAAGGGAAAAGGCAGUGUGAAGAAGAACUUACCAUUUGUCAUGGCCCUGGGACUAACCGCUGUGAGGCUGGUCGACCCCAUCAACGUGGUGGGACUGCUGUUGCUCACAAGGAGUGGGAAGCGGAGCUGGCCCCCUAGCGAAGUACUCACAGCUGUUGGCCUGAUAUGCGC
KU991811    UCCUCUCUCUGAAGGGAAAAGGCAGUGUGAAGAAGAACUUACCAUUUGUCAUGGCCCUGGGACUAACCGCUGUGAGGCUGGUCGACCCCAUCAACGUGGUGGGACUGCUGUUGCUCACAAGGAGUGGGAAGCGGAGCUGGCCCCCUAGCGAAGUACUCACAGCUGUUGGCCUGAUAUGCGC
KU509998    UCCUCUCUCUGAAGGGAAAAGGCAGUGUGAAGAAGAACUUACCAUUUGUCAUGGCCCUGGGACUAACCGCUGUGAGGCUGGUCGACCCCAUCAACGUGGUGGGGCUGCUGUUGCUCACAAGGAGUGGGAAGCGGAGCUGGCCCCCUAGCGAAGUACUCACAGCUGUUGGCCUGAUAUGCGC
KX056898    UCCUCUCUCUGAAGGGAAAAGGCAGUGUGAAGAAGAACUUACCAUUUGUCAUGGCCCUGGGACUAACCGCUGUGAGGCUGGUCGACCCCAUCAACGUGGUGGGACUGCUGUUGCUCACAAGGAGUGGGAAGCGGAGCUGGCCCCCUAGCGAAGUACUCACAGCUGUUGGCCUGAUAUGCGC
KX117076    UCCUCUCUCUGAAGGGAAAAGGCAGUGUGAAGAAGAACUUACCAUUUGUCAUGGCCCUGGGACUAACCGCUGUGAGGCUGGUCGACCCCAUCAACGUGGUGGGACUGCUGUUGCUCACAAGGAGUGGGAAGCGGAGCUGGCCCCCUAGCGAAGUACUCACAGCUGUUGGCCUGAUAUGCGC
KX185891    UCCUCUCUCUGAAGGGAAAAGGCAGUGUGAAGAAGAACUUACCAUUUGUCAUGGCCCUGGGACUAACCGCUGUGAGGCUGGUCGACCCCAUCAACGUGGUGGGACUGCUGUUGCUCACAAGGAGUGGGAAGCGGAGCUGGCCCCCUAGCGAAGUACUCACAGCUGUUGGCCUGAUAUGCGC
KX247646    UCCUCUCUCUGAAGGGAAAAGGCAGUGUGAAGAAGAACUUACCAUUUGUCAUGGCCCUGGGACUAACCGCUGUGAGGCUGGUCGACCCCAUCAACGUGGUGGGACUGCUGUUGCUCACAAGGAGUGGGAAGCGGAGCUGGCCCCCUAGCGAAGUACUCACAGCUGUUGGCCUGAUAUGCGC
KX262887    UCCUCUCUCUGAAGGGAAAAGGCAGUGUGAAGAAGAACUUACCAUUUGUCAUGGCCCUGGGACUAACCGCUGUGAGGCUGGUCGACCCCAUCAACGUGGUGGGACUGCUGUUGCUCACAAGGAGUGGGAAGCGGAGCUGGCCCCCUAGCGAAGUACUCACAGCUGUUGGCCUGAUAUGCGC
KX280026    UCCUCUCUCUGAAGGGAAAAGGCAGUGUGAAGAAGAACUUACCAUUUGUCAUGGCCCUGGGACUAACCGCUGUGAGGCUGGUCGACCCCAUCAACGUGGUGGGACUGCUGUUGCUCACAAGGAGUGGGAAGCGGAGCUGGCCCCCUAGCGAAGUACUCACAGCUGUUGGCCUGAUAUGCGC
KU758877    UCCUCUCUCUGAAGGGAAAAGGCAGUGUGAAGAAGAACUUACCAUUUGUCAUGGCCCUGGGACUAACCGCUGUGAGGCUGGUCGACCCCAUCAACGUGGUGGGACUGCUGUUGCUCACAAGGAGUGGGAAGCGGAGCUGGCCCCCUAGCGAAGUACUCACAGCUGUUGGCCUGAUAUGCGC
KU866423    UCCUCUCUCUGAAGGGAAAAGGCAGUGUGAAGAAGAACUUACCAUUUGUCAUGGCCCUGGGACUAACCGCUGUGAGGCUGGUCGACCCCAUCAACGUGGUGGGACUGCUGUUGCUCACAAGGAGUGGGAAGCGGAGCUGGCCCCCUAGCGAAGUACUCACAGCUGUUGGCCUGAUAUGCGC
KX377336    UCCUCUCUCUGAAGGGGAAAGGUAGUGUGAAGAAGAACCUACCAUUUGUCAUGGCCUUGGGACUAACCGCUGUGAGGCUGGUUGACCCCAUCAACGUGGUGGGACUGCUGUUGCUCACAAGGAGUGGGAAGCGGAGCUGGCCCCCUAGUGAAGUACUCACAGCUGUUGGCCUGAUAUGUGC
KX377337    UCCUCUCUCUGAAGGGAAAAGGCAGUGUGAAGAAGAACUUACCAUUUGUCAUGGCCCUGGGACUAACCGCUGUGAGGCUGGUCGACCCCAUCAACGUGGUGGGACUGCUGUUGCUCACAAGGAGUGGGAAGCGGAGCUGGCCCCCUAGCGAAGUACUCACAGCUGUUGGCCUGAUAUGCGC
KX369547    UCCUCUCUCUGAAGGGAAAAGGCAGUGUGAAGAAGAACUUACCAUUUGUCAUGGCCCUGGGACUAACCGCUGUGAGGCUGGUCGACCCCAUCAACGUGGUGGGACUGCUGUUGCUCACAAGGAGUGGGAAGCGGAGCUGGCCCCCUAGCGAAGUACUCACAGCUGUUGGCCUGAUAUGCGC
KX548902    UCCUCUCUCUGAAGGGAAAAGGCAGUGUGAAGAAGAACUUACCAUUUGUCAUGGCCCUGGGACUAACCGCUGUGAGGCUGGUCGACCCCAUCAACGUGGUGGGACUGCUGUUGCUCACAAGGAGUGGGAAGCGGAGCUGGCCCCCUAGCGAAGUACUCACAGCUGUUGGCCUGAUAUGCGC
KX601166    UCCUUUCCCUGAAAGGGAAAGGUAGUGUGAAGAAGAACCUGCCUUUUGUCAUGGCCUUGGGGUUGACCGCUGUGAGGAUAGUGGACCCCAUCAAUGUGGUAGGACUACUGUUACUCACAAGGAGUGGAAAACGGAGCUGGCCCUCUAGUGAAGUGCUUACAGCUGUUGGCCUGAUAUGUGC
KX601167    UCCUCUCUCUGAAGGGGAAAGGUAGUGUGAAGAAGAACCUACCAUUUGUCAUGGCCUUGGGACUAACCGCUGUGAGGCUGGUUGACCCCAUCAACGUGGUGGGACUGCUGUUGCUCACAAGGAGUGGGAAGCGGAGCUGGCCCCCUAGUGAAGUACUCACAGCUGUUGGCCUGAUAUGUGC
KX601168    UCCUCUCUCUGAAGGGAAAAGGCAGUGUGAAGAAGAACUUACCAUUUGUCAUGGCCCUGGGACUAACCGCUGUGAGGCUGGUCGACCCCAUCAACGUGGUGGGACUGCUGUUGCUCACAAGGAGUGGGAAGCGGAGCUGGCCCCCUAGCGAAGUACUCACAGCUGUUGGCCUGAUAUGCGC
KX766028    UCCUCUCUCUGAAGGGAAAAGGCAGUGUGAAGAAGAACUUACCAUUUGUCAUGGCCCUGGGACUAACCGCUGUGAGGCUGGUCGACCCCAUCAACGUGGUGGGACUGCUGCUGCUCACAAGGAGUGGGAAGCGGAGCUGGCCCCCUAGCGAAGUACUCACAGCUGUUGGCCUGAUAUGCGC
KX766029    UCCUCUCUCUGAAGGGAAAAGGCAGUGUGAAGAAGAACUUACCAUUUGUCAUGGCCCUGGGACUAACCGCUGUGAGGCUGGUCGACCCCAUCAACGUGGUGGGACUGCUGUUGCUCACAAGGAGUGGGAAGCGGAGCUGGCCCCCUAGCGAAGUACUCACAGCUGUUGGCCUGAUAUGCGC
KU940224    UCCUCUCUCUGAAGGGAAAAGGCAGUGUGAAGAAGAACUUACCAUUUGUCAUGGCCCUGGGACUAACCGCUGUGAGGCUGGUCGACCCCAUCAACGUGGUGGGACUGCUGUUGCUCACAAGGAGUGGGAAGCGGAGCUGGCCCCCCAGCGAAGUACUCACAGCUGUUGGCCUGAUAUGCGC


            4444444444444444444444444444444444444444444444444444444444444444444444444444444444444444444444444444444444444444444444444444444444444444444444444444444444444444444444444444444
            1111111111111111111111111111111111112222222222222222222222222222222222222222222222222222222222222222222222222222222222222222222222222222333333333333333333333333333333333333333333333
            6666667777777777888888888899999999990000000000111111111122222222223333333333444444444455555555556666666666777777777788888888889999999999000000000011111111112222222222333333333344444
Sequence    4567890123456789012345678901234567890123456789012345678901234567890123456789012345678901234567890123456789012345678901234567890123456789012345678901234567890123456789012345678901234
-------------------------------------------------------------------------------------------------------------------------------------------------------------------------------------------------------
NC_012532   ACUGGCCGGAGGGUUUGCCAAGGCAGACAUUGAGAUGGCUGGACCCAUGGCUGCAGUAGGCUUGCUAAUUGUCAGCUAUGUGGUCUCGGGAAAGAGUGUGGACAUGUACAUUGAAAGAGCAGGUGACAUCACAUGGGAAAAGGACGCGGAAGUCACUGGAAACAGUCCUCGGCUUGACGUG
KU922960    AUUGGCUGGAGGGUUCGCCAAGGCAGAUAUAGAGAUGGCUGGGCCCAUGGCCGCGGUCGGUCUGCUAAUUGUCAGUUACGUGGUCUCAGGAAAGAGUGUGGACAUGUACAUUGAAAGAGCAGGUGACAUCACAUGGGAAAAAGAUGCGGAAGUCACUGGAAACAGUCCCCGGCUCGACGUG
KU922923    AUUGGCUGGAGGGUUCGCCAAGGCAGAUAUAGAGAUGGCUGGGCCCAUGGCCGCGGUCGGUCUGCUAAUUGUCAGUUACGUGGUCUCAGGAAAGAGUGUGGACAUGUACAUUGAAAGAGCAGGUGACAUCACAUGGGAAAAAGAUGCGGAAGUCACUGGAAACAGUCCCCGGCUCGACGUG
KU820898    AUUGGCUGGAGGGUUCGCCAAGGCAGAUAUAGAGAUGGCUGGGCCCAUGGCCGCGGUCGGUCUGCUAAUUGUCAGUUACGUGGUCUCAGGAAAGAGUGUGGACAUGUACAUUGAAAGAGCAGGUGACAUCACAUGGGAAAAAGAUGCGGAAGUCACUGGAAACAGUCCCCGGCUCGAUGUG
KU321639    AUUGGCUGGAGGGUUCGCCAAGGCAGAUAUAGAGAUGGCUGGGCCCAUGGCCGCGGUCGGUCUGCUAAUUGUCAGUUACGUGGUCUCAGGAAAGAGUGUGGACAUGUACAUUGAAAGAGCAGGUGACAUCACAUGGGAAAAAGAUGCGGAAGUCACUGGAAACAGUCCCCGGCUCGAUGUG
KU820899    AUUGGCUGGAGGGUUCGCCAAGGCAGAUAUAGAGAUGGCUGGGCCCAUGGCCGCGGUCGGUCUGCUAAUUGUCAGUUACGUGGUCUCAGGAAAGAGUGUGGACAUGUACAUUGAAAGAGCAGGUGACAUCACAUGGGAAAAAGAUGCGGAAGUCACUGGAAACAGUCCCCGGCUUGAUGUG
KU853012    AUUGGCUGGAGGGUUCGCCAAGGCAGAUAUAGAGAUGGCUGGGCCCAUGGCCGCAGUCGGUCUGCUAAUUGUCAGUUACGUGGUCUCAGGAAAGAGUGUGGACAUGUACAUUGAAAGAGCAGGUGACAUCACAUGGGAAAAAGAUGCGGAAGUCACUGGAAACAGUCCCCGGCUCGAUGUG
KU729217    AUUGGCUGGAGGGUUCGCCAAGGCAGAUAUAGAGAUGGCUGGGCCCAUAGCCGCGGUCGGUCUGCUAAUUGUCAGUUACGUGGUCUCAGGAAAGAGUGUGGACAUGUACAUUGAAAGAGCAGGUGACAUCACAUGGGAAAAAGAUGCGGAAGUCACUGGAAACAGUCCCCGGCUCGAUGUG
KU729218    AUUGGCUGGAGGGUUCGCCAAGGCAGAUAUAGAGAUGGCUGGGCCCAUGGCCGCGGUCGGUCUGCUAAUUGUCAGUUACGUGGUCUCAGGAAAGAGUGUGGACAUGUACAUUGAAAGAGCAGGUGACAUCACAUGGGAAAAAGAUGCGGAAGUCACUGGAAACAGUCCCCGGCUCGAUGUG
KU761564    AUUGGCUGGAGGGUUCGCCAAGGCAGAUAUAGAGAUGGCUGGGCCCAUGGCCGCGGUCGGUCUGCUAAUUGUCAGUUACGUGGUCUCAGGAAAGAGUGUGGACAUGUACAUUGAAAGAGCAGGUGACAUCACAUGGGAAAAAGAUGCGGAAGUCACUGGAAACAGUCCCCGGCUCGAUGUG
KU740184    AUUGGCUGGAGGGUUCGCCAAGGCAGAUAUAGAGAUGGCUGGGCCCAUGGCCGCGGUCGGUCUGCUAAUUGUCAGUUACGUGGUCUCAGGAAAGAGUGUGGACAUGUACAUUGAAAGAGCAGGUGACAUCACAUGGGAAAAAGAUGCGGAAGUCACUGGAAACAGUCCCCGGCUCGAUGUG
KU744693    AUUGGCUGGAGGGUUCGCCAAGGCAGAUAUAGAGAUGGCUGGGCCCAUGGCCGCGGUCGGUCUGCUAAUUGUCAGUUACGUGGUCUCAGGAAAGAGUGUGGACAUGUACAUUGAAAGAGCAGGUGACAUCACAUGGGAAAAAGAUGCGGAAGUCACUGGAAACAGUCCCCGGCUCGAUGUG
KU497555    AUUGGCUGGAGGGUUCGCCAAGGCAGAUAUAGAGAUGGCUGGGCCCAUGGCCGCGGUCGGUCUGCUAAUUGUCAGUUACGUGGUCUCAGGAAAGAGUGUGGACAUGUACAUUGAAAGAGCAGGUGACAUCACAUGGGAAAAAGAUGCGGAAGUCACUGGAAACAGUCCCCGGCUCGAUGUG
KU527068    AUUGGCUGGAGGGUUCGCCAAGGCAGAUAUAGAGAUGGCUGGGCCCAUGGCCGCGGUCGGUCUGCUAAUUGUCAGUUACGUGGUCUCAGGAAAGAGUGUGGACAUGUACAUUGAAAGAGCAGGUGACAUCACAUGGGAAAAAGAUGCGGAAGUCACUGGAAACAGUCCCCGGCUCGAUGUG
KU501217    AUUGGCUGGAGGGUUCGCCAAGGCAGAUAUAGAGAUGGCUGGGCCCAUGGCCGCGGUCGGUCUGCUAAUUGUCAGUUACGUGGUCUCAGGAAAGAGUGUGGACAUGUACAUUGAAAGAGCAGGUGACAUCACAUGGGAAAAAGAUGCGGAAGUCACUGGAAACAGUCCCCGGCUCGAUGUG
KU501216    AUUGGCUGGAGGGUUCGCCAAGGCAGAUAUAGAGAUGGCUGGGCCCAUGGCCGCGGUCGGUCUGCUAAUUGUCAGUUACGUGGUCUCAGGAAAGAGUGUGGACAUGUACAUUGAAAGAGCAGGUGACAUCACAUGGGAAAAAGAUGCGGAAGUCACUGGAAACAGUCCCCGGCUCGAUGUG
KU501215    AUUGGCUGGAGGGUUCGCCAAGGCAGAUAUAGAGAUGGCUGGGCCCAUGGCCGCGGUCGGUCUGCUAAUUGUCAGUUACGUGGUCUCAGGAAAGAGUGUGGACAUGUACAUUGAAAGAGCAGGUGACAUCACAUGGGAAAAAGAUGCGGAAGUCACUGGAAACAGUCCCCGGCUCGAUGUG
KU365780    AUUGGCUGGAGGGUUCGCCAAGGCAGAUAUAGAGAUGGCUGGGCCCAUGGCCGCGGUCGGUCUGCUAAUUGUCAGUUACGUGGUCUCAGGAAAGAGUGUGGACAUGUACAUUGAAAGAGCAGGUGACAUCACAUGGGAAAAAGAUGCGGAAGUCACUGGAAACAGUCCCCGGCUCGAUGUG
KU365779    AUUGGCUGGAGGGUUCGCCAAGGCAGAUAUAGAGAUGGCUGGGCCCAUGGCCGCGGUCGGUCUGCUAAUUGUCAGUUACGUGGUCUCAGGAAAGAGUGUGGACAUGUACAUUGAAAGAGCAGGUGACAUCACAUGGGAAAAAGAUGCGGAAGUCACUGGAAACAGUCCCCGGCUCGAUGUG
KU707826    AUUGGCUGGAGGGUUCGCCAAGGCAGAUAUAGAGAUGGCUGGGCCCAUGGCCGCGGUCGGUCUGCUAAUUGUCAGUUACGUGGUCUCAGGAAAGAGUGUGGACAUGUACAUUGAAAGAGCAGGUGACAUCACAUGGGAAAAAGAUGCGGAAGUCACUGGAAACAGUCCCCGGCUCGAUGUG
KU365778    AUUGGCUGGAGGGUUCGCCAAGGCAGAUAUAGAGAUGGCUGGGCCCAUGGCCGCGGUCGGUCUGCUAAUUGUCAGUUACGUGGUCUCAGGAAAGAGUGUGGACAUGUACAUUGAAAGAGCAGGUGACAUCACAUGGGAAAAAGAUGCGGAAGUCACUGGAAACAGUCCCCGGCUCGAUGUG
KU365777    AUUGGCUGGAGGGUUCGCCAAGGCAGAUAUAGAGAUGGCUGGGCCCAUGGCCGCGGUCGGUCUGCUAAUUGUCAGUUACGUGGUCUCAGGAAAGAGUGUGGACAUGUACAUUGAAAGAGCAGGUGACAUCACAUGGGAAAAAGAUGCGGAAGUCACUGGAAACAGUCCCCGGCUCGAUGUG
KU312312    AUUGGCUGGAGGGUUCGCCAAGGCAGAUAUAGAGAUGGCUGGGCCCAUGGCCGCGGUCGGUCUGCUAAUUGUCAGUUACGUGGUCUCAGGAAAGAGUGUGGACAUGUACAUUGAAAGAGCAGGUGACAUCACAUGGGAAAAAGAUGCGGAAGUCACUGGAAACAGUCCCCGGCUCGAUGUG
KF268950    ACUGGCCGGAGGCUUUGCCAAGGCAGACAUUGAGAUGGCUGGGCCCAUGGCUGCAGUAGGCUUGCUGAUUGUCAGUUAUGUGGUCUCAGGAAAGAGUGUGGAUAUGUACAUUGAAAGAGCAGGUGACAUCACAUGGGAAAAGGACGCGGAAGUUACUGGAAAUAGUCCUCGGCUUGACGUG
KF268949    ACUGGCCGGAGGGUUUGCCAAGGCAGACAUUGAGAUGGCUGGGCCCAUGGCUGCAGUAGGCUUGCUAAUUGUCAGUUAUGUGGUCUCAGGAAAGAGUGUGGAUAUGUACAUUGAAAGAGCAGGUGACAUCACAUGGGAAAAGGACGCGGAAGUUACUGGAAAUAGUCCUCGGCUUGACGUG
KF268948    ACUGGCCGGAGGCUUUGCCAAGGCAGACAUUGAGAUGGCUGGGCCCAUGGCUGCAGUAGGCUUGCUGAUUGUCAGUUAUGUGGUCUCAGGAAAGAGUGUGGAUAUGUACAUUGAAAGAGCAGGUGACAUCACAUGGGAAAAGGACGCGGAAGUUACUGGAAAUAGUCCUCGGCUUGACGUG
```

```
KJ776791    AUUGGCUGGAGGGUUCGCCAAGGCAGAUAUAGAGAUGGCUGGGCCCAUGGCCGCGGUCGGUCUGCUAAUUGUCAGUUACGUGGUCUCAGGAAAGAGUGUGGACAUGUACAUUGAAAGAGCAGGUGACAUCACAUGGGAAAAAGAUGCGGAAGUCACUGGAAACAGUCCCCGGCUCGAUGUG
KF383119    ACUGGCCGGAGGGUUUGCCAAGGCAGACAUUGAGAUGGCUGGACCCAUGGCUGCAGUAGGCUUGCUAAUUGUCAGCUAUGUGGUCUCGGGAAAGAGUGUGGACAUGUACAUUGAAAGAGCAGGUGACAUCACAUGGGAAAAGGACGCGGAAGUCACUGGAAACAGUCCUCGGCUUGACGUG
KF383118    ACUGGCCGGAGGGUUUGCCAAGGCAGACAUUGAGAUGGCUGGACCCAUGGCUGCAGUAGGCUUGCUAAUUGUCAGCUAUGUGGUCUCGGGAAAGAGUGUGGACAUGUACAUUGAAAGAGCAGGUGACAUCACAUGGGAAAAGGACGCGGAAGUCACUGGAAACAGUCCUCGGCUUGACGUG
KF383117    ACUGGCCGGAGGGUUUGCCAAGGCAGACAUAGAGAUGGCUGGGCCCAUGGCUGCAGUAGGCCUGCUAAUUGUCAGUUAUGUGGUCUCGGGAAAGAGUGUGGACAUGUACAUCGAAAGAGCAGGUGAUAUCACAUGGGAAAAAGACGCGGAAGUCACUGGAAACAGUCCCCGGCUUGACGUG
KF383116    ACUGGCCGGAGGGUUUGCCAAGGCAGAUAUAGAGAUGGCUGGGCCCAUGGCUGCAGUAGGCCUGCUAAUUGUCAGUUAUGUGGUCUCGGGAAAGAGUGUGGACAUGUACAUCGAAAGAGCAGGUGAUAUCACAUGGGAAAAAGAC?CGGAAGUCACUGGAAACAGUCCUCGGCUUGACGUG
KF383115    ACUGGCCGGAGGGUUUGCCAAGGCAGAUUGAGAUGGCUGGGCCCAUGGCUGCAGUAGGCUUGCUAAUUGUCAGUUAUGUGGUCUCAGGAAAGAGUGUGGAUAUGUACAUUGAAAGAGCAGGUGACAUCACAUGGGAAAAAGACGCGGAAGUUACUGGAAAUAGUCCUCGGCUUGACGUG
EU545988    GUUGGCCGGAGGGUUCGCCAAGGCGGAUAUAGAGAUGGCUGGGGCCCAUGGCCGCGGUCGGUCUGCUAAUUGUCAGUUACGUGGUCUCAGGAAAGAGUGUGGACAUGUACAUUGAAAGAGCAGGUGACAUCACAUGGGAAAAAGAUGCGGAAGUCACUGGAAACAGUCCCCGGCUCGAUGUG
KU955589    AUUGGCUGGAGGGUUCGCCAAGGCAGAUAUAGAGAUGGCUGGGCCCAUGGCCGCGGUCGGUCUGCUAAUUGUCAGUUACGUGGUCUCAGGAAAGAGUGUGGACAUGUACAUUGAAAGAGCAGGUGACAUCACAUGGGAAAAAGAUGCGGAAGUCACUGGAAACAGUCCCCGGCUUGAUGUG
KU955590    AUUGGCUGGAGGGUUCGCCAAGGCAGAUAUAGAGAUGGCUGGGCCCAUGGCCGCGGUCGGUCUGCUAAUUGUCAGUUACGUGGUCUCAGGAAAGAGUGUGGACAUGUACAUUGAAAGAGCAGGUGACAUCACAUGGGAAAAAGAUGCGGAAGUCACUGGAAACAGUCCCCGGCUCGAUGUG
KU940228    AUUGGCUGGAGGGUUCGCCAAGGCAGAUAUAGAGAUGGCUGGGCCCAUGGCCGCGGUCGGUCUGCUAAUUGUCAGUUACGUGGUCUCAGGAAAGAGUGUGGACAUGUACAUUGAAAGAGCAGGUGACAUCACAUGGGAAAAAGAUGCGGAAGUCACUGGAAACAGUCCCCGGCUCGAUGUG
KU991811    AUUGGCUGGAGGGUUCGCCAAGGCAGAUAUAGAGAUGGCUGGGCCCAUGGCCGCGGUCGGUCUGCUAAUUGUCAGUUACGUGGUCUCAGGAAAGAGUGUGGACAUGUACAUUGAAAGAGCAGGUGACAUCACAUGGGAAAAAGAUGCGGAAGUCACUGGAAACAGUCCCCGGCUCGAUGUG
KU509998    AUUGGCUGGAGGGUUCGCCAAGGCAGAUAUAGAGAUGGCUGGGCCCAUGGCCGCGGUCGGUCUGCUAAUUGUCAGUUACGUGGUCUCAGGAAAGAGUGUGGACAUGUACAUUGAAAGAGCAGGUGACAUCACAUGGGAAAAAGAUGCGGAAGUCACUGGAAACAGUCCCCGGCUCGAUGUG
KX056898    AUUGGCUGGAGGGUUCGCCAAGGCAGAUAUAGAGAUGGCUGGGCCCAUGGCCGCGGUCGGUCUGCUAAUUGUCAGUUACGUGGUCUCAGGAAAGAGUGUGGACAUGUACAUUGAAAGAGCAGGUGACAUCACAUGGGAAAAAGAUGCGGAAGUCACUGGAAACAGUCCCCGGCUCGAUGUG
KX117076    AUUGGCUGGAGGGUUCGCCAAGGCAGAUAUAGAGAUGGCUGGGCCCAUGGCCGCGGUCGGUCUGCUAAUUGUCAGUUACGUGGUCUCAGGAAAGAGUGUGGACAUGUACAUUGAAAGAGCAGGUGACAUCACAUGGGAAAAAGAUGCGGAAGUCACUGGAAACAGUCCCCGGCUUGAUGUG
KX185891    AUUGGCUGGAGGGUUCGCCAAGGCAGAUAUAGAGAUGGCUGGGCCCAUGGCCGCGGUCGGUCUGCUAAUUGUCAGUUACGUGGUCUCAGGAAAGAGUGUGGACAUGUACAUUGAAAGAGCAGGUGACAUCACAUGGGAAAAAGAUGCGGAAGUCACUGGAAACAGUCCCCGGCUUGAUGUG
KX247646    AUUGGCUGGAGGGUUCGCCAAGGCAGAUAUAGAGAUGGCUGGGCCCAUGGCCGCGGUUGGUCUGCUAAUUGUCAGUUACGUGGUCUCAGGAAAGAGUGUGGACAUGUACAUUGAAAGAGCAGGUGACAUCACAUGGGAAAAAGAUGCGGAAGUCACUGGAAACAGUCCCCGGCUCGAUGUG
KX262887    AUUGGCUGGAGGGUUCGCCAAGGCAGAUAUAGAGAUGGCUGGGCCCAUGGCCGCGGUCGGUCUGCUAAUUGUCAGUUACGUGGUCUCAGGAAAGAGUGUGGACAUGUACAUUGAAAGAGCAGGUGACAUCACAUGGGAAAAAGAUGCGGAAGUCACUGGAAACAGUCCCCGGCUCGAUGUG
KX280026    AUUGGCUGGAGGGUUCGCCAAGGCAGAUAUAGAGAUGGCUGGGCCCAUGGCCGCGGUCGGUCUGCUAAUUGUCAGUUACGUGGUCUCAGGAAAGAGUGUGGACAUGUACAUUGAAAGAGCAGGUGACAUCACAUGGGAAAAAGAUGCGGAAGUCACUGGAAACAGUCCCCGGCUCGAUGUG
KU758877    AUUGGCUGGAGGGUUCGCCAAGGCAGAUAUAGAGAUGGCUGGGCCCAUGGCCGCGGUCGGUCUGCUAAUUGUCAGUUACGUGGUCUCAGGAAAGAGUGUGGACAUGUACAUUGAAAGAGCAGGUGACAUCACAUGGGAAAAAGAUGCGGAAGUCACUGGAAACAGUCCCCGGCUCGAUGUG
KU866423    AUUGGCUGGAGGGUUCGCCAAGGCAGAUAUAGAGAUGGCUGGGCCCAUGGCCGCGGUCGGUCUGCUAAUUGUCAGUUACGUGGUCUCAGGAAAGAGUGUGGACAUGUACAUUGAAAGAGCAGGUGACAUCACAUGGGAAAAAGAUGCGGAAGUCACUGGAAACAGUCCCCGGCUUGAUGUG
KX377336    ACUGGCCGGAGGGUUCGCCAAAGCAGAUAUAGAGAUGGCUGGGCCCAUGGCUGCAGUUGGCCUGCUAAUUGUUAGUUACGUGGUCUCAGGAAAGAGUGUGGACAUGUACAUUGAAAGAGCAGGUGACAUCACAUGGGAAAAAGAUGCGGAAGUUACUGGAAACAGCCCCCGGCUCGAUGUG
KX377337    AUUGGCUGGAGGGUUCGCCAAGGCAGAUAUAGAGAUGGCUGGGCCCAUGGCCGCGGUCGGUCUGCUAAUUGUCAGUUACGUGGUCUCAGGAAAGAGUGUGGACAUGUACAUUGAAAGAGCAGGUGACAUCACAUGGGAAAAAGAUGCGGAAGUCACUGGAAACAGUCCCCGGCUCGAUGUG
KX369547    AUUGGCUGGAGGGUUCGCCAAGGCAGAUAUAGAGAUGGCUGGGCCCAUGGCCGCGGUCGGUCUGCUAAUUGUCAGUUACGUGGUCUCAGGAAAGAGUGUGGACAUGUACAUUGAAAGAGCAGGUGACAUCACAUGGGAAAAAGAUGCGGAAGUCACUGGAAACAGUCCCCGGCUCGAUGUG
KX548902    AUUGGCUGGAGGGUUCGCCAAGGCAGAUAUAGAGAUGGCUGGGCCCAUGGCCGCGGUCGGUCUGCUAAUUGUCAGUUACGUGGUCUCAGGAAAGAGUGUGGACAUGUACAUUGAAAGAGCAGGUGACAUCACAUGGGAAAAAGAUGCGGAAGUCACUGGAAACAGUCCCCGGCUCGAUGUG
KX601166    ACUGGCCGGAGGGUUUGCCAAGGCAGACAUAGAGAUGGCUGGGCCCAUGGCUGCAGUGGGCCUGCUAAUUGUCAGUUAUGUGGUCUCGGGAAAGAGUGUGGACAUGUACAUCGAAAGAGCAGGUGAUAUCACAUGGGAAAAAGACGCGGAAGUCACUGGAAACAGUCCUCGGCUUGACGUG
KX601167    ACUGGCCGGAGGGUUCGCCAAAGCAGAUAUAGAGAUGGCUGGGCCCAUGGCUGCAGUUGGCCUGCUAAUUGUUAGUUACGUGGUCUCAGGAAAGAGUGUGGACAUGUACAUUGAAAGAGCAGGUGACAUCACAUGGGAAAAAGAUGCGGAAGUUACUGGAAACAGCCCCCGGCUCGAUGUG
KX601168    AUUGGCUGGAGGGUUCGCCAAGGCAGAUAUAGAGAUGGCUGGGCCCAUGGCCGCGGUCGGUCUGCUAAUUGUCAGUUACGUGGUCUCAGGAAAGAGUGUGGACAUGUACAUUGAAAGAGCAGGUGACAUCACAUGGGAAAAAGAUGCGGAAGUCACUGGAAACAGUCCCCGGCUCGAUGUG
KX766028    AUUGGCUGGAGGGUUCGCCAAGGCAGAUAUAGAGAUGGCUGGGCCCAUGGCCGCGGUCGGUCUGCUAAUUGUCAGUUACGUGGUCUCAGGAAAGAGUGUGGACAUGUACAUUGAAAGAGCAGGUGACAUCACAUGGGAAAAAGAUGCGGAAGUCACUGGAAACAGUCCCCGGCUCGAUGUG
KX766029    AUUGGCUGGAGGGUUCGCCAAGGCAGAUAUAGAGAUGGCUGGGCCCAUGGCCGCGGUCGGUCUGCUAAUUGUCAGUUACGUGGUCUCAGGAAAGAGUGUGGACAUGUACAUUGAAAGAGCAGGUGACAUCACAUGGGAAAAAGAUGCGGAAGUCACUGGAAACAGUCCCCGGCUCGAUGUG
KU940224    AUUGGCUGGAGGGUUCGCCAAGGCAGAUAUAGAGAUGGCUGGGCCCAUGGCCGCGGUCGGUCUGCUAAUUGUCAGUUACGUGGUCUCAGGAAAGAGUGUGGACAUGUACAUUGAAAGAGCAGGUGACAUCACAUGGGAAAAAGAUGCGGAAGUCACUGGAAACAGUCCCCGGCUCGAUGUG


            4444444444444444444444444444444444444444444444444444444444444444444444444444444444444444444444444444444444444444444444444444444444444444444444444444444444444444444444444444444444444444
            3333333333333333333333333333333333333333333333333333333344444444444444444444444444444444444444444444444444444444444444444444444444444444444444444444444444444444445555555555555555555555
            4444455555555555666666666677777777778888888888999999999900000000001111111111222222222233333333334444444444555555555566666666667777777777888888888899999999990000000000111111111122222
Sequence    5678901234567890123456789012345678901234567890123456789012345678901234567890123456789012345678901234567890123456789012345678901234567890123456789012345678901234567890123456789012345
------------------------------------------------------------------------------------------------------------------------------------------------------------------------------------------------------------------------------
NC_012532   GCACUGGAUGAGAGUGGUGACUUCUCCUUGGUAGAGGAAGAUGGUCCACCCAUGAGAGAGAUCAUACUCAAGGUGGUCCUGAUGGCCAUCUGUGGCAUGAACCCAAUAGCUAUACCUUUUGCUGCAGGAGCGUGGUAUGUGUAUGUGAAGACUGGGAAAAGGAGUGGCGCCCUCUGGGACG
KU922960    GCGCUAGAUGAGAGUGGUGAUUUCUCCCUGGUGGAGGAUGACGGUCCCCCCAUGAGAGAGAUCAUACUCAAGGUGGUCCUGAUGACCAUCUGUGGCAUGAACCCAAUAGCCAUACCCUUUGCAGCUGGAGCGUGGUACGUAUACGUGAAGACUGGAAAAAGGAGUGGUGCUCUAUGGGAUG
KU922923    GCGCUAGAUGAGAGUGGUGAUUUCUCCCUGGUGGAGGAUGACGGUCCCCCCAUGAGAGAGAUCAUACUCAAGGUGGUCCUGAUGACCAUCUGUGGCAUGAACCCAAUAGCCAUACCCUUUGCAGCUGGAGCGUGGUACGUAUACGUGAAGACUGGAAAAAGGAGUGGUGCUCUAUGGGAUG
KU820898    GCGCUAGAUGAGAGUGGUGAUUUCUCCCUGGUGGAGGAUGACGGUCCCCCCAUGAGAGAGAUCAUACUCAAGGUGGUCCUGAUGACCAUCUGUGGCAUGAACCCAAUAGCCAUACCCUUUGCAGCUGGAGCGUGGUACGUAUACGUGAAGACUGGAAAAAGGAGUGGUGCUCUAUGGGAUG
KU321639    GCGCUAGAUGAGAGUGGUGAUUUCUCCCUGGUGGAGGAUGACGGUCCCCCCAUGAGAGAGAUCAUACUCAAGGUGGUCCUGAUGACCAUCUGUGGCAUGAACCCAAUAGCCAUACCCUUUGCAGCUGGAGCGUGGUACGUAUACGUGAAGACUGGAAAAAGGAGUGGUGCUCUAUGGGAUG
KU820899    GCGCUAGAUGAGAGUGGUGAUUUCUCCCUGGUGGAGGAUGACGGUCCCCCCAUGAGAGAGAUCAUACUCAAGGUGGUCCUGAUGACCAUCUGUGGCAUGAACCCAAUAGCCAUACCCUUUGCAGCUGGAGCGUGGUACGUAUACGUGAAGACUGGAAAAAGGAGUGGAGCUCUAUGGGAUG
KU853012    GCGCUAGAUGAGAGUGGUGAUUUCUCCCUGGUGGAGGAUGACGGUCCCCCCAUGAGAGAGAUCAUACUCAAGGUGGUCCUGAUGACCAUCUGUGGCAUGAACCCAAUAGCCAUACCCUUUGCAGCUGGAGCGUGGUACGUAUACGUGAAGACUGGAAAAAGGAGUGGUGCUCUAUGGGAUG
KU729217    GCGCUAGAUGAGAGUGGUGAUUUCUCCCUGGUGGAGGAUGACGGUCCCCCCAUGAGAGAGAUCAUACUCAAGGUGGUCCUGAUGACCAUCUGUGGCAUGAACCCAAUAGCCAUACCCUUUGCAGCUGGAGCGUGGUACGUAUACGUGAAGACUGGAAAAAGGAGUGGUGCUCUAUGGGAUG
KU729218    GCGCUAGAUGAGAGUGGUGAUUUCUCCCUGGUGGAGGAUGACGGUCCCCCCAUGAGAGAGAUCAUACUCAAGGUGGUCCUGAUGACCAUCUGUGGCAUGAACCCAAUAGCCAUACCCUUUGCAGCUGGAGCGUGGUACGUAUACGUGAAGACUGGAAAAAGGAGUGGUGCUCUAUGGGAUG
KU761564    GCGCUAGAUGAGAGUGGUGAUUUCUCCCUGGUGGAGGAUGACGGUCCCCCCAUGAGAGAGAUCAUACUCAAGGUGGUCCUGAUGACCAUCUGUGGCAUGAACCCAAUAGCCAUACCCUUUGCAGCUGGAGCGUGGUACGUAUACGUGAAGACUGGAAAAAGGAGUGGUGCUCUAUGGGAUG
KU740184    GCGCUAGAUGAGAGUGGUGAUUUCUCCCUGGUGGAGGAUGACGGUCCCCCCAUGAGAGAGAUCAUACUCAAGGUGGUCCUGAUGACCAUCUGUGGCAUGAACCCAAUAGCCAUACCCUUUGCAGCUGGAGCGUGGUACGUAUACGUGAAGACUGGAAAAAGGAGUGGUGCUCUAUGGGAUG
KU744693    GCGCUAGAUGAGAGUGGUGAUUUCUCCCUGGUGGAGGAUGACGGUCCCCCCAUGAGAGAGAUCAUACUCAAGGUGGUCCUGAUGACCAUCUGUGGCAUGAACCCAAUAGCCAUACCCUUUGCAGCUGGAGCGUGGUACGUAUACGUGAAGACUGGAAAAAGGAGUGGUGCUCUAUGGGAUG
KU497555    GCGCUAGAUGAGAGUGGUGACUUCUCCCUGGUGGAGGAUGACGGUCCCCCCAUGAGAGAGAUCAUACUCAAGGUGGUCCUGAUGACCAUCUGUGGCAUGAACCCAAUAGCCAUACCCUUUGCAGCUGGAGCGUGGUACGUAUACGUGAAGACUGGAAAAAGGAGUGGUGCUCUAUGGGAUG
KU527068    GCGCUAGAUGAGAGUGGUGAUUUCUCCCUGGUGGAGGAUGACGGUCCCCCCAUGAGAGAGAUCAUACUCAAGGUGGUCCUGAUGACCAUCUGUGGCAUGAACCCAAUAGCCAUACCCUUUGCAGCUGGAGCGUGGUACGUAUACGUGAAGACUGGAAAAAGGAGUGGUGCUCUAUGGGAUG
KU501217    GCGCUAGAUGAGAGUGGUGAUUUCUCCCUGGUGGAGGAUGACGGUCCCCCCAUGAGAGAGAUCAUACUCAAGGUGGUCCUGAUGACCAUCUGUGGCAUGAACCCAAUAGCCAUACCCUUUGCAGCUGGAGCGUGGUACGUAUACGUGAAGACUGGAAAAAGGAGUGGUGCUCUAUGGGAUG
KU501216    GCGCUAGAUGAGAGUGGUGAUUUCUCCCUGGUGGAGGAUGACGGUCCCCCCAUGAGAGAGAUCAUACUCAAGGUGGUCCUGAUGACCAUCUGUGGCAUGAACCCAAUAGCCAUACCCUUUGCAGCUGGAGCGUGGUACGUAUACGUGAAGACUGGAAAAAGGAGUGGUGCUCUAUGGGAUG
KU501215    GCGCUAGAUGAGAGUGGUGAUUUCUCCCUGGUGGAGGAUGACGGUCCCCCCAUGAGAGAGAUCAUACUCAAGGUGGUCCUGAUGACCAUCUGUGGCAUGAACCCAAUAGCCAUACCCUUUGCAGCUGGAGCGUGGUACGUAUACGUGAAGACUGGAAAAAGGAGUGGUGCUCUAUGGGAUG
KU365780    GCGCUAGAUGAGAGUGGUGAUUUCUCCCUGGUGGAGGAUGACGGUCCCCCCAUGAGAGAGAUCAUACUCAAGGUGGUCCUGAUGACCAUCUGUGGCAUGAACCCAAUAGCCAUACCCUUUGCAGCUGGAGCGUGGUACGUAUACGUGAAGACUGGAAAAAGGAGUGGUGCUCUAUGGGAUG
KU365779    GCGCUAGAUGAGAGUGGUGAUUUCUCCCUGGUGGAGGAUGACGGUCCCCCCAUGAGAGAGAUCAUACUCAAGGUGGUCCUGAUGACCAUCUGUGGCAUGAACCCAAUAGCCAUACCCUUUGCAGCUGGAGCGUGGUACGUAUACGUGAAGACUGGAAAAAGGAGUGGUGCUCUAUGGGAUG
KU707826    GCGCUAGAUGAGAGUGGUGAUUUCUCCCUGGUGGAGGAUGACGGUCCCCCCAUGAGAGAGAUCAUACUCAAGGUGGUCCUGAUGACCAUCUGUGGCAUGAACCCAAUAGCCAUACCCUUUGCAGCUGGAGCGUGGUACGUAUACGUGAAGACUGGAAAAAGGAGUGGUGCUCUAUGGGAUG
KU365778    GCGCUAGAUGAGAGUGGUGAUUUCUCCCUGGUGGAGGAUGACGGUCCCCCCAUGAGAGAGAUCAUACUCAAGGUGGUCCUGAUGACCAUCUGUGGCAUGAAUCCAAUAGCCAUACCCUUUGCAGCUGGAGCGUGGUACGUAUACGUGAAGACUGGAAAAAGGAGUGGUGCUCUAUGGGAUG
KU365777    GCGCUAGAUGAGAGUGGUGAUUUCUCCCUGGUGGAGGAUGACGGUCCCCCCAUGAGAGAGAUCAUACUCAAGGUGGUCCUGAUGACCAUCUGUGGCAUGAACCCAAUAGCCAUACCCUUUGCAGCUGGAGCGUGGUACGUAUACGUGAAGACUGGAAAAAGGAGUGGUGCUCUAUGGGAUG
KU312312    GCGCUAGAUGAGAGUGGUGAUUUCUCCCUGGUGGAGGAUGACGGUCCCCCCAUGAGAGAGAUCAUACUCAAGGUGGUCCUGAUGACCAUCUGUGGCAUGAACCCAAUAGCCAUACCCUUUGCAGCUGGAGCGUGGUACGUAUACGUGAAGACUGGAAAAAGGAGUGGUGCUCUAUGGGAUG
KF268950    GCACUAGAUGAGAGUGGUGAUUUCUCUCUGGUGGAGGAAGAUGGUCCACCCAUGAGAGAGAUCAUACUCAAGGUGGUCCUGAUGGCCAUCUGUGGCAUGAACCCAAUAGCCAUACCCUUUGCUGCAGGAGCGUGGUAUGUGUAUGUGAAGACUGGAAAAAGGAGUGGUGCCCUCUGGGAUG
KF268949    GCACUAGAUGAGAGUGGUGAUUUCUCUCUGGUGGAGGAAGAUGGUCCACCCAUGAGAGAGAUCAUACUCAAGGUGGUCCUGAUGGCCAUCUGUGGCAUGAACCCAAUAGCCAUACCCUUUGCUGCAGGAGCGUGGUAUGUGUAUGUGAAGACUGGGAAAAGGAGUGGUGCCCUCUGGGAUG
KF268948    GCACUAGAUGAGAGUGGUGAUUUCUCUCUGGUGGAGGAAGAUGGUCCACCCAUGAGAGAGAUCAUACUCAAGGUGGUCCUGAUGGCCAUCUGUGGCAUGAACCCAAUAGCCAUACCCUUUGCUGCAGGAGCGUGGUAUGUGUAUGUGAAGACUGGAAAAAGGAGUGGUGCCCUCUGGGAUG
KJ776791    GCGCUAGAUGAGAGUGGUGAUUUCUCCCUGGUGGAGGAUGACGGUCCCCCCAUGAGAGAGAUCAUACUCAAGGUGGUCCUGAUGACCAUCUGUGGCAUGAACCCAAUAGCCAUACCCUUUGCAGCUGGAGCGUGGUACGUAUACGUGAAGACUGGAAAAAGGAGUGGUGCUCUAUGGGAUG
KF383119    GCACUGGAUGAGAGUGGUGAUUUCUCCUUGGUAGAGGAAGAUGGUCCACCCAUGAGAGAGAUCAUACUCAAGGUGGUCCUGAUGGCCAUCUGUGGCAUGAACCCAAUAGCUAUACCUUUUGCUGCAGGAGCGUGGUAUGUGUAUGUGAAGACUGGGAAAAGGAGUGGCGCCCUCUGGGACG
KF383118    GCACUGGAUGAGAGUGGUGAUUUCUCCUUGGUAGAGGAAGAUGGUCCACCCAUGAGAGAGAUCAUACUCAAGGUGGUCCUGAUGGCCAUCUGUGGCAUGAACCCAAUAGCUAUACCUUUUGCUGCAGGAGCGUGGUAUGUGUAUGUGAAGACUGGGAAAAGGAGUGGCGCCCUCUGGGACG
KF383117    GCACUGGAUGAGAGUGGUGAUUUCUCCUUGGUAGAGGAGGAUGGCCCACCCAUGAGAGAGAUCAUACUCAAGGUGGUCCUGAUGGCCAUCUGUGGCAUGAACCCAAUAGCCAUACCCUUCGCUGCAGGAGCGUGGUAUGUGUAUGUAAAGACUGGGAAAAAGGAGUGGUGCCCUCUGGGACG
KF383116    GCACUAGAUGAGAGUGGUGAUUUCUCCUUGGUAGAGGAGGAUGGCCCACCCAUGAGAGAGAUCAUACUCAAGGUGGUCCUGAUGGCCAUCUGUGGCAUGAACCCAAUAGCCAUACCCUUCGCUGCAGGAGCGUGGUAUGUGUAUGUAAAGACUGGAAAAAGGAGUGGUGCCCUCUGGGACG
KF383115    GCACUAGAUGAGAGUGGUGAUUUCUCUCUGGUAGAGGAAGAUGGUCCACCCAUGAGAGAGAUCAUACUCAAGGUGGUCCUGAUGGCCAUCUGUGGCAUGAACCCAAUAGCCAUACCUUUUGCUGCAGGAGCGUGGUAUGUGUAUGUGAAGACUGGGAAAAGGAGUGGUGCCCUCUGGGAUG
EU545988    GCACUGGAUGAGAGUGGUGAUUUCUCCCUAGUGGAGGAUGAUGGUCCCCCCAUGAGAGAGAUCAUACUCAAAGUGGUCCUGAUGACCAUCUGUGGCAUGAACCCAAUAGCCAUACCCUUUGCAGCUGGAGCGUGGUACGUGUAUGUGAAGACUGGAAAAAGGAGUGGUGCUCUAUGGGAUG
KU955589    GCGCUAGAUGAGAGUGGUGAUUUCUCCCUGGUGGAGGAUGACGGUCCCCCCAUGAGAGAGAUCAUACUCAAGGUGGUCCUGAUGACCAUCUGUGGCAUGAACCCAAUAGCCAUACCCUUUGCAGCUGGAGCGUGGUACGUAUACGUGAAGACUGGAAAAAGGAGUGGAGCUCUAUGGGAUG
KU955590    GCGCUAGAUGAGAGUGGUGAUUUCUCCCUGGUGGAGGAUGACGGUCCCCCCAUGAGAGAGAUCAUACUCAAGGUGGUCCUGAUGACCAUCUGUGGCAUGAACCCAAUAGCCAUACCCUUUGCAGCUGGAGCGUGGUACGUAUACGUGAAGACUGGAAAAAGGAGUGGUGCUCUAUGGGAUG
KU940228    GCGCUAGAUGAGAGUGGUGAUUUCUCCCUGGUGGAGGAUGACGGUCCCCCCAUGAGAGAGAUCAUACUCAAGGUGGUCCUGAUGACCAUCUGUGGCAUGAACCCAAUAGCCAUACCCUUUGCAGCUGGAGCGUGGUACGUAUACGUGAAGACUGGAAAAAGGAGUGGUGCUCUAUGGGAUG
KU991811    GCGCUAGAUGAGAGUGGUGAUUUCUCCCUGGUGGAGGAUGACGGUCCCCCCAUGAGAGAGAUCAUACUCAAGGUGGUCCUGAUGACCAUCUGUGGCAUGAACCCAAUAGCCAUACCCUUUGCAGCUGGAGCGUGGUACGUAUACGUGAAGACUGGAAAAAGGAGUGGUGCUCUAUGGGAUG
KU509998    GCGCUAGAUGAGAGUGGUGAUUUCUCCCUGGUGGAGGAUGACGGUCCCCCCAUGAGAGAGAUCAUACUCAAGGUGGUCCUGAUGACCAUCUGUGGCAUGAACCCAAUAGCCAUACCCUUUGCAGCUGGAGCGUGGUACGUAUACGUGAAGACUGGAAAAAGGAGUGGUGCUCUAUGGGAUG
KX056898    GCGCUAGAUGAGAGUGGUGAUUUCUCCCUGGUGGAGGAUGACGGUCCCCCCAUGAGAGAGAUCAUACUCAAGGUGGUCCUGAUGACCAUCUGUGGCAUGAACCCAAUAGCCAUACCCUUUGCAGCUGGAGCGUGGUACGUAUACGUGAAGACUGGAAAAAGGAGUGGUGCUCUAUGGGAUG
KX117076    GCGCUAGAUGAGAGUGGUGAUUUCUCCCUGGUGGAGGAUGACGGUCCCCCCAUGAGAGAGAUCAUACUCAAGGUGGUCCUGAUGACCAUCUGUGGCAUGAACCCAAUAGCCAUACCCUUUGCAGCUGGAGCGUGGUACGUAUACGUGAAGACUGGAAAAAGGAGUGGAGCUCUAUGGGAUG
KX185891    GCGCUAGAUGAGAGUGGUGAUUUCUCCCUGGUGGAGGAUGACGGUCCCCCCAUGAGAGAGAUCAUACUCAAGGUGGUCCUGAUGACCAUCUGUGGCAUGAACCCAAUAGCCAUACCCUUUGCAGCUGGAGCGUGGUACGUAUACGUGAAGACUGGAAAAAGGAGUGGAGCUCUAUGGGAUG
```

```
KX247646        GCGCUAGAUGAGAGUGGUGAUUUCUCCCUGGUGGAGGAUGACGGUCCCCCAUGAGAGAGAUCAUACUCAAGGUGGUCCUGAUGACCAUCUGUGGCAUGAACCCAAUAGCCAUACCCUUUGCAGCUGGAGCGUGGUACGUAUACGUGAAGACUGGAAAAAGGAGUGGUGCGCUAUGGGAUG
KX262887        GCGCUAGAUGAGAGUGGUGAUUUCUCCCUGGUGGAGGAUGACGGUCCCCCAUGAGAGAGAUCAUACUCAAGGUGGUCCUGAUGACCAUCUGUGGCAUGAACCCAAUAGCCAUACCCUUCGCAGCUGGAGCGUGGUACGUAUACGUGAAGACUGGAAAAAGGAGUGGUGCUCUAUGGGAUG
KX280026        GCGCUAGAUGAGAGUGGUGAUUUCUCCCUGGUGGAGGAUGACGGUCCCCCAUGAGAGAGAUCAUACUCAAGGUGGUCCUGAUGACCAUCUGUGGCAUGAACCCAAUAGCCAUACCCUUUGCAGCUGGAGCGUGGUACGUAUACGUGAAGACUGGAAAAAGGAGUGGUGCUCUAUGGGAUG
KU758877        GCGCUAGAUGAGAGUGGUGAUUUCUCCCUGGUGGAGGAUGACGGUCCCCCAUGAGAGAGAUCAUACUCAAGGUGGUCCUGAUGACCAUCUGUGGCAUGAACCCAAUAGCCAUACCCUUUGCAGCUGGAGCGUGGUACGUAUACGUGAAGACUGGAAAAAGGAGUGGUGCUCUAUGGGAUG
KU866423        GCGCUAGAUGAGAGUGGUGAUUUCUCCCUGGUGGAGGAUGACGGUCCCCCAUGAGAGAGAUCAUACUCAAGGUGGUCCUGAUGACCAUCUGUGGCAUGAACCCAAUAGCCAUACCCUUUGCAGCUGGAGCGUGGUACGUAUACGUGAAGACUGGAAAAAGGAGUGGAGCUCUAUGGGAUG
KX377336        GCACUAGAUGAGAGUGGUGAUUUCUCCCUGGUGGAGGAUGAUGGUCCCCCAUGAGAGAGAUCAUACUCAAGGUGGUCCUGAUGACCAUCUGUGGCAUGAACCCAAUAGCCAUACCCUUUGCAGCUGGAGCGUGGUAUGUGUAUGUGAAGACUGGAAAGAGGAGUGGUGCUCUAUGGGAUG
KX377337        GCGCUAGAUGAGAGUGGUGAUUUCUCCCUGGUGGAGGAUGACGGUCCCCCAUGAGAGAGAUCAUACUCAAGGUGGUCCUGAUGACCAUCUGUGGCAUGAACCCAAUAGCCAUACCCUUUGCAGCUGGAGCGUGGUACGUAUACGUGAAGACUGGAAAAAGGAGUGGUGCUCUAUGGGAUG
KX369547        GCGCUAGAUGAGAGUGGUGAUUUCUCCCUGGUGGAGGAUGACGGUCCCCCAUGAGAGAGAUCAUACUCAAGGUGGUCCUGAUGACCAUCUGUGGCAUGAACCCAAUAGCCAUACCCUUUGCAGCUGGAGCGUGGUACGUAUACGUGAAGACUGGAAAAAGGAGUGGUGCUCUAUGGGAUG
KX548902        GCGCUAGAUGAGAGUGGUGAUUUCUCCCUGGUGGAGGAUGACGGUCCCCCAUGAGAGAGAUCAUACUCAAGGUGGUCCUGAUGACCAUCUGUGGCAUGAACCCAAUAGCCAUACCCUUUGCAGCUGGAGCGUGGUACGUAUACGUGAAGACUGGAAAAAGGAGUGGUGCUCUAUGGGAUG
KX601166        GCACUAGAUGAGAGUGGUGAUUUCUCCUUGGUAGAGGAGGAUGGCCCACCCAUGAGAGAGAUCAUACUCAAGGUGGUUCUGAUGGCCAUCUGUGGCAUGAACCCAAUAGCCAUACCCUUCGCUGCAGGAGCGUGGUAUGUGUAUGUAAAGACUGGAAAAAGGAGUGGUGCCCUCUGGGACG
KX601167        GCACUAGAUGAGAGUGGUGAUUUCUCCCUGGUGGAGGAUGAUGGUCCCCCAUGAGAGAGAUCAUACUCAAGGUGGUCCUGAUGACCAUCUGUGGCAUGAACCCAAUAGCCAUACCCUUUGCAGCUGGAGCGUGGUAUGUGUAUGUGAAGACUGGAAAGAGGAGUGGUGCUCUAUGGGAUG
KX601168        GCGCUAGAUGAGAGUGGUGAUUUCUCCCUGGUGGAGGAUGACGGUCCCCCAUGAGAGAGAUCAUACUCAAGGUGGUCCUGAUGACCAUCUGUGGCAUGAACCCAAUAGCCAUACCCUUUGCAGCUGGAGCGUGGUACGUAUACGUGAAGACUGGAAAAAGGAGUGGUGCUCUAUGGGAUG
KX766028        GCGCUAGAUGAGAGUGGUGAUUUCUCCCUGGUGGAGGAUGACGGUCCCCCAUGAGAGAGAUCAUACUCAAGGUGGUCCUGAUGACCAUCUGUGGCAUGAACCCAAUAGCCAUACCCUUUGCAGCUGGAGCGUGGUACGUAUACGUGAAGACUGGAAAAAGGAGUGGUGCUCUAUGGGAUG
KX766029        GCGCUAGAUGAGAGUGGUGAUUUCUCCCUGGUGGAGGAUGACGGUCCCCCAUGAGAGAGAUCAUACUCAAGGUGGUCCUGAUGACCAUCUGUGGCAUGAACCCAAUAGCCAUACCCUUUGCAGCUGGAGCGUGGUACGUAUACGUGAAGACUGGGAAAAGGAGUGGUGCUCUAUGGGAUG
KU940224        GCGCUAGAUGAGAGUGGUGAUUUCUCCCUGGUGGAGGAUGACGGUCCCCCAUGAGAGAGAUCAUACUCAAGGUGGUCCUGAUGACCAUCUGUGGCAUGAACCCAAUAGCCAUACCCUUUGCAGCUGGAGCGUGGUACGUAUACGUGAAGACUGGAAAAAGGAGUGGUGCUCUAUGGGAUG


                4444444444444444444444444444444444444444444444444444444444444444444444444444444444444444444444444444444444444444444444444444444444444444444444444444444444444444444444444444444
                5555555555555555555555555555555555555555555555555555555555555555555555555566666666666666666666666666666666666666666666666666666666666666666666666666666666666666666666666666667777777
                2222333333333344444444445555555555666666666677777777778888888888999999999900000000001111111111222222222233333333334444444444555555555566666666667777777777888888888899999999990000000
Sequence        6789012345678901234567890123456789012345678901234567890123456789012345678901234567890123456789012345678901234567890123456789012345678901234567890123456789012345678901234567890123456
-----------------------------------------------------------------------------------------------------------------------------------------------------------------------------------------------------
NC_012532       UGCCUGCUCCCAAAGAAGUGAAGAAAGGAGAGACCACAGAUGGAGUGUACAGAGUGAUGACUCGCAGACUGCUAGGUUCAACACAGGUUGGAGUGGGAGUCAUGCAAGAGGGAGUCUUCCACACCAUGUGGCACGUUACAAAAGGAGCCGCACUGAGGAGCGGUGAGGGAAGACUUGAUCC
KU922960        UGCCUGCUCCCAAGGAAGUAAAAAAGGGGGAGACCACAGAUGGAGUGUACAGAGUAAUGACUCGUAGACUGCUAGGUUCAACACAAGUUGGAGUGGGAGUUAUGCAAGAGGGGGUCUUUCACACUAUGUGGCACGUCACAAAAGGAUCCGCGCUGAGAAGCGGUGAAGGGAGACUUGAUCC
KU922923        UGCCUGCUCCCAAGGAAGUAAAAAAGGGGGAGACCACAGAUGGAGUGUACAGAGUAAUGACUCGUAGACUGCUAGGUUCAACACAAGUUGGAGUGGGAGUUAUGCAAGAGGGGGUCUUUCACACUAUGUGGCACGUCACAAAAGGAUCCGCGCUGAGAAGCGGUGAAGGGAGACUUGAUCC
KU820898        UGCCUGCUCCCAAGGAAGUAAAAAAGGGGGAGACCACAGAUGGAGUGUACAGAGUAAUGACUCGCAGACUGCUAGGUUCAACACAAGUUGGAGUGGGAGUUAUGCAAGAGGGGGUCUUUCACACUAUGUGGCACGUCACAAAAGGAUCCGCGCUGAGAAGCGGUGAAGGGAGACUUGAUCC
KU321639        UGCCUGCUCCCAAGGAAGUAAAAAAGGGGGAGACCACAGAUGGAGUGUACAGAGUAAUGACUCGUAGACUGCUAGGUUCAACACAAGUUGGAGUGGGAGUUAUGCAAGAGGGGGUCUUUCACACUAUGUGGCACGUCACAAAAGGAUCCGCGCUGAGAAGCGGUGAAGGGAGACUUGAUCC
KU820899        UGCCUGCUCCCAAGGAAGUAAAAAAGGGGGAGACCACAGAUGGAGUGUACAGAGUGAUGACUCGUAGACUGCUAGGUUCAACACAAGUUGGAGUGGGAGUUAUGCAAGAGGGGGUCUUUCACACCAUGUGGCACGUCACAAAAGGAUCCGCGCUGAGAAGCGGUGAAGGGAGACUUGAUCC
KU853012        UGCCUGCUCCCAAGGAAGUAAAAAAGGGGGAGACCACAGAUGGAGUGUACAGAGUAAUGACUCGUAGACUGCUAGGUUCAACACAAGUUGGAGUGGGAGUUAUGCAAGAGGGGGUCUUUCACACUAUGUGGCACGUCACAAAAGGAUCCGCGCUGAGAAGCGGUGAAGGGAGACUUGAUCC
KU729217        UGCCUGCUCCCAAGGAAGUAAAAAAGGGGGAGACCACAGAUGGAGUGUACAGAGUAAUGACUCGUAGACUGCUAGGUUCAACACAAGUUGGAGUGGGAGUUAUGCAAGAGGGGGUCUUUCACACUAUGUGGCACGUCACAAAAGGAUCCGCGCUGAGAAGCGGUGAAGGGAGACUUGAUCC
KU729218        UGCCUGCUCCCAAGGAAGUAAAAAAGGGGGAGACCACAGAUGGAGUGUACAGAGUAAUGACUCGUAGACUGCUAGGUUCAACACAAGUUGGAGUGGGAGUUAUGCAAGAGGGGGUCUUUCACACUAUGUGGCACGUCACAAAAGGAUCCGCGCUGAGAAGCGGUGAAGGGAGACUUGAUCC
KU761564        UGCCUGCUCCCAAGGAAGUAAAAAAGGGGGAGACCACAGAUGGAGUGUACAGAGUAAUGACUCGCAGACUGCUAGGUUCAACACAAGUUGGAGUGGGAGUUAUGCAAGAGGGGGUCUUUCACACUAUGUGGCACGUCACAAAAGGAUCCGCGCUGAGAAGCGGUGAAGGGAGACUUGAUCC
KU740184        UGCCUGCUCCCAAGGAAGUAAAAAAGGGGGAGACCACAGAUGGAGUGUACAGAGUAAUGACUCGCAGACUGCUAGGUUCAACACAAGUUGGAGUGGGAGUUAUGCAAGAGGGGGUCUUUCACACUAUGUGGCACGUCACAAAAGGAUCCGCGCUGAGAAGCGGUGAAGGGAGACUUGAUCC
KU744693        UGCCUGCUCCCAAGGAAGUAAAAAAGGGGGAGACCACAGAUGGAGUGUACAGAGUAAUGACUCGCAGACUGCUAGGUUCAACACAAGUUGGAGUGGGAGUUAUGCAAGAGGGGGUCUUUCACACUAUGUGGCACGUCACAAAAGGAUCCGCGCUGAGAAGCGGUGAAGGGAGACUUGAUCC
KU497555        UGCCUGCUCCCAAGGAAGUAAAAAAGGGGGAGACCACAGAUGGAGUGUACAGAGUAAUGACUCGUAGACUGCUAGGUUCAACACAAGUUGGAGUGGGAGUUAUGCAAGAGGGGGUCUUUCACACUAUGUGGCACGUCACAAAAGGAUCCGCGCUGAGAAGCGGUGAAGGGAGACUUGAUCC
KU527068        UGCCUGCUCCCAAGGAAGUAAAAAAGGGGGAGACCACAGAUGGAGUGUACAGAGUAAUGACUCGUAGACUGCUAGGUUCAACACAAGUUGGAGUGGGAGUUAUGCAAGAGGGGGUCUUUCACACUAUGUGGCACGUCACAAAAGGAUCCGCGCUGAGAAGCGGUGAAGGGAGACUUGAUCC
KU501217        UGCCUGCUCCCAAGGAAGUAAAAAAGGGGGAGACCACAGAUGGAGUGUACAGAGUAAUGACUCGUAGACUGCUAGGUUCAACACAAGUUGGAGUGGGAGUUAUGCAAGAGGGGGUCUUUCACACUAUGUGGCACGUCACAAAAGGAUCCGCACUGAGAAGCGGUGAAGGGAGACUUGAUCC
KU501216        UGCCUGCUCCCAAGGAAGUAAAAAAGGGGGAGACCACAGAUGGAGUGUACAGAGUAAUGACUCGUAGACUGCUAGGUUCAACACAAGUUGGAGUGGGAGUUAUGCAAGAGGGGGUCUUUCACACUAUGUGGCACGUCACAAAAGGAUCCGCACUGAGAAGCGGUGAAGGGAGACUUGAUCC
KU501215        UGCCUGCUCCCAAGGAAGUAAAAAAGGGGGAGACCACAGAUGGAGUGUACAGAGUAAUGACUCGUAGACUGCUAGGUUCAACACAAGUUGGAGUGGGAGUUAUGCAAGAGGGGGUCUUUCACACUAUGUGGCACGUCACAAAAGGAUCCGCGCUGAGAAGCGGUGAAGGGAGACUUGAUCC
KU365780        UGCCUGCUCCCAAGGAAGUAAAAAAGGGGGAGACCACAGAUGGAGUGUACAGAGUAAUGACUCGUAGACUGCUAGGUUCAACACAAGUUGGAGUGGGAGUUAUGCAAGAGGGGGUCUUUCACACUAUGUGGCACGUCACAAAAGGAUCCGCGCUGAGAAGCGGUGAAGGGAGACUUGAUCC
KU365779        UGCCUGCUCCCAAGGAAGUAAAAAAGGGGGAGACCACAGAUGGAGUGUACAGAGUAAUGACUCGUAGACUGCUAGGUUCAACACAAGUUGGAGUGGGAGUUAUGCAAGAGGGGGUCUUUCACACUAUGUGGCACGUCACAAAAGGAUCCGCGCUGAGAAGCGGUGAAGGGAGACUUGAUCC
KU707826        UGCCUGCUCCCAAGGAAGUAAAAAAGGGGGAGACCACAGAUGGAGUGUACAGAGUAAUGACUCGUAGACUGCUAGGUUCAACACAAGUUGGAGUGGGAGUUAUGCAAGAGGGGGUCUUUCACACUAUGUGGCACGUCACAAAAGGAUCCGCGCUGAGAAGCGGUGAAGGGAGACUUGAUCC
KU365778        UGCCUGCUCCCAAGGAAGUAAAAAAGGGGGAGACCACAGAUGGAGUGUACAGAGUAAUGACUCGUAGACUGCUAGGUUCAACACAAGUUGGAGUGGGAGUUAUGCAAGAGGGGGUCUUUCACACUAUGUGGCACGUCACAAAAGGAUCCGCGCUGAGAAGCGGUGAAGGGAGACUUGAUCC
KU365777        UGCCUGCUCCCAAGGAAGUAAAAAAGGGGGAGACCACAGAUGGAGUGUACAGAGUAAUGACUCGUAGACUGCUAGGUUCAACACAAGUUGGAGUGGGAGUUAUGCAAGAGGGGGUCUUUCACACUAUGUGGCACGUCACAAAAGGAUCCGCGCUGAGAAGCGGUGAAGGGAGACUUGAUCC
KU312312        UGCCUGCUCCCAAGGAAGUAAAAAAGGGGGAGACCACAGAUGGAGUGUACAGAGUAAUGACUCGUAGACUGCUAGGUUCAACACAAGUUGGAGUGGGAGUUAUGCAAGAGGGGGUCUUUCACACUAUGUGGCACGUCACAAAAGGAUCCGCGCUGAGAAGCGGUGAAGGGAGACUUGAUCC
KF268950        UGCCCGCUCCCAAAGAAGUGAAAAAAGGAGAGACCACAGAUGGGGUAUACAGAGUGAUGACCCGCAGACUGCUGGGUUCAACACAGGUUGGAGUGGGAGUCAUGCAAGAGGGAGUCUUCCACACCAUGUGGCACGUCACAAAAGGAGCUGCACUGAGGAGCGGUGAAGGGAGACUUGAUCC
KF268949        UGCCCGCUCCCAAAGAAGUGAAAAAAGGAGAGACCACAGAUGGAGUAUACAGAGUGAUGACCCGCAGACUGCUGGGUUCAACACAGGUUGGAGUUGGAGUCAUGCAAGAGGGAGUCUUCCACACCAUGUGGCACGUCACAAAAGGAGCUGCACUGAGGAGCGGCGAAGGGAGACUUGAUCC
KF268948        UGCCCGCUCCCAAAGAAGUGAAAAAAGGAGAGACCACAGAUGGGGUAUACAGAGUGAUGACCCGCAGACUGCUGGGUUCAACACAGGUUGGAGUGGGAGUCAUGCAAGAGGGAGUCUUCCACACCAUGUGGCACGUCACAAAAGGAGCUGCACUGAGGAGCGGUGAAGGGAGACUUGAUCC
KJ776791        UGCCUGCUCCCAAGGAAGUAAAAAAGGGGGAGACCACAGAUGGAGUGUACAGAGUAAUGACUCGUAGACUGCUAGGUUCAACACAAGUUGGAGUGGGAGUUAUGCAAGAGGGGGUCUUUCACACUAUGUGGCACGUCACAAAAGGAUCCGCGCUGAGAAGCGGUGAAGGGAGACUUGAUCC
KF383119        UGCCUGCUCCCAAAGAAGUGAAGAAAGGAGAGACCACAGAUGGAGUGUACAGAGUGAUGACUCGCAGACUGCUAGGUUCAACACAGGUUGGAGUGGGAGUCAUGCAAGAGGGAGUCUUCCACACCAUGUGGCACGUUACAAAAGGAGCCGCACUGAGGAGCGGUGAGGGAAGACUUGAUCC
KF383118        UGCCUGCUCCCAAAGAAGUGAAGAAAGGAGAGACCACAGAUGGAGUGUACAGAGUGAUGACUCGCAGACUGCUAGGUUCAACACAGGUUGGAGUGGGAGUCAUGCAAGAGGGAGUCUUCCACACCAUGUGGCACGUUACAAAAGGAGCCGCACUGAGGAGCGGUGAGGGAAGACUUGAUCC
KF383117        UGCCUGCUCCCAAAGAAGUGAAAAAAGGAGAGACUACAGAUGGAGUGUACAGAGUGAUGACUCGCAGACUGCUGGGUUCAACACAGGUUGGAGUGGGGGUCAUGCAAGAGGGAGUCUUCCACACCAUGUGGCACGUCACAAAAGGAGCUGCAUUGAGGAGCGGUGAAGGAAGACUUGAUCC
KF383116        UGCCUGCUCCCAAAGAAGUGAAAAAAGGAGAGACUACAGAUGGAGUGUACAGAGUGAUGACUCGCAGACUGCUGGGUUCAACACAGGUUGGAGUGGGAGUCAUGCAAGAGGGAGUCUUCCACACCAUGUGGCACGUCACAAAAGGAGCCGCAUUGAGGAGCGGUGAAGGAAGACUUGAUCC
KF383115        UGCCAGCUCCUAAAGAAGUGAAAAAAGGAGAGACCACAGAUGGAGUAUACAGAGUGAUGACCCGCAGAUUGCUGGGUUCAACACAGGUUGGAGUGGGAGUCAUGCAGGAGGGAGUCUUCCACACCAUGUGGCACGUCACAAAAGGAGCCGCACUGAGGAGCGGUGAAGGGAGACUUGAUCC
EU545988        UGCCUGCUCCCAAGGAAGUAAAAAAGGGGGAGACCACAGAUGGAGUGUACAGAGUAAUGACUCGUAGACUGCUUGGUUCAACACAAGUUGGAGUGGGAGUCAUGCAAGAGGGGGUCUUCCACACUAUGUGGCACGUCACAAAAGGAUCCGCGCUGAGAAGCGGUGAAGGGAGACUUGAUCC
KU955589        UGCCUGCUCCCAAGGAAGUAAAAAAGGGGGAGACCACAGAUGGAGUGUACAGAGUGAUGACUCGUAGACUGCUAGGUUCAACACAAGUUGGAGUGGGAGUUAUGCAAGAGGGGGUCUUUCACACCAUGUGGCACGUCACAAAAGGAUCCGCGCUGAGAAGCGGUGAAGGGAGACUUGAUCC
KU955590        UGCCUGCUCCCAAGGAAGUAAAAAAGGGGGAGACCACAGAUGGAGUGUACAGAGUAAUGACUCGUAGACUGCUAGGUUCAACACAAGUUGGAGUGGGAGUUAUGCAAGAGGGGGUCUUUCACACUAUGUGGCACGUCACAAAAGGAUCCGCGCUGAGAAGCGGUGAAGGGAGACUUGAUCC
KU940228        UGCCUGCUCCCAAGGAAGUAAAAAAGGGGGAGACCACAGAUGGAGUGUACAGAGUAAUGACUCGUAGACUGCUAGGUUCAACACAAGUUGGAGUGGGAGUUAUGCAAGAGGGGGUCUUUCACACUAUGUGGCACGUCACAAAAGGAUCCGCGCUGAGAAGCGGUGAAGGGAGACUUGAUCC
KU991811        UGCCUGCUCCCAAGGAAGUAAAAAAGGGGGAGACCACAGAUGGAGUGUACAGAGUAAUGACUCGCAGACUGCUAGGUUCAACACAAGUUGGAGUGGGAGUUAUGCAAGAGGGGGUCUUUCACACUAUGUGGCACGUCACAAAAGGAUCCGCGCUGAGAAGCGGUGAAGGGAGACUUGAUCC
KU509998        UGCCUGCUCCCAAGGAAGUAAAAAAGGGGGAGACCACAGAUGGAGUGUACAGAGUAAUGACUCGUAGACUGCUAGGUUCAACACAAGUUGGAGUGGGAGUUAUGCAAGAGGGGGUCUUUCACACUAUGUGGCACGUCACAAAAGGAUCCGCGCUGAGAAGCGGUGAAGGGAGACUUGAUCC
KX056898        UGCCUGCUCCCAAGGAAGUAAAAAAGGGGGAGACCACAGAUGGAGUGUACAGAGUAAUGACUCGUAGACUGCUAGGUUCAACACAAGUUGGAGUGGGAGUUAUGCAAGAGGGGGUCUUUCACACUAUGUGGCACGUCACAAAAGGAUCCGCGCUGAGAAGCGGUGAAGGGAGACUUGAUCC
KX117076        UGCCUGCUCCCAAGGAAGUAAAAAAGGGGGAGACCACAGAUGGAGUGUACAGAGUAAUGACUCGUAGACUGCUAGGUUCAACACAAGUUGGAGUGGGAGUUAUGCAAGAGGGGGUCUUUCACACCAUGUGGCACGUCACAAAAGGAUCCGCGCUGAGAAGCGGUGAAGGGAGACUUGAUCC
KX185891        UGCCUGCUCCCAAGGAAGUAAAAAAGGGGGAGACCACAGAUGGAGUGUACAGAGUGAUGACUCGUAGACUGCUAGGUUCAACACAAGUUGGAGUGGGAGUUAUGCAAGAGGGGGUCUUUCACACCAUGUGGCACGUCACAAAAGGAUCCGCGCUGAGAAGCGGUGAAGGGAGACUUGAUCC
KX247646        UGCCUGCUCCCAAGGAAGUAAAAAAGGGGGAGACCACAGAUGGAGUGUACAGAGUAAUGACUCGUAGACUGCUAGGUUCAACACAAGUUGGAGUGGGAGUUAUGCAAGAGGGGGUCUUUCACACUAUGUGGCACGUCACAAAAGGAUCCGCGCUGAGAAGCGGUGAAGGGAGACUUGAUCC
KX262887        UGCCUGCUCCCAAGGAAGUAAAAAAGGGGGAGACCACAGAUGGAGUGUACAGAGUAAUGACUCGUAGACUGCUAGGUUCAACACAAGUUGGAGUGGGAGUUAUGCAAGAGGGGGUCUUUCACACUAUGUGGCACGUCACAAAAGGAUCCGCACUGAGAAGCGGUGAAGGGAGACUUGAUCC
KX280026        UGCCUGCUCCCAAGGAAGUAAAAAAGGGGGAGACCACAGAUGGAGUGUACAGAGUAAUGACUCGUAGACUGCUAGGUUCAACACAAGUUGGAGUGGGAGUUAUGCAAGAGGGGGUCUUUCACACUAUGUGGCACGUCACAAAAGGAUCCGCGCUGAGAAGCGGUGAAGGGAGACUUGAUCC
KU758877        UGCCUGCUCCCAAGGAAGUAAAAAAGGGGGAGACCACAGAUGGAGUGUACAGAGUAAUGACUCGUAGACUGCUAGGUUCAACACAAGUUGGAGUGGGAGUUAUGCAAGAGGGGGUCUUUCACACUAUGUGGCACGUCACAAAAGGAUCCGCGCUGAGAAGCGGUGAAGGGAGACUUGAUCC
KU866423        UGCCUGCUCCCAAGGAAGUAAAAAAGGGGGAGACCACAGAUGGAGUGUACAGAGUGAUGACUCGUAGACUGCUAGGUUCAACACAAGUUGGAGUGGGAGUUAUGCAAGAGGGGGUCUUUCACACCAUGUGGCACGUCACAAAAGGAUCCGCGCUGAGAAGCGGUGAAGGGAGACUUGAUCC
KX377336        UGCCUGCUCCCAAGGAAGUAAAAAAGGGGGAGACCACAGAUGGAGUGUAUAGAGUGAUGACUCGCAGACUGCUAGGUUCAACACAAGUUGGAGUGGGAGUCAUGCAAGAGGGGGUCUUCCACACUAUGUGGCACGUCACAAAAGGAUCCGCGCUGAGGAGCGGUGAAGGGAGACUUGAUCC
KX377337        UGCCUGCUCCCAAGGAAGUAAAAAAGGGGGAGACCACAGAUGGAGUGUACAGAGUAAUGACUCGUAGACUGCUAGGUUCAACACAAGUUGGAGUGGGAGUUAUGCAAGAGGGGGUCUUUCACACUAUGUGGCACGUCACAAAAGGAUCCGCGCUGAGAAGCGGUGAAGGGAGACUUGAUCC
KX369547        UGCCUGCUCCCAAGGAAGUAAAAAAGGGGGAGACCACAGAUGGAGUGUACAGAGUAAUGACUCGUAGACUGCUAGGUUCAACACAAGUUGGAGUGGGAGUUAUGCAAGAGGGGGUCUUUCACACUAUGUGGCACGUCACAAAAGGAUCCGCGCUGAGAAGCGGUGAAGGGAGACUUGAUCC
KX548902        UGCCUGCUCCCAAGGAAGUAAAAAAGGGGGAGACCACAGAUGGAGUGUACAGAGUAAUGACUCGUAGACUGCUAGGUUCAACACAAGUUGGAGUGGGAGUUAUGCAAGAGGGGGUCUUUCACACUAUGUGGCACGUCACAAAAGGAUCCGCGCUGAGAAGCGGUGAAGGGAGACUUGAUCC
KX601166        UGCCUGCUCCCAAAGAAGUGAAAAAAGGAGAGACUACAGAUGGAGUGUACAGAGUGAUGACUCGCAGACUGCUGGGUUCAACACAGGUUGGAGUGGGAGUCAUGCAAGAGGGAGUCUUCCACACCAUGUGGCACGUCACAAAAGGAGCCGCAUUGAGGAGCGGUGAAGGAAGACUUGAUCC
KX601167        UGCCUGCUCCCAAGGAAGUAAAAAAGGGGGAGACCACAGAUGGAGUGUAUAGAGUGAUGACUCGCAGACUGCUAGGUUCAACACAAGUUGGAGUGGGAGUCAUGCAAGAGGGGGUCUUCCACACUAUGUGGCACGUCACAAAAGGAUCCGCGCUGAGGAGCGGUGAAGGGAGACUUGAUCC
KX601168        UGCCUGCUCCCAAGGAAGUAAAAAAGGGGGAGACCACAGAUGGAGUGUACAGAGUAAUGACUCGUAGACUGCUAGGUUCAACACAAGUUGGAGUGGGAGUUAUGCAAGAGGGGGUCUUUCACACUAUGUGGCACGUCACAAAAGGAUCCGCGCUGAGAAGCGGUGAAGGGAGACUUGAUCC
KX766028        UGCCUGCUCCCAAGGAAGUAAAAAAGGGGGAGACCACAGAUGGAGUGUACAGAGUAAUGACUCGCAGACUGCUAGGUUCAACACAAGUUGGAGUGGGAGUUAUGCAAGAGGGGGUCUUUCACACUAUGUGGCACGUCACAAAAGGAUCCGCGCUGAGAAGCGGUGAAGGGAGACUUGAUCC
KX766029        UGCCUGCUCCCAAGGAAGUAAAAAAGGGGGAGACCACAGAUGGAGUGUACAGAGUAAUGACUCGUAGACUGCUAGGUUCAACACAAGUUGGAGUGGGAAUUAUGCAAGAGGGGGUCUUUCACACUAUGUGGCACGUCACAAAAGGAUCCGCACUGAGAAGCGGUGAAGGGAGACUUGAUCC
KU940224        UGCCUGCUCCCAAGGAAGUAAAAAAGGGGGAGACCACAGAUGGAGUGUACAGAGUAAUGACUCGUAGACUGCUAGGUUCAACACAAGUUGGAGUGGGAGUUAUGCAAGAGGGGGUCUUUCACACUAUGUGGCACGUCACAAAAGGAUCCGCGCUGAGAAGCGGUGAAGGGAGACUUGAUCC
```

```
                4444444444444444444444444444444444444444444444444444444444444444444444444444444444444444444444444444444444444444444444444444444444444444444444444444444444444444444444444444444
                7777777777777777777777777777777777777777777777777777777777777777777777777777777777777777777778888888888888888888888888888888888888888888888888888888888888888888888888888888888
                0001111111111222222222233333333334444444444555555555566666666667777777777888888888899999999990000000000111111111122222222223333333333444444444455555555556666666666777777777788888888
Sequence        7890123456789012345678901234567890123456789012345678901234567890123456789012345678901234567890123456789012345678901234567890123456789012345678901234567890123456789012345678901234567
-----------------------------------------------------------------------------------------------------------------------------------------------------------------------------------------------------
NC_012532       AUACUGGGGGGAUGUCAAGCAGGACUUGGUGUCAUACUGUGGGCCUUGGAAGUUGGAUGCAGCUUGGGAUGGACUCAGCGAGGUACAGCUUUUGGCCGUACCUCCCGGAGAGAGGGCCAGAAACAUUCAGACCCUGCCUGGAAUAUUCAAGACAAAGGACGGGGACAUCGGAGCAGUUGCU
KU922960        AUACUGGGGAGAUGUCAAGCAGGAUCUGGUGUCAUACUGUGGUCCAUGGAAGCUAGAUGCCGCCUGGGACGGGCACAGCGAGGUGCAGCUCUUGGCCGUGCCCCCGGAGAGAGAGAGAGGAACAUCCAGACUCUGCCCGGAAUAUUUAAGACAAAGGAUGGGGACAUUGGAGCGGUUGCG
KU922923        AUACUGGGGAGAUGUCAAGCAGGAUCUGGUGUCAUACUGUGGUCCAUGGAAGCUAGAUGCCGCCUGGGACGGGCACAGCGAGGUGCAGCUCUUGGCCGUGCCCCCGGAGAGAGAGAGAGGAACAUCCAGACUCUGCCCGGAAUAUUUAAGACAAAGGAUGGGGACAUUGGAGCGGUUGCG
KU820898        AUACUGGGGAGAUGUCAAGCAGGAUCUGGUGUCAUACUGUGGUCCAUGGAAGCUAGAUGCCGCCUGGGACGGGCACAGCGAGGUGCAGCUCUUGGCCGUGCCCCCGGAGAGAGAGCGAGGAACAUCCAGACUCUGCCCGGAAUAUUUAAGACAAAGGAUGGGGACAUUGGAGCGGUUGCG
KU321639        AUACUGGGGAGAUGUCAAGCAGGAUCUGGUGUCAUACUGUGGUCCAUGGAAGCUAGAUGCCGCCUGGGACGGGCACAGCGAGGUGCAGCUCUUGGCCGUGCCCCCGGAGAGAGAGCGAGGAACAUCCAGACUCUGCCCGGAAUAUUUAAGACAAAGGAUGGGGACAUUGGAGCGGUUGCG
KU820899        AUACUGGGGAGAUGUCAAGCAGGAUCUGGUGUCAUACUGUGGUCCAUGGAAGCUAGAUGCCGCCUGGGACGGGCACAGCGAGGUGCAGCUCUUGGCCGUGCCCCCGGAGAGAGAGCGAGGAACAUCCAGACUCUGCCCGGAAUAUUUAAGACAAAGGAUGGGGACAUUGGAGCGGUUGCG
KU853012        AUACUGGGGAGAUGUCAAGCAGGAUCUGGUGUCAUACUGUGGUCCAUGGAAGCUAGAUGCCGCCUGGGACGGGCACAGCGAGGUGCAGCUCUUGGCCGUGCCCCCGGAGAGAGAGCGAGGAACAUCCAGACUCUGCCCGGAAUAUUUAAGACAAAGGAUGGGGACAUUGGAGCGGUUGCG
KU729217        AUACUGGGGAGAUGUCAAGCAGGAUCUGGUGUCAUACUGUGGUCCAUGGAAGCUAGAUGCCGCCUGGGACGGGCACAGCGAGGUGCAGCUCUUGGCCGUGCCCCCGGAGAGAGAGCGAGGAACAUCCAGACUCUGCCCGGAAUAUUUAAGACAAAGGAUGGGGACAUUGGAGCGGUUGCG
KU729218        AUACUGGGGAGAUGUCAAGCAGGAUCUGGUGUCAUACUGUGGUCCAUGGAAGCUAGAUGCCGCCUGGGACGGGCACAGCGAGGUGCAGCUCUUGGCCGUGCCCCCGGAGAGAGAGCGAGGAACAUCCAGACUCUGCCCGGAAUAUUUAAGACAAAGGAUGGGGACAUUGGAGCGGUUGCG
KU761564        AUACUGGGGAGAUGUCAAGCAGGAUCUGGUGUCAUACUGUGGUCCAUGGAAGCUAGAUGCCGCCUGGGACGGGCACAGCGAGGUGCAGCUCUUGGCCGUGCCCCCGGAGAGAGAGCGAGGAACAUCCAGACUCUGCCCGGAAUAUUUAAGACAAAGGAUGGGGACAUUGGAGCGGUUGCG
KU740184        AUACUGGGGAGAUGUCAAGCAGGAUCUGGUGUCAUACUGUGGUCCAUGGAAGCUAGAUGCCGCCUGGGACGGGCACAGCGAGGUGCAGCUCUUGGCCGUGCCCCCGGAGAGAGAGCGAGGAACAUCCAGACUCUGCCCGGAAUAUUUAAGACAAAGGAUGGGGACAUUGGAGCGGUUGCG
KU744693        AUACUGGGGAGAUGUCAAGCAGGAUCUGGUGUCAUACUGUGGUCCAUGGAAGCUAGAUGCCGCCUGGGACGGGCACAGCGAGGUGCAGCUCUUGGCCGUGCCCCCGGAGAGAGAGCGAGGAACAUCCAGACUCUGCCCGGAAUAUUUAAGACAAAGGAUGGGGACAUUGGAGCGGUUGCA
KU497555        AUACUGGGGAGAUGUCAAGCAGGAUCUGGUGUCAUACUGUGGUCCAUGGAAGCUAGAUGCCGCCUGGGACGGGCACAGCGAGGUGCAGCUCUUGGCCGUGCCCCCGGAGAGAGAGCGAGGAACAUCCAGACUCUGCCCGGAAUAUUUAAGACAAAGGAUGGGGACAUUGGAGCGGUUGCG
KU527068        AUACUGGGGAGAUGUCAAGCAGGAUCUGGUGUCAUACUGUGGUCCAUGGAAGCUAGAUGCCGCCUGGGACGGGCACAGCGAGGUGCAGCUCUUGGCCGUGCCCCCGGAGAGAGAGCGAGGAACAUCCAGACUCUGCCCGGAAUAUUUAAGACAAAGGAUGGGGACAUUGGAGCGGUUGCG
KU501217        AUACUGGGGAGAUGUCAAGCAGGAUCUGGUGUCAUACUGUGGUCCAUGGAAGCUAGAUGCCGCCUGGGACGGGCACAGCGAGGUGCAGCUCUUGGCCGUGCCCCCGGAGAGAGAGCGAGGAACAUCCAGACUCUGCCCGGAAUAUUUAAGACAAAGGAUGGGGACAUUGGAGCGGUUGCG
KU501216        AUACUGGGGAGAUGUCAAGCAGGAUCUGGUGUCAUACUGUGGUCCAUGGAAGCUAGAUGCCGCCUGGGACGGGCACAGCGAGGUGCAGCUCUUGGCCGUGCCCCCGGAGAGAGAGCGAGGAACAUCCAGACUCUGCCCGGAAUAUUUAAGACAAAGGAUGGGGACAUUGGAGCGGUUGCG
KU501215        AUACUGGGGAGAUGUCAAGCAGGAUCUGGUGUCAUACUGUGGUCCAUGGAAGCUAGAUGCCGCCUGGGAUGGGCACAGCGAGGUGCAGCUCUUGGCCGUGCCCCCGGAGAGAGAGCGAGGAACAUCCAGACUCUGCCCGGAAUAUUUAAGACAAAGGAUGGGGACAUUGGAGCGGUUGCG
KU365780        AUACUGGGGAGAUGUCAAGCAGGAUCUGGUGUCAUACUGUGGUCCAUGGAAGCUAGAUGCCGCCUGGGACGGGCACAGCGAGGUGCAGCUCUUGGCCGUGCCCCCGGAGAGAGAGCGAGGAACAUCCAGACUCUGCCCGGAAUAUUUAAGACAAAGGAUGGGGACAUUGGAGCGGUUGCG
KU365779        AUACUGGGGAGAUGUCAAGCAGGAUCUGGUGUCAUACUGUGGUCCAUGGAAGCUAGAUGCCGCCUGGGACGGGCACAGCGAGGUGCAGCUCUUGGCCGUGCCCCCGGAGAGAGAGCGAGGAACAUCCAGACUCUGCCCGGAAUAUUUAAGACAAAGGAUGGGGACAUUGGAGCGGUUGCG
KU707826        AUACUGGGGAGAUGUCAAGCAGGAUCUGGUGUCAUACUGUGGUCCAUGGAAGCUAGAUGCCGCCUGGGACGGGCACAGCGAGGUGCAGCUCUUGGCCGUGCCCCCGGAGAGAGAGCGAGGAACAUCCAGACUCUGCCCGGAAUAUUUAAGACAAAGGAUGGGGACAUUGGAGCGGUUGCG
KU365778        AUACUGGGGAGAUGUCAAGCAGGAUCUGGUGUCAUACUGUGGUCCAUGGAAGCUAGAUGCCGCCUGGGACGGGCACAGCGAGGUGCAGCUCUUGGCCGUGCCCCCGGAGAGAGAGCGAGGAACAUCCAGACUCUGCCCGGAAUAUUUAAGACAAAGGAUGGGGACAUUGGAGCGGUUGCG
KU365777        AUACUGGGGAGAUGUCAAGCAGGAUCUGGUGUCAUACUGUGGUCCAUGGAAGCUAGAUGCCGCCUGGGACGGGCACAGCGAGGUGCAGCUCUUGGCCGUGCCCCCGGAGAGAGAGCGAGGAACAUCCAGACUCUGCCCGGAAUAUUUAAGACAAAGGAUGGGGACAUUGGAGCGGUUGCG
KU312312        AUACUGGGGAGAUGUCAAGCAGGAUCUGGUGUCAUACUGUGGUCCAUGGAAGCUAGAUGCCGCCUGGGACGGGCACAGCGAGGUGCAGCUCUUGGCCGUGCCCCCGGAGAGAGAGCGAGGAACAUCCAGACUCUGCCCGGAAUAUUUAAGACAAAGGAUGGGGACAUUGGAGCGGUUGCG
KF268950        AUACUGGGGGGAUGUCAAGCAGGACUUAGUGUCAUACUGUGGGCCUUGGAAGUUGGAUGCAGCUUGGGAUGGACUUAGUGAGGUGCAGCUCUUGGCAGUACCCCCCGGAGAGAGGGCCAGAAACAUUCAGACUCUGCCUGGAAUAUUCAAGACAAAGGAUGGGGACAUCGGAGCAGUCGCU
KF268949        AUACUGGGGGGAUGUCAAGCAGGACUUAGUGUCAUACUGUGGGCCUUGGAAGUUGGAUGCAGCUUGGGAUGGACUUAGUGAGGUGCAGCUCUUGGCAGUACCCCCCGGAGAGAGGGCCAGAAACAUUCAGACUCUGCCUGGAAUAUUCAAGACAAAGGAUGGGGACAUCGGAGCAGUUGCU
KF268948        AUACUGGGGGGAUGUCAAGCAGGACUUAGUGUCAUACUGUGGGCCUUGGAAGUUGGAUGCAGCUUGGGAUGGACUUAGUGAGGUGCAGCUCUUGGCAGUACCCCCCGGAGAGAGGGCCAGAAACAUUCAGACUCUGCCUGGAAUAUUCAAGACAAAGGAUGGGGACAUCGGAGCAGUCGCU
KJ776791        AUACUGGGGAGAUGUCAAGCAGGAUCUGGUGUCAUACUGUGGUCCAUGGAAGCUAGAUGCCGCCUGGGACGGGCACAGCGAGGUGCAGCUCUUGGCCGUGCCCCCGGAGAGAGAGCGAGGAACAUCCAGACUCUGCCCGGAAUAUUUAAGACAAAGGAUGGGGACAUUGGAGCGGUUGCG
KF383119        AUACUGGGGGGAUGUCAAGCAGGACUUGGUGUCAUACUGUGGGCCUUGGAAGUUGGAUGCAGCUUGGGAUGGACUUAGCGAGGUACAGCUUUUGGCCGUACCUCCCGGAGAGAGGGCCAGAAACAUUCAGACCCUGCCUGGAAUAUUCAAGACAAAGGACGGGGACAUCGGAGCAGUUGCU
KF383118        AUACUGGGGGGAUGUCAAGCAGGACUUGGUGUCAUACUGUGGGCCUUGGAAGUUGGAUGCAGCUUGGGAUGGACUUAGCGAGGUACAGCUUUUGGCCGUACCUCCCGGAGAGAGGGCCAGAAACAUUCAGACCCUGCCUGGAAUAUUCAAGACAAAGGACGGGGACAUCGGAGCAGUUGCU
KF383117        AUAUUGGGGGGACGUCAAGCAGGACUUGGUGUCAUAUUGUGGGCCGUGGAAGUUGGACGCAACCUGGGAUGGACUAAGUGAAGUGCAGCUUUUGGCCGUACCCCCCGGAGAGAGGGCUAGAAACAUUCAGACUCUGCCUGGAAUAUUCAAGACAAAGGAUGGGGACAUCGGAGCGGUUGCU
KF383116        AUAUUGGGGGGACGUCAAGCAGGACUUGGUGUCAUAUUGUGGGCCGUGGAAGUUGGAUGCAGCCUGGGAUGGACUAAGUGAAGUGCAGCUUUUGGCCGUACCCCCCGGAGAGAGGGCUAGAAACAUUCAGACUCUGCCUGGAAUAUUCAAGACAAAGGAUGGGGACAUCGGAGCAGUCGCU
KF383115        AUACUGGGGUGAUGUCAAGCAGGACUUGGUGUCAUACUGUGGGCCUUGGAAGCUGGAUGCAGCUUGGGAUGGACUUAGUGAGGUGCAGCUCUUGGCAGUACCUCCCGGAGAGAGGGCCAGAAACAUCCAGACUCUGCCUGGAAUAUUCAAGACAAAGGAUGGGGACAUCGGAGCAGUUGCU
EU545988        AUACUGGGGAGAUGUCAAGCAGGAUCUGGUGUCAUAUUGUGGUCCGUGGAAGCUAGACGCCGCCUGGGACGGGCACAGCGAGGUGCAGCUCUUGGCCGUGCCCCCGGAGAGAGAGCGAGGAACAUCCAGACUCCGCCCGGAAUAUUUAAGACAAAGGAUGGGGACAUUGGAGCAGUUGCG
KU955589        AUACUGGGGAGAUGUCAAGCAGGAUCUGGUGUCAUACUGUGGUCCAUGGAAGCUAGAUGCCGCCUGGGACGGGCACAGCGAGGUGCAGCUCUUGGCCGUGCCCCCGGAGAGAGAGCGAGGAACAUCCAGACUCUGCCCGGAAUAUUUAAGACAAAGGAUGGGGACAUUGGAGCGGUUGCG
KU955590        AUACUGGGGAGAUGUCAAGCAGGAUCUGGUGUCAUACUGUGGUCCAUGGAAGCUAGAUGCCGCCUGGGACGGGCACAGCGAGGUGCAGCUCUUGGCCGUGCCCCCGGAGAGAGAGCGAGGAACAUCCAGACUCUGCCCGGAAUAUUUAAGACAAAGGAUGGGGACAUUGGAGCGGUUGCG
KU940228        AUACUGGGGAGAUGUCAAGCAGGAUCUGGUGUCAUACUGUGGUCCAUGGAAGCUAGAUGCCGCCUGGGACGGGCACAGCGAGGUGCAGCUCUUGGCCGUGCCCCCGGAGAGAGAGCGAGGAACAUCCAGACUCUGCCCGGAAUAUUUAAGACAAAGGGUGGGGACAUUGGAGCGGUUGCG
KU991811        AUACUGGGGAGAUGUCAAGCAGGAUCUGGUGUCAUACUGUGGUCCGUGGAAGCUAGAUGCCGCCUGGGACGGGCACAGCGAGGUGCAGCUCUUGGCCGUGCCCCCGGAGAGAGAGCGAGGAACAUCCAGACUCUGCCCGGAAUAUUUAAGACAAAGGAUGGGGACAUUGGAGCGGUUGCG
KU509998        AUACUGGGGAGAUGUCAAGCAGGAUCUGGUGUCAUACUGUGGUCCAUGGAAGCUAGAUGCCGCCUGGGACGGGCACAGCGAGGUGCAGCUCUUGGCCGUGCCCCCGGAGAGAGAGCGAGGAACAUCCAGACUCUGCCCGGAAUAUUUAAGACAAAGGAUGGGGACAUUGGAGCGGUUGCG
KX056898        AUACUGGGGAGAUGUCAAGCAGGAUCUGGUGUCAUACUGUGGUCCAUGGAAGCUAGAUGCCGCCUGGGACGGGCACAGCGAGGUGCAGCUCUUGGCCGUGCCCCCGGAGAGAGAGCGAGGAACAUCCAGACUCUGCCCGGAAUAUUUAAGACAAAGGAUGGGGACAUUGGAGCGGUUGCG
KX117076        AUACUGGGGAGAUGUCAAGCAGGAUCUGGUGUCAUACUGUGGUCCAUGGAAGCUAGAUGCCGCCUGGGACGGGCACAGCGAGGUGCAGCUCUUGGCCGUGCCCCCGGAGAGAGAGCGAGGAACAUCCAGACUCUGCCCGGAAUAUUUAAGACAAAGGAUGGGGACAUUGGAGCGGUUGCG
KX185891        AUACUGGGGAGAUGUCAAGCAGGAUCUGGUGUCAUACUGUGGUCCAUGGAAGCUAGAUGCCGCCUGGGACGGGCACAGCGAGGUGCAGCUCUUGGCCGUGCCCCCGGAGAGAGAGCGAGGAACAUCCAGACUCUGCCCGGAAUAUUUAAGACAAAGGAUGGGGACAUUGGAGCGGUUGCG
KX247646        AUACUGGGGAGAUGUCAAGCAGGAUCUGGUGUCAUACUGUGGUCCAUGGAAGCUAGAUGCCGCCUGGGACGGGCACAGCGAGGUGCAGCUCUUGGCCGUGCCCCCGGAGAGAGAGCGAGGAACAUCCAGACUCUGCCCGGAAUAUUUAAGACAAAGGAUGGGGACAUUGGAGCGGUUGCG
KX262887        AUACUGGGGAGAUGUCAAGCAGGAUCUGGUGUCAUACUGUGGUCCAUGGAAGCUAGAUGCCGCCUGGGACGGGCACAGCGAGGUGCAGCUCUUGGCCGUGCCCCCGGAGAGAGAGCGAGGAACAUCCAGACUCUGCCCGGAAUAUUUAAGACAAAGGAUGGGGACAUUGGAGCGGUUGCG
KX280026        AUACUGGGGAGAUGUCAAGCAGGAUCUGGUGUCAUACUGUGGUCCAUGGAAGCUAGAUGCCGCCUGGGACGGGCACAGCGAGGUGCAGCUCUUGGCCGUGCCCCCGGAGAGAGAGCGAGGAACAUCCAGACUCUGCCCGGAAUAUUUAAGACAAAGGAUGGGGACAUUGGAGCGGUUGCG
KU758877        AUACUGGGGAGAUGUCAAGCAGGAUCUGGUGUCAUACUGUGGUCCAUGGAAGCUAGAUGCCGCCUGGGACGGGCACAGCGAGGUGCAGCUCUUGGCCGUGCCCCCGGAGAGAGAGCGAGGAACAUCCAGACUCUGCCCGGAAUAUUUAAGACAAAGGAUGGGGACAUUGGAGCGGUUGCG
KU866423        AUACUGGGGAGAUGUCAAGCAGGAUCUGGUGUCAUACUGUGGUCCAUGGAAGCUAGAUGCCGCCUGGGACGGGCACAGCGAGGUGCAGCUCUUGGCCGUGCCCCCGGAGAGAGAGCGAGGAACAUCCAGACUCUGCCCGGAAUAUUUAAGACAAAGGAUGGGGACAUUGGAGCGGUUGCG
KX377336        AUACUGGGGAGAUGUUAAGCAGGAUCUGGUGUCAUACUGUGGCCCGUGGAAGCUAGAUGCCGCUUGGGACGGACACAGCGAGGUGCAGCUUUUGGCCGUGCCCCCGGAGAGAGAGCGAGGAACAUCCAGACUCUGCCCGGAAUAUUCAAGACAAAGGAUGGGGACAUCGGAGCAGUUGCU
KX377337        AUACUGGGGAGAUGUCAAGCAGGAUCUGGUGUCAUACUGUGGUCCAUGGAAGCUAGAUGCCGCCUGGGAUGGGCACAGCGAGGUGCAGCUCUUGGCCGUGCCCCCGGAGAGAGAGCGAGGAACAUCCAGACUCUGCCCGGAAUAUUUAAGACAAAGGAUGGGGACAUUGGAGCGGUUGCG
KX369547        AUACUGGGGAGAUGUCAAGCAGGAUCUGGUGUCAUACUGUGGUCCAUGGAAGCUAGAUGCCGCCUGGGACGGGCACAGCGAGGUGCAGCUCUUGGCCGUGCCCCCGGAGAGAGAGCGAGGAACAUCCAGACUCUGCCCGGAAUAUUUAAGACAAAGGAUGGGGACAUUGGAGCGGUUGCG
KX548902        AUACUGGGGAGAUGUCAAGCAGGAUCUGGUGUCAUACUGUGGUCCAUGGAAGCUAGAUGCCGCCUGGGACGGGCACAGCGAGGUGCAGCUCUUGGCCGUGCCCCCGGAGAGAGAGCGAGGAACAUCCAGACUCUGCCCGGAAUAUUUAAGACAAAGGAUGGGGACAUUGGAGCGGUUGCG
KX601166        AUAUUGGGGGGACGUCAAGCAGGACUUGGUGUCAUAUUGUGGGCCUUGGAAGUUGGAUGCAGCCUGGGAUGGACUAAGUGAAGUGCAGCUUUUGGCCGUACCCCCCGGAGAGAGGGCUAGAAACAUUCAGACUCUGCCUGGAAUAUUCAAGACAAAGGAUGGGGACAUCGGAGCAGUCGCU
KX601167        AUACUGGGGAGAUGUUAAGCAGGAUCUGGUGUCAUACUGUGGCCCGUGGAAGCUAGAUGCCGCUUGGGACGGACACAGCGAGGUGCAGCUUUUGGCCGUGCCCCCGGAGAGAGAGCGAGGAACAUCCAGACUCUGCCCGGAAUAUUCAAGACAAAGGAUGGGGACAUCGGAGCAGUUGCU
KX601168        AUACUGGGGAGAUGUCAAGCAGGAUCUGGUGUCAUACUGUGGUCCAUGGAAGCUAGAUGCCGCCUGGGAUGGGCACAGCGAGGUGCAGCUCUUGGCCGUGCCCCCGGAGAGAGAGCGAGGAACAUCCAGACUCUGCCCGGAAUAUUUAAGACAAAGGAUGGGGACAUUGGAGCGGUUGCG
KX766028        AUACUGGGGAGAUGUCAAGCAGGAUCUGGUGUCAUACUGUGGUCCAUGGAAGCUAGAUGCCGCCUGGGACGGGCACAGCGAGGUGCAGCUCUUGGCCGUGCCCCCGGAGAGAGAGCGAGGAACAUCCAGACUCUGCCCGGAAUAUUUAAGACAAAGGAUGGGGACAUUGGAGCGGUUGCG
KX766029        AUACUGGGGAGAUGUCAAGCAGGAUCUGGUGUCAUACUGUGGUCCAUGGAAGCUAGAUGCCGCCUGGGACGGGCACAGCGAGGUGCAGCUCUUGGCCGUGCCCCCGGAGAGAGAGCGAGGAACAUCCAGACUCUGCCCGGAAUAUUUAAGACAAAGGAUGGGGACAUUGGAGCGGUUGCG
KU940224        AUACUGGGGAGAUGUCAAGCAGGAUCUGGUGUCAUACUGUGGUCCAUGGAAGCUAGAUGCCGCCUGGGACGGGCACAGCGAGGUGCAGCUCUUGGCCGUGCCCCCGGAGAGAGAGCGAGGAACAUCCAGACUCUGCCCGGAAUAUUUAAGACAAAGGGUGGGGACAUUGGAGCGGUUGCG


                4444444444444444444444444444444444444444444444444444444444444444444444444444444444444444444444444444444444444444555555555555555555555555555555555555555555555555555555555555555555555
                8888888888889999999999999999999999999999999999999999999999999999999999999999999999999999999999999999999999999999000000000000000000000000000000000000000000000000000000000000000000000
                8899999999990000000000111111111122222222223333333333444444444455555555556666666666777777777788888888889999999999000000000011111111112222222222333333333344444444445555555555666666666
Sequence        8901234567890123456789012345678901234567890123456789012345678901234567890123456789012345678901234567890123456789012345678901234567890123456789012345678901234567890123456789012345678
-----------------------------------------------------------------------------------------------------------------------------------------------------------------------------------------------------
NC_012532       CUGGACUACCCUGCAGGGACCUCAGGAUCUCCGAUCCUAGACAAAUGUGGAAGAGUGAUAGGACUCUAUGGCAAUGGGGUUGUGAUCAAGAAUGGAAGCUAUGUUAGUGCUAUAACCCAGGGAAAGAGGGAGGAGGAGACUCCGGUUGAAUGUUUCGAACCCUCGAUGCUGAAGAAGAAGC
KU922960        CUGGAUUACCCAGCAGGAACUUCAGGAUCUCCAAUCCUAGACAAGUGUGGGAGAGUGAUAGGACUUUAUGGCAAUGGGGUCGUGAUCAAAAAUGGGAGUUAUGUUAGUGCCAUCACCCAAGGGAGGAGGGAGGAAGAGACUCCUGUUGAGUGCUUCGAGCCUUCGAUGCUGAAGAAGAAGC
KU922923        CUGGAUUACCCAGCAGGAACUUCAGGAUCUCCAAUCCUAGACAAGUGUGGGAGAGUGAUAGGACUUUAUGGCAAUGGGGUCGUGAUCAAAAAUGGGAGUUAUGUUAGUGCCAUCACCCAAGGGAGGAGGGAGGAAGAGACUCCUGUUGAGUGCUUCGAGCCUUCGAUGCUGAAGAAGAAGC
KU820898        CUGGAUUACCCAGCAGGAACUUCAGGAUCUCCAAUCCUAGACAAGUGUGGGAGAGUGAUAGGACUUUAUGGCAAUGGGGUCGUGAUCAAAAAUGGGAGUUAUGUUAGUGCCAUCACCCAAGGGAGGAGGGAGGAAGAGACUCCUGUUGAGUGCUUCGAGCCUUCGAUGCUGAAGAAGAAGC
KU321639        CUGGAUUACCCAGCAGGAACUUCAGGAUCUCCAAUCCUAGACAAGUGUGGGAGAGUGAUAGGACUUUAUGGCAAUGGGGUCGUGAUCAAAAAUGGGAGUUAUGUUAGUGCCAUCACCCAAGGGAGGAGGGAGGAAGAGACUCCUGUUGAGUGCUUCGAGCCUUCGAUGCUGAAGAAGAAGC
KU820899        CUGGAUUACCCAGCAGGAACUUCAGGAUCUCCAAUCCUAGACAAGUGUGGGAGAGUGAUAGGACUUUAUGGCAAUGGGGUCGUGAUCAAAAAUGGGAGUUAUGUUAGUGCCAUCACCCAAGGGAGGAGGGAGGAAGAGACUCCUGUUGAGUGCUUCGAGCCUUCGAUGCUGAAGAAGAAGC
KU853012        CUGGAUUACCCAGCAGGAACUUCAGGAUCUCCAAUCCUAGACAAGUGUGGGAGAGUGAUAGGACUUUAUGGCAAUGGGGUCGUGAUCAAAAAUGGGAGUUAUGUUAGUGCCAUCACCCAAGGGAGGAGGGAGGAAGAGACUCCUGUUGAGUGCUUCGAGCCUUCGAUGCUGAAGAAGAAGC
```

```
KU729217    CUGGAUUACCCAGCAGGAACUUCAGGAUCUCCAAUCCUAGACAAGUGUGGGAGAGUGAUAGGACUUUAUGGCAAUGGGGUCGUGAUCAAAAAUGGGAGUUAUGUUAGUGCCAUCACCCAAGGGAGGAGGGAGGAAGAGACUCCUGUUGAGUGCUUCGAGCCUUCGAUGCUGAAGAAGAAGC
KU729218    CUGGAUUACCCAGCAGGAACUUCAGGAUCUCCAAUCCUAGACAAGUGCGGGAGAGUGAUAGGACUUUAUGGCAAUGGGGUCGUGAUCAAAAAUGGGAGUUAUGUUAGUGCCAUCACCCAAGGGAGGAGGGAGGAAGAGACUCCUGUUGAGUGCUUCGAGCCUUCGAUGCUGAAGAAGAAGC
KU761564    CUGGAUUACCCAGCAGGAACUUCAGGAUCUCCAAUCCUAGACAAGUGUGGGAGAGUGAUAGGACUUUAUGGCAAUGGGGUCGUGAUCAAAAAUGGGAGUUAUGUUAGUGCCAUCACCCAAGGGAGGAGGGAGGAAGAGACUCCUGUUGAGUGCUUCGAGCCUUCGAUGCUGAAGAAGAAGC
KU740184    CUGGAUUACCCAGCAGGAACUUCAGGAUCUCCAAUCCUAGACAAGUGUGGGAGAGUGAUAGGACUUUAUGGCAAUGGGGUCGUGAUCAAAAAUGGGAGUUAUGUUAGUGCCAUCACCCAAGGGAGGAGGGAGGAAGAGACUCCUGUUGAGUGCUUCGAGCCUUCGAUGCUGAAGAAGAAGC
KU744693    CUGGAUUACCCAGCAGGAACUUCAGGAUCUCCAAUCCUAGACAAGUGUGGGAGAGUGAUAGGACUUUAUGGCAAUGGGGUCGUGAUCAAAAAUGGGAGUUAUGUUAGUGCCAUCACCCAAGGGAGGAGGGAGGAAGAGACUCCUGUUGAGUGCUUCGAGCCUUCGAUGCUGAAGAAGAAGC
KU497555    CUGGAUUACCCAGCAGGAACUUCAGGAUCUCCAAUCCUAGACAAGUGUGGGAGAGUGAUAGGACUUUAUGGCAAUGGGGUCGUGAUAAAAAAUGGGAGUUAUGUUAGUGCCAUCACCCAAGGGAGGAGGGAGGAAGAGACUCCUGUUGAGUGCUUCGAGCCUUCGAUGCUGAAGAAGAAGC
KU527068    CUGGAUUACCCAGCAGGAACUUCAGGAUCUCCAAUCCUAGACAAGUGUGGGAGAGUGAUAGGACUUUAUGGCAAUGGGGUCGUGAUCAAAAAUGGGAGUUAUGUUAGUGCCAUCACCCAAGGGAGGAGGGAGGAAGAGACUCCUGUUGAGUGCUUCGAGCCUUCGAUGCUGAAGAAGAAGC
KU501217    CUGGAUUACCCAGCAGGAACUUCAGGAUCUCCAAUCCUAGACAAGUGUGGGAGAGUGAUAGGACUUUAUGGCAAUGGGGUCGUGAUCAAAAAUGGGAGUUAUGUUAGUGCCAUCACCCAAGGGAGGAGGGAGGAAGAGACUCCUGUUGAGUGCUUCGAGCCUUCGAUGCUGAAGAAGAAGC
KU501216    CUGGAUUACCCAGCAGGAACUUCAGGAUCUCCAAUCCUAGACAAGUGUGGGAGAGUGAUAGGACUUUAUGGCAAUGGGGUCGUGAUCAAAAAUGGGAGUUAUGUUAGUGCCAUCACCCAAGGGAGGAGGGAGGAAGAGACUCCUGUUGAGUGCUUCGAGCCUUCGAUGCUGAAGAAGAAGC
KU501215    CUGGAUUACCCAGCAGGAACUUCAGGAUCUCCAAUCCUAGACAAGUGUGGGAGAGUGAUAGGACUUUAUGGCAAUGGGGUCGUGAUCAAAAACGGGAGUUAUGUUAGUGCCAUCACCCAAGGGAGGAGGGAGGAAGAGACUCCUGUUGAGUGCUUCGAGCCCUCGAUGCUGAAGAAGAAGC
KU365780    CUGGAUUACCCAGCAGGAACUUCAGGAUCUCCAAUCCUAGACAAGUGUGGGAGAGUGAUAGGACUUUAUGGCAAUGGGGUCGUGAUCAAAAAUGGGAGUUAUGUUAGUGCCAUCACCCAAGGGAGGAGGGAGGAAGAGACUCCUGUUGAGUGCUUCGAGCCUUCGAUGCUGAAGAAGAAGC
KU365779    CUGGAUUACCCAGCAGGAACUUCAGGAUCUCCAAUCCUAGACAAGUGUGGGAGAGUGAUAGGACUUUAUGGCAAUGGGGUCGUGAUCAAAAAUGGGAGUUAUGUUAGUGCCAUCACCCAAGGGAGGAGGGAGGAAGAGACUCCUGUUGAGUGCUUCGAGCCUUCGAUGCUGAAGAAGAAGC
KU707826    CUGGAUUACCCAGCAGGAACUUCAGGAUCUCCAAUCCUAGACAAGUGUGGGAGAGUGAUAGGACUUUAUGGCAAUGGGGUCGUGAUCAAAAAUGGGAGUUAUGUUAGUGCCAUCACCCAAGGGAGGAGGGAGGAAGAGACUCCUGUUGAGUGCUUCGAGCCUUCGAUGCUGAAGAAGAAGC
KU365778    CUGGAUUACCCAGCAGGAACUUCAGGAUCUCCAAUCCUAGACAAGUGUGGGAGAGUGAUAGGACUUUAUGGCAAUGGGGUCGUGAUCAAAAAUGGGAGUUAUGUUAGUGCCAUCACCCAAGGGAGGAGGGAAGAAGAGACUCCUGUUGAGUGCUUCGAGCCCUCGAUGCUGAAGAAGAAGC
KU365777    CUGGAUUACCCAGCAGGAACUUCAGGAUCUCCAAUCCUAGACAAGUGUGGGAGAGUGAUAGGACUUUAUGGCAAUGGGGUCGUGAUCAAAAAUGGGAGUUAUGUUAGUGCCAUCACCCAAGGGAGGAGGGAGGAAGAGACUCCUGUUGAGUGCUUCGAGCCUUCGAUGCUGAAGAAGAAGC
KU312312    CUGGAUUACCCAGCAGGAACUUCAGGAUCUCCUAUCCUAGACAAGUGUGGGAGAGUGAUAGGACUUUAUGGCAAUGGGGUCGUGAUCAAAAAUGGGAGUUAUGUUAGUGCCAUCACCCAAGGGAGGAGGGAGGAAGAGACUCCUGUUGAGUGCUUCGAGCCUUCGAUGCUGAAGAAGAAGC
KF268950    CUGGACUACCCCGCAGGAACUUCAGGAUCCCCGAUCCUAGACAAAUGCGGAAGAGUGAUAGGACUUUAUGGCAAUGGGGUUGUGAUCAAGAAUGGAAGCUAUGUUAGUGCCAUAACCCAGGGAAAGAGGGAGGAGGAGACUCCGGUUGAAUGUUUCGAACCCUCGAUGCUGAAGAAGAAGC
KF268949    CUGGACUACCCCGCAGGAACUUCAGGAUCCCCGAUCCUAGACAAAUGCGGAAGAGUGAUAGGACUUUAUGGCAAUGGGGUUGUGAUCAAGAAUGGAAGCUAUGUUAGUGCCAUAACCCAGGGAAAGAGGGAGGAGGAGACUCCGGUUGAAUGUUUCGAACCCUCGAUGCUGAAGAAGAAGC
KF268948    CUGGACUACCCCGCAGGAACUUCAGGAUCCCCGAUCCUAGACAAAUGCGGAAGAGUGAUAGGACUUUAUGGCAAUGGGGUUGUGAUCAAGAAUGGAAGCUAUGUUAGUGCCAUAACCCAGGGAAAGAGGGAGGAGGAGACUCCGGUUGAAUGUUUCGAACCCUCGAUGCUGAAGAAGAAGC
KJ776791    CUGGAUUACCCAGCAGGAACUUCAGGAUCUCCAAUCCUAGACAAGUGUGGGAGAGUGAUAGGACUUUAUGGCAAUGGGGUCGUGAUCAAAAAUGGGAGUUAUGUUAGUGCCAUCACCCAAGGGAGGAGGGAGGAAGAGACUCCUGUUGAGUGCUUCGAGCCUUCGAUGCUGAAGAAGAAGC
KF383119    CUGGACUACCCUGCAGGGACCUCAGGAUCUCCGAUCCUAGACAAAUGUGGAAGAGUGAUAGGACUCUAUGGCAAUGGGGUUGUGAUCAAGAAUGGAAGCUAUGUUAGUGCUAUAACCCAGGGAAAGAGGGAGGAGGAGACUCCGGUUGAAUGUUUCGAACCCUCGAUGCUGAAGAAGAAGC
KF383118    CUGGACUACCCUGCAGGGACCUCAGGAUCUCCGAUCCUAGACAAAUGUGGAAGAGUGAUAGGACUCUAUGGCAAUGGGGUUGUGAUCAAGAAUGGAAGCUAUGUUAGUGCUAUAACCCAGGGAAAGAGGGAGGAGGAGACUCCGGUUGAAUGUUUCGAACCCUCGAUGCUGAAGAAGAAGC
KF383117    CUAGACUACCCCGCAGGAACCUCAGGAUCUCCAAUCCUAGACAAAUGCGGAAGAGUGAUAGGACUUUAUGGCAAUGGGGUUGUGAUCAAGAAUGGAAGCUAUGUCAGUGCAAUAACCCAGGGAAAAAGGGAGGAGGAGGCCCCAGUUGAGUGCUUUGAACCCUCGAUGCUGAGGAAGAAGC
KF383116    CUAGACUACCCCGCAGGAACCUCAGGAUCUCCAAUCCUAGACAAAUGCGGAAGAGUGAUAGGACUUUAUGGCAAUGGGGUUGUGAUCAAGAAUGGAAGCUAUGUUAGUGCCAUAACCCAGGGAAAAAGGGAGGAGGAGGCUCCAGUUGAGUGCUUUGAACCCUCGAUGCUGAGGAAGAAGC
KF383115    CUGGACUACCCCGCAGGAACCUCAGGAUCUCCGAUCCUAGACAAAUGCGGAAGAGUGAUAGGACUUUAUGGCAAUGGGGUUGUGAUCAAGAAUGGAAGCUAUGUUAGUGCCAUAACCCAGGGAAAGAGGGAGGAGGAGACUCCGGUUGAAUGUUUCGAACCCUCGAUGCUGAAGAAGAAGC
EU545988    UUGGACUACCCAGCAGGAACUUCAGGAUCUCCAAUCCUAGACAAGUGUGGGAGAGUGAUAGGACUCUAUGGUAAUGGGGUCGUGAUAAAAAAUGGGAGUUAUGUUAGUGCCAUCACCCAAGGGAGGAGGGAGGAAGAGACUCCUGUUGAGUGCUUCGAGCCUUCGAUGUUGAAGAAGAAGC
KU955589    CUGGAUUACCCAGCAGGAACUUCAGGAUCUCCAAUCCUAGACAAGUGUGGGAGAGUGAUAGGACUUUAUGGCAAUGGGGUCGUGAUCAAAAAUGGGAGUUAUGUUAGUGCCAUCACCCAAGGGAGGAGGGAGGAAGAGACUCCUGUUGAGUGCUUCGAGCCUUCGAUGCUGAAGAAGAAGC
KU955590    CUGGAUUACCCAGCAGGAACUUCAGGAUCUCCAAUCCUAGACAAGUGUGGGAGAGUGAUAGGACUUUAUGGCAAUGGGGUCGUGAUCAAAAAUGGGAGUUAUGUUAGUGCCAUCACCCAAGGGAGGAGGGAGGAAGAGACUCCUGUUGAGUGCUUCGAGCCUUCGAUGCUGAAGAAGAAGC
KU940228    CUGGAUUACCCAGCAGGAACUUCAGGAUCUCCAAUCCUAGACAAGUGUGGGAGAGUGAUAGGACUUUAUGGCAAUGGGGUCGUGAUCAAAAAUGGGAGUUAUGUUAGUGCCAUCACCCAAGGGAGGAGGGAGGAAGAGACUCCUGUUGAGUGCUUCGAGCCUUCGAUGCUGAAGAAGAAGC
KU991811    CUGGAUUACCCAGCAGGAACUUCAGGAUCUCCAAUCCUAGACAAGUGUGGGAGAGUGAUAGGACUUUAUGGCAAUGGGGUCGUGAUCAAAAAUGGGAGUUAUGUUAGUGCCAUCACCCAAGGGAGGAGGGAGGAAGAGACUCCUGUUGAUUGCUUCGAGCCUUCGAUGCUGAAGAAGAAGC
KU509998    CUGGAUUACCCAGCAGGAACUUCAGGAUCUCCAAUCCUAGACAAGUGUGGGAGAGUGAUAGGACUUUAUGGCAAUGGGGUCGUGAUCAAAAAUGGGAGUUAUGUUAGUGCCAUCACCCAAGGGAGGAGGGAGGAAGAGACUCCUGUUGAGUGCUUCGAGCCUUCGAUGCUGAAGAAGAAGC
KX056898    CUGGAUUACCCAGCAGGAACUUCAGGAUCUCCAAUCCUAGACAAGUGUGGGAGAGUGAUAGGACUUUAUGGCAAUGGGGUCGUGAUCAAAAAUGGGAGUUAUGUUAGUGCCAUCACCCAAGGGAGGAGGGAGGAAGAGACUCCUGUUGAGUGCUUCGAGCCUUCGAUGCUGAAGAAGAAGC
KX117076    CUGGAUUACCCAGCAGGAACUUCAGGAUCUCCAAUCCUAGACAAGUGUGGGAGAGUGAUAGGACUUUAUGGCAAUGGGGUCGUGAUCAAAAAUGGGAGUUAUGUUAGUGCCAUCACCCAAGGGAGGAGGGAGGAAGAGACUCCUGUUGAGUGCUUCGAGCCUUCGAUGCUGAAGAAGAAGC
KX185891    CUGGAUUACCCAGCAGGAACUUCAGGAUCUCCAAUCCUAGACAAGUGUGGGAGAGUGAUAGGACUUUAUGGCAAUGGGGUCGUGAUCAAAAAUGGGAGUUAUGUUAGUGCCAUCACCCAAGGGAGGAGGGAGGAAGAGACUCCUGUUGAGUGCUUCGAGCCUUCGAUGCUGAAGAAGAAGC
KX247646    CUGGAUUACCCAGCAGGAACUUCAGGAUCUCCAAUCCUAGACAAGUGUGGGAGAGUGAUAGGACUUUAUGGCAAUGGGGUCGUGAUCAAAAAUGGGAGUUAUGUUAGUGCCAUCACCCAAGGGAGGAGGGAGGAAGAGACUCCUGUUGAGUGCUUCGAGCCUUCGAUGCUGAAGAAGAAGC
KX262887    CUGGAUUACCCAGCAGGAACUUCAGGAUCUCCAAUCCUAGACAAGUGUGGGAGAGUGAUAGGACUUUAUGGCAAUGGGGUCGUGAUCAAAAAUGGGAGUUAUGUUAGUGCCAUCACCCAAGGGAGGAGGGAGGAAGAGACUCCUGUUGAGUGCUUCGAGCCUUCGAUGCUGAAGAAGAAGC
KX280026    CUGGAUUACCCAGCAGGAACUUCAGGAUCUCCAAUCCUAGACAAGUGUGGGAGAGUGAUAGGACUUUAUGGCAAUGGGGUCGUGAUCAAAAAUGGGAGUUAUGUUAGUGCCAUCACCCAAGGGAGGAGGGAGGAAGAGACUCCUGUUGAGUGCUUCGAGCCUUCGAUGCUGAAGAAGAAGC
KU758877    CUGGAUUACCCAGCAGGAACUUCAGGAUCUCCAAUCCUAGACAAGUGUGGGAGAGUGAUAGGACUUUAUGGCAAUGGGGUCGUGAUCAAAAAUGGGAGUUAUGUUAGUGCCAUCACCCAAGGGAGGAGGGAGGAAGAGACUCCUGUUGAGUGCUUCGAGCCCUCGAUGCUGAAGAAGAAGC
KU866423    CUGGAUUACCCAGCAGGAACUUCAGGAUCUCCAAUCCUAGACAAGUGUGGGAGAGUGAUAGGACUUUAUGGCAAUGGGGUCGUGAUCAAAAAUGGGAGUUAUGUUAGUGCCAUCACCCAAGGGAGGAGGGAGGAAGAGACUCCUGUUGAGUGCUUCGAGCCUUCGAUGCUGAAGAAGAAGC
KX377336    CUGGACUACCCAGCAGGAACUUCAGGAUCUCCGAUCCUAGACAAGUGUGGGAGAGUGAUAGGACUCUAUGGCAAUGGGGUCGUGAUCAAAAAUGGAAGUUAUGUUAGUGCCAUCACCCAAGGGAGGAGGGAGGAAGAGACUCCUGUUGAAUGCUUCGAACCUUCGAUGCUGAAGAAGAAGC
KX377337    CUGGAUUACCCAGCAGGAACUUCAGGAUCUCCAAUCCUAGACAAGUGUGGGAGAGUGAUAGGACUUUAUGGCAAUGGGGUCGUGAUCAAAAACGGGAGUUAUGUUAGUGCCAUCACCCAAGGGAGGAGGGAGGAAGAGACUCCUGUUGAGUGCUUCGAGCCCUCGAUGCUGAAGAAGAAGC
KX369547    CUGGAUUACCCAGCAGGAACUUCAGGAUCUCCAAUCCUAGACAAGUGUGGGAGAGUGAUAGGACUUUAUGGCAAUGGGGUCGUGAUCAAAAAUGGGAGUUAUGUUAGUGCCAUCACCCAAGGGAGGAGGGAGGAAGAGACUCCUGUUGAGUGCUUCGAGCCUUCGAUGCUGAAGAAGAAGC
KX548902    CUGGAUUACCCAGCAGGAACUUCAGGAUCUCCAAUCCUAGACAAGUGUGGGAGAGUGAUAGGACUUUAUGGCAAUGGGGUCGUGAUCAAAAAUGGGAGUUAUGUUAGUGCCAUCACCCAAGGGAGGAGGGAGGAAGAGACUCCUGUUGAGUGCUUCGAGCCUUCGAUGCUGAAGAAGAAGC
KX601166    CUAGACUACCCCGCAGGAACCUCAGGAUCUCCAAUCCUAGACAAAUGCGGAAGAGUGAUAGGACUUUAUGGCAAUGGGGUUGUGAUCAAGAAUGGAAGCUAUGUUAGUGCCAUAACCCAGGGAAAAAGGGAGGAGGAGGCUCCAGUUGAGUGCUUUGAACCCUCGAUGCUGAGGAAGAAGC
KX601167    CUGGACUACCCAGCAGGAACUUCAGGAUCUCCGAUCCUAGACAAGUGUGGGAGAGUGAUAGGACUCUAUGGCAAUGGGGUCGUGAUCAAAAAUGGAAGUUAUGUUAGUGCCAUCACCCAAGGGAGGAGGGAGGAAGAGACUCCUGUUGAAUGCUUCGAACCUUCGAUGCUGAAGAAGAAGC
KX601168    CUGGAUUACCCAGCAGGAACUUCAGGAUCUCCAAUCCUAGACAAGUGUGGGAGAGUGAUAGGACUUUAUGGCAAUGGGGUCGUGAUCAAAAACGGGAGUUAUGUUAGUGCCAUCACCCAAGGGAGGAGGGAGGAAGAGACUCCUGUUGAGUGCUUCGAGCCCUCGAUGCUGAAGAAGAAGC
KX766028    CUGGAUUACCCAGCAGGAACUUCAGGAUCUCCAAUCCUAGACAAGUGUGGGAGAGUGAUAGGACUUUAUGGCAAUGGGGUCGUGAUCAAAAAUGGGAGUUAUGUUAGUGCCAUCACCCAAGGGAGGAGGGAGGAAGAGACUCCUGUUGAGUGCUUCGAGCCUUCGAUGCUGAAGAAGAAGC
KX766029    CUGGAUUACCCAGCAGGAACUUCAGGAUCUCCAAUCCUAGACAAGUGUGGGAGAGUGAUAGGACUUUAUGGCAAUGGGGUCGUGAUCAAAAACGGGAGUUAUGUUAGUGCCAUCACCCAAGGGAGGAGGGAGGAAGAGACUCCUGUUGAGUGCUUCGAGCCUUCGAUGCUGAAGAAGAAGC
KU940224    CUGGAUUACCCAGCAGGAACUUCAGGAUCUCCAAUCCUAGACAAGUGUGGGAGAGUGAUAGGACUUUAUGGCAAUGGGGUCGUGAUCAAAAAUGGGAGUUAUGUUAGUGCCAUCACCCAAGGGAGGAGGGAGGAAGAGACUCCUGUUGAGUGCUUCGAGCCUUCGAUGCUGAAGAAGAAGC


            5555555555555555555555555555555555555555555555555555555555555555555555555555555555555555555555555555555555555555555555555555555555555555555555555555555555555555555555555555555555555
            0000000000000000000000000000000111111111111111111111111111111111111111111111111111111111111111111111111111111111111111111111111111111112222222222222222222222222222222222222222222222222
            6777777777788888888889999999999000000000011111111112222222222333333333344444444445555555555666666666677777777778888888888999999999900000000001111111111222222222233333333334444444444
Sequence    9012345678901234567890123456789012345678901234567890123456789012345678901234567890123456789012345678901234567890123456789012345678901234567890123456789012345678901234567890123456789
--------------------------------------------------------------------------------------------------------------------------------------------------------------------------------------------------------
NC_012532   AGCUAACUGUCUUGGAUCUGCAUCCAGGAGCCGGAAAAACCAGGAGAGUUCUUCCUGAAAUAGUCCGUGAAGCCAUAAAAAAAGAGACUCCGGACAGUGAUCUUGGCACCAACUAGGGUUGUCGCUGCUGAGAUGGAGGAGGCCUUGAGAGGACUUCCGGUGCGUUACAUGACAACAGCAGU
KU922960    AGCUAACUGUCUUAGACUUGCAUCCUGGAGCUGGGAAAACCAGGAGAGUUCUUCCUGAAAUAGUCCGUGAAGCCAUAAAAACAAGACUCCGUACUGUGAUCUUAGCUCCAACCAGGGUUGUCGCUGCUGAAAUGGAGGAAGCCCUUAGAGGGCUUCCAGUGCGUUAUAUGACAACAGCAGU
KU922923    AGCUAACUGUCUUAGACUUGCAUCCUGGAGCUGGGAAAACCAGGAGAGUUCUUCCUGAAAUAGUCCGUGAAGCCAUAAAAACAAGACUCCGUACUGUGAUCUUAGCUCCAACCAGGGUUGUCGCUGCUGAAAUGGAGGAAGCCCUUAGAGGGCUUCCAGUGCGUUAUAUGACAACAGCAGU
KU820898    AGCUAACUGUCUUAGACUUGCAUCCUGGAGCUGGGAAAACCAGGAGAGUUCUUCCUGAAAUAGUCCGUGAAGCCAUAAAAACAAGACUCCGUACUGUGAUCUUAGCUCCAACCAGGGUUGUCGCUGCUGAAAUGGAGGAGGCCCUUAGAGGGCUUCCAGUGCGUUAUAUGACAACAGCAGU
KU321639    AGCUAACUGUCUUAGACUUGCAUCCUGGAGCUGGGAAAACCAGGAGAGUUCUUCCUGAAAUAGUCCGUGAAGCCAUAAAAACAAGACUCCGUACUGUGAUCUUAGCUCCAACCAGGGUUGUCGCUGCUGAAAUGGAGGAAGCCCUUAGAGGGCUUCCAGUGCGUUAUAUGACAACAGCAGU
KU820899    AGCUAACUGUCUUAGACUUGCAUCCUGGAGCUGGGAAAACCAGGAGAGUUCUUCCUGAAAUAGUCCGUGAAGCCAUAAAAACAAGACUCCGUACUGUGAUCUUAGCUCCAACCAGGGUUGUCGCUGCCGAAAUGGAGGAAGCCCUUAGAGGGCUUCCAGUGCGUUAUAUGACAACAGCAGU
KU853012    AGCUAACUGUCUUAGACUUGCAUCCUGGAGCUGGGAAAACCAGGAGAGUUCUUCCUGAAAUAGUCCGUGAAGCUAUAAAAACAAGACUCCGUACUGUGAUCUUAGCUCCAACCAGGGUUGUCGCUGCUGAAAUGGAGGAAGCCCUUAGAGGGCUUCCAGUGCGUUAUAUGACAACAGCAGU
KU729217    AGCUAACUGUCUUAGACUUGCAUCCUGGAGCUGGGAAAACCAGGAGAGUUCUUCCUGAAAUAGUCCGUGAAGCCAUAAAAACAAGACUCCGUACUGUGAUCUUAGCUCCAACCAGGGUUGUCGCUGCUGAAAUGGAGGAAGCCCUUAGAGGGCUUCCAGUGCGUUAUAUGACAACAGCAGU
KU729218    AGCUAACUGUCUUAGACUUGCAUCCUGGAGCUGGGAAAACCAGGAGAGUUCUUCCUGAAAUAGUCCGUGAAGCCAUAAAAACAAGACUCCGUACUGUGAUCUUAGCUCCAACCAGGGUUGUCGCUGCUGAAAUGGAGGAGGCCCUUAGAGGGCUUCCAGUGCGUUAUAUGACAACAGCAGU
KU761564    AGCUAACUGUCUUAGACUUGCAUCCUGGAGCUGGGAAAACCAGGAGAGUUCUUCCUGAAAUAGUCCGUGAAGCCAUAAAAACAAGACUCCGUACUGUGAUCUUAGCUCCAACCAGGGUUGUCGCUGCUGAAAUGGAGGAGGCCCUUAGAGGGCUUCCAGUGCGUUAUAUGACAACAGCAGU
KU740184    AGCUAACUGUCUUAGACUUGCAUCCUGGAGCUGGGAAAACCAGGAGAGUUCUUCCUGAAAUAGUCCGUGAAGCCAUAAAAACAAGACUCCGUACUGUGAUCUUAGCUCCAACCAGGGUUGUCGCUGCUGAAAUGGAGGAGGCCCUUAGAGGGCUUCCAGUGCGUUAUAUGACAACAGCAGU
KU744693    AGCUAACUGUCUUAGACUUGCAUCCUGGAGCUGGGAAAACCAGGAGAGUUCUUCCUGAAAUAGUCCGUGAAGCCAUAAAAACAAGACUCCGUACUGUGAUCUUGGCUCCAACCAGGGUUGUCGCUGCUGAAAUGGAGGAGGCCCUUAGAGGGCUUCCAGUGCGUUAUAUGACAACAGCAGU
KU497555    AGCUAACUGUCUUAGACUUGCAUCCUGGAGCUGGGAAAACCAGGAGAGUUCUUCCUGAAAUAGUCCGUGAAGCCAUAAAAACAAGACUCCGUACUGUGAUCUUAGCUCCAACCAGGGUUGUCGCUGCUGAAAUGGAGGAAGCCCUUAGAGGGCUUCCAGUGCGUUAUAUGACAACAGCAGU
KU527068    AGCUAACUGUCUUAGACUUGCAUCCUGGAGCUGGGAAAACCAGGAGAGUUCUUCCUGAAAUAGUCCGUGAAGCCAUAAAAACAAGACUCCGUACUGUGAUCUUAGCUCCAACCAGGGUUGUCGCUGCUGAAAUGGAGGAAGCCCUUAGAGGGCUUCCAGUGCGUUAUAUGACAACAGCAGU
KU501217    AGCUAACUGUCUUAGACUUACAUCCUGGAGCUGGGAAAACCAGGAGAGUUCUUCCUGAAAUAGUCCGUGAAGCCAUAAAAACAAGACUCCGUACUGUGAUCUUAGCUCCAACCAGGGUUGUCGCUGCUGAAAUGGAGGAGGCCCUUAGAGGGCUUCCAGUGCGUUAUAUGACAACAGCAGU
KU501216    AGCUAACUGUCUUAGACUUACAUCCUGGAGCUGGGAAAACCAGGAGAGUUCUUCCUGAAAUAGUCCGUGAAGCCAUAAAAACAAGACUCCGUACUGUGAUCUUAGCUCCAACCAGGGUUGUCGCUGCUGAAAUGGAGGAGGCCCUUAGAGGGCUUCCAGUGCGUUAUAUGACAACAGCAGU
KU501215    AGCUAACUGUCUUAGACUUGCAUCCUGGAGCUGGGAAAACCAGGAGAGUUCUUCCUGAAAUAGUCCGUGAAGCCAUAAAAACAAGACUCCGUACUGUGAUCUUAGCUCCAACCAGGGUUGUCGCUGCUGAAAUGGAGGAGGCCCUUAGAGGGCUUCCAGUGCGUUAUAUGACAACAGCAGU
KU365780    AGCUAACUGUCUUAGACUUGCAUCCUGGAGCUGGGAAAACCAGGAGAGUUCUUCCUGAAAUAGUCCGUGAAGCCAUAAAAACAAGACUCCGCACCGUGAUCUUAGCUCCAACCAGGGUUGUCGCUGCUGAAAUGGAGGAGGCCCUUAGAGGGCUUCCAGUGCGUUAUAUGACAACAGCAGU
KU365779    AGCUAACUGUCUUAGACUUGCAUCCUGGAGCUGGGAAAACCAGGAGAGUUCUUCCUGAAAUAGUCCGUGAAGCCAUAAAAACAAGACUCCGUACUGUGAUCUUAGCUCCAACCAGGGUUGUCGCUGCUGAAAUGGAGGAGGCCCUUAGAGGGCUUCCAGUGCGUUAUAUGACAACAGCAGU
KU707826    AGCUAACUGUCUUAGACUUGCAUCCUGGAGCUGGGAAAACCAGGAGAGUUCUUCCUGAAAUAGUCCGUGAAGCCAUAAAAACAAGACUCCGUACUGUGAUCUUAGCUCCAACCAGGGUUGUCGCUGCUGAAAUGGAGGAGGCCCUUAGAGGGCUUCCAGUGCGUUAUAUGACAACAGCAGU
KU365778    AGCUAACUGUCUUAGACUUGCAUCCUGGAGCUGGGAAAACCAGGAGAGUUCUUCCUGAAAUAGUCCGUGAAGCCAUAAAAACAAGACUCCGUACUGUGAUCUUAGCUCCAACCAGGGUUGUCGCUGCUGAAAUGGAGGAGGCCCUUAGAGGGCUUCCAGUGCGUUAUAUGACAACAGCAGU
KU365777    AGCUAACUGUCUUAGACUUGCAUCCUGGAGCUGGGAAAACCAGGAGAGUUCUUCCUGAAAUAGUCCGUGAAGCCAUAAAAACAAGACUCCGCACCGUGAUCUUAGCUCCAACCAGGGUUGUCGCUGCUGAAAUGGAGGAGGCCCUUAGAGGGCUUCCAGUGCGUUAUAUGACAACAGCAGU
```

KU312312	AGCUAACUGUCUUAGACUUGCAUCCUGGAGCUGGGAAAACCAGGAGAGUUCUUCCUGAAAUAGUCCGUGAAGCCAUAAAAACAAGACUCCGUACUGUGAUCUUAGCUCCAACCAGGGUUGUCGCUGCUGAAAUGGAGGAGGCCCUUAGAGGGCUUCCAGUGCGUUAUAUGACAACAGCAGU
KF268950	AGCUAACUGUCCUGGAUCUGCAUCCAGGAGCCGGAAAGACUAGGAGAGUUCUUCCUGAAAUAGUCCGUGAAGCCAUAAAAAAGAGACUCCGCACAGUGAUUUUGGCUCCAACCAGGGUUGUCGCUGCUGAGAUGGAGGAAGCCUUGAGAGGACUUCCGGUGCGUUACAUGACAACAGCAGU
KF268949	AGCUAACUGUCCUGGAUCUGCAUCCAGGAGCCGGAAAGACUAGGAGAGUUCUUCCUGAAAUAGUCCGUGAAGCCAUAAAAAAGAGACUCCGCACAGUGAUUUUGGCUCCAACCAGGGUUGUCGCUGCUGAGAUGGAGGAAGCCUUGAGAGGACUUCCGGUGCGUUACAUGACAACAGCAGU
KF268948	AGCUAACUGUCCUGGAUCUGCAUCCAGGAGCCGGAAAGACUAGGAGAGUUCUUCCUGAAAUAGUCCGUGAAGCCAUAAAAAAGAGACUCCGCACAGUGAUUUUGGCUCCAACCAGGGUUGUCGCUGCUGAGAUGGAGGAAGCCUUGAGAGGACUUCCGGUGCGUUACAUGACAACAGCAGU
KJ776791	AGCUAACUGUCUUAGACUUGCAUCCUGGAGCUGGGAAAACCAGGAGAGUUCUUCCUGAAAUAGUCCGUGAAGCCAUAAAAACAAGACUCCGUACUGUGAUCUUAGCUCCAACCAGGGUUGUCGCUGCUGAAAUGGAGGAAGCCCUUAGAGGGCUUCCAGUGCGUUAUAUGACAACAGCAGU
KF383119	AGCUAACUGUCCUGGAUCUGCAUCCAGGAGCCGGAAAAACCAGGAGAGUUCUUCCUGAAAUAGUCCGUGAAGCCAUAAAAAAGAGACUCCGGACAGUGAUCUUGGCACCAACUAGGGUUGUCGCUGCUGAGAUGGAGGAAGCCUUGAGAGGACUUCCGGUGCGUUACAUGACAACAGCAGU
KF383118	AGCUAACUGUCCUGGAUCUGCAUCCAGGAGCCGGAAAAACCAGGAGAGUUCUUCCUGAAAUAGUCCGUGAAGCCAUAAAAAAGAGACUCCGGACAGUGAUCUUGGCACCAACUCCAGUUGAGAGAUCAUACUCAAGGUGGUCCUUGUGGCCAUCUGUGGCACGUACCCAGACAACAGCAGU
KF383117	AGCUAACAGUCUUGGAUCUGCAUCCAGGAGCCGGAAAAACCAGGAGGGUUCUUCCUGAAAUAGUCCGUGAAGCCAUAAAAAAGAGACUCCGGACAGUGAUCUUGGCACCAACUAGGGUUGUCGCUGCUGAGAUGGAGGAGGCCUUGAGAGGACUUCCGGUGCGUUACAUGACAACAGCAGU
KF383116	AGCUAACAGUCUUGGAUCUGCAUCCAGGAGCCGGGAAAACCAGGAGGGUUCUUCCUGAAAUAGUCCGUGAAGCCAUAAAGAAGAGACUUCGCACAUUGAUCCUAGCACCAACCAGGGUCGUUGCUGCUGAGAUGGAGGAAGCCCUAAGAGGACUUC??GUGCGUUACAUGACAACAGCAGU
KF383115	AGCUAACUGUCCUGGACCUGCAUCCAGGAGCCGGAAAGACUAGGAGAGUUCUUCCUGAAAUAGUCCGUGAAGCCAUAAAAAAGAGACUCCGCACAGUGAUCUUGGCUCCAACCAGGGUUGUCGCUGCUGAGAUGGAGGAAGCCUUGAGAGGACUUC??GU?CGUUACAUGACAACAGCAGU
EU545988	AGCUAACUGUCUUAGACCUGCAUCCUGGAGCUGGGAAAACCAGGAGAGUUCUUCCUGAAAUAGUCCGUGAAGCCAUAAAAACAAGACUCCGUACUGUGAUCUUAGCUCCAACCAGGGUUGUCGCUGCUGAAAUGGAGGAAGCCCUUAGAGGGCUUCCAGUGCGUUAUAUGACAACAGCAGU
KU955589	AGCUAACUGUCUUAGACUUGCAUCCUGGAGCUGGGAAAACCAGGAGAGUUCUUCCUGAAAUAGUCCGUGAAGCCAUAAAAACAAGACUCCGUACUGUGAUCUUAGCUCCAACCAGGGUUGUCGCUGCCGAAAUGGAGGAAGCCCUUAGAGGGCUUCCAGUGCGUUAUAUGACAACAGCAGU
KU955590	AGCUAACUGUCUUAGACUUGCAUCCUGGAGCUGGGAAAACCAGGAGAGUUCUUCCUGAAAUAGUCCGUGAAGCCAUAAAAACAAGACUCCGUACUGUGAUCUUAGCUCCAACCAGGGUUGUCGCUGCUGAAAUGGAGGAGGCCCUUAGAGGGCUUCCAGUGCGUUAUAUGACAACAGCAGU
KU940228	AGCUAACUGUCUUAGACUUGCAUCCUGGAGCUGGGAAAACCAGGAGAGUUCUUCCUGAAAUAGUCCGUGAAGCCAUAAAAACAAGACUCCGUACUGUGAUCUUAGCUCCAACCAGGGUUGUCGCUGCUGAAAUGGAGGAAGCCCUUAGAGGGCUUCCAGUGCGUUAUAUGACAACAGCAGU
KU991811	AGCUAACUGUCUUAGACUUGCAUCCUGGAGCUGGGAAAACCAGGAGAGUUCUUCCUGAAAUAGUCCGUGAAGCCAUAAAAACAAGACUCCGUACUGUGAUCUUAGCUCCAACCAGGGUUGUCGCUGCUGAAAUGGAGGAAGCCCUUAGAGGGCUUCCAGUGCGUUAUAUGACAACAGCAGU
KU509998	AGCUAACUGUCUUAGACUUGCAUCCUGGAGCUGGGAAAACCAGGAGAGUUCUUCCUGAAAUAGUCCGUGAAGCCAUAAAAACAAGACUCCGUACUGUGAUCUUAGCUCCAACCAGGGUUGUCGCUGCUGAAAUGGAGGAAGCCCUUAGAGGGCUUCCAGUGCGUUAUAUGACAACAGCAGU
KX056898	AGCUAACUGUCUUAGACUUGCAUCCUGGAGCUGGGAAAACCAGGAGAGUUCUUCCUGAAAUAGUCCGUGAAGCCAUAAAAACAAGACUCCGUACUGUGAUCUUAGCUCCAACCAGGGUUGUCGCUGCUGAAAUGGAGGAGGCCCUUAGAGGGCUUCCAGUGCGUUAUAUGACAACAGCAGU
KX117076	AGCUAACUGUCUUAGACUUGCAUCCUGGAGCUGGGAAAACCAGGAGAGUUCUUCCUGAAAUAGUCCGUGAAGCCAUAAAAACAAGACUCCGUACUGUGAUCUUAGCUCCAACCAGGGUUGUCGCUGCCGAAAUGGAGGAAGCCCUUAGAGGGCUUCCAGUGCGUUAUAUGACAACAGCAGU
KX185891	AGCUAACUGUCUUAGACUUGCAUCCUGGAGCUGGGAAAACCAGGAGAGUUCUUCCUGAAAUAGUCCGUGAAGCCAUAAAAACAAGACUCCGUACUGUGAUCUUAGCUCCAACCAGGGUUGUCGCUGCCGAAAUGGAGGAAGCCCUUAGAGGGCUUCCAGUGCGUUAUAUGACAACAGCAGU
KX247646	AGCUAACUGUCUUAGACUUGCAUCCUGGAGCUGGGAAAACCAGGAGAGUUCUUCCUGAAAUAGUCCGUGAAGCCAUAAAAACAAGACUCCGUACUGUGAUCUUAGCUCCAACCAGGGUUGUCGCUGCUGAAAUGGAGGAAGCCCUUAGAGGGCUUCCAGUGCGUUAUAUGACAACAGCAGU
KX262887	AGCUAACUGUCUUAGACUUACAUCCUGGAGCUGGGAAAACCAGGAGAGUUCUUCCUGAAAUAGUCCGUGAAGCCAUAAAAACAAGACUCCGUACUGUGAUCUUAGCUCCAACCAGGGUUGUCGCUGCUGAAAUGGAGGAGGCCCUUAGAGGGCUUCCAGUGCGUUAUAUGACAACAGCAGU
KX280026	AGCUAACUGUCUUAGACUUGCAUCCUGGAGCUGGGAAAACCAGGAGAGUUCUUCCUGAAAUAGUCCGUGAAGCCAUAAAAACAAGACUCCGUACUGUGAUCUUAGCUCCAACCAGGGUUGUCGCUGCUGAAAUGGAGGAAGCCCUUAGAGGGCUUCCAGUGCGUUAUAUGACAACAGCAGU
KU758877	AGCUAACUGUCUUAGACUUGCAUCCUGGAGCUGGGAAAACCAGGAGAGUUCUUCCUGAAAUAGUCCGUGAAGCCAUAAAAACAAGACUCCGUACUGUGAUCUUAGCUCCAACCAGGGUUGUCGCUGCUGAAAUGGAGGAGGCCCUUAGAGGGCUUCCAGUGCGUUAUAUGACAACAGCAGU
KU866423	AGCUAACUGUCUUAGACUUGCAUCCUGGAGCUGGGAAAACCAGGAGAGUUCUUCCUGAAAUAGUCCGUGAAGCCAUAAAAACAAGACUCCGUACUGUGAUCUUAGCUCCAACCAGGGUUGUCGCUGCCGAAAUGGAGGAAGCCCUUAGAGGGCUUCCAGUGCGUUAUAUGACAACAGCAGU
KX377336	AGCUAACUGUCUUGGAUCUGCAUCCUGGAGCUGGGAAAACCAGGAGAGUUCUUCCUGAAAUAGUCCGUGAAGCCAUAAAAACAAGACUCCGCACGGUGAUCCUGGCUCCAACCAGGGUUGUCGCUGCUGAAAUGGAGGAAGCCCUUAGAGGGCUUCCAGUGCGUUACAUGACAACAGCAGU
KX377337	AGCUAACUGUCUUAGACUUGCAUCCUGGAGCUGGGAAAACCAGGAGAGUUCUUCCUGAAAUAGUCCGUGAAGCCAUAAAAACAAGACUCCGUACUGUGAUCUUAGCUCCAACCAGGGUUGUCGCUGCUGAAAUGGAGGAGGCCCUUAGAGGGCUUCCAGUGCGUUAUAUGACAACAGCAGU
KX369547	AGCUAACUGUCUUAGACUUGCAUCCUGGAGCUGGGAAAACCAGGAGAGUUCUUCCUGAAAUAGUCCGUGAAGCCAUAAAAACAAGACUCCGUACUGUGAUCUUAGCUCCAACCAGGGUUGUCGCUGCUGAAAUGGAGGAAGCCCUUAGAGGGCUUCCAGUGCGUUAUAUGACAACAGCAGU
KX548902	AGCUAACUGUCUUAGACUUGCAUCCUGGAGCUGGGAAAACCAGGAGAGUUCUUCCUGAAAUAGUCCGUGAAGCCAUAAAAACAAGACUCCGUACUGUGAUCUUAGCUCCAACCAGGGUUGUCGCUGCUGAAAUGGAGGAAGCCCUUAGAGGGCUUCCAGUGCGUUAUAUGACAACAGCAGU
KX601166	AGCUAACAGUCUUGGAUCUGCAUCCAGGAGCCGGGAAAACCAGGAGGGUUCUUCCUGAAAUAGUCCGUGAAGCCAUAAAGAAGAGACUUCGCACAGUGAUCCUAGCACCAACCAGGGUCGUUGCCGCUGAGAUGGAGGAAGCCCUAAGAGGACUGCCGGUGCGUUACAUGACAACAGCAGU
KX601167	AGCUAACUGUCUUGGAUCUGCAUCCUGGAGCUGGGAAAACCAGGAGAGUUCUUCCUGAAAUAGUCCGUGAAGCCAUAAAAACAAGACUCCGCACGGUGAUCCUGGCUCCAACCAGGGUUGUCGCUGCUGAAAUGGAGGAAGCCCUUAGAGGGCUUCCAGUGCGUUACAUGACAACAGCAGU
KX601168	AGCUAACUGUCUUAGACUUGCAUCCUGGAGCUGGGAAAACCAGGAGAGUUCUUCCUGAAAUAGUCCGUGAAGCCAUAAAAACAAGACUCCGUACUGUGAUCUUAGCUCCAACCAGGGUUGUCGCUGCUGAAAUGGAGGAGGCCCUUAGAGGGCUUCCAGUGCGUUAUAUGACAACAGCAGU
KX766028	AGCUAACUGUCUUAGACUUGCAUCCUGGAGCUGGGAAAACCAGGAGAGUUCUUCCUGAAAUAGUCCGUGAAGCCAUAAAAACAAGACUCCGUACUGUGAUCUUAGCUCCAACCAGGGUUGUCGCUGCUGAAAUGGAGGAGGCCCUUAGAGGGCUUCCAGUGCGUUAUAUGACAACAGCAGU
KX766029	AGCUAACUGUCUUAGACUUACAUCCUGGAGCUGGGAAAACCAGGAGAGUUCUUCCUGAAAUAGUCCGUGAAGCCAUAAAAACAAGACUCCGUACUGUGAUCUUAGCUCCAACCAGGGUUGUCGCUGCUGAAAUGGAGGAGGCCCUUAGAGGGCUUCCAGUGCGUUAUAUGACAACAGCAGU
KU940224	AGCUAACUGUCUUAGACUUGCAUCCUGGAGCUGGGAAAACCAGGAGAGUUCUUCCUGAAAUAGUCCGUGAAGCCAUAAAAACAAGACUCCGUACUGUGAUCUUAGCUCCAACCAGGGUUGUCGCUGCUGAAAUGGAGGAAGCCCUUAGAGGGCUUCCAGUGCGUUAUAUGACAACAGCAGU

	5555555555555555555555555555555555555555555555555555555555555555555555555555555555555555555555555555555555555555555555555555555555555555555555555555555555555555555555555555555555555
	2222222222222222222222222222222222222222222222222233333333333333333333333333333333333333333333333333333333333333333333333333333333333333333333333333333334444444444444444444444444444444
	5555555555666666666677777777778888888888999999999900000000001111111111222222222233333333334444444444555555555566666666667777777777888888888899999999990000000000111111111122222222223
Sequence	0123456789012345678901234567890123456789012345678901234567890123456789012345678901234567890123456789012345678901234567890123456789012345678901234567890123456789012345678901234567890
NC_012532	CAACGUCACCCAUUCUGGGACAGAAAUCGUUGAUUUGAUGUGCCAUGCCACUUUCACUUCACGCUUACUACAACCCAUCAGAGUCCCUAAUUACAAUCUCAACAUCAUGGAUGAAGCCCACUUCACAGACCCCUCAAGUAUAGCUGCAAGAGGAUACAUAUCAACAAGGGUUGAAAUGGGC
KU922960	CAAUGUCACCCACUCUGGAACAGAAAUCGUCGACUUAAUGUGCCAUGCCACCUUCACUUCACGUCUACUACAGCCAAUCAGAGUCCCCAACUAUAAUCUGUAUAUUAUGGAUGAGGCCCACUUCACAGAUCCCUCAAGUAUAGCAGCAAGAGGAUACAUUUCAACAAGGGUUGAGAUGGGC
KU922923	CAAUGUCACCCACUCUGGAACAGAAAUCGUCGACUUAAUGUGCCAUGCCACCUUCACUUCACGUCUACUACAGCCAAUCAGAGUCCCCAACUAUAAUCUGUAUAUUAUGGAUGAGGCCCACUUCACAGAUCCCUCAAGUAUAGCAGCAAGAGGAUACAUUUCAACAAGGGUUGAGAUGGGC
KU820898	CAAUGUCACCCACUCUGGAACAGAAAUCGUCGACUUAAUGUGCCAUGCCACCUUCACUUCACGUCUACUACAGCCAAUUAGAGUCCCCAACUAUAAUCUGUAUAUUAUGGAUGAGGCCCACUUCACAGAUCCCUCAAGUAUAGCAGCAAGAGGAUACAUUUCAACAAGGGUUGAGAUGGGC
KU321639	CAAUGUCACCCACUCUGGAACAGAAAUCGUCGACUUAAUGUGCCAUGCCACCUUCACUUCACGUCUACUACAGCCAAUCAGAGUCCCCAACUAUAAUCUGUAUAUUAUGGAUGAGGCCCACUUCACAGAUCCCUCAAGUAUAGCAGCAAGAGGAUACAUUUCAACAAGGGUUGAGAUGGGC
KU820899	CAAUGUCACCCACUCUGGAACAGAAAUCGUCGACUUAAUGUGCCAUGCCACCUUCACUUCACGUCUACUACAGCCAAUCAGAGUCCCCAACUAUAAUCUGUAUAUUAUGGAUGAGGCCCACUUCACAGAUCCCUCAAGUAUAGCAGCAAGAGGAUACAUUUCAACAAGGGUUGAGAUGGGC
KU853012	CAAUGUCACCCAUUCUGGAACAGAAAUCGUCGACUUAAUGUGCCAUGCCACCUUCACUUCACGUCUACUACAGCCAAUCAGAGUCCCCAACUAUAAUCUGUACAUUAUGGAUGAGGCCCACUUCACAGAUCCCUCAAGUAUAGCAGCAAGAGGAUACAUUUCAACAAGGGUUGAGAUGGGC
KU729217	CAAUGUCACCCACUCUGGAACAGAAAUCGUCGACUUAAUGUGCCAUGCCACCUUCACUUCACGCCUACUACAGCCAAUCAGAGUCCCCAACUAUAAUCUGUAUAUUAUGGAUGAGGCCCACUUCACAGAUCCCUCAAGUAUAGCAGCAAGAGGAUACAUUUCAACAAGGGUUGAGAUGGGC
KU729218	CAAUGUCACCCACUCUGGAACAGAAAUCGUCGACUUAAUGUGCCAUGCCACCUUCACUUCACGUCUACUACAGCCAAUCAGAGUCCCCAAUUAUAAUCUGUAUAUUAUGGAUGAGGCCCACUUCACAGAUCCCUCAAGUAUAGCAGCAAGAGGAUACAUUUCAACAAGGGUUGAGAUGGGC
KU761564	CAAUGUCACCCACUCUGGAACAGAAAUCGUCGACUUAAUGUGCCAUGCCACCUUCACUUCACGUCUACUACAGCCAAUUAGAGUCCCCAACUAUAAUCUGUAUAUUAUGGAUGAGGCCCACUUCACAGAUCCCUCAAGUAUAGCAGCAAGAGGAUACAUUUCAACAAGGGUUGAGAUGGGC
KU740184	CAAUGUCACCCACUCUGGAACAGAAAUCGUCGACUUAAUGUGCCAUGCCACCUUCACUUCACGUCUACUACAGCCAAUUAGAGUCCCCAACUAUAAUCUGUAUAUUAUGGAUGAGGCCCACUUCACAGAUCCCUCAAGUAUAGCAGCAAGAGGAUACAUUUCAACAAGGGUUGAGAUGGGC
KU744693	CAAUGUCACCCACUCUGGAACAGAAAUCGUCGACUUAAUGUGCCAUGCCACCUUCACUUCACGUCUACUACAGCCAAUUAGAGUCCCCAACUAUAAUCUGUAUAUUAUGGAUGAGGCCCACUUCACAGAUCCCUCAAGUAUAGCAGCAAGAGGAUACAUUUCAACAAGGGUUGAGAUGGGC
KU497555	CAAUGUCACCCACUCUGGAACAGAAAUCGUCGACUUAAUGUGCCAUGCCACCUUCACUUCACGUCUACUACAGCCAAUCAGAGUCCCCAACUAUAAUCUGUAUAUUAUGGAUGAGGCCCACUUCACAGAUCCCUCAAGCAUAGCAGCAAGAGGAUACAUUUCAACAAGGGUUGAGAUGGGC
KU527068	CAAUGUCACCCACUCUGGAACAGAAAUCGUCGACUUAAUGUGCCAUGCCACCUUCACUUCACGUCUACUACAGCCAAUCAGAGUCCCCAACUAUAAUCUGUAUAUUAUGGAUGAGGCCCACUUCACAGAUCCCUCAAGUAUAGCAGCAAGAGGAUACAUUUCAACAAGGGUUGAGAUGGGC
KU501217	CAAUGUCACCCACUCUGGAACAGAAAUCGUCGACUUAAUGUGCCAUGCCACCUUCACUUCACGUCUACUACAGCCAAUCAGAGUCCCCAACUAUAAUCUGUAUAUUAUGGAUGAGGCCCACUUCACAGAUCCCUCAAGUAUAGCAGCAAGAGGAUACAUUUCAACAAGGGUUGAGAUGGGC
KU501216	CAAUGUCACCCACUCUGGAACAGAAAUCGUCGACUUAAUGUGCCAUGCCACCUUCACUUCACGUCUACUACAGCCAAUCAGAGUCCCCAACUAUAAUCUGUAUAUUAUGGAUGAGGCCCACUUCACAGAUCCCUCAAGUAUAGCAGCAAGAGGAUACAUUUCAACAAGGGUUGAGAUGGGC
KU501215	CAAUGUCACCCACUCUGGAACAGAAAUCGUCGACUUAAUGUGCCAUGCCACCUUCACUUCACGUCUACUACAGCCAAUCAGAGUCCCCAACUAUAAUCUGUAUAUUAUGGAUGAGGCCCACUUCACAGAUCCCUCAAGUAUAGCAGCAAGAGGAUACAUUUCAACAAGGGUUGAGAUGGGC
KU365780	CAAUGUCACCCACUCUGGAACAGAAAUCGUCGACUUAAUGUGCCAUGCCACCUUCACUUCACGUCUACUACAGCCAAUCAGAGUCCCCAACUAUAAUCUGUAUAUUAUGGAUGAGGCCCACUUCACAGAUCCCUCAAGUAUAGCAGCAAGAGGAUACAUUUCAACAAGGGUUGAGAUGGGC
KU365779	CAAUGUCACCCACUCUGGAACAGAAAUCGUCGACUUAAUGUGCCAUGCCACCUUCACUUCACGUCUACUACAGCCAAUCAGAGUCCCCAACUAUAAUCUGUAUAUUAUGGAUGAGGCCCACUUCACAGAUCCCUCAAGUAUAGCAGCAAGAGGAUACAUUUCAACAAGGGUUGAGAUGGGC
KU707826	CAAUGUCACCCACUCUGGAACAGAAAUCGUCGACUUAAUGUGCCAUGCCACCUUCACUUCACGUCUACUACAGCCAAUCAGAGUCCCCAACUAUAAUCUGUAUAUUAUGGAUGAGGCCCACUUCACAGAUCCCUCAAGUAUAGCAGCAAGAGGAUACAUUUCAACAAGGGUUGAGAUGGGC
KU365778	CAAUGUCACCCACUCUGGAACAGAAAUCGUCGACUUAAUGUGCCAUGCCACCUUCACUUCACGUCUACUACAGCCAAUCAGAGUCCCCAACUAUAAUCUGUAUAUUAUGGAUGAGGCCCACUUCACAGAUCCCUCAAGUAUAGCAGCAAGAGGAUACAUUUCAACAAGGGUUGAGAUGGGC
KU365777	CAAUGUCACCCACUCUGGAACAGAAAUCGUCGACUUAAUGUGCCAUGCCACCUUCACUUCACGUCUACUACAGCCAAUCAGAGUCCCCAACUAUAAUCUGUAUAUUAUGGAUGAGGCCCACUUCACAGAUCCCUCAAGUAUAGCAGCAAGAGGAUACAUUUCAACAAGGGUUGAGAUGGGC
KU312312	CAAUGUCACCCACUCUGGAACAGAAAUCGUCGACUUAAUGUGCCAUGCCACCUUCACUUCGCGUCUACUACAGCCAAUCAGAGUCCCCAACUAUAAUCUGUAUAUUAUGGAUGAGGCCCACUUCACAGAUCCCUCAAGUAUAGCAGCAAGAGGAUACAUUUCAACAAGGGUUGAGAUGGGC
KF268950	UAACGUCACCCACUCUGGGACAGAAAUCGUUGAUUUGAUGUGCCAUGCCACCUUCACCUCACGCCUAUUACAACCCAUCAGAGUCCCUAAUUACAAUCUCUACAUCAUGGAUGAAGCCCAUUUCACAGACCCCUCAAGUAUAGCUGCAAGAGGAUACAUAUCAACAAGGGUUGAAAUGGGC
KF268949	UAACGUCACCCACUCUGGGACAGAAAUCGUUGAUUUGAUGUGCCAUGCCACCUUCACUUCACGCUUAUUACAACCCAUCAGAGUCCCUAAUUACAAUCUCUACAUCAUGGAUGAAGCCCAUUUCACAGACCCCUCAAGUAUAGCUGCAAGAGGAUACAUAUCAACAAGGGUUGAAAUGGGC
KF268948	UAACGUCACCCACUCUGGGACAGAAAUCGUUGAUUUGAUGUGCCAUGCCACCUUCACCUCACGCCUAUUACAACCCAUCAGAGUCCCUAAUUACAAUCUCUACAUCAUGGAUGAAGCCCAUUUCACAGACCCCUCAAGUAUAGCUGCAAGAGGAUACAUAUCAACAAGGGUUGAAAUGGGC
KJ776791	CAAUGUCACCCACUCUGGAACAGAAAUCGUCGACUUAAUGUGCCAUGCCACCUUCACUUCACGUCUACUACAGCCAAUCAGAGUCCCCAACUAUAAUCUGUAUAUUAUGGAUGAGGCCCACUUCACAGAUCCCUCAAGUAUAGCAGCAAGAGGAUACAUUUCAACAAGGGUUGAGAUGGGC
KF383119	CAACGUCACCCAUUCUGGGACAGAAAUCGUUGAUUUGAUGUGCCAUGCCACUUUCACUUCACGCUUACUACAACCCAUCAGAGUCCCUAAUUACAAUCUCAACAUCAUGGAUGAAGCCCACUUCACAGACCCCUCAAGUAUAGCUGCAAGAGGAUACAUAUCAACAAGGGUUGAAAUGGGC
KF383118	CAACGUCACCCAUUCUGGGACAGAAAUCGUUGAUUUGAUGUGCCAUGCCACUUUCACUUCACGCUUACUACAACCCAUCAGAGUCCCUAAUUACAAUCUCAACAUCAUGGAUGAAGCCCACUUCACAGACCCCUCAAGUAUAGCUGCAAGAGGAUACAUAUCAACAAGGGUUGAAAUGGGC
KF383117	CAACGUCACCCAUUCUGGGACAGAAAUCGUUGAUUUGAUGUGCCAUGCCACUUUCACUUCACGCUUACUACAACCCAUCAGAGUCCCUAAUUACAAUCUCAACAUCAUGGAUGAAGCCCACUUCACAGACCCCUCAAGUAUAGCUGCAAGAGGAUACAUAUCAACAAGGGUUGAAAUGGGC
KF383116	CAACGUCACCCAUUCUGGGACAGAAAUCGUUGAUUUGAUGUGCCAUGCCACUUUCACUUCACGCUUACUACAACCCAUCAGAGUCCCUAAUUACAAUCUCAACAUCAUGGAUGAAGCCCACUUCACAGACCCCUCAAGUAUAGCUGCAAGAGGAUACAUAUCAACAAGGGUUGAAAUGGGC
KF383115	CAACGUCACCCAUUCUGGGACAGAAAUCGUUGAUUUGAUGUGCCAUGCCACUUUCACUUCACGCUUACUACAACCCAUCAGAGUCCCUAAUUACAAUCUCAACAUCAUGGAUGAAGCCCACUUCACAGACCCCUCAAGUAUAGCUGCAAGAGGAUACAUAUCAACAAGGGUUGAAAUGGGC
EU545988	CAAUGUCACCCAUUCUGGGACAGAAAUUGUUGACUUAAUGUGCCAUGCCACCUUCACUUCACGUCUACUACAACCAAUCAGAGUCCCCAACUAUAAUCUGUAUAUUAUGGACGAGGCCCACUUCACAGAUCCCUCAAGUAUAGCAGCAAGAGGAUACAUUUCAACAAGGGUUGAGAUGGGC
KU955589	CAAUGUCACCCACUCUGGAACAGAAAUCGUCGACUUAAUGUGCCAUGCCACCUUCACUUCACGUCUACUACAGCCAAUCAGAGUCCCCAACUAUAAUCUGUAUAUUAUGGAUGAGGCCCACUUCACAGAUCCCUCAAGUAUAGCAGCAAGAGGAUACAUUUCAACAAGGGUUGAGAUGGGC
KU955590	CAAUGUCACCCACUCUGGAACAGAAAUCGUCGACUUAAUGUGCCAUGCCACCUUCACUUCACGUCUACUACAGCCAAUUAGAGUCCCCAACUAUAAUCUGUAUAUUAUGGAUGAGGCCCACUUUACAGAUCCCUCAAGUAUAGCAGCAAGAGGAUACAUUUCAACAAGGGUUGAGAUGGGC
KU940228	CAAUGUCACCCACUCUGGAACAGAAAUCGUCGACUUAAUGUGCCAUGCCACCUUCACUUCACGUCUACUACAGCCAAUCAGAGUCCCCAAUUAUAAUCUGUAUAUUAUGGAUGAGGCCCACUUCACAGAUCCCUCAAGUAUAGCAGCAAGAGGAUACAUUUCAACAAGGGUUGAGAUGGGC
KU991811	CAAUGUCACCCACUCUGGAACAGAAAUCGUCGACUUAAUGUGCCAUGCCACCUUCACUUCACGUCUACUACAGCCAAUCAGAGUCCCCAACUAUAAUCUGUAUAUUAUGGAUGAGGCCCACUUCACAGAUCCCUCAAGUAUAGCAGCAAGAGGAUACAUUUCAACAAGGGUUGAGAUGGGC

```
KU509998    CAAUGUCACCCACUCUGGAACAGAAAUCGUCGACUUAAUGUGCCAUGCCACCUUCACUUCACGUCUACUACAGCCAAUCAGAGUCCCCAACUAUAAUCUGUAUAUUAUGGAUGAGGCCCACUUCACAGAUCCCUCAAGUAUAGCAGCAAGAGGAUACAUUUCAACAAGGGUUGAGAUGGGC
KX056898    CAAUGUCACCCACUCUGGAACAGAAAUCGUCGACUUAAUGUGCCAUGCCACCUUCACUUCACGUCUACUACAGCCAAUUAGAGUCCCCAACUAUAAUCUGUAUAUUAUGGAUGAGGCCCACUUUACAGAUCCCUCAAGUAUAGCAGCAAGAGGAUACAUUUCAACAAGGGUUGAGAUGGGC
KX117076    CAAUGUCACCCACUCUGGAACAGAAAUCGUCGACUUAAUGUGCCAUGCCACCUUCACUUCACGUCUACUACAGCCAAUCAGAGUCCCCAACUAUAAUCUGUAUAUUAUGGAUGAGGCCCACUUCACAGAUCCCUCAAGUAUAGCAGCAAGAGGAUACAUUUCAACAAGGGUUGAGAUGGGC
KX185891    CAAUGUCACCCACUCUGGAACAGAAAUCGUCGACUUAAUGUGCCAUGCCACCUUCACUUCACGUCUACUACAGCCAAUCAGAGUCCCCAACUAUAAUCUGUAUAUUAUGGAUGAGGCCCACUUCACAGAUCCCUCAAGUAUAGCAGCAAGAGGAUACAUUUCAACAAGGGUUGAGAUGGGC
KX247646    CAAUGUCACCCACUCUGGAACAGAAAUCGUCGACUUAAUGUGCCAUGCCACCUUCACUUCACGUCUACUACAGCCAAUCAGAGUCCCCAACUAUAAUCUGUAUAUUAUGGAUGAGGCCCACUUCACAGAUCCCUCAAGUAUAGCAGCAAGAGGAUACAUUUCAACAAGGGUUGAGAUGGGC
KX262887    CAAUGUCACCCACUCUGGAACAGAAAUCGUCGACUUAAUGUGCCAUGCCACCUUCACUUCACGUCUACUACAGCCAAUCAGAGUCCCCAACUAUAAUCUGUAUAUUAUGGAUGAGGCCCACUUCACAGAUCCCUCAAGUAUAGCAGCAAGAGGAUACAUUUCAACAAGGGUUGAGAUGGGC
KX280026    CAAUGUCACCCACUCUGGAACAGAAAUCGUCGACUUAAUGUGCCAUGCCACCUUCACUUCACGUCUACUACAGCCAAUCAGGGUCCCCAACUAUAAUCUGUAUAUUAUGGAUGAGGCCCACUUCACAGAUCCCUCAAGUAUAGCAGCAAGAGGAUACAUUUCAACAAGGGUUGAGAUGGGC
KU758877    CAAUGUCACCCACUCUGGAACAGAAAUCGUCGACUUAAUGUGCCAUGCCACCUUCACUUCACGUCUACUACAGCCAAUCAGAGUCCCCAACUAUAAUCUGUAUAUUAUGGAUGAGGCCCACUUCACAGAUCCCUCAAGUAUAGCAGCAAGAGGAUACAUUUCAACAAGGGUUGAGAUGGGC
KU866423    CAAUGUCACCCACUCUGGAACAGAAAUCGUCGACUUAAUGUGCCAUGCCACCUUCACUUCACGUCUACUACAGCCAAUCAGAGUCCCCAACUAUAAUCUGUAUAUUAUGGAUGAGGCCCACUUCACAGAUCCCUCAAGUAUAGCAGCAAGAGGAUACAUUUCAACAAGGGUUGAGAUGGGC
KX377336    UAAUGUCACCCACUCUGGGACAGAAAUCGUUGAUUUAAUGUGCCAUGCCACCUUCACUUCACGCCUACUACAACCCAUUAGAGUCCCCAACUACAAUCUUUACAUUAUGGAUGAGGCCCACUUCACAGAUCCCUCAAGUAUAGCAGCAAGAGGAUACAUAUCAACAAGGGUUGAGAUGGGC
KX377337    CAAUGUCACCCACUCUGGAACAGAAAUCGUCGACUUAAUGUGCCAUGCCACCUUCACUUCACGUCUACUACAGCCAAUCAGAGUCCCCAACUAUAAUCUGUAUAUUAUGGAUGAGGCCCACUUCACAGAUCCCUCAAGUAUAGCAGCAAGAGGAUACAUUUCAACAAGGGUUGAGAUGGGC
KX369547    CAAUGUCACCCACUCUGGAACAGAAAUCGUCGACUUAAUGUGCCAUGCCACCUUCACUUCACGUCUACUACAGCCAAUCAGAGUCCCCAACUAUAAUCUGUAUAUUAUGGAUGAGGCCCACUUCACAGAUCCCUCAAGUAUAGCAGCAAGAGGAUACAUUUCAACAAGGGUUGAGAUGGGC
KX548902    CAAUGUCACCCACUCUGGAACAGAAAUCGUCGACUUAAUGUGCCAUGCCACCUUCACUUCACGUCUACUACAGCCAAUCAGAGUCCCCCACGAUAAUCUGUAUAUUAUGGAUGAGGCCCACUUCACAGAUCCCUCAAGUAUAGCAGCAAGAGGAUACAUUUCAACAAGGGUUGAGAUGGGC
KX601166    CAACGUCACCCACUCUGGGACAGAAAUCGUCGAUUUGAUGUGCCAUGCCACCUUCACUUCACGCCUACUACAACCCAUCAGAGUCCCCAACUACAACCUUUACAUCAUGGAUGAAGCUCAUUUCACAGAUCCCUCAAGCAUAGCUGCACGAGGAUAUAUAUCAACAAGGGUUGAAAUGGGC
KX601167    UAAUGUCACCCACUCUGGGACAGAAAUCGUUGAUUUAAUGUGCCAUGCCACCUUCACUUCACGCCUACUACAACCCAUUAGAGUCCCCAACUACAAUCUUUACAUUAUGGAUGAGGCCCACUUCACAGAUCCCUCAAGUAUAGCAGCAAGAGGAUACAUAUCAACAAGGGUUGAGAUGGGC
KX601168    CAAUGUCACCCACUCUGGAACAGAAAUCGUCGACUUAAUGUGCCAUGCCACCUUCACUUCACGUCUACUACAGCCAAUCAGAGUCCCCAACUAUAAUCUGUAUAUUAUGGAUGAGGCCCACUUCACAGAUCCCUCAAGUAUAGCAGCAAGAGGAUACAUUUCAACAAGGGUUGAGAUGGGC
KX766028    CAAUGUCACCCACUCUGGAACAGAAAUCGUCGACUUAAUGUGCCAUGCCACCUUCACUUCACGUCUACUACAGCCAAUUAGAGUCCCCAACUAUAAUCUGUAUAUUAUGGAUGAGGCCCACUUCACAGAUCCCUCAAGUAUAGCAGCAAGAGGAUACAUUUCAACAAGGGUUGAGAUGGGC
KX766029    CAAUGUCACCCACUCUGGAACAGAAAUCGUCGACUUAAUGUGCCAUGCCACCUUCACUUCACGUCUACUACAGCCAAUCAGAGUCCCCAACUAUAAUCUGUAUAUUAUGGAUGAGGCCCACUUCACAGAUCCCUCAAGUAUAGCAGCAAGAGGAUACAUUUCAACAAGGGUUGAGAUGGGC
KU940224    CAAUGUCACCCACUCUGGAACAGAAAUCGUCGACUUAAUGUGCCAUGCCACCUUCACUUCACGUCUACUACAGCCAAUCAGAGUCCCCAAUUAUAAUCUGUAUAUUAUGGAUGAGGCCCACUUCACAGAUCCCUCAAGUAUAGCAGCAAGAGGAUACAUUUCAACAAGGGUUGAGAUGGGC


            5555555555555555555555555555555555555555555555555555555555555555555555555555555555555555555555555555555555555555555555555555555555555555555555555555555555555555555555555555555
            4444444444444444444444444444444444444444444444444444444444444444444445555555555555555555555555555555555555555555555555555555555555555555555555555555555555555555555666666666666
            3333333334444444444555555555566666666667777777777888888888899999999990000000000111111111122222222223333333333444444444455555555556666666666777777777788888888889999999999000000000011
Sequence    1234567890123456789012345678901234567890123456789012345678901234567890123456789012345678901234567890123456789012345678901234567890123456789012345678901234567890123456789012345678901
-------------------------------------------------------------------------------------------------------------------------------------------------------------------------------------------------
NC_012532   GAGGCGGCUGCCAUUUUUAUGACUGCCACACCACCAGGAACCCGUGAUGCGUUUCCUGACUCUAACUCACCAAUCAUGGACACAGAAGUGGAAGUCCCAGAGAGAGCCUGGAGCUCAGGCUUUGAUUGGGUGACAGACCAUUCUGGGAAAACAGUUUGGUUCGUUCCAAGCGUGAGAAACG
KU922960    GAGGCGGCUGCCAUCUUCAUGACCGCCACGCCACCAGGAACCCGUGACGCAUUUCCGGACUCCAACUCACCAAUUAUGGACACCGAAGUGGAAGUCCCAGAGAGAGCCUGGAGCUCAGGCUUUGAUUGGGUGACGGAUCAUUCUGGAAAAACAGUUUGGUUUGUUCCAAGCGUGAGGAACG
KU922923    GAGGCGGCUGCCAUCUUCAUGACCGCCACGCCACCAGGAACCCGUGACGCAUUUCCGGACUCCAACUCACCAAUUAUGGACACCGAAGUGGAAGUCCCAGAGAGAGCCUGGAGCUCAGGCUUUGAUUGGGUGACGGAUCAUUCUGGAAAAACAGUUUGGUUUGUUCCAAGCGUGAGGAACG
KU820898    GAGGCGGCUGCCAUCUUCAUGACCGCCACGCCACCAGGAACCCGUGACGCAUUUCCGGACUCCAACUCACCAAUUAUGGACACCGAAGUGGAAGUCCCAGAGAGAGCCUGGAGCUCAGGCUUUGAUUGGGUGACGGAUCAUUCUGGAAAAACAGUUUGGUUUGUUCCAAGCGUGAGGAACG
KU321639    GAGGCGGCUGCCAUCUUCAUGACCGCCACGCCACCAGGAACCCGUGACGCAUUUCCGGACUCCAACUCACCAAUUAUGGACACCGAAGUGGAAGUCCCAGAGAGAGCCUGGAGCUCAGGCUUUGAUUGGGUGACGGAUUAUUCUGGAAAAACAGUUUGGUUUGUUCCAAGCGUGAGGAACG
KU820899    GAGGCGGCUGCCAUCUUCAUGACCGCCACGCCACCAGGAACCCGUGACGCAUUUCCGGACUCCAACUCACCAAUUAUGGACACCGAAGUGGAAGUCCCAGAGAGAGCCUGGAGCUCAGGCUUUGAUUGGGUGACGGAUCAUUCUGGAAAAACAGUCUGGUUUGUUCCAAGCGUGAGGAACG
KU853012    GAGGCGGCUGCCAUCUUCAUGACCGCCACGCCACCAGGAACCCGUGACGCAUUUCCGGACUCCAACUCACCAAUUAUGGACACCGAAGUGGAAGUCCCAGAGAGAGCCUGGAGCUCAGGCUUUGAUUGGGUGACGGAUCAUUCUGGAAAAACAGUUUGGUUUGUUCCAAGCGUGAGGAACG
KU729217    GAGGCGGCUGCCAUCUUCAUGACCGCCACGCCACCAGGAACCCGUGACGCAUUUCCGGACUCCAACUCACCAAUUAUGGACACCGAAGUGGAAGUCCCAGAGAGAGCCUGGAGCUCAGGCUUUGAUUGGGUGACGGAUCAUUCUGGAAAAACAGUUUGGUUUGUUCCAAGCGUGAGGAACG
KU729218    GAGGCGGCUGCCAUCUUCAUGACCGCCACGCCACCAGGAACCCGUGACGCAUUUCCGGACUCCAACUCACCAAUUACGGACACCGAAGUGGAAGUCCCAGAGAGAGCCUGGAGCUCAGGCUUUGAUUGGGUGACGGAUCAUUCUGGAAAAACAGUUUGGUUUGUUCCAAGCGUGAGGAACG
KU761564    GAGGCGGCUGCCAUCUUCAUGACCGCCACGCCACCAGGAACCCGUGACGCAUUUCCGGACUCCAACUCACCAAUUAUGGACACCGAAGUGGAAGUCCCAGAGAGAGCCUGGAGCUCAGGCUUUGAUUGGGUGACGGAUCAUUCUGGAAAAACAGUUUGGUUUGUUCCAAGCGUGAGGAACG
KU740184    GAGGCGGCUGCCAUCUUCAUGACCGCCACGCCACCAGGAACCCGUGACGCAUUUCCGGACUCCAACUCACCAAUUAUGGACACCGAAGUGGAAGUCCCAGAGAGAGCCUGGAGCUCAGGCUUUGAUUGGGUGACGGAUCAUUCUGGAAAAACAGUUUGGUUUGUUCCAAGCGUGAGGAACG
KU744693    GAGGCGGCUGCCAUCUUCAUGACCGCCACGCCACCAGGAACCCGUGACGCAUUUCCGGACUCCAACUCACCAAUUAUGGACACCGAAGUGGAAGUCCCAGAGAGAGCCUGGAGCUCAGGCUUUGAUUGGGUGACGGAGUAUUCUGGAAAAACAGUUUGGUUUGUUCCACGCGUGAGGAACG
KU497555    GAGGCGGCUGCCAUCUUCAUGACCGCCACGCCACCAGGAACCCGUGACGCAUUUCCGGACUCCAACUCACCAAUUAUGGACACCGAAGUGGAAGUCCCAGAGAGAGCCUGGAGCUCAGGCUUUGAUUGGGUGACGGAUCAUUCUGGAAAAACAGUUUGGUUUGUUCCAAGCGUGAGGAACG
KU527068    GAGGCGGCUGCCAUCUUCAUGACCGCCACGCCACCAGGAACCCGUGACGCAUUUCCGGACUCCAACUCACCAAUUAUGGACACCGAAGUGGAAGUCCCAGAGAGAGCCUGGAGCUCAGGCUUUGAUUGGGUGACGGAUCAUUCUGGAAAAACAGUUUGGUUUGUUCCAAGCGUGAGGAACG
KU501217    GAGGCGGCUGCCAUCUUCAUGACCGCCACGCCACCAGGAACCCGUGACGCAUUUCCGGACUCCAACUCACCAAUUAUGGACACCGAAGUGGAAGUCCCAGAGAGAGCCUGGAGCUCAGGCUUUGAUUGGGUGACGGAUCAUUCUGGAAAAACAGUUUGGUUUGUUCCAAGCGUGAGGAACG
KU501216    GAGGCGGCUGCCAUCUUCAUGACCGCCACGCCACCAGGAACCCGUGACGCAUUUCCGGACUCCAACUCACCAAUUAUGGACACCGAAGUGGAAGUCCCAGAGAGAGCCUGGAGCUCAGGCUUUGAUUGGGUGACGGAUCAUUCUGGAAAAACAGUUUGGUUUGUUCCAAGCGUGAGGAACG
KU501215    GAGGCGGCUGCCAUCUUCAUGACCGCCACGCCACCAGGAACCCGUGACGCAUUUCCGGACUCCAACUCACCAAUUAUGGACACCGAAGUGGAAGUCCCAGAGAGAGCCUGGAGCUCAGGCUUUGAUUGGGUGACGGAUCAUUCUGGAAAAACAGUUUGGUUUGUUCCAAGCGUGAGGAACG
KU365780    GAGGCGGCUGCCAUCUUCAUGACCGCCACGCCACCAGGAACCCGUGACGCAUUUCCGGACUCCAACUCACCAAUUAUGGACACCGAAGUGGAAGUCCCAGAGAGAGCCUGGAGCUCAGGCUUUGAUUGGGUGACGGAUCAUUCUGGAAAAACAGUUUGGUUUGUUCCAAGCGUGAGGAACG
KU365779    GAGGCGGCUGCCAUCUUCAUGACCGCCACGCCACCAGGAACCCGUGACGCAUUUCCGGACUCCAACUCACCAAUUAUGGACACCGAAGUGGAAGUCCCAGAGAGAGCCUGGAGCUCAGGCUUUGAUUGGGUGACGGAUCAUUCUGGAAAAACAGUUUGGUUUGUUCCAAGCGUGAGGAACG
KU707826    GAGGCGGCUGCCAUCUUCAUGACCGCCACGCCACCAGGAACCCGUGACGCAUUUCCGGACUCCAACUCACCAAUUAUGGACACCGAAGUGGAAGUCCCAGAGAGAGCCUGGAGCUCAGGCUUUGAUUGGGUGACGGAUCAUUCUGGAAAAACAGUUUGGUUUGUUCCAAGCGUGAGGAACG
KU365778    GAGGCGGCUGCCAUCUUCAUGACCGCCACGCCACCAGGAACCCGUGACGCAUUUCCGGACUCCAACUCACCAAUUAUGGACACCGAAGUGGAAGUCCCAGAGAGAGCCUGGAGCUCAGGCUUUGAUUGGGUGACGGAUCAUUCUGGAAAAACAGUUUGGUUUGUUCCAAGCGUGAGGAACG
KU365777    GAGGCGGCUGCCAUCUUCAUGACCGCCACGCCACCAGGAACCCGUGACGCAUUUCCGGACUCCAACUCACCAAUUAUGGACACCGAAGUGGAAGUCCCAGAGAGAGCCUGGAGCUCAGGCUUUGAUUGGGUGACGGAUCAUUCUGGAAAAACAGUUUGGUUUGUUCCAAGCGUGAGGAACG
KU312312    GAGGCGGCCGCCAUCUUCAUGACCGCCACGCCACCAGGAACCCGUGACGCAUUUCCGGACUCCAACUCACCAAUUAUGGACACCGAAGUGGAAGUCCCAGAGAGAGCCUGGAGCUCAGGCUUUGAUUGGGUGACGGAUCAUUCUGGAAAAACAGUUUGGUUUGUUCCAAGCGUGAGGAACG
KF268950    GAGGCGGCUGCCAUCUUCAUGACAGCCACGCCACCAGGAACCCGAGAUGCAUUUCCAGACUCCAACUCACCAAUCAUGGACACAGAAGUGGAAGUCCCAGAGAGAGCCUGGAGCUCAGGCUUUGAUUGGGUGACAGACCAUUCUGGGAAAACAGUUUGGUUCGUUCCAAGUGUGAGAAAUG
KF268949    GAGGCGGCUGCCAUCUUUAUGACAGCCACGCCACCAGGAACCCGAGAUGCAUUUCCAGACUCCAACUCACCAAUCAUGGACACAGAAGUGGAAGUCCCAGAGAGAGCCUGGAGCUCAGGCUUUGAUUGGGUGACAGACCAUUCUGGAAAAACAGUUUGGUUUGUUCCAAGUGUGAGAAAUG
KF268948    GAGGCGGCUGCCAUCUUCAUGACAGCCACGCCACCAGGAACCCGAGAUGCAUUUCCAGACUCCAACUCACCAAUCAUGGACACAGAAGUGGAAGUCCCAGAGAGAGCCUGGAGCUCAGGCUUUGAUUGGGUGACAGACCAUUCUGGGAAAACAGUUUGGUUCGUUCCAAGUGUGAGAAAUG
KJ776791    GAGGCGGCUGCCAUCUUCAUGACCGCCACGCCACCAGGAACCCGUGACGCAUUUCCGGACUCCAACUCACCAAUUAUGGACACCGAAGUGGAAGUCCCAGAGAGAGCCUGGAGCUCAGGCUUUGAUUGGGUGACGGAUCAUUCUGGAAAAACAGUUUGGUUUGUUCCAAGCGUGAGGAACG
KF383119    GAGGCGGCUGCCAUUUUUAUGACUGCCACACCACCAGGAACCCGUGAUGCGUUUCCUGACUCUAACUCACCAAUCAUGGACACAGAAGUGGAAGUCCCAGAGAGAGCCUGGAGCUCAGGCUUUGAUUGGGUGACAGACCAUUCUGGGAAAACAGUUUGGUUCGUUCCAAGCGUGAGAAACG
KF383118    GAGGCGGCUGCCAUUUUUAUGACUGCCACACCACCAGGAACCCGUGAUGCGUUUCCUGACUCUAACUCACCAAUCAUGGACACAGAAGUGGAAGUCCCAGAGAGAGCCUGGAGCUCAGGCUUUGAUUGGGUGACAGACCAUUCUGGGAAAACAGUUUGGUUCGUUCCAAGCGUGAGAUCUG
KF383117    GAGGCGGCUGCCAUUUUUAUGACUGCCACACCACCAGGAACCCGUGAUGCGUUUCCUGACUCUAACUCACCAAUCAUGGACACAGAAGUGGAAGUCCCAGAGAGAGCCUGGAGCUCAGGCUUUGAUUGGGUGACAGACCAUUCUGGGAAAACAAUUUGGUUUGUUCCAAGUGUGAGAAACG
KF383116    GAGGCGGCUGCCAUUUUUAUGACUGCCACACCACCAGGAACCCGUGAUGCGUUUCCUGACUCUAACUCACCAAUCAUGGACACAGAAGUGGAAGUCCCAGAGAGAGCCUGGAGCUCAGGCUUUGACUGGGUGACGGACCAUUCUGGGAAAACAAUUUGGUUUGUUCCAAGUGUGAGAUCUG
KF383115    GAGGCGGCUGCCAUUUUUAUGACUGCCACACCACCAGGAACCCGUGAUGCGUUUCCUGACUCUAACUCACCAAUCAUGGACACAGAAGUGGAAGUCCCAGAGAGAGCCUGGAGCUCAGGCUUUGAUUGGGUGACAGACCAUUCUGGGAAAACAGUUUGGUUCGUUCCAAGUGUGAGAAAUG
EU545988    GAGGCGGCCGCCAUCUUCAUGACCGCCACCCCACCAGGAACCCGUGACGCAUUCCCGGACUCCAACUCACCAAUUAUGGACACCGAAGUGGAAGUCCCAGAGAGAGCCUGGAGCUCAGGCUUUGAUUGGGUGACGGAUCAUUCUGGAAAAACAGUUUGGUUUGUUCCAAGCGUGAGGAACG
KU955589    GAGGCGGCUGCCAUCUUCAUGACCGCCACGCCACCAGGAACCCGUGACGCAUUUCCGGACUCCAACUCACCAAUUAUGGACACCGAAGUGGAAGUCCCAGAGAGAGCCUGGAGCUCAGGCUUUGAUUGGGUGACGGAUCAUUCUGGAAAAACAGUCUGGUUUGUUCCAAGCGUGAGGAACG
KU955590    GAGGCGGCUGCCAUCUUCAUGACCGCCACGCCACCAGGAACCCGUGACGCAUUUCCGGACUCCAACUCACCAAUUAUGGACACCGAAGUGGAAGUCCCAGAGAGAGCCUGGAGCUCAGGCUUUGAUUGGGUGACGGAUCAUUCUGGAAAAACAGUUUGGUUUGUUCCAAGCGUGAGGAACG
KU940228    GAGGCGGCUGCCAUCUUCAUGACCGCCACGCCACCAGGAACCCGUGACGCAUUUCCGGACUCCAACUCACCAAUUAUGGACACCGAAGUGGAAGUCCCAGAGAGAGCCUGGAGCUCAGGCUUUGAUUGGGUGACGGAUCAUUCUGGAAAAACAGUUUGGUUUGUUCCAAGCGUGAGGAACG
KU991811    GAGGCGGCUGCCAUCUUCAUGACCGCCACGCCACCAGGAACCCGUGACGCAUUUCCGGACUCCAACUCACCAAUUAUGGACACCGAAGUGGAAGUCCCAGAGAGAGCCUGGAGCUCAGGCUUUGAUUGGGUGACGGAUCAUUCUGGAAAAACAGUUUGGUUUGUUCCAAGCGUGAGGAACG
KU509998    GAGGCGGCUGCCAUCUUCAUGACCGCCACGCCACCAGGAACCCGUGACGCAUUUCCGGACUCCAACUCACCAAUUAUGGACACCGAAGUGGAAGUCCCAGAGAGAGCCUGGAGCUCAGGCUUUGAUUGGGUGACGGAUUAUUCUGGAAAAACAGUUUGGUUUGUUCCAAGCGUGAGGAACG
KX056898    GAGGCGGCUGCCAUCUUCAUGACCGCCACGCCACCAGGAACCCGUGACGCAUUUCCGGACUCCAACUCACCAAUUAUGGACACCGAAGUGGAAGUCCCAGAGAGAGCCUGGAGCUCAGGCUUUGAUUGGGUGACGGAUCAUUCUGGAAAAACAGUUUGGUUUGUUCCAAGCGUGAGGAACG
KX117076    GAGGCGGCUGCCAUCUUCAUGACCGCCACGCCACCAGGAACCCGUGACGCAUUUCCGGACUCCAACUCACCAAUUAUGGACACCGAAGUGGAAGUCCCAGAGAGAGCCUGGAGCUCAGGCUUUGAUUGGGUGACGGAUCAUUCUGGAAAAACAGUCUGGUUUGUUCCAAGCGUGAGGAACG
KX185891    GAGGCGGCUGCCAUCUUCAUGACCGCCACGCCACCAGGAACCCGUGACGCAUUUCCGGACUCCAACUCACCAAUUAUGGACACCGAAGUGGAAGUCCCAGAGAGAGCCUGGAGCUCAGGCUUUGAUUGGGUGACGGAUCAUUCUGGAAAAACAGUCUGGUUUGUUCCAAGCGUGAGGAACG
KX247646    GAGGCGGCUGCCAUCUUCAUGACCGCCACGCCACCAGGAACCCGUGACGCAUUUCCGGACUCCAACUCACCAAUUAUGGACACCGAAGUGGAAGUCCCAGAGAGAGCCUGGAGCUCAGGCUUUGAUUGGGUGACGGAUCAUUCUGGAAAAACAGUUUGGUUUGUUCCAAGCGUGAGGAACG
KX262887    GAGGCGGCUGCCAUCUUCAUGACCGCCACGCCACCAGGAACCCGUGACGCAUUUCCGGACUCCAACUCACCAAUUAUGGACACCGAAGUGGAAGUCCCAGAGAGAGCCUGGAGCUCAGGCUUUGAUUGGGUGACGGAUCAUUCUGGAAAAACAGUUUGGUUUGUUCCAAGCGUGAGGAACG
KX280026    GAGGCGGCUGCCAUCUUCAUGACCGCCACGCCACCAGGAACCCGUGACGCAUUUCCGGACUCCAACUCACCAAUUAUGGACACCGAAGUGGAAGUCCCAGAGAGAGCCUGGAGCUCAGGCUUUGAUUGGGUGACGGAUCAUUCUGGAAAAACAGUUUGGUUUGUUCCAAGCGUGAGGAACG
KU758877    GAGGCGGCUGCCAUCUUCAUGACCGCCACGCCACCAGGAACCCGUGACGCAUUUCCGGACUCCAACUCACCAAUUAUGGACACCGAAGUGGAAGUCCCAGAGAGAGCCUGGAGCUCAGGCUUUGAUUGGGUGACGGAUCAUUCUGGAAAAACAGUUUGGUUUGUUCCAAGCGUGAGGAACG
KU866423    GAGGCGGCUGCCAUCUUCAUGACCGCCACGCCACCAGGAACCCGUGACGCAUUUCCGGACUCCAACUCACCAAUUAUGGACACCGAAGUGGAAGUCCCAGAGAGAGCCUGGAGCUCAGGCUUUGAUUGGGUGACGGAUCAUUCUGGAAAAACAGUCUGGUUUGUUCCAAGCGUGAGGAACG
KX377336    GAGGCGGCUGCCAUCUUCAUGACCGCCACACCACCAGGAACCCGCGACGCAUUUCCGGACUCUAACUCACCAAUCAUGGACACAGAAGUGGAAGUCCCAGAGAGAGCCUGGAGCUCAGGCUUUGAUUGGGUGACGGAUCAUUCUGGAAAAACAGUUUGGUUUGUUCCAAGCGUGAGGAACG
KX377337    GAGGCGGCUGCCAUCUUCAUGACCGCCACGCCACCAGGAACCCGUGACGCAUUUCCGGACUCCAACUCACCAAUUAUGGACACCGAAGUGGAAGUCCCAGAGAGAGCCUGGAGCUCAGGCUUUGAUUGGGUGACGGAUCAUUCUGGAAAAACAGUUUGGUUUGUUCCAAGCGUGAGGAACG
KX369547    GAGGCGGCUGCCAUCUUCAUGACCGCCACGCCACCAGGAACCCGUGACGCAUUUCCGGACUCCAACUCACCAAUUAUGGACACCGAAGUGGAAGUCCCAGAGAGAGCCUGGAGCUCAGGCUUUGAUUGGGUGACGGAUCAUUCUGGAAAAACAGUUUGGUUUGUUCCAAGCGUGAGGAACG
KX548902    GAGGCGGCUGCCAUCUUCAUGACCGCCACGCCACCAGGAACCCGUGACGCAUUUCCGGACUCCAACUCACCAAUUAUGGACACCGAAGUGGAAGUCCCAGAGAGAGCCUGGAGCUCAGGCUUUGAUUGGGUGACGGAUCAUUCUGGAAAAACAGUUUGGUUUGUUCCAAGCGUGAGGAACG
KX601166    GAGGCGGCUGCUAUCUUCAUGACUGCUACACCACCAGGAACCCGCGAUGCGUUUCCGGAUUCCAACUCACCAAUCAUGGACACAGAAGUGGAAGUUCCAGAGAGAGCCUGGAGCUCAGGCUUUGACUGGGUGACGGAUCAUUCUGGGAAAACAAUUUGGUUUGUUCCAAGUGUGAGAAACG
KX601167    GAGGCGGCUGCCAUCUUCAUGACCGCCACACCACCAGGAACCCGCGACGCAUUUCCGGACUCUAACUCACCAAUCAUGGACACAGAAGUGGAAGUCCCAGAGAGAGCCUGGAGCUCAGGCUUUGAUUGGGUGACGGAUCAUUCUGGAAAAACAGUUUGGUUUGUUCCAAGCGUGAGGAACG
```

```
KX601168    GAGGCGGCUGCCAUCUUCAUGACCGCCACGCCACCAGGAACCCGUGACGCAUUUCCGGACUCCAACUCACCAAUUAUGGACACCGAAGUGGAAGUCCCAGAGAGAGCCUGGAGCUCAGGCUUUGAUUGGGUGACGGAUCAUUCUGGAAAAACAGUUUGGUUUGUUCCAAGCGUGAGGAACG
KX766028    GAGGCGGCUGCCAUCUUCAUGACCGCCACGCCACCAGGAACCCGUGACGCAUUUCCGGACUCCAACUCACCAAUUAUGGACACCGAAGUGGAAGUCCCAGAGAGAGCCUGGAGCUCAGGCUUUGAUUGGGUGACGGAUCAUUCUGGAAAAACAGUUUGGUUUGUUCCAAGCGUGAGGAACG
KX766029    GAGGCGGCUGCCAUCUUCAUGACCGCCACGCCACCAGGAACCCGUGACGCAUUUCCGGACUCCAACUCACCAAUUAUGGACACCGAAGUGGAAGUCCCAGAGAGAGCCUGGAGCUCAGGCUUUGAUUGGGUGACGGAUCAUUCUGGGAAAACAGUUUGGUUUGUUCCAAGCGUGAGGAACG
KU940224    GAGGCGGCUGCCAUCUUCAUGACCGCCACGCCACCAGGAACCCGUGACGCAUUUCCGGACUCCAACUCACCAAUUAUGGACACCGAAGUGGAAGUCCCAGAGAGAGCCUGGAGCUCAGGCUUUGAUUGGGUGACGGAUCAUUCUGGAAAAACAGUUUGGUUUGUUCCAAGCGUGAGGAACG


            5555555555555555555555555555555555555555555555555555555555555555555555555555555555555555555555555555555555555555555555555555555555555555555555555555555555555555555555555555555
            6666666666666666666666666666666666666666666666666666666666666666666666666666666666666666777777777777777777777777777777777777777777777777777777777777777777777777777777777777777777777
            1111111122222222223333333333444444444455555555556666666666777777777788888888889999999999000000000011111111112222222222333333333344444444445555555555666666666677777777778888888888999
Sequence    2345678901234567890123456789012345678901234567890123456789012345678901234567890123456789012345678901234567890123456789012345678901234567890123456789012345678901234567890123456789012
-------------------------------------------------------------------------------------------------------------------------------------------------------------------------------------------------
NC_012532   GAAAUGAAAUCGCAGCCUGUCUGACAAAGGCUGGAAAGCGGGUCAUACAGCUCAGCAGGAAGACUUUUGAGACAGAAUUUCAGAAAACAAAAAAUCAAGAGUGGGACUUUGUCAUAACAACUGACAUCUCAGAGAUGGGCGCCAACUUCAAGGCUGACCGGGUCAUAGACUCUAGGAGAUG
KU922960    GCAAUGAGAUCGCAGCUUGUCUGACAAAGGCUGGAAAACGGGUCAUACAGCUCAGCAGAAAGACUUUUGAGACAGAGUUCCAGAAAACAAAACAUCAAGAGUGGGACUUUGUCGUGACAACUGACAUUUCAGAGAUGGGCGCCAACUUUAAAGCUGACCGUGUCAUAGAUUCCAGGAGAUG
KU922923    GCAAUGAGAUCGCAGCUUGUCUGACAAAGGCUGGAAAACGGGUCAUACAGCUCAGCAGAAAGACUUUUGAGACAGAGUUCCAGAAAACAAAACAUCAAGAGUGGGACUUUGUCGUGACAACUGACAUUUCAGAGAUGGGCGCCAACUUUAAAGCUGACCGUGUCAUAGAUUCCAGGAGAUG
KU820898    GCAAUGAGAUCGCAGCUUGUCUGACAAAGGCUGGAAAACGGGUCAUACAGCUCAGCAGAAAGACUUUUGAGACAGAGUUCCAGAAAACAAAACAUCAAGAGUGGGACUUUGUCGUGACAACUGACAUUUCAGAGAUGGGCGCCAACUUUAAAGCUGACCGUGUCAUAGAUUCCAGGAGAUG
KU321639    GCAAUGAGAUCGCAGCUUGUCUGACAAAGGCUGGAAAACGGGUCAUACAGCUCAGCAGAAAGACUUUUGAGACAGAGUUCCAGAAAACAAAACAUCAAGAGUGGGACUUUGUCGUGACAACUGACAUUUCAGAGAUGGGCGCCAACUUUAAAGCUGACCGUGUCAUAGAUUCCAGGAGAUG
KU820899    GCAAUGAGAUCGCAGCUUGUCUGACAAAGGCUGGAAAACGGGUCAUACAGCUCAGCAGAAAGACUUUUGAGACAGAGUUCCAGAAAACAAAACAUCAAGAGUGGGACUUUGUCGUGACAACUGACAUUUCAGAGAUGGGCGCCAACUUUAAAGCUGACCGUGUCAUAGAUUCCAGGAGAUG
KU853012    GCAAUGAGAUCGCAGCUUGUCUGACAAAGGCUGGAAAACGGGUCAUACAGCUCAGCAGAAAGACUUUUGAGACAGAGUUCCAGAAAACAAAACAUCAAGAGUGGGACUUUGUCGUGACAACCGACAUUUCAGAGAUGGGCGCCAACUUUAAAGCUGACCGUGUCAUAGAUUCCAGGAGAUG
KU729217    GCAAUGAGAUCGCAGCUUGUCUGACAAAGGCUGGAAAACGGGUCAUACAGCUCAGCAGAAAGACUUUUGAGACAGAGUUCCAGAAAACAAAACAUCAAGAGUGGGACUUUGUCGUGACAACUGACAUUUCAGAGAUGGGCGCCAACUUUAAAGCUGACCGUGUCAUAGAUUCCAGGAGAUG
KU729218    GCAAUGAGAUCGCAGCUUGUCUGACAAAGGCUGGAAAACGGGUCAUACAGCUCAGCAGAAAGACUUUUGAGACAGAGUUCCAGAAAACAAAACAUCAAGAGUGGGACUUUGUCGUGACAACUGACAUUUCAGAGAUGGGCGCCAACUUUAAAGCUGACCGUGUCAUAGAUUCCAGGAGAUG
KU761564    GCAAUGAGAUCGCAGCUUGUCUGACAAAGGCUGGAAAACGGGUCAUACAGCUCAGCAGAAAGACUUUUGAGACAGAGUUCCAGAAAACAAAACAUCAAGAGUGGGACUUUGUCGUGACAACUGACAUUUCAGAGAUGGGCGCCAACUUUAAAGCUGACCGUGUCAUAGAUUCCAGGAGAUG
KU740184    GCAAUGAGAUCGCAGCUUGUCUGACAAAGGCUGGAAAACGGGUCAUACAGCUCAGCAGAAAGACUUUUGAGACAGAGUUCCAGAAAACAAAACAUCAAGAGUGGGACUUUGUCGUGACAACUGACAUUUCAGAGAUGGGCGCCAACUUUAAAGCUGACCGUGUCAUAGAUUCCAGGAGAUG
KU744693    GCAAUGAGAUCGCAGCUUGUCUGACAAAGGCUGGAAAACGGGUCAUACAGCUCAGCAGAAAGACUUUUGAGACAGAGUUCCAGAAAACAAAACAUCAAGAGUGGGACUUUGUCGUGACAACUGACAUUUCAGAGAUGGGCGCCAACUUUAAAGCUGACCGUGUCAUAGAUUCCAGGAGAUG
KU497555    GCAAUGAGAUCGCAGCUUGUCUGACAAAGGCUGGAAAACGGGUCAUACAGCUCAGCAGAAAGACUUUUGAGACAGAGUUCCAGAAAACAAAACAUCAAGAGUGGGACUUUGUCGUGACAACUGACAUUUCAGAGAUGGGCGCCAACUUUAAAGCUGACCGUGUCAUAGAUUCCAGGAGAUG
KU527068    GCAAUGAGAUCGCAGCUUGUCUGACAAAGGCUGGAAAACGGGUCAUACAGCUCAGCAGAAAGACUUUUGAGACAGAGUUCCAGAAAACAAAACAUCAAGAGUGGGACUUUGUCGUGACAACUGACAUUUCAGAGAUGGGCGCCAACUUUAAAGCUGACCGUGUCAUAGAUUCCAGGAGAUG
KU501217    GCAAUGAGAUCGCAGCUUGUCUGACAAAGGCUGGAAAACGGGUCAUACAGCUCAGCAGAAAGACUUUUGAGACAGAGUUCCAGAAAACAAAACAUCAAGAGUGGGACUUUGUCGUGACAACUGACAUUUCAGAGAUGGGCGCCAACUUUAAAGCUGACCGUGUCAUAGAUUCCAGGAGAUG
KU501216    GCAAUGAGAUCGCAGCUUGUCUGACAAAGGCUGGAAAACGGGUCAUACAGCUCAGCAGAAAGACUUUUGAGACAGAGUUCCAGAAAACAAAACAUCAAGAGUGGGACUUUGUCGUGACAACUGACAUUUCAGAGAUGGGCGCCAACUUUAAAGCUGACCGUGUCAUAGAUUCCAGGAGAUG
KU501215    GCAAUGAGAUCGCAGCUUGUCUGACAAAGGCUGGAAAACGGGUCAUACAGCUCAGCAGAAAGACUUUUGAGACAGAGUUCCAGAAAACAAAACAUCAAGAGUGGGACUUUGUCGUGACAACUGACAUUUCAGAGAUGGGCGCCAACUUUAAAGCUGACCGUGUCAUAGAUUCCAGGAGAUG
KU365780    GCAAUGAGAUCGCAGCUUGUCUGACAAAGGCUGGAAAACGGGUCAUACAGCUCAGCAGAAAGACUUUUGAGACAGAGUUCCAGAAAACAAAACAUCAAGAGUGGGACUUUGUCGUGACAACUGACAUUUCAGAGAUGGGCGCCAACUUUAAAGCUGACCGUGUCAUAGAUUCCAGGAGAUG
KU365779    GCAAUGAGAUCGCAGCUUGUCUGACAAAGGCUGGAAAACGGGUCAUACAGCUCAGCAGAAAGACUUUUGAGACAGAGUUCCAGAAAACAAAACAUCAAGAGUGGGACUUUGUCGUGACAACUGACAUUUCAGAGAUGGGCGCCAACUUUAAAGCUGACCGUGUCAUAGAUUCCAGGAGAUG
KU707826    GCAAUGAGAUCGCAGCUUGUCUGACAAAGGCUGGAAAACGGGUCAUACAGCUCAGCAGAAAGACUUUUGAGACAGAGUUCCAGAAAACAAAACAUCAAGAGUGGGACUUUGUCGUGACAACUGACAUUUCAGAGAUGGGCGCCAACUUUAAAGCUGACCGUGUCAUAGAUUCCAGGAGAUG
KU365778    GCAAUGAGAUCGCAGCUUGUCUGACAAAGGCUGGAAAACGGGUCAUACAGCUCAGCAGAAAGACUUUUGAGACAGAGUUCCAGAAAACAAAACAUCAAGAGUGGGACUUUGUCGUGACAACUGACAUUUCAGAGAUGGGCGCCAACUUUAAAGCUGACCGUGUCAUAGAUUCCAGGAGAUG
KU365777    GCAAUGAGAUCGCAGCUUGUCUGACAAAGGCUGGAAAACGGGUCAUACAGCUCAGCAGAAAGACUUUUGAGACAGAGUUCCAGAAAACAAAACAUCAAGAGUGGGACUUUGUCGUGACAACUGACAUUUCAGAGAUGGGCGCCAACUUUAAAGCUGACCGUGUCAUAGAUUCCAGGAGAUG
KU312312    GCAAUGAGAUCGCAGCUUGUCUGACAAAGGCUGGAAAACGGGUCAUACAGCUCAGCAGAAAGACUUUUGAGACAGAGUUCCAGAAAACAAAACAUCAAGAGUGGGACUUUGUCGUGACAACUGACAUUUCAGAGAUGGGCGCCAACUUUAAAGCUGACCGUGUCAUAGAUUCCAGGAGAUG
KF268950    GAAAUGAAAUCGCAGCCUGUCUGACAAAGGCUGGAAAGCGGGUCAUACAACUCAGCAGGAAGACUUUUGAGACAGAGUUUCAGAAAACAAAAAAUCAAGAGUGGGACUUUGUCAUAACAACUGACAUCUCAGAGAUGGGCGCGAAUUUCAAAGCUGACCGGGUCAUAGACUCUAGGAGAUG
KF268949    GAAAUGAAAUCGCAGCCUGUCUGACAAAGGCUGGAAAGCGGGUCAUACAACUCAGCAGGAAGACUUUUGAGACAGAGUUUCAGAAAACAAAAAAUCAAGAGUGGGACUUUGUCAUAACAACUGACAUCUCAGAGAUGGGUGCCAAUUUCAAGGCUGACCGGGUCAUAGACUCUAGGAGAUG
KF268948    GAAAUGAAAUCGCAGCCUGUCUGACAAAGGCUGGAAAGCGGGUCAUACAACUCAGCAGGAAGACUUUUGAGACAGAGUUUCAGAAAACAAAAAAUCAAGAGUGGGACUUUGUCAUAACAACUGACAUCUCAGAGAUGGGCGCGAAUUUCAAAGCUGACCGGGUCAUAGACUCUAGGAGAUG
KJ776791    GCAAUGAGAUCGCAGCUUGUCUGACAAAGGCUGGAAAACGGGUCAUACAGCUCAGCAGAAAGACUUUUGAGACAGAGUUCCAGAAAACAAAACAUCAAGAGUGGGACUUUGUCGUGACAACUGACAUUUCAGAGAUGGGCGCCAACUUUAAAGCUGACCGUGUCAUAGAUUCCAGGAGAUG
KF383119    GAAAUGAAAUCGCAGCCUGUCUGACAAAGGCUGGAAAGCGGGUCAUACAGCUCAGCAGGAAGACUUUUGAGACAGAAUUUCAGAAAACAAAAAAUCAAGAGUGGGACUUUGUCAUAACAACUGACAUCUCAGAGAUGGGCGCCAACUUCAAGGCUGACCGGGUCAUAGACUCUAGGAGAUG
KF383118    GAGAAGAAAGCGCAGCCUGUCUGACAAAGGCUGGAAAGCGGGUCAUACAGCUCAGCAGGAAGACUUUUGAGACAGAAUUUCAGAAAACAAAAAAUCAAGAGUGGGACUUUGUCAUAACAACUGACAUCUCAGAGAUGGGCGCCAACUUCAAGGCUGACCGGGUCAUAGACUCUAGGAGAUG
KF383117    GAAAUGAAAUCGCAGCCUGUCUGACAAAGGCUGGAAAGCGGGUUAUACAGCUCAGCAGGAAGACUUUUGAGACAGAGUUUCAGAAGACAAAAAAUCAAGAGUGGGACUUUGUCAUAACAACUGACAUUUCAGAGAUGGGUGCCAAUUUCAAGGCUGACCGGGUCAUCGAUUCCAGGAGAUG
KF383116    GAGAAGAAAGCGCAGCCUGUCUGACAAAGGCUGGAAAGCGGGUUAUACAGCUCAGCAGGAAGACUUUUGAGACAGAGUUUCAGAAGACAAAAAAUCAAGAGUGGGACUUUGUCAUAACAACUGACAUUUCAGAGAUGGGUGCCAAUUUCAAGGCUGACCGGGUCAUAGAUUCCAGGAGAUG
KF383115    GAAAUGAAAUCACAGCCUGCCUGACAAAGGCUGGAAAGCGGGUCAUACAGCUCAGCAGGAAGACUUUUGAGACAGAGUUUCAGAAAACAAAAAACCAAGAGUGGGACUUUGUCAUAACAACUGACAUCUCAGAGAUGGGCGCCAAUUUCAAGGCUGACCGGGUCAUAGACUCUAGGAGAUG
EU545988    GCAAUGAGAUCGCAGCUUGUCUGACAAAGGCUGGAAAACGGGUCAUACAGCUCAGCAGAAAGACUUUUGAGACAGAGUUCCUGAAAACAAAAAAUCAAGAGUGGGACUUCGUCGUGACAACUGACAUUUCAGAGAUGGGCGCCAACUUUAAAGCUGACCGUGUCAUAGAUUCCAGGAGAUG
KU955589    GCAAUGAGAUCGCAGCUUGUCUGACAAAGGCUGGAAAACGGGUCAUACAGCUCAGCAGAAAGACUUUUGAGACAGAGUUCCAGAAAACAAAACAUCAAGAGUGGGACUUUGUCGUGACAACUGACAUUUCAGAGAUGGGCGCCAACUUUAAAGCUGACCGUGUCAUAGAUUCCAGGAGAUG
KU955590    GCAAUGAGAUCGCAGCUUGUCUGACAAAGGCUGGAAAACGGGUCAUACAGCUCAGCAGAAAGACUUUUGAGACAGAGUUCCAGAAAACAAAACAUCAAGAGUGGGACUUUGUCGUGACAACUGACAUUUCAGAGAUGGGCGCCAACUUUAAAGCUGACCGUGUCAUAGAUUCCAGGAGAUG
KU940228    GCAAUGAGAUCGCAGCUUGUCUGACAAAGGCUGGAAAACGGGUCAUACAGCUCAGCAGAAAGACUUUUGAGACAGAGUUCCAGAAAACAAAACAUCAAGAGUGGGACUUUGUCGUGACAACUGACAUUUCAGAGAUGGGCGCCAACUUUAAAGCUGACCGUGUCAUAGAUUCCAGGAGAUG
KU991811    GCAAUGAGAUCGCAGCUUGUCUGACAAAGGCUGGAAAACGGGUCAUACAGCUCAGCAGAAAGACUUUUGAGACAGAGUUCCAGAAAACAAAACAUCAAGAGUGGGACUUUGUCGUGACAACUGACAUUUCAGAGAUGGGCGCCAACUUUAAAGCUGACCGUGUCAUAGAUUCCAGGAGAUG
KU509998    GCAAUGAGAUCGCAGCUUGUCUGACAAAGGCUGGAAAACGGGUCAUACAGCUCAGCAGAAAGACUUUUGAGACAGAGUUCCAGAAAACAAAACAUCAAGAGUGGGACUUUGUCGUGACAACUGACAUUUCAGAGAUGGGCGCCAACUUUAAAGCUGACCGUGUCAUAGAUUCCAGGAGAUG
KX056898    GCAAUGAGAUCGCAGCUUGUCUGACAAAGGCUGGAAAACGGGUCAUACAGCUCAGCAGAAAGACUUUUGAGACAGAGUUCCAGAAAACAAAACAUCAAGAGUGGGACUUUGUCGUGACAACUGACAUUUCAGAGAUGGGCGCCAACUUUAAAGCUGACCGUGUCAUAGAUUCCAGGAGAUG
KX117076    GCAAUGAGAUCGCAGCUUGUCUGACAAAGGCUGGAAAACGGGUCAUACAGCUCAGCAGAAAGACUUUUGAGACAGAGUUCCAGAAAACAAAACAUCAAGAGUGGGACUUUGUCGUGACAACUGACAUUUCAGAGAUGGGCGCCAACUUUAAAGCUGACCGUGUCAUAGAUUCCAGGAGAUG
KX185891    GCAAUGAGAUCGCAGCUUGUCUGACAAAGGCUGGAAAACGGGUCAUACAGCUCAGCAGAAAGACUUUUGAGACAGAGUUCCAGAAAACAAAACAUCAAGAGUGGGACUUUGUCGUGACAACUGACAUUUCAGAGAUGGGCGCCAACUUUAAAGCUGACCGUGUCAUAGAUUCCAGGAGAUG
KX247646    GCAAUGAGAUCGCAGCUUGUCUGACAAAGGCUGGAAAACGGGUCAUACAGCUCAGCAGAAAGACUUUUGAGACAGAGUUCCAGAAAACAAAACAUCAAGAGUGGGACUUUGUCGUGACAACUGACAUUUCAGAGAUGGGCGCCAACUUUAAAGCUGACCGUGUCAUAGAUUCCAGGAGAUG
KX262887    GCAAUGAGAUCGCAGCUUGUCUGACAAAGGCUGGAAAACGGGUCAUACAGCUCAGCAGAAAGACUUUUGAGACAGAGUUCCAGAAAACAAAACAUCAAGAGUGGGACUUUGUCGUGACAACUGACAUUUCAGAGAUGGGCGCCAACUUUAAAGCUGACCGUGUCAUAGAUUCCAGGAGAUG
KX280026    GCAAUGAGAUCGCAGCUUGUCUGACAAAGGCUGGAAAACGGGUCAUACAGCUCAGCAGAAAGACUUUUGAGACAGAGUUCCAGAAAACAAAACAUCAAGAGUGGGACUUUGUCGUGACAACUGACAUUUCAGAGAUGGGCGCCAACUUUAAAGCUGACCGUGUCAUAGAUUCCAGGAGAUG
KU758877    GCAAUGAGAUCGCAGCUUGUCUGACAAAGGCUGGAAAACGGGUCAUACAGCUCAGCAGAAAGACUUUUGAGACAGAGUUCCAGAAAACAAAACAUCAAGAGUGGGACUUUGUCGUGACAACUGACAUUUCAGAGAUGGGCGCCAACUUUAAAGCUGACCGUGUCAUAGAUUCCAGGAGAUG
KU866423    GCAAUGAGAUCGCAGCUUGUCUGACAAAGGCUGGAAAACGGGUCAUACAGCUCAGCAGAAAGACUUUUGAGACAGAGUUCCAGAAAACAAAACAUCAAGAGUGGGACUUUGUCGUGACAACUGACAUUUCAGAGAUGGGCGCCAACUUUAAAGCUGACCGUGUCAUAGAUUCCAGGAGAUG
KX377336    GCAACGAGAUCGCGGCUUGUCUGACAAAAGCUGGAAAACGGGUCAUACAGCUCAGCAGAAAGACUUUUGAGACAGAGUUCCAGAAAACAAAAAAUCAAGAGUGGGACUUCGUCGUAACAACUGACAUCUCAGAGAUGGGCGCCAACUUCAAAGCUGACCGGGUCAUAGAUUCCAGGAGAUG
KX377337    GCAAUGAGAUCGCAGCUUGUCUGACAAAGGCUGGAAAACGGGUCAUACAGCUCAGCAGAAAGACUUUUGAGACAGAGUUCCAGAAAACAAAACAUCAAGAGUGGGACUUUGUCGUGACAACUGACAUUUCAGAGAUGGGCGCCAACUUUAAAGCUGACCGUGUCAUAGAUUCCAGGAGAUG
KX369547    GCAAUGAGAUCGCAGCUUGUCUGACAAAGGCUGGAAAACGGGUCAUACAGCUCAGCAGAAAGACUUUUGAGACAGAGUUCCAGAAAACAAAACAUCAAGAGUGGGACUUUGUCGUGACAACUGACAUUUCAGAGAUGGGCGCCAACUUUAAAGCUGACCGUGUCAUAGAUUCCAGGAGAUG
KX548902    GCAAUGAGAUCGCAGCUUGUCUGACAAAGGCUGGAAAACGGGUCAUACAGCUCAGCAGAAAGACUUUUGAGACAGAGUUCCAGAAAACAAAACAUCAAGAGUGGGACUUUGUCGUGACAACUGACAUUUCAGAGAUGGGCGCCAACUUUAAAGCUGACCGUGUCAUAGAUUCCAGGAGAUG
KX601166    GAAAUGAAAUCGCAGCCUGUCUGACAAAGGCUGGAAAGCGGGUCAUACAGCUCAGCAGGAAGACUUUUGAGACAGAGUUUCAGAAGACAAAAAAUCAAGAGUGGGACUUUGUCAUAACAACUGACAUUUCAGAGAUGGGUGCCAAUUUCAAGGCUGACCGGGUCAUAGAUUCCAGGAGAUG
KX601167    GCAACGAGAUCGCGGCUUGUCUGACAAAAGCUGGAAAACGGGUCAUACAGCUCAGCAGAAAGACUUUUGAGACAGAGUUCCAGAAAACAAAAAAUCAAGAGUGGGACUUCGUCGUAACAACUGACAUCUCAGAGAUGGGCGCCAACUUCAAAGCUGACCGGGUCAUAGAUUCCAGGAGAUG
KX601168    GCAAUGAGAUCGCAGCUUGUCUGACAAAGGCUGGAAAACGGGUCAUACAGCUCAGCAGAAAGACUUUUGAGACAGAGUUCCAGAAAACAAAACAUCAAGAGUGGGACUUUGUCGUGACAACUGACAUUUCAGAGAUGGGCGCCAACUUUAAAGCUGACCGUGUCAUAGAUUCCAGGAGAUG
KX766028    GCAAUGAGAUCGCAGCUUGUCUGACAAAGGCUGGAAAACGGGUCAUACAGCUCAGCAGAAAGACUUUUGAGACAGAGUUCCAGAAAACAAAACAUCAAGAGUGGGACUUUGUCGUGACAACUGACAUUUCAGAGAUGGGCGCCAACUUUAAAGCUGACCGUGUCAUAGAUUCCAGGAGAUG
KX766029    GCAAUGAGAUCGCAGCUUGUCUGACAAAGGCUGGAAAACGGGUCAUACAGCUCAGCAGAAAGACUUUUGAGACAGAGUUCCAGAAAACAAAACAUCAAGAGUGGGACUUUGUCGUGACAACUGACAUUUCAGAGAUGGGCGCCAACUUUAAAGCUGACCGUGUCAUAGAUUCCAGGAGAUG
KU940224    GCAAUGAGAUCGCAGCUUGUCUGACAAAGGCUGGAAAACGGGUCAUACAGCUCAGCAGAAAGACUUUUGAGACAGAGUUCCAGAAAACAAAACAUCAAGAGUGGGACUUUGUCGUGACAACUGACAUUUCAGAGAUGGGCGCCAACUUUAAAGCUGACCGUGUCAUAGAUUCCAGGAGAUG


            5555555555555555555555555555555555555555555555555555555555555555555555555555555555555555555555555555555555555555555555555555555555555555555555555555555555555555555555555555555
            7777777888888888888888888888888888888888888888888888888888888888888888888888888888888888888888888888888888999999999999999999999999999999999999999999999999999999999999999999999999
            9999999000000000011111111112222222222333333333344444444445555555555666666666677777777778888888888999999999900000000001111111111222222222233333333334444444444555555555566666666667777
Sequence    3456789012345678901234567890123456789012345678901234567890123456789012345678901234567890123456789012345678901234567890123456789012345678901234567890123456789012345678901234567890123
-------------------------------------------------------------------------------------------------------------------------------------------------------------------------------------------------
NC_012532   CCUAAAACCAGUCAUACUUGAUGGUGAGAGAGUCAUCUUGGCUGGGCCCAUGCCUGUCACGCAUGCUAGUGCUGCUCAGAGGAGAGGACGUAUAGGCAGGAACCCUAACAAACCUGGAGAUGAGUACAUGUAUGGAGGUGGGUGUGCAGAGACUGAUGAAGGCCAUGCACACUGGCUUGAA
KU922960    CCUAAAGCCGGUCAUACUUGAUGGCGAGAGAGUCAUUCUGGCUGGACCCAUGCCUGUCACACAUGCCAGCGCUGCCCAGAGGAGGGGGCGCAUAGGCAGGAAUCCCAAUAAACCUGGAGAUGAGUAUCUGUAUGGAGGUGGGUGCGCAGAGACUGACGAAGACCAUGCACACUGGCUUGAA
KU922923    CCUAAAGCCGGUCAUACUUGAUGGCGAGAGAGUCAUUCUGGCUGGACCCAUGCCUGUCACACAUGCCAGCGCUGCCCAGAGGAGGGGGCGCAUAGGCAGGAAUCCCAAUAAACCUGGAGAUGAGUAUCUGUAUGGAGGUGGGUGCGCAGAGACUGACGAAGACCAUGCACACUGGCUUGAA
```

```
KU820898        CCUAAAGCCGGUCAUACUUGAUGGCGAGAGAGUCAUUCUGGCUGGACCCAUGCCUGUCACACAUGCCAGCGCUGCCCAGAGGAGGGGGCGCAUAGGCAGGAAUCCCAACAAACCUGGAGAUGAGUAUCUGUAUGGAGGUGGGUGCGCAGAGACUGACGAAGACCAUGCACACUGGCUUGAA
KU321639        CCUAAAGCCGGUCAUACUUGAUGGCGAGAGAGUCAUUCUGGCUGGACCCAUGCCUGUCACACAUGCCAGCGCUGCCCAGAGGAGGGGGCGCAUAGGCAGGAAUCCCAACAAACCUGGAGAUGAGUAUCUGUAUGGAGGUGGGUGCGCAGAGACUGACGAAGACCAUGCACACUGGCUUGAA
KU820899        CCUAAAGCCGGUCAUACUUGAUGGCGAGAGAGUCAUUCUGGCUGGACCCAUGCCUGUCACACAUGCCAGCGCUGCCCAGAGGAGGGGGCGCAUAGGCAGGAAUCCCAACAAACCUGGAGAUGAGUAUCUGUAUGGAGGUGGGUGCGCAGAGACUGACGAAGACCAUGCACACUGGCUUGAA
KU853012        CCUAAAGCCGGUCAUACUUGAUGGCGAGAGAGUCAUUCUGGCUGGACCCAUGCCUGUCACACAUGCCAGCGCUGCCCAGAGGAGGGGGCGCAUAGGCAGGAAUCCCAACAAACCUGGAGAUGAGUAUCUGUAUGGAGGUGGGUGCGCAGAGACUGACGAAGACCAUGCACACUGGCUUGAA
KU729217        CCUAAAGCCGGUCAUACUUGAUGGCGAGAGAGUCAUUCUGGCUGGACCCAUGCCUGUCACACAUGCCAGCGCUGCCCAGAGGAGGGGGCGCAUAGGCAGGAAUCCCAACAAACCUGGAGAUGAGUAUCUGUAUGGAGGUGGGUGCGCAGAGACUGACGAAGACCAUGCACACUGGCUUGAA
KU729218        CCUAAAGCCGGUCAUACUUGAUGGCGAGAGAGUCAUUCUGGCUGGACCCAUGCCUGUCACACAUGCCAGCGCUGCCCAGAGGAGGGGGCGCAUAGGCAGGAAUCCCAACAAACCUGGAGAUGAGUAUCUGUAUGGAGGUGGGUGCGCAGAGACUGACGAAGACCAUGCACACUGGCUUGAA
KU761564        CCUAAAGCCGGUCAUACUUGAUGGCGAGAGAGUCAUUCUGGCUGGACCCAUGCCUGUCACACAUGCCAGCGCUGCCCAGAGGAGGGGGCGCAUAGGCAGGAAUCCCAACAAACCUGGAGAUGAGUAUCUGUAUGGAGGUGGGUGCGCAGAGACUGACGAAGACCAUGCACACUGGCUUGAA
KU740184        CCUAAAGCCGGUCAUACUUGAUGGCGAGAGAGUCAUUCUGGCUGGACCCAUGCCUGUCACACAUGCCAGCGCUGCCCAGAGGAGGGGGCGCAUAGGCAGGAAUCCCAACAAACCUGGAGAUGAGUAUCUGUAUGGAGGUGGGUGCGCAGAGACUGACGAAGACCAUGCACACUGGCUUGAA
KU744693        CCUAAAGCCGGUCAUACUUGGUGGCGAGAGAGUCAUUCUGGCUGGACCCAUGCCUGUCACACAUGCCAGCGCUGCCCAGAGGAGGGGGCGCAUAGGCAGGAAUCCCAACAAACCUGGAGAUGAGUAUCUGUAUGGAGGUGGGUGCGCAGAGACUGACGAAGACCAUGCACACUGGCUUGAA
KU497555        CCUAAAGCCGGUCAUACUUGAUGGCGAGAGAGUCAUUCUGGCUGGACCCAUGCCUGUCACACAUGCCAGCGCUGCCCAGAGGAGGGGGCGCAUAGGCAGGAAUCCCAACAAACCUGGAGAUGAGUACCUGUAUGGAGGUGGGUGCGCAGAGACUGACGAAGACCAUGCACACUGGCUUGAA
KU527068        CCUAAAGCCGGUCAUACUUGAUGGCGAGAGAGUCAUUUUGGCUGGACCCAUGCCUGUCACACAUGCCAGCGCUGCCCAGAGGAGGGGGCGCAUAGGCAGGAAUCCCAACAAACCUGGAGAUGAGUAUCUGUAUGGAGGUGGGUGCGCAGAGACUGACGAAGACCAUGCACACUGGCUUGAA
KU501217        CCUAAAGCCGGUCAUACUUGAUGGCGAGAGAGUCAUUCUGGCUGGACCCAUGCCUGUCACACAUGCCAGCGCUGCCCAGAGGAGGGGGCGCAUAGGCAGGAAUCCCAACAAACCUGGAGAUGAGUAUCUGUAUGGAGGUGGGUGCGCAGAGACUGACGAAGACCAUGCACACUGGCUUGAA
KU501216        CCUAAAGCCGGUCAUACUUGAUGGCGAGAGAGUCAUUCUGGCUGGACCCAUGCCUGUCACACAUGCCAGCGCUGCCCAGAGGAGGGGGCGCAUAGGCAGGAAUCCCAACAAACCUGGAGAUGAGUAUCUGUAUGGAGGUGGGUGCGCAGAGACUGACGAAGACCAUGCACACUGGCUUGAA
KU501215        CCUAAAGCCGGUCAUACUUGAUGGCGAGAGAGUCAUUCUGGCUGGACCCAUGCCUGUCACACAUGCCAGCGCUGCCCAGAGGAGGGGGCGCAUAGGCAGGAAUCCCAACAAACCUGGAGAUGAGUAUCUGUAUGGAGGUGGGUGCGCAGAGACUGACGAAGACCAUGCACACUGGCUUGAA
KU365780        CCUAAAGCCGGUCAUACUUGAUGGCGAGAGAGUCAUUCUGGCUGGACCCAUGCCUGUCACACAUGCCAGCGCUGCCCAGAGGAGGGGGCGCAUAGGCAGGAAUCCCAACAAACCUGGAGAUGAGUAUCUGUAUGGAGGUGGGUGCGCAGAGACUGACGAAGACCAUGCACACUGGCUUGAA
KU365779        CCUAAAGCCGGUCAUACUUGAUGGCGAGAGAGUCAUUCUGGCUGGACCCAUGCCUGUCACACAUGCCAGCGCUGCCCAGAGGAGGGGGCGCAUAGGCAGGAAUCCCAACAAACCUGGAGAUGAGUAUCUGUAUGGAGGUGGGUGCGCAGAGACUGACGAAGACCAUGCACACUGGCUUGAA
KU707826        CCUAAAGCCGGUCAUACUUGAUGGCGAGAGAGUCAUUCUGGCUGGACCCAUGCCUGUCACACAUGCCAGCGCUGCCCAGAGGAGGGGGCGCAUAGGCAGGAAUCCCAACAAACCUGGAGAUGAGUAUCUGUAUGGAGGUGGGUGCGCAGAGACUGACGAAGACCAUGCACACUGGCUUGAA
KU365778        CCUAAAGCCGGUCAUACUUGAUGGCGAGAGAGUCAUUCUGGCUGGACCCAUGCCUGUCACACAUGCCAGCGCUGCCCAGAGGAGGGGGCGCAUAGGCAGGAAUCCCAACAAACCUGGAGAUGAGUAUCUGUAUGGAGGUGGGUGCGCAGAGACUGACGAAGACCAUGCACACUGGCUUGAA
KU365777        CCUAAAGCCGGUCAUACUUGAUGGCGAGAGAGUCAUUCUGGCUGGACCCAUGCCUGUCACACAUGCCAGCGCUGCCCAGAGGAGGGGGCGCAUAGGCAGGAAUCCCAACAAACCUGGAGAUGAGUAUCUGUAUGGAGGUGGGUGCGCAGAGACUGACGAAGACCAUGCACACUGGCUUGAA
KU312312        CCUAAAGCCGGUCAUACUUGAUGGCGAGAGAGUCAUUCUGGCUGGACCCAUGCCUGUCACACAUGCCAGCGCUGCCCAGAGGAGGGGGCGCAUAGGCAGGAAUCCCAACAAACCUGGAGAUGAGUAUCUGUAUGGAGGUGGGUGCGCAGAGACUGACGAAGACCAUGCACACUGGCUUGAA
KF268950        CCUAAAGCCAGUCAUACUUGAUGGUGAGAGAGUCAUCUUGGCUGGGCCCAUGCCUGUCACGCAUGCUAGUGCUGCUCAAAGGAGAGGGCGUAUAGGCAGGAACCCUAACAAACCUGGAGAUGAGUACAUGUAUGGAGGUGGAUGUGCAGAGACCGAUGAAGACCAUGCACACUGGCUUGAA
KF268949        CCUAAAGCCAGUUAUACUUGAUGGUGAGAGAGUCAUCUUGGCUGGGCCCAUGCCUGUCACGCAUGCUAGUGCUGCUCAAAGGAGAGGGCGUAUAGGCAGGAACCCUAACAAACCUGGAGAUGAGUACAUGUAUGGAGGUGGAUGUGCAGAGACCGAUGAAGACCAUGCACACUGGCUUGAA
KF268948        CCUAAAGCCAGUCAUACUUGAUGGUGAGAGAGUCAUCUUGGCUGGGCCCAUGCCUGUCACGCAUGCUAGUGCUGCUCAAAGGAGAGGGCGUAUAGGCAGGAACCCUAACAAACCUGGAGAUGAGUACAUGUAUGGAGGUGGAUGUGCAGAGACCGAUGAAGACCAUGCACACUGGCUUGAA
KJ776791        CCUAAAGCCGGUCAUACUUGAUGGCGAGAGAGUCAUUCUGGCUGGACCCAUGCCUGUCACACAUGCCAGCGCUGCCCAGAGGAGGGGGCGCAUAGGCAGGAAUCCCAACAAACCUGGAGAUGAGUAUCUGUAUGGAGGUGGGUGCGCAGAGACUGACGAAGACCAUGCACACUGGCUUGAA
KF383119        CCUAAAACCAGUCAUACUUGAUGGUGAGAGAGUCAUCUUGGCUGGGCCCAUGCCUGUCACGCAUGCUAGUGCUGCUCAGAGGAGAGGACGUAUAGGCAGGAACCCUAACAAACCUGGAGAUGAGUACAUGUAUGGAGGUGGGUGUGCAGAGACUGAUGAAGGCCAUGCACACUGGCUUGAA
KF383118        CCUAAAGCCAGUCAUACUUGAUGGUGAGAGAGUCAUCUUGGCUGGGCCCAUGCCUGUCACGCAUGCUAGUGCUGCUCAGAGGAGAGGACGUAUAGGCAGGAACCCUAACAAACCUGGAGAUGAGUACAUGUAUGGAGGUGGGUGUGCAGAGACUGAUGAAGACCAUGCACACUGGCUUGAA
KF383117        CCUAAAGCCAGUCAUACUUGAUGGUGAGAGAGUCAUCCUGGCUGGGCCCAUGCCUGUCACGCAUGCCAGUGCUGCUCAGAGGAGAGGACGUAUAGGCAGGAACCCCAACAAACCCGGAGAUGAGUACAUGUAUGGAGGUGGGUGUGCAGAGACUGAUGAAGACCAUGCACACUGGCUUGAA
KF383116        CCUAAAGCCAGUCAUACUCGAUGGUGAGAGAGUCAUCCUGGCUGGGCCCAUGCCUGUCACGCAUGCCAGUGCUGCUCAGAGGAGAGGACGUAUAGGCAGGAACCCCAACAAACCUGGAGAUGAGUACAUGUAUGGAGGUGGGUGUGCAGAGACUGAUGAAGACCAUGCACACUGGCUUGAA
KF383115        CCUAAAGCCAGUCAUACUUGAUGGUGAGAGAGUCAUCUUGGCUGGGCCCAUGCCUGUCACGCAUGCUAGUGCUGCUCAGAGGAGAGGACGUAUAGGCAGGAACCCUAACAAACCUGGAGAUGAGUACAUGUAUGGAGGUGGGUGUGCAGAGACUGAUGAAGACCACGCACACUGGCUUGAA
EU545988        CCUAAAGCCGGUCAUACUUGAUGGCGAGAGAGUCAUUCUGGCUGGACCCAUGCCUGUCACACAUGCCAGCGCUGCCCAGAGGAGGGGGCGCAUAGGCAGGAAUCCCAACAAACCUGGAGAUGAGUAUCUGUAUGGAGGUGGGUGCGCAGAGACUGAUGAAGACCAUGCACACUGGCUUGAA
KU955589        CCUAAAGCCGGUCAUACUUGAUGGCGAGAGAGUCAUUCUGGCUGGACCCAUGCCUGUCACACAUGCCAGCGCUGCCCAGAGGAGGGGGCGCAUAGGCAGGAAUCCCAACAAACCUGGAGAUGAGUAUCUGUAUGGAGGUGGGUGCGCAGAGACUGACGAAGACCAUGCACACUGGCUUGAA
KU955590        CCUAAAGCCGGUCAUACUUGAUGGCGAGAGAGUCAUUCUGGCUGGACCCAUGCCUGUCACACAUGCCAGCGCUGCCCAGAGGAGGGGGCGCAUAGGCAGGAAUCCCAACAAACCUGGAGAUGAGUAUCUGUAUGGAGGUGGGUGCGCAGAGACUGACGAAGACCAUGCACAUUGGCUUGAA
KU940228        CCUAAAGCCGGUCAUACUUGAUGGCGAGAGAGUCAUUCUGGCUGGACCCAUGCCUGUCACACAUGCCAGCGCUGCCCAGAGGAGGGGGCGCAUAGGCAGGAAUCCCAACAAACCUGGAGAUGAGUAUCUGUAUGGAGGUGGGUGCGCAGAGACUGACGAAGACCAUGCACACUGGCUUGAA
KU991811        CCUAAAGCCGGUCAUACUUGAUGGCGAGAGAGUCAUUCUGGCUGGACCCAUGCCUGUCACACAUGCCAGCGCUGCCCAGAGGAGGGGGCGCAUAGGCAGGAAUCCCAACAAACCUGGAGAUGAGUAUCUGUAUGGAGGUGGGUGCGCAGAGACUGACGAAGACCAUGCACACUGGCUUGAA
KU509998        CCUAAAGCCGGUCAUACUUGAUGGCGAGAGAGUCAUUCUGGCUGGACCCAUGCCUGUCACACAUGCCAGCGCUGCCCAGAGGAGGGGGCGCAUAGGCAGGAAUCCCAACAAACCUGGAGAUGAGUAUCUGUAUGGAGGUGGGUGCGCAGAGACUGACGAAGACCAUGCACACUGGCUUGAA
KX056898        CCUAAAGCCGGUCAUACUUGAUGGCGAGAGAGUCAUUCUGGCUGGACCCAUGCCUGUCACACAUGCCAGCGCUGCCCAGAGGAGGGGGCGCAUAGGCAGGAAUCCCAACAAACCUGGAGAUGAGUAUCUGUAUGGAGGUGGGUGCGCAGAGACUGACGAAGACCAUGCACAUUGGCUUGAA
KX117076        CCUAAAGCCGGUCAUACUUGAUGGCGAGAGAGUCAUUCUGGCUGGACCCAUGCCUGUCACACAUGCCAGCGCUGCCCAGAGGAGGGGGCGCAUAGGCAGGAAUCCCAACAAACCUGGAGAUGAGUAUCUGUAUGGAGGUGGGUGCGCAGAGACUGACGAAGACCAUGCACACUGGCUUGAA
KX185891        CCUAAAGCCGGUCAUACUUGAUGGCGAGAGAGUCAUUCUGGCUGGACCCAUGCCUGUCACACAUGCCAGCGCUGCCCAGAGGAGGGGGCGCAUAGGCAGGAAUCCCAACAAACCUGGAGAUGAGUAUCUGUAUGGAGGUGGGUGCGCAGAGACUGACGAAGACCAUGCACACUGGCUUGAA
KX247646        CCUAAAGCCGGUCAUACUUGAUGGCGAGAGAGUCAUUCUGGCUGGACCCAUGCCUGUCACACAUGCCAGCGCUGCCCAGAGGAGGGGGCGCAUAGGCAGGAAUCCCAAUAAACCUGGAGAUGAGUAUCUGUAUGGAGGUGGGUGCGCAGAGACUGACGAAGACCAUGCACACUGGCUUGAA
KX262887        CCUAAAGCCGGUCAUACUUGAUGGCGAGAGAGUCAUUCUGGCUGGACCCAUGCCUGUCACACAUGCCAGCGCUGCCCAGAGGAGGGGGCGCAUAGGCAGGAAUCCCAACAAACCUGGAGAUGAGUAUCUGUAUGGAGGUGGGUGCGCAGAGACUGACGAAGACCAUGCACACUGGCUUGAA
KX280026        CCUAAAGCCGGUCAUACUUGAUGGCGAGAGAGUCAUUCUGGCUGGACCCAUGCCUGUCACACAUGCCAGCGCUGCCCAGAGGAGGGGGCGCAUAGGCAGGAAUCCCAACAAACCUGGAGAUGAGUAUCUGUAUGGAGGUGGGUGCGCAGAGACUGACGAAGACCAUGCACACUGGCUUGAA
KU758877        CCUAAAGCCGGUCAUACUUGAUGGCGAGAGAGUCAUUCUGGCUGGACCCAUGCCUGUCACACAUGCCAGCGCUGCCCAGAGGAGGGGGCGCAUAGGCAGGAAUCCCAACAAACCUGGAGAUGAGUAUCUGUAUGGAGGUGGGUGCGCAGAGACUGACGAAGACCAUGCACACUGGCUUGAA
KU866423        CCUAAAGCCGGUCAUACUUGAUGGCGAGAGAGUCAUUCUGGCUGGACCCAUGCCUGUCACACAUGCCAGCGCUGCCCAGAGGAGGGGGCGCAUAGGCAGGAAUCCCAACAAACCUGGAGAUGAGUAUCUGUAUGGAGGUGGGUGCGCAGAGACUGACGAAGACCAUGCACACUGGCUUGAA
KX377336        CCUGAAGCCGGUCAUACUUGAUGGCGAGAGAGUCAUUCUGGCUGGACCCAUGCCUGUCACACAUGCCAGCGCUGCCCAGAGGAGGGGGCGCAUAGGCAGGAAUCCCAACAAACCUGGAGAUGAGUAUAUGUAUGGAGGUGGGUGCGCAGAGACUGAUGAAGACCAUGCACACUGGCUUGAA
KX377337        CCUAAAGCCGGUCAUACUUGAUGGCGAGAGAGUCAUUCUGGCUGGACCCAUGCCUGUCACACAUGCCAGCGCUGCCCAGAGGAGGGGGCGCAUAGGCAGGAAUCCCAACAAACCUGGAGAUGAGUAUCUGUAUGGAGGUGGGUGCGCAGAGACUGACGAAGACCAUGCACACUGGCUUGAA
KX369547        CCUAAAGCCGGUCAUACUUGAUGGCGAGAGAGUCAUUCUGGCUGGACCCAUGCCUGUCACACAUGCCAGCGCUGCCCAGAGGAGGGGGCGCAUAGGCAGGAAUCCCAACAAACCUGGAGAUGAGUAUCUGUAUGGAGGUGGGUGCGCAGAGACUGACGAAGACCAUGCACACUGGCUUGAA
KX548902        CCUAAAGCCGGUCAUACUUGAUGGCGAGAGAGUCAUUCUGGCUGGACCCAUGCCUGUCACACAUGCCAGCGCUGCCCAGAGGAGGGGGCGCAUAGGCAGGAAUCCCAAUAAACCUGGAGAUGAGUAUCUGUAUGGAGGUGGGUGCGCAGAGACUGACGAAGACCAUGCACACUGGCUUGAA
KX601166        CCUAAAGCCAGUCAUACUCGAUGGCGAGAGAGUCAUCCUGGCUGGGCCCAUGCCUGUCACGCAUGCCAGUGCUGCUCAGAGGAGAGGACGUGUAGGCAGGAACCCCAACAAACCUGGAGAUGAGUACAUGUAUGGAGGUGGGUGUGCAGAGACUGAUGAAGACCAUGCACACUGGCUUGAA
KX601167        CCUGAAGCCGGUCAUACUUGAUGGCGAGAGAGUCAUUCUGGCUGGACCCAUGCCUGUCACACAUGCCAGCGCUGCCCAGAGGAGGGGGCGCAUAGGCAGGAAUCCCAACAAACCUGGAGAUGAGUAUAUGUAUGGAGGUGGGUGCGCAGAGACUGAUGAAGACCAUGCACACUGGCUUGAA
KX601168        CCUAAAGCCGGUCAUACUUGAUGGCGAGAGAGUCAUUCUGGCUGGACCCAUGCCUGUCACACAUGCCAGCGCUGCCCAGAGGAGGGGGCGCAUAGGCAGGAAUCCCAACAAACCUGGAGAUGAGUAUCUGUAUGGAGGUGGGUGCGCAGAGACUGACGAAGACCAUGCACACUGGCUUGAA
KX766028        CCUAAAGCCGGUCAUACUUGAUGGCGAGAGAGUCAUUCUGGCUGGACCCAUGCCUGUCACACAUGCCAGCGCUGCCCAGAGGAGGGGGCGCAUAGGCAGGAAUCCCAACAAACCUGGAGAUGAGUAUCUGUAUGGAGGUGGGUGCGCAGAGACUGACGAAGACCAUGCACACUGGCUUGAA
KX766029        CCUAAAGCCGGUCAUACUUGAUGGCGAGAGAGUCAUUCUGGCUGGACCCAUGCCUGUCACACAUGCCAGCGCUGCCCAGAGGAGGGGGCGCAUAGGCAGGAAUCCCAACAAACCUGGAGAUGAGUAUCUGUAUGGAGGUGGGUGCGCAGAGACUGACGAAGACCAUGCACACUGGCUUGAA
KU940224        CCUAAAGCCGGUCAUACUUGAUGGCGAGAGAGUCAUUCUGGCUGGACCCAUGCCUGUCACACAUGCCAGCGCUGCCCAGAGGAGGGGGCGCAUAGGCAGGAAUCCCAACAAACCUGGAGAUGAGUAUCUGUAUGGAGGUGGGUGCGCAGAGACUGACGAAGACCAUGCACACUGGCUUGAA
```

```
                555555555555555555555555556666666666666666666666666666666666666666666666666666666666666666666666666666666666666666666666666666666666666666666666666666666666666666666666666666666666666666666666666
                999999999999999999999999990000000000000000000000000000000000000000000000000000000000000000000000000000000000000000000000000000111111111111111111111111111111111111111111111111111111111111111111111111111
                777777888888888899999999990000000000111111111122222222223333333333444444444455555555556666666666777777777788888888889999999999000000000011111111112222222222333333333344444444445555555
Sequence        456789012345678901234567890123456789012345678901234567890123456789012345678901234567890123456789012345678901234567890123456789012345678901234567890123456789012345678901234567890123456789012345678901234
-----------------------------------------------------------------------------------------------------------------------------------------------------------------------------------------------------------------------------
NC_012532       GCAAGAAUGCUUCUUGACAACAUCUACCUCCAGGAUGGCCUCAUAGCCUCGCUCUAUCGGCCUGAGGCCGAUAAGGUAGCCGCCAUUGAGGGAGAGUUUAAGCUGAGGACAGAGCAAAGGAAGACCUUCGUGGAACUCAUGAAGAGAGGAGACCUUCCCGUCUGGCUAGCCUAUCAGGUUG
KU922960        GCAAGAAUGCUCCUUGACAAUAUUUACCUCCAAGAUGGCCUCAUAGCCUCGCUCUAUCGACCUGAGGCCGACAAAGUAGCAGCCAUUGAGGGAGAGUUCAAGCUUAGGACGGAGCAAAGGAAGACCUUUGUGGAACUCAUGAAAAGAGGAGAUCUUCCUGUUUGGCUGGCCUAUCAGGUUG
KU922923        GCAAGAAUGCUCCUUGACAAUAUUUACCUCCAAGAUGGCCUCAUAGCCUCGCUCUAUCGACCUGAGGCCGACAAAGUAGCAGCCAUUGAGGGAGAGUUCAAGCUUAGGACGGAGCAAAGGAAGACCUUUGUGGAACUCAUGAAAAGAGGAGAUCUUCCUGUUUGGCUGGCCUAUCAGGUUG
KU820898        GCAAGAAUGCUCCUUGACAAUAUUUACCUCCAAGAUGGCCUCAUAGCCUCGCUCUAUCGACCUGAGGCCGACAAAGUAGCAGCCAUUGAGGGAGAGUUCAAGCUUAGGACGGAGCAAAGGAAGACCUUUGUGGAACUCAUGAAAAGAGGAGAUCUUCCUGUUUGGCUGGCCUAUCAGGUUG
KU321639        GCAAGAAUGCUCCUUGACAAUAUUUACCUCCAAGAUGGCCUCAUAGCCUCGCUCUAUCGACCUGAGGCCGACAAAGUAGCAGCCAUUGAGGGAGAGUUCAAGCUUAGGACGGAGCAAAGGAAGACCUUUGUGGAACUCAUGAAAAGAGGAGAUCUUCCUGUUUGGCUGGCCUAUCAGGUUG
KU820899        GCAAGAAUGCUCCUUGACAAUAUUUACCUCCAAGAUGGCCUCAUAGCCUCGCUCUAUCGACCUGAGGCCGACAAAGUAGCAGCCAUUGAGGGAGAGUUCAAGCUUAGGACGGAGCAAAGGAAGACCUUUGUGGAACUCAUGAAAAGAGGAGAUCUUCCUGUUUGGCUGGCCUAUCAGGUUG
KU853012        GCAAGAAUGCUCCUUGACAAUAUUUACCUCCAAGAUGGCCUCAUAGCCUCGCUCUAUCGACCUGAGGCCGACAAAGUAGCAGCCAUUGAGGGAGAGUUCAAGCUUAGGACGGAGCAAAGGAAGACCUUUGUGGAACUCAUGAAAAGAGGAGAUCUUCCUGUUUGGCUGGCCUAUCAGGUUG
KU729217        GCAAGAAUGCUCCUUGACAAUAUUUACCUCCAAGAUGGCCUCAUAGCCUCGCUCUAUCGACCUGAGGCCGACAAAGUAGCAGCCAUUGAGGGAGAGUUCAAGCUUAGGACGGAGCAAAGGAAGACCUUUGUGGAACUCAUGAAAAGAGGAGAUCUUCCUGUUUGGCUGGCCUAUCAGGUUG
KU729218        GCAAGAAUGCUCCUUGACAAUAUUUACCUCCAAGAUGGCCUCAUAGCCUCGCUCUAUCGACCUGAGGCCGACAAAGUAGCAGCCAUUGAGGGAGAGUUCAAGCUUAGGACGGAGCAAAGGAAGACCUUUGUGGAACUCAUGAAAAGAGGAGAUCUUCCUGUUUGGCUGGCCUAUCAGGUUG
KU761564        GCAAGAAUGCUCCUUGACAAUAUUUACCUCCAAGAUGGCCUCAUAGCCUCGCUCUAUCGACCUGAGGCCGACAAAGUAGCAGCCAUUGAGGGAGAGUUCAAGCUUAGGACGGAGCAAAGGAAGACCUUUGUGGAACUCAUGAAAAGAGGAGAUCUUCCUGUUUGGCUGGCCUAUCAGGUUG
KU740184        GCAAGAAUGCUCCUUGACAAUAUUUACCUCCAAGAUGGCCUCAUAGCCUCGCUCUAUCGACCUGAGGCCGACAAAGUAGCAGCCAUUGAGGGAGAGUUCAAGCUUAGGACGGAGCAAAGGAAGACCUUUGUGGAACUCAUGAAAAGAGGAGAUCUUCCUGUUUGGCUGGCCUAUCAGGUUG
KU744693        GCAAGAAUGCUCCUUGACAAUAUUUACCUCCAAGAUGGCCUCAUAGCCUCGCUCUAUCGACCUGAGGCCGACAAAGUAGCAGCCAUUGAGGGAGAGUUCAAGCUUAGGACGGAGCAAAGGAAGACCUUUGUGGAACUCAUGAAAAGAGGAGAUCUUCCUGUUUGGCUGGCCUAUCAGGUUG
KU497555        GCAAGAAUGCUCCUUGACAAUAUUUACCUCCAAGAUGGCCUCAUAGCCUCGCUCUAUCGACCUGAGGCCGACAAAGUAGCAGCCAUUGAGGGAGAGUUCAAGCUUAGGACGGAGCAAAGGAAGACCUUUGUGGAACUCAUGAAAAGAGGAGAUCUUCCUGUUUGGCUGGCCUAUCAGGUUG
KU527068        GCAAGAAUGCUCCUUGACAAUAUUUACCUCCAAGAUGGCCUCAUAGCCUCGCUCUAUCGACCUGAGGCCGACAAAGUAGCAGCCAUUGAGGGAGAGUUCAAGCUUAGGACGGAGCAAAGGAAGACCUUUGUGGAACUCAUGAAAAGAGGAGAUCUUCCUGUUUGGCUGGCCUAUCAGGUUG
KU501217        GCAAGAAUGCUCCUUGACAAUAUUUACCUCCAAGAUGGCCUCAUAGCCUCGCUCUAUCGACCUGAGGCCGACAAAGUAGCAGCCAUUGAGGGAGAGUUCAAGCUUAGGACGGAGCAAAGGAAGACCUUUGUGGAACUCAUGAAAAGAGGAGAUCUUCCUGUUUGGCUGGCCUAUCAGGUUG
KU501216        GCAAGAAUGCUCCUUGACAAUAUUUACCUCCAAGAUGGCCUCAUAGCCUCGCUCUAUCGACCUGAGGCCGACAAAGUAGCAGCCAUUGAGGGAGAGUUCAAGCUUAGGACGGAGCAAAGGAAGACCUUUGUGGAACUCAUGAAAAGAGGAGAUCUUCCUGUUUGGCUGGCCUAUCAGGUUG
KU501215        GCAAGAAUGCUCCUUGACAAUAUUUACCUCCAAGAUGGCCUCAUAGCCUCGCUCUAUCGACCUGAGGCCGACAAAGUAGCAGCCAUUGAGGGAGAGUUCAAGCUUAGGACGGAGCAAAGGAAGACCUUUGUGGAACUCAUGAAAAGAGGAGAUCUUCCUGUUUGGCUGGCCUAUCAGGUUG
KU365780        GCAAGAAUGCUCCUUGACAAUAUUUACCUCCAAGAUGGCCUCAUAGCCUCGCUCUAUCGACCUGAGGCCGACAAAGUAGCAGCCAUUGAGGGAGAGUUCAAGCUUAGGACGGAGCAAAGGAAGACCUUUGUGGAACUCAUGAAAAGAGGAGAUCUUCCUGUUUGGCUGGCCUAUCAGGUUG
```

```
KU365779      GCAAGAAUGCUCCUUGACAAUAUUUACCUCCAAGAUGGCCUCAUAGCCUCGCUCUAUCGACCUGAGGCCGACAAAGUAGCAGCCAUUGAGGGAGAGUUCAAGCUUAGGACGGAGCAAAGGAAGACCUUUGUGGAACUCAUGAAAAGAGGAGAUCUUCCUGUUUGGCUGGCCUAUCAGGUUG
KU707826      GCAAGAAUGCUCCUUGACAAUAUUUACCUCCAAGAUGGCCUCAUAGCCUCGCUCUAUCGACCUGAGGCCGACAAAGUAGCAGCCAUUGAGGGAGAGUUCAAGCUUAGGACGGAGCAAAGGAAGACCUUUGUGGAACUCAUGAAAAGAGGAGAUCUUCCUGUUUGGCUGGCCUAUCAGGUUG
KU365778      GCAAGAAUGCUCCUUGACAAUAUUUACCUCCAAGAUGGCCUCAUAGCCUCGCUCUAUCGACCUGAGGCCGACAAAGUAGCAGCCAUUGAGGGAGAGUUCAAGCUUAGGACGGAGCAAAGGAAGACCUUUGUGGAACUCAUGAAAAGAGGAGAUCUUCCUGUUUGGCUGGCCUAUCAGGUUG
KU365777      GCAAGAAUGCUCCUUGACAAUAUUUACCUCCAAGAUGGCCUCAUAGCCUCGCUCUAUCGACCUGAGGCCGACAAAGUAGCAGCCAUUGAGGGAGAGUUCAAGCUUAGGACGGAGCAAAGGAAGACCUUUGUGGAACUCAUGAAAAGAGGAGAUCUUCCUGUUUGGCUGGCCUAUCAGGUUG
KU312312      GCAAGAAUGCUCCUUGACAAUAUUUACCUCCAAGAUGGCCUCAUAGCCUCGCUCUAUCGACCUGAGGCCGACAAAGUAGCAGCCAUUGAGGGAGAGUUCAAGCUUAGGACGGAGCAAAGGAAGACCUUUGUGGAACUCAUGAAAAGAGGAGAUCUUCCUGUUUGGCUGGCCUAUCAGGUUG
KF268950      GCAAGAAUGCUUCUUGACAACAUCUACCUCCAGGAUGGCCUCAUAGCCUCGCUCUACCGGCCUGAGGCCGAUAAGGUAGCUGCCAUUGAGGGAGAGUUCAAGCUGAGGACAGAGCAAAGGAAAACCUUUGUGGAACUUAUGAAGAGAGGAGACCUUCCCGUUUGGCUAGCCUAUCAGGUUG
KF268949      GCAAGAAUGCUUCUUGACAACAUCUACCUCCAGGAUGGCCUCAUAGCUUCGCUCUACCGGCCUGAGGCCGACAAGGUAGCCGCCAUUGAGGGAGAGUUCAAGCUGAGGACGGAGCAAAGGAAAACCUUUGUGGAACUUAUGAAGAGAGGAGACCUUCCCGUUUGGCUGGCCUAUCAGGUUG
KF268948      GCAAGAAUGCUUCUUGACAACAUCUACCUCCAGGAUGGCCUCAUAGCCUCGCUCUACCGGCCUGAGGCCGAUAAGGUAGCUGCCAUUGAGGGAGAGUUCAAGCUGAGGACAGAGCAAAGGAAAACCUUUGUGGAACUUAUGAAGAGAGGAGACCUUCCCGUUUGGCUAGCCUAUCAGGUUG
KJ776791      GCAAGAAUGCUCCUUGACAAUAUUUACCUCCAAGAUGGCCUCAUAGCCUCGCUCUAUCGACCUGAGGCCGACAAAGUAGCAGCCAUUGAGGGAGAGUUCAAGCUUAGGACGGAGCAAAGGAAGACCUUUGUGGAACUCAUGAAAAGAGGAGAUCUUCCUGUUUGGCUGGCCUAUCAGGUUG
KF383119      GCAAGAAUGCUUCUUGACAACAUCUACCUCCAGGAUGGCCUCAUAGCCUCGCUCUAUCGGCCUGAGGCCGAUAAGGUAGCCGCCAUUGAGGGAGAGUUUAAGCUGAGGACAGAGCAAAGGAAGACCUUCGUGGAACUCAUGAAGAGAGGAGACCUUCCCGUCUGGCUAGCCUAUCAGGUUG
KF383118      GCAAGAAUGCUUCUUGACAACAUCUACCUCCAGGAUGGCCUCAUAGCCUCGCUCUAUCGGCCUGAGGCCGAUAAGGUAGCCGCCAUUGAGGGAGAGUUUAAGCUGAGGACAGAGCAAAGGAAGACCUUCGUGGAACUCAUGAAGAGAGGAGACCUUCCCGUCUGGCUAGCCUAUCAGGUUG
KF383117      GCAAGAAUGCUUCUUGACAACAUUUACCUCCAAGAUGGCCUCAUAGCCUCGCUCUAUCGGCCUGAGGCCGACAAGGUAGCCGCCAUUGAGGGAGAGUUCAAGCUGAGGACAGAGCAAAGGAAGACCUUUGUGGAACUCAUGAAGAGAGGAGACCUUCCCGUUUGGCUGGCCUAUCAAGUAG
KF383116      GCAAGAAUGCUUCUUGACAACAUUUACCUCCAGGAUGGCCUCAUAGCCUCGCUCUAUCGGCCUGAGGCCGACAAGGUAGCCGCCAUUGAGGGAGAGUUCAAGCUGAGGACAGAGCAAAGGAAGACCUUUGUGGAACUCAUGAAGAGAGGAGACCUUCCCGUUUGGCUGGCCUAUCAAGUAG
KF383115      GCAAGAAUGCUUCUUGACAACAUCUACCUCCAGGAUGGCCUCAUAGCCUCGCUCUAUCGGCCUGAGGCCGAUAAGGUGGCCGCCAUUGAGGGAGAGUUUAAGCUGAGGACAGAGCAAAGGAAGACCUUUGUGGAACUUAUGAAGAGAGGAGACCUUCCCGUUUGGCUAGCCUAUCAGGUUG
EU545988      GCAAGAAUGCUUCUUGACAACAUUUACCUCCAAGAUGGCCUCAUAGCCUCGCUCUAUCGACCUGAGGCCGACAAAGUAGCAGCUAUUGAGGGAGAGUUCAAGCUUAGGACGGAGCAAAGGAAGACCUUUGUGGAACUCAUGAAAAGAGGAGAUCUUCCUGUUUGGCUGGCCUAUCAGGUUG
KU955589      GCAAGAAUGCUCCUUGACAAUAUUUACCUCCAAGAUGGCCUCAUAGCCUCGCUCUAUCGACCUGAGGCCGACAAAGUAGCAGCCAUUGAGGGAGAGUUCAAGCUUAGGACGGAGCAAAGGAAGACCUUUGUGGAACUCAUGAAAAGAGGAGAUCUUCCUGUUUGGCUGGCCUAUCAGGUUG
KU955590      GCAAGAAUGCUCCUUGACAAUAUUUACCUCCAAGAUGGCCUCAUAGCCUCGCUCUAUCGACCUGAGGCCGACAAAGUAGCAGCCAUUGAGGGAGAGUUCAAGCUUAGGACGGAGCAAAGGAAGACCUUUGUGGAACUCAUGAAAAGAGGAGAUCUUCCUGUUUGGCUGGCCUAUCAGGUUG
KU940228      GCAAGAAUGCUCCUUGACAAUAUUUACCUCCAAGAUGGCCUCAUAGCCUCGCUCUAUCGACCUGAGGCCGACAAAGUAGCAGCCAUUGAGGGAGAGUUCAAGCUUAGGACGGAGCAAAGGAAGACCUUUGUGGAACUCAUGAAAAGAGGAGAUCUUCCUGUUUGGCUGGCCUAUCAGGUUG
KU991811      GCAAGAAUGCUCCUUGACAAUAUUUACCUCCAAGAUGGCCUCAUAGCCUCGCUCUAUCGACCUGAGGCCGACAAAGUAGCAGCCAUUGAGGGAGAGUUCAAGCUUAGGACGGAGCAAAGGAAGACCUUUGUGGAACUCAUGAAAAGAGGAGAUCUUCCUGUUUGGCUGGCCUAUCAGGUUG
KU509998      GCAAGAAUGCUCCUUGACAAUAUUUACCUCCAAGAUGGCCUCAUAGCCUCGCUCUAUCGACCUGAGGCCGACAAAGUAGCAGCCAUUGAGGGAGAGUUCAAGCUUAGGACGGAGCAAAGGAAGACCUUUGUGGAACUCAUGAAAAGAGGAGAUCUUCCUGUUUGGCUGGCCUAUCAGGUUG
KX056898      GCAAGAAUGCUCCUUGACAAUAUUUACCUCCAAGAUGGCCUCAUAGCCUCGCUCUAUCGACCUGAGGCCGACAAAGUAGCAGCCAUUGAGGGAGAGUUCAAGCUUAGGACGGAGCAAAGGAAGACCUUUGUGGAACUCAUGAAAAGAGGAGAUCUUCCUGUUUGGCUGGCCUAUCAGGUUG
KX117076      GCAAGAAUGCUCCUUGACAAUAUUUACCUCCAAGAUGGCCUCAUAGCCUCGCUCUAUCGACCUGAGGCCGACAAAGUAGCAGCCAUUGAGGGAGAGUUCAAGCUUAGGACGGAGCAAAGGAAGACCUUUGUGGAACUCAUGAAAAGAGGAGAUCUUCCUGUUUGGCUGGCCUAUCAGGUUG
KX185891      GCAAGAAUGCUCCUUGACAAUAUUUACCUCCAAGAUGGCCUCAUAGCCUCGCUCUAUCGACCUGAGGCCGACAAAGUAGCAGCCAUUGAGGGAGAGUUCAAGCUUAGGACGGAGCAAAGGAAGACCUUUGUGGAACUCAUGAAAAGAGGAGAUCUUCCUGUUUGGCUGGCCUAUCAGGUUG
KX247646      GCAAGAAUGCUCCUUGACAAUAUUUACCUCCAAGAUGGCCUCAUAGCCUCGCUCUAUCGACCUGAGGCCGACAAAGUAGCAGCCAUUGAGGGAGAGUUCAAGCUUAGGACGGAGCAAAGGAAGACCUUUGUGGAACUCAUGAAAAGAGGAGAUCUUCCUGUUUGGCUGGCCUAUCAGGUUG
KX262887      GCAAGAAUGCUCCUUGACAAUAUUUACCUCCAAGAUGGCCUCAUAGCCUCGCUCUAUCGACCUGAGGCCGACAAAGUAGCAGCCAUUGAGGGAGAGUUCAAGCUUAGGACGGAGCAAAGGAAGACCUUUGUGGAACUCAUGAAAAGAGGAGAUCUUCCUGUUUGGCUGGCCUAUCAGGUUG
KX280026      GCAAGAAUGCUCCUUGACAAUAUUUACCUCCAAGAUGGCCUCAUAGCCUCGCUCUAUCGACCUGAGGCCGACAAAGUAGCAGCCAUUGAGGGAGAGUUCAAGCUUAGGACGGAGCAAAGGAAGACCUUUGUGGAACUCAUGAAAAGAGGAGAUCUUCCUGUUUGGCUGGCCUAUCAGGUUG
KU758877      GCAAGAAUGCUCCUUGACAAUAUUUACCUCCAAGAUGGCCUCAUAGCCUCGCUCUAUCGACCUGAGGCCGACAAAGUAGCAGCCAUUGAGGGAGAGUUCAAGCUUAGGACGGAGCAAAGGAAGACCUUUGUGGAACUCAUGAAAAGAGGAGAUCUUCCUGUUUGGCUGGCCUAUCAGGUUG
KU866423      GCAAGAAUGCUCCUUGACAAUAUUUACCUCCAAGAUGGCCUCAUAGCCUCGCUCUAUCGACCUGAGGCCGACAAAGUAGCAGCCAUUGAGGGAGAGUUCAAGCUUAGGACGGAGCAAAGGAAGACCUUUGUGGAACUCAUGAAAAGAGGAGAUCUUCCUGUUUGGCUGGCCUAUCAGGUUG
KX377336      GCAAGAAUGCUUCUUGAUAACAUUUACCUCCAAGAUGGCCUCAUAGCCUCGCUCUAUCGACCUGAGGCCGAUAAGGUAGCAGCCAUUGAGGGAGAGUUCAAGCUUAGGACGGAGCAAAGGAAGACCUUUGUGGAACUCAUGAAAAGAGGAGAUCUUCCUGUUUGGCUGGCCUAUCAGGUUG
KX377337      GCAAGAAUGCUCCUUGACAAUAUUUACCUCCAAGAUGGCCUCAUAGCCUCGCUCUAUCGACCUGAGGCCGACAAAGUAGCAGCCAUUGAGGGAGAGUUCAAGCUUAGGACGGAGCAAAGGAAGACCUUUGUGGAACUCAUGAAAAGAGGAGAUCUUCCUGUUUGGCUGGCCUAUCAGGUUG
KX369547      GCAAGAAUGCUCCUUGACAAUAUUUACCUCCAAGAUGGCCUCAUAGCCUCGCUCUAUCGACCUGAGGCCGACAAAGUAGCAGCCAUUGAGGGAGAGUUCAAGCUUAGGACGGAGCAAAGGAAGACCUUUGUGGAACUCAUGAAAAGAGGAGAUCUUCCUGUUUGGCUGGCCUAUCAGGUUG
KX548902      GCAAGAAUGCUCCUUGACAAUAUUUACCUCCAAGAUGGCCUCAUAGCCUCGCUCUAUCGACCUGAGGCCGACAAAGUAGCAGCCAUUGAGGGAGAGUUCAAGCUUAGGACGGAGCAAAGGAAGACCUUUGUGGAACUCAUGAAAAGAGGAGAUCUUCCUGUUUGGCUGGCCUAUCAGGUUG
KX601166      GCAAGAAUGCUUCUUGACAACAUUUACCUCCAGGAUGGCCUCAUAGCCUCGCUCUAUCGGCCUGAGGCCGACAAGGUAGCCGCCAUUGAGGGAGAGUUCAAGCUGAGGACAGAGCAAAGGAAGACCUUUGUGGAACUCAUGAAGAGAGGAGACCUUCCCGUUUGGCUGGCCUAUCAAGUAG
KX601167      GCAAGAAUGCUUCUUGAUAACAUUUACCUCCAAGAUGGCCUCAUAGCCUCGCUCUAUCGACCUGAGGCCGAUAAGGUAGCAGCCAUUGAGGGAGAGUUCAAGCUUAGGACGGAGCAAAGGAAGACCUUUGUGGAACUCAUGAAAAGAGGAGAUCUUCCUGUUUGGCUGGCCUAUCAGGUUG
KX601168      GCAAGAAUGCUCCUUGACAAUAUUUACCUCCAAGAUGGCCUCAUAGCCUCGCUCUAUCGACCUGAGGCCGACAAAGUAGCAGCCAUUGAGGGAGAGUUCAAGCUUAGGACGGAGCAAAGGAAGACCUUUGUGGAACUCAUGAAAAGAGGAGAUCUUCCUGUUUGGCUGGCCUAUCAGGUUG
KX766028      GCAAGAAUGCUCCUUGACAAUAUUUACCUCCAAGAUGGCCUCAUAGCCUCGCUCUAUCGACCUGAGGCCGACAAAGUAGCAGCCAUUGAGGGAGAGUUCAAGCUUAGGACGGAGCAAAGGAAGACCUUUGUGGAACUCAUGAAAAGAGGAGAUCUUCCUGUUUGGCUGGCCUAUCAGGUUG
KX766029      GCAAGAAUGCUCCUUGACAAUAUUUACCUCCAAGAUGGCCUCAUAGCCUCGCUCUAUCGACCUGAGGCCGACAAAGUAGCAGCCAUUGAGGGAGAGUUCAAGCUUAGGACGGAGCAAAGGAAGACCUUUGUGGAACUCAUGAAAAGAGGAGAUCUUCCUGUUUGGCUGGCCUAUCAGGUUG
KU940224      GCAAGAAUGCUCCUUGACAAUAUUUACCUCCAAGAUGGCCUCAUAGCCUCGCUCUAUCGACCUGAGGCCGACAAAGUAGCAGCCAUUGAGGGAGAGUUCAAGCUUAGGACGGAGCAAAGGAAGACCUUUGUGGAACUCAUGAAAAGAGGAGAUCUUCCUGUUUGGCUGGCCUAUCAGGUUG


              6666666666666666666666666666666666666666666666666666666666666666666666666666666666666666666666666666666666666666666666666666666666666666666666666666666666666666666666666666666
              1111111111111111111111111111111111111111111112222222222222222222222222222222222222222222222222222222222222222222222222222222222222222222222222223333333333333333333333333333333333
              5555566666666667777777777888888888899999999990000000000111111111122222222223333333333444444444455555555556666666666777777777788888888889999999999000000000011111111112222222222333333
Sequence      5678901234567890123456789012345678901234567890123456789012345678901234567890123456789012345678901234567890123456789012345678901234567890123456789012345678901234567890123456789012345
-------------------------------------------------------------------------------------------------------------------------------------------------------------------------------------------------
NC_012532     CAUCUGCCGGAAUAACUUACACAGACAGAAGAUGGUGCUUUGAUGGCACAACCAACAACACCAUAAUGGAAGACAGUGUACCAGCAGAGGUUUGGACAAAGUAUGGAGAGAAGAGAGUGCUCAAACCGAGAUGGAUGGAUGCUAGGGUCUGUUCAGACCAUGCGGCCCUGAAGUCGUUCAA
KU922960      CAUCUGCCGGAAUAACCUACACAGAUAGAAGAUGGUGCUUUGAUGGCACGACCAACAACACCAUAAUGGAAGACAGUGUGCCGGCAGAGGUGUGGACCAGACACGGAGAGAAAAGAGUGCUCAAACCGAGGUGGAUGGACGCCAGAGUUUGUUCAGAUCAUGCGGCCCUGAAGUCAUUCAA
KU922923      CAUCUGCCGGAAUAACCUACACAGAUAGAAGAUGGUGCUUUGAUGGCACGACCAACAACACCAUAAUGGAAGACAGUGUGCCGGCAGAGGUGUGGACCAGACACGGAGAGAAAAGAGUGCUCAAACCGAGGUGGAUGGACGCCAGAGUUUGUUCAGAUCAUGCGGCCCUGAAGUCAUUCAA
KU820898      CAUCUGCCGGAAUAACCUACACAGAUAGAAGAUGGUGCUUUGAUGGCACGACCAACAACACCAUAAUGGAAGACAGUGUGCCGGCAGAGGUGUGGACCAGACACGGAGAGAAAAGAGUGCUCAAACCGAGGUGGAUGGACGCCAGAGUUUGUUCAGAUCAUGCGGCCCUGAAGUCAUUCAA
KU321639      CAUCUGCCGGAAUAACCUACACAGAUAGAAGAUGGUGCUUUGAUGGCACGACCAACAACACCAUAAUGGAAGACAGUGUGCCGGCAGAGGUGUGGACCAGACACGGAGAGAAAAGAGUGCUCAAACCGAGGUGGAUGGACGCCAGAGUUUGUUCAGAUCAUGCGGCCCUGAAGUCAUUCAA
KU820899      CAUCUGCCGGAAUAACCUACACAGAUAGAAGAUGGUGCUUUGAUGGCACGACCAACAACACCAUAAUGGAAGACAGUGUGCCGGCAGAGGUGUGGACCAGACACGGAGAGAAAAGAGUGCUCAAACCGAGGUGGAUGGACGCCAGAGUUUGUUCAGAUCACGCGGCCCUGAAGUCAUUCAA
KU853012      CAUCUGCCGGAAUAACUUACACAGAUAGAAGAUGGUGCUUUGAUGGCACGACCAACAACACCAUAAUGGAAGACAGUGUGCCGGCAGAGGUGUGGACCAGACACGGAGAGAAAAGAGUGCUCAAACCGAGGUGGAUGGACGCCAGAGUUUGUUCAGAUCAUGCGGCCCUGAAGUCAUUCAA
KU729217      CAUCUGCCGGAAUAACCUACACAGAUAGAAGAUGGUGCUUUGAUGGCACGACCAACAACACCAUAAUGGAAGACAGUGUGCCGGCAGAGGUGUGGACCAGACACGGAGAGAAAAGAGUGCUCAAACCGAGGUGGAUGGACGCCAGAGUUUGUUCAGAUCAUGCGGCCCUGAAGUCAUUCAA
KU729218      CAUCUGCCGGAAUAACCUACACAGAUAGAAGAUGGUGCUUUGAUGGCACGACCAACAACACCAUAAUGGAAGACAGUGUGCCGGCAGAGGUGUGGACCAGACACGGAGAGAAAAGAGUGCUCAAACCGAGGUGGAUGGACGCCAGAGUUUGUUCAGAUCAUGCGGCCCUGAAGUCAUUCAA
KU761564      CAUCUGCCGGAAUAACCUACACAGAUAGAAGAUGGUGCUUUGAUGGCACGACCAACAACACCAUAAUGGAAGACAGUGUGCCGGCAGAGGUGUGGACCAGACACGGAGAGAAAAGAGUGCUCAAACCGAGGUGGAUGGACGCCAGAGUUUGUUCAGAUCAUGCGGCCCUGAAGUCAUUCAA
KU740184      CAUCUGCCGGAAUAACCUACACAGAUAGAAGAUGGUGCUUUGAUGGCACGACCAACAACACCAUAAUGGAAGACAGUGUGCCGGCAGAGGUGUGGACCAGACACGGAGAGAAAAGAGUGCUCAAACCGAGGUGGAUGGACGCCAGAGUUUGUUCAGAUCAUGCGGCCCUGAAGUCAUUCAA
KU744693      CAUCUGCCGGAAUAACCUACACAGAUAGAAGAUGGUGCUUUGAUGGCACGACCAACAACACCAUAAUGGAAGACAGUGUGCCGGCAGAGGUGUGGACCAGACACGGAGAGAAAAGAGUGCUCAAACCGAGGUGGAUGGACGCCAGAGUUUGUUCAGAUCAUGCGGCCCUGAAGUCAUUCAA
KU497555      CAUCUGCCGGAAUAACCUACACAGAUAGAAGAUGGUGCUUUGAUGGCACGACCAACAACACCAUAAUGGAAGACAGUGUGCCGGCAGAGGUGUGGACCAGACACGGAGAGAAAAGAGUGCUCAAACCGAGGUGGAUGGACGCCAGAGUUUGUUCAGAUCAUGCGGCCCUGAAGUCAUUCAA
KU527068      CAUCUGCCGGAAUAACCUACACAGAUAGAAGAUGGUGCUUUGAUGGCACGACCAACAACACCAUAAUGGAAGACAGUGUGCCGGCAGAGGUGUGGACCAGACACGGAGAGAAAAGAGUGCUCAAACCGAGGUGGAUGGACGCCAGAGUUUGUUCAGAUCAUGCGGCCCUGAAGUCAUUCAA
KU501217      CAUCUGCCGGAAUAACCUACACAGAUAGAAGAUGGUGCUUUGAUGGCACGACCAACAACACCAUACUGGAAGACAGUGUGCCGGCAGAGGUGUGGACCAGACACGGAGAGAAAAGAGUGCUCAAACCGAGGUGGAUGGACGCCAGAGUUUGUUCAGAUCAUGCGGCCCUGAAGUCAUUCAA
KU501216      CAUCUGCCGGAAUAACCUACACAGAUAGAAGAUGGUGCUUUGAUGGCACGACCAACAACACCAUACUGGAAGACAGUGUGCCGGCAGAGGUGUGGACCAGACACGGAGAGAAAAGAGUGCUCAAACCGAGGUGGAUGGACGCCAGAGUUUGUUCAGAUCAUGCGGCCCUGAAGUCAUUCAA
KU501215      CAUCUGCCGGAAUAACCUACACAGAUAGAAGAUGGUGCUUUGAUGGCACGACCAACAACACCAUAAUGGAAGACAGUGUGCCGGCAGAGGUGUGGACCAGACACGGAGAGAAAAGAGUGCUCAAACCGAGGUGGAUGGACGCCAGAGUUUGUUCAGAUCAUGCGGCCCUGAAGUCAUUCAA
KU365780      CAUCUGCCGGAAUAACCUACACAGAUAGAAGAUGGUGCUUUGAUGGCACGACCAACAACACCAUAAUGGAAGACAGUGUGCCGGCAGAGGUGUGGACCAGACACGGAGAGAAAAGAGUGCUCAAACCGAGGUGGAUGGACGCCAGAGUUUGUUCAGAUCAUGCGGCCCUGAAGUCAUUCAA
KU365779      CAUCUGCCGGAAUAACCUACACAGAUAGAAGAUGGUGCUUUGAUGGCACGACCAACAACACCAUAAUGGAAGACAGUGUGCCGGCAGAGGUGUGGACCAGACACGGAGAGAAAAGAGUGCUCAAACCGAGGUGGAUGGACGCCAGAGUUUGUUCAGAUCAUGCGGCCCUGAAGUCAUUCAA
KU707826      CAUCUGCCGGAAUAACCUACACAGAUAGAAGAUGGUGCUUUGAUGGCACGACCAACAACACCAUAAUGGAAGACAGUGUGCCGGCAGAGGUGUGGACCAGACACGGAGAGAAAAGAGUGCUCAAACCGAGGUGGAUGGACGCCAGAGUUUGUUCAGAUCAUGCGGCCCUGAAGUCAUUCAA
KU365778      CAUCUGCCGGAAUAACCUACACAGAUAGAAGAUGGUGCUUUGAUGGCACGACCAACAACACCAUAAUGGAAGAUAGUGUGCCGGCAGAGGUGUGGACCAGACACGGAGAGAAAAGAGUGCUCAAACCGAGGUGGAUGGACGCCAGAGUUUGUUCAGAUCAUGCGGCCCUGAAGUCAUUCAA
KU365777      CAUCUGCCGGAAUAACCUACACAGAUAGAAGAUGGUGCUUUGAUGGCACGACCAACAACACCAUAAUGGAAGACAGUGUGCCGGCAGAGGUGUGGACCAGACACGGAGAGAAAAGAGUGCUCAAACCGAGGUGGAUGGACGCCAGAGUUUGUUCAGAUCAUGCGGCCCUGAAGUCAUUCAA
KU312312      CAUCUGCCGGAAUAACCUACACAGAUAGAAGAUGGUGCUUUGAUGGCACGACCAACAACACCAUAAUGGAAGACAGUGUGCCGGCAGAAGUGUGGACCAGACACGGAGAGAAAAGAGUGCUCAAACCGAGGUGGAUGGACGCCAGAGUUUGUUCAGAUCAUGCGGCCCUGAAGUCAUUCAA
KF268950      CAUCUGCCGGAAUAACUUACACAGACAGAAGAUGGUGCUUUGAUGGCACAACCAACAACACCAUAAUGGAAGAUAGUGUACCAGCAGAAGUGUGGACAAAAUAUGGAGAGAAGAGAGUGCUCAAACCAAGAUGGAUGGAUGCGAGGGUCUGUUCCGAUCAUGCUGCCCUGAAGUCGUUCAA
KF268949      CAUCUGCCGGAAUAACUUACACAGACAGAAGAUGGUGCUUUGAUGGCACAACCAACAACACCAUAAUGGAAGAUAGUGUACCAGCAGAGGUGUGGACAAAAUAUGGAGAGAAGAGAGUGCUUAAACCAAGAUGGAUGGAUGCGAGGGUCUGUUCUGAUCAUGCUGCCCUGAAGUCGUUCAA
KF268948      CAUCUGCCGGAAUAACUUACACAGACAGAAGAUGGUGCUUUGAUGGCACAACCAACAACACCAUAAUGGAAGAUAGUGUACCAGCAGAAGUGUGGACAAAAUAUGGAGAGAAGAGAGUGCUCAAACCAAGAUGGAUGGAUGCGAGGGUCUGUUCCGAUCAUGCUGCCCUGAAGUCGUUCAA
KJ776791      CAUCUGCCGGAAUAACCUACACAGAUAGAAGAUGGUGCUUUGAUGGCACGACCAACAACACCAUAAUGGAAGACAGUGUGCCGGCAGAGGUGUGGACCAGACACGGAGAGAAAAGAGUGCUCAAACCGAGGUGGAUGGACGCCAGAGUUUGUUCAGAUCAUGCGGCCCUGAAGUCAUUCAA
KF383119      CAUCUGCCGGAAUAACUUACACAGACAGAAGAUGGUGCUUUGAUGGCACAACCAACAACACCAUAAUGGAAGACAGUGUACCAGCAGAGGUUUGGACAAAGUAUGGAGAGAAGAGAGUGCUCAAACCGAGAUGGAUGGAUGCUAGGGUCUGUUCAGACCAUGCGGCCCUGAAGUCGUUCAA
KF383118      CAUCUGCCGGAAUAACUUACACAGACAGAAGAUGGUGCUUUGAUGGCACAACCAACAACACCAUAAUGGAAGACAGCGUACCAGCAGAGGUGUGGACAAAGUAUGGAGAGAAGAGAGUGCUCAAACCGAGAUGGAUGGAUGCUAGGGUCUGUUCAGACCAUGCGGCCCUGAAGUCGUUCAA
KF383117      CAUCUGCCGGAAUAACUUACACAGACAGAAGAUGGUGCUUUGAUGGCACUACCAACAACACCAUAAUGGAAGACAGUGUACCAGCAGAGGUGUGGACCAAGUAUGGAGAGAAGAGAGUGCUUAAACCGAGGUGGAUGGAUGCUAGGGUCUGUUCAGAUCAUGCGGCUCUGAAGUCGUUCAA
KF383116      CAUCUGCCGGAAUAACUUACACAGACAGAAGAUGGUGCUUUGAUGGCACUACCAACAACACCAUAAUGGAAGACAGUGUACCAGCAGAGGUGUGGACCAAGUAUGGAGAGAAGAGAGUGCUUAAACCGAGGUGGAUGGAUGCUAGGGUCUGUUCAGAUCAUGCGGCUUUGAAGUCGUUCAA
KF383115      CAUCUGCCGGAAUAACUUACACAGACAGAAGAUGGUGCUUUGAUGGCACAACUAACAACACCAUAAUGGAAGACAGUGUACCAGCAGAGGUGUGGACAAAAUAUGGAGAGAAGAGAGUGCUCAAACCAAGAUGGAUGGAUGCUAGAGUCUGUUCAGAUCAUGCCGCCCUGAAGUCGUUCAA
EU545988      CAUCUGCCGGAAUAACCUACACAGAUAGAAAAUGGUGCUUUGAUGGCACGACCAACAACACCAUAAUGGAAGACAGUGUGCCGGCAGAGGUGUGGACCAGAUACGGAGAGAAAAGAGUGCUCAAACCAAGGUGGAUGGACGCCAGAGUUUGUUCAGAUCAUGCGGCCCUGAAGUCAUUCAA
```

```
KU955589    CAUCUGCCGGAAUAACCUACACAGAUAGAAGAUGGUGCUUUGAUGGCACGACCAACAACACCAUAAUGGAAGACAGUGUGCCGGCAGAGGUGUGGACCAGACACGGAGAGAAAAGAGUGCUCAAACCGAGGUGGAUGGACGCCAGAGUUUGUUCAGAUCACGCGGCCCUGAAGUCAUUCAA
KU955590    CAUCUGCCGGAAUAACCUACACAGAUAGAAGAUGGUGCUUUGAUGGCACGACCAACAACACCAUAAUGGAAGACAGUGUGCCGGCAGAGGUGUGGACCAGACACGGAGAGAAAAGAGUGCUCAAACCGAGGUGGAUGGACGCCAGAGUUUGUUCAGAUCAUGCGGCCCUGAAGUCAUUCAA
KU940228    CAUCUGCCGGAAUAACCUACACAGAUAGAAGAUGGUGCUUUGAUGGCACGACCAACAACACCAUAAUGGAAGACAGUGUGCCGGCAGAGGUGUGGACCAGACACGGAGAGAAAAGAGUGCUCAAACCGAGGUGGAUGGAUGCCAGAGUUUGUUCAGAUCAUGCGGCCCUGAAGUCAUUCAA
KU991811    CAUCUGCCGGAAUAACCUACACAGAUAGAAGAUGGUGCUUUGAUGGCACGACCAACAACACCAUAAUGGAAGACAGUGUGCCGGCAGAGGUGUGGACCAGACACGGAGAGAAAAGAGUGCUCAAACCGAGGUGGAUGGACGCCAGAGUUUGUUCAGAUCAUGCGGCCCUGAAGUCAUUCAA
KU509998    CAUCUGCCGGAAUAACCUACACAGAUAGAAGAUGGUGCUUUGAUGGCACGACCAACAACACCAUAAUGGAAGACAGUGUGCCGGCAGAGGUGUGGACCAGACACGGAGAGAAAAGAGUGCUCAAACCGAGGUGGAUGGACGCCAGAGUUUGUUCAGAUCAUGCGGCCCUGAAGUCAUUCAA
KX056898    CAUCUGCCGGAAUAACCUACACAGAUAGAAGAUGGUGCUUUGAUGGCACGACCAACAACACCAUAAUGGAAGACAGUGUGCCGGCAGAGGUGUGGACCAGACACGGAGAGAAAAGAGUGCUCAAACCGAGGUGGAUGGACGCCAGAGUUUGUUCAGAUCAUGCGGCCCUGAAGUCAUUCAA
KX117076    CAUCUGCCGGAAUAACCUACACAGAUAGAAGAUGGUGCUUUGAUGGCACGACCAACAACACCAUAAUGGAAGACAGUGUGCCGGCAGAGGUGUGGACCAGACACGGAGAGAAAAGAGUGCUCAAACCGAGGUGGAUGGACGCCAGAGUUUGUUCAGAUCACGCGGCCCUGAAGUCAUUCAA
KX185891    CAUCUGCCGGAAUAACCUACACAGAUAGAAGAUGGUGCUUUGAUGGCACGACCAACAACACCAUAAUGGAAGACAGUGUGCCGGCAGAGGUGUGGACCAGACACGGAGAGAAAAGAGUGCUCAAACCGAGGUGGAUGGACGCCAGAGUUUGUUCAGAUCACGCGGCCCUGAAGUCAUUCAA
KX247646    CAUCUGCCGGAAUAACCUACACAGAUAGAAGAUGGUGCUUUGAUGGCACGACCAACAACACCAUAAUGGAAGACAGUGUGCCGGCAGAGGUGUGGACCAGACACGGAGAGAAAAGAGUGCUCAAACCGAGGUGGAUGGACGCCAGAGUUUGUUCAGAUCAUGCGGCCCUGAAGUCAUUCAA
KX262887    CAUCUGCCGGAAUAACCUACACAGAUAGAAGAUGGUGCUUUGAUGGCACGACCAACAACACCAUACUGGAAGACAGCGUGCCGGCAGAGGUGUGGACCAGACACGGAGAGAAAAGAGUGCUCAAACCGAGGUGGAUGGACGCCAGAGUUUGUUCAGAUCAUGCGGCCCUGAAGUCAUUCAA
KX280026    CAUCUGCCGGAAUAACCUACACAGAUAGAAGAUGGUGCUUUGAUGGCACGACCAACAAUACCAUAAUGGAAGACAGUGUGCCGGCAGAGGUGUGGACCAGACACGGAGAGAAAAGAGUGCUCAAACCGAGGUGGAUGGACGCCAGAGUUUGUUCAGAUCAUGCGGCCCUGAAGUCAUUCAA
KU758877    CAUCUGCCGGAAUAACCUACACAGAUAGAAGAUGGUGCUUUGAUGGCACGACCAACAACACCAUAAUGGAAGACAGUGUGCCGGCAGAGGUGUGGACCAGACACGGAGAGAAAAGAGUGCUCAAACCGAGGUGGAUGGACGCCAGAGUUUGUUCAGAUCAUGCGGCCCUGAAGUCAUUCAA
KU866423    CAUCUGCCGGAAUAACCUACACAGAUAGAAGAUGGUGCUUUGAUGGCACGACCAACAACACCAUAAUGGAAGACAGUGUGCCGGCAGAGGUGUGGACCAGACACGGAGAGAAAAGAGUGCUCAAACCGAGGUGGAUGGACGCCAGAGUUUGUUCAGAUCACGCGGCCCUGAAGUCAUUCAA
KX377336    CAUCUGCCGGAAUAACCUACACAGAUAGAAGAUGGUGUUUUGAUGGCACGACCAACAACACCAUAAUGGAAGACAGUGUGCCGGCAGAGGUGUGGACCAGAUACGGAGAGAAAAGAGUGCUCAAACCGAGGUGGAUGGACGCCAGAGUUUGUUCAGAUCAUGCGGCCCUGAAGUCAUUCAA
KX377337    CAUCUGCCGGAAUAACCUACACAGAUAGAAGAUGGUGCUUUGAUGGCACGACCAACAACACCAUAAUGGAAGACAGUGUGCCGGCAGAGGUGUGGACCAGACACGGAGAGAAAAGAGUGCUCAAACCGAGGUGGAUGGACGCCAGAGUUUGUUCAGAUCAUGCGGCCCUGAAGUCAUUCAA
KX369547    CAUCUGCCGGAAUAACCUACACAGAUAGAAGAUGGUGCUUUGAUGGCACGACCAACAACACCAUAAUGGAAGACAGUGUGCCGGCAGAGGUGUGGACCAGACACGGAGAGAAAAGAGUGCUCAAACCGAGGUGGAUGGACGCCAGAGUUUGUUCAGAUCAUGCGGCCCUGAAGUCAUUCAA
KX548902    CAUCUGCCGGAAUAACCUACACAGAUAGAAGAUGGUGCUUUGAUGGCACGACCAACAACACCAUAAUGGAAGACAGUGUGCCGGCAGAGGUGUGGACCAGACACGGAGAGAAAAGAGUGCUCAAACCGAGGUGGAUGGACGCCAGAGUUUGUUCAGAUCAUGCGGCCCUGAAGUCAUUCAA
KX601166    CAUCUGCCGGAAUAACUUACACAGAUAGAAGAUGGUGCUUUGAUGGCACUACCAACAACACCAUAAUGGAAGACAGUGUACCAGCAGAGGUGUGGACCAAGUAUGGAGAGAAGAGAGUGCUUAAACCGAGGUGGAUGGAUGCUAGGGUCUGUUCAGAUCAUGCGGCUCUGAAGUCGUUCAA
KX601167    CAUCUGCCGGAAUAACCUACACAGAUAGAAGAUGGUGUUUUGAUGGCACGACCAACAACACCAUAAUGGAAGACAGUGUGCCGGCAGAGGUGUGGACCAGAUACGGAGAGAAAAGAGUGCUCAAACCGAGGUGGAUGGACGCCAGAGUUUGUUCAGAUCAUGCGGCCCUGAAGUCAUUCAA
KX601168    CAUCUGCCGGAAUAACCUACACAGAUAGAAGAUGGUGCUUUGAUGGCACGACCAACAACACCAUAAUGGAAGACAGUGUGCCGGCAGAGGUGUGGACCAGACACGGAGAGAAAAGAGUGCUCAAACCGAGGUGGAUGGACGCCAGAGUUUGUUCAGAUCAUGCGGCCCUGAAGUCAUUCAA
KX766028    CAUCUGCCGGAAUAACCUACACAGAUAGAAGAUGGUGCUUUGAUGGCACGACCAACAACACCAUAAUGGAAGACAGUGUGCCGGCAGAGGUGUGGACCAGACAUGGAGAGAAAAGAGUGCUCAAACCGAGGUGGAUGGACGCCAGAGUUUGUUCAGAUCAUGCGGCCCUGAAGUCAUUCAA
KX766029    CAUCUGCCGGAAUAACCUACACAGAUAGAAGAUGGUGCUUUGAUGGCACGACCAACAACACCAUACUGGAAGACAGUGUGCCGGCAGAGGUGUGGACCAGACACGGAGAGAAAAGAGUGCUCAAACCGAGGUGGAUGGACGCCAGAGUUUGUUCAGAUCAUGCGGCCCUGAAGUCAUUCAA
KU940224    CAUCUGCCGGAAUAACCUACACAGAUAGAAGAUGGUGCUUUGAUGGCACGACCAACAACACCAUAAUGGAAGACAGUGUGCCGGCAGAGGUGUGGACCAGACACGGAGAGAAAAGAGUGCUCAAACCGAGGUGGAUGGAUGCCAGAGUUUGUUCAGAUCAUGCGGCCCUGAAGUCAUUCAA


            6666666666666666666666666666666666666666666666666666666666666666666666666666666666666666666666666666666666666666666666666666666666666666666666666666666666666666666666666666666
            3333333333333333333333333333333333333333333333333333333333333333444444444444444444444444444444444444444444444444444444444444444444444444444444444444444444444444455555555555555555
            3333444444444455555555556666666666777777777788888888889999999999000000000011111111112222222222333333333344444444445555555555666666666677777777778888888888999999999900000000001111111
Sequence    6789012345678901234567890123456789012345678901234567890123456789012345678901234567890123456789012345678901234567890123456789012345678901234567890123456789012345678901234567890123456
-----------------------------------------------------------------------------------------------------------------------------------------------------------------------------------------------------------
NC_012532   AGAAUUCGCCGCUGGAAAAGAGGAGCGGCUUUGGGAGUAAUGGAGGCCCUGGGAACACUGCCAGGACACAUGACAGAGAGGUUUCAGGAAGCCAUUGACAACCUCGCCGUGCUCAUGCGAGCAGAGACUGGAAGCAGGCCUUAUAAGGCAGCGGCAGCCCAACUGCCGGAGACCCUAGAG
KU922960    GGAGUUUGCCGCUGGGAAAAGAGGAGCGGCUUUUGGAGUGAUGGAAGCCCUGGGAACACUGCCAGGACACAUGACAGAGAGAUUCCAGGAAGCCAUUGACAACCUCGCUGUGCUCAUGCGGGCAGAGACUGGAAGCAGGCCUUACAAAGCCGCGGCGGCCCAAUUGCCGGAGACCCUAGAG
KU922923    GGAGUUUGCCGCUGGGAAAAGAGGAGCGGCUUUUGGAGUGAUGGAAGCCCUGGGAACACUGCCAGGACACAUGACAGAGAGAUUCCAGGAAGCCAUUGACAACCUCGCUGUGCUCAUGCGGGCAGAGACUGGAAGCAGGCCUUACAAAGCCGCGGCGGCCCAAUUGCCGGAGACCCUAGAG
KU820898    GGAGUUUGCCGCUGGGAAAAGAGGAGCGGCUUUUGGAGUGAUGGAAGCCCUGGGAACACUGCCAGGACACAUGACAGAGAGAUUCCAGGAAGCCAUUGACAACCUCGCUGUGCUCAUGCGGGCAGAGACUGGAAGCAGGCCUUACAAAGCCGCGGCAGCCCAAUUGCCGGAGACCCUAGAG
KU321639    GGAGUUUGCCGCUGGGAAAAGAGGAGCGGCUUUUGGAGUGAUGGAAGCCCUGGGAACACUGCCAGGACACAUGACAGAGAGAUUCCAGGAAGCCAUUGACAACCUCGCUGUGCUCAUGCGGGCAGAGACUGGAAGCAGGCCUUACAAAGCCGCGGCGGCCCAAUUGCCGGAGACCCUAGAG
KU820899    GGAGUUUGCCGCUGGGAAAAGAGGAGCGGCUUUUGGAGUGAUGGAAGCCUUGGGAACACUGCCAGGACACAUGACAGAGAGAUUCCAGGAAGCCAUUGACAACCUCGCUGUGCUCAUGCGGGCAGAGACUGGAAGCAGGCCUUACAAAGCCGCGGCGGCCCAAUUGCCGGAGACCCUAGAG
KU853012    GGAGUUUGCCGCUGGGAAAAGAGGAGCGGCUUUUGGAGUGAUGGAAGCCCUGGGAACACUGCCAGGACACAUGACAGAGAGAUUCCAGGAAGCCAUUGACAACCUCGCUGUGCUCAUGCGGGCAGAGACUGGAAGCAGGCCUUACAAAGCCGCGGCGGCCCAAUUGCCGGAGACCCUAGAG
KU729217    GGAGUUUGCCGCUGGGAAAAGAGGAGCGGCUUUUGGAGUGAUGGAAGCCCUGGGAACACUGCCAGGACACAUGACAGAGAGAUUCCAGGAAGCCAUUGACAACCUCGCUGUGCUCAUGCGGGCAGAGACUGGAAGCAGGCCUUACAAAGCCGCGGCGGCCCAAUUGCCGGAGACCCUAGAG
KU729218    GGAGUUUGCCGCUGGGAAAAGAGGAGCGGCUUUUGGAGUGAUGGAAGCCCUGGGAACACUGCCAGGACACAUGACAGAGAGAUUCCAGGAAGCCAUUGACAACCUCGCUGUGCUCAUGCGGGCAGAGACUGGAAGCAGGCCUUACAAAGCCGCGGCGGCCCAAUUGCCGGAGACCCUAGAG
KU761564    GGAGUUUGCCGCUGGGAAAAGAGGAGCGGCUUUUGGAGUGAUGGAAGCCCUGGGAACACUGCCAGGACACAUGACAGAGAGAUUCCAGGAAGCCAUUGACAACCUCGCUGUGCUCAUGCGGGCAGAGACUGGAAGCAGGCCUUACAAAGCCGCGGCAGCCCAAUUGCCGGAGACCCUAGAG
KU740184    GGAGUUUGCCGCUGGGAAAAGAGGAGCGGCUUUUGGAGUGAUGGAAGCCCUGGGAACACUGCCAGGACACAUGACAGAGAGAUUCCAGGAAGCCAUUGACAACCUCGCUGUGCUCAUGCGGGCAGAGACUGGAAGCAGGCCUUACAAAGCCGCGGCAGCCCAAUUGCCGGAGACCCUAGAG
KU744693    GGAGUUUGCCGCUGGGAAAAGAGGAGCGGCUUUUGGAGUGAUGGAAGCCCUGGGAACACUGCCAGGACACAUGACAGAGAGAUUCCAGGAAGCCAUUGACAACCUCGCUGUGCUCAUGCGGGCAGAGACUGGAAGCAGGCCUUACAAAGCCGCGGCGGCCCAAUUGCCGGAGACCCUAGAG
KU497555    GGAGUUUGCCGCUGGGAAAAGAGGAGCGGCUUUUGGAGUGAUGGAAGCCCUGGGAACACUGCCAGGACACAUGACAGAGAGAUUCCAGGAAGCCAUUGACAACCUCGCUGUGCUCAUGCGGGCAGAGACUGGAAGCAGGCCUUACAAAGCCGCGGCGGCCCAAUUGCCGGAGACCCUAGAG
KU527068    GGAGUUUGCCGCUGGGAAAAGAGGAGCGGCUUUUGGAGUGAUGGAAGCCCUGGGAACACUGCCAGGACACAUGACAGAGAGAUUCCAGGAAGCCAUUGACAACCUCGCUGUGCUCAUGCGGGCAGAGACUGGAAGCAGGCCUUACAAAGCCGCGGCGGCCCAAUUGCCGGAGACCCUAGAG
KU501217    GGAGUUUGCCGCUGGGAAAAGAGGAGCGGCUUUUGGAGUGAUGGAAGCCUUGGGAACACUGCCAGGACACAUGACAGAGAGAUUCCAGGAAGCCAUUGACAACCUCGCUGUGCUCAUGCGGGCAGAGACUGGAAGCAGGCCUUACAAAGCCGCGGCGGCCCAAUUGCCGGAGACCCUAGAG
KU501216    GGAGUUUGCCGCUGGGAAAAGAGGAGCGGCUUUUGGAGUGAUGGAAGCCUUGGGAACACUGCCAGGACACAUGACAGAGAGAUUCCAGGAAGCCAUUGACAACCUCGCUGUGCUCAUGCGGGCAGAGACUGGAAGCAGGCCUUACAAAGCCGCGGCGGCCCAAUUGCCGGAGACCCUAGAG
KU501215    GGAGUUUGCCGCUGGGAAAAGAGGAGCGGCUUUUGGAGUGAUGGAAGCCCUGGGAACACUGCCAGGACACAUGACAGAGAGAUUCCAGGAAGCCAUUGACAACCUCGCUGUGCUCAUGCGGGCAGAGACUGGAAGCAGGCCUUACAAAGCCGCGGCGGCCCAAUUGCCGGAGACCCUAGAG
KU365780    GGAGUUUGCCGCUGGGAAAAGAGGAGCGGCUUUUGGAGUGAUGGAAGCCCUGGGAACACUGCCAGGACACAUGACAGAGAGAUUCCAGGAAGCCAUUGACAACCUCGCUGUGCUCAUGCGGGCAGAGACUGGAAGCAGGCCUUACAAAGCCGCGGCGGCCCAAUUGCCGGAGACCCUAGAG
KU365779    GGAGUUUGCCGCUGGGAAAAGAGGAGCGGCUUUUGGAGUGAUGGAAGCCCUGGGAACACUGCCAGGACACAUGACAGAGAGAUUCCAGGAAGCCAUUGACAACCUCGCUGUGCUCAUGCGGGCAGAGACUGGAAGCAGGCCUUACAAAGCCGCGGCGGCCCAAUUGCCGGAGACCCUAGAG
KU707826    GGAGUUUGCCGCUGGGAAAAGAGGAGCGGCUUUUGGAGUGAUGGAAGCCCUGGGAACACUGCCAGGACACAUGACAGAGAGAUUCCAGGAAGCCAUUGACAACCUCGCUGUGCUCAUGCGGGCAGAGACUGGAAGCAGGCCUUACAAAGCCGCGGCGGCCCAAUUGCCGGAGACCCUAGAG
KU365778    GGAGUUUGCCGCUGGGAAAAGAGGAGCGGCUUUUGGAGUGAUGGAAGCCCUGGGAACACUGCCAGGACACAUGACAGAGAGAUUCCAGGAAGCCAUUGACAACCUCGCUGUGCUCAUGCGGGCAGAGACUGGAAGCAGGCCUUACAAAGCCGCGGCGGCCCAAUUGCCGGAGACCCUAGAG
KU365777    GGAGUUUGCCGCUGGGAAAAGAGGAGCGGCUUUUGGAGUGAUGGAAGCCCUGGGAACACUGCCAGGACACAUGACAGAGAGAUUCCAGGAAGCCAUUGACAACCUCGCUGUGCUCAUGCGGGCAGAGACUGGAAGCAGGCCUUACAAAGCCGCGGCGGCCCAAUUGCCGGAGACCCUAGAG
KU312312    GGAGUUUGCCGCUGGGAAAAGAGGAGCGGCUUUUGGAGUGAUGGAAGCCCUGGGAACACUGCCAGGACACAUGACAGAGAGAUUCCAGGAAGCCAUUGACAACCUCGCUGUGCUCAUGCGGGCAGAGACUGGAAGCAGGCCUUACAAAGCCGCGGCGGCCCAAUUGCCGGAGACCCUAGAG
KF268950    AGAAUUUGCUGCCGGGAAAAGAGGAGUGGCUUUAGGAGUAAUGGAGGCCCUGGGAACAUUGCCAGGACACAUGACAGAGAGGUUUCAGGAGGCCAUUGACAAUCUCGCUGUGCUCAUGCGAGCAGAGACUGGAAGCAGGCCUUACAAGGCAGCGGCAGCCCAACUGCCGGAGACCCUAGAG
KF268949    AGAAUUUGCUGCCGGGAAAAGAGGAGCGGCUUUAGGAGUAAUGGAGGCCCUGGGAACAUUACCAGGACACAUGACAGAGAGGUUUCAGGAGGCCAUUGACAAUCUCGCUGUGCUUAUGCGAGCAGAGACUGGAAGUAGGCCUUACAAGGCAGCGGCAGCCCAACUGCCGGAGACCCUAGAG
KF268948    AGAAUUUGCUGCCGGGAAAAGAGGAGUGGCUUUAGGAGUAAUGGAGGCCCUGGGAACAUUGCCAGGACACAUGACAGAGAGGUUUCAGGAGGCCAUUGACAAUCUCGCUGUGCUCAUGCGAGCAGAGACUGGAAGCAGGCCUUACAAGGCAGCGGCAGCCCAACUGCCGGAGACCCUAGAG
KJ776791    GGAGUUUGCCGCUGGGAAAAGAGGAGCGGCUUUUGGAGUGAUGGAAGCCCUGGGAACACUGCCAGGACACAUGACAGAGAGAUUCCAGGAAGCCAUUGACAACCUCGCUGUGCUCAUGCGGGCAGAGACUGGAAGCAGGCCUUACAAAGCCGCGGCGGCCCAAUUGCCGGAGACCCUAGAG
KF383119    AGAAUUCGCCGCUGGAAAAGAGGAGCGGCUUUGGGAGUAAUGGAGGCCCUGGGAACACUGCCAGGACACAUGACAGAGAGGUUUCAGGAAGCCAUUGACAACCUCGCCGUGCUCAUGCGAGCAGAGACUGGAAGCAGGCCUUAUAAGGCAGCGGCAGCCCAACUGCCGGAGACCCUAGAG
KF383118    AGAAUUCGCCGCUGGAAAAAGAGGAGCGGCUUUGGGAGUAAUGGAGGCCCUGGGAACACUGCCAGGACACAUGACAGAGAGGUUUCAGGAAGCCAUUGACAACCUCGCCGUGCUCAUGCGAGCAGAGACUGGAAGCAGGCCUUAUAAGGCAGCGGCAGCCCAACUGCCGGAGACCCUAGAG
KF383117    AGAAUUUGCCGCUGGGAAGAGAGGAGCGGCUUUGGGAGUAAUGGAUGCCCUGGGAACAUUGCCAGGACACAUGACAGAGAGGUUUCAGGAAGCCAUUGACAAUCUUGCUGUGCUCAUGCGAGCAGAGACUGGAAGUAGGCCCUACAAAGCAGCGGCAGCUCAACUGCCGGAGACCCUAGAG
KF383116    AGAAUUUGCCGCUGGGAAGAGAGGAGCGGCUUUGGGAGUGAUGGAUGCCCUGGGAACAUUGCCAGGACACAUGACAGAGAGGUUUCAGGAAGCCAUUGACAAUCUCGCUGUGCUCAUGCGAGCAGAGACUGGAAGUAGGCCCUACAAAGCAGCGGCAGCUCAACUGCCGGAGACCCUAGAG
KF383115    AGAGUUUGCUGCCGGGAAAAGAGGAGCGGCUUUGGGAGUAAUGGAGGCCCUGGGAACAUUGCCAGGACACAUGACAGAGAGGUUUCAGGAAGCCAUUGACAAUCUCGCUGUGCUUAUGCGAGCAGAAACUGGAAGCAGGCCUUACAAGGCAGCGGCAGCCCCACUGCCGGAGACCCUAGAG
EU545988    AGAGUUUGCCGCUGGGAAAAGAGGAGYGGCCUUUGGAGUGAUGGAAGCCCUGGGAACAUUGCCGGGACACAUGACAGAGAGAUUCCAGGAAGCCAUUGACAACCUCGCUGUGCUCAUGCGGGCAGAGACUGGAAGCAGGCCUUACGAAGCCGCGGCGGCCCAAUUGCCGGAGACCUUAGAG
KU955589    GGAGUUUGCCGCUGGGAAAAGAGGAGCGGCUUUUGGAGUGAUGGAAGCCUUGGGAACACUGCCAGGACACAUGACAGAGAGAUUCCAGGAAGCCAUUGACAACCUCGCUGUGCUCAUGCGGGCAGAGACUGGAAGCAGGCCUUACAAAGCCGCGGCGGCCCAAUUGCCGGAGACCCUAGAG
KU955590    GGAGUUUGCCGCUGGGAAAAGAGGAGCGGCUUUUGGAGUGAUGGAAGCCCUGGGAACACUGCCAGGACACAUGACAGAGAGAUUCCAGGAAGCCAUUGACAACCUCGCUGUGCUCAUGCGGGCAGAGACUGGAAGCAGGCCUUACAAAGCCGCGGCAGCCCAAUUGCCGGAGACCCUAGAG
KU940228    GGAGUUUGCCGCUGGGAAAAGAGGAGCGGCUCUUGGAGUGAUGGAAGCCCUGGGAACACUGCCAGGACACAUGACAGAGAGAUUCCAGGAAGCCAUUGACAACCUCGCUGUGCUCAUGCGGGCAGAGACUGGAAGCAGGCCUUACAAAGCCGCGGCGGCCCAAAUGCCGGAGACCCUAGAG
KU991811    GGAGUUUGCCGCUGGGAAAAGAGGAGCGGCUUUUGGAGUGAUGGAAGCCCUGGGAACACUGCCAGGACACAUGACAGAGAGAUUCCAGGAAGCCAUUGACAACCUCGCUGUGCUCAUGCGGGCAGAGACUGGAAGCAGGCCUUACAAAGCCGCGGCGGCCCAAUUGCCGGAGACCCUAGAG
KU509998    GGAGUUUGCCGCUGGGAAAAGAGGAGCGGCUUUUGGAGUGAUGGAAGCCCUGGGAACACUGCCAGGACACAUGACAGAGAGAUUCCAGGAAGCCAUUGACAACCUCGCUGUGCUCAUGCGGGCAGAGACUGGAAGCAGGCCUUACAAAGCCGCGGCGGCCCAAUUGCCGGAGACCCUAGAG
KX056898    GGAGUUUGCCGCUGGGAAAAGAGGAGCGGCUUUUGGAGUGAUGGAAGCCCUGGGAACACUGCCAGGACACAUGACAGAGAGAUUCCAGGAAGCCAUUGACAACCUCGCUGUGCUCAUGCGGGCAGAGACUGGAAGCAGGCCUUACAAAGCCGCGGCAGCCCAAUUGCCGGAGACCCUAGAG
KX117076    GGAGUUUGCCGCUGGGAAAAGAGGAGCGGCUUUUGGAGUGAUGGAAGCCUUGGGAACACUGCCAGGACACAUGACAGAGAGAUUCCAGGAAGCCAUUGACAACCUCGCUGUGCUCAUGCGGGCAGAGACUGGAAGCAGGCCUUACAAAGCCGCGGCGGCCCAAUUGCCGGAGACCCUAGAG
KX185891    GGAGUUUGCCGCUGGGAAAAGAGGAGCGGCUUUUGGAGUGAUGGAAGCCUUGGGAACACUGCCAGGACACAUGACAGAGAGAUUCCAGGAAGCCAUUGACAACCUCGCUGUGCUCAUGCGGGCAGAGACUGGAAGCAGGCCUUACAAAGCCGCGGCGGCCCAAUUGCCGGAGACCCUAGAG
KX247646    GGAGUUUGCCGCUGGGAAAAGAGGAGCGGCUUUUGGAGUGAUGGAAGCCCUGGGAACACUGCCAGGACACAUGACAGAGAGAUUCCAGGAAGCCAUUGACAACCUCGCUGUGCUCAUGCGGGCAGAGACUGGAAGCAGGCCUUACAAAGCCGCGGCGGCCCAAUUGCCGGAGACCCUAGAG
KX262887    GGAGUUUGCCGCUGGGAAAAGAGGAGCGGCUUUUGGAGUGAUGGAAGCCCUGGGAACACUGCCAGGACACAUGACAGAGAGAUUCCAGGAAGCCAUUGACAACCUCGCUGUGCUCAUGCGGGCAGAGACUGGAAGCAGGCCUUACAAAGCCGCGGCGGCCCAAUUGCCGGAGACCCUAGAG
KX280026    GGAGUUUGCCGCUGGGAAAAGAGGAGCGGCUUUUGGAGUGAUGGAAGCCCUGGGAACACUGCCAGGACACAUGACAGAGAGAUUCCAGGAAGCCAUUGACAACCUCGCUGUGCUCAUGCGGGCAGAGACUGGAAGCAGGCCUUACAAAGCCGCGGCGGCCCAAUUGCCGGAGACCCUAGAG
KU758877    GGAGUUUGCCGCUGGGAAAAGAGGAGCGGCUUUUGGAGUGAUGGAAGCCCUGGGAACACUGCCAGGACACAUGACAGAGAGAUUCCAGGAAGCCAUUGACAACCUCGCUGUGCUCAUGCGGGCAGAGACUGGAAGCAGGCCUUACAAAGCCGCGGCGGCCCAAUUGCCGGAGACCCUAGAG
KU866423    GGAGUUUGCCGCUGGGAAAAGAGGAGCGGCUUUUGGAGUGAUGGAAGCCUUGGGAACACUGCCAGGACACAUGACAGAGAGAUUCCAGGAAGCCAUUGACAACCUCGCUGUGCUCAUGCGGGCAGAGACUGGAAGCAGGCCUUACAAAGCCGCGGCGGCCCAAUUGCCGGAGACCCUAGAG
KX377336    AGAAUUUGCCGCUGGGAAAAGAGGAGCGGCCUUUGGAGUGAUGGAAGCCCUGGGAACACUGCCAGGACACAUGACAGAGAGGUUUCAGGAAGCCAUUGACAACCUCGCUGUGCUCAUGCGGGCAGAGACUGGAAGCAGGCCCUACAAAGCCGCGGCGGCCCAAUUACCGGAGACCUUAGAG
KX377337    GGAGUUUGCCGCUGGGAAAAGAGGAGCGGCUUUUGGAGUGAUGGAAGCCCUGGGAACACUGCCAGGACACAUGACAGAGAGAUUCCAGGAAGCCAUUGACAACCUCGCUGUGCUCAUGCGGGCAGAGACUGGAAGCAGGCCUUACAAAGCCGCGGCGGCCCAAUUGCCGGAGACCCUAGAG
```

```
KX369547   GGAGUUUGCCGCUGGGAAAAGAGGAGCGGCUUUUGGAGUGAUGGAAGCCCUGGGAACACUGCCAGGACACAUGACAGAGAGAUUCCAGGAAGCCAUUGACAACCUCGCUGUGCUCAUGCGGGCAGAGACUGGAAGCAGGCCUUACAAAGCCGCGGCGGCCCAAUUGCCGGAGACCCUAGAG
KX548902   GGAGUUUGCCGCUGGGAAAAGAGGAGCGGCUUUUGGAGUGAUGGAAGCCCUGGGAACACUGCCAGGACACAUGACAGAGAGAUUCCAGGAAGCCAUUGACAACCUCGCUGUGCUCAUGCGGGCAGAGACUGGAAGCAGGCCUUACAAAGCCGCGGCGGCCCAAUUGCCGGAGACCCUAGAG
KX601166   AGAAUUUGCCGCUGGGAAGAGAGGAGCGGCUUUGGGAGUAAUGGAUGCCCUGGGAACAUUGCCAGGACACAUGACAGAGAGGUUUCAGGAAGCCAUUGACAAUCUCGCUGUGCUCAUGCGAGCAGAGACUGGAAGUAGGCCCUACAAAGCAGCGGCAGCUCAACUGCCGGAGACCUUAGAG
KX601167   AGAAUUUGCCGCUGGGAAAAGAGGAGCGGCCUUUGGAGUGAUGGAAGCCCUGGGAACACUGCCAGGACACAUGACAGAGAGGUUUCAGGAAGCCAUUGACAACCUCGCUGUGCUCAUGCGGGCAGAGACUGGAAGCAGGCCCUACAAAGCCGCGGCGGCCCAAUUACCGGAGACCUUAGAG
KX601168   GGAGUUUGCCGCUGGGAAAAGAGGAGCGGCUUUUGGAGUGAUGGAAGCCCUGGGAACACUGCCAGGACACAUGACAGAGAGAUUCCAGGAAGCCAUUGACAACCUCGCUGUGCUCAUGCGGGCAGAGACUGGAAGCAGGCCUUACAAAGCCGCGGCGGCCCAAUUGCCGGAGACCCUAGAG
KX766028   GGAGUUUGCCGCUGGGAAAAGAGGAGCGGCUUUUGGAGUGAUGGAAGCCCUGGGAACACUGCCAGGACACAUGACAGAGAGAUUCCAGGAAGCCAUUGACAACCUCGCUGUGCUCAUGCGGGCAGAGACUGGAAGCAGGCCUUACAAAGCCGCGGCAGCCCAAUUGCCGGAGACCCUAGAG
KX766029   GGAGUUUGCCGCUGGAAAAAGAGGAGCGGCUUUUGGGGUGAUGGAAGCCCUGGGAACACUGCCAGGACACAUGACAGAGAGAUUCCAGGAAGCCAUUGACAACCUCGCUGUGCUCAUGCGGGCAGAGACUGGAAGCAGGCCUUACAAAGCCGCGGCGGCCCAAUUGCCGGAGACCCUAGAG
KU940224   GGAGUUUGCCGCUGGGAAAAGAGGAGCGGCUCUUGGAGUGAUGGAAGCCCUGGGAACACUGCCAGGACACAUGACAGAGAGAUUCCAGGAAGCCAUUGACAACCUCGCUGUGCUCAUGCGGGCAGAGACUGGAAGCAGGCCUUACAAAGCCGCGGCGGCCCAAAUGCCGGAGACCCUAGAG

           6666666666666666666666666666666666666666666666666666666666666666666666666666666666666666666666666666666666666666666666666666666666666666666666666666666666666666666666666666666
           5555555555555555555555555555555555555555555555555555555555555555555555555555555555566666666666666666666666666666666666666666666666666666666666666666666666666666666666666666666666
           1112222222222333333333344444444445555555555666666666677777777778888888888999999999900000000001111111111222222222233333333334444444444555555555566666666667777777777888888888899999999
Sequence   7890123456789012345678901234567890123456789012345678901234567890123456789012345678901234567890123456789012345678901234567890123456789012345678901234567890123456789012345678901234567
-------------------------------------------------------------------------------------------------------------------------------------------------------------------------------------------------------
NC_012532  ACCAUUAUGCUCUUAGGUUUGCUGGGAACAGUUUCACUGGGGAUCUUCUUCGUCUUGAUGCGGAAUAAGGGCAUCGGGAAGAUGGGCUUUGGAAUGGUAACCCUUGGGGCCAGUGCAUGGCUCAUGUGGCUUUCGGAAAUUGAACCAGCCAGAAUUGCAUGUGUCCUCAUUGUUGUGUUUU
KU922960   ACCAUUAUGCUUUUGGGGUUGCUGGGAACAGUCUCGCUGGGAAUCUUUUUCGUCUUGAUGAGGAACAAGGGCAUAGGGAAGAUGGGCUUUGGAAUGGUGACUCUUGGGGCCAGCGCAUGGCUCAUGUGGCUCUCGGAAAUUGAGCCAGCCAGAAUUGCAUGUGUCCUCAUUGUUGUGUUCC
KU922923   ACCAUUAUGCUUUUGGGGUUGCUGGGAACAGUCUCGCUGGGAAUCUUUUUCGUCUUGAUGAGGAACAAGGGCAUAGGGAAGAUGGGCUUUGGAAUGGUGACUCUUGGGGCCAGCGCAUGGCUCAUGUGGCUCUCGGAAAUUGAGCCAGCCAGAAUUGCAUGUGUCCUCAUUGUUGUGUUCC
KU820898   ACCAUUAUGCUUUUGGGGUUGCUGGGAACAGUCUCGCUGGGAAUCUUCUUCGUCUUGAUGAGGAACAAGGGCAUAGGGAAGAUGGGCUUUGGAAUGGUGACUCUUGGGGCCAGCGCAUGGCUCAUGUGGCUCUCGGAAAUUGAGCCAGCCAGAAUUGCAUGUGUCCUCAUUGUUGUGUUCC
KU321639   ACCAUUAUGCUUUUGGGGUUGCUGGGAACAGUCUCGCUGGGAAUCUUUUUCGUCUUGAUGAGGAACAAGGGCAUAGGGAAGAUGGGCUUUGGAAUGGUGACUCUUGGGGCCAGCGCAUGGCUCAUGUGGCUCUCGGAAAUUGAGCCAGCCAGAAUUGCAUGUGUCCUCAUUGUUGUGUUCC
KU820899   ACCAUUAUGCUUUUGGGGUUGCUGGGAACAGUCUCGCUGGGAAUCUUUUUCGUCUUGAUGAGGAACAAGGGCAUAGGGAAGAUGGGCUUUGGAAUGGUGACUCUUGGGGCCAGCGCAUGGCUCAUGUGGCUCUCGGAAAUUGAGCCAGCCAGAAUUGCAUGUGUCCUCAUUGUUGUGUUCC
KU853012   ACCAUUAUGCUUUUGGGGUUGCUGGGAACAGUCUCGCUGGGAAUCUUUUUCGUCUUGAUGAGGAACAAGGGCAUAGGGAAGAUGGGCUUUGGAAUGGUGACUCUUGGGGCCAGCGCAUGGCUCAUGUGGCUCUCGGAAAUUGAGCCAGCCAGAAUUGCAUGUGUCCUCAUUGUUGUGUUCC
KU729217   ACCAUUAUGCUUUUGGGGUUGCUGGGAACAGUCUCGCUGGGAAUCUUUUUCGUCUUGAUGAGGAACAAGGGCAUAGGGAAGAUGGGCUUUGGAAUGGUGACUCUUGGGGCCAGCGCAUGGCUCAUGUGGCUCUCGGAAAUUGAGCCAGCCAGAAUUGCAUGUGUCCUCAUUGUUGUGUUCC
KU729218   ACCAUUAUGCUUUUGGGGUUGCUGGGAACAGUCUCGCUGGGAAUCUUUUUCGUCUUGAUGAGGAACAAGGGCAUAGGGAAGAUGGGCUUUGGAAUGGUGACUCUUGGGGCCAGCGCAUGGCUCAUGUGGCUCUCGGAAAUUGAGCCAGCCAGAAUUGCAUGUGUCCUCAUUGUUGUGUUCC
KU761564   ACCAUUAUGCUUUUGGGGUUGCUGGGAACAGUCUCGCUGGGAAUCUUCUUCGUCUUGAUGAGGAACAAGGGCAUAGGGAAGAUGGGCUUUGGAAUGGUGACUCUUGGGGCCAGCGCAUGGCUCAUGUGGCUCUCGGAAAUUGAGCCAGCCAGAAUUGCAUGUGUCCUCAUUGUUGUGUUCC
KU740184   ACCAUUAUGCUUUUGGGGUUGCUGGGAACAGUCUCGCUGGGAAUCUUCUUCGUCUUGAUGAGGAACAAGGGCAUAGGGAAGAUGGGCUUUGGAAUGGUGACUCUUGGGGCCAGCGCAUGGCUCAUGUGGCUCUCGGAAAUUGAGCCAGCCAGAAUUGCAUGUGUCCUCAUUGUUGUGUUCC
KU744693   ACCAUUAUGCUUUUGGGGUUGCUGGGAACAGUCUCGCUGGGAAUCUUUUUCGUCUUGAUGAGGAACAAGGGCAUAGGGAAGAUGGGCUUUGGAAUGGUGACUCUUGGGGCCAGCGCAUGGCUCAUGUGGCUCUCGGAAAUUGAGCCAGCCAGAAUUGCAUGUGUCCUCAUUGUUGUGUUCC
KU497555   ACCAUUAUGCUUUUGGGGUUGCUGGGAACAGUCUCGCUGGGAAUCUUUUUCGUCUUGAUGAGGAACAAGGGCAUAGGGAAGAUGGGCUUUGGAAUGGUGACUCUUGGGGCCAGCGCAUGGCUCAUGUGGCUCUCGGAAAUUGAGCCAGCCAGAAUUGCAUGUGUCCUCAUUGUUGUGUUCC
KU527068   ACCAUUAUGCUUUUGGGGUUGCUGGGAACAGUCUCGCUGGGAAUCUUUUUCGUCUUGAUGAGGAACAAGGGCAUAGGGAAGAUGGGCUUUGGAAUGGUGACUCUUGGGGCCAGCGCAUGGCUCAUGUGGCUCUCGGAAAUUGAGCCAGCCAGAAUUGCAUGUGUCCUCAUUGUUGUGUUCC
KU501217   ACCAUUAUGCUUUUGGGGUUGCUGGGAACAGUCUCGCUGGGAAUCUUUUUCGUCUUGAUGAGGAACAAGGGCAUAGGGAAGAUGGGCUUUGGAAUGGUGACCCUUGGGGCCAGUGCAUGGCUCAUGUGGCUCUCGGAAAUUGAGCCAGCCAGAAUUGCAUGUGUCCUCAUUGUUGUGUUCC
KU501216   ACCAUUAUGCUUUUGGGGUUGCUGGGAACAGUCUCGCUGGGAAUCUUUUUCGUCUUGAUGAGGAACAAGGGCAUAGGGAAGAUGGGCUUUGGAAUGGUGACCCUUGGGGCCAGUGCAUGGCUCAUGUGGCUCUCGGAAAUUGAGCCAGCCAGAAUUGCAUGUGUCCUCAUUGUUGUGUUCC
KU501215   ACCAUAAUGCUUUUGGGGUUGCUGGGAACAGUCUCGCUGGGAAUCUUCUUCGUCUUGAUGAGGAACAAGGGCAUAGGGAAGAUGGGCUUUGGAAUGGUGACUCUUGGGGCCAGCGCAUGGCUCAUGUGGCUCUCGGAAAUUGAGCCAGCCAGAAUUGCAUGUGUCCUCAUUGUUGUGUUCC
KU365780   ACCAUUAUGCUUUUGGGGUUGCUGGGAACAGUCUCGCUGGGAAUCUUCUUCGUCUUGAUGAGGAACAAGGGCAUAGGGAAGAUGGGCUUUGGAAUGGUGACUCUUGGGGCCAGCGCAUGGCUCAUGUGGCUCUCGGAAAUUGAGCCAGCCAGAAUUGCAUGUGUCCUCAUUGUUGUGUUUC
KU365779   ACCAUUAUGCUUUUGGGGUUGCUGGGAACAGUCUCGCUGGGAAUCUUCUUCGUCUUGAUGAGGAACAAGGGCAUAGGGAAGAUGGGCUUUGGAAUGGUGACUCUUGGGGCCAGCGCAUGGCUCAUGUGGCUCUCGGAAAUUGAGCCAGCCAGAAUUGCAUGUGUCCUCAUUGUUGUGUUUC
KU707826   ACCAUUAUGCUUUUGGGGUUGCUGGGAACAGUCUCGCUGGGAAUCUUCUUCGUCUUGAUGAGGAACAAGGGCAUAGGGAAGAUGGGCUUUGGAAUGGUGACUCUUGGGGCCAGCGCAUGGCUCAUGUGGCUCUCGGAAAUUGAGCCAGCCAGAAUUGCAUGUGUCCUCAUUGUUGUGUUUC
KU365778   ACCAUAAUGCUUUUGGGGUUGCUGGGAACAGUCUCGCUGGGAAUCUUCUUCGUCUUGAUGAGGAACAAGGGCAUAGGGAAGAUGGGCUUUGGAAUGGUGACUCUUGGGGCCAGCGCAUGGCUCAUGUGGCUCUCGGAAAUUGAGCCAGCCAGAAUUGCAUGUGUCCUCAUUGUUGUGUUCC
KU365777   ACCAUUAUGCUUUUGGGGUUGCUGGGAACAGUCUCGCUGGGAAUCUUCUUCGUCUUGAUGAGGAACAAGGGCAUAGGGAAGAUGGGCUUUGGAAUGGUGACUCUUGGGGCCAGCGCAUGGCUCAUGUGGCUCUCGGAAAUUGAGCCAGCCAGAAUUGCAUGUGUCCUCAUUGUUGUGUUUC
KU312312   ACCAUUAUGCUUUUGGGGUUGCUGGGAACAGUCUCGCUGGGAAUCUUCUUCGUCUUGAUGAGGAACAAGGGCAUAGGGAAGAUGGGCUUUGGAAUGGUGACUCUUGGGGCCAGCGCAUGGCUCAUGUGGCUCUCGGAAAUUGAGCCAGCCAGAAUUGCAUGUGUCCUCAUUGUUGUGUUCC
KF268950   ACCAUCAUGCUCUUAGGCUUGUUGGGAACAGUUUCGUUGGGGAUCUUCUUUGUCUUGAUGCGGAACAAGGGCAUCGGGAAGAUGGGUUUUGGAAUGGUAACUCUUGGGGCCAGUGCAUGGCUCAUGUGGCUUUCGGAAAUUGAACCAGCCAGAAUUGCAUGUGUCCUCAUUGUUGUGUUCU
KF268949   ACCAUCAUGCUCCUAGGUUUGCUGGGAACAGUUUCGUUGGGGAUCUUCUUUGUCUUGAUGCGGAACAAGGGCAUCGGGAAGAUGGGUUUUGGAAUGGUAACUCUUGGGGCCAGUGCAUGGCUCAUGUGGCUUUCGGAAAUUGAGCCAGCCAGAAUUGCAUGUGUCCUCAUUGUUGUGUUCU
KF268948   ACCAUCAUGCUCUUAGGCUUGUUGGGAACAGUUUCGUUGGGGAUCUUCUUUGUCUUGAUGCGGAACAAGGGCAUCGGGAAGAUGGGUUUUGGAAUGGUAACUCUUGGGGCCAGUGCAUGGCUCAUGUGGCUUUCGGAAAUUGAACCAGCCAGAAUUGCAUGUGUCCUCAUUGUUGUGUUCU
KJ776791   ACCAUUAUGCUUUUGGGGUUGCUGGGAACAGUCUCGCUGGGAAUCUUUUUCGUCUUGAUGAGGAACAAGGGCAUAGGGAAGAUGGGCUUUGGAAUGGUGACUCUUGGGGCCAGCGCAUGGCUCAUGUGGCUCUCGGAAAUUGAGCCAGCCAGAAUUGCAUGUGUCCUCAUUGUUGUGUUCC
KF383119   ACCAUUAUGCUCUUAGGUUUGCUGGGAACAGUUUCACUGGGGAUCUUCUUUGUCUUGAUGCGGAAUAAGGGCAUCGGGAAGAUGGGCUUUGGAAUGGUAACCCUUGGGGCCAGUGCAUGGCUCAUGUGGCUUUCGGAAAUUGAACCAGCCAGAAUUGCAUGUGUCCUCAUUGUUGUGUUUU
KF383118   ACCAUUAUGCUCUUAGGUUUGCUGGGAACAGUUUCACUGGGGAUCUUCUUCGUCUUGAUGCGGAAUAAGGGCAUCGGGAAGAUGGGCUUUGGAAUGGUAACCCUUGGGGCCAGUGCAUGGCUCAUGUGGCUUUCGGAAAUUGAACCAGCCAGAAUUGCAUGUGUCCUCAUUGUUGUGUUUU
KF383117   ACCAUCAUGCUUUUGGGCUUAUUGGGAACAGUUUCGCUAGGAAUCUUCUUUGUCUUGAUGCGGAACAAGGGCAUCGGGAAGAUGGGCUUCGGAAUGGUAACCCUUGGGGCCAGCGCAUGGCUCAUGUGGCUCUCGGAAAUUGAACCAGCCAGAAUCGCAUGUGUCCUCAUUGUCGUGUUUU
KF383116   ACCAUCAUGCUUUUGGGCUUAUUGGGAACAGUUUCGCUAGGAAUCUUCUUUGUCUUGAUGCGGAACAAGGGCAUCGGGAAGAUGGGCUUCGGAAUGGUAACCCUUGGGGCCAGCGCAUGGCUCAUGUGGCUUUCGGAAAUUGAACCAGCCAGAAUCGCAUGUGUCCUCAUUGUCGUGUUUU
KF383115   ACCAUCAUGCUCUUAGGUUUGCUGGGAACAGUUUCGUUGGGGAUAUUCUUUGUCUUGAUGCGGAACAAGGGCAUCGGGAAGAUGGGGUUUGGAAUGGUAACUCUUGGGGCCAGUGCAUGGCUCAUGUGGCUUUCGGAAAUUGAACCAGCCAGAAUUGCAUGUGUCCUCAUUGUUGUGUUUU
EU545988   ACCAUCAUGCUUUUGGGGUUGCUGGGAACAGUCUCGCUGGGAAUCUUUUUCGUCUUGAUGCGGAAUAAGGGCAUCGGGAAGAUGGGCUUUGGAAUGGUGACUCUUGGGGCCAGCGCAUGGCUUAUGUGGCUCUCGGAAAUUGAGCCAGCCAGAAUUGCAUGUGUCCUCAUUGUUGUGUUCC
KU955589   ACCAUUAUGCUUUUGGGGUUGCUGGGAACAGUCUCGCUGGGAAUCUUUUUCGUCUUGAUGAGGAACAAGGGCAUAGGGAAGAUGGGCUUUGGAAUGGUGACUCUUGGGGCCAGCGCAUGGCUCAUGUGGCUCUCGGAAAUUGAGCCAGCCAGAAUUGCAUGUGUCCUCAUUGUUGUGUUCC
KU955590   ACCAUUAUGCUUUUGGGGUUGCUGGGAACAGUCUCGCUGGGAAUCUUCUUCGUCUUGAUGAGGAACAAGGGCAUAGGGAAGAUGGGCUUUGGAAUGGUGACUCUUGGGGCCAGCGCAUGGCUCAUGUGGCUCUCGGAAAUUGAGCCAGCCAGAAUUGCAUGUGUCCUCAUUGUUGUGUUCC
KU940228   ACCAUUAUGCUUUUGGGGUUGCUAGGAACAGUCUCGCUGGGAAUCUUUUUCGUCUUGAUGAGGAACAAGGGCAUAGGGAAGAUGGGCUUUGGAAUGGUGACUCUUGGGGCCAGCGCAUGGCUCAUGUGGCUCUCGGAAAUUGAGCCAGCCAGAAUUGCAUGUGUCCUCAUUGUUGUGUUCC
KU991811   ACCAUUAUGCUUUUGGGGUUGCUGGGAACAGUCUCGCUGGGAAUCUUUUUCGUCUUGAUGAGGAACAAGGGCAUAGGGAAGAUGGGCUUUGGAAUGGUGACUCUUGGGGCCAGCGCAUGGCUCAUGUGGCUCUCGGAAAUUGAGCCAGCCAGAAUUGCAUGUGUCCUCAUUGUUGUGUUCC
KU509998   ACCAUUAUGCUUUUGGGGUUGCUGGGAACAGUCUCGCUGGGAAUCUUUUUCGUCUUGAUGAGGAACAAGGGCAUAGGGAAGAUGGGCUUUGGAAUGGUGACUCUUGGGGCCAGCGCAUGGCUCAUGUGGCUCUCGGAAAUUGAGCCAGCCAGAAUUGCAUGUGUCCUCAUUGUUGUGUUCC
KX056898   ACCAUUAUGCUUUUGGGGUUGCUGGGAACAGUCUCGCUGGGAAUCUUCUUCGUCUUGAUGAGGAACAAGGGCAUAGGGAAGAUGGGCUUUGGAAUGGUGACUCUUGGGGCCAGCGCAUGGCUCAUGUGGCUCUCGGAAAUUGAGCCAGCCAGAAUUGCAUGUGUCCUCAUUGUUGUGUUCC
KX117076   ACCAUUAUGCUUUUGGGGUUGCUGGGAACAGUCUCGCUGGGAAUCUUUUUCGUCUUGAUGAGGAACAAGGGCAUAGGGAAGAUGGGCUUUGGAAUGGUGACUCUUGGGGCCAGCGCAUGGCUCAUGUGGCUCUCGGAAAUUGAGCCAGCCAGAAUUGCAUGUGUCCUCAUUGUUGUGUUCC
KX185891   ACCAUUAUGCUUUUGGGGUUGCUGGGAACAGUCUCGCUGGGAAUCUUUUUCGUCUUGAUGAGGAACAAGGGCAUAGGGAAGAUGGGCUUUGGAAUGGUGACUCUUGGGGCCAGCGCAUGGCUCAUGUGGCUCUCGGAAAUUGAGCCAGCCAGAAUUGCAUGUGUCCUCAUUGUUGUGUUCC
KX247646   ACCAUUAUGCUUUUGGGGUUGCUGGGAACAGUCUCGUUGGGAAUCUUUUUCGUCUUGAUGAGGAACAAGGGCAUAGGGAAGAUGGGCUUUGGAAUGGUGACUCUUGGGGCCAGCGCAUGGCUCAUGUGGCUCUCGGAAAUUGAGCCAGCCAGAAUUGCAUGUGUCCUCAUUGUUGUGUUCC
KX262887   ACCAUUAUGCUUUUGGGGUUGCUGGGAACAGUCUCGCUGGGAAUCUUUUUCGUCUUGAUGAGGAACAAGGGCAUAGGGAAGAUGGGCUUUGGAAUGGUGACCCUUGGGGCCAGUGCAUGGCUCAUGUGGCUCUCGGAAAUUGAGCCAGCCAGAAUUGCAUGCGUCCUCAUUGUUGUGUUCC
KX280026   ACCAUUAUGCUUUUGGGGUUGCUGGGAACAGUCUCGCUGGGAAUCUUUUUCGUCUUGAUGAGGAACAAGGGCAUAGGGAAGAUGGGCUUUGGAAUGGUGACUCUUGGGGCCAGCGCAUGGCUCAUGUGGCUCUCGGAAAUUGAGCCAGCCAGAAUUGCAUGUGUCCUCAUUGUUGUGUUCC
KU758877   ACCAUAAUGCUUUUGGGGUUGCUGGGAACAGUCUCGCUGGGAAUCUUCUUCGUCUUGAUGAGGAACAAGGGCAUAGGGAAGAUGGGCUUUGGAAUGGUGACCCUUGGGGCCAGCGCAUGGCUCAUGUGGCUCUCGGAAAUUGAGCCAGCCAGGAUUGCAUGUGUCCUCAUUGUUGUGUUCC
KU866423   ACCAUUAUGCUUUUGGGGUUGCUGGGAACAGUCUCGCUGGGAAUCUUUUUCGUCUUGAUGAGGAACAAGGGCAUAGGGAAGAUGGGCUUUGGAAUGGUGACUCUUGGGGCCAGCGCAUGGCUCAUGUGGCUCUCGGAAAUUGAGCCAGCCAGAAUUGCAUGUGUCCUCAUUGUUGUGUUCC
KX377336   ACCAUCAUGCUUUUGGGUUUGCUGGGAACAGUCUCGCUGGGAAUCUUCUUUGUCUUGAUGCGGAACAAGGGCAUAGGGAAGAUGGGCUUUGGAAUGGUGACCCUUGGGGCCAGUGCAUGGCUUAUGUGGCUCUCGGAAAUUGAGCCAGCCAGAAUUGCAUGUGUCCUCAUUGUCGUGUUUC
KX377337   ACCAUAAUGCUUUUGGGGUUGCUGGGAACAGUCUCGCUGGGAAUCUUCUUCGUCUUGAUGAGGAACAAGGGCAUAGGGAAGAUGGGCUUUGGAAUGGUGACUCUUGGGGCCAGCGCAUGGCUCAUGUGGCUCUCGGAAAUUGAGCCAGCCAGAAUUGCAUGUGUCCUCAUUGUUGUGUUCC
KX369547   ACCAUUAUGCUUUUGGGGUUGCUGGGAACAGUCUCGCUGGGAAUCUUUUUCGUCUUGAUGAGGAACAAGGGCAUAGGGAAGAUGGGCUUUGGAAUGGUGACUCUUGGGGCCAGCGCAUGGCUCAUGUGGCUCUCGGAAAUUGAGCCAGCCAGAAUUGCAUGUGUCCUCAUUGUUGUGUUCC
KX548902   ACCAUUAUGCUUUUGGGGUUGCUGGGAACAGUCUCGCUGGGAAUCUUUUUCGUCUUGAUGAGGAACAAGGGCAUAGGGAAGAUGGGCUUUGGAAUGGUGACUCUUGGGGCCAGCGCAUGGCUCAUGUGGCUCUCGGAAAUUGAGCCAGCCAGAAUUGCAUGUGUCCUCAUUGUUGUGUUCC
KX601166   ACUAUCAUGCUUCUGGGCUUAUUGGGAACAGUUUCGCUAGGAAUCUUCUUUGUCUUGAUGCGGAACAAGGGCAUCGGGAAGAUGGGCUUCGGAAUGGUAACCCUUGGGGCCAGCGCAUGGCUCAUGUGGCUUUCGGAAAUUGAACCAGCCAGAAUCGCAUGUGUCCUCAUUGUCGUGUUUU
KX601167   ACCAUCAUGCUUUUGGGUUUGCUGGGAACAGUCUCGCUGGGAAUCUUCUUUGUCUUGAUGCGGAACAAGGGCAUAGGGAAGAUGGGCUUUGGAAUGGUGACCCUUGGGGCCAGUGCAUGGCUUAUGUGGCUCUCGGAAAUUGAGCCAGCCAGAAUUGCAUGUGUCCUCAUUGUCGUGUUUC
KX601168   ACCAUAAUGCUUUUGGGGUUGCUGGGAACAGUCUCGCUGGGAAUCUUCUUCGUCUUGAUGAGGAACAAGGGCAUAGGGAAGAUGGGCUUUGGAAUGGUGACUCUUGGGGCCAGCGCAUGGCUCAUGUGGCUCUCGGAAAUUGAGCCAGCCAGAAUUGCAUGUGUCCUCAUUGUUGUGUUCC
KX766028   ACCAUUAUGCUUUUGGGGUUGCUGGGAACAGUCUCGCUGGGAAUCUUCUUCGUCUUGAUGAGGAACAAGGGCAUAGGGAAGAUGGGCUUUGGAAUGGUGACUCUUGGGGCCAGCGCAUGGCUCAUGUGGCUCUCGGAAAUUGAGCCAGCCAGAAUUGCAUGUGUCCUCAUUGUUGUGUUCC
KX766029   ACCAUUAUGCUUUUGGGGUUGCUGGGAACAGUCUCGCUGGGAAUCUUUUUCGUCUUGAUGAGGAACAAGGGCAUAGGGAAGAUGGGCUUUGGAAUGGUGACCCUUGGGGCCAGUGCAUGGCUCAUGUGGCUCUCGGAAAUUGAGCCAGCCAGAAUUGCAUGUGUCCUCAUUGUUGUGUUCC
KU940224   ACCAUUAUGCUUUUGGGGUUGCUAGGAACAGUCUCGCUGGGAAUCUUUUUCGUCUUGAUGAGGAACAAGGGCAUAGGGAAGAUGGGCUUUGGAAUGGUGACUCUUGGGGCCAGCGCAUGGCUCAUGUGGCUCUCGGAAAUUGAGCCAGCCAGAAUUGCAUGUGUCCUCAUUGUUGUGUUCC

           6666666666666666666666666666666666666666666666666666666666666666666666666666666666666666666666666666666666666666666666666666666666666666666666666666666666666666666666666666666
           6677777777777777777777777777777777777777777777777777777777777777777777777777777777777777777777777777788888888888888888888888888888888888888888888888888888888888888888888888888888
           9900000000001111111111222222222233333333334444444444555555555566666666667777777777888888888899999999990000000000111111111122222222223333333333444444444455555555556666666666777777777
Sequence   8901234567890123456789012345678901234567890123456789012345678901234567890123456789012345678901234567890123456789012345678901234567890123456789012345678901234567890123456789012345678
```

```
---------------------------------------------------------------------------------------------------------------------------------------------------------------------------------------------------
NC_012532    UAUUACUGGUGGUGCUCAUACCCGAGCCAGAGAAGCAAAGAUCUCCCCAAGAUAACCAGAUGGCAAUUAUCAUCAUGGUGGCAGUGGGCCUUCUAGGUUUGAUAACUGCAAACGAACUUGGAUGGCUGGAAAGAACAAAAAAUGACAUAGCUCAUCUAAUGGGAAGGAGAGAAGAAGGAGC
KU922960     UAUUGCUGGUGGUGCUCAUACCUGAGCCAGAAAAGCAAAGAUCUCCCCAGGACAACCAAAUGGCAAUCAUCAUCAUGGUAGCAGUAGGUCUUCUGGGCUUGAUUACCGCCAAUGAACUCGGAUGGUUGGAGAGAACAAAGAGUGACCUAAGCCAUCUAAUGGGAAGGAGAGAGGAGGGGGC
KU922923     UAUUGCUGGUGGUGCUCAUACCUGAGCCAGAAAAGCAAAGAUCUCCCCAGGACAACCAAAUGGCAAUCAUCAUCAUGGUAGCAGUAGGUCUUCUGGGCUUGAUUACCGCCAAUGAACUCGGAUGGUUGGAGAGAACAAAGAGUGACCUAAGCCAUCUAAUGGGAAGGAGAGAGGAGGGGGC
KU820898     UAUUGCUGGUGGUGCUCAUACCUGAGCCAGAAAAGCAAAGAUCUCCCCAGGACAACCAAAUGGCAAUCAUCAUCAUGGUAGCAGUAGGUCUUCUGGGCUUGAUUACCGCCAAUGAACUCGGAUGGUUGGAGAGAACAAAGAGUGACCUAAGCCAUCUAAUGGGAAGGAGAGAGGAGGGGGC
KU321639     UAUUGCUGGUGGUGCUCAUACCUGAGCCAGAAAAGCAAAGAUCUCCCCAGGACAACCAAAUGGCAAUCAUCAUCAUGGUAGCAGUAGGUCUUCUGGGCUUGAUUACCGCCAAUGAACUCGGAUGGUUGGAGAGAACAAAGAGUGACCUAAGCCAUCUAAUGGGAAGGAGAGAGGAGGGGGC
KU820899     UAUUGCUGGUGGUGCUCAUACCUGAGCCAGAAAAGCAAAGAUCUCCCCAGGACAACCAAAUGGCAAUCAUCAUCAUGGUAGCAGUAGGUCUUCUGGGCUUGAUUACCGCCAAUGAACUCGGAUGGUUGGAGAGAACAAAGAGUGACCUAAGCCAUCUAAUGGGAAGGAGAGAGGAGGGGGC
KU853012     UACUGCUGGUGGUGCUCAUACCUGAGCCAGAAAAGCAAAGAUCUCCCCAGGACAACCAAAUGGCAAUCAUCAUCAUGGUAGCAGUAGGUCUUCUGGGCUUGAUCACCGCCAAUGAACUCGGAUGGUUGGAGAGAACAAAGAGUGACCUAAGCCAUCUAAUGGGAAGGAGAGAGGAGGGAGC
KU729217     UAUUGCUGGUGGUGCUCAUACCUGAGCCAGAAAAGCAAAGAUCUCCCCAGGACAACCAAAUGGCAAUCAUCAUCAUGGUAGCAGUAGGUCUUCUGGGCUUGAUUACCGCCAAUGAACUCGGAUGGUUGGAGAGAACAAAGAGUGACCUAAGCCAUCUAAUGGGAAGGAGAGAGGAGGGAGC
KU729218     UAUUGCUGGUGGUGCUCAUACCUGAGCCAGAAAAGCAAAGAUCUCCCCAGGACAACCAAAUGGCAAUCAUCAUCAUGGUAGCAGUAGGUCUUCUGGGCUUGAUUACCGCCAAUGAACUCGGAUGGUUGGAGAGAACAAAGAGUGACCUAAGCCAUCUAAUGGGAAGGAGAGAGGAGGGGGC
KU761564     UAUUGCUGGUGGUGCUCAUACCUGAGCCAGAAAAGCAAAGAUCUCCCCAGGACAACCAAAUGGCAAUCAUCAUCAUGGUAGCAGUAGGUCUUCUGGGCUUGAUUACCGCCAAUGAACUCGGAUGGUUGGAGAGAACAAAGAGUGACCUAAGCCAUCUAAUGGGAAGGAGAGAGGAGGGGGC
KU740184     UAUUGCUGGUGGUGCUCAUACCUGAGCCAGAAAAGCAAAGAUCUCCCCAGGACAACCAAAUGGCAAUCAUCAUCAUGGUAGCAGUAGGUCUUCUGGGCUUGAUUACCGCCAAUGAACUCGGAUGGUUGGAGAGAACAAAGAGUGACCUAAGCCAUCUAAUGGGAAGGAGAGAGGAGGGGGC
KU744693     UAUUGCUGGUGGUGCUCAUACCUGAGCCAGAAAAGCAAAGAUCUCCCCAGGACAACCAAAUGGCCAUCAUCAUCAUGGUAGCAGUAGGUCUUCUGGGCUUGAUUACCGCCAAUGAACUCGGAUGGUUGGAGAGAACAAAGAGUGACCUAAGCCAUCUAAUGGGAAGGAGAGAGGAGGGGGC
KU497555     UAUUGCUGGUGGUGCUCAUACCUGAGCCAGAAAAGCAAAGAUCUCCCCAGGACAACCAAAUGGCAAUCAUCAUCAUGGUAGCAGUAGGUCUUCUGGGCUUGAUUACCGCCAAUGAACUCGGAUGGUUGGAGAGAACAAAGAGUGACCUAAGCCAUCUAAUGGGAAGGAGAGAGGAGGGGGC
KU527068     UAUUGCUGGUGGUGCUCAUACCUGAGCCAGAAAAGCAAAGAUCUCCCCAGGACAACCAAAUGGCAAUCAUCAUCAUGGUAGCAGUAGGUCUUCUGGGCUUGAUUACCGCCAAUGAACUCGGAUGGUUGGAGAGAACAAAGAGUGACCUAAGCCAUCUAAUGGGAAGGAGAGAGGAGGGAGC
KU501217     UAUUGCUGGUGGUGCUCAUACCUGAGCCAGAAAAGCAAAGAUCUCCCCAGGACAACCAAAUGGCAAUCAUCAUCAUGGUAGCAGUAGGUCUUCUGGGCUUGAUUACCGCCAAUGAACUCGGAUGGUUGGAGAGAACAAAGAGUGACCUAAGCCAUCUAAUGGGAAGGAGAGAGGAGGGAGC
KU501216     UAUUGCUGGUGGUGCUCAUACCUGAGCCAGAAAAGCAAAGAUCUCCCCAGGACAACCAAAUGGCAAUCAUCAUCAUGGUAGCAGUAGGUCUUCUGGGCUUGAUUACCGCCAAUGAACUCGGAUGGUUGGAGAGAACAAAGAGUGACCUAAGCCAUCUAAUGGGAAGGAGAGAGGAGGGAGC
KU501215     UAUUGCUGGUGGUGCUCAUACCUGAGCCAGAAAAGCAAAGAUCUCCCCAGGACAACCAAAUGGCAAUCAUCAUCAUGGUAGCAGUAGGUCUUCUGGGCUUGAUUACCGCCAAUGAACUCGGAUGGUUGGAGAGAACAAAGAGUGACCUAAGCCAUCUAAUGGGAAGGAGAGAGGAGGGGGC
KU365780     UAUUGCUGGUGGUGCUCAUACCUGAGCCAGAAAAGCAAAGAUCUCCCCAGGACAACCAAAUGGCAAUCAUCAUCAUGGUAGCAGUAGGUCUUCUGGGCUUGAUUACCGCCAAUGAACUCGGAUGGUUGGAGAGAACAAAGAGUGACCUAAGCCAUCUAAUGGGAAGGAGAGAGGAGGGGGC
KU365779     UAUUGCUGGUGGUGCUCAUACCUGAGCCAGAAAAGCAAAGAUCUCCCCAGGACAACCAAAUGGCAAUCAUCAUCAUGGUAGCAGUAGGUCUUCUGGGCUUGAUUACCGCCAAUGAACUCGGAUGGUUGGAGAGAACAAAGAGUGACCUAAGCCAUCUAAUGGGAAGGAGAGAGGAGGGGGC
KU707826     UAUUGCUGGUGGUGCUCAUACCUGAGCCAGAAAAGCAAAGAUCUCCCCAGGACAACCAAAUGGCAAUCAUCAUCAUGGUAGCAGUAGGUCUUCUGGGCUUGAUUACCGCCAAUGAACUCGGAUGGUUGGAGAGAACAAAGAGUGACCUAAGCCAUCUAAUGGGAAGGAGAGAGGAGGGGGC
KU365778     UAUUGCUGGUGGUGCUCAUACCUGAGCCAGAAAAGCAAAGAUCUCCCCAGGACAACCAAAUGGCAAUCAUCAUCAUGGUAGCAGUAGGUCUUCUGGGCUUGAUUACCGCCAAUGAACUCGGAUGGUUGGAGAGAACAAAGAGUGACCUAAGCCAUCUAAUGGGAAGGAGAGAGGAGGGGGC
KU365777     UAUUGCUGGUGGUGCUCAUACCUGAGCCAGAAAAGCAAAGAUCUCCCCAGGACAACCAAAUGGCAAUCAUCAUCAUGGUAGCAGUAGGUCUUCUGGGCUUGAUUACCGCCAAUGAACUCGGAUGGUUGGAGAGAACAAAGAGUGACCUAAGCCAUCUAAUGGGAAGGAGAGAGGAGGGGGC
KU312312     UAUUGCUGGUGGUGCUCAUACCUGAGCCAGAAAAGCAAAGAUCUCCCCAGGACAACCAAAUGGCAAUCAUCAUCAUGGUAGCAGUAGGUCUUCUGGGCUUGAUUACCGCCAAUGAACUCGGAUGGUUGGAGAGAACAAAGAGUGACCUAAGCCAUCUAAUGGGAAGGAGAGAGGAGGGGGC
KF268950     UGUUACUGGUGGUACUCAUACCCGAGCCAGAGAAGCAAAGAUCUCCCCAGGACAACCAGAUGGCAAUCAUCAUCAUGGUGGCAGUGGGCCUUCUGGGUUUGAUAACUGCAAACGAACUUGGAUGGCUGGAAAGAACAAAAAAUGACAUAGCUCAUCUAAUGGGAAGGAGAGAAGAAGGAGU
KF268949     UGUUACUGGUGGUACUCAUACCCGAGCCAGAGAAGCAAAGAUCUCCCCAGGACAACCAGAUGGCAAUCAUCAUCAUGGUGGCAGUGGGCCUUCUGGGUUUGAUAACUGCAAACGAACUUGGAUGGCUGGAAAGAACAAAAAAUGACAUAGCUCAUCUAAUGGGAAGGAGAGAAGAAGGAGU
KF268948     UGUUACUGGUGGUACUCAUACCCGAGCCAGAGAAGCAAAGAUCUCCCCAGGACAACCAGAUGGCAAUCAUCAUCAUGGUGGCAGUGGGCCUUCUGGGUUUGAUAACUGCAAACGAACUUGGAUGGCUGGAAAGAACAAAAAAUGACAUAGCUCAUCUAAUGGGAAGGAGAGAAGAAGGAGU
KJ776791     UAUUGCUGGUGGUGCUCAUACCUGAGCCAGAAAAGCAAAGAUCUCCCCAGGACAACCAAAUGGCAAUCAUCAUCAUGGUAGCAGUAGGUCUUCUGGGCUUGAUUACCGCCAAUGAACUCGGAUGGUUGGAGAGAACAAAGAGUGACCUAAGCCAUCUAAUGGGAAGGAGAGAGGAGGGGGC
KF383119     UAUUACUGGUGGUGCUCAUACCCGAGCCAGAGAAGCAAAGAUCUCCCCAAGAUAACCAGAUGGCAAUUAUCAUCAUGGUGGCAGUGGGCCUUCUAGGUUUGAUAACUGCAAACGAACUUGGAUGGCUGGAAAGAACAAAAAAUGACAUAGCUCAUCUAAUGGGAAGGAGAGAAGAAGGAGC
KF383118     UAUUACUGGUGGUGCUCAUACCCGAGCCAGAGAAGCAAAGAUCUCCCCAAGAUAACCAGAUGGCAAUUAUCAUCAUGGUGGCAGUGGGCCUUCUAGGUUUGAUAACUGCAAACGAACUUGGAUGGCUGGAAAGAACAAAAAAUGACAUAGCUCAUCUAAUGGGAAGGAGAGAAGAAGGAGC
KF383117     UGUUACUGGUGGUGCUCAUACCCGAGCCAGAGAAGCAAAGAUCUCCCCAGGAUAAUCAAAUGGCAAUCAUCAUCAUGGUGGCAGUUGGCCUUUUGGGUUUGAUAACUGCAAAUGAACUCGGAUGGCUGGAAAGGACAAAAAGUGAUAUAGCUUAUCUGAUGGGAAGGAAAGAAGAGGGGAC
KF383116     UGUUACUGGUGGUGCUCAUACCCGAGCCAGAGAAGCAAAGAUCUCCCCAGGAUAAUCAAAUGGCAAUCAUCAUCAUGGUGGCAGUUGGCCUUCUGGGUUUGAUAACAGCAAAUGAACUCGGAUGGCUGGAAAGGACAAAAAGUGAUAUAGCUCAUCUAAUGGGAAGGAAAGAAGAGGGGAC
KF383115     UGUUACUGGUGGUACUCAUACCUGAGCCAGAGAAGCAAAGAUCUCCCCAGGAUAACCAGAUGGCAAUCAUCAUCAUGGUGGCAGUGGGCCUUCUGGGGUUGAUAACUGCAAAUGAACUUGGAUGGCUGGAAAGAACAAAAAAUGACAUAGCUCAUCUAAUGGGAAGGAGAGAAGAAGGAGU
EU545988     UAUUGCUGGUGGUGCUCAUACCUGAGCCAGAAAAGCAAAGAUCUCCCCAGGACAACCAAAUGGCAAUCAUCAUCAUGAUAGCAGUGGGUCUUCUGGGUUUGAUUACCGCCAAUGAACUUGGAUGGUUGGAAAGAACAAAGAGUGACCUAAGCCAUCUAAUGGGAAGGAGAGAGGAGGGGGC
KU955589     UAUUGCUGGUGGUGCUCAUACCUGAGCCAGAAAAGCAAAGAUCUCCCCAGGACAACCAAAUGGCAAUCAUCAUCAUGGUAGCAGUAGGUCUUCUGGGCUUGAUUACCGCCAAUGAACUCGGAUGGUUGGAGAGAACAAAGAGUGACCUAAGCCAUCUAAUGGGAAGGAGAGAGGAGGGGGC
KU955590     UAUUGCUGGUGGUGCUCAUACCUGAGCCAGAAAAGCAAAGAUCUCCCCAGGACAACCAAAUGGCAAUCAUCAUUAUGGUAGCAGUAGGUCUUCUGGGCUUGAUUACCGCCAAUGAACUCGGAUGGUUGGAGAGAACAAAGAGUGACCUAAGCCAUCUAAUGGGAAGGAGAGAGGAGGGGGC
KU940228     UAUUGCUGGUGGUGCUCAUACCUGAGCCAGAAAAGCAAAGAUCUCCCCAGGACAACCAAAUGGCAAUCAUCAUCAUGGUAGCAGUAGGUCUUCUGGGCUUGAUUACCGCCAAUGAACUCGGAUGGUUGGAGAGAACAAAGAGUGACCUAAGCCAUCUAAUGGGAAGGAGAGAGGAGGGGGC
KU991811     UAUUGCUGGUGGUGCUCAUACCUGAGCCAGAAAAGCAAAGAUCUCCCCAGGACAACCAAAUGGCAAUCAUCAUCAUGGUAGCAGUAGGUCUUCUGGGCUUGAUUACCGCCAAUGAACUCGGAUGGUUGGAGAGAACAAAGAGUGACCUAAGCCAUCUAAUGGGAAGGAGAGAGGAGGGGGC
KU509998     UAUUGCUGGUGGUGCUCAUACCUGAGCCAGAAAAGCAAAGAUCUCCCCAGGACAACCAAAUGGCAAUCAUCAUCAUGGUAGCAGUAGGUCUUCUGGGCUUGAUUACCGCCAAUGAACUCGGAUGGUUGGAGAGAACAAAGAGUGACCUAAGCCAUCUAAUGGGAAGGAGAGAGGAGGGGGC
KX056898     UAUUGCUGGUGGUGCUCAUACCUGAGCCAGAAAAGCAAAGAUCUCCCCAGGACAACCAAAUGGCAAUCAUCAUUAUGGUAGCAGUAGGUCUUCUGGGCUUGAUUACCGCCAAUGAACUCGGAUGGUUGGAGAGAACAAAGAGUGACCUAAGCCAUCUAAUGGGAAGGAGAGAGGAGGGGGC
KX117076     UAUUGCUGGUGGUGCUCAUACCUGAGCCAGAAAAGCAAAGAUCUCCCCAGGACAACCAAAUGGCAAUCAUCAUCAUGGUAGCAGUAGGUCUUCUGGGCUUGAUUACCGCCAAUGAACUCGGAUGGUUGGAGAGAACAAAGAGUGACCUAAGCCAUCUAAUGGGAAGGAGAGAGGAGGGGGC
KX185891     UAUUGCUGGUGGUGCUCAUACCUGAGCCAGAAAAGCAAAGAUCUCCCCAGGACAACCAAAUGGCAAUCAUCAUCAUGGUAGCAGUAGGUCUUCUGGGCUUGAUUACCGCCAAUGAACUCGGAUGGUUGGAGAGAACAAAGAGUGACCUAAGCCAUCUAAUGGGAAGGAGAGAGGAGGGGGC
KX247646     UAUUGCUGGUGGUGCUCAUACCUGAGCCAGAAAAGCAAAGAUCUCCCCAGGACAACCAAAUGGCAAUCAUCAUCAUGGUAGCAGUAGGUCUUCUGGGCUUGAUUACCGCCAAUGAACUCGGAUGGUUGGAGAGAACAAAGAGUGACCUAAGCCAUCUAAUGGGAAGGAGAGAGGAGGGGGC
KX262887     UAUUGCUGGUGGUGCUCAUACCUGAGCCAGAAAAGCAAAGAUCUCCCCAGGACAACCAAAUGGCAAUCAUCAUCAUGGUAGCAGUAGGUCUUCUGGGCUUGAUUACCGCCAAUGAACUCGGAUGGUUGGAGAGAACAAAGAGUGACCUAAGCCAUCUAAUGGGAAGGAGAGAGGAGGGGGC
KX280026     UAUUGCUGGUGGUGCUCAUACCUGAGCCAGAAAAGCAAAGAUCUCCCCAGGACAACCAAAUGGCAAUCAUCAUCAUGGUAGCAGUAGGUCUUCUGGGCUUGAUUACCGCCAAUGAACUCGGAUGGUUGGAGAGAACAAAGAGUGACCUAAGCCACCUAAUGGGAAGGAGAGAGGAGGGAGC
KU758877     UAUUGCUGGUGGUGCUCAUACCUGAGCCAGAAAAGCAAAGAUCUCCCCAGGACAACCAAAUGGCAAUCAUCAUCAUGGUAGCAGUAGGUCUUCUGGGCUUGAUUACCGCCAAUGAACUCGGAUGGUUGGAGAGAACAAAGAGUGACCUAAGCCAUCUAAUGGGAAGGAGAGAGGAGGGGGC
KU866423     UAUUGCUGGUGGUGCUCAUACCUGAGCCAGAAAAGCAAAGAUCUCCCCAGGACAACCAAAUGGCAAUCAUCAUCAUGGUAGCAGUAGGUCUUCUGGGCUUGAUUACCGCCAAUGAACUCGGAUGGUUGGAGAGAACAAAGAGUGACCUAAGCCAUCUAAUGGGAAGGAGAGAGGAGGGGGC
KX377336     UAUUGCUGGUGGUGCUCAUACCUGAGCCAGAAAAGCAGAGAUCUCCCCAGGACAACCAAAUGGCAAUUAUCAUCAUGGUAGCAGUGGGUCUUCUGGGCUUGAUAACCGCCAAUGAACUCGGAUGGUUGGAGAGAACAAAAAGUGACCUAGGCCAUCUAAUGGGAAGGAGAGAGGAGGGGGC
KX377337     UAUUGCUGGUGGUGCUCAUACCUGAGCCAGAAAAGCAAAGAUCUCCCCAGGACAACCAAAUGGCAAUCAUCAUCAUGGUAGCAGUAGGUCUUCUGGGCUUGAUUACCGCCAAUGAACUCGGAUGGUUGGAGAGAACAAAGAGUGACCUAAGCCAUCUAAUGGGAAGGAGAGAGGAGGGGGC
KX369547     UAUUGCUGGUGGUGCUCAUACCUGAGCCAGAAAAGCAAAGAUCUCCCCAGGACAACCAAAUGGCAAUCAUCAUCAUGGUAGCAGUAGGUCUUCUGGGCUUGAUUACCGCCAAUGAACUCGGAUGGUUGGAGAGAACAAAGAGUGACCUAAGCCAUCUAAUGGGAAGGAGAGAGGAGGGGGC
KX548902     UAUUGCUGGUGGUGCUCAUACCUGAGCCAGAAAAGCAAAGAUCUCCCCAGGACAACCAAAUGGCAAUCAUCAUCAUGGUAGCAGUAGGUCUUCUGGGCUUGAUUACCGCCAAUGAACUCGGAUGGUUGGAGAGAACAAAGAGUGACCUAAGCCAUCUAAUGGGAAGGAGAGAGGAGGGGGC
KX601166     UGUUGCUGGUGGUGCUCAUACCCGAGCCAGAGAAGCAAAGAUCUCCCCAGGAUAAUCAAAUGGCAAUCAUCAUCAUGGUGGCAGUUGGCCUUCUGGGUUUGAUAACUGCAAAUGAACUCGGAUGGCUGGAAAGGACAAAAAGUGAUAUAGCUCAUCUAAUGGGAAGGAAAGAAGAGGGGAC
KX601167     UAUUGCUGGUGGUGCUCAUACCUGAGCCAGAAAAGCAGAGAUCUCCCCAGGACAACCAAAUGGCAAUUAUCAUCAUGGUAGCAGUGGGUCUUCUGGGCUUGAUAACCGCCAAUGAACUCGGAUGGUUGGAGAGAACAAAAAGUGACCUAGGCCAUCUAAUGGGAAGGAGAGAGGAGGGGGC
KX601168     UAUUGCUGGUGGUGCUCAUACCUGAGCCAGAAAAGCAAAGAUCUCCCCAGGACAACCAAAUGGCAAUCAUCAUCAUGGUAGCAGUAGGUCUUCUGGGCUUGAUUACCGCCAAUGAACUCGGAUGGUUGGAGAGAACAAAGAGUGACCUAAGCCAUCUAAUGGGAAGGAGAGAGGAGGGGGC
KX766028     UAUUGCUGGUGGUGCUCAUACCUGAGCCAGAAAAGCAAAGAUCUCCCCAGGACAACCAAAUGGCAAUCAUCAUCAUGGUAGCAGUAGGUCUUCUGGGCUUGAUUACCGCCAAUGAACUCGGAUGGUUGGAGAGAACAAAGAGUGACCUAAGCCAUCUAAUGGGAAGGAGAGAGGAGGGGGC
KX766029     UAUUGCUGGUGGUGCUCAUACCUGAGCCAGAAAAGCAAAGAUCUCCCCAGGACAACCAAAUGGCAAUCAUCAUCAUGGUAGCAGUAGGUCUUCUGGGCUUGAUUACCGCCAAUGAACUCGGAUGGUUGGAGAGAACAAAGAGUGACCUAAGCCAUCUAAUGGGAAGGAGAGAGGAGGGGGC
KU940224     UAUUGCUGGUGGUGCUCAUACCUGAGCCAGAAAAGCAAAGAUCUCCCCAGGACAACCAAAUGGCAAUCAUCAUCAUGGUAGCAGUAGGUCUUCUGGGCUUGAUUACCGCCAAUGAACUCGGAUGGUUGGAGAGAACAAAGAGUGACCUAAGCCAUCUAAUGGGAAGGAGAGAGGAGGGGGC


             6666666666666666666666666666666666666666666666666666666666666666666666666666666666666666666666666666666666666666666666666777777777777777777777777777777777777777777777777777777777777
             8888888888888888888889999999999999999999999999999999999999999999999999999999999999999999999999999999999999999999999999999000000000000000000000000000000000000000000000000000000000000
             7888888888899999999990000000000111111111122222222223333333333444444444455555555556666666666777777777788888888889999999999000000000011111111112222222222333333333344444444445555555555
Sequence     9012345678901234567890123456789012345678901234567890123456789012345678901234567890123456789012345678901234567890123456789012345678901234567890123456789012345678901234567890123456789
---------------------------------------------------------------------------------------------------------------------------------------------------------------------------------------------------
NC_012532    AACCAUGGGAUUCUCAAUGGACAUUGAUCUGCGGCCAGCCUCCGCCUGGGCUAUCUAUGCCGCAUUGACAACUCUCAUCACCCCAGCUGUCCAACAUGCGGUAACCACUUCAUACAACAACUACUCCUUAAUGGCGAUGGCCACACAAGCUGGAGUGCUGUUUGGCAUGGGCAAAGGGAUG
KU922960     AACCAUAGGAUUCUCAAUGGACAUUGACCUGCGGCCAGCCUCAGCUUGGGCCAUCUAUGCUGCCUUGACAACUUUCAUUACCCCAGCCGUCCAACAUGCAGUGACCACUUCAUACAACAACUACUCCUUAAUGGCGAUGGCCACGCAAGCUGGAGUGUUGUUUGGUAUGGGCAAAGGGAUG
KU922923     AACCAUAGGAUUCUCAAUGGACAUUGACCUGCGGCCAGCCUCAGCUUGGGCCAUCUAUGCUGCCUUGACAACUUUCAUUACCCCAGCCGUCCAACAUGCAGUGACCACUUCAUACAACAACUACUCCUUAAUGGCGAUGGCCACGCAAGCUGGAGUGUUGUUUGGUAUGGGCAAAGGGAUG
KU820898     AACCAUAGGAUUCUCAAUGGACAUUGACCUGCGGCCAGCCUCAGCUUGGGCCAUCUAUGCUGCCUUGACAACUUUCAUUACCCCAGCCGUCCAACAUGCAGUGACCACCUCAUACAACAACUACUCCUUAAUGGCGAUGGCCACGCAAGCUGGAGUGUUGUUUGGUAUGGGCAAAGGGAUG
KU321639     AACCAUGGGAUUCUCAAUGGACAUUGACCUGCGGCCAGCCUCAGCUUGGGCCAUCUAUGCUGCCUUGACAACUUUCAUUACCCCAGCCGUCCAACAUGCAGUGACCACUUCAUACAACAACUACUCCUUAAUGGCGAUGGCCACGCAAGCUGGAGUGUUGUUUGGUAUGGGCAAAGGGAUG
KU820899     AACCAUAGGAUUCUCAAUGGACAUUGACCUGCGGCCAGCCUCAGCUUGGGCCAUCUACGCUGCCUUGACAACUUUCAUUACCCCAGCCGUCCAACAUGCAGUGACCACUUCAUACAACAACUACUCCUUAAUGGCGAUGGCCACGCAAGCUGGAGUGUUGUUUGGUAUGGGCAAAGGGAUG
KU853012     AACCAUAGGAUUCUCAAUGGACAUUGACCUGCGGCCAGCCUCAGCUUGGGCCAUCUAUGCUGCCUUGACAACUUUCAUUACCCCAGCCGUCCAACAUGCAGUGACCACUUCAUACAACAACUACUCCUUAAUGGCGAUGGCCACGCAAGCUGGAGUGUUGUUUGGUAUGGGCAAAGGGAUG
KU729217     AACCAUAGGAUUCUCAAUGGACAUUGACCUGCGGCCAGCCUCAGCUUGGGCCAUCUAUGCUGCCUUGACAACUUUCAUUACCCCAGCCGUCCAACAUGCAGUGACCACUUCAUACAACAACUACUCCUUAAUGGCGAUGGCCACGCAAGCUGGAGUGUUGUUUGGUAUGGGCAAAGGGAUG
KU729218     AACCAUAGGAUUCUCAAUGGACAUUGACCUGCGGCCAGCCUCAGCUUGGGCCAUCUAUGCUGCCUUGACAACUUUCAUUACCCCAGCCGUCCAACAUGCAGUGACCACUUCAUACAACAACUACUCCUUAAUGGCGAUGGCCACGCAAGCUGGAGUGUUGUUUGGUAUGGGCAAAGGGAUG
KU761564     AACCAUAGGAUUCUCAAUGGACAUUGACCUGCGGCCAGCCUCAGCUUGGGCCAUCUAUGCUGCCUUGACAACUUUCAUUACCCCAGCCGUCCAACAUGCAGUGACCACCUCAUACAACAACUACUCCUUAAUGGCGAUGGCCACGCAAGCUGGAGUGUUGUUUGGUAUGGGCAAAGGGAUG
KU740184     AACCAUAGGAUUCUCAAUGGACAUUGACCUGCGGCCAGCCUCAGCUUGGGCCAUCUAUGCUGCCUUGACAACUUUCAUUACCCCAGCCGUCCAACAUGCAGUGACCACCUCAUACAACAACUACUCCUUAAUGGCGAUGGCCACGCAAGCUGGAGUGUUGUUUGGUAUGGGCAAAGGGAUG
KU744693     AACCAUGGGAUUCUCAAUGGACAUUGACCUGCGGCCAGCCUCAGCUUGGGCCAUCUAUCCUGCCUUGACAUCUUUCAUUACCCCAGCCGUCCAACAUGCAGUGACCACUUCAUACAACAACUACUCCUUAAUGGCGAUGGCCACGCAAGCUGGAGUGUUGUUUGGUAUGGGCAAAGGGAUG
KU497555     AACCAUAGGAUUCUCAAUGGACAUUGACCUGCGGCCAGCCUCAGCUUGGGCCAUCUAUGCUGCCUUGACAACUUUCAUUACCCCAGCCGUCCAACAUGCAGUGACCACUUCAUACAACAACUACUCCUUAAUGGCGAUGGCCACGCAAGCUGGAGUGUUGUUUGGUAUGGGCAAAGGGAUG
KU527068     AACCAUAGGAUUCUCAAUGGACAUUGACCUGCGGCCAGCCUCAGCUUGGGCCAUCUAUGCUGCCUUGACAACUUUCAUUACCCCAGCCGUCCAACAUGCAGUGACCACUUCAUACAACAACUACUCCUUAAUGGCGAUGGCCACGCAAGCUGGAGUGUUGUUUGGUAUGGGCAAAGGGAUG
```

```
KU501217    AACCAUAGGAUUCUCAAUGGACAUUGACCUGCGGCCAGCCUCAGCUUGGGCCAUCUAUGCUGCCUUGACAACUUUCAUUACCCCAGCCGUCCAACAUGCAGUGACCACUUCAUACAACAACUACUCCUUAAUGGCGAUGGCCACGCAAGCUGGAGUGUUGUUUGGUAUGGGCAAAGGAUG
KU501216    AACCAUAGGAUUCUCAAUGGACAUUGACCUGCGGCCAGCCUCAGCUUGGGCCAUCUAUGCUGCCUUGACAACUUUCAUUACCCCAGCCGUCCAACAUGCAGUGACCACUUCAUACAACAACUACUCCUUAAUGGCGAUGGCCACGCAAGCUGGAGUGUUGUUUGGUAUGGGCAAAGGAUG
KU501215    AACCAUAGGAUUCUCAAUGGACAUUGACCUGCGGCCAGCCUCAGCUUGGGCCAUCUAUGCUGCCUUGACAACUUUCAUUACCCCAGCCGUCCAACAUGCAGUGACCACCUCAUACAACAACUACUCCUUAAUGGCGAUGGCCACGCAAGCUGGAGUGUUGUUUGGCAUGGGCAAAGGAUG
KU365780    AACCAUAGGAUUCUCAAUGGACAUUGACCUGCGGCCAGCCUCAGCUUGGGCCAUCUAUGCUGCCUUGACAACUUUCAUUACCCCAGCCGUCCAACAUGCAGUGACCACUUCAUACAACAACUACUCCUUAAUGGCGAUGGCCACGCAAGCUGGAGUGUUGUUUGGUAUGGGCAAAGGAUG
KU365779    AACCAUAGGAUUCUCAAUGGACAUUGACCUGCGGCCAGCCUCAGCUUGGGCCAUCUAUGCUGCCUUGACAACUUUCAUUACCCCAGCCGUCCAACAUGCAGUGACCACUUCAUACAACAACUACUCCUUAAUGGCGAUGGCCACGCAAGCUGGAGUGUUGUUUGGUAUGGGCAAAGGAUG
KU707826    AACCAUAGGAUUCUCAAUGGACAUUGACCUGCGGCCAGCCUCAGCUUGGGCCAUCUAUGCUGCCUUGACAACUUUCAUUACCCCAGCCGUCCAACAUGCAGUGACCACUUCAUACAACAACUACUCCUUAAUGGCGAUGGCCACGCAAGCUGGAGUGUUGUUUGGUAUGGGCAAAGGAUG
KU365778    AACCAUAGGAUUCUCAAUGGACAUUGACCUGCGGCCAGCCUCAGCUUGGGCCAUCUAUGCUGCCUUGACAACUUUCAUUACCCCAGCCGUCCAACAUGCAGUGACCACCUCAUACAACAACUACUCCUUAAUGGCGAUGGCCACGCAAGCUGGAGUGUUGUUUGGUAUGGGCAAAGGAUG
KU365777    AACCAUAGGAUUCUCAAUGGACAUUGACCUGCGGCCAGCCUCAGCUUGGGCCAUCUAUGCUGCCUUGACAACUUUCAUUACCCCAGCCGUCCAACAUGCAGUGACCACUUCAUACAACAACUACUCCUUAAUGGCGAUGGCCACGCAAGCUGGAGUGUUGUUUGGUAUGGGCAAAGGAUG
KU312312    AACCAUAGGAUUCUCAAUGGACAUUGACCUGCGGCCAGCCUCAGCUUGGGCCAUCUAUGCUGCCUUGACAACUUUCAUUACCCCAGCCGUCCAACAUGCAGUGACCACCUCAUACAACAACUACUCCUUAAUGGCGAUGGCCACGCAAGCUGGAGUGUUGUUUGGUAUGGGCAAAGGAUG
KF268950    AACUAUGGGAUUCUCAAUGGACAUCGAUUUGCGGCCAGCCUCCGCCUGGGCUAUCUAUGCCGCACUGACAACUCUCAUCACCCCAGCCGUCCAACACGCGGUAACCACUUCAUACAACAACUACUCCCUAAUGGCGAUGGCCACACAAGCUGGAGUGCUAUUUGGCAUGGGCAAAGGAUG
KF268949    AACUAUGGGAUUCUCAAUGGACAUCGAUUUGCGGCCAGCCUCCGCCUGGGCUAUUUAUGCCGCACUGACAACUCUCAUCACCCCAGCUGUCCAACACGCGGUAACCACUUCAUACAACAACUACUCCCUAAUGGCGAUGGCCACACAAGCUGGAGUGCUAUUUGGCAUGGGCAAAGGAUG
KF268948    AACUAUGGGAUUCUCAAUGGACAUCGAUUUGCGGCCAGCCUCCGCCUGGGCUAUCUAUGCCGCACUGACAACUCUCAUCACCCCAGCCGUCCAACACGCGGUAACCACUUCAUACAACAACUACUCCCUAAUGGCGAUGGCCACACAAGCUGGAGUGCUAUUUGGCAUGGGCAAAGGAUG
KJ776791    AACCAUAGGAUUCUCAAUGGACAUUGACCUGCGGCCAGCCUCAGCUUGGGCCAUCUAUGCUGCCUUGACAACUUUCAUUACCCCAGCCGUCCAACAUGCAGUGACCACUUCAUACAACAACUACUCCUUAAUGGCGAUGGCCACGCAAGCUGGAGUGUUGUUUGGUAUGGGCAAAGGAUG
KF383119    AACCAUGGGAUUCUCAAUGGACAUUGAUCUGCGGCCAGCCUCCGCCUGGGCUAUCUAUGCCGCAUUGACAACUCUCAUCACCCCAGCUGUCCAACAUGCGGUAACCACUUCAUACAACAACUACUCCUUAAUGGCGAUGGCCACACAAGCUGGAGUGCUGUUUGGCAUGGGCAAAGGAUG
KF383118    AACCAUGGGAUUCUCAAUGGACAUUGAUCUGCGGCCAGCCUCCGCCUGGGCUAUCUAUGCCGCAUUGACAACUCUCAUCACCCCAGCUGUCCAACAUGCGGUAACCACUUCAUACAACAACUACUCCUUAAUGGCGAUGGCCACACAAGCUGGAGUGCUGUUUGGCAUGGGCAAAGGAUG
KF383117    AACCAUAGGAUUCUCAAUGGAUAUUGAUCUGCGGCCAGCCUCUGCCUGGGCUAUUUAUGCCGCAUUGACAACUCUCAUCACCCCAGCCGUCCAACAUGCGGUAACCACCUCAUACAACAACUACUCCCUGAUGGCGAUGGCCACACAAGCUGGAGUGCUGUUUGGCAUGGGCAAAGGAUG
KF383116    AACCAUAGGAUUCUCAAUGGAUAUUGAUCUGCGGCCAGCCUCCGCCUGGGCUAUUUAUGCCGCAUUGACAACUCUCAUCACCCCAGCCGUCCAACAUGCGGUAACCACCUCAUACAACAACUACUCCCUGAUGGCGAUGGCCACACAAGCUGGAGUGCUGUUUGGCAUGGGCAAAGGAUG
KF383115    AACUAUGGGAUUCUCAAUGGACAUCGAUUUGAGGCCGGCCUCCGCCUGGGCUAUCUAUGCUGCAUUGACAACUCUCAUCACCCCAGCCGUCCAACAUGCGGUAACCACUUCAUAUAACAACUACUCCUUAAUGGCGAUGGCCACACAAGCUGGAGUGCUGUUUGGCAUGGGCAAAGGAUG
EU545988    AACCAUAGGAUUCUCAAUGGACAUUGACCUGCGGCCAGCCUCAGCUUGGGCUAUCUAUGCUGCUCUGACAACUUUCAUCACCCCAGCCGUCCAACAUGCGGUGACCACUUCAUACAACAACUACUCCUUAAUGGCGAUGGCCACGCAAGCUGGAGUGUUGUUUGGUAUGGGUAAAGGAUG
KU955589    AACCAUAGGAUUCUCAAUGGACAUUGACCUGCGGCCAGCCUCAGCUUGGGCCAUCUACGCUGCCUUGACAACUUUCAUUACCCCAGCCGUCCAACAUGCAGUGACCACUUCAUACAACAACUACUCCUUAAUGGCGAUGGCCACGCAAGCUGGAGUGUUGUUUGGUAUGGGCAAAGGAUG
KU955590    AACCAUAGGAUUCUCAAUGGACAUUGACCUGCGGCCAGCCUCAGCUUGGGCCAUCUAUGCUGCCUUGACAACUUUCAUUACCCCAGCCGUCCAACAUGCAGUGACCACCUCAUACAACAACUACUCCUUAAUGGCGAUGGCCACGCAAGCUGGAGUGUUGUUUGGUAUGGGCAAAGGAUG
KU940228    AACCAUAGGAUUCUCAAUGGACAUUGACCUGCGGCCAGCCUCAGCUUGGGCCAUCUAUGCUGCCUUGACAACUUUCAUUACCCCAGCCGUCCAACAUGCAGUGACCACUUCAUACAACAACUACUCCUUAAUGGCGAUGGCCACGCAAGCUGGAGUGUUGUUUGGUAUGGGCAAAGGAUG
KU991811    AACCAUAGGAUUCUCAAUGGACAUUGACCUGCGGCCAGCCUCAGCUUGGGCCAUCUAUGCUGCCUUGACAACUUUCAUUACCCCAGCCGUCCAACAUGCAGUGACCACUUCAUACAACAACUACUCCUUAAUGGCGAUGGCCACGCAAGCUGGAGUGUUGUUUGGUAUGGGCAAAGGAUG
KU509998    AACCAUGGGAUUCUCAAUGGACAUUGACCUGCGGCCAGCCUCAGCUUGGGCCAUCUAUGCUGCCUUGACAACUUUCAUUACCCCAGCCGUCCAACAUGCAGUGACCACUUCAUACAACAACUACUCCUUAAUGGCGAUGGCCACGCAAGCUGGAGUGUUGUUUGGUAUGGGCAAAGGAUG
KX056898    AACCAUAGGAUUCUCAAUGGACAUUGACCUGCGGCCAGCCUCAGCUUGGGCCAUCUAUGCUGCCUUGACAACUUUCAUUACCCCAGCCGUCCAACAUGCAGUGACCACCUCAUACAACAACUACUCCUUAAUGGCGAUGGCCACGCAAGCUGGAGUGUUGUUUGGUAUGGGCAAAGGAUG
KX117076    AACCAUAGGAUUCUCAAUGGACAUUGACCUGCGGCCAGCCUCAGCUUGGGCCAUCUACGCUGCCUUGACAACUUUCAUUACCCCAGCCGUCCAACAUGCAGUGACCACUUCAUACAACAACUACUCCUUAAUGGCGAUGGCCACGCAAGCUGGAGUGUUGUUUGGUAUGGGCAAAGGAUG
KX185891    AACCAUAGGAUUCUCAAUGGACAUUGACCUGCGGCCAGCCUCAGCUUGGGCCAUCUACGCUGCCUUGACAACUUUCAUUACCCCAGCCGUCCAACAUGCAGUGACCACUUCAUACAACAACUACUCCUUAAUGGCGAUGGCCACGCAAGCUGGAGUGUUGUUUGGUAUGGGCAAAGGAUG
KX247646    AACCAUAGGAUUCUCAAUGGACAUUGACCUGCGGCCAGCCUCAGCUUGGGCCAUCUAUGCUGCCUUGACAACUUUCAUUACCCCAGCCGUCCAACAUGCAGUGACCACUUCAUACAACAACUACUCCUUAAUGGCGAUGGCCACGCAAGCUGGAGUGUUGUUUGGUAUGGGCAAAGGAUG
KX262887    AACCAUAGGAUUCUCAAUGGACAUUGACCUGCGGCCAGCCUCAGCUUGGGCCAUCUAUGCUGCCUUGACAACUUUCAUUACCCCAGCCGUCCAACAUGCAGUGACCACUUCAUACAACAACUACUCCUUAAUGGCGAUGGCCACGCAAGCUGGAGUGUUGUUUGGUAUGGGCAAAGGAUG
KX280026    AACCAUAGGAUUCUCAAUGGACAUUGACCUGCGGCCAGCCUCAGCUUGGGCCAUCUAUGCUGCCUUGACAACUUUCAUUACCCCAGCCGUCCAACAUGCAGUGACCACUUCAUACAACAACUACUCCUUAAUGGCGAUGGCCACGCAAGCUGGAGUGUUGUUUGGUAUGGGCAAAGGAUG
KU758877    AACCAUAGGAUUCUCAAUGGACAUUGACCUGCGGCCAGCCUCAGCUUGGGCCAUCUAUGCUGCCUUGACAACUUUCAUUACCCCAGCCGUCCAACAUGCAGUGACCACCUCAUACAACAACUACUCCUUAAUGGCGAUGGCCACGCAAGCUGGAGUGUUGUUUGGUAUGGGCAAAGGAUG
KU866423    AACCAUAGGAUUCUCAAUGGACAUUGACCUGCGGCCAGCCUCAGCUUGGGCCAUCUACGCUGCCUUGACAACUUUCAUUACCCCAGCCGUCCAACAUGCAGUGACCACUUCAUACAACAACUACUCCUUAAUGGCGAUGGCCACGCAAGCUGGAGUGUUGUUUGGUAUGGGCAAAGGAUG
KX377336    AACCAUGGGAUUCUCAAUGGACAUUGACUUGCGGCCAGCCUCAGCUUGGGCUAUCUAUGCCGCUCUGACAACUCUCAUCACCCCAGCCGUCCAACAUGCGGUAACCACUUCAUACAACAACUACUCCUUAAUGGCGAUGGCCACGCAAGCCGGAGUGUUGUUUGGCAUGGGCAAAGGAUG
KX377337    AACCAUAGGAUUCUCAAUGGACAUUGACCUGCGGCCAGCCUCAGCUUGGGCCAUCUAUGCUGCCUUGACAACUUUCAUUACCCCAGCCGUCCAACAUGCAGUGACCACCUCAUACAACAACUACUCCUUAAUGGCGAUGGCCACGCAAGCUGGAGUGUUGUUUGGCAUGGGCAAAGGAUG
KX369547    AACCAUAGGAUUCUCAAUGGACAUUGACCUGCGGCCAGCCUCAGCUUGGGCCAUCUAUGCUGCCUUGACAACUUUCAUUACCCCAGCCGUCCAACAUGCAGUGACCACUUCAUACAACAACUACUCCUUAAUGGCGAUGGCCACGCAAGCUGGAGUGUUGUUUGGUAUGGGCAAAGGAUG
KX548902    AACCAUAGGAUUCUCAAUGGACAUUGACCUGCGGCCAGCCUCAGCUUGGGCCAUCUAUGCUGCCUUGACAACUUUCAUUACCCCAGCCGUCCAACAUGCAGUGACCACUUCAUACAACAACUACUCCUUAAUGGCGAUGGCCACGCAAGCUGGAGUGUUGUUUGGUAUGGGCAAAGGAUG
KX601166    AACCAUGGGAUUCUCAAUGGAUAUUGAUCUGCGGCCAGCCUCCGCCUGGGCUAUUUAUGCCGCAUUGACAACUCUCAUCACCCCAGCCGUCCAACAUGCGGUAACCACCUCAUACAACAACUACUCCCUGAUGGCGAUGGCCACACAAGCUGGAGUGCUGUUUGGCAUGGGCAAAGGAUG
KX601167    AACCAUGGGAUUCUCAAUGGACAUUGACUUGCGGCCAGCCUCAGCUUGGGCUAUCUAUGCCGCUCUGACAACUCUCAUCACCCCAGCCGUCCAACAUGCGGUAACCACUUCAUACAACAACUACUCCUUAAUGGCGAUGGCCACGCAAGCCGGAGUGUUGUUUGGCAUGGGCAAAGGAUG
KX601168    AACCAUAGGAUUCUCAAUGGACAUUGACCUGCGGCCAGCCUCAGCUUGGGCCAUCUAUGCUGCCUUGACAACUUUCAUUACCCCAGCCGUCCAACAUGCAGUGACCACCUCAUACAACAACUACUCCUUAAUGGCGAUGGCCACGCAAGCUGGAGUGUUGUUUGGCAUGGGCAAAGGAUG
KX766028    AACCAUAGGAUUCUCAAUGGACAUUGACCUGCGGCCAGCCUCAGCUUGGGCCAUCUAUGCUGCCUUGACAACUUUCAUUACCCCAGCCGUCCAACAUGCAGUGACCACCUCAUACAACAACUACUCCUUAAUGGCGAUGGCCACGCAAGCUGGAGUGUUGUUUGGUAUGGGCAAAGGAUG
KX766029    AACCAUAGGAUUCUCAAUGGACAUUGACCUGCGGCCAGCCUCAGCUUGGGCCAUCUAUGCUGCCUUGACAACUUUCAUUACCCCAGCCGUCCAACAUGCAGUGACCACUUCAUACAACAACUACUCCUUAAUGGCGAUGGCCACGCAAGCUGGAGUGUUGUUUGGUAUGGGCAAAGGAUG
KU940224    AACCAUAGGAUUCUCAAUGGACAUUGACCUGCGGCCAGCCUCAGCUUGGGCCAUCUAUGCUGCCUUGACAACUUUCAUUACCCCAGCCGUCCAACAUGCAGUGACCACUUCAUACAACAACUACUCCUUAAUGGCGAUGGCCACGCAAGCUGGAGUGUUGUUUGGUAUGGGCAAAGGAUG


            7777777777777777777777777777777777777777777777777777777777777777777777777777777777777777777777777777777777777777777777777777777777777777777777777777777777777777777777777777777
            0000000000000000000000000000000000000000111111111111111111111111111111111111111111111111111111111111111111111111111111111111111111111111111111112222222222222222222222222222222222222222
            6666666666777777777788888888889999999999000000000011111111112222222222333333333344444444445555555555666666666677777777778888888888999999999900000000001111111111222222222233333333334
Sequence    0123456789012345678901234567890123456789012345678901234567890123456789012345678901234567890123456789012345678901234567890123456789012345678901234567890123456789012345678901234567890
---------------------------------------------------------------------------------------------------------------------------------------------------------------------------------------------------------
NC_012532   CCAUUUAUGCAUGGGGACCUUGGAGUCCCGCUGCUAAUGAUGGGUUGCUAUUCACAAUUAACACCCCUGACUCUGAUAGUAGCUAUCAUUCUGCUUGUGGCGCACUACAUGUACUUGAUCCCAGGCCUACAAGCGGCAGCAGCGCGUGCUGCCCAGAAAAGGACAGCAGCUGGCAUCAUGA
KU922960    CCAUUCUACGCAUGGGACUUUGGAGUCCCACUGCUAAUGAUAGGUUGCUACUCACAAUUAACACCCCUGACCCUAAUAGUGGCCAUCAUUUUGCUCGUGGCGCACUACAUGUACUUGAUCCCAGGGCUGCAGGCAGCAGCUGCGCGUGCUGCCCAGAAGAGAACGGCAGCUGGCAUCAUGA
KU922923    CCAUUCUACGCAUGGGACUUUGGAGUCCCACUGCUAAUGAUAGGUUGCUACUCACAAUUAACACCCCUGACCCUAAUAGUGGCCAUCAUUUUGCUCGUGGCGCACUACAUGUACUUGAUCCCAGGGCUGCAGGCAGCAGCUGCGCGUGCUGCCCAGAAGAGAACGGCAGCUGGCAUCAUGA
KU820898    CCAUUCUACGCAUGGGACUUUGGAGUCCCGCUGCUAAUGAUAGGCUGCUACUCACAAUUAACACCCCUGACCCUAAUAGUGGCCAUCAUUUUGCUCGUGGCGCACUACAUGUACUUGAUCCCAGGGCUGCAGGCAGCAGCUGCGCGUGCUGCCCAGAAGAGAACGGCAGCUGGCAUCAUGA
KU321639    CCAUUCUACGCAUGGGACUUUGGAGUCCCGCUGCUAAUGAUAGGUUGCUACUCACAAUUAACGCCCCUGACCCUAAUAGUGGCCAUCAUUUUGCUCGUGGCGCACUACAUGUACUUGAUCCCAGGGCUGCAGGCAGCAGCUGCGCGUGCUGCCCAGAAGAGAACGGCAGCUGGCAUCAUGA
KU820899    CCAUUCUACGCAUGGGACUUUGGAGUCCCGCUGCUAAUGAUAGGUUGCUACUCACAAUUAACACCCCUGACCCUAAUAGUAGCCAUCAUUUUGCUCGUGGCGCACUACAUGUACUUGAUCCCAGGGCUGCAGGCAGCAGCUGCGCGUGCUGCCCAGAAGAGAACGGCAGCUGGCAUCAUGA
KU853012    CCAUUCUACGCAUGGGACUUUGGAGUCCCGCUGCUAAUGAUAGGUUGCUACUCACAAUUAACACCCCUGACCCUAAUAGUGGCCAUCAUUUUGCUCGUGGCGCACUACAUGUACUUGAUCCCAGGGCUGCAGGCAGCAGCUGCGCGUGCUGCCCAGAAGAGAACGGCAGCUGGCAUCAUGA
KU729217    CCAUUCUACGCAUGGGACUUUGGAGUCCCGCUGCUAAUGAUAGGUUGCUACUCACAAUUAACACCCCUGACCCUAAUAGUGGCCAUCAUUUUGCUCGUGGCGCACUACAUGUACUUGAUCCCAGGGCUGCAGGCAGCAGCUGCGCGUGCUGCCCAGAAGAGAACGGCAGCUGGCAUCAUGA
KU729218    CCAUUCUACGCAUGGGACUUUGGAGUCCCGCUGCUAAUGAUAGGUUGCUACUCACAAUUAACACCCCUGACCCUAAUAGUGGCCAUCAUUUUGCUCGUGGCGCACUACAUGUACUUGAUCCCAGGGCUGCAGGCAGCAGCUGCGCGUGCUGCCCAGAAGAGAACGGCAGCUGGCAUCAUGA
KU761564    CCAUUCUACGCAUGGGACUUUGGAGUCCCGCUGCUAAUGAUAGGCUGCUACUCACAAUUAACACCCCUGACCCUAAUAGUGGCCAUCAUUUUGCUCGUGGCGCACUACAUGUACUUGAUCCCAGGGCUGCAGGCAGCAGCUGCGCGUGCUGCCCAGAAGAGAACGGCAGCUGGCAUCAUGA
KU740184    CCAUUCUACGCAUGGGACUUUGGAGUCCCGCUGCUAAUGAUAGGCUGCUACUCACAAUUAACACCCCUGACCCUAAUAGUGGCCAUCAUUUUGCUCGUGGCGCACUACAUGUACUUGAUCCCAGGGCUGCAGGCAGCAGCUGCGCGUGCUGCCCAGAAGAGAACGGCAGCUGGCAUCAUGA
KU744693    CCAUUCUACGCAUGGGACUUUGGAGUCCCGCUGCUAAUGAUAGGUUGCUACUCACAAUUAACGCCCCUGACCCUAAUAGUGGCCAUCAUUUUGCUCGUGGCGCACUACAUGUACUUGAUCCCAGGGCUGCAGGCAGCAGCUGCGCGUGCUGCCCAGAAGAGAACGGCAGCUGGCAUCAUGA
KU497555    CCAUUCUACGCAUGGGACUUUGGAGUCCCGCUGCUAAUGAUAGGUUGCUACUCACAAUUAACACCCCUGACCCUAAUAGUGGCCAUCAUUUUGCUCGUGGCGCACUACAUGUACUUGAUCCCAGGGCUGCAGGCAGCAGCUGCGCGUGCUGCCCAGAAGAGAACGGCAGCUGGCAUCAUGA
KU527068    CCAUUCUACGCAUGGGACUUUGGAGUCCCGCUGCUAAUGAUAGGUUGCUACUCACAAUUAACACCCCUGACCCUAAUAGUGGCCAUCAUUUUGCUCGUGGCGCACUACAUGUACUUGAUCCCAGGGCUGCAGGCAGCAGCUGCGCGUGCUGCCCAGAAGAGAACGGCAGCUGGCAUCAUGA
KU501217    CCAUUCUACGCAUGGGACUUUGGAGUCCCGCUGCUAAUGAUAGGUUGCUACUCACAAUUAACACCCCUGACCCUAAUAGUGGCCAUCAUUUUGCUCGUGGCGCACUACAUGUACUUGAUCCCAGGGCUGCAGGCAGCAGCUGCGCGUGCUGCCCAGAAGAGAACGGCAGCUGGCAUCAUGA
KU501216    CCAUUCUACGCAUGGGACUUUGGAGUCCCGCUGCUAAUGAUAGGUUGCUACUCACAAUUAACACCCCUGACCCUAAUAGUGGCCAUCAUUUUGCUCGUGGCGCACUACAUGUACUUGAUCCCAGGGCUGCAGGCAGCAGCUGCGCGUGCUGCCCAGAAGAGAACGGCAGCUGGCAUCAUGA
KU501215    CCAUUCUACGCAUGGGACUUUGGAGUCCCGCUGCUAAUGAUAGGUUGCUACUCACAAUUAACACCCCUGACCCUAAUAGUGGCCAUCAUUUUGCUCGUGGCGCACUACAUGUACUUGAUCCCAGGGCUGCAGGCAGCAGCUGCGCGUGCUGCCCAGAAGAGAACGGCAGCUGGCAUCAUGA
KU365780    CCAUUCUACGCAUGGGACUUUGGAGUCCCGCUGCUAAUGAUAGGUUGCUACUCACAAUUAACACCCCUGACCCUAAUAGUGGCCAUCAUUUUGCUCGUGGCGCACUACAUGUACUUGAUCCCAGGGCUGCAGGCAGCAGCUGCGCGUGCUGCCCAGAAGAGAACGGCAGCUGGCAUCAUGA
KU365779    CCAUUCUACGCAUGGGACUUUGGAGUCCCGCUGCUAAUGAUAGGUUGCUACUCACAAUUAACACCCCUGACCCUAAUAGUGGCCAUCAUUUUGCUCGUGGCGCACUACAUGUACUUGAUCCCAGGGCUGCAGGCAGCAGCUGCGCGUGCUGCCCAGAAGAGAACGGCAGCUGGCAUCAUGA
KU707826    CCAUUCUACGCAUGGGACUUUGGAGUCCCGCUGCUAAUGAUAGGUUGCUACUCACAAUUAACACCCCUGACCCUAAUAGUGGCCAUCAUUUUGCUCGUGGCGCACUACAUGUACUUGAUCCCAGGGCUGCAGGCAGCAGCUGCGCGUGCUGCCCAGAAGAGAACGGCAGCUGGCAUCAUGA
KU365778    CCAUUCUACGCAUGGGACUUUGGAGUCCCGCUGCUAAUGAUAGGUUGCUACUCACAAUUAACACCCCUGACCCUAAUAGUGGCCAUCAUUUUGCUCGUGGCGCACUACAUGUACUUGAUCCCAGGGCUGCAGGCAGCAGCUGCGCGUGCUGCCCAGAAGAGAACGGCAGCUGGCAUCAUGA
KU365777    CCAUUCUACGCAUGGGACUUUGGAGUCCCGCUGCUAAUGAUAGGUUGCUACUCACAAUUAACACCCCUGACCCUAAUAGUGGCCAUCAUUUUGCUCGUGGCGCACUACAUGUACUUGAUCCCAGGGCUGCAGGCAGCAGCUGCGCGUGCUGCCCAGAAGAGAACGGCAGCUGGCAUCAUGA
KU312312    CCAUUCUACGCAUGGGACUUUGGAGUCCCGCUGCUAAUGAUAGGUUGCUACUCACAAUUAACACCCCUGACCCUAAUAGUGGCCAUCAUUUUGCUCGUGGCGCACUACAUGUACUUGAUCCCAGGGCUGCAGGCAGCAGCUGCGCGUGCUGCCCAGAAGAGAACGGCAGCUGGCAUCAUGA
KF268950    CCAUUUUAUGCAUGGGACUUUGGAGUCCCGCUGCUAAUGAUGGGUUGCUACUCACAAUUAACACCCCUAACCUUGAUAGUGGCUAUCAUUCUGCUUGUGGCACACUAUAUGUACUUGAUCCCAGGCCUACAGGCAGCAGCAGCGCGUGCUGCCCAGAAGAGAACAGCAGCUGGCAUCAUGA
KF268949    CCAUUUUAUGCAUGGGACUUUGGAGUCCCGCUGCUAAUGGUGGGUUGCUACUCACAAUUAACACCCCUAACCUUGAUAGUGGCUAUCAUUCUGCUUGUGGCACACUACAUGUACUUGAUCCCAGGCCUACAGGCAGCAGCAGCGCGUGCUGCCCAGAAGAGAACAGCAGCUGGCAUCAUGA
KF268948    CCAUUUUAUGCAUGGGACUUUGGAGUCCCGCUGCUAAUGAUGGGUUGCUACUCACAAUUAACACCCCUAACCUUGAUAGUGGCUAUCAUUCUGCUUGUGGCACACUAUAUGUACUUGAUCCCAGGCCUACAGGCAGCAGCAGCGCGUGCUGCCCAGAAGAGAACAGCAGCUGGCAUCAUGA
KJ776791    CCAUUCUACGCAUGGGACUUUGGAGUCCCGCUGCUAAUGAUAGGUUGCUACUCACAAUUAACACCCCUGACCCUAAUAGUGGCCAUCAUUUUGCUCGUGGCGCACUACAUGUACUUGAUCCCAGGGCUGCAGGCAGCAGCUGCGCGUGCUGCCCAGAAGAGAACGGCAGCUGGCAUCAUGA
KF383119    CCAUUUUAUGCAUGGGACUUUGGAGUCCCGCUGCUAAUGAUGGGUUGCUAUUCACAAUUAACACCCCUGACUCUGAUAGUAGCUAUCAUUCUGCUUGUGGCGCACUACAUGUACUUGAUCCCAGGCCUACAAGCGGCAGCAGCGCGUGCUGCCCAGAAAAGGACAGCAGCUGGCAUCAUGA
KF383118    CCAUUUAUGCAUGGGGACCUUGGAGUCCCGCUGCUAAUGAUGGGUUGCUAUUCACAAUUAACACCCCUGACUCUGAUAGUAGCUAUCAUUCUGCUUGUGGCGCACUACAUGUACUUGAUCCCAGGCCUACAAGCGGCAGCAGCGCGUGCUGCCCAGAAAAGGACAGCAGCUGGCAUCAUGA
```

```
KF383117    CCAUUUUAUGCAUGGGACUUUGGAGUCCCGCUGCUAAUGAUAGGUUGCUACUCACAGUUAACACCCCUGACCCUGAUAGUAGCCAUCAUUCUGCUUGUAGCACACUACAUGUACUUGAUCCCAGGUUUGCAGGCAGCAGCAGCACGUGCUGCCCAGAAGAGGACAGCAGCUGGCAUCAUGA
KF383116    CCAUUUUAUGCAUGGGACUUUGGAGUCCCGCUGCUAAUGAUGGGUUGCUACUCACAAUUAACACCCCUGACCCUGAUAGUGGCCAUCAUUCUGCUUGUGGCACACUACAUGUACUUGAUCCCAGGUUUGCAGGCAGCAGCACGUGCUGCCCAGAAGAGGACAGCAGCUGGCAUCAUGA
KF383115    CCAUUUUAUGCAUGGGACUUUGGAGUCCCGCUGCUAAUGAUGGGUUGCUACUCACAAUUAACACCCCUGACCCUGAUAGUGGCUAUCAUUCUGCUUGUGGCACACUACAUGUACUUGAUCCCAGGCCUACAGGCAGCAGCAGCGCGUGCUGCCCAGAAGAGAACAGCAGCUGGCAUCAUGA
EU545988    CCAUUCUACGCAUGGGACUUUGGAGUCCCGCUGCUAAUGAUGGGUUGCUACUCACAAUUAACACCCCUGACCCUAAUAGUGGCCAUCAUUUUGCUCGUGGCGCACUACAUGUACUUGAUCCCAGGGCUGCAGGCAGCAGCUGCGCGUGCUGCCCAGAAGAGAACGGCAGCUGGCAUCAUGA
KU955589    CCAUUCUACGCAUGGGACUUUGGAGUCCCGCUGCUAAUGAUAGGUUGCUACUCACAAUUAACACCCCUGACCCUAAUAGUAGCCAUCAUUUUGCUCGUGGCGCACUACAUGUACUUGAUCCCAGGGCUGCAGGCAGCAGCUGCGCGUGCUGCCCAGAAGAGAACGGCAGCUGGCAUCAUGA
KU955590    CCAUUCUACGCAUGGGACUUUGGAGUCCCGCUGCUAAUGAUAGGUUGCUACUCACAAUUAACACCCCUGACCCUAAUAGUGGCCAUCAUUUUGCUCGUGGCGCACUACAUGUACUUGAUCCCAGGGCUGCAGGCAGCAGCUGCGCGUGCUGCCCAGAAGAGAACGGCAGCUGGCAUCAUGA
KU940228    CCAUUCUACGCAUGGGACUUUGGAGUCCCGCUGCUAAUGAUAGGUUGCUACUCACAAUUAACACCCCUGACCCUAAUAGUGGCCAUCAUUUUGCUCGUGGCGCACUACAUGUACUUGAUCCCAGGGCUGCAGGCAGCAGCUGCGCGUGCUGCCCAGAAGAGAACGGCAGCUGGCAUCAUGA
KU991811    CCAUUCUACGCAUGGGACUUUGGAGUCCCGCUGCUAAUGAUAGGUUGCUACUCACAAUUAACACCCCUGACCCUAAUAGUGGCCAUCAUUUUGCUCGUGGCGCACUACAUGUACUUGAUCCCAGGGCUGCAGGCAGCAGCUGCGCGUGCUGCCCAGAAGAGAACGGCAGCUGGCAUCAUGA
KU509998    CCAUUCUACGCAUGGGACUUUGGAGUCCCGCUGCUAAUGAUAGGUUGCUACUCACAAUUAACGCCCCUGACCCUAAUAGUGGCCAUCAUUUUGCUCGUGGCGCACUACAUGUACUUGAUCCCAGGGCUGCAGGCAGCAGCUGCGCGUGCUGCCCAGAAGAGAACGGCAGCUGGCAUCAUGA
KX056898    CCAUUCUACGCAUGGGACUUUGGAGUCCCGCUGCUAAUGAUAGGUUGCUACUCACAAUUAACACCCCUGACCCUAAUAGUGGCCAUCAUUUUGCUCGUGGCGCACUACAUGUACUUGAUCCCAGGGCUGCAGGCAGCAGCUGCGCGUGCUGCCCAGAAGAGAACGGCAGCUGGCAUCAUGA
KX117076    CCAUUCUACGCAUGGGACUUUGGAGUCCCGCUGCUAAUGAUAGGUUGCUACUCACAAUUAACACCCCUGACCCUAAUAGUAGCCAUCAUUUUGCUCGUGGCGCACUACAUGUACUUGAUCCCAGGGCUGCAGGCAGCAGCUGCGCGUGCUGCCCAGAAGAGAACGGCAGCUGGCAUCAUGA
KX185891    CCAUUCUACGCAUGGGACUUUGGAGUCCCGCUGCUAAUGAUAGGUUGCUACUCACAAUUAACACCCCUGACCCUAAUAGUAGCCAUCAUUUUGCUCGUGGCGCACUACAUGUACUUGAUCCCAGGGCUGCAGGCAGCAGCUGCGCGUGCUGCCCAGAAGAGAACGGCAGCUGGCAUCAUGA
KX247646    CCAUUCUACGCAUGGGACUUUGGAGUCCCGCUGCUAAUGAUAGGUUGCUACUCACAAUUAACACCCCUGACCCUAAUAGUGGCCAUCAUUUUGCUCGUGGCGCACUACAUGUACUUGAUCCCAGGGCUGCAGGCAGCAGCUGCGCGUGCUGCCCAGAAGAGAACGGCAGCUGGCAUCAUGA
KX262887    CCAUUCUACGCAUGGGACUUUGGAGUCCCGCUGCUAAUGAUAGGUUGCUACUCACAAUUAACACCCCUGACCCUAAUAGUGGCCAUCAUUUUGCUCGUGGCGCACUACAUGUACUUGAUCCCAGGGCUGCAGGCAGCAGCUGCGCGUGCUGCCCAGAAGAGAACGGCAGCUGGCAUCAUGA
KX280026    CCAUUCUACGCAUGGGACUUUGGAGUCCCGCUGCUAAUGAUAGGUUGCUACUCACAAUUAACACCCCUGACCCUAAUAGUGGCCAUCAUUUUGCUCGUGGCGCACUACAUGUACUUGAUCCCAGGGCUGCAGGCAGCAGCUGCGCGUGCUGCCCAGAAGAGAACGGCAGCUGGCAUCAUGA
KU758877    CCAUUCUACGCAUGGGACUUUGGAGUCCCGCUGCUAAUGAUAGGUUGCUACUCACAAUUAACACCCCUGACCCUAAUAGUGGCCAUCAUUUUGCUCGUGGCGCACUACAUGUACUUGAUCCCAGGGCUGCAGGCAGCAGCUGCGCGUGCUGCCCAGAAGAGAACGGCAGCUGGCAUCAUGA
KU866423    CCAUUCUACGCAUGGGACUUUGGAGUCCCGCUGCUAAUGAUAGGUUGCUACUCACAAUUAACACCCCUGACCCUAAUAGUGGCCAUCAUUUUGCUCGUGGCGCACUACAUGUACUUGAUCCCAGGGCUGCAGGCAGCAGCUGCGCGUGCUGCCCAGAAGAGAACGGCAGCUGGCAUCAUGA
KX377336    CCAUUCUAUGCGUGGGACUUCGGAGUCCCGCUGCUAAUGAUGGGUUGCUACUCACAAUUAACACCCUUGACCUUAAUAGUGGCCAUCAUUCUGCUCGUGGCGCACUACAUGUACUUGAUCCCAGGUCUACAGGCAGCAGCGGCGCGCGCUGCCCAGAAGAGAACGGCAGCUGGCAUCAUGA
KX377337    CCAUUCUACGCAUGGGACUUUGGAGUCCCGCUGCUAAUGAUAGGUUGCUACUCACAAUUAACACCCCUGACCCUAAUAGUGGCCAUCAUUUUGCUCGUGGCGCACUACAUGUACUUGAUCCCAGGGCUGCAGGCAGCAGCUGCGCGUGCUGCCCAGAAGAGAACGGCAGCUGGCAUCAUGA
KX369547    CCAUUCUACGCAUGGGACUUUGGAGUCCCGCUGCUAAUGAUAGGUUGCUACUCACAAUUAACACCCCUGACCCUAAUAGUGGCCAUCAUUUUGCUCGUGGCGCACUACAUGUACUUGAUCCCAGGGCUGCAGGCAGCAGCUGCGCGUGCUGCCCAGAAGAGAACGGCAGCUGGCAUCAUGA
KX548902    CCAUUCUACGCAUGGGACUUUGGAGUCCCGCUGCUAAUGAUAGGUUGCUACUCACAAUUAACACCCCUGACCCUAAUAGUGGCCAUCAUUUUGCUCGUGGCGCACUACAUGUACUUGAUCCCAGGGCUGCAGGCAGCAGCUGCGCGUGCUGCCCAGAAGAGAACGGCAGCUGGCAUCAUGA
KX601166    CCAUUUUAUGCAUGGGACUUUGGAGUCCCGCUGCUAAUGAUGGGUUGCUACUCACAAUUAACACCCCUGACCCUGAUAGUGGCCAUCAUUCUGCUUGUGGCACACUACAUGUACUUGAUCCCAGGUUUGCAGGCAGCAGCAGCACGUGCUGCCCAGAAGAGGACAGCAGCUGGCAUCAUGA
KX601167    CCAUUCUAUGCGUGGGACUUCGGAGUCCCGCUGCUAAUGAUGGGUUGCUACUCACAAUUAACACCCUUGACCUUAAUAGUGGCCAUCAUUCUGCUCGUGGCGCACUACAUGUACUUGAUCCCAGGUCUACAGGCAGCAGCGGCGCGCGCUGCCCAGAAGAGAACGGCAGCUGGCAUCAUGA
KX601168    CCAUUCUACGCAUGGGACUUUGGAGUCCCGCUGCUAAUGAUAGGUUGCUACUCACAAUUAACACCCCUGACCCUAAUAGUGGCCAUCAUUUUGCUCGUGGCGCACUACAUGUACUUGAUCCCAGGGCUGCAGGCAGCAGCUGCGCGUGCUGCCCAGAAGAGAACGGCAGCUGGCAUCAUGA
KX766028    CCAUUCUACGCAUGGGACUUUGGAGUCCCGCUGCUAAUGAUAGGUUGCUACUCACAAUUAACACCCCUGACCCUAAUAGUGGCCAUCAUUUUGCUCGUGGCGCACUACAUGUACUUGAUCCCAGGGCUGCAGGCAGCAGCUGCGCGUGCUGCCCAGAAGAGAACGGCAGCUGGCAUCAUGA
KX766029    CCAUUCUACGCAUGGGACUUUGGAGUCCCGCUACUAAUGAUAGGUUGCUACUCACAAUUAACACCCCUGACCCUAAUAGUGGCCAUCAUUUUGCUCGUGGCGCACUACAUGUACUUGAUCCCAGGGCUGCAGGCAGCAGCCGCGCGUGCUGCCCAGAAGAGAACGGCAGCUGGCAUCAUGA
KU940224    CCAUUCUACGCAUGGGACUUUGGAGUCCCGCUGCUAAUGAUAGGUUGCUACUCACAAUUAACACCCCUGACCCUAAUAGUGGCCAUCAUUUUGCUCGUGGCGCACUACAUGUACUUGAUCCCAGGGCUGCAGGCAGCAGCUGCGCGUGCUGCCCAGAAGAGAACGGCAGCUGGCAUCAUGA


            7777777777777777777777777777777777777777777777777777777777777777777777777777777777777777777777777777777777777777777777777777777777777777777777777777777777777777777777777777777
            2222222222222222222222222222222222222222222222222222222222233333333333333333333333333333333333333333333333333333333333333333333333333333333333333333333333333334444444444444444444444
            4444444445555555555666666666677777777778888888888999999999900000000001111111111222222222233333333334444444444555555555566666666667777777777888888888899999999990000000000111111111122
Sequence    1234567890123456789012345678901234567890123456789012345678901234567890123456789012345678901234567890123456789012345678901234567890123456789012345678901234567890123456789012345678901
-------------------------------------------------------------------------------------------------------------------------------------------------------------------------------------------------------
NC_012532   AGAAUCCCGUUGUGUGGAUGGAAUAGUGGUAACUGACAUUGACACAAUGACAAUAGACCCCCAGGUGGAGAAGAAGAUGGGACAAGUGUUACUCAUAGCAGUAGCCAUCUCCAGUGCUGUGCUGCUGCGGACCGCCUGGGGAUGGGGGAGGCUGGAGCUCUGAUCACAGCAGCGACCUCCAC
KU922960    AGAACCCUGUUGUGUGGAUGGAAUAGUGGUGACUGACAUUGACACAAUGACAAUUGACCCCCAAGUGGAGAAAAAGAUGGGACAGGUGCUACUCAUAGCAGUAGCCGUCUCCAGCGCCAUACUGUCGCGGACCGCCUGGGGGUGGGGGAGGCUGGGGCCCUGAUCACAGCCGCAACUUCCAC
KU922923    AGAACCCUGUUGUGUGGAUGGAAUAGUGGUGACUGACAUUGACACAAUGACAAUUGACCCCCAAGUGGAGAAAAAGAUGGGACAGGUGCUACUCAUAGCAGUAGCCGUCUCCAGCGCCAUACUGUCGCGGACCGCCUGGGGGUGGGGGAGGCUGGGGCCCUGAUCACAGCCGCAACUUCCAC
KU820898    AGAACCCUGUUGUGUGGAUGGAAUAGUGGUGACUGACAUUGACACAAUGACAAUUGACCCCCAAGUGGAGAAAAAGAUGGGACAGGUGCUACUCAUAGCAGUAGCCGUCUCCAGCGCCAUACUGUCGCGGACCGCCUGGGGGUGGGGGAGGCUGGGGCCCUGAUCACAGCCGCAACUUCCAC
KU321639    AGAACCCUGUUGUGUGGAUGGAAUAGUGGUGACUGACAUUGACACAAUGACAAUUGACCCCCAAGUGGAGAAAAAGAUGGGACAGGUGCUACUCAUGGCAGUAGCCGUCUCCAGCGCCAUACUGUCGCGGACCGCCUGGGGGUGGGGGAGGCUGGGGCCCUGAUCACAGCCGCAACUUCCAC
KU820899    AGAACCCUGUUGUGUGGAUGGAAUAGUGGUGACUGACAUUGACACAAUGACAAUUGACCCCCAAGUGGAGAAAAAGAUGGGACAGGUGCUACUCAUAGCAGUAGCCGUCUCCAGCGCCAUACUGUCGCGGACCGCCUGGGGGUGGGGGAGGCUGGGGCCCUGAUCACAGCUGCAACUUCCAC
KU853012    AGAACCCUGUUGUGUGGAUGGAAUAGUGGUGACUGACAUUGACACAAUGACAAUUGACCCCCAAGUGGAGAAAAAGAUGGGACAGGUGCUACUCAUAGCAGUAGCCGUCUCCAGCGCCAUACUGUCGCGGACCGCCUGGGGGUGGGGGAGGCUGGGGCCCUGAUCACAGCCGCAACUUCCAC
KU729217    AGAACCCUGUUGUGUGGAUGGAAUAGUGGUGACUGACAUUGACACAAUGACAAUUGACCCCCAAGUGGAGAAAAAGAUGGGACAGGUGCUACUCAUAGCAGUAGCCGUCUCCAGCGCCAUACUGUCGCGGACCGCCUGGGGGUGGGGGAGGCUGGGGCCCUGAUUACAGCCGCAACUUCCAC
KU729218    AGAACCCUGUUGUGUGGAUGGAAUAGUGGUGACUGACAUUGACACAAUGACAAUUGACCCCCAAGUGGAGAAAAAGAUGGGACAGGUGCUACUCAUAGCAGUAGCCGUCUCCAGCGCCAUACUGUCGCGGACCGCCUGGGGGUGGGGGAGGCUGGGGCCCUGAUCACAGCCGCAACUUCCAC
KU761564    AGAACCCUGUUGUGUGGAUGGAAUAGUGGUGACUGACAUUGACACAAUGACAAUUGACCCCCAAGUGGAGAAAAAGAUGGGACAGGUGCUACUCAUAGCAGUAGCCGUCUCCAGCGCCAUACUGUCGCGGACCGCCUGGGGGUGGGGGAGGCUGGGGCCCUGAUCACAGCCGCAACUUCCAC
KU740184    AGAACCCUGUUGUGUGGAUGGAAUAGUGGUGACUGACAUUGACACAAUGACAAUUGACCCCCAAGUGGAGAAAAAGAUGGGACAGGUGCUACUCAUAGCAGUAGCCGUCUCCAGCGCCAUACUGUCGCGGACCGCCUGGGGGUGGGGGAGGCUGGGGCCCUGAUCACAGCCGCAACUUCCAC
KU744693    AGAACCCUGUUGUGUGGAGGGAAUAGUGGUGACUGACAUUGACACAAUGACAAUUGACCCCCAAGUGGAGAAAAAGAUGGGACAGGUGCUACUCAUGGCAGUAGCCGUCUCCAGCGCCAUACUGUCGAGGACCGCCUGGGGGUGGGGGAGGCUGGGGCCCUGAUCACAGCCGCAACUUCCAC
KU497555    AGAACCCUGUUGUGUGGAUGGAAUAGUGGUGACUGACAUUGACACAAUGACAAUUGACCCCCAAGUGGAGAAAAAGAUGGGACAGGUGCUACUCAUAGCAGUAGCCGUCUCCAGCGCCAUACUGUCGCGGACCGCCUGGGGGUGGGGGAGGCUGGGGCCCUGAUCACAGCCGCAACUUCCAC
KU527068    AGAACCCUGUUGUGUGGAUGGAAUAGUGGUGACUGACAUUGACACAAUGACAAUUGACCCCCAAGUGGAGAAAAAGAUGGGACAGGUGCUACUCAUAGCAGUAGCAGUCUCCAGCGCCAUACUGUCGCGGACCGCCUGGGGGUGGGGGAGGCUGGGGCCCUGAUCACAGCCGCAACUUCCAC
KU501217    AGAACCCUGUUGUGUGGAUGGAAUAGUGGUGACUGACAUUGACACAAUGACAAUUGACCCCCAAGUGGAGAAAAAGAUGGGACAGGUGCUACUCAUAGCAGUAGCCGUCUCCAGCGCCAUACUGUCGCGGACCGCCUGGGGGUGGGGGAGGCUGGGGCCCUGAUCACAGCCGCAACUUCCAC
KU501216    AGAACCCUGUUGUGUGGAUGGAAUAGUGGUGACUGACAUUGACACAAUGACAAUUGACCCCCAAGUGGAGAAAAAGAUGGGACAGGUGCUACUCAUAGCAGUAGCCGUCUCCAGCGCCAUACUGUCGCGGACCGCCUGGGGGUGGGGGAGGCUGGGGCCCUGAUCACAGCCGCAACUUCCAC
KU501215    AGAACCCUGUUGUGUGGAUGGAAUAGUGGUGACUGACAUUGACACAAUGACAAUUGACCCCCAAGUGGAGAAAAAGAUGGGACAGGUGCUACUCAUAGCAGUAGCCGUCUCCAGCGCCAUACUGUCGCGGACCGCCUGGGGGUGGGGGAGGCUGGGGCUCUGAUCACAGCCGCAACUUCCAC
KU365780    AGAACCCUGUUGUGUGGAUGGAAUAGUGGUGACUGACAUUGACACAAUGACAAUUGACCCCCAAGUGGAGAAAAAGAUGGGACAGGUGCUACUCAUAGCAGUAGCCGUCUCCAGCGCCAUACUGUCGCGGACCGCCUGGGGGUGGGGGAGGCUGGGGCCCUGAUCACAGCCGCAACUUCCAC
KU365779    AGAACCCUGUUGUGUGGAUGGAAUAGUGGUGACUGACAUUGACACAAUGACAAUUGACCCCCAAGUGGAGAAAAAGAUGGGACAGGUGCUACUCAUAGCAGUAGCCGUCUCCAGCGCCAUACUGUCGCGGACCGCCUGGGGGUGGGGGAGGCUGGGGCCCUGAUCACAGCCGCAACUUCCAC
KU707826    AGAACCCUGUUGUGUGGAUGGAAUAGUGGUGACUGACAUUGACACAAUGACAAUUGACCCCCAAGUGGAGAAAAAGAUGGGACAGGUGCUACUCAUAGCAGUAGCCGUCUCCAGCGCCAUACUGUCGCGGACCGCCUGGGGGUGGGGGAGGCUGGGGCCCUGAUCACAGCCGCAACUUCCAC
KU365778    AGAACCCUGUUGUGUGGAUGGAAUAGUGGUGACUGACAUUGACACAAUGACAAUUGACCCCCAAGUGGAGAAAAAGAUGGGACAGGUGCUACUCAUAGCAGUAGCCGUCUCCAGCGCCAUACUGUCGCGGACCGCCUGGGGGUGGGGGAGGCUGGGGCCCUGAUCACAGCCGCAACUUCCAC
KU365777    AGAACCCUGUUGUGUGGAUGGAAUAGUGGUGACUGACAUUGACACAAUGACAAUUGACCCCCAAGUGGAGAAAAAGAUGGGACAGGUGCUACUCAUAGCAGUAGCCGUCUCCAGCGCCAUACUGUCGCGGACCGCCUGGGGGUGGGGGAGGCUGGGGCCCUGAUCACAGCCGCAACUUCCAC
KU312312    AGAACCCUGUUGUGUGGAUGGAAUAGUGGUGACUGACAUUGACACAAUGACAAUUGACCCCCAAGUGGAGAAAAAGAUGGGACAGGUGCUACUCAUAGCAGUAGCCGUCUCCAGCGCCAUACUGUCGCGGACCGCCUGGGGGUGGGGGAGGCUGGGGCCCUGAUCACAGCCGCAACUUCCAC
KF268950    AGAAUCCCGUUGUGUGGAUGGAAUAGUGGUAACUGACAUUGACACAAUGACAAUUGACCCCCAAGUGGAGAAGAAGAUGGGACAAGUGUUACUUAUAGCAGUAGCCAUCUCCAGUGCUGUGCUGCUGCGGACUGCUUGGGGAUGGGGGAGGCUGGAGCUCUGAUUACUGCAGCAACCUCCAC
KF268949    AGAAUCCCGUUGUGUGGAUGGAAUAGUGGUAACUGACAUUGACACAAUGACAAUUGACCCCCAAGUGGAGAAGAAGAUGGGACAAGUGUUACUCAUAGCAGUAGCCAUCUCCAGUGCUGUGCUGCUGCGAACUGCUUGGGGAUGGGGGAGGCUGGAGCUCUGAUCACAGCAGCAACCUCCAC
KF268948    AGAAUCCCGUUGUGUGGAUGGAAUAGUGGUAACUGACAUUGACACAAUGACAAUUGACCCCCAAGUGGAGAAGAAGAUGGGACAAGUGUUACUUAUAGCAGUAGCCAUCUCCAGUGCUGUGCUGCUGCGGACUGCUUGGGGAUGGGGGAGGCUGGAGCUCUGAUUACUGCAGCAACCUCCAC
KJ776791    AGAACCCUGUUGUGUGGAUGGAAUAGUGGUGACUGACAUUGACACAAUGACAAUUGACCCCCAAGUGGAGAAAAAGAUGGGACAGGUGCUACUCAUAGCAGUAGCCGUCUCCAGCGCCAUACUGUCGCGGACCGCCUGGGGGUGGGGGAGGCUGGGGCCCUGAUCACAGCGGCAACUUCCAC
KF383119    AGAAUCCCGUUGUGUGGAUGGAAUAGUGGUAACUGACAUUGACACAAUGACAAUAGACCCCCAGGUGGAGAAGAAGAUGGGACAAGUGUUACUCAUAGCAGUAGCCAUCUCCAGUGCUGUGCUGCUGCGGACCGCCUGGGGAUGGGGGAGGCUGGAGCUCUGAUCACAGCAGCGACCUCCAC
KF383118    AGAAUCCCGUUGUGUGGAUGGAAUAGUGGUAACUGACAUUGACACAAUGACAAUAGACCCCCAGGUGGAGAAGAAGAUGGGACAAGUGUUACUCAUAGCAGUAGCCAUCUCCAGUGCUGUGCUGCUGCGGACCGCCUGGGGAUGGGGGAGGCUGGAGCUCUGAUCACAGCAGCGACCUCCAC
KF383117    AAAAUCCCGUUGUGUGGAUGGAAUAGUGGUGACUGACAUUGACACAAUGACAAUUGACCCCCAAGUGGAGAAGAGAUGGGACAAGUGUUACUCAUAGCAGUAGCUGUCUCCAGUGCUGUGCUGCUGCGGACCGCCUGGGGGUGGGGGAGGCUGGAGCUCUGAUCACAGCAGCUACCUCCAC
KF383116    AGAAUCCCGUUGUGUGGAUGGAAUAGUGGUGACUGACAUUGACACAAUGACAAUUGACCCCCAAGUGGAGAAGAAGAUGGGACAAGUGUUACUCAUAGCAGUAGCUGUCUCCAGUGCUGUGCUGCUGCGGACCGCUUGGGGAUGGGGGAGGCUGGGGCUCUGAUCACAGCAGCAACCUCCAC
KF383115    AGAAUCCCGUUGUGUGGAUGGAAUAGUGGUAACUGACAUUGACACAAUGACAAUUGACCCCCAAGUGGAGAAGAAGAUGGGACAAGUGUUACUCAUAGCAGUAGCCAUCUCCAGUGCUGUGCUGCUGCGGACUGCUUGGGGAUGGGGGAAGCUGGAGCUCUGAUCACAGCAGCAACCUCCAC
EU545988    AGAACCCUGUUGUGUGGAUGGAAUAGUGGUGACUGACAUUGACACAAUGACAAUUGACCACGAGUGGAGAAAAAGAUGGGACAGGUGCUACUCAUAGCAGUAGCCGUCUCCAGCGCCAUACUGUCGCGGACCGCCUGGGGGUGGGGGAGGCUGGGGCCCUGAUCACAGCUGCAACUUCCAC
KU955589    AGAACCCUGUUGUGUGGAUGGAAUAGUGGUGACUGACAUUGACACAAUGACAAUUGACCCCCAAGUGGAGAAAAAGAUGGGACAGGUGCUACUCAUAGCAGUAGCCGUCUCCAGCGCCAUACUGUCGCGGACCGCCUGGGGGUGGGGGAGGCUGGGGCCCUGAUCACAGCUGCAACUUCCAC
KU955590    AGAACCCUGUUGUGUGGAUGGAAUAGUGGUGACUGACAUUGACACAAUGACAAUUGACCCCCAAGUGGAGAAAAAGAUGGGACAGGUGCUACUCAUAGCAGUAGCCGUCUCCAGCGCCAUACUGUCGCGGACCGCCUGGGGGUGGGGGAGGCUGGGGCCCUGAUCACAGCCGCAACUUCCAC
KU940228    AGAACCCUGUUGUGUGGAUGGAAUAGUGGUGACUGACAUUGACACAAUGACAAUUGACCCCCAAGUGGAGAAAAAGAUGGGACAGGUGCUACUCAUAGCAGUAGCCGUCUCCAGCGCCAUACUGUCGCGGACCGCCUGGGGGUGGGGGAGGCUGGGGCUCUGAUCACAGCCGCAACUUCCAC
KU991811    AGAACCCUGUUGUGUGGAUGGAAUAGUGGUGACUGACAUUGACACAAUGACAAUUGACCCCCAAGUGGAGAAAAAGAUGGGACAGGUGCUACUCAUAGCAGUAGCCGUCUCCAGCGCCAUACUGUCGCGGACCGCCUGGGGGUGGGGGAGGCUGGGGCCCUGAUCACAGCCGCAACUUCCAC
KU509998    AGAACCCUGUUGUGUGGAUGGAAUAGUGGUGACUGACAUUGACACAAUGACAAUUGACCCCCAAGUGGAGAAAAAGAUGGGACAGGUGCUACUCAUGGCAGUAGCCGUCUCCAGCGCCAUACUGUCGCGGACCGCCUGGGGGUGGGGGAGGCUGGGGCCCUGAUCACAGCCGCAACUUCCAC
KX056898    AGAACCCUGUUGUGUGGAUGGAAUAGUGGUGACUGACAUUGACACAAUGACAAUUGACCCCCAAGUGGAGAAAAAGAUGGGACAGGUGCUACUCAUAGCAGUAGCCGUCUCCAGCGCCAUACUGUCGCGGACCGCCUGGGGGUGGGGGAGGCUGGGGCCCUGAUCACAGCCGCAACUUCCAC
KX117076    AGAACCCUGUUGUGUGGAUGGAAUAGUGGUGACUGACAUUGACACAAUGACAAUUGACCCCCAAGUGGAGAAAAAGAUGGGACAGGUGCUACUCAUAGCAGUAGCCGUCUCCAGCGCCAUACUGUCGCGGACCGCCUGGGGGUGGGGGAGGCUGGGGCCCUGAUCACAGCUGCAACUUCCAC
KX185891    AGAACCCUGUUGUGUGGAUGGAAUAGUGGUGACUGACAUUGACACAAUGACAAUUGACCCCCAAGUGGAGAAAAAGAUGGGACAGGUGCUACUCAUAGCAGUAGCCGUCUCCAGCGCCAUACUGUCGCGGACCGCCUGGGGGUGGGGGAGGCUGGGGCCCUGAUCACAGCUGCAACUUCCAC
KX247646    AGAACCCUGUUGUGUGGAUGGAAUAGUGGUGACUGACAUUGACACAAUGACAAUUGACCCCCAAGUGGAGAAAAAGAUGGGACAGGUGCUACUCAUAGCAGUAGCCGUCUCCAGCGCCAUACUGUCGCGGACCGCCUGGGGGUGGGGGAGGCUGGGGCCCUGAUCACAGCCGCAACUUCCAC
KX262887    AGAACCCUGUUGUGUGGAUGGAAUAGUGGUGACUGACAUUGACACAAUGACAAUUGACCCCCAAGUGGAGAAAAAGAUGGGACAGGUGCUACUCAUAGCAGUAGCCGUCUCCAGCGCCAUACUGUCGCGGACCGCCUGGGGGUGGGGGAGGCUGGGGCCCUGAUCACAGCCGCAACUUCCAC
KX280026    AGAACCCUGUUGUGUGGAUGGAAUAGUGGUGACUGACAUUGACACAAUGACAAUUGACCCCCAAGUGGAGAAAAAGAUGGGACAGGUGCUACUCAUAGCAGUAGCCGUCUCCAGCGCCAUACUGUCGCGGACCGCCUGGGGGUGGGGAGAGGCUGGGGCCCUGAUCACAGCCGCAACUUCCAC
```

```
KU758877    AGAACCCUGUUGUGGAUGGAAUAGUGGUGACUGACAUUGACACAAUGACAAUUGACCCCCAAGUGGAGAAAAAGAUGGGACAGGUGCUACUCAUAGCAGUAGCCGUCUCCAGCGCCAUACUGUCGCGGACCGCCUGGGGGUGGGGGGAGGCUGGGGCCCUGAUCACAGCCGCAACUUCCAC
KU866423    AGAACCCUGUUGUGGAUGGAAUAGUGGUGACUGACAUUGACACAAUGACAAUUGACCCCCAAGUGGAGAAAAAGAUGGGACAGGUGCUACUCAUAGCAGUAGCCGUCUCCAGCGCCAUACUGUCGCGGACCGCCUGGGGGUGGGGGGAGGCUGGGGCCCUGAUCACAGCUGCAACUUCCAC
KX377336    AGAACCCUGUUGUGGAUGGAAUAGUGGUGACUGACAUUGACACAAUGACAAUUGACCCCCAAGUGGAGAAAAAGAUGGGACAAGUGCUACUCAUAGCAGUAGCCAUCUCCAGUGCCGUUCUGCUGCGCACCGCCUGGGGGUGGGGGGAGGCUGGGGCCCUGAUCACAGCCGCAACUUCCAC
KX377337    AGAACCCUGUUGUGGAUGGAAUAGUGGUGACUGACAUUGACACAAUGACAAUUGACCCCCAAGUGGAGAAAAAGAUGGGACAGGUGCUACUCAUAGCAGUAGCCGUCUCCAGCGCCAUACUGUCGCGGACCGCCUGGGGGUGGGGGGAGGCUGGGGCUCUGAUCACAGCCGCAACUUCCAC
KX369547    AGAACCCUGUUGUGGAUGGAAUAGUGGUGACUGACAUUGACACAAUGACAAUUGACCCCCAAGUGGAGAAAAAGAUGGGACAGGUGCUACUCAUAGCAGUAGCCGUCUCCAGCGCCAUACUGUCGCGGACCGCCUGGGGGUGGGGGGAGGCUGGGGCCCUGAUCACAGCUGCAACUUCCAC
KX548902    AGAACCCUGUUGUGGAUGGAAUAGUGGUGACUGACAUUGACACAAUGACAAUUGACCCCCAAGUGGAGAAAAAGAUGGGACAGGUGCUACUCAUAGCAGUAGCCGUCUCCAGCGCCAUACUGUCGCGGACCGCCUGGGGGUGGGGGGAGGCUGGGGCCCUGAUCACAGCCGCAACUUCCAC
KX601166    AGAAUCCCGUUGUGGAUGGAAUAGUGGUGACUGACAUUGACACAAUGACAAUUGACCCCCAAGUGGAGAAGAAGAUGGGACAAGUGUUACUCAUAGCAGUAGCUGUCUCCAGUGCUGUGCUACUGCGGACCGCUUGGGGAUGGGGGGAGGCUGGGGCUCUGAUCACAGCAGCAACCUCCAC
KX601167    AGAACCCUGUUGUGGAUGGAAUAGUGGUGACUGACAUUGACACAAUGACAAUUGACCCCCAAGUGGAGAAAAAGAUGGGACAAGUGCUACUCAUAGCAGUAGCCAUCUCCAGUGCCGUUCUGCUGCGCACCGCCUGGGGGUGGGGGGAGGCUGGGGCCCUGAUCACAGCCGCAACUUCCAC
KX601168    AGAACCCUGUUGUGGAUGGAAUAGUGGUGACUGACAUUGACACAAUGACAAUUGACCCCCAAGUGGAGAAAAAGAUGGGACAGGUGCUACUCAUAGCAGUAGCCGUCUCCAGCGCCAUACUGUCGCGGACCGCCUGGGGGUGGGGGGAGGCUGGGGCUCUGAUCACAGCCGCAACUUCCAC
KX766028    AGAAUCCUGUUGUGGAUGGAAUAGUGGUGACUGACAUUGACACAAUGACAAUUGACCCCCAAGUGGAGAAAAAGAUGGGACAGGUGCUACUCAUAGCAGUAGCCGUCUCCAGCGCCAUACUGUCGCGGACCGCCUGGGGGUGGGGGGAGGCUGGGGCCCUGAUCACAGCCGCAACUUCCAC
KX766029    AGAACCCUGUUGUGGAUGGAAUAGUGGUGACUGACAUUGACACAAUGACAAUUGACCCCCAAGUGGAGAAAAAGAUGGGACAGGUGCUACUCAUAGCAGUAGCCGUCUCCAGCGCCAUACUGUCGCGGACCGCCUGGGGGUGGGGGGAGGCUGGGGCCCUGAUCACAGCCGCAACUUCCAC
KU940224    AGAACCCUGUUGUGGAUGGAAUAGUGGUGACUGACAUUGACACAAUGACAAUUGACCCCCAAGUGGAGAAAAAGAUGGGACAGGUGCUACUCAUAGCAGUAGCCGUCUCCAGCGCCAUACUGUCGCGGACCGCCUGGGGGUGGGGGGAGGCUGGGGCUCUGAUCACAGCCGCAACUUCCAC


            7777777777777777777777777777777777777777777777777777777777777777777777777777777777777777777777777777777777777777777777777777777777777777777777777777777777777777777777777777777777777
            4444444444444444444444444444444444444444444444444444444444444444444444444444445555555555555555555555555555555555555555555555555555555555555555555555555555555555555555555555555555666
            2222222233333333334444444444555555555566666666667777777777888888888899999999990000000000111111111122222222223333333333444444444455555555556666666666777777777788888888889999999999000
Sequence    2345678901234567890123456789012345678901234567890123456789012345678901234567890123456789012345678901234567890123456789012345678901234567890123456789012345678901234567890123456789012
---------------------------------------------------------------------------------------------------------------------------------------------------------------------------------------------------------
NC_012532   CUUGUGGGAAGGCUCUCCAAACAAAUACUGGAACUCCUCUACAGCCACCUCACUGUGCAACAUCUUCAGAGGAAGCUAUCUGGCAGGAGCUUCCCUUAUCUAUACAGUGACGAGAAACGCUGGCCUGGUUAAGAGACGUGGAGGUGGGACGGGAGAGACUCUGGGAGAGAAGUGGAAAGCU
KU922960    UUUGUGGGAAGGCUCUCCGAACAAGUACUGGAACUCCUCUACAGCCACUUCACUGUGUAACAUUUUUAGGGGAAGUUACUUGGCUGGAGCUUCUCUAAUCUACACAGUAACAAGAAACGCUGGCUUGGUCAAGAGACGUGGGGGUGGAACAGGAGAGACCCUGGGAGAGAAAUGGAAGGCC
KU922923    UUUGUGGGAAGGCUCUCCGAACAAGUACUGGAACUCCUCUACAGCCACUUCACUGUGUAACAUUUUUAGGGGAAGUUACUUGGCUGGAGCUUCUCUAAUCUACACAGUAACAAGAAACGCUGGCUUGGUCAAGAGACGUGGGGGUGGAACAGGAGAGACCCUGGGAGAGAAAUGGAAGGCC
KU820898    UUUGUGGGAAGGCUCUCCGAACAAGUACUGGAACUCCUCUACAGCCACUUCACUGUGUAACAUUUUUAGGGGAAGUUACUUGGCUGGAGCUUCUCUAAUCUACACAGUAACAAGAAACGCUGGCUUGGUCAAGAGACGUGGGGGUGGAACAGGAGAGACCCUGGGAGAGAAAUGGAAGGCC
KU321639    UUUGUGGGAAGGCUCUCCGAACAAGUACUGGAACUCCUCUACAGCCACUUCACUGUGUAACAUUUUUAGGGGAAGUUACUUGGCUGGAGCUUCUCUAAUCUACACAGUAACAAGAAACGCUGGCUUGGUCAAGAGACGUGGGGGUGGAACAGGAGAGACCCUGGGAGAGAAAUGGAAGGCC
KU820899    UUUGUGGGAAGGCUCUCCGAACAAGUACUGGAACUCCUCUACAGCCACUUCACUGUGUAACAUUUUUAGGGGAAGUUACUUGGCUGGAGCUUCUCUAAUCUACACAGUAACAAGAAACGCUGGCUUGGUCAAGAGACGUGGGGGUGGAACAGGAGAGACCCUGGGAGAGAAAUGGAAGGCC
KU853012    UUUGUGGGAAGGCUCUCCGAACAAGUACUGGAACUCCUCUACAGCCACUUCACUGUGUAACAUUUUUAGGGGAAGUUACUUGGCUGGAGCUUCUCUAAUCUACACAGUAACAAGAAACGCUGGCUUGGUCAAGAGACGUGGGGGUGGAACAGGAGAGACCCUGGGAGAGAAAUGGAAGGCC
KU729217    UUUGUGGGAAGGCUCUCCGAACAAGUACUGGAACUCCUCUACAGCCACUUCACUGUGUAACAUUUUUAGGGGAAGUUACUUGGCUGGAGCUUCUCUAAUCUACACAGUAACAAGAAACGCUGGCUUGGUCAAGAGACGUGGGGGUGGAACAGGAGAGACCCUGGGAGAGAAAUGGAAGGCC
KU729218    UUUGUGGGAAGGCUCUCCGAACAAGUACUGGAACUCCUCUACAGCCACUUCACUGUGUAACAUUUUUAGGGGAAGUUACUUGGCUGGAGCUUCUCUGAUCUACACAGUAACAAGAAACGCUGGCUUGGUCAAGAGACGUGGGGGUGGAACAGGAGAGACCCUGGGAGAGAAAUGGAAGGCC
KU761564    UUUGUGGGAAGGCUCUCCGAACAAGUACUGGAACUCCUCUACAGCCACUUCACUGUGUAACAUUUUUAGGGGAAGUUACUUGGCUGGAGCUUCUCUAAUCUACACAGUAACAAGAAACGCUGGCUUGGUCAAGAGACGUGGGGGUGGAACAGGAGAGACCCUGGGAGAGAAAUGGAAGGCC
KU740184    UUUGUGGGAAGGCUCUCCGAACAAGUACUGGAACUCCUCUACAGCCACUUCACUGUGUAACAUUUUUAGGGGAAGUUACUUGGCUGGAGCUUCUCUAAUCUACACAGUAACAAGAAACGCUGGCUUGGUCAAGAGACGUGGGGGUGGAACAGGAGAGACCCUGGGAGAGAAAUGGAAGGCC
KU744693    UUUGUGGGAAGGCUCUCCGAACAAGUACUGGAACUCCUCUACAGCCACCUCACUGUGUAACAUUUUUAGGGGAAGUUACUUGGCUGGAGCUUCUCUAAUCUACACAGUAACAAGAAACGCUGGCUUGGUCAAGAGACGUGGGGGUGGAACAGGAGAGACCCUGGGAGAGAAAUGGAAGGCC
KU497555    UUUGUGGGAAGGCUCUCCGAACAAGUACUGGAACUCCUCUACAGCCACUUCACUGUGUAACAUUUUUAGGGGAAGUUACUUGGCUGGAGCUUCUCUAAUCUACACAGUAACAAGAAACGCUGGCUUGGUCAAGAGACGUGGGGGUGGAACAGGAGAGACCCUGGGAGAGAAAUGGAAGGCC
KU527068    UUUGUGGGAAGGCUCUCCGAACAAGUACUGGAACUCCUCUACAGCCACUUCACUGUGUAACAUUUUUAGGGGAAGUUACUUGGCUGGAGCUUCUCUAAUCUACAUAGUAACAAGAAACGCUGGCUUGGUCAAGAGACGUGGGGGUGGAACAGGAGAGACCCUGGGAGAGAAAUGGAAGGCC
KU501217    UUUGUGGGAAGGCUCUCCGAACAAGUACUGGAACUCCUCUACAGCCACUUCACUGUGUAACAUUUUUAGGGGAAGUUACUUGGCUGGAGCUUCUCUAAUCUACACAGUAACAAGAAACGCUGGCUUGGUCAAGAGACGUGGGGGUGGAACAGGAGAGACCCUGGGAGAGAAAUGGAAGGCC
KU501216    UUUGUGGGAAGGCUCUCCGAACAAGUACUGGAACUCCUCUACAGCCACUUCACUGUGUAACAUUUUUAGGGGAAGUUAUUUGGCUGGAGCUUCUCUAAUCUACACAGUAACAAGAAACGCUGGCUUGGUCAAGAGACGUGGGGGUGGAACAGGAGAGACCCUGGGAGAGAAAUGGAAGGCC
KU501215    UUUGUGGGAAGGCUCUCCGAACAAGUACUGGAACUCCUCUACAGCCACUUCACUGUGUAACAUUUUUAGGGGAAGUUACUUGGCUGGAGCUUCUCUAAUCUACACAGUAACAAGAAACGCUGGCUUGGUCAAGAGACGUGGGGGUGGAACAGGAGAGACCCUGGGAGAGAAAUGGAAGGCC
KU365780    UUUGUGGGAAGGCUCUCCGAACAAGUACUGGAACUCCUCUACAGCCACUUCACUGUGUAACAUUUUUAGGGGAAGUUACUUGGCUGGAGCUUCUCUAAUCUACACAGUAACAAGAAACGCUGGCUUGGUCAAGAGACGUGGGGGUGGAACAGGAGAGACCCUGGGAGAGAAAUGGAAGGCC
KU365779    UUUGUGGGAAGGCUCUCCGAACAAGUACUGGAACUCCUCUACAGCCACUUCACUGUGUAACAUUUUUAGGGGAAGUUACUUGGCUGGAGCUUCUCUAAUCUACACAGUAACAAGAAACGCUGGCUUGGUCAAGAGACGUGGGGGUGGAACAGGAGAGACCCUGGGAGAGAAAUGGAAGGCC
KU707826    UUUGUGGGAAGGCUCUCCGAACAAGUACUGGAACUCCUCUACAGCCACUUCACUGUGUAACAUUUUUAGGGGAAGUUACUUGGCUGGAGCUUCUCUAAUCUACACAGUAACAAGAAACGCUGGCUUGGUCAAGAGACGUGGGGGUGGAACAGGAGAGACCCUGGGAGAGAAAUGGAAGGCC
KU365778    UUUGUGGGAAGGCUCUCCGAACAAGUACUGGAACUCCUCUACAGCCACUUCACUGUGUAACAUUUUUAGGGGAAGUUACUUGGCUGGAGCUUCUCUAAUCUACACAGUAACAAGAAACGCUGGCUUGGUCAAGAGACGUGGGGGUGGAACAGGAGAGACCCUGGGAGAGAAAUGGAAGGCC
KU365777    UUUGUGGGAAGGCUCUCCGAACAAGUACUGGAACUCCUCUACAGCCACUUCACUGUGUAACAUUUUUAGGGGAAGUUACUUGGCUGGAGCUUCUCUAAUCUACACAGUAACAAGAAACGCUGGCUUGGUCAAGAGACGUGGGGGUGGAACAGGAGAGACCCUGGGAGAGAAAUGGAAGGCC
KU312312    UUUGUGGGAAGGCUCUCCGAACAAGUACUGGAACUCCUCUACAGCCACUUCACUGUGUAACAUUUUUAGGGGAAGUUACUUGGCUGGAGCUUCUCUAAUCUACACAGUAACAAGAAACGCUGGCUUGGUCAAGAGACGUGGGGGUGGAACAGGAGAGACCCUGGGAGAGAAAUGGAAGGCC
KF268950    CUUGUGGGAAGGUUCUCCAAACAAAUACUGGAACUCCUCUACAGCCACCUCACUGUGCAACAUCUUCAGAGGAAGUUACCUGGCAGGCGCUUCUCUUAUUUAUACAGUGACAAGAAAUGCUGGCCUAGUCAAGAGACGUGGAGGUGGAACGGGAGAGACUCUAGGAGAGAAGUGGAAAGCU
KF268949    CUUGUGGGAAGGUUCUCCAAACAAAUACUGGAACUCCUCUACAGCCACCUCACUGUGCAACAUCUUUAGAGGAAGUUACCUGGCAGGCGCUUCUCUCAUUUAUACAGUGACAAGAAAUGCUGGCCUAGUCAAGAGACGUGGAGGUGGAACGGGAGAGACUCUAGGAGAGAAGUGGAAAGCU
KF268948    CUUGUGGGAAGGUUCUCCAAACAAAUACUGGAACUCCUCUACAGCCACCUCACUGUGCAACAUCUUCAGAGGAAGUUACCUGGCAGGCGCUUCUCUUAUUUAUACAGUGACAAGAAAUGCUGGCCUAGUCAAGAGACGUGGAGGUGGAACGGGAGAGACUCUAGGAGAGAAGUGGAAAGCU
KJ776791    UUUGUGGGAAGGCUCUCCGAACAAGUACUGGAACUCCUCUACAGCCACUUCACUGUGUAACAUUUUUAGGGGAAGUUACUUGGCUGGAGCUUCUCUAAUCUACACAGUAACAAGAAACGCUGGCUUGGUCAAGAGACGUGGGGGUGGAACAGGAGAGACCCUGGGAGAGAAAUGGAAGGCC
KF383119    CUUGUGGGAAGGCUCUCCAAACAAAUACUGGAACUCCUCUACAGCCACCUCACUGUGCAACAUCUUCAGAGGAAGCUAUCUGGCAGGAGCUUCCCUUAUCUAUACAGUGACGAGAAACGCUGGCCUGGUUAAGAGACGUGGAGGUGGGACGGGAGAGACUCUGGGAGAGAAGUGGAAAGCU
KF383118    CUUGUGGGAAGGCUCUCCAAACAAAUACUGGAACUCCUCUACAGCCACCUCACUGUGCAACAUCUUCAGAGGAAGCUAUCUGGCAGGAGCUUCCCUUAUCUAUACAGUGACGAGAAACGCUGGCCUGGUUAAGAGACGUGGAGGUGGGACGGGAGAGACUCUGGGAGAGAAGUGGAAAGCU
KF383117    CUUAUGGGAAGGCUCUCCAAACAAAUACUGGAACUCCUCUACAGCCACCUCACUAUGCAACAUCUUUAGAGGAAGUUAUUUGGCAGGGGCUUCCCUUAUUUACACAGUGACAAGAAAUGCCGGUCUGGUUAAGAGACGUGGAGGUGGAACGGGAGAGACCCUGGGAGAGAAGUGGAAAGCC
KF383116    CUUAUGGGAAGGCUCUCCAAACAAAUACUGGAACUCCUCCACAGCCACCUCACUGUGCAACAUCUUUAGAGGAAGUUAUUUGGCAGGGGCUUCCCUUAUUUACACAGUGACAAGAAAUGCCGGUCUGGUUAAGAGACGUGGAGGUGGAACGGGAGAGACCCUGGGAGAGAAGUGGAAAGCC
KF383115    UUUGUGGGAAGGUUCUCCAAACAAAUACUGGAACUCCUCUACAGCCACCUCACUGUGCAACAUCUUUAGAGGAAGUUACCUGGCAGGCGCUUCUCUUAUUUACACAGUGACAAGAAAUGCUGGCCUGGUCAAGAGACGUGGAGGUGGAACGGGAGAGACUCUAGGAGAGAAGUGGAAAGCU
EU545988    UUUGUGGGAAGGCUCUCCGAACAAGUACUGGAACUCCUCCACAGCCACUUCACUGUGUAACAUUUUUAGGGGAAGUUACUUGGCUGGAGCUUCUCUAAUCUACACAGUAACAAGAAACGCUGGCUUGGUCAAGAGACGUGGGGGUGGAACGGGAGAGACCCUGGGAGAGAAAUGGAAGGCC
KU955589    UUUGUGGGAAGGCUCUCCGAACAAGUACUGGAACUCCUCUACAGCCACUUCACUGUGUAACAUUUUUAGGGGAAGUUACUUGGCUGGAGCUUCUCUAAUCUACACAGUAACAAGAAACGCUGGCUUGGUCAAGAGACGUGGGGGUGGAACAGGAGAGACCCUGGGAGAGAAAUGGAAGGCC
KU955590    UUUGUGGGAAGGCUCUCCGAACAAGUACUGGAACUCCUCUACAGCCACUUCACUGUGUAACAUUUUUAGGGGAAGUUACUUGGCUGGAGCUUCUCUAAUCUACACAGUAACAAGAAACGCUGGCUUGGUCAAGAGACGUGGGGGUGGAACAGGAGAGACCCUGGGAGAGAAAUGGAAGGCC
KU940228    UUUGUGGGAAGGCUCUCCGAACAAGUACUGGAACUCCUCUACAGCCACUUCACUGUGUAACAUUUUUAGGGGAAGUUACUUGGCUGGAGCUUCUCUAAUCUACACAGUAACAAGAAACGCUGGCUUGGUCAAGAGACGUGGGGGUGGAACAGGAGAGACCCUGGGAGAGAAAUGGAAGGCC
KU991811    UUUGUGGGAAGGCUCUCCGAACAAGUACUGGAACUCCUCUACAGCCACUUCACUGUGUAACAUUUUUAGGGGAAGUUACUUGGCUGGAGCUUCUCUAAUCUACACAGUAACAAGAAACGCUGGCUUGGUCAAGAGACGUGGGGGUGGAACAGGAGAGACCCUGGGAGAGAAAUGGAAGGCC
KU509998    UUUGUGGGAAGGCUCUCCGAACAAGUACUGGAACUCCUCUACAGCCACUUCACUGUGUAACAUUUUUAGGGGAAGUUACUUGGCUGGAGCUUCUCUAAUCUACACAGUAACAAGAAACGCUGGCUUGGUCAAGAGACGUGGGGGUGGAACAGGAGAGACCCUGGGAGAGAAAUGGAAGGCC
KX056898    UUUGUGGGAAGGCUCUCCGAACAAGUACUGGAACUCCUCUACAGCCACUUCACUGUGUAACAUUUUUAGGGGAAGUUACUUGGCUGGAGCUUCUCUAAUCUACACAGUAACAAGAAACGCUGGCUUGGUCAAGAGACGUGGGGGUGGAACAGGAGAGACCCUGGGAGAGAAAUGGAAGGCC
KX117076    UUUGUGGGAAGGCUCUCCGAACAAGUACUGGAACUCCUCUACAGCCACUUCACUGUGUAACAUUUUUAGGGGAAGUUACUUGGCUGGAGCUUCUCUAAUCUACACAGUAACAAGAAACGCUGGCUUGGUCAAGAGACGUGGGGGUGGAACAGGAGAGACCCUGGGAGAGAAAUGGAAGGCC
KX185891    UUUGUGGGAAGGCUCUCCGAACAAGUACUGGAACUCCUCUACAGCCACUUCACUGUGUAACAUUUUUAGGGGAAGUUACUUGGCUGGAGCUUCUCUAAUCUACACAGUAACAAGAAACGCUGGCUUGGUCAAGAGACGUGGGGGUGGAACAGGAGAGACCCUGGGAGAGAAAUGGAAGGCC
KX247646    UUUGUGGGAAGGCUCUCCGAACAAGUACUGGAACUCCUCUACAGCCACUUCACUGUGUAACAUUUUUAGGGGAAGUUACUUGGCUGGAGCUUCUCUAAUCUACACAGUAACAAGAAACGCUGGCUUGGUCAAGAGACGUGGGGGUGGAACAGGAGAGACCCUGGGAGAGAAAUGGAAGGCC
KX262887    UUUGUGGGAAGGCUCUCCGAACAAGUACUGGAACUCCUCUACAGCCACUUCACUGUGUAACAUUUUUAGGGGAAGUUACUUGGCUGGAGCCUCUCUAAUCUACACAGUAACAAGAAACGCUGGCUUGGUCAAGAGACGUGGGGGUGGAACAGGAGAGACCCUGGGAGAGAAAUGGAAGGCC
KX280026    UUUGUGGGAAGGCUCUCCGAACAAGUACUGGAACUCCUCUACAGCCACUUCACUGUGUAACAUUUUUAGGGGAAGUUACUUGGCUGGAGCUUCUCUAAUCUACACAGUAACAAGAAACGCUGGCUUGGUCAAGAGACGUGGGGGUGGAACAGGAGAGACCCUGGGAGAGAAAUGGAAGGCC
KU758877    UUUGUGGGAAGGCUCUCCGAACAAGUACUGGAACUCCUCUACAGCCACUUCACUGUGUAACAUUUUUAGGGGAAGUUACUUGGCUGGAGCUUCUCUAAUCUACACAGUAACAAGAAACGCUGGCUUGGUCAAGAGACGUGGGGGUGGAACAGGAGAGACCCUGGGAGAGAAAUGGAAGGCC
KU866423    UUUGUGGGAAGGCUCUCCGAACAAGUACUGGAACUCCUCUACAGCCACUUCACUGUGUAACAUUUUUAGGGGAAGUUACUUGGCUGGAGCUUCUCUAAUCUACACAGUAACAAGAAACGCUGGCUUGGUCAAGAGACGUGGGGGUGGAACAGGAGAGACCCUGGGAGAGAAAUGGAAGGCC
KX377336    UUUGUGGGAAGGCUCUCCGAAUAAAUACUGGAACUCCUCCACAGCCACUUCACUGUGUAACAUUUUUAGGGGAAGUUACUUGGCUGGAGCUUCUCUUAUUUACACAGUAACAAGAAACGCUGGCCUGGUCAAGAGACGUGGAGGUGGAACGGGAGAGACCCUGGGGGAGAAAUGGAAGGCC
KX377337    UUUGUGGGAAGGCUCUCCGAACAAGUACUGGAACUCCUCUACAGCCACUUCACUGUGUAACAUUUUUAGGGGAAGUUACUUGGCUGGAGCUUCUCUAAUCUACACAGUAACAAGAAACGCUGGCUUGGUCAAGAGACGUGGGGGUGGAACAGGAGAGACCCUGGGAGAGAAAUGGAAGGCC
KX369547    UUUGUGGGAAGGCUCUCCGAACAAGUACUGGAACUCCUCUACAGCCACUUCACUGUGUAACAUUUUUAGGGGAAGUUACUUGGCUGGAGCUUCUCUAAUCUACACAGUAACAAGAAACGCUGGCUUGGUCAAGAGACGUGGGGGUGGAACAGGAGAGACCCUGGGAGAGAAAUGGAAGGCC
KX548902    UUUGUGGGAAGGCUCUCCGAACAAGUACUGGAACUCCUCUACAGCCACUUCACUGUGUAACAUUUUUAGGGGAAGUUACUUGGCUGGAGCUUCUCUAAUCUACACAGUAACAAGAAACGCUGGCUUGGUCAAGAGACGUGGGGGUGGAACAGGAGAGACCCUGGGAGAGAAAUGGAAGGCC
KX601166    CUUAUGGGAAGGCUCUCCAAACAAAUACUGGAACUCCUCCACAGCCACCUCACUGUGCAACAUCUUUAGAGGAAGUUAUUUGGCAGGGGCUUCCCUUAUUUACACAGUGACAAGAAAUGCCGGUCUGGUUAAGAGACGUGGAGGUGGAACGGGAGAGACCCUGGGAGAGAAGUGGAAAGCC
KX601167    UUUGUGGGAAGGCUCUCCGAAUAAAUACUGGAACUCCUCCACAGCCACUUCACUGUGUAACAUUUUUAGGGGAAGUUACUUGGCUGGAGCUUCUCUUAUUUACACAGUAACAAGAAACGCUGGCCUGGUCAAGAGACGUGGAGGUGGAACGGGAGAGACCCUGGGGGAGAAAUGGAAGGCC
KX601168    UUUGUGGGAAGGCUCUCCGAACAAGUACUGGAACUCCUCUACAGCCACUUCACUGUGUAACAUUUUUAGGGGAAGUUACUUGGCUGGAGCUUCUCUAAUCUACACAGUAACAAGAAACGCUGGCUUGGUCAAGAGACGUGGGGGUGGAACAGGAGAGACCCUGGGAGAGAAAUGGAAGGCC
KX766028    UUUGUGGGAAGGCUCUCCGAACAAGUACUGGAACUCCUCUACAGCCACUUCACUGUGUAACAUUUUUAGGGGAAGUUACUUGGCUGGAGCUUCUCUAAUCUACACAGUAACAAGAAACGCUGGCUUGGUCAAGAGACGUGGGGGUGGAACAGGAGAGACCCUGGGAGAGAAAUGGAAGGCC
KX766029    UUUGUGGGAAGGCUCUCCGAACAAGUACUGGAACUCCUCUACAGCCACUUCACUGUGUAACAUUUUUAGGGGAAGUUACUUGGCUGGAGCUUCUCUAAUCUACACAGUAACAAGAAACGCUGGCUUGGUCAAGAGACGUGGGGGUGGAACAGGAGAGACCCUGGGAGAGAAAUGGAAGGCC
KU940224    UUUGUGGGAAGGCUCUCCGAACAAGUACUGGAACUCCUCUACAGCCACUUCACUGUGUAACAUUUUUAGGGGAAGUUACUUGGCUGGAGCUUCUCUAAUCUACACAGUAACAAGAAACGCUGGCUUGGUCAAGAGACGUGGGGGUGGAACAGGAGAGACCCUGGGAGAGAAAUGGAAGGCC
```

```
               7777777777777777777777777777777777777777777777777777777777777777777777777777777777777777777777777777777777777777777777777777777777777777777777777777777777777777777777777
               6666666666666666666666666666666666666666666666666666666666666666666666666666666666666666666666666777777777777777777777777777777777777777777777777777777777777777777777777777
               0000000111111111122222222223333333333444444444455555555556666666666777777777788888888889999999999000000000011111111112222222222333333333344444444445555555555666666666677777777778888
Sequence       3456789012345678901234567890123456789012345678901234567890123456789012345678901234567890123456789012345678901234567890123456789012345678901234567890123456789012345678901234567890123
----------------------------------------------------------------------------------------------------------------------------------------------------------------------------------------------------
NC_012532      CGUCUGAAUCAGAUGUCGGCCCUGGAGUUCUACUCUUAUAAAAAGUCAGGUAUCACUGAAGUGUGUAGAGAGGAGGCUCGCCGUGCCCUCAAGGAUGGAGUGGCCACAGGAGGACAUGCCGUAUCCCGGGGAAGUGCAAAGAUCAGAUGGUUGGAGGAGAGAGGAUAUCUGCAGCCCUAUG
KU922960       CGCUUGAACCAGAUGUCGGCCCUGGAGUUCUACUCCUACAAAAAGUCAGGCAUCACCGAGGUGUGCAGAGAAGAGGCCCGCCGCGCCCUCAAGGACGGUGUGGCAACGGGAGGCCAUGCUGUGUCCCGAGGAAGUGCAAAGCUGAGAUGGUUGGUGGAGCGGGGAUACCUGCAGCCCUAUG
KU922923       CGCUUGAACCAGAUGUCGGCCCUGGAGUUCUACUCCUACAAAAAGUCAGGCAUCACCGAGGUGUGCAGAGAAGAGGCCCGCCGCGCCCUCAAGGACGGUGUGGCAACGGGAGGCCAUGCUGUGUCCCGAGGAAGUGCAAAGCUGAGAUGGUUGGUGGAGCGGGGAUACCUGCAGCCCUAUG
KU820898       CGCUUGAACCAGAUGUCGGCCCUGGAGUUCUACUCCUACAAAAAGUCAGGCAUCACCGAGGUGUGCAGAGAAGAGGCCCGCCGCGCCCUCAAGGACGGUGUGGCAACGGGAGGCCAUGCUGUGUCCCGAGGAAGUGCAAAGCUGAGAUGGUUGGUGGAGCGGGGAUACCUGCAGCCCUAUG
KU321639       CGCUUGAACCAGAUGUCGGCCCUGGAGUUCUACUCCUACAAAAAGUCAGGCAUCACCGAGGUGUGCAGAGAAGAGGCCCGCCGCGCCCUCAAGGACGGUGUGGCAACGGGAGGCCAUGCUGUGUCCCGAGGAAGUGCAAAGCUGAGAUGGUUGGUGGAGCGGGGAUACCUGCAGCCCUAUG
KU820899       CGCUUGAACCAGAUGUCGGCCCUGGAGUUCUACUCCUACAAAAAGUCAGGCAUCACCGAGGUGUGCAGAGAAGAGGCCCGCCGCGCCCUCAAGGACGGUGUGGCAACGGGAGGCCAUGCUGUGUCCCGAGGAAGUGCAAAGCUGAGAUGGUUGGUGGAGCGGGGAUACCUGCAGCCCUAUG
KU853012       CGCUUGAACCAGAUGUCGGCCCUGGAGUUCUACUCCUACAAAAAGUCAGGCAUCACCGAGGUGUGCAGAGAAGAGGCCCGCCGCGCCCUCAAGGACGGUGUGGCAACGGGAGGCCAUGCUGUGUCCCGAGGAAGUGCAAAGCUGAGAUGGUUGGUGGAGCGGGGAUACCUGCAGCCCUAUG
KU729217       CGCUUGAACCAGAUGUCGGCCCUGGAGUUCUACUCCUACAAAAAGUCAGGCAUCACCGAGGUGUGCAGAGAAGAGGCCCGCCGCGCCCUCAAGGACGGUGUGGCAACGGGAGGCCAUGCUGUGUCCCGAGGAAGUGCAAAGCUGAGAUGGUUGGUGGAGCGGGGAUACCUGCAGCCCUAUG
KU729218       CGCUUGAACCAGAUGUCGGCUCUGGAGUUCUACUCCUACAAAAAGUCAGGCAUCACCGAGGUGUGCAGAGAAGAGGCCCGCCGCGCCCUCAAGGACGGUGUGGCAACGGGAGGCCAUGCUGUGUCCCGAGGAAGUGCAAAGCUGAGAUGGUUGGUGGAGCGGGGAUACCUGCAGCCCUAUG
KU761564       CGCUUGAACCAGAUGUCGGCCCUGGAGUUCUACUCCUACAAAAAGUCAGGCAUCACCGAGGUGUGCAGAGAAGAGGCCCGCCGCGCCCUCAAGGACGGUGUGGCAACGGGAGGCCAUGCUGUGUCCCGAGGAAGUGCAAAGCUGAGAUGGUUGGUGGAGCGGGGAUACCUGCAGCCCUAUG
KU740184       CGCUUGAACCAGAUGUCGGCCCUGGAGUUCUACUCCUACAAAAAGUCAGGCAUCACCGAGGUGUGCAGAGAAGAGGCCCGCCGCGCCCUCAAGGACGGUGUGGCAACGGGAGGCCAUGCUGUGUCCCGAGGAAGUGCAAAGCUGAGAUGGUUGGUGGAGCGGGGAUACCUGCAGCCCUAUG
KU744693       CGCUUGAACCAGAUGUCGGCCCUGGAGUUCUACUCCUACAAAAAGUCAGGCAUCACCGAGGUGUGCAGAGAAGAGGCCCGCCGCGCCCUCAAGGACGGUGUGGCAACGGGAGGCCAUGCUGUGUCCCGAGGAAGUGCAAAGCUGAGAUGGUUGGUGGAGCGGGGAUACCUGCAGCCCUAUG
KU497555       CGCUUGAACCAGAUGUCGGCCCUGGAGUUCUACUCCUACAAAAAGUCAGGCAUCACCGAGGUGUGCAGAGAAGAGGCCCGCCGCGCCCUCAAGGACGGUGUGGCAACGGGAGGCCAUGCUGUGUCCCGAGGAAGUGCAAAGCUGAGAUGGUUGGUGGAGCGGGGAUACCUGCAGCCCUAUG
KU527068       CGCUUGAACCAGAUGUCGGCCCUGGAGUUCUACUCCUACAAAAAGUCAGGCAUCACCGAGGUGUGCAGAGAAGAGGCCCGCCGCGCCCUCAAGGAUGGUGUGGCAACGGGAGGCCAUGCUGUGUCCCGAGGAAGUGCAAAGCUGAGAUGGUUGGUGGAGCGGGGAUACCUGCAGCCCUAUG
KU501217       CGCUUGAACCAGAUGUCGGCCCUGGAGUUCUACUCCUACAAAAAGUCAGGCAUCACUGAGGUGUGCAGAGAAGAGGCCCGCCGCGCCCUCAAGGACGGUGUGGCAACGGGAGGCCAUGCUGUGUCCCGAGGAAGUGCAAAGCUGAGAUGGUUGGUGGAGCGGGGAUACCUGCAGCCCUAUG
KU501216       CGCUUGAACCAGAUGUCGGCCCUGGAGUUCUACUCCUACAAAAAGUCAGGCAUCACUGAGGUGUGCAGAGAAGAGGCCCGCCGCGCCCUCAAGGACGGUGUGGCAACGGGAGGCCAUGCUGUGUCCCGAGGAAGUGCAAAGCUGAGAUGGUUGGUGGAGCGGGGAUACCUGCAGCCCUAUG
KU501215       CGCUUGAACCAGAUGUCGGCCCUGGAGUUCUACUCCUACAAAAAGUCAGGCAUCACCGAGGUGUGCAGAGAAGAGGCCCGCCGCGCCCUCAAGGACGGUGUGGCAACGGGAGGCCAUGCUGUGUCCCGAGGAAGUGCAAAGCUGAGAUGGUUGGUGGAGCGGGGAUACCUGCAGCCCUAUG
KU365780       CGCUUGAACCAGAUGUCGGCCCUGGAGUUCUACUCCUACAAAAAGUCAGGCAUCACCGAGGUGUGCAGAGAAGAGGCCCGCCGCGCCCUCAAGGACGGUGUGGCAACGGGAGGCCAUGCUGUGUCCCGAGGAAGUGCAAAGCUGAGAUGGUUGGUGGAGCGGGGAUACCUGCAGCCCUAUG
KU365779       CGCUUGAACCAGAUGUCGGCCCUGGAGUUCUACUCCUACAAAAAGUCAGGCAUCACCGAGGUGUGCAGAGAAGAGGCCCGCCGCGCCCUCAAGGACGGUGUGGCAACGGGAGGCCAUGCUGUGUCCCGAGGAAGUGCAAAGCUGAGAUGGUUGGUGGAGCGGGGAUACCUGCAGCCCUAUG
KU707826       CGCUUGAACCAGAUGUCGGCCCUGGAGUUCUACUCCUACAAAAAGUCAGGCAUCACCGAGGUGUGCAGAGAAGAGGCCCGCCGCGCCCUCAAGGACGGUGUGGCAACGGGAGGCCAUGCUGUGUCCCGAGGAAGUGCAAAGCUGAGAUGGUUGGUGGAGCGGGGAUACCUGCAGCCCUAUG
KU365778       CGCUUGAACCAGAUGUCGGCCCUGGAGUUCUACUCCUACAAAAAGUCAGGCAUCACCGAGGUGUGCAGAGAAGAGGCCCGCCGCGCCCUCAAGGACGGUGUGGCAACGGGAGGCCAUGCUGUGUCCCGAGGAAGUGCAAAGCUGAGAUGGUUGGUGGAGCGGGGAUACCUGCAGCCCUAUG
KU365777       CGCUUGAACCAGAUGUCGGCCCUGGAGUUCUACUCCUACAAAAAGUCAGGCAUCACCGAGGUGUGCAGAGAAGAGGCCCGCCGCGCCCUCAAGGACGGUGUGGCAACGGGAGGCCAUGCUGUGUCCCGAGGAAGUGCAAAGCUGAGAUGGUUGGUGGAGCGGGGAUACCUGCAGCCCUAUG
KU312312       CGCUUGAACCAGAUGUCGGCCCUGGAGUUCUACUCCUACAAAAAGUCAGGCAUCACCGAGGUGUGCAGAGAAGAGGCCCGCCGCGCCCUCAAGGACGGUGUGGCAACGGGAGGCCAUGCUGUGUCCCGAGGAAGUGCAAAGCUGAGAUGGUUGGUGGAGCGGGGAUACCUGCAGCCCUAUG
KF268950       CGCCUGAAUCAGAUGUCGGCCCUGGAGUUCUACUCUUACAAAAAGUCAGGCAUCACUGAAGUGUGUAGAGAGGAGGCUCGCCGCGCCCUCAAGGAUGGAGUGGCCACAGGAGGACAUGCUGUAUCCCGGGGAAGCGCAAAGCUCAGAUGGCUGGUGGAGAGAGGAUACCUGCAACCCCAUG
KF268949       CGCCUGAAUCAGAUGUCGGCCCUGGAGUUCUACUCUUACAAAAAGUCAGGCAUCACUGAAGUGUGUAGAGAGGAGGCUCGCCGCGCCCUCAAGAAUGGAGUGGCCACAGGAGGACAUGCUGUAUCCCGGGGAAGCGCAAAGCUCAGAUGGCUGGUGGAGAGAGGAUACCUGCAACCCCAUG
KF268948       CGCCUGAAUCAGAUGUCGGCCCUGGAGUUCUACUCUUACAAAAAGUCAGGCAUCACUGAAGUGUGUAGAGAGGAGGCUCGCCGCGCCCUCAAGGAUGGAGUGGCCACAGGAGGACAUGCUGUAUCCCGGGGAAGCGCAAAGCUCAGAUGGCUGGUGGAGAGAGGAUACCUGCAACCCCAUG
KJ776791       CGCUUGAACCAGAUGUCGGCCCUGGAGUUCUACUCCUACAAAAAGUCAGGCAUCACCGAGGUGUGCAGAGAAGAGGCCCGCCGCGCCCUCAAGGACGGUGUGGCAACGGGAGGCCAUGCUGUGUCCCGAGGAAGUGCAAAGCUGAGAUGGUUGGUGGAGCGGGGAUACCUGCAGCCCUAUG
KF383119       CGUCUGAAUCAGAUGUCGGCCCUGGAGUUCUACUCUUAUAAAAAGUCAGGUAUCACUGAAGUGUGUAGAGAGGAGGCUCGCCGUGCCCUCAAGGAUGGAGUGGCCACAGGAGGACAUGCCGUAUCCCGGGGAAGUGCAAAGCUCAGAUGGUUGGUGGAGAGAGGAUAUCUGCAGCCCCAUG
KF383118       CGUCUGAAUCAGAUGUCGGCCCUGGAGUUCUACUCUUAUAAAAAGUCAGGUAUCACUGAAGUGUGUAGAGAGGAGGCUCGCCGUGCCCUCAAGGAUGGAGUGGCCACAGGAGGACAUGCCGUAUCCCGGGGAAGUGCAAAGCUCAGAUGGUUGGUGGAGAGAGGAUAUCUGCAGCCCCAUG
KF383117       CGCCUGAACCAGAUGUCGGCCCUGGAGUUUUACUCUUACAAAAAGUCAGGCAUCACCGAAGUGUGUAGGGAGGAGGCGCGCCGCGCUCUCAAGGAUGGAGUGGCCACAGGAGGGCAUGCUGUAUCCCGGGGAAGCGCAAAGCUUAGAUGGUUGGUAGAGAGAGGAUACCUGCAGCCCCAUG
KF383116       CGCCUGAACCAGAUGUCGGCCUUGGAGUUUUACUCUUACAAAAAGUCAGGCAUCACCGAAGUGUGUAGGGAGGAGGCGCGCCGCGCCCUCAAGGAUGGAGUGGCCACAGGAGGACAUGCUGUAUCCCGGGGAAGCGCAAAGCUUAGAUGGUUGGUAGAGAGAGGAUACCUGCAGCCCCAUG
KF383115       CGCCUGAAUCAGAUGUCGGCCCUGGAGUUCUACUCUUACAAAAAGUCGGGCAUCACUGAAGUGUGUAGAGAGGAGGCUCGCCGCGCCCUCAAGGAUGGAGUGGCCACAGGAGGACAUGCUGUAUCCCGGGGAAGCGCAAAGCUCAGAUGGCUGGUGGAGAGAGGAUACCUGCAGCCCCAUG
EU545988       CGCCUGAACCAGAUGUCGGCCCUAGAGUUCUACUCCUACAAAAAGUCAGGCAUCACCGAGGUGUGCAGAGAAGAGGCCCGCCGCGCCCUCAAGGACGGUGUGGCAACAGGAGGCCAUGCUGUGUCCCGAGGAAGUGCAAAGCUUAGAUGGUUGGUGGAGAGAGGAUACCUGCAGCCCUAUG
KU955589       CGCUUGAACCAGAUGUCGGCCCUGGAGUUCUACUCCUACAAAAAGUCAGGCAUCACCGAGGUGUGCAGAGAAGAGGCCCGCCGCGCCCUCAAGGACGGUGUGGCAACGGGAGGCCAUGCUGUGUCCCGAGGAAGUGCAAAGCUGAGAUGGUUGGUGGAGCGGGGAUACCUGCAGCCCUAUG
KU955590       CGCUUGAACCAGAUGUCGGCCCUGGAGUUCUACUCCUACAAAAAGUCAGGCAUCACCGAGGUGUGCAGAGAAGAGGCCCGCCGCGCCCUCAAGGACGGUGUGGCAACGGGAGGCCAUGCUGUGUCCCGAGGAAGUGCAAAGCUGAGAUGGUUGGUGGAUCGGGGAUACCUGCAGCCCUAUG
KU940228       CGCUUGAACCAGAUGUCGGCCCUGGAGUUCUACUCCUACAAAAAGUCAGGCAUCACCGAGGUGUGCAGAGAAGAGGCCCGCCGCGCCCUCAAGGACGGUGUGGCAACGGGAGGCCAUGCUGUGUCCCGAGGAAGUGCAAAGCUGAGAUGGUUGGUGGAGCGGGGAUACCUGCAGCCCCAUG
KU991811       CGCUUGAACCAGAUGUCGGCCCUGGAGUUCUACUCCUACAAAAAGUCAGGCAUCACCGAGGUGUGCAGAGAAGAGGCCCGCCGCGCCCUCAAGGACGGUGUGGCAACGGGAGGCCAUGCUGUGUCCCGAGGAAGUGCAAAGCUGAGAUGGUUGGUGGAGCGGGGAUACCUGCAGCCCUAUG
KU509998       CGCUUGAACCAGAUGUCGGCCCUGGAGUUCUACUCCUACAAAAAGUCAGGCAUCACCGAGGUGUGCAGAGAAGAGGCCCGCCGCGCCCUCAAGGACGGUGUGGCAACGGGAGGCCAUGCUGUGUCCCGAGGAAGUGCAAAGCUGAGAUGGUUGGUGGAGCGGGGAUACCUGCAGCCCUAUG
KX056898       CGCUUGAACCAGAUGUCGGCCCUGGAGUUCUACUCCUACAAAAAGUCAGGCAUCACCGAGGUGUGCAGAGAAGAGGCCCGCCGCGCCCUCAAGGACGGUGUGGCAACGGGAGGCCAUGCUGUGUCCCGAGGAAGUGCAAAGCUGAGAUGGUUGGUGGAUCGGGGAUACCUGCAGCCCUAUG
KX117076       CGCUUGAACCAGAUGUCGGCCCUGGAGUUCUACUCCUACAAAAAGUCAGGCAUCACCGAGGUGUGCAGAGAAGAGGCCCGCCGCGCCCUCAAGGACGGUGUGGCAACGGGAGGCCAUGCUGUGUCCCGAGGAAGUGCAAAGCUGAGAUGGUUGGUGGAGCGGGGAUACCUGCAGCCCUAUG
KX185891       CGCUUGAACCAGAUGUCGGCCCUGGAGUUCUACUCCUACAAAAAGUCAGGCAUCACCGAGGUGUGCAGAGAAGAGGCCCGCCGCGCCCUCAAGGACGGUGUGGCAACGGGAGGCCAUGCUGUGUCCCGAGGAAGUGCAAAGCUGAGAUGGUUGGUGGAGCGGGGAUACCUGCAGCCCUAUG
KX247646       CGCUUGAACCAGAUGUCGGCCCUGGAGUUCUACUCCUACAAAAAGUCAGGCAUCACCGAGGUGUGCAGAGAAGAGGCCCGCCGCGCCCUCAAGGACGGUGUGGCAACGGGAGGCCAUGCUGUGUCCCGAGGAAGUGCAAAGCUGAGAUGGUUGGUGGAGCGGGGAUACCUGCAGCCCUAUG
KX262887       CGCUUGAACCAGAUGUCGGCCCUGGAGUUCUACUCCUACAAAAAGUCAGGCAUCACCGAGGUGUGCAGAGAAGAGGCCCGCCGCGCCCUCAAGGACGGUGUGGCAACGGGAGGCCAUGCUGUGUCCCGAGGAAGUGCAAAGCUGAGAUGGUUGGUGGAGCGGGGAUACCUGCAGCCCUAUG
KX280026       CGCUUGAACCAGAUGUCGGCCCUGGAGUUCUACUCCUACAAAAAGUCAGGCAUCACCGAGGUGUGCAGAGAAGAGGCCCGCCGCGCCCUCAAGGACGGUGUGGCAACGGGAGGCCAUGCUGUGUCCCGAGGAAGUGCAAAGCUGAGAUGGUUGGUGGAGCGGGGAUACCUGCAGCCCUAUG
KU758877       CGCUUGAACCAGAUGUCGGCCCUGGAGUUCUACUCCUACAAAAAGUCAGGCAUCACCGAGGUGUGCAGAGAAGAGGCCCGCCGCGCCCUCAAGGACGGUGUGGCAACGGGAGGCCAUGCUGUGUCCCGAGGAAGUGCAAAGCUGAGAUGGUUGGUGGAGCGGGGAUACCUGCAGCCCUAUG
KU866423       CGCUUGAACCAGAUGUCGGCCCUGGAGUUCUACUCCUACAAAAAGUCAGGCAUCACCGAGGUGUGCAGAGAAGAGGCCCGCCGCGCCCUCAAGGACGGUGUGGCAACGGGAGGCCAUGCUGUGUCCCGAGGAAGUGCAAAGCUGAGAUGGUUGGUGGAGCGGGGAUACCUGCAGCCCUAUG
KX377336       CGCCUGAACCAGAUGUCGGCCCUGGAGUUUUACUCCUACAAAAAGUCAGGCAUCACCGAAGUGUGCAGAGAAGAAGCCCGCCGCGCCCUCAAGGACGGAGUGGCAACAGGAGGCCAUGCUGUGUCCCGAGGAAGCGCAAAGCUUAGAUGGUUGGUGGAGAGAGGAUACCUGCAGCCCUAUG
KX377337       CGCUUGAACCAGAUGUCGGCCCUGGAGUUCUACUCCUACAAAAAGUCAGGCAUCACCGAGGUGUGCAGAGAAGAGGCCCGCCGCGCCCUCAAGGACGGUGUGGCAACGGGAGGCCAUGCUGUGUCCCGAGGAAGUGCAAAGCUGAGAUGGUUGGUGGAGCGGGGAUACCUGCAGCCCUAUG
KX369547       CGCUUGAACCAGAUGUCGGCCCUGGAGUUCUACUCCUACAAAAAGUCAGGCAUCACCGAGGUGUGCAGAGAAGAGGCCCGCCGCGCCCUCAAGGACGGUGUGGCAACGGGAGGCCAUGCUGUGUCCCGAGGAAGUGCAAAGCUGAGAUGGUUGGUGGAGCGGGGAUACCUGCAGCCCUAUG
KX548902       CGCUUGAACCAGAUGUCGGCCCUGGAGUUCUACUCCUACAAAAAGUCAGGCAUCACCGAGGUGUGCAGAGAAGAGGCCCGCCGCGCCCUCAAGGACGGUGUGGCAACGGGAGGCCAUGCUGUGUCCCGAGGAAGUGCAAAGCUGAGAUGGUUGGUGGAGCGGGGAUACCUGCAGCCCUAUG
KX601166       CGCCUGAACCAGAUGUCGGCCUUGGAGUUCUACUCUUACAAAAAGUCAGGCAUCACCGAAGUGUGUAGGGAGGAGGCGCGCCGCGCCCUCAAGGAUGGAGUGGCCACAGGAGGACAUGCUGUAUCCCGGGGAAGCGCAAAGCUUAGAUGGUUGGUAGAGAGAGGAUACCUGCAGCCCCAUG
KX601167       CGCCUGAACCAGAUGUCGGCCCUGGAGUUUUACUCCUACAAAAAGUCAGGCAUCACCGAAGUGUGCAGAGAAGAAGCCCGCCGCGCCCUCAAGGACGGAGUGGCAACAGGAGGCCAUGCUGUGUCCCGAGGAAGCGCAAAGCUUAGAUGGUUGGUGGAGAGAGGAUACCUGCAGCCCUAUG
KX601168       CGCUUGAACCAGAUGUCGGCCCUGGAGUUCUACUCCUACAAAAAGUCAGGCAUCACCGAGGUGUGCAGAGAAGAGGCCCGCCGCGCCCUCAAGGACGGUGUGGCAACGGGAGGCCAUGCUGUGUCCCGAGGAAGUGCAAAGCUGAGAUGGUUGGUGGAGCGGGGAUACCUGCAGCCCUAUG
KX766028       CGCUUGAACCAGAUGUCGGCCCUGGAGUUCUACUCCUACAAAAAGUCAGGCAUCACCGAGGUGUGCAGAGAAGAGGCCCGCCGCGCCCUCAAGGACGGUGUGGCAACGGGAGGCCAUGCUGUGUCCCGAGGAAGUGCAAAGCUGAGAUGGUUGGUGGAGCGGGGAUACCUGCAGCCCUAUG
KX766029       CGCUUGAACCAGAUGUCGGCCCUGGAGUUCUACUCCUACAAAAAGUCAGGCAUCACCGAGGUGUGCAGAGAAGAGGCCCGCCGCGCCCUCAAGGACGGUGUGGCAACGGGAGGCCAUGCUGUGUCCCGAGGAAGUGCAAAGCUGAGAUGGUUGGUGGAGCGGGGAUACCUGCAGCCCUAUG
KU940224       CGCUUGAACCAGAUGUCGGCCCUGGAGUUCUACUCCUACAAAAAGUCAGGCAUCACCGAGGUGUGCAGAGAAGAGGCCCGCCGCGCCCUCAAGGACGGUGUGGCAACGGGAGGCCAUGCUGUGUCCCGAGGAAGUGCAAAGCUGAGAUGGUUGGUGGAGCGGGGAUACCUGCAGCCCCAUG


               7777777777777777777777777777777777777777777777777777777777777777777777777777777777777777777777777777777777777777777777777777777777777777777777777777777777777777777777777
               7777777777777777888888888888888888888888888888888888888888888888888888888888888888888888888888888888888888888888888888999999999999999999999999999999999999999999999999999999999999999
               8888889999999999000000000011111111112222222222333333333344444444445555555555666666666677777777778888888888999999999900000000001111111111222222222233333333334444444444555555555566666
Sequence       4567890123456789012345678901234567890123456789012345678901234567890123456789012345678901234567890123456789012345678901234567890123456789012345678901234567890123456789012345678901234
----------------------------------------------------------------------------------------------------------------------------------------------------------------------------------------------------
NC_012532      GGAAGGUUGUUGACCUCGGAUGUGGCAGAGGGGGCUGGAGCUAUUAUGCCGCCACCAUCCGCAAAGUGCAGGAGGUGAGAGGAUACACAAAGGGAGGUCCCGGUCAUGAAGAACCCAUGCUGGUGCAAAGCUAUGGGUGGAACAUAGUUCGUCUCAAGAGUGGAGUGGACGUCUUCCACAU
KU922960       GAAAGGUCAUUGAUCUUGGAUGUGGCAGAGGGGGCUGGAGUUACUACGCCGCCACCAUCCGCAAAGUUCAAGAAGUGAAAGGAUACACAAAAGGAGGCCCUGGUCAUGAAGAACCCGUGUUGGUGCAAAGCUAUGGGUGGAACAUAGUCCGUCUUAAGAGUGGGGUGGACGUCUUUCAUAU
KU922923       GAAAGGUCAUUGAUCUUGGAUGUGGCAGAGGGGGCUGGAGUUACUACGCCGCCACCAUCCGCAAAGUUCAAGAAGUGAAAGGAUACACAAAAGGAGGCCCUGGUCAUGAAGAACCCGUGUUGGUGCAAAGCUAUGGGUGGAACAUAGUCCGUCUUAAGAGUGGGGUGGACGUCUUUCAUAU
KU820898       GAAAGGUCAUUGAUCUUGGAUGUGGCAGAGGGGGCUGGAGUUACUACGCCGCCACCAUCCGCAAAGUUCAAGAAGUGAAAGGAUACACAAAAGGAGGCCCUGGUCAUGAAGAACCCGUGUUGGUGCAAAGCUAUGGGUGGAACAUAGUCCGUCUUAAGAGUGGGGUGGACGUCUUUCAUAU
KU321639       GAAAGGUCAUUGAUCUUGGAUGUGGCAGAGGGGGCUGGAGUUACUACGCCGCCACCAUCCGCAAAGUUCAAGAAGUGAAAGGAUACACAAAAGGAGGCCCUGGUCAUGAAGAACCCGUGUUGGUGCAAAGCUAUGGGUGGAACAUAGUCCGUCUUAAGAGUGGGGUGGACGUCUUUCAUAU
KU820899       GAAAGGUCAUUGAUCUUGGAUGUGGCAGAGGGGGCUGGAGUUACUACGCCGCCACCAUCCGCAAAGUUCAAGAAGUGAAAGGAUACACAAAAGGAGGCCCUGGUCAUGAAGAACCCAUGUUGGUGCAAAGCUAUGGGUGGAACAUAGUCCGUCUUAAGAGUGGGGUGGACGUCUUUCAUAU
KU853012       GAAAGGUCAUUGAUCUUGGAUGUGGCAGAGGGGGCUGGAGUUACUACGCCGCCACCAUCCGCAAAGUUCAAGAAGUGAAAGGAUACACAAAAGGAGGCCCUGGUCAUGAAGAACCCGUGUUGGUGCAAAGCUAUGGGUGGAACAUAGUCCGUCUCAAGAGUGGGGUGGACGUCUUUCAUAU
KU729217       GAAAGGUCAUUGAUCUUGGAUGUGGCAGAGGGGGCUGGAGUUACUACGCCGCCACCAUCCGCAAAGUUCAAGAAGUGAAAGGAUACACAAAAGGAGGCCCUGGUCAUGAAGAACCCGUGUUGGUGCAAAGCUAUGGGUGGAACAUAGUCCGUCUCAAGAGUGGGGUGGACGUCUUUCAUAU
KU729218       GAAAGGUCAUUGAUCUUGGAUGUGGCAGAGGGGGCUGGAGUUACUACGCCGCCACCAUCCGCAAAGUUCAAGAAGUGAAAGGAUACACAAAAGGAGGCCCUGGUCAUGAAGAACCCGUGUUGGUGCAAAGCUAUGGGUGGAACAUAGUCCGUCUUAAGAGUGGGGUGGACGUCUUUCAUAU
KU761564       GAAAGGUCAUUGAUCUUGGAUGUGGCAGAGGGGGCUGGAGUUACUACGCCGCCACCAUCCGCAAAGUUCAAGAAGUGAAAGGAUACACAAAAGGAGGCCCUGGUCAUGAAGAACCCGUGUUGGUGCAAAGCUAUGGGUGGAACAUAGUCCGUCUUAAGAGUGGGGUGGACGUCUUUCAUAU
```

```
KU740184       GAAAGGUCAUUGAUCUUGGAUGUGGCAGAGGGGGCUGGAGUUACUACGCCGCCACCAUCCGCAAAGUUCAAGAAGUGAAAGGAUACACAAAAGGAGGCCCUGGUCAUGAAGAACCCGUGUUGGUGCAAAGCUAUGGGUGGAACAUAGUCCGUCUUAAGAGUGGGGUGGACGUCUUUCAUAU
KU744693       GAAAGGUCAUUGAUCUUGGAUGUGGCAGAGGGGGCUGGAGUUACUACGCCGCCACCAUCCGCAAAGUUCAAGAAGUGAAAGGAUACACAAAAGGAGGCCCUGGUCAUGAAGAACCCGUGUUGGUGCAAAGCUAUGGGUGGAACAUAGUCCGUCUUAAGAGUGGGGUGGACGUCUUUCAUAU
KU497555       GAAAGGUCAUUGAUCUUGGAUGUGGCAGAGGGGGCUGGAGUUACUACGCCGCCACCAUCCGCAAAGUUCAAGAAGUGAAAGGAUACACAAAAGGAGGCCCUGGUCAUGAAGAACCCGUGUUGGUGCAAAGCUAUGGGUGGAACAUAGUCCGUCUUAAGAGUGGGGUGGACGUCUUUCAUAU
KU527068       GAAAGGUCAUUGAUCUUGGAUGUGGCAGAGGGGGCUGGAGUUACUACGCCGCCACCAUCCGCAAAGUUCAAGAAGUGAAAGGAUACACAAAAGGAGGCCCUGGUCAUGAAGAACCCGUGUUGGUGCAAAGCUAUGGGUGGAACAUAGUCCGUCUUAAGAGUGGGGUGGACGUCUUUCAUAU
KU501217       GAAAGGUCAUUGAUCUUGGAUGUGGCAGAGGGGGCUGGAGUUACUACGCCGCCACCAUCCGCAAAGUUCAAGAAGUGAAAGGAUACACAAAAGGAGGCCCUGGUCAUGAAGAACCCGUGUUGGUGCAAAGCUAUGGGUGGAACAUAGUCCGUCUUAAGAGUGGGGUGGACGUCUUUCAUAU
KU501216       GAAAGGUCAUUGAUCUUGGAUGUGGCAGAGGGGGCUGGAGUUACUACGCCGCCACCAUCCGCAAAGUUCAAGAAGUGAAAGGAUACACAAAAGGAGGCCCUGGUCAUGAAGAACCCGUGUUGGUGCAAAGCUAUGGGUGGAACAUAGUCCGUCUUAAGAGUGGGGUGGACGUCUUUCAUAU
KU501215       GAAAGGUCAUUGAUCUUGGAUGUGGCAGAGGGGGCUGGAGUUACUACGUCGCCACCAUCCGCAAAGUUCAAGAAGUGAAAGGAUACACAAAAGGAGGCCCUGGUCAUGAAGAACCCGUGUUGGUGCAAAGCUAUGGGUGGAACAUAGUCCGUCUUAAGAGUGGGGUGGACGUCUUUCAUAU
KU365780       GAAAGGUCAUUGAUCUUGGAUGUGGCAGAGGGGGCUGGAGUUACUACGCCGCCACCAUCCGCAAAGUUCAAGAAGUGAAAGGAUACACAAAAGGAGGCCCUGGUCAUGAAGAACCCGUGUUGGUGCAAAGCUAUGGGUGGAACAUAGUCCGUCUUAAGAGUGGGGUGGACGUCUUUCAUAU
KU365779       GAAAGGUCAUUGAUCUUGGAUGUGGCAGAGGGGGCUGGAGUUACUACGCCGCCACCAUCCGCAAAGUUCAAGAAGUGAAAGGAUACACAAAAGGAGGCCCUGGUCAUGAAGAACCCGUGUUGGUGCAAAGCUAUGGGUGGAACAUAGUCCGUCUUAAGAGUGGGGUGGACGUCUUUCAUAU
KU707826       GAAAGGUCAUUGAUCUUGGAUGUGGCAGAGGGGGCUGGAGUUACUACGCCGCCACCAUCCGCAAAGUUCAAGAAGUGAAAGGAUACACAAAAGGAGGCCCUGGUCAUGAAGAACCCGUGUUGGUGCAAAGCUAUGGGUGGAACAUAGUCCGUCUUAAGAGUGGGGUGGACGUCUUUCAUAU
KU365778       GAAAGGUCAUUGAUCUUGGAUGUGGCAGAGGGGGCUGGAGUUACUACGCCGCCACCAUCCGCAAAGUUCAAGAAGUGAAAGGAUACACAAAAGGAGGCCCUGGUCAUGAAGAACCCGUGUUGGUGCAAAGCUAUGGGUGGAACAUAGUCCGUCUUAAGAGUGGGGUGGACGUCUUUCAUAU
KU365777       GAAAGGUCAUUGAUCUUGGAUGUGGCAGAGGGGGCUGGAGUUACUACGCCGCCACCAUCCGCAAAGUUCAAGAAGUGAAAGGAUACACAAAAGGAGGCCCUGGUCAUGAAGAACCCGUGUUGGUGCAAAGCUAUGGGUGGAACAUAGUCCGUCUUAAGAGUGGGGUGGACGUCUUUCAUAU
KU312312       GAAAGGUCAUUGAUCUUGGAUGUGGCAGAGGGGGCUGGAGUUACUACGCCGCCACCAUCCGCAAAGUUCAAGAAGUGAAAGGAUACACAAAAGGAGGCCCUGGUCAUGAAGAACCCGUGUUGGUGCAAAGCUAUGGGUGGAACAUAGUCCGUCUUAAGAGUGGGGUGGACGUCUUUCAUAU
KF268950       GAAAGGUUGUUGACCUCGGAUGUGGCAGAGGGGGUUGGAGCUAUUAUGCCGCCACCAUCCGGAAAGUGCAGGAGGUGAAAGGAUACACAAAGGGAGGUCCCGGUCAUGAAGAACCCAUGCUGGUGCAAAGCUAUGGGUGGAACAUCGUUCGUCUCAAGAGUGGAGUGGACGUCUUCCACAU
KF268949       GAAAGGUUGUUGACCUCGGAUGUGGCAGAGGGGGUUGGAGCUAUUAUGCCGCCACCAUCCGGAAAGUGCAGGAGGUGAAAGGAUACACAAAGGGAGGUCCUGGUCAUGAAGAACCCAUGCUGGUGCAAAGCUAUGGGUGGAACAUAGUUCGUCUCAAGAGUGGAGUGGAUGUCUUCCACAU
KF268948       GAAAGGUUGUUGACCUCGGAUGUGGCAGAGGGGGUUGGAGCUAUUAUGCCGCCACCAUCCGGAAAGUGCAGGAGGUGAAAGGAUACACAAAGGGAGGUCCCGGUCAUGAAGAACCCAUGCUGGUGCAAAGCUAUGGGUGGAACAUCGUUCGUCUCAAGAGUGGAGUGGACGUCUUCCACAU
KJ776791       GAAAGGUCAUUGAUCUUGGAUGUGGCAGAGGGGGCUGGAGUUACUACGCCGCCACCAUCCGCAAAGUUCAAGAAGUGAAAGGAUACACAAAAGGAGGCCCUGGUCAUGAAGAACCCAUGUUGGUGCAAAGCUAUGGGUGGAACAUAGUCCGUCUUAAGAGUGGGGUGGACGUCUUUCAUAU
KF383119       GGAAGGUUGUUGACCUCGGAUGUGGCAGAGGGGGCUGGAGCUAUUAUGCCGCCACCAUCCGCAAAGUGCAGGAGGUGAGAGGAUACACAAAGGGAGGUCCCGGUCAUGAAGAACCCAUGCUGGUGCAAAGCUAUGGGUGGAACAUAGUUCGUCUCAAGAGUGGAGUGGACGUCUUCCACAU
KF383118       GGAAGGUUGUUGACCUCGGAUGUGGCAGAGGGGGCUGGAGCUAUUAUGCCGCCACCAUCCGCAAAGUGCAGGAGGUGAGAGGAUACACAAAGGGAGGUCCCGGUCAUGAAGAACCCAUGCUGGUGCAAAGCUAUGGGUGGAACAUAGUUCGUCUCAAGAGUGGAGUGGACGUCUUCCACAU
KF383117       GAAAGGUUGUUGACCUUGGAUGUGGCAGAGGAGGCUGGAGUUAUUACGCUGCCACUAUCCGUAAAGUGCAGGAGGUCAGAGGAUACACAAAGGGAGGUCCUGGUCAUGAAGAACCCAUGCUGGUGCAAAGCUAUGGGUGGAACAUAGUUCGCCUCAAGAGUGGAGUGGACGUCUUCCACAU
KF383116       GAAAGGUUGUUGACCUUGGAUGUGGCAGAGGAGGCUGGAGUUAUUACGCUGCCACCAUCCGUAAAGUGCAGGAGGUCAGAGGAUACACAAAGGGAGGUCCUGGUCAUGAAGAACCCAUGCUGGUGCAAAGCUAUGGGUGGAACAUAGUUCGCCUCAAGAGUGGAGUGGACGUCUUCCACAU
KF383115       GAAAGGUUGUUGACCUCGGAUGUGGCAGAGGGGGUUGGAGCUAUUAUGCCGCUACCAUCCGGAAAGUGCAGGAGGUGAAAGGAUACACAAAGGGAGGUCCCGGUCAUGAAGAACCCAUGCUGGUGCAAAGCUAUGGAUGGAACAUAGUUCGUCUCAAGAGUGGAGUGGACGUCUUCCACAU
EU545988       GAAAGGUCAUUGAUCUUGGAUGUGGCAGAGGGGGCUGGAGUUACUACGCCGCCACCAUCCGCAAAGUUCAAGAAGUGAAAGGAUACACAAAAGGAGGCCCUGGUCAUGAAGAACCCAUGUUGGUGCAAAGCUAUGGGUGGAACAUAGUCCGUCUUAAGAGUGGGGUGGACGUCUUUCAUAU
KU955589       GAAAGGUCAUUGAUCUUGGAUGUGGCAGAGGGGGCUGGAGUUACUACGCCGCCACCAUCCGCAAAGUUCAAGAAGUGAAAGGAUACACAAAAGGAGGCCCUGGUCAUGAAGAACCCAUGUUGGUGCAAAGCUAUGGGUGGAACAUAGUCCGUCUUAAGAGUGGGGUGGACGUCUUUCAUAU
KU955590       GAAAGGUCAUUGAUCUUGGAUGUGGCAGAGGGGGCUGGAGUUACUACGCCGCCACCAUCCGCAAAGUUCAAGAAGUGAAAGGAUACACAAAAGGAGGCCCUGGUCAUGAAGAACCCGUGUUGGUGCAAAGCUAUGGGUGGAACAUAGUCCGUCUUAAGAGUGGGGUGGACGUCUUUCAUAU
KU940228       GAAAGGUCAUUGAUCUUGGAUGUGGCAGAGGGGGCUGGAGUUACUACGCCGCCACCAUCCGCAAAGUUCAAGAAGUGAAAGGAUACACAAAAGGAGGCCCUGGUCAUGAAGAACCCGUGUUGGUGCAAAGCUAUGGGUGGAACAUAGUCCGUCUUAAGAGUGGGGUGGACGUCUUUCAUAU
KU991811       GAAAGGUCAUUGAUCUUGGAUGUGGCAGAGGGGGCUGGAGUUACUACGCCGCCACCAUCCGCAAAGUUCAAGAAGUGAAAGGAUACACAAAAGGAGGCCCUGGUCAUGAAGAACCCGUGUUGGUGCAAAGCUAUGGGUGGAACAUAGUCCGUCUUAAGAGUGGGGUGGACGUCUUUCAUAU
KU509998       GAAAGGUCAUUGAUCUUGGAUGUGGCAGAGGGGGCUGGAGUUACUACGCCGCCACCAUCCGCAAAGUUCAAGAAGUGAAAGGAUACACAAAAGGAGGCCCUGGUCAUGAAGAACCCGUGUUGGUGCAAAGCUAUGGGUGGAACAUAGUCCGUCUUAAGAGUGGGGUGGACGUCUUUCAUAU
KX056898       GAAAGGUCAUUGAUCUUGGAUGUGGCAGAGGGGGCUGGAGUUACUACGCCGCCACCAUCCGCAAAGUUCAAGAAGUGAAAGGAUACACAAAAGGAGGCCCUGGUCAUGAAGAACCCGUGUUGGUGCAAAGCUAUGGGUGGAACAUAGUCCGUCUUAAGAGUGGGGUGGACGUCUUUCAUAU
KX117076       GAAAGGUCAUUGAUCUUGGAUGUGGCAGAGGGGGCUGGAGUUACUACGCCGCCACCAUCCGCAAAGUUCAAGAAGUGAAAGGAUACACAAAAGGAGGCCCUGGUCAUGAAGAACCCAUGUUGGUGCAAAGCUAUGGGUGGAACAUAGUCCGUCUUAAGAGUGGGGUGGACGUCUUUCAUAU
KX185891       GAAAGGUCAUUGAUCUUGGAUGUGGCAGAGGGGGCUGGAGUUACUACGCCGCCACCAUCCGCAAAGUUCAAGAAGUGAAAGGAUACACAAAAGGAGGCCCUGGUCAUGAAGAACCCAUGUUGGUGCAAAGCUAUGGGUGGAACAUAGUCCGUCUUAAGAGUGGGGUGGACGUCUUUCAUAU
KX247646       GAAAGGUCAUUGAUCUUGGAUGUGGCAGAGGGGGCUGGAGUUACUACGCCGCCACCAUCCGCAAAGUUCAAGAAGUGAAAGGAUACACAAAAGGAGGCCCUGGUCAUGAAGAACCCGUGUUGGUGCAAAGCUAUGGGUGGAACAUAGUCCGUCUUAAGAGUGGGGUGGACGUCUUUCAUAU
KX262887       GAAAGGUCAUUGAUCUUGGAUGUGGCAGAGGGGGCUGGAGUUACUACGCCGCCACCAUCCGCAAAGUUCAAGAAGUGAAAGGAUACACAAAAGGAGGCCCUGGUCAUGAAGAACCCGUGUUGGUGCAAAGCUAUGGGUGGAACAUUGUCCGUCUUAAGAGUGGGGUGGACGUCUUUCAUAU
KX280026       GAAAGGUCAUUGAUCUUGGAUGUGGCAGAGGGGGCUGGAGUUACUACGCCGCCACCAUCCGCAAAGUUCAAGAAGUGAAAGGAUACACAAAAGGAGGCCCUGGUCAUGAAGAACCCGUGUUGGUGCAAAGCUAUGGGUGGAACAUAGUCCGUCUUAAGAGUGGGGUGGACGUCUUUCAUAU
KU758877       GAAAGGUCAUUGAUCUUGGAUGUGGCAGAGGGGGCUGGAGUUACUACGCCGCCACCAUCCGCAAAGUUCAAGAAGUGAAAGGAUACACAAAAGGAGGCCCUGGUCAUGAAGAACCCGUGUUGGUGCAAAGCUAUGGGUGGAACAUAGUCCGUCUUAAGAGUGGGGUGGACGUCUUUCAUAU
KU866423       GAAAGGUCAUUGAUCUUGGAUGUGGCAGAGGGGGCUGGAGUUACUACGCCGCCACCAUCCGCAAAGUUCAAGAAGUGAAAGGAUACACAAAAGGAGGCCCUGGUCAUGAAGAACCCAUGUUGGUGCAAAGCUAUGGGUGGAACAUAGUCCGUCUUAAGAGUGGGGUGGACGUCUUUCAUAU
KX377336       GAAAGGUCAUUGAUCUUGGAUGUGGCAGAGGGGGCUGGAGUUACUACGCCGCCACCAUCCGCAAAGUUCAAGAGGUGAAAGGAUACACAAAGGGAGGCCCUGGUCAUGAAGAACCCACGUUGGUGCAAAGCUAUGGAUGGAACAUAGUCCGUCUUAAGAGUGGGGUGGACGUCUUUCACAU
KX377337       GAAAGGUCAUUGAUCUUGGAUGUGGCAGAGGGGGCUGGAGUUACUACGUCGCCACCAUCCGCAAAGUUCAAGAAGUGAAAGGAUACACAAAAGGAGGCCCUGGUCAUGAAGAACCCGUGUUGGUGCAAAGCUAUGGGUGGAACAUAGUCCGUCUUAAGAGUGGGGUGGACGUCUUUCAUAU
KX369547       GAAAGGUCAUUGAUCUUGGAUGUGGCAGAGGGGGCUGGAGUUACUACGCCGCCACCAUCCGCAAAGUUCAAGAAGUGAAAGGAUACACAAAAGGAGGCCCUGGUCAUGAAGAACCCAUGUUGGUGCAAAGCUAUGGGUGGAACAUAGUCCGUCUUAAGAGUGGGGUGGACGUCUUUCAUAU
KX548902       GAAAGGUCAUUGAUCUUGGAUGUGGCAGAGGGGGCUGGAGUUACUACGCCGCCACCAUCCGCAAAGUUCAAGAAGUGAAAGGAUACACAAAAGGAGGCCCUGGUCAUGAAGAACCCGUGUUGGUGCAAAGCUAUGGGUGGAAUAUAGUCCGUCUUAAGAGUGGGGUGGACGUCUUUCAUAU
KX601166       GAAAGGUUGUUGACCUUGGAUGUGGCAGAGGAGGCUGGAGUUAUUACGCUGCCACCAUCCGUAAAGUGCAGGAGGUCAGAGGAUACACAAAGGGAGGUCCUGGCCAUGAAGAGCCCAUGCUGGUGCAAAGCUAUGGGUGGAACAUAGUUCGCCUCAAGAGUGGAGUGGACGUCUUCCACAU
KX601167       GAAAGGUCAUUGAUCUUGGAUGUGGCAGAGGGGGCUGGAGUUACUACGCCGCCACCAUCCGCAAAGUUCAAGAGGUGAAAGGAUACACAAAGGGAGGCCCUGGUCAUGAAGAACCCACGUUGGUGCAAAGCUAUGGAUGGAACAUAGUCCGUCUUAAGAGUGGGGUGGACGUCUUUCACAU
KX601168       GAAAGGUCAUUGAUCUUGGAUGUGGCAGAGGGGGCUGGAGUUACUACGUCGCCACCAUCCGCAAAGUUCAAGAAGUGAAAGGAUACACAAAAGGAGGCCCUGGUCAUGAAGAACCCGUGUUGGUGCAAAGCUAUGGGUGGAACAUAGUCCGUCUUAAGAGUGGGGUGGACGUCUUUCAUAU
KX766028       GAAAGGUCAUUGAUCUUGGAUGUGGCAGAGGGGGCUGGAGUUACUACGCCGCCACCAUCCGCAAAGUUCAAGAAGUGAAAGGAUACACAAAAGGAGGCCCUGGUCAUGAAGAACCCGUGUUGGUGCAAAGCUAUGGGUGGAACAUAGUCCGUCUUAAGAGUGGGGUGGACGUCUUUCAUAU
KX766029       GAAAGGUCAUUGAUCUUGGAUGUGGCAGAGGGGGCUGGAGUUACUACGCCGCCACCAUCCGCAAAGUUCAAGAAGUGAAAGGAUACACAAAAGGAGGCCCUGGUCAUGAGGAACCCGUGUUGGUGCAAAGCUAUGGGUGGAACAUAGUCCGUCUUAAGAGUGGGGUGGACGUCUUUCAUAU
KU940224       GAAAGGUCAUUGAUCUUGGAUGUGGCAGAGGGGGCUGGAGUUACUACGCCGCCACCAUCCGCAAAGUUCAAGAAGUGAAAGGAUACACAAAAGGAGGCCCUGGUCAUGAAGAACCCGUGUUGGUGCAAAGCUAUGGGUGGAACAUAGUCCGUCUUAAGAGUGGGGUGGACGUCUUUCAUAU


               77777777777777777777777777777777777888888888888888888888888888888888888888888888888888888888888888888888888888888888888888888888888888888888888888888888888888888888888888888888888
               99999999999999999999999999999999999000000000000000000000000000000000000000000000000000000000000000000000000000000000000000000000000000001111111111111111111111111111111111111111111111
               66666777777777788888888889999999999000000000011111111112222222222333333333344444444445555555555666666666677777777778888888888999999999900000000001111111111222222222233333333334444444
Sequence       56789012345678901234567890123456789012345678901234567890123456789012345678901234567890123456789012345678901234567890123456789012345678901234567890123456789012345678901234567890123456789012345
----------------------------------------------------------------------------------------------------------------------------------------------------------------------------------------------------------------
NC_012532      GGCGGCUGAGCCGUGUGACACUCUGCUGUGUGACAUAGGUGAGUCAUCAUCUAGUCCUGAAGUGGAAGAGACACGAACACUCAGAGUGCUCUCUAUGGUGGGGGACUGGCUUGAAAAAAGACCAGGGGCCUUCUGUAUAAAGGUGCUGUGCCCAUACACCAGCACUAUGAUGGAAACCAUG
KU922960       GGCGGCUGAGCCGUGUGACACGUUGCUGUGUGACAUAGGUGAGUCAUCAUCUAGUCCUGAAGUGGAAGAAGCACGGACGCUCAGAGUCCUCUCCAUGGUGGGGGAUUGGCUUGAAAAAAGACCAGGAGCCUUUUGUAUAAAAGUGUUGUGCCCAUACACCAGCACUAUGAUGGAAACCCUG
KU922923       GGCGGCUGAGCCGUGUGACACGUUGCUGUGUGACAUAGGUGAGUCAUCAUCUAGUCCUGAAGUGGAAGAAGCACGGACGCUCAGAGUCCUCUCCAUGGUGGGGGAUUGGCUUGAAAAAAGACCAGGAGCCUUUUGUAUAAAAGUGUUGUGCCCAUACACCAGCACUAUGAUGGAAACCCUG
KU820898       GGCGGCUGAGCCGUGUGACACGUUGCUGUGUGACAUAGGUGAGUCAUCAUCUAGUCCUGAAGUGGAAGAAGCACGGACGCUCAGAGUCCUCUCCAUGGUGGGGGAUUGGCUUGAAAAAAGACCAGGAGCCUUUUGCAUAAAAGUGUUGUGCCCAUACACCAGCACUAUGAUGGAAACCCUG
KU321639       GGCGGCUGAGCCGUGUGACACGUUGCUGUGUGACAUAGGUGAGUCAUCAUCUAGUCCUGAAGUGGAAGAAGCACGGACGCUCAGAGUCCUCUCCAUGGUGGGGGAUUGGCUUGAAAAAAGACCAGGAGCCUUUUGUAUAAAAGUGUUGUGCCCAUACACCAGCACUAUGAUGGAAACCCUG
KU820899       GGCGGCUGAGCCGUGUGACACGUUGCUGUGUGACAUAGGUGAGUCAUCAUCUAGUCCUGAAGUGGAAGAAGCACGGACGCUCAGAGUCCUUUCCAUGGUGGGGGAUUGGCUUGAAAAAAGACCAGGAGCCUUUUGUAUAAAAGUGUUGUGCCCAUACACCAGCACUAUGAUGGAAACCCUG
KU853012       GGCGGCUGAGCCGUGUGACACGUUGCUGUGUGACAUAGGUGAGUCAUCAUCUAGUCCUGAAGUGGAAGAAGCACGGACGCUCAGAGUCCUCUCCAUGGUGGGGGAUUGGCUUGAAAAAAGACCAGGAGCCUUUUGUAUAAAAGUGUUGUGCCCAUACACCAGCACUAUGAUGGAAACCCUG
KU729217       GGCGGCUGAGCCGUGUGACACGUUGCUGUGUGACAUAGGUGAGUCAUCAUCUAGUCCUGAAGUGGAAGAAGCACGGACGCUCAGAGUCCUCUCCAUGGUGGGGGAUUGGCUUGAAAAAAGACCAGGAGCCUUUUGCAUAAAAGUGUUGUGCCCAUACACCAGCACUAUGAUGGAAACCUUG
KU729218       GGCGGCUGAGCCGUGUGACACGUUGCUGUGUGACAUAGGUGAGUCAUCAUCUAGUCCUGAAGUGGAAGAAGCACGGACGCUCAGAGUCCUCUCCAUGGUGGGGGAUUGGCUUGAAAAAAGACCAGGAGCCUUUUGUAUAAAAGUGUUGUGCCCAUACACCAGCACUAUGAUGGAAACCCUG
KU761564       GGCGGCUGAGCCGUGUGACACGUUGCUGUGUGACAUAGGUGAGUCAUCAUCUAGUCCUGAAGUGGAAGAAGCACGGACGCUCAGAGUCCUCUCCAUGGUGGGGGAUUGGCUUGAAAAAAGACCAGGAGCCUUUUGCAUAAAAGUGUUGUGCCCAUACACCAGCACUAUGAUGGAAACCCUG
KU740184       GGCGGCUGAGCCGUGUGACACGUUGCUGUGUGACAUAGGUGAGUCAUCAUCUAGUCCUGAAGUGGAAGAAGCACGGACGCUCAGAGUCCUCUCCAUGGUGGGGGAUUGGCUUGAAAAAAGACCAGGAGCCUUUUGCAUAAAAGUGUUGUGCCCAUACACCAGCACUAUGAUGGAAACCCUG
KU744693       GGCGGCUGAGCCGUGUGACACGUUGCUGUGUGACAUAGGUGAGUCAUCAUCUAGUCCUGAAGUGGAAGAAGCACGGACGCUCAGAGUCCUCUCCAUGGUGGGGGAUUGGCUUGAAAAAAGACCAGGAGCCUUUUGUAUAAAAGUGUUGUGCCCAUACACCAGCACUAUGAUGGAAACCCUG
KU497555       GGCGGCUGAGCCGUGUGACACGUUGCUGUGUGACAUAGGUGAGUCAUCAUCUAGUCCUGAAGUGGAAGAAGCACGGACGCUCAGAGUCCUCUCCAUGGUGGGGGAUUGGCUUGAAAAAAGACCAGGAGCCUUUUGCAUAAAAGUGUUGUGCCCAUACACCAGCACUAUGAUGGAAACCCUG
KU527068       GGCGGCUGAGCCGUGUGACACGUUGCUGUGUGACAUAGGUGAGUCAUCAUCUAGUCCUGAAGUGGAAGAAGCACGGACGCUCAGAGUCCUCUCCAUGGUGGGGGAUUGGCUUGAAAAAAGACCAGGAGCCUUUUGUAUAAAAGUGUUGUGCCCAUACACCAGCACUAUGAUGGAAACCCUG
KU501217       GGCGGCUGAGCCGUGUGACACGUUGCUGUGUGACAUAGGCGAGUCAUCAUCUAGUCCUGAAGUGGAAGAAGCACGGACGCUCAGAGUCCUCUCCAUGGUGGGGGAUUGGCUUGAAAGAAGACCAGGAGCCUUUUGUAUAAAAGUGUUGUGCCCAUACACCAGCACUAUGAUGGAAACCCUG
KU501216       GGCGGCUGAGCCGUGUGACACGUUGCUGUGUGACAUAGGCGAGUCAUCAUCUAGUCCUGAAGUGGAAGAAGCACGGACGCUCAGAGUCCUCUCCAUGGUGGGGGAUUGGCUUGAAAGAAGACCAGGAGCCUUUUGUAUAAAAGUGUUGUGCCCAUACACCAGCACUAUGAUGGAAACCCUG
KU501215       GGCGGCUGAGCCGUGUGACACGUUGCUGUGUGACAUAGGUGAGUCAUCAUCUAGUCCUGAAGUGGAAGAAGCACGGACGCUCAGAGUCCUCUCCAUGGUGGGGGAUUGGCUUGAAAAAAGACCAGGAGCCUUUUGUAUAAAAGUGUUGUGCCCAUACACCAGCACUAUGAUGGAAACCCUG
KU365780       GGCGGCUGAGCCGUGUGACACGUUGCUGUGUGACAUAGGUGAGUCAUCAUCUAGUCCUGAAGUGGAAGAAGCACGGACGCUCAGAGUCCUCUCCAUGGUGGGGGAUUGGCUUGAAAAAAGACCAGGAGCCUUUUGUAUAAAGGUGUUGUGCCCAUACACCAGCACUAUGAUGGAAACCCUG
KU365779       GGCGGCUGAGCCGUGUGACACGUUGCUGUGUGACAUAGGUGAGUCAUCAUCUAGUCCUGAAGUGGAAGAAGCACGGACGCUCAGAGUCCUCUCCAUGGUGGGGGAUUGGCUUGAAAAAAGACCAGGAGCCUUUUGUAUAAAGGUGUUGUGCCCAUACACCAGCACUAUGAUGGAAACCCUG
KU707826       GGCGGCUGAGCCGUGUGACACGUUGCUGUGUGACAUAGGUGAGUCAUCAUCUAGUCCUGAAGUGGAAGAAGCACGGACGCUCAGAGUCCUCUCCAUGGUGGGGGAUUGGCUUGAAAAAAGACCAGGAGCCUUUUGUAUAAAGGUGUUGUGCCCAUACACCAGCACUAUGAUGGAAACCCUG
KU365778       GGCGGCUGAGCCGUGUGACACGCUGCUGUGUGACAUAGGUGAGUCAUCAUCUAGUCCUGAAGUGGAAGAAGCACGGACGCUCAGAGUCCUCUCCAUGGUGGGGGAUUGGCUUGAAAAAAGACCAGGAGCCUUUUGUAUAAAAGUGUUGUGCCCAUACACCAGCACUAUGAUGGAAACCCUG
KU365777       GGCGGCUGAGCCGUGUGACACGUUGCUGUGUGACAUAGGUGAGUCAUCAUCUAGUCCUGAAGUGGAAGAAGCACGGACGCUCAGAGUCCUCUCCAUGGUGGGGGAUUGGCUUGAAAAAAGACCAGGAGCCUUUUGUAUAAAGGUGUUGUGCCCAUACACCAGCACUAUGAUGGAAACCCUG
KU312312       GGCGGCUGAGCCGUGUGACACGUUGCUGUGUGACAUAGGUGAGUCAUCAUCUAGUCCUGAAGUGGAAGAAGCACGGACGCUCAGAGUCCUCUCCAUGGUGGGGGAUUGGCUUGAAAAAAGACCAGGAGCCUUUUGUAUAAAAGUGUUGUGCCCAUACACCAGCACUAUGAUGGAAACCCUG
KF268950       GGCGGCCGAGCCGUGUGAUACCUUGCUGUGUGACAUAGGUGAGUCAUCAUCUAGUCCUGAAGUGGAAGAGACGCGAACACUCCGAGUGCUCUCUAUGGUGGGGGACUGGCUUGAGAAAAGACCGGGGGCCUUCUGUAUAAAGGUGCUGUGUCCAUACACCAGCACUAUGAUGGAGACCAUG
KF268949       GGCGGCCGAGCCGUGUGACACCUUGCUGUGCGACAUAGGUGAGUCAUCAUCUAGUCCUGAAGUGGAAGAGACGCGAACACUCAGAGUGCUCUCUAUGGUGGGGGACUGGCUUGAGAAAAGACCGGGAGCCUUCUGCAUAAAGGUGCUGUGCCCAUACACCAGCACUAUGAUGGAGACCAUG
```

```
KF268948    GGCGGCCGAGCCGUGUGAUACCUUGCUGUGUGACAUAGGUGAGUCAUCAUCUAGUCCUGAAGUGGAAGAGACGCGAACACUCCGAGUGCUCUCUAUGGUGGGGGACUGGCUUGAGAAAAGACCGGGGGCCUUCUGUAUAAAGGUGCUGUGUCCAUACACCAGCACUAUGAUGGAGACCAUG
KJ776791    GGCGGCUGAGCCGUGUGACACGUUGCUGUGUGACAUAGGUGAGUCAUCAUCUAGUCCUGAAGUGGAAGAAGCACGGACGCUCAGAGUCCUCUCCAUGGUGGGGGAUUGGCUUGAAAAAAGACCAGGAGCCUUUUGUAUAAAAGUGUUGUGCCCAUACACCAGCACUAUGAUGGAAACCCUG
KF383119    GGCGGCUGAGCCGUGUGACACUCUGCUGUGUGACAUAGGUGAGUCAUCAUCUAGUCCUGAAGUGGAAGAGACACGAACACUCAGAGUGCUCUCUAUGGUGGGGGACUGGCUUGAAAAAAGACCAGGGGCCUUCUGUAUAAAGGUGCUGUGCCCAUACACCAGCACUAUGAUGGAAACCAUG
KF383118    GGCGGCUGAGCCGUGUGACACUCUGCUGUGUGACAUAGGUGAGUCAUCAUCUAGUCCUGAAGUGGAAGAGACACGAACACUCAGAGUGCUCUCUAUGGUGGGGGACUGGCUUGAAAAAAGACCAGGGGCCUUCUGUAUAAAGGUGCUGUGCCCAUACACCAGCACUAUGAUGGAAACCAUG
KF383117    GGCGGCUGAGCCGUGUGACACUCUGCUGUGUGACAUAGGCGAGUCAUCAUCUAGUCCUGAAGUGGAAGAGACGCGAACACUCAGAGUGCUCUCCAUGGUAGAAGACUGGCUUGAGAAAAGACCAGGGGCCUUCUGCAUAAAGGUGCUGUGCCCAUACACCAGCACAAUGAUGGAGACCAUG
KF383116    GGCGGCUGAGCCGUGUGACACUCUGCUGUGUGACAUCGGCGAGUCAUCAUCUAGUCCUGAAGUGGAAGAGACGCGAACACUCAGAGUGCUCUCCAUGGUGGGAGACUGGCUUGAGAAAAGACCAGGGGCCUUCUGCAUAAAGGUGCUGUGCCCAUACACCAGCACAAUGAUGGAGACCAUG
KF383115    GGCGGCUGAGCCGUGUGAUACUCUGCUGUGUGACAUAGGUGAGUCAUCAUCCAGUCCUGAAGUAGAAGAGACGCGAACACUCAGAGUGCUCUCUAUGGUGGGGGACUGGCUUGAAAAAAGACCAGGGGCCUUCUGCAUAAAGGUGCUGUGCCCAUACACCAGCACUAUGAUGGAGACCAUG
EU545988    GGCGGCUGAGCCGUGUGACACUUUGCUGUGUGAUAUAGGUGAGUCAUCAUCUAGUCCUGAAGUGGAAGAAGCACGGACGCUCAGAGUCCUUUCCAUGGUGGGGGAUUGGCUUGAAAAAAGACCAGGAGCCUUUUGUAUAAAAGUGUUGUGCCCAUACACCAGCACUAUGAUGGAAACCCUG
KU955589    GGCGGCUGAGCCGUGUGACACGUUGCUGUGUGACAUAGGUGAGUCAUCAUCUAGUCCUGAAGUGGAAGAAGCACGGACGCUCAGAGUCCUUUCCAUGGUGGGGGAUUGGCUUGAAAAAAGACCAGGAGCCUUUUGUAUAAAAGUGUUGUGCCCAUACACCAGCACUAUGAUGGAAACCCUG
KU955590    GGCGGCUGAGCCGUGUGACACGUUGCUGUGUGACAUAGGUGAGUCAUCAUCUAGUCCUGAAGUGGAAGAAGCACGGACGCUCAGAGUCCUCUCCAUGGUGGGGGAUUGGCUUGAAAAAAGACCAGGAGCCUUUUGCAUAAAAGUGUUGUGCCCAUACACCAGCACUAUGAUGGAAACCCUG
KU940228    GGCGGCUGAGCCGUGUGACACGUUGCUGUGUGACAUAGGUGAGUCAUCAUCUAGUCCUGAAGUGGAAGAAGCACGGACGCUCAGAGUUCUCUCCAUGGUGGGGGAUUGGCUUGAAAAAAGACCAGGAGCCUUUUGUAUAAAAGUGUUGUGCCCAUACACCAGCACUAUGAUGGAAACCCUG
KU991811    GGCGGCUGAGCCGUGUGACACGUUGCUGUGUGACAUAGGUGAGUCAUCAUCUAGUCCUGAAGUGGAAGAAGCACGGACGCUCAGAGUCCUCUCCAUGGUGGGGGAUUGGCUUGAAAAAAGACCAGGAGCCUUUUGUAUAAAAGUGUUGUGCCCAUACACCAGCACUAUGAUGGAAACCCUG
KU509998    GGCGGCUGAGCCGUGUGACACGUUGCUGUGUGACAUAGGUGAGUCAUCAUCUAGUCCUGAAGUGGAAGAAGCACGGACGCUCAGAGUCCUCUCCAUGGUGGGGGAUUGGCUUGAAAAAAGACCAGGAGCCUUUUGUAUAAAAGUGUUGUGCCCAUACACCAGCACUAUGAUGGAAACCCUG
KX056898    GGCGGCUGAGCCGUGUGACACGUUGCUGUGUGACAUAGGUGAGUCAUCAUCUAGUCCUGAAGUGGAAGAAGCACGGACGCUCAGAGUCCUCUCCAUGGUGGGGGAUUGGCUUGAAAAAAGACCAGGAGCCUUUUGCAUAAAAGUGUUGUGCCCAUACACCAGCACUAUGAUGGAAACCCUG
KX117076    GGCGGCUGAGCCGUGUGACACGUUGCUGUGUGACAUAGGUGAGUCAUCAUCUAGUCCUGAAGUGGAAGAAGCACGGACGCUCAGAGUCCUUUCCAUGGUGGGGGAUUGGCUUGAAAAAAGACCAGGAGCCUUUUGUAUAAAAGUGUUGUGCCCAUACACCAGCACUAUGAUGGAAACCCUG
KX185891    GGCGGCUGAGCCGUGUGACACGUUGCUGUGUGACAUAGGUGAGUCAUCAUCUAGUCCUGAAGUGGAAGAAGCACGGACGCUCAGAGUCCUUUCCAUGGUGGGGGAUUGGCUUGAAAAAAGACCAGGAGCCUUUUGUAUAAAAGUGUUGUGCCCAUACACCAGCACUAUGAUGGAAACCCUG
KX247646    GGCGGCUGAGCCGUGUGACACGUUGCUGUGUGACAUAGGUGAGUCAUCAUCUAGUCCUGAAGUGGAAGAAGCACGGACGCUCAGAGUCCUCUCCAUGGUGGGGGAUUGGCUUGAAAAAAGACCAGGAGCCUUUUGUAUAAAAGUGUUGUGCCCAUACACCAGCACUAUGAUGGAAACCCUG
KX262887    GGCGGCUGAGCCGUGUGACACGUUGCUGUGUGACAUAGGUGAGUCAUCAUCUAGUCCUGAAGUGGAAGAAGCACGGACGCUCAGAGUCCUCUCCAUGGUGGGGGAUUGGCUUGAAAAAAGACCAGGAGCCUUUUGUAUAAAAGUGUUGUGCCCAUACACCAGCACUAUGAUGGAAACCCUG
KX280026    GGCGGCUGAGCCGUGUGACACGUUGCUGUGUGACAUAGGUGAGUCAUCAUCUAGUCCUGAAGUGGAAGAAGCACGGACGCUCAGAGUCCUCUCCAUGGUGGGGGAUUGGCUUGAAAAAAGACCAGGAGCCUUUUGUAUAAAAGUGUUGUGCCCAUACACCAGCACUAUGAUGGAAACCCUG
KU758877    GGCGGCUGAGCCGUGUGACACGUUGCUGUGUGACAUAGGUGAGUCAUCAUCUAGUCCUGAAGUGGAAGAAGCACGGACGCUCAGAGUCCUCUCCAUGGUGGGGGAUUGGCUUGAAAAAAGACCAGGAGCCUUUUGUAUAAAAGUGUUGUGCCCAUACACCAGCACUAUGAUGGAAACCCUG
KU866423    GGCGGCUGAGCCGUGUGACACGUUGCUGUGUGACAUAGGUGAGUCAUCAUCUAGUCCUGAAGUGGAAGAAGCACGGACGCUCAGAGUCCUUUCCAUGGUGGGGGAUUGGCUUGAAAAAAGACCAGGAGCCUUUUGUAUAAAAGUGUUGUGUCCAUACACCAGCACUAUGAUGGAAACCCUG
KX377336    GGCGGCGGAGUCGUGUGACACUUUGCUGUGUGACAUAGGUGAGUCAUCAUCUAGUCCUGAAGUGGAAGAAGCACGGACGCUCAGAGUACUCUCCAUGGUGGGGGAUUGGCUUGAAAAAAGACCAGGGGCCUUUUGUAUAAAGGUGUUGUGCCCAUACACCAGCACCAUGAUGGAAACCCUA
KX377337    GGCGGCUGAGCCGUGUGACACGUUGCUGUGUGACAUAGGUGAGUCAUCAUCUAGUCCUGAAGUGGAAGAAGCACGGACGCUCAGAGUCCUCUCCAUGGUGGGGGAUUGGCUUGAAAAAAGACCAGGAGCCUUUUGUAUAAAAGUGUUGUGCCCAUACACCAGCACUAUGAUGGAAACCCUG
KX369547    GGCGGCUGAGCCGUGUGACACGUUGCUGUGUGACAUAGGUGAGUCAUCAUCUAGUCCUGAAGUGGAAGAAGCACGGACGCUCAGAGUCCUCUCCAUGGUGGGGGAUUGGCUUGAAAAAAGACCAGGAGCCUUUUGUAUAAAAGUGUUGUGCCCAUACACCAGCACUAUGAUGGAAACCCUG
KX548902    GGCGGCUGAGCCGUGUGACACGUUGCUGUGUGACAUAGGUGAGUCAUCAUCUAGUCCUGAAGUGGAAGAAGCACGGACGCUCAGAGUCCUCUCCAUGGUGGGGGAUUGGCUUGAAAAAAGACCAGGAGCCUUUUGUAUAAAAGUGUUGUGCCCAUACACCAGCACUAUGAUGGAAACCCUG
KX601166    GGCGGCUGAACCGUGUGACACUCUGCUGUGUGACAUAGGCGAGUCAUCAUCCAGUCCUGAAGUGGAAGAGACGCGAACACUCAGAGUGCUCUCCAUGGUGGGAGACUGGCUUGAAAAAAGGCCAGGGGCCUUCUGCAUAAAGGUGCUGUGCCCAUACACCAGCACAAUGAUGGAGACCAUG
KX601167    GGCGGCGGAGUCGUGUGACACUUUGCUGUGUGACAUAGGUGAGUCAUCAUCUAGUCCUGAAGUGGAAGAAGCACGGACGCUCAGAGUACUCUCCAUGGUGGGGGAUUGGCUUGAAAAAAGACCAGGGGCCUUUUGUAUAAAGGUGUUGUGCCCAUACACCAGCACCAUGAUGGAAACCCUA
KX601168    GGCGGCUGAGCCGUGUGACACGUUGCUGUGUGACAUAGGUGAGUCAUCAUCUAGUCCUGAAGUGGAAGAAGCACGGACGCUCAGAGUCCUCUCCAUGGUGGGGGAUUGGCUUGAAAAAAGACCAGGAGCCUUUUGUAUAAAAGUGUUGUGCCCAUACACCAGCACUAUGAUGGAAACCCUG
KX766028    GGCGGCUGAGCCGUGUGACACGUUGCUGUGUGACAUAGGUGAGUCAUCAUCUAGUCCUGAAGUGGAAGAAGCACGGACGCUCAGAGUCCUCUCCAUGGUGGGGGAUUGGCUUGAAAAAAGACCAGGAGCCUUUUGCAUAAAAGUGUUGUGCCCAUACACCAGCACUAUGAUGGAAACCCUG
KX766029    GGCGGCUGAGCCGUGUGACACGUUGCUGUGUGACAUAGGUGAGUCAUCAUCUAGUCCUGAAGUGGAAGAAGCACGGACGCUCAGAGUCCUCUCCAUGGUGGGGGAUUGGCUUGAAAAAAGACCAGGAGCCUUUUGUAUAAAAGUGUUGUGCCCAUACACCAGCACUAUGAUGGAAACCCUG
KU940224    GGCGGCUGAGCCGUGUGACACGUUGCUGUGUGACAUAGGUGAGUCAUCAUCUAGUCCUGAAGUGGAAGAAGCACGGACGCUCAGAGUUCUCUCCAUGGUGGGGGAUUGGCUUGAAAAAAGACCAGGAGCCUUUUGUAUAAAAGUGUUGUGCCCAUACACCAGCACUAUGAUGGAAACCCUG


            8888888888888888888888888888888888888888888888888888888888888888888888888888888888888888888888888888888888888888888888888888888888888888888888888888888888888888888888888888888
            1111111111111111111111111111111111111111111111111111112222222222222222222222222222222222222222222222222222222222222222222222222222222222222222222222222222333333333333333333333333333
            4444555555555566666666667777777777888888888899999999990000000000111111111122222222223333333333444444444455555555556666666666777777777788888888889999999999000000000011111111112222222
Sequence    6789012345678901234567890123456789012345678901234567890123456789012345678901234567890123456789012345678901234567890123456789012345678901234567890123456789012345678901234567890123456
-----------------------------------------------------------------------------------------------------------------------------------------------------------------------------------------------------------
NC_012532   GAGCGACUGCAACGUAGGCAUGGGGGAGGAUUAGUCAGAGUGCCAUUGUGUCGCAACUCCACACAUGAGAUGUACUGGGUCUCUGGGGCAAAGAGCAACAUCAUAAAAAGUGUGUCCACCACAAGUCAGCUCCUCCUGGGACGCAUGGAUGGCCCCAGGAGGCCAGUGAAAUAUGAGGAGG
KU922960    GAGCGACUGCAGCGUAGGUAUGGGGGAGGACUGGUCAGAGUGCCACUCUCCCGCAACUCUACACAUGAGAUGUACUGGGUCUCUGGAGCGAAAAGCAACACCAUAAAAAGUGUGUCCACCACGAGCCAGCUCCUCUUGGGGCGCAUGGACGGGCCUAGGAGGCCAGUGAAAUAUGAGGAGG
KU922923    GAGCGACUGCAGCGUAGGUAUGGGGGAGGACUGGUCAGAGUGCCACUCUCCCGCAACUCUACACAUGAGAUGUACUGGGUCUCUGGAGCGAAAAGCAACACCAUAAAAAGUGUGUCCACCACGAGCCAGCUCCUCUUGGGGCGCAUGGACGGGCCUAGGAGGCCAGUGAAAUAUGAGGAGG
KU820898    GAGCGACUGCAGCGUAGGUAUGGGGGAGGACUGGUCAGAGUGCCACUCUCCCGCAACUCUACACAUGAGAUGUACUGGGUCUCUGGAGCGAAAAGCAACAUCAUAAAAAGUGUGUCCACCACGAGCCAGCUCCUCUUGGGGCGCAUGGACGGGCCUAGGAGGCCAGUGAAAUAUGAGGAGG
KU321639    GAGCGACUGCAGCGUAGGUAUGGGGGAGGACUGGUCAGAGUGCCACUCUCCCGCAACUCUACACAUGAGAUGUACUGGGUCUCUGGAGCGAAAAGCAACACCAUAAAAAGUGUGUCCACCACGAGCCAGCUCCUCUUGGGGCGCAUGGACGGGCCUAGGAGGCCAGUGAAAUAUGAGGAGG
KU820899    GAGCGACUGCAGCGUAGGUAUGGAGGAGGACUGGUCAGAGUGCCACUCUCCCGCAACUCUACACAUGAGAUGUACUGGGUCUCUGGAGCGAAAAGCAACACCAUAAAAAGUGUGUCCACCACGAGCCAGCUCCUCUUGGGGCGCAUGGACGGGCCCAGGAGGCCAGUGAAAUAUGAGGAGG
KU853012    GAGCGACUGCAGCGUAGGUAUGGGGGAGGACUGGUCAGAGUGCCACUCUCCCGCAACUCUACACAUGAGAUGUACUGGGUCUCUGGAGCGAAAAGCAACACCAUAAAAAGUGUGUCCACCACGAGCCAGCUCCUCUUGGGGCGCAUGGACGGGCCUAGGAGGCCAGUGAAAUAUGAGGAGG
KU729217    GAGCGACUGCAGCGUAGGUAUGGGGGAGGACUGGUCAGAGUGCCACUCUCCCGCAACUCUACACAUGAGAUGUACUGGGUCUCUGGAGCGAAAAGCAACACCAUAAAAAGUGUGUCCACCACGAGCCAGCUCCUCUUGGGGCGCAUGGACGGGCCUAGGAGGCCAGUGAAAUAUGAGGAGG
KU729218    GAGCGACUGCAGCGUAGGUAUGGGGGAGGACUGGUCAGAGUGCCACUCUCCCGCAACUCCACACAUGAGAUGUACUGGGUCUCUGGAGCGAAAAGCAACACCAUAAAAAGUGUGUCCACCACGAGCCAGCUCCUCUUGGGGCGCAUGGACGGGCCUAGGAGGCCAGUGAAAUAUGAGGAGG
KU761564    GAGCGACUGCAGCGUAGGUAUGGGGGAGGACUGGUCAGAGUGCCACUCUCCCGCAACUCUACACAUGAGAUGUACUGGGUCUCUGGAGCGAAAAGCAACAUCAUAAAAAGUGUGUCCACCACGAGCCAGCUCCUCUUGGGGCGCAUGGACGGGCCUAGGAGGCCAGUGAAAUAUGAGGAGG
KU740184    GAGCGACUGCAGCGUAGGUAUGGGGGAGGACUGGUCAGAGUGCCACUCUCCCGCAACUCUACACAUGAGAUGUACUGGGUCUCUGGAGCGAAAAGCAACAUCAUAAAAAGUGUGUCCACCACGAGCCAGCUCCUCUUGGGGCGCAUGGACGGGCCUAGGAGGCCAGUGAAAUAUGAGGAGG
KU744693    GAGCGACUGCAGCGUAGGUAUGGGGGAGGACUGGUCAGAGUGCCACUCUCCCGCAACUCUACACAUGAGAUGUACUGGGUCUCUGGAGCGAAAAGCAACACCAUAAAAAGUGUGUCCACCACGAGCCAGCUCCUCUUGGGGCGCAUGGACGGGCCUAGGAGGCCAGUGAAAUAUGAGGAGG
KU497555    GAGCGACUGCAGCGUAGGUAUGGGGGAGGACUGGUCAGAGUGCCACUCUCCCGCAACUCUACACAUGAGAUGUACUGGGUCUCUGGAGCGAAAAGCAACACCAUAAAAAGUGUGUCCACCACGAGCCAGCUCCUCUUGGGGCGCAUGGACGGGCCUAGGAGGCCAGUGAAAUAUGAGGAGG
KU527068    GAGCGACUGCAGCGUAGGUAUGGGGGAGGACUGGUCAGAGUGCCACUCUCCCGCAACUCUACACAUGAGAUGUACUGGGUCUCUGGAGCGAAAAGCAACACCAUAAAAAGUGUGUCCACCACGAGCCAGCUCCUCUUGGGGCGCAUGGACGGGCCUAGGAGGCCAGUGAAAUAUGAGGAGG
KU501217    GAGCGACUGCAGCGUAGGUAUGGGGGAGGACUGGUCAGAGUGCCACUCUCCCGCAACUCUACACAUGAGAUGUACUGGGUCUCUGGAGCGAAAAGCAACACCAUAAAAAGUGUGUCCACCACGAGCCAGCUCCUCUUGGGGCGCAUGGACGGGCCUAGGAGGCCAGUGAAAUAUGAGGAGG
KU501216    GAGCGACUGCAGCGUAGGUAUGGGGGAGGACUGGUCAGAGUGCCACUCUCCCGCAACUCUACACAUGAGAUGUACUGGGUCUCUGGAGCGAAAAGCAACACCAUAAAAAGUGUGUCCACCACGAGCCAGCUCCUCUUGGGGCGCAUGGACGGGCCUAGGAGGCCAGUGAAAUAUGAGGAGG
KU501215    GAGCGACUGCAGCGUAGGUAUGGGGGAGGACUGGUCAGAGUGCCACUCUCCCGCAACUCUACACAUGAGAUGUACUGGGUCUCUGGAGCGAAAAGCAACACCAUAAAAAGUGUGUCCACCACGAGCCAGCUCCUCUUGGGGCGCAUGGACGGGCCUAGGAGGCCAGUGAAAUAUGAGGAGG
KU365780    GAGCGACUGCAGCGUAGGUAUGGGGGAGGACUGGUCAGAGUGCCACUCUCCCGCAACUCUACACAUGAGAUGUAUUGGGUCUCUGGAGCGAAAAGCAACACCAUAAAAAGUGUGUCCACCACGAGCCAGCUCCUCUUGGGGCGCAUGGACGGGCCUAGGAGGCCAGUGAAAUAUGAGGAGG
KU365779    GAGCGACUGCAGCGUAGGUAUGGGGGAGGACUGGUCAGAGUGCCACUCUCCCGCAACUCUACACAUGAGAUGUAUUGGGUCUCUGGAGCGAAAAGCAACACCAUAAAAAGUGUGUCCACCACGAGCCAGCUCCUCUUGGGGCGCAUGGACGGGCCUAGGAGGCCAGUGAAAUAUGAGGAGG
KU707826    GAGCGACUGCAGCGUAGGUAUGGGGGAGGACUGGUCAGAGUGCCACUCUCCCGCAACUCUACACAUGAGAUGUAUUGGGUCUCUGGAGCGAAAAGCAACACCAUAAAAAGUGUGUCCACCACGAGCCAGCUCCUCUUGGGGCGCAUGGACGGGCCUAGGAGGCCAGUGAAAUAUGAGGAGG
KU365778    GAGCGACUGCAGCGUAGGUAUGGGGGAGGACUGGUCAGAGUGCCACUCUCCCGCAACUCUACACAUGAGAUGUACUGGGUCUCUGGAGCGAAAAGCAACACCAUAAAAAGUGUGUCCACCACGAGCCAGCUCCUCUUGGGGCGCAUGGACGGGCCUAGGAGGCCAGUGAAAUAUGAGGAGG
KU365777    GAGCGACUGCAGCGUAGGUAUGGGGGAGGACUGGUCAGAGUGCCACUCUCCCGCAACUCUACACAUGAGAUGUAUUGGGUCUCUGGAGCGAAAAGCAACACCAUAAAAAGUGUGUCCACCACGAGCCAGCUCCUCUUGGGGCGCAUGGACGGGCCUAGGAGGCCAGUGAAAUAUGAGGAGG
KU312312    GAGCGACUGCAGCGUAGGUAUGGGGGAGGACUGGUCAGAGUGCCACUCUCCCGCAACUCUACACAUGAGAUGUACUGGGUCUCUGGAGCGAAAAGCAACACCAUAAAAAGUGUGUCCACCACGAGCCAGCUCCUCUUGGGGCGCAUGGACGGGCCUAGGAGGCCAGUGAAAUAUGAGGAGG
KF268950    GAGCGCCUGCAACGUAGGUAUGGGGGAGGAUUAGUCAGAGUGCCAUUGUCUCGCAACUCCACACAUGAGAUGUAUUGGGUCUCUGGAGCAAAAAGUAACGUCAUAAAAAGUGUGUCCACCACAAGUCAGCUCCUCCUGGGACGCAUGGAUGGUCCCAGGAGGCCAGUGAAAUAUGAGGAAG
KF268949    GAGCGCCUGCAACGUAGGUAUGGGGGAGGAUUAGUCAGAGUGCCAUUGUCUCGCAACUCCACACAUGAAAUGUAUUGGGUCUCUGGAGCAAAAAGUAACAUCAUAAAAAGUGUGUCCACCACAAGUCAGCUCCUCCUGGGACGCAUGGAUGGCCCAGGAGGCCAGUGAAAUAUGAGGAAG
KF268948    GAGCGCCUGCAACGUAGGUAUGGGGGAGGAUUAGUCAGAGUGCCAUUGUCUCGCAACUCCACACAUGAGAUGUAUUGGGUCUCUGGAGCAAAAAGUAACGUCAUAAAAAGUGUGUCCACCACAAGUCAGCUCCUCCUGGGACGCAUGGAUGGUCCCAGGAGGCCAGUGAAAUAUGAGGAAG
KJ776791    GAGCGACUGCAGCGUAGGUAUGGGGGAGGACUGGUCAGAGUGCCACUCUCCCGCAACUCUACACAUGAGAUGUACUGGGUCUCUGGAGCGAAAAGCAACACCAUAAAAAGUGUGUCCACCACGAGCCAGCUCCUCUUGGGGCGCAUGGACGGGCCCAGGAGGCCAGUGAAAUAUGAGGAGG
KF383119    GAGCGACUGCAACGUAGGCAUGGGGGAGGAUUAGUCAGAGUGCCAUUGUCUCGCAACUCCACACAUGAGAUGUACUGGGUCUCUGGGGCAAAAAGCAACAUCAUAAAAAGUGUGUCCACCACAAGUCAGCUCCUCCUGGGACGCAUGGAUGGCCCCAGGAGGCCAGUGAAAUAUGAGGAGG
KF383118    GAGCGACUGCAACGUAGGCAUGGGGGAGGAUUAGUCAGAGUGCCAUUGUCUCGCAACUCCACACAUGAGAUGUACUGGGUCUCUGGGGCAAAAAGCAACAUCAUAAAAAGUGUGUCCACCACAAGUCAGCUCCUCCUGGGACGCAUGGAUGGCCCCAGGAGGCCAGUGAAAUAUGAGGAGG
KF383117    GAGCGACUGCAACGUAGGCAUGGGGGAGGAUUGGUCAGAGUGCCAUUGUCCCGCAACUCCACACAUGAGAUGUAUUGGGUCUCUGGAGCCAAAAGUAACAUCAUAAAGAGUGUGUCCACCACAAGUCAGCUCCUCUUGGGACGCAUGGAUGGGCCUAGGAGGCCAGUGAAAUAUGAGGAGG
KF383116    GAGCGACUGCAACGUAGGCAUGGGGGAGGAUUGGUCAGAGUGCCAUUGUCCCGCAACUCCACACAUGAGAUGUAUUGGGUCUCUGGAGCCAAAAGUAACAUCAUAAAGAGUGUGUCCACCACAAGUCAGCUCCUCUUGGGACGCAUGGAUGGGCCUAGGAGGCCAGUGAAAUAUGAGGAGG
KF383115    GAGCGACUGCAACGUAGGUAUGGGGGAGGAUUAGUCAGAGUGCCAUUGUCUCGCAACUCCACACAUGAGAUGUAUUGGGUCUCUGGAGCAAAAAGCAACAUCAUAAAAAGUGUGUCCACCACAAGCCAGCUCCUCCUGGGACGCAUGGAUGGGCCCAGGAGGCCAGUGAAAUAUGAGGAGG
EU545988    GAGCGACUGCAGCGUAGGUAUGGGGGAGGACUGGUCAGAGUGCCACUCUCCCGCAACUCUACACAUGAGAUGUACUGGGUCUCUGGAGCGAAAAGCAACACCAUAAAAAGUGUGUCCACCACGAGCCAGCUCCUCUUGGGGCGCAUGGACGGGCCCAGGAGGCCAGUGAAAUAUGAGGAGG
KU955589    GAGCGACUGCAGCGUAGGUAUGGGGGAGGACUGGUCAGAGUGCCACUCUCCCGCAACUCUACACAUGAGAUGUACUGGGUCUCUGGAGCGAAAAGCAACACCAUAAAAAGUGUGUCCACCACGAGCCAGCUCCUCUUGGGGCGCAUGGACGGGCCCAGGAGGCCAGUGAAAUAUGAGGAGG
KU955590    GAGCGACUGCAGCGUAGGUAUGGGGGAGGACUGGUCAGAGUGCCACUCUCCCGCAACUCUACACAUGAGAUGUACUGGGUCUCUGGAGCGAAAAGCAACACCAUAAAAAGUGUGUCCACCACGAGCCAGCUCCUCUUGGGGCGCAUGGACGGGCCUAGGAGGCCAGUGAAAUAUGAGGAGG
KU940228    GAGCGACUGCAGCGUAGGUAUGGGGGAGGACUGGUCAGAGUGCCACUCUCCCGCAACUCUACACAUGAGAUGUACUGGGUCUCUGGAGCGAAAAGCAACACCAUAAAAAGUGUGUCCACCACGAGCCAGCUCCUCUUGGGGCGCAUGGACGGGCCUAGGAGGCCAGUGAAAUAUGAGGAGG
KU991811    GAGCGACUGCAGCGUAGGUAUGGGGGAGGACUGGUCAGAGUGCCACUCUCCCGCAACUCUACACAUGAGAUGUACUGGGUCUCUGGAGCGAAAAGCAACACCAUAAAAAGUGUGUCCACCACGAGCCAGCUCCUCUUGGGGCGCAUGGACGGGCCUAGGAGGCCAGUGAAAUAUGAGGAGG
KU509998    GAGCGACUGCAGCGUAGGUAUGGGGGAGGACUGGUCAGAGUGCCACUCUCCCGCAACUCUACACAUGAGAUGUACUGGGUCUCUGGAGCGAAAAGCAACACCAUAAAAAGUGUGUCCACCACGAGCCAGCUCCUCUUGGGGCGCAUGGACGGGCCUAGGAGGCCAGUGAAAUAUGAGGAGG
KX056898    GAGCGACUGCAGCGUAGGUAUGGGGGAGGACUGGUCAGAGUGCCACUCUCCCGCAACUCUACACAUGAGAUGUACUGGGUCUCUGGAGCGAAAAGCAACACCAUAAAAAGUGUGUCCACCACGAGCCAGCUCCUCUUGGGGCGCAUGGACGGGCCUAGGAGGCCAGUGAAAUAUGAGGAGG
KX117076    GAGCGACUGCAGCGUAGGUAUGGGGGAGGACUGGUCAGAGUGCCACUCUCCCGCAACUCUACACAUGAGAUGUACUGGGUCUCUGGAGCGAAAAGCAACACCAUAAAAAGUGUGUCCACCACGAGCCAGCUCCUCUUGGGGCGCAUGGACGGGCCCAGGAGGCCAGUGAAAUAUGAGGAGG
```

```
KX185891    GAGCGACUGCAGCGUAGGUAUGGGGGAGGACUGGUCAGAGUGCCACUCUCCCGCAACUCUACACAUGAGAUGUACUGGGUCUCUGGAGCGAAAAGCAACACCAUAAAAAGUGUGUCCACCACGAGCCAGCUCCUCUUGGGGCGCAUGGACGGGCCCAGGAGGCCAGUGAAAUAUGAGGAGG
KX247646    GAGCGACUGCAGCGUAGGUAUGGGGGAGGACUGGUCAGAGUGCCACUCUCCCGCAACUCUACACAUGAGAUGUACUGGGUCUCUGGAGCGAAAAGCAACACCAUAAAAAGUGUGUCCACCACGAGCCAGCUCCUCUUGGGGCGCAUGGACGGGCCUAGGAGGCCAGUGAAAUAUGAGGAGG
KX262887    GAGCGACUGCAGCGUAGGUAUGGGGGAGGACUGGUCAGAGUGCCACUCUCCCGCAACUCUACACAUGAGAUGUACUGGGUCUCUGGAGCGAAAAGCAACACCAUAAAAAGUGUGUCCACCACGAGCCAGCUCCUCUUGGGGCGCAUGGACGGGCCUAGGAGGCCAGUGAAAUAUGAGGAGG
KX280026    GAGCGACUGCAGCGUAGGUAUGGGGGAGGACUGGUCAGAGUGCCACUCUCCCGCAACUCUACACAUGAGAUGUACUGGGUCUCUGGAGCGAAAAGCAACACCAUAAAAAGUGUGUCCACCACGAGCCAGCUCCUCUUGGGGCGCAUGGACGGGCCAAGGAGGCCAGUGAAAUAUGAGGAGG
KU758877    GAGCGACUGCAGCGUAGGUAUGGGGGAGGACUGGUCAGAGUGCCACUCUCCCGCAACUCUACACAUGAGAUGUACUGGGUCUCUGGAGCGAAAAGCAACACCAUAAAAAGUGUGUCCACCACGAGCCAGCUCCUCUUGGGGCGCAUGGACGGGCCUAGGAGGCCAGUGAAAUAUGAGGAGG
KU866423    GAGCGACUGCAGCGUAGGUAUGGGGGAGGACUGGUCAGAGUGCCACUCUCCCGCAACUCUACACAUGAGAUGUACUGGGUCUCUGGAGCGAAAAGCAACACCAUAAAAAGUGUGUCCACCACGAGCCAGCUCCUCUUGGGGCGCAUGGACGGGCCCAGGAGGCCAGUGAAAUAUGAGGAGG
KX377336    GAGCGACUGCAGCGUAGGUAUGGGGGAGGACUGGUCAGAGUGCCACUCUCCCGCAACUCUACACAUGAGAUGUACUGGGUCUCUGGAGCGAAAAGCAACAUCAUAAAAAGUGUGUCCACCACGAGCCAGCUCCUCUUGGGACGCAUGGACGGGCCCAGGAGGCCAGUGAAAUAUGAGGAGG
KX377337    GAGCGACUGCAGCGUAGGUAUGGGGGAGGAUUGGUCAGAGUGCCACUCUCCCGCAACUCUACACAUGAGAUGUACUGGGUCUCUGGAGCGAAAAGCAACACCAUAAAAAGUGUGUCCACCACGAGCCAGCUCCUCUUGGGGCGCAUGGACGGGCCUAGGAGGCCAGUGAAAUAUGAGGAGG
KX369547    GAGCGACUGCAGCGUAGGUAUGGGGGAGGACUGGUCAGAGUGCCACUCUCCCGCAACUCUACACAUGAGAUGUACUGGGUCUCUGGAGCGAAAAGCAACACCAUAAAAAGUGUGUCCACCACGAGCCAGCUCCUCUUGGGGCGCAUGGACGGGCCCAGGAGGCCAGUGAAAUAUGAGGAGG
KX548902    GAGCGACUGCAGCGUAGGUAUGGGGGAGGACUGGUCAGAGUGCCACUCUCCCGCAACUCUACACAUGAGAUGUACUGGGUCUCUGGAGCGAAAAGCAACACCAUAAAAAGUGUGUCCACCACGAGCCAGCUCCUCUUGGGGCGCAUGGACGGGCCUAGGAGGCCAGUGAAAUAUGAGGAGG
KX601166    GAGCGACUGCAACGUAGGCAUGGGGGAGGAUUAGUCAGAGUGCCAUUGUCCCGCAACUCUACACAUGAGAUGUAUUGGGUCUCUGGAGCCAAAAGUAACAUCAUAAAGAGUGUGUCCACCACAAGUCAGCUCCUCUUGGGACGCAUGGAAGGGCCUAGGAGGCCAGUGAAAUAUGAGGAGG
KX601167    GAGCGACUGCAGCGUAGGUAUGGGGGAGGACUGGUCAGAGUGCCACUCUCCCGCAACUCUACACAUGAGAUGUACUGGGUCUCUGGAGCGAAAAGCAACAUCAUAAAAAGUGUGUCCACCACGAGCCAGCUCCUCUUGGGACGCAUGGACGGGCCCAGGAGGCCAGUGAAAUAUGAGGAGG
KX601168    GAGCGACUGCAGCGUAGGUAUGGGGGAGGACUGGUCAGAGUGCCACUCUCCCGCAACUCUACACAUGAGAUGUACUGGGUCUCUGGAGCGAAAAGCAACACCAUAAAAAGUGUGUCCACCACGAGCCAGCUCCUCUUGGGGCGCAUGGACGGGCCUAGGAGGCCAGUGAAAUAUGAGGAGG
KX766028    GAGCGACUGCAGCGUAGGUAUGGGGGAGGACUGGUCAGAGUGCCACUCUCCCGCAACUCUACACAUGAGAUGUACUGGGUCUCUGGAGCGAAAAGCAACACCAUAAAAAGUGUGUCCACCACGAGCCAGCUCCUCUUGGGGCGCAUGGACGGGCCUAGGAGGCCAGUGAAAUAUGAGGAGG
KX766029    GAGCGACUGCAGCGUAGGUAUGGGGGAGGACUGGUCAGAGUGCCACUAUCCCGCAACUCUACACAUGAGAUGUACUGGGUCUCUGGAGCGAAAAGCAACACCAUAAAAAGUGUGUCCACCACGAGCCAGCUCCUCUUGGGGCGCAUGGACGGGCCUAGGAGGCCAGUGAAAUAUGAGGAGG
KU940224    GAGCGACUGCAGCGUAGGUAUGGGGGAGGACUGGUCAGAGUGCCACUCUCCCGCAACUCUACACAUGAGAUGUACUGGGUCUCUGGAGCGAAAAGCAACACCAUAAAAAGUGUGUCCACCACGAGCCAGCUCCUCUUGGGGCGCAUGGACGGGCCUAGGAGGCCAGUGAAAUAUGAGGAGG


            8888888888888888888888888888888888888888888888888888888888888888888888888888888888888888888888888888888888888888888888888888888888888888888888888888888888888888888888888888888
            3333333333333333333333333333333333333333333333333333333333333333333333333444444444444444444444444444444444444444444444444444444444444444444444444444444444444444444444444455555555
            2223333333333444444444455555555556666666666777777777788888888889999999999000000000011111111112222222222333333333344444444445555555555666666666677777777778888888888999999999900000000
Sequence    7890123456789012345678901234567890123456789012345678901234567890123456789012345678901234567890123456789012345678901234567890123456789012345678901234567890123456789012345678901234567
--------------------------------------------------------------------------------------------------------------------------------------------------------------------------------------------------------
NC_012532   AUGUGAACCUCGGCUCGGGUACACGAGCUGUGGCAAGCUGUGCUGAGGCUCCUAACAUGAAAAUCAUCGGCAGGCGCAUUGAGAGAAUCCGCAAUGAACAUGCAGAAACAUGGUUUCUUGAUGAAAACCACCCAUACAGGACAUGGGCCUACCAUGGGAGCUACGAAGCCCCCACGCAAGG
KU922960    AUGUGAAUCUCGGCUCUGGCACGCGGGCUGUGGUAAGCUGCGCUGAAGCUCCCAACAUGAAGAUCAUUGGUAACCGCAUUGAAAGGAUCCGCAGUGAGCAAGCGGAAACGUGGUUCUUUGACGAGAACCACCCAUAUAGGACAUGGGCUUACCAUGGAAGCUAUGAGGCCCCCACACAAGG
KU922923    AUGUGAAUCUCGGCUCUGGCACGCGGGCUGUGGUAAGCUGCGCUGAAGCUCCCAACAUGAAGAUCAUUGGUAACCGCAUUGAAAGGAUCCGCAGUGAGCAAGCGGAAACGUGGUUCUUUGACGAGAACCACCCAUAUAGGACAUGGGCUUACCAUGGAAGCUAUGAGGCCCCCACACAAGG
KU820898    AUGUGAAUCUCGGCUCUGGCACGCGGGCUGUGGCAAGCUGCGCUGAAGCUCCCAACAUGAAGAUCAUUGGUAACCGCAUUGAAAGGAUCCGCAGUGAGCACGCGGAAACGUGGUUCUUUGACGAGAACCACCCAUAUAGGACAUGGGCUUACCAUGGAAGCUAUGAGGCCCCCACACAAGG
KU321639    AUGUGAAUCUCGGCUCUGGCACGCGGGCUGUGGUAAGCUGCGCUGAAGCUCCCAACAUGAAGAUCAUUGGUAACCGCAUUGAAAGGAUCCGCAGUGAGCACGCGGAAACGUGGUUCUUUGACGAGAACCACCCAUAUAGGACAUGGGCUUACCAUGGAAGCUAUGAGGCCCCCACACAAGG
KU820899    AUGUGAAUCUCGGCUCUGGCACGCGGGCUGUGGUAAGCUGCGCUGAAGCUCCCAACAUGAAGAUCAUUGGUAACCGCAUUGAAAGGAUCCGCAGUGAGCACGCGGAAACGUGGUUCUUUGACGAGAACCACCCAUAUAGGACAUGGGCUUACCAUGGAAGCUAUGAGGCCCCCACACAAGG
KU853012    AUGUGAAUCUCGGCUCUGGCACGCGGGCUGUGGUAAGCUGCGCUGAAGCUCCCAACAUGAAGAUCAUUGGUAACCGCAUUGAAAGGAUCCGCAGUGAGCACGCGGAAACGUGGUUCUUUGACGAGAACCACCCAUAUAGGACAUGGGCUUACCAUGGAAGCUAUGAGGCCCCCACACAAGG
KU729217    AUGUGAAUCUCGGCUCUGGCACGCGGGCUGUGGUAAGCUGCGCUGAAGCUCCCAACAUGAAGAUCAUUGGUGACCGCAUUGAAAGGAUCCGCAGUGAGCACGCGGAAACGUGGUUCUUUGACGAGAACCACCCAUAUAGGACAUGGGCUUACCAUGGAAGCUAUGAGGCCCCCACACAAGG
KU729218    AUGUGAAUCUCGGCUCUGGCACGCGGGCUGUGGUAAGCUGCGCUGAAGCUCCCAACAUGAAGAUCAUUGGUAACCGCAUUGAAAGGAUCCGCAGUGAGCACGCGGAAACGUGGUUCUUUGACGAGAACCACCCAUAUAGGACAUGGGCUUACCAUGGAAGCUAUGAGGCCCCCACACAAGG
KU761564    AUGUGAAUCUCGGCUCUGGCACGCGGGCUGUGGCAAGCUGCGCUGAAGCUCCCAACAUGAAGAUCAUUGGUAACCGCAUUGAAAGGAUCCGCAGUGAGCACGCGGAAACGUGGUUCUUUGACGAGAACCACCCAUAUAGGACAUGGGCUUACCAUGGAAGCUAUGAGGCCCCCACACAAGG
KU740184    AUGUGAAUCUCGGCUCUGGCACGCGGGCUGUGGCAAGCUGCGCUGAAGCUCCCAACAUGAAGAUCAUUGGUAACCGCAUUGAAAGGAUCCGCAGUGAGCACGCGGAAACGUGGUUCUUUGACGAGAACCACCCAUAUAGGACAUGGGCUUACCAUGGAAGCUAUGAGGCCCCCACACAAGG
KU744693    AUGUGAAUCUCGGCUCUGGCACGCGGGCUGUGGUAAGCUGCGCUGAAGCUCCCAACAUGAAGAUCAUUGGUAACCGCAUUGAAAGGAUCCGCGCUGAGAAAGCGGAAACGUGGUUCUUUGACGAGAACCACCCAUAUAGGACAUGGGCUUACCAUGGAAGCUAUGAUGCCGCCACACAAGG
KU497555    AUGUGAAUCUCGGCUCUGGCACGCGGGCUGUGGUAAGCUGCGCUGAAGCUCCCAACAUGAAGAUCAUUGGUAACCGCAUUGAAAGGAUCCGCAGUGAGCACGCGGAAACGUGGUUCUUUGACGAAAACCACCCAUAUAGGACAUGGGCUUACCAUGGAAGCUAUGUGGCCCCCACACAAGG
KU527068    AUGUGAAUCUCGGCUCUGGCACGCGGGCUGUGGUAAGCUGCGCUGAAGCUCCCAACAUGAAGAUCAUUGGUAACCGCAUUGAAAGGAUCCGCAGUGAGCACGCGGAAACGUGGUUCUUUGACGAGAACCACCCAUAUAGGACAUGGGCUUACCAUGGAAGCUAUGAGGCCCCCACACAAGG
KU501217    AUGUGAAUCUCGGCUCUGGCACGCGGGCUGUGGUAAGCUGCGCUGAAGCUCCCAACAUGAAGAUCAUUGGUAACCGCAUUGAAAGGAUCCGCAGUGAGCACGCGGAAACGUGGUUCUUUGACGAGAACCACCCAUAUAGGACAUGGGCUUACCAUGGAAGCUAUGAGGCCCCCACACAAGG
KU501216    AUGUGAAUCUCGGCUCUGGCACGCGGGCUGUGGUAAGCUGCGCUGAAGCUCCCAACAUGAAGAUCAUUGGUAACCGCAUUGAAAGGAUCCGCAGUGAGCACGCGGAAACGUGGUUCUUUGACGAGAACCACCCAUAUAGGACAUGGGCUUACCAUGGAAGCUAUGAGGCCCCCACACAAGG
KU501215    AUGUGAAUCUCGGCUCUGGCACGCGGGCUGUGGUAAGCUGCGCUGAAGCUCCCAACAUGAAGAUCAUUGGUAACCGCAUUGAAAGGAUCCGCAGUGAGCACGCGGAAACGUGGUUCUUUGACGAGAACCACCCAUAUAGGACAUGGGCUUACCAUGGAAGCUAUGAGGCCCCCACACAAGG
KU365780    AUGUGGAUCUCGGCUCUGGCACGCGGGCUGUGGUAAGCUGCGCUGAAGCUCCCAACAUGAAGAUCAUUGGUAACCGCAUUGAAAGGAUCCGCAGUGAGCACGCGGAAACGUGGUUCUUUGACGAGAACCACCCAUAUAGGACAUGGGCUUACCAUGGAAGCUAUGAGGCCCCCACACAAGG
KU365779    AUGUGAAUCUCGGCUCUGGCACGCGGGCUGUGGUAAGCUGCGCUGAAGCUCCCAACAUGAAGAUCAUUGGUAACCGCAUUGAAAGGAUCCGCAGUGAGCACGCGGAAACGUGGUUCUUUGACGAGAACCACCCAUAUAGGACAUGGGCUUACCAUGGAAGCUAUGAGGCCCCCACACAAGG
KU707826    AUGUGAAUCUCGGCUCUGGCACGCGGGCUGUGGUAAGCUGCGCUGAAGCUCCCAACAUGAAGAUCAUUGGUAACCGCAUUGAAAGGAUCCGCAGUGAGCACGCGGAAACGUGGUUCUUUGACGAGAACCACCCAUAUAGGACAUGGGCUUACCAUGGAAGCUAUGAGGCCCCCACACAAGG
KU365778    AUGUGAAUCUCGGCUCUGGCACGCGGGCUGUGGUAAGCUGCGCUGAAGCUCCCAACAUGAAGAUCAUUGGUAACCGCAUUGAAAGGAUCCGCAGUGAGCACGCGGAAACGUGGUUCUUUGACGAGAACCACCCAUAUAGGACAUGGGCUUACCAUGGAAGCUAUGAGGCCCCCACACAAGG
KU365777    AUGUGGAUCUCGGCUCUGGCACGCGGGCUGUGGUAAGCUGCGCUGAAGCUCCCAACAUGAAGAUCAUUGGUAACCGCAUUGAAAGGAUCCGCAGUGAGCACGCGGAAACGUGGUUCUUUGACGAGAACCACCCAUAUAGGACAUGGGCUUACCAUGGAAGCUAUGAGGCCCCCACACAAGG
KU312312    AUGUGAAUCUCGGCUCUGGCACGCGGGCUGUGGUAAGCUGCGCUGAAGCUCCCAACAUGAAGAUCAUUGGUAACCGCAUUGAAAGGAUCCGCAGUGAGCACGCGGAAACGUGGUUCUUUGACGAGAACCACCCAUAUAGGACAUGGGCUUACCAUGGAAGCUAUGAGGCCCCCACACAAGG
KF268950    AUGUGAACCUCGGCUCGGGCACACGAGCUGUGGCAAGCUGUGCUGAAGCUCCCAACAUGAAGGUCAUUGGCAAGCGCAUUGAGAGAAUCCGCAGUGAACAUGCAGAAACAUGGUUCUUUGAUGAAAACCAUCCAUACAGGACAUGGGCCUACCAUGGGAGCUACGAAGCCCCCACACAAGG
KF268949    AUGUGAACCUCGGCUCGGGCACACGAGCUGUGGCAAGCUGUGCUGAAGCUCCCAACAUGAAGAUCAUUGGCAAGCGCAUUGAGAGAAUCCGCAGUGAACAUGCAGAAACAUGGUUCUUUGAUGAAAACCACCCAUACAGGACAUGGGCCUACCAUGGGAGCUACGAAGCCCCCACCCAAGG
KF268948    AUGUGAACCUCGGCUCGGGCACACGAGCUGUGGCAAGCUGUGCUGAAGCUCCCAACAUGAAGGUCAUUGGCAAGCGCAUUGAGAGAAUCCGCAGUGAACAUGCAGAAACAUGGUUCUUUGAUGAAAACCAUCCAUACAGGACAUGGGCCUACCAUGGGAGCUACGAAGCCCCCACACAAGG
KJ776791    AUGUGAAUCUCGGCUCUGGCACGCGGGCUGUGGUAAGCUGCGCUGAAGCUCCCAACAUGAAGAUCAUUGGUAACCGCAUUGAAAGGAUCCGCAGUGAGCACGCGGAAACGUGGUUCUUUGACGAGAACCACCCAUAUAGGACAUGGGCUUACCAUGGAAGCUAUGAGGCCCCCACACAAGG
KF383119    AUGUGAACCUCGGCUCGGGUACACGAGCUGUGGCAAGCUGUGCUGAGGCUCCUAACAUGAAAAUCAUCGGCAGGCGCAUUGAGAGAAUCCGCAAUGAACAUGCAGAAACAUGGUUUCUUGAUGAAAACCACCCAUACAGGACAUGGGCCUACCAUGGGAGCUACGAAGCCCCCACGCAAGG
KF383118    AUGUGAACCUCGGCUCGGGUACACGAGCUGUGGCAAGCUGUGCUGAGGCUCCUAACAUGAAAAUCAUCGGCAGGCGCAUUGAGAGAAUCCGCAAUGAACAUGCAGAAACAUGGUUUCUUGAUGAAAACCACCCAUACAGGACAUGGGCCUACCAUGGGAG?UACGAAGCCCCCACGCAAGG
KF383117    AUGUGAACCUCGGCUCAGGCACACGAGCUGUGGCAAGCUGCGCUGAGGCUCCCAACAUGAAGAUCAUUGGUAGGCGCAUUGAGAGAAUCCGCAAUGAACAUGCAGAGACAUGGUUCUUUGAUGAAAACCACCCAUACAGGACAUGGGCCUACCAUGGGAGCUACGAAGCCCCCACGCAGGG
KF383116    AUGUGAACCUCGGCUCAGGCACACGAGCUGUGGCAAGCUGCGCUGAGGCUCCCAACAUGAAGAUCAUUGGUAGGCGCAUUGAGAGAAUCCGCAAUGAACAUGCAGAGACAUGGUUCUUUGAUGAAAACCACCCAUACAGGACAUGGGCCUACCAUGGGAGCUACGAAGCCCCCACGCAGGG
KF383115    AUGUGAACCUCGGCUCGGGCACACGAGCUGUGGCAAGCUGUGCUGAGGCUCCCAACAUGAAGAUCAUUGGUAAGCGCAUUGAGAGAAUCCGCAGUGAACAUGCAGAAACAUGGUUCUUUGAUGAAAACCACCCAUACAGGACAUGGGCUUACCAUGGGAGCUACGAAGCCCCCACGCAAGG
EU545988    AUGUGAAUCUCGGCUCCGGCACGCGGGCUGUGGUAAGCUGCGCUGAAGCUCCCAACAUGAAGAUCAUUGGUAACCGCAUUGAGAGGAUCCGCAGUGAGCACGCGGAAACGUGGUUCUUUGACGAGAACCACCCAUAUAGGACAUGGGCUUACCAUGGAAGCUAUGAGGCCCCUACACAAGG
KU955589    AUGUGAAUCUCGGCUCUGGCACGCGGGCUGUGGUAAGCUGCGCUGAAGCUCCCAACAUGAAGAUCAUUGGUAACCGCAUUGAAAGGAUCCGCAGUGAGCACGCGGAAACGUGGUUCUUUGACGAGAACCACCCAUAUAGGACAUGGGCUUACCAUGGAAGCUAUGAGGCCCCCACACAAGG
KU955590    AUGUGAAUCUCGGCUCUGGCACGCGGGCUGUGGCAAGCUGCGCUGAAGCUCCCAACAUGAAGAUCAUUGGUAACCGCAUUGAAAGGAUCCGCAGUGAGCACGCGGAAACGUGGUUCUUUGACGAGAACCACCCAUAUAGGACAUGGGCUUACCAUGGAAGCUAUGAGGCCCCCACACAAGG
KU940228    AUGUGAAUCUCGGCUCUGGCACGCGGGCUGUGGUAAGCUGCGCUGAAGCUCCCAACAUGAAGAUCAUUGGUAACCGCAUUGAAAGGAUCCGCAGUGAGCACGCGGAAACGUGGUUCUUUGACGAGAACCACCCAUAUAGGACAUGGGCUUACCAUGGAAGCUAUGAGGCCCCCACACAAGG
KU991811    AUGUGAAUCUCGGCUCUGGCACGCGGGCUGUGGUAAGCUGCGCUGAAGCUCCCAAUAUGAAGAUCAUUGGUAACCGCAUUGAAAGGAUCCGCAGUGAGCACGCGGAAACGUGGUUCUUUGACGAGAACCACCCAUAUAGGACAUGGGCUUACCAUGGAAGCUAUGAGGCCCCCACACAAGG
KU509998    AUGUGAAUCUCGGCUCUGGCACGCGGGCUGUGGUAAGCUGCGCUGAAGCUCCCAACAUGAAGAUCAUUGGUAACCGCAUUGAAAGGAUCCGCAGUGAGCACGCGGAAACGUGGUUCUUUGACGAGAACCACCCAUAUAGGACAUGGGCUUACCAUGGAAGCUAUGAGGCCCCCACACAAGG
KX056898    AUGUGAAUCUCGGCUCUGGCACGCGGGCUGUGGCAAGCUGCGCUGAAGCUCCCAACAUGAAGAUCAUUGGUAACCGCAUUGAAAGGAUCCGCAGUGAGCACGCGGAAACGUGGUUCUUUGACGAGAACCACCCAUAUAGGACAUGGGCUUACCAUGGAAGCUAUGAGGCCCCCACACAAGG
KX117076    AUGUGAAUCUCGGCUCUGGCACGCGGGCUGUGGCAAGCUGCGCUGAAGCUCCCAACAUGAAGAUCAUUGGUAACCGCAUUGAAAGGAUCCGCAGUGAGCACGCGGAAACGUGGUUCUUUGACGAGAACCACCCAUAUAGGACAUGGGCUUACCAUGGAAGCUAUGAGGCCCCCACACAAGG
KX185891    AUGUGAAUCUCGGCUCUGGCACGCGGGCUGUGGUAAGCUGCGCUGAAGCUCCCAACAUGAAGAUCAUUGGUAACCGCAUUGAAAGGAUCCGCAGUGAGCACGCGGAAACGUGGUUCUUUGACGAGAACCACCCAUAUAGGACAUGGGCUUACCAUGGAAGCUAUGAGGCCCCCACACAAGG
KX247646    AUGUGAAUCUCGGCUCUGGCACGCGGGCUGUGGUAAGCUGCGCUGAAGCUCCCAACAUGAAGAUCAUUGGUAACCGCAUUGAAAGGAUCCGCAGUGAGCACGCGGAAACGUGGUUCUUUGACGAGAACCACCCAUAUAGGACAUGGGCUUACCAUGGAAGCUAUGAGGCCCCCACACAAGG
KX262887    AUGUGAAUCUCGGCUCUGGCACGCGGGCUGUGGUAAGCUGCGCUGAAGCUCCCAACAUGAAGAUCAUUGGUAACCGCAUUGAAAGGAUCCGCAGUGAGCACGCGGAAACGUGGUUCUUUGACGAGAACCACCCAUAUAGGACAUGGGCUUACCAUGGAAGCUAUGAGGCCCCCACACAAGG
KX280026    AUGUGAAUCUCGGCUCUGGCACGCGGGCUGUGGUAAGCUGCGCUGAAGCUCCCAACAUGAAGAUCAUUGGUAACCGCAUUGAAAGGAUCCGCAGUGAGCACGCGGAAACGUGGUUCUUUGACGAGAACCACCCAUAUAGGACAUGGGCUUACCAUGGAAGCUAUGAGGCCCCCACACAAGG
KU758877    AUGUGAAUCUCGGCUCUGGCACGCGGGCUGUGGUAAGCUGCGCUGAAGCUCCCAACAUGAAGAUCAUUGGUAACCGCAUUGAAAGGAUCCGCAGUGAGCACGCGGAAACGUGGUUCUUUGACGAGAACCACCCAUAUAGGACAUGGGCUUACCAUGGAAGCUAUGAGGCCCCCACACAAGG
KU866423    AUGUGAAUCUCGGCUCUGGCACGCGGGCUGUGGUAAGCUGCGCUGAAGCUCCCAACAUGAAGAUCAUUGGUAACCGCAUUGAAAGGAUCCGCAGUGAGCACGCGGAAACGUGGUUCUUUGACGAGAACCACCCAUAUAGGACAUGGGCUUACCAUGGAAGCUAUGAGGCCCCCACACAAGG
KX377336    AUGUGAAUCUCGGCUCCGGCACGCGAGCUGUGGCAAGCUGCGCCGAAGCUCCCAACCUGAAGAUCAUUGGUAACCGCGUUGAGAGGAUCCGCAGUGAGCAUGCGGAAACGUGGUUCUUUGAUGAGAACCACCCAUACAGGACAUGGGCUUACCAUGGGAGCUACGAGGCCCCUACACAAGG
KX377337    AUGUGAAUCUCGGCUCUGGCACGCGGGCUGUGGUAAGCUGCGCUGAAGCUCCCAACAUGAAGAUCAUUGGUAACCGCAUUGAAAGGAUCCGCAGUGAGCACGCGGAAACGUGGUUCUUUGACGAGAACCACCCAUAUAGGACAUGGGCUUACCAUGGAAGCUAUGAGGCCCCCACACAAGG
KX369547    AUGUGAAUCUCGGCUCUGGCACGCGGGCUGUGGUAAGCUGCGCUGAAGCUCCCAACAUGAAGAUCAUUGGUAACCGCAUUGAAAGGAUCCGCAGUGAGCACGCGGAAACGUGGUUCUUUGACGAGAACCACCCAUAUAGGACAUGGGCUUACCAUGGAAGCUAUGAGGCCCCCACACAAGG
KX548902    AUGUGAAUCUCGGCUCUGGCACGCGGGCUGUGGUAAGCUGCGCUGAAGCUCCCAACAUGAAGAUCAUUGGUAACCGCAUUGAAAGGAUCCGCAGUGAGCACGCGGAAACGUGGUUCUUUGACGAGAACCACCCAUAUAGGACAUGGGCUUACCAUGGAAGCUAUGAGGCCCCCACACAAGG
KX601166    AUGUGAACCUCGGCUCAGGCACACGAGCUGUGGCAAGCUGCGCUGAGGCUCCCAACAUGAAGAUCAUUGGUAGGCGCAUUGAGAGAAUCCGCAAUGAACAUGCAGAGACAUGGUUCUUUGAUGAAAACCACCCAUACAGGACAUGGGCCUACCAUGGGAGCUACGAAGCCCCCACGCAGGG
KX601167    AUGUGAAUCUCGGCUCCGGCACGCGAGCUGUGGCAAGCUGCGCCGAAGCUCCCAACCUGAAGAUCAUUGGUAACCGCGUUGAGAGGAUCCGCAGUGAGCAUGCGGAAACGUGGUUCUUUGAUGAGAACCACCCAUACAGGACAUGGGCUUACCAUGGGAGCUACGAGGCCCCUACACAAGG
KX601168    AUGUGAAUCUCGGCUCUGGCACGCGGGCUGUGGUAAGCUGCGCUGAAGCUCCCAACAUGAAGAUCAUUGGUAACCGCAUUGAAAGGAUCCGCAGUGAGCACGCGGAAACGUGGUUCUUUGACGAGAACCACCCAUAUAGGACAUGGGCUUACCAUGGAAGCUAUGAGGCCCCCACACAAGG
KX766028    AUGUGAAUCUCGGCUCUGGCACGCGGGCUGUGGCAAGCUGCGCUGAAGCUCCCAACAUGAAGAUCAUUGGUAACCGCAUUGAAAGGAUCCGCAGUGAGCACGCGGAAACGUGGUUCUUUGACGAGAACCACCCAUAUAGGACAUGGGCUUACCAUGGAAGCUAUGAGGCCCCCACACAAGG
KX766029    AUGUGAAUCUCGGCUCUGGCACGCGGGCUGUGGUAAGCUGCGCUGAAGCUCCCAACAUGAAGAUCAUUGGUAACCGCAUUGAAAGGAUCCGCAGUGAGCACGCGGAAACGUGGUUCUUUGACGAGAACCACCCAUAUAGGACAUGGGCUUACCAUGGAAGCUAUGAGGCCCCCACACAAGG
```

```
KU940224    AUGUGAAUCUCGGCUCUGGCACGCGGGCUGUGGUAAGCUGCGCUGAAGCUCCCAACAUGAAGAUCAUUGGUAACCGCAUUGAAAGGAUCCGCAGUGAGCACGCGGAAACGUGGUUCUUUGACGAGAACCACCCAUAUAGGACAUGGGCUUACCAUGGAAGCUAUGAGGCCCCCACACAAGG


            8888888888888888888888888888888888888888888888888888888888888888888888888888888888888888888888888888888888888888888888888888888888888888888888888888888888888888888888888888888
            5555555555555555555555555555555555555555555555555555555555555555555555555555555555555555555555556666666666666666666666666666666666666666666666666666666666666666666666666666666666666666
            0011111111112222222222333333333344444444445555555555666666666677777777778888888888999999999900000000001111111111222222222233333333334444444444555555555566666666667777777777888888888
Sequence    8901234567890123456789012345678901234567890123456789012345678901234567890123456789012345678901234567890123456789012345678901234567890123456789012345678901234567890123456789012345678
-------------------------------------------------------------------------------------------------------------------------------------------------------------------------------------------------
NC_012532   AUCAGCGUCUUCCCUCGUGAACGGGGUUGUUAGACUCCUGUCAAAGCCUUGGGACGUGGUGACUGGAGUUACAGGAAUAGCCAUGACUGACACCACACCAUACGGCCAACAAAGAGUCUUCAAAGAAAAAGUGGACACCAGGGUGCCAGAUCCCCAAGAAGGCACUCGCCAGGUAAUGAAC
KU922960    GUCAGCGUCCUCUCUAAUAAACGGGGUUGUCAGGCUCCUGUCAAAACCCUGGGAUGUGGUGACUGGAGUCACAGGAAUAGCCAUGACCGACACCACACCGUAUGGUCAGCAAAGAGUUUUCAAGGAAAAAGUGGACACUAGGGUGCCAGACCCCCAAGAAGGCACUCGUCAGGUUAUGAGC
KU922923    GUCAGCGUCCUCUCUAAUAAACGGGGUUGUCAGGCUCCUGUCAAAACCCUGGGAUGUGGUGACUGGAGUCACAGGAAUAGCCAUGACCGACACCACACCGUAUGGUCAGCAAAGAGUUUUCAAGGAAAAAGUGGACACUAGGGUGCCAGACCCCCAAGAAGGCACUCGUCAGGUUAUGAGC
KU820898    GUCAGCGUCCUCUCUAAUAAACGGGGUUGUCAGGCUCCUGUCAAAACCCUGGGAUGUGGUGACUGGAGUCACAGGAAUAGCCAUGACCGACACCACACCGUAUGGUCAGCAAAGAGUUUUCAAGGAAAAAGUGGACACUAGGGUGCCAGACCCCCAAGAAGGCACUCGUCAGGUUAUGAGC
KU321639    GUCAGCGUCCUCUCUAAUAAACGGGGUUGUCAGGCUCCUGUCAAAACCCUGGGAUGUGGUGACUGGAGUCACAGGAAUAGCCAUGACCGACACCACACCGUAUGGUCAGCAAAGAGUUUUCAAGGAAAAAGUGGACACUAGGGUGCCAGACCCCCAAGAAGGUACUCGUCAGGUUAUGAGC
KU820899    GUCAGCGUCCUCUCUAAUAAACGGGGUUGUCAGGCUCCUGUCAAAACCCUGGGAUGUGGUGACUGGAGUCACAGGAAUAGCCAUGACCGACACCACACCGUAUGGUCAGCAAAGAGUUUUCAAGGAAAAAGUGGACACUAGGGUGCCAGAUCCCCAAGAAGGCACUCGUCAGGUUAUGAGC
KU853012    GUCAGCAUCCUCUCUAGUAAACGGGGUUGUCAGGCUCCUGUCAAAACCCUGGGAUGUGGUGACUGGAGUCACAGGAAUAGCCAUGACCGACACCACACCGUAUGGUCAGCAAAGAGUUUUCAAGGAAAAAGUGGACACUAGGGUGCCAGACCCCCAAGAAGGCACUCGUCAGGUUAUGAGC
KU729217    GUCAGCGUCCUCUCUAAUAAACGGGGUUGUCAGGCUCCUGUCAAAACCCUGGGAUGUGGUGACUGGAGUCACAGGAAUAGCCAUGACCGACACCACACCGUAUGGUCAGCAAAGAGUUUUCAAGGAAAAAGUGGACACUAGGGUGCCAGACCCCCAAGAAGGCACUCGUCAGGUUAUGAGC
KU729218    GUCAGCGUCCUCUCUAAUAAACGGGGUUGUCAGGCUCCUGUCAAAACCCUGGGAUGUGGUGACUGGAGUCACAGGAAUAGCCAUGACCGACACCACACCGUAUGGUCAGCAAAGAGUUUUCAAGGAAAAAGUGGACACUAGGGUGCCAGACCCCCAAGAAGGCACUCGUCAGGUUAUGAGC
KU761564    GUCAGCGUCCUCUCUAAUAAACGGGGUUGUCAGGCUCCUGUCAAAACCCUGGGAUGUGGUGACUGGAGUCACAGGAAUAGCCAUGACCGACACCACACCGUAUGGUCAGCAAAGAGUUUUCAAGGAAAAAGUGGACACUAGGGUGCCAGACCCCCAAGAAGGCACUCGUCAGGUUAUGAGC
KU740184    GUCAGCGUCCUCUCUAAUAAACGGGGUUGUCAGGCUCCUGUCAAAACCCUGGGAUGUGGUGACUGGAGUCACAGGAAUAGCCAUGACCGACACCACACCGUAUGGUCAGCAAAGAGUUUUCAAGGAAAAAGUGGACACUAGGGUGCCAGACCCCCAAGAAGGCACUCGUCAGGUUAUGAGC
KU744693    GUCAGCGUCCUCUCUAAUAAACGGGGUUGUCAGGCUCCUGUCAAAACCCUGGGAUGUGGUGACUGGAGUCACAGGAAUAGCCAUGACCGACACCACACCGUAUGGUCAGCAAAGAGUUUUCAAGGAAAAAGUGGACACUAGGGUGCCAGACCCCCAAGAAGGCACUCGUCAGGUUAUGAGC
KU497555    GUCAGCGUCCUCUCUAAUAAACGGGGUUGUCAGGCUCCUGUCAAAACCCUGGGAUGUGGUGACUGGAGUCACAGGAAUAGCCAUGACCGACACCACACCGUAUGGUCAGCAAAGAGUUUUCAAGGAAAAAGUGGACACUAGGGUGCCAGACCCCCAAGAAGGCACUCGUCAGGUUAUGAGC
KU527068    GUCAGCGUCCUCUCUAAUAAACGGGGUUGUCAGGCUCCUGUCAAAACCCUGGGAUGUGGUGACUGGAGUCACAGGAAUAGCCAUGACCGACACCACACCGUAUGGUCAGCAAAGAGUUUUCAAGGAAAAAGUGGACACUAGGGUGCCAGACCCCCAAGAAGGCACUCGUCAGGUUAUGAGC
KU501217    GUCAGCGUCCUCUCUAAUAAACGGGGUUGUCAGGCUCCUGUCAAAACCCUGGGAUGUGGUGACUGGAGUCACAGGAAUAGCCAUGACCGACACCACACCGUAUGGUCAGCAAAGAGUUUUCAAGGAAAAAGUGGACACUAGGGUGCCAGACCCCCAAGAAGGCACUCGUCAGGUUAUGAGC
KU501216    GUCAGCGUCCUCUCUAAUAAACGGGGUUGUCAGGCUCCUGUCAAAACCCUGGGAUGUGGUGACUGGAGUCACAGGAAUAGCCAUGACCGACACCACACCGUAUGGUCAGCAAAGAGUUUUCAAGGAAAAAGUGGACACUAGGGUGCCAGACCCCCAAGAAGGCACUCGUCAGGUUAUGAGC
KU501215    GUCAGCGUCCUCUCUAAUAAACGGGGUUGUCAGGCUCCUGUCAAAACCCUGGGAUGUGGUGACUGGAGUCACAGGAAUAGCCAUGACCGACACCACACCGUAUGGUCAGCAAAGAGUUUUCAAGGAAAAAGUGGACACUAGGGUGCCAGACCCCCAAGAAGGCACUCGUCAGGUUAUGAGC
KU365780    GUCAGCGUCCUCUCUAAUAAACGGGGUUGUCAGGCUCCUGUCAAAACCCUGGGAUGUGGUGACUGGAGUCACAGGAAUAGCCAUGACCGACACCACACCGUAUGGUCAGCAAAGAGUUUUCAAGGAAAAAGUGGACACUAGGGUGCCAGACCCCCAAGAAGGCACUCGUCAGGUUAUGAGC
KU365779    GUCAGCGUCCUCUCUAAUAAACGGGGUUGUCAGGCUCCUGUCAAAACCCUGGGAUGUGGUGACUGGAGUCACAGGAAUAGCCAUGACCGACACCACACCGUAUGGUCAGCAAAGAGUUUUCAAGGAAAAAGUGGACACUAGGGUGCCAGACCCCCAAGAAGGCACUCGUCAGGUUAUGAGC
KU707826    GUCAGCGUCCUCUCUAAUAAACGGGGUUGUCAGGCUCCUGUCAAAACCCUGGGAUGUGGUGACUGGAGUCACAGGAAUAGCCAUGACCGACACCACACCGUAUGGUCAGCAAAGAGUUUUCAAGGAAAAAGUGGACACUAGGGUGCCAGACCCCCAAGAAGGCACUCGUCAGGUUAUGAGC
KU365778    GUCAGCGUCCUCUCUAAUAAACGGGGUUGUCAGGCUCCUGUCAAAACCCUGGGAUGUGGUGACUGGAGUCACAGGAAUAGCCAUGACCGACACCACACCGUAUGGUCAGCAAAGAGUUUUCAAGGAAAAAGUGGACACUAGGGUGCCAGACCCCCAAGAAGGCACUCGUCAGGUUAUGAGC
KU365777    GUCAGCGUCCUCUCUAAUAAACGGGGUUGUCAGGCUCCUGUCAAAACCCUGGGAUGUGGUGACUGGAGUCACAGGAAUAGCCAUGACCGACACCACACCGUAUGGUCAGCAAAGAGUUUUCAAGGAAAAAGUGGACACUAGGGUGCCAGACCCCCAAGAAGGCACUCGUCAGGUUAUGAGC
KU312312    GUCAGCGUCCUCUCUAAUAAACGGGGUUGUCAGGCUCCUGUCAAAACCCUGGGAUGUGGUGACUGGAGUCACAGGAAUAGCCAUGACCGACACCACACCGUAUGGUCAGCAAAGAGUUUUCAAGGAAAAAGUGGACACUAGGGUGCCAGACCCCCAAGAAGGCACUCGUCAGGUUAUGAGC
KF268950    GUCAGCAUCUUCCCUCGUGAAUGGGGUUGUUAGACUCCUGUCAAAGCCCUGGGAUGUAGUGACUGGAGUUACAGGAAUAGCUAUGACUGAUACAACACCAUACGGCCAACAAAGAGUCUUCAAAGAAAAAGUGGACACCAGGGUGCCAGAUCCCCAAGAAGGCACUCGCCAGGCAAUGAAC
KF268949    AUCAGCGUCUUCCCUCGUGAAUGGGGUUGUUAGACUCCUGUCAAAGCCCUGGGAUGUUGUGACUGGAGUUACAGGAAUAGCUAUGACUGACACUACACCAUACGGCCAACAAAGAGUCUUCAAAGAAAAAGUGGACACCAGGGUGCCAGAUCCUCAAGAAGGCACUCGCCAGACAAUGAAC
KF268948    GUCAGCAUCUUCCCUCGUGAAUGGGGUUGUUAGACUCCUGUCAAAGCCCUGGGAUGUAGUGACUGGAGUUACAGGAAUAGCUAUGACUGAUACAACACCAUACGGCCAACAAAGAGUCUUCAAAGAAAAAGUGGACACCAGGGUGCCAGAUCCCCAAGAAGGCACUCGCCAGGCAAUGAAC
KJ776791    GUCAGCGUCCUCUCUAAUAAACGGGGUUGUCAGGCUCCUGUCAAAACCCUGGGAUGUGGUGACUGGAGUCACAGGAAUAGCCAUGACCGACACCACACCGUAUGGUCAGCAAAGAGUUUUCAAGGAAAAAGUGGACACUAGGGUGCCAGACCCCCAAGAAGGCACUCGUCAGGUUAUGAGC
KF383119    AUCAGCGUCUUCCCUCGUGAACGGGGUUGUUAGACUCCUGUCAAAGCCUUGGGACGUGGUGACUGGAGUUACAGGAAUAGCCAUGACUGACACCACACCAUACGGCCAACAAAGAGUCUUCAAAGAAAAAGUGGACACCAGGGUGCCAGAUCCCCAAGAAGGCACUCGCCAGGUAAUGAAC
KF383118    AUCAGCGUCUUCCCUCGUGAACGGGGUUGUUAGACUCCUGUCAAAGCCUUGGGACGUGGUGACUGGAGUUACAGGAAUAGCCAUGACUGACACCACACCAUACGGCCAACAAAGAGUCUUCAAAGAAAAAGUGGACACCAGGGUGCCAGAUCCCCAAGAAGGCACUCGCCAGGUAAUGAAC
KF383117    GUCAGCGUCAUCCCUCGUGAAUGGGGUUGUUAGACUCUUGUCAAAGCCCUGGGAUGUGGUGACUGGAGUUACAGGAAUAGCUAUGACUGACACCACGCCAUACGGCCAACAAAGAGUCUUCAAGGAAAAAGUGGACACAAGGGUGCCAGAUCCCCAAGAAGGCACUCGCCAAGUAAUGAAC
KF383116    GUCAGCGUCAUCCCUCGUGAACGGGGUUGUUAGACUCUUGUCAAAACCCUGGGAUGUGGUGACUGGAGUUACAGGAAUAGCUAUGACUGACACCACGCCAUACGGCCAACAAAGAGUCUUCAAGGAAAAAGUGGACACUAGGGUGCCAGAUCCCCAAGAAGGCACUCGCCAAGUAAUGAAC
KF383115    GUCAGCGUCUUCCCUCGUGAAUGGGGUUGUUAGGCUCCUGUCAAAGCCCUGGGAUGUAGUGACUGGAGUUACAGGAAUAGCUAUGACUGACACCACACCAUAUGGCCAACAAAGAGUCUUCAAAGAAAAAGUGGACACCAGGGUGCCAGAUCCCCAAGAAGGCACUCGCCAGACAAUGAAC
EU545988    GUCAGCGUCCUCUCUAAUAAACGGGGUUGUCAGGCUCCUGUCAAAACCCUGGGAUGUGGUGACUGGAGUCACAGGAAUAGCCAUGACCGACACCACACCGUAUGGUCAGCAAAGAGUUUUCAAGGAAAAAGUGGACACUAGGGUGCCAGACCCCCAAGAAGGCACUCGUCAGGUUAUGAGC
KU955589    GUCAGCGUCCUCUCUAAUAAACGGGGUUGUCAGGCUCCUGUCAAAACCCUGGGACGUGGUGACUGGAGUCACAGGAAUAGCCAUGACCGACACCACACCGUAUGGUCAGCAAAGAGUUUUCAAGGAAAAAGUGGACACUAGGGUGCCAGAUCCCCAAGAAGGCACUCGUCAGGUUAUGAGC
KU955590    GUCAGCGUCCUCUCUAAUAAACGGGGUUGUCAGGCUCCUGUCAAAACCCUGGGAUGUGGUGACUGGAGUCACAGGAAUAGCCAUGACCGACACCACACCGUAUGGUCAGCAAAGAGUUUUCAAGGAAAAAGUGGACACUAGGGUGCCAGACCCCCAAGAAGGCACUCGUCAGGUUAUGAGC
KU940228    GUCAGCGUCCUCUCUAGUAAACGGGGUUGUCAGGCUCCUGUCAAAACCCUGGGAUGUGGUGACUGGAGUCACAGGAAUAGCCAUGACCGACACCACACCGUAUGGUCAGCAAAGAGUUUUCAAGGAAAAAGUGGACACUAGGGUGCCAGACCCCCAAGAAGGCACUCGUCAGGUUAUGAGC
KU991811    GUCAGCGUCCUCUCUAAUAAACGGGGUUGUCAGGCUCCUGUCAAAACCCUGGGAUGUGGUGACUGGAGUCACAGGAAUAGCCAUGACCGACACCACACCGUAUGGUCAGCAAAGAGUUUUCAAGGAAAAAGUGGACACUAGGGUGCCAGACCCCCAAGAAGGCACUCGUCAGGUUAUGAGC
KU509998    GUCAGCGUCCUCUCUAAUAAACGGGGUUGUCAGGCUCCUGUCAAAACCCUGGGAUGUGGUGACUGGAGUCACAGGAAUAGCCAUGACCGACACCACACCGUAUGGUCAGCAAAGAGUUUUCAAGGAAAAAGUGGACACUAGGGUGCCAGACCCCCAAGAAGGCACUCGUCAGGUUAUGAGC
KX056898    GUCAGCGUCCUCUCUAAUAAACGGGGUUGUCAGGCUCCUGUCAAAACCCUGGGAUGUGGUGACUGGAGUCACAGGAAUAGCCAUGACCGACACCACACCGUAUGGUCAGCAAAGAGUUUUCAAGGAAAAAGUGGACACUAGGGUGCCAGACCCCCAAGAAGGCACUCGUCAGGUUAUGAGC
KX117076    GUCAGCGUCCUCUCUAAUAAACGGGGUUGUCAGGCUCCUGUCAAAACCCUGGGAUGUGGUGACUGGAGUCACAGGAAUAGCCAUGACCGACACCACACCGUAUGGUCAGCAAAGAGUUUUCAAGGAAAAAGUGGACACUAGGGUGCCAGAUCCCCAAGAAGGCACUCGUCAGGUUAUGAGC
KX185891    GUCAGCGUCCUCUCUAAUAAACGGGGUUGUCAGGCUCCUGUCAAAACCCUGGGAUGUGGUGACUGGAGUCACAGGAAUAGCCAUGACCGACACCACACCGUAUGGUCAGCAAAGAGUUUUCAAGGAAAAAGUGGACACUAGGGUGCCAGAUCCCCAAGAAGGCACUCGUCAGGUUAUGAGC
KX247646    GUCAGCGUCCUCUCUAAUAAACGGGGUUGUCAGGCUCCUGUCAAAACCCUGGGAUGUGGUGACUGGAGUCACAGGAAUAGCCAUGACCGACACCACACCGUAUGGUCAGCAAAGAGUUUUCAAGGAAAAAGUGGACACUAGGGUGCCAGACCCCCAAGAAGGCACUCGUCAGGUUAUGAGC
KX262887    GUCAGCGUCCUCUCUAAUAAACGGGGUUGUCAGGCUCCUGUCAAAACCCUGGGAUGUGGUGACUGGAGUCACAGGAAUAGCCAUGACCGACACCACACCGUAUGGUCAGCAAAGAGUUUUCAAGGAAAAAGUGGACACUAGGGUGCCAGACCCCCAAGAAGGCACUCGUCAGGUUAUGAGC
KX280026    GUCAGCGUCCUCUCUAAUAAACGGGGUUGUCAGGCUCCUGUCAAAACCCUGGGAUGUGGUGACUGGAGUCACAGGAAUAGCCAUGACCGACACCACACCGUAUGGUCAGCAAAGAGUUUUCAAGGAAAAAGUGGACACUAGGGUGCCAGACCCCCAAGAAGGCACUCGUCAGGUUAUGAGC
KU758877    GUCAGCGUCCUCUCUAAUAAACGGGGUUGUCAGGCUCCUGUCAAAACCCUGGGAUGUGGUGACUGGAGUCACAGGAAUAGCCAUGACCGACACCACACCGUAUGGUCAGCAAAGAGUUUUCAAGGAAAAAGUGGACACUAGGGUGCCAGACCCCCAAGAAGGCACUCGUCAGGUUAUGAGC
KU866423    GUCAGCGUCCUCUCUAAUAAACGGGGUUGUCAGGCUCCUGUCAAAACCCUGGGAUGUGGUGACUGGAGUCACAGGAAUAGCCAUGACCGACACCACACCGUAUGGUCAGCAAAGAGUUUUCAAGGAAAAAGUGGACACUAGGGUGCCAGAUCCCCAAGAAGGCACUCGUCAGGUUAUGAGC
KX377336    GUCAGCGUCUUCUCUCAUAAACGGGGUUGUCAGGCUCCUGUCAAAGCCCUGGGAUGUGGUGACUGGAGUCACAGGAAUAGCCAUGACCGACACCACACCGUAUGGCCAGCAAAGAGUUUUCAAGGAAAAAGUGGACACUAGGGUGCCAGACCCCCAGGAAGGCACUCGUCAGGUGAUGAAC
KX377337    GUCAGCGUCCUCUCUAAUAAACGGGGUUGUCAGGCUCCUGUCAAAACCCUGGGAUGUGGUGACUGGAGUCACAGGAAUAGCCAUGACCGACACCACACCGUAUGGUCAGCAAAGAGUUUUCAAGGAAAAAGUGGACACUAGGGUGCCAGACCCCCAAGAAGGCACUCGUCAGGUUAUGAGC
KX369547    GUCAGCGUCCUCUCUAAUAAACGGGGUUGUCAGGCUCCUGUCAAAACCCUGGGAUGUGGUGACUGGAGUCACAGGAAUAGCCAUGACCGACACCACACCGUAUGGUCAGCAAAGAGUUUUCAAGGAAAAAGUGGACACUAGGGUGCCAGACCCCCAAGAAGGCACUCGUCAGGUUAUGAGC
KX548902    GUCAGCGUCCUCUCUAAUAAACGGGGUUGUCAGGCUCCUGUCAAAACCCUGGGAUGUGGUGACUGGAGUCACAGGAAUAGCCAUGACCGACACCACACCGUAUGGUCAGCAAAGAGUUUUCAAGGAAAAAGUGGACACUAGGGUGCCAGACCCCCAAGAAGGCACUCGUCAGGUUAUGAGC
KX601166    GUCAGCGUCAUCCCUCGUGAACGGGGUUGUUAGACUCUUGUCAAAGCCCUGGGAUGUGGUGACUGGAGUUACAGGAAUAGCUAUGACUGACACCACGCCAUACGGCCAACAAAGAGUCUUCAAGGAAAAAGUGGACACCAGGGUGCCAGAUCCCCAAGAAGGCACUCGCCAAGUGAUGAAC
KX601167    GUCAGCGUCUUCUCUCAUAAACGGGGUUGUCAGGCUCCUGUCAAAGCCCUGGGAUGUGGUGACUGGAGUCACAGGAAUAGCCAUGACCGACACCACACCGUAUGGCCAGCAAAGAGUUUUCAAGGAAAAAGUGGACACUAGGGUGCCAGACCCCCAGGAAGGCACUCGUCAGGUGAUGAAC
KX601168    GUCAGCGUCCUCUCUAAUAAACGGGGUUGUCAGGCUCCUGUCAAAACCCUGGGAUGUGGUGACUGGAGUCACAGGAAUAGCCAUGACCGACACCACACCGUAUGGUCAGCAAAGAGUUUUCAAGGAAAAAGUGGACACUAGGGUGCCAGACCCCCAAGAAGGCACUCGUCAGGUUAUGAGC
KX766028    GUCAGCGUCCUCUCUAAUAAACGGGGUUGUCAGGCUCCUGUCAAAACCCUGGGAUGUGGUGACUGGAGUCACAGGAAUAGCCAUGACCGACACCACACCGUAUGGUCAGCAAAGAGUUUUCAAGGAAAAAGUGGACACUAGGGUGCCAGACCCCCAAGAAGGCACUCGUCAGGUUAUGAGC
KX766029    GUCAGCGUCCUCUCUAAUAAACGGGGUUGUCAGGCUCCUGUCAAAACCCUGGGAUGUGGUGACUGGAGUCACAGGAAUAGCCAUGACCGACACCACACCGUAUGGUCAGCAAAGAGUUUUCAAGGAAAAAGUGGACACUAGGGUGCCAGACCCCCAAGAAGGCACUCGUCAGGUUAUGAGC
KU940224    GUCAGCGUCCUCUCUAGUAAACGGGGUUGUCAGGCUCCUGUCAAAACCCUGGGAUGUGGUGACUGGAGUCACAGGAAUAGCCAUGACCGACACCACACCGUAUGGUCAGCAAAGAGUUUUCAAGGAAAAAGUGGACACUAGGGUGCCAGACCCCCAAGAAGGCACUCGUCAGGUUAUGAGC


            8888888888888888888888888888888888888888888888888888888888888888888888888888888888888888888888888888888888888888888888888888888888888888888888888888888888888888888888888888888
            6666666666677777777777777777777777777777777777777777777777777777777777777777777777777777777777777777777777777888888888888888888888888888888888888888888888888888888888888888888888
            8999999999900000000001111111111222222222233333333334444444444555555555566666666667777777777888888888899999999990000000000111111111122222222223333333333444444444455555555556666666666
Sequence    9012345678901234567890123456789012345678901234567890123456789012345678901234567890123456789012345678901234567890123456789012345678901234567890123456789012345678901234567890123456789
-------------------------------------------------------------------------------------------------------------------------------------------------------------------------------------------------
NC_012532   AUAGUCUCUUCCUGGCUGUGGAAGGAGCUGGGGAAACGCAAGCGGCCACGCGUCUGCACCAAAGAAGAGUUUAUCAACAAGGUGCGCAGCAAUGCAGCACUGGGAGCAAUAUUUGAAGAGGAAAAAGAAUGGAAGACGGCUGUGGAAGCUGUGAAUGAUCCAAGGUUUUGGGCCCUAGUGG
KU922960    AUGGUCUCUUCCUGGUUGUGGAAAGAGCUAGGCAAACACAAACGGCCACGAGUCUGUACCAAAGAAGAGUUCAUCAACAAGGUUCGUAGCAAUGCAGCAUUAGGGGCAAUAUUUGAAGAGGAAAAAGAGUGGAAGACUGCAGUGGAAGCUGUGAACGAUCCAAGGUUCUGGGCUCUAGUGG
KU922923    AUGGUCUCUUCCUGGUUGUGGAAAGAGCUAGGCAAACACAAACGGCCACGAGUCUGUACCAAAGAAGAGUUCAUCAACAAGGUUCGUAGCAAUGCAGCAUUAGGGGCAAUAUUUGAAGAGGAAAAAGAGUGGAAGACUGCAGUGGAAGCUGUGAACGAUCCAAGGUUCUGGGCUCUAGUGG
KU820898    AUGGUCUCUUCCUGGUUGUGGAAAGAGCUAGGCAAACACAAACGGCCACGAGUCUGUACCAAAGAAGAGUUCAUCAACAAGGUUCGUAGCAAUGCAGCAUUAGGGGCAAUAUUUGAAGAGGAAAAAGAGUGGAAGACUGCAGUGGAAGCUGUGAACGAUCCAAGGUUCUGGGCUCUAGUGG
KU321639    AUGGUCUCUUCCUGGUUGUGGAAAGAGCUAGGCAAACACAAACGGCCACGAGUCUGUACCAAAGAAGAGUUCAUCAACAAGGUUCGUAGCAAUGCAGCAUUAGGGGCAAUAUUUGAAGAGGAAAAAGAGUGGAAGACUGCAGUGGAAGCUGUGAACGAUCCAAGGUUCUGGGCUCUAGUGG
KU820899    AUGGUCUCUUCCUGGUUGUGGAAAGAGCUAGGCAAACACAAACGGCCACGAGUCUGUACCAAAGAAGAGUUCAUCAACAAGGUUCGUAGCAAUGCAGCAUUAGGGGCAAUAUUUGAAGAGGAAAAAGAGUGGAAGACUGCAGUGGAAGCUGUGAACGAUCCAAGGUUCUGGGCUCUAGUGG
```

```
KU853012       AUGGUCUCUUCCUGGUUGUGGAAAGAGCUAGGCAAACACAAACGGCCACGAGUCUGUACCAAAGAAGAGUUCAUCAACAAGGUUCGUAGCAAUGCAGCAUUAGGGGCAAUAUUUGAAGAGGAAAAAGAGUGGAAGACUGCAGUGGAAGCUGUGAACGAUCCAAGGUUCUGGGCUCUAGUGG
KU729217       AUGGUCUCUUCCUGGUUGUGGAAAGAGCUAGGCAAACACAAACGGCCACGAGUCUGUACCAAAGAAGAGUUCAUCAACAAGGUUCGUAGCAAUGCAGCAUUAGGGGCAAUAUUUGAAGAGGAAAAAGAGUGGAAGACUGCAGUGGAGGCUGUGAACGAUCCAAGGUUCUGGGCUCUAGUGG
KU729218       AUGGUCUCUUCCUGGUUGUGGAAAGAGCUAGGCAAACACAAACGGCCACGAGUCUGUACCAAAGAAGAGUUCAUCAACAAGGUUCGUAGCAAUGCAGCAUUAGGGGCAAUAUUUGAAGAGGAAAAAGAGUGGAAGACUGCAGUGGAAGCUGUGAACGAUCCAAGGUUCUGGGCUCUAGUGG
KU761564       AUGGUCUCUUCCUGGUUGUGGAAAGAGCUAGGCAAACACAAACGGCCACGAGUCUGUACCAAAGAAGAGUUCAUCAACAAGGUUCGUAGCAAUGCAGCAUUAGGGGCAAUAUUUGAAGAGGAAAAAGAGUGGAAGACUGCAGUGGAAGCUGUGAACGAUCCAAGGUUCUGGGCUCUAGUGG
KU740184       AUGGUCUCUUCCUGGUUGUGGAAAGAGCUAGGCAAACACAAACGGCCACGAGUCUGUACCAAAGAAGAGUUCAUCAACAAGGUUCGUAGCAAUGCAGCAUUAGGGGCAAUAUUUGAAGAGGAAAAAGAGUGGAAGACUGCAGUGGAAGCUGUGAACGAUCCAAGGUUCUGGGCUCUAGUGG
KU744693       AUGGUCUCUUCCUGGUUGUGGAAAGAGCUAGGCAAACACAAACGGCCACGAGUCUGUACCAAAGAAGAGUUCAUCAACAAGGUUCGUAGCAAUGCAGCAUUAGGGGCAAUAUUUGAAGAGGAAAAAGAGUGGAAGACUGCAGUGGAAGCUGUGAACGAUCCAAGGUUCUGGGCUCUAGUGG
KU497555       AUGGUCUCUUCCUGGUUGUGGAAAGAGCUAGGCAAACACAAACGACCACGAGUCUGUACCAAAGAAGAGUUCAUCAACAAGGUUCGUAGCAAUGCAGCAUUAGGGGCAAUAUUUGAAGAGGAAAAAGAGUGGAAGACUGCAGUGGAAGCUGUGAACGAUCCAAGGUUCUGGGCUCUAGUGG
KU527068       AUGGUCUCUUCCUGGUUGUGGAAAGAGCUAGGCAAACACAAACGGCCACGAGUCUGUACCAAAGAAGAGUUCAUCAACAAGGUUCGUAGCAAUGCAGCAUUAGGGGCAAUAUUUGAAGAGGAAAAAGAGUGGAAGACUGCAGUGGAAGCUGUGAACGAUCCAAGGUUCUGGGCUCUAGUGG
KU501217       AUGGUCUCUUCCUGGUUGUGGAAAGAGCUAGGCAAACACAAACGGCCACGAGUCUGUACCAAAGAAGAGUUCAUCAACAAGGUUCGUAGCAAUGCAGCAUUAGGGGCAAUAUUUGAAGAGGAAAAAGAGUGGAAGACUGCAGUGGAAGCUGUGAACGAUCCAAGGUUCUGGGCUCUAGUGG
KU501216       AUGGUCUCUUCCUGGUUGUGGAAAGAGCUAGGCAAACACAAACGGCCACGAGUCUGUACCAAAGAAGAGUUCAUCAACAAGGUUCGUAGCAAUGCAGCAUUAGGGGCAAUAUUUGAAGAGGAAAAAGAGUGGAAGACUGCAGUGGAAGCUGUGAACGAUCCAAGGUUCUGGGCUCUAGUGG
KU501215       AUGGUCUCUUCCUGGUUGUGGAAAGAGCUAGGCAAACACAAACGGCCACGAGUCUGCACCAAAGAAGAGUUCAUCAACAAGGUUCGUAGCAAUGCAGCAUUAGGGGCAAUAUUUGAAGAGGAAAAAGAGUGGAAGACUGCAGUGGAAGCUGUGAACGAUCCAAGGUUCUGGGCUCUAGUGG
KU365780       AUGGUCUCUUCCUGGUUGUGGAAAGAGCUAGGCAAACACAAACGGCCACGAGUCUGUACCAAAGAAGAGUUCAUCAACAAGGUUCGUAGCAAUGCAGCAUUAGGGGCAAUAUUUGAAGAGGAAAAAGAGUGGAAGACUGCAGUGGAAGCUGUGAACGAUCCAAGGUUCUGGGCUCUAGUGG
KU365779       AUGGUCUCUUCCUGGUUGUGGAAAGAGCUAGGCAAACACAAACGGCCACGAGUCUGUACCAAAGAAGAGUUCAUCAACAAGGUUCGUAGCAAUGCAGCAUUAGGGGCAAUAUUUGAAGAGGAAAAAGAGUGGAAGACUGCAGUGGAAGCUGUGAACGAUCCAAGGUUCUGGGCUCUAGUGG
KU707826       AUGGUCUCUUCCUGGUUGUGGAAAGAGCUAGGCAAACACAAACGGCCACGAGUCUGUACCAAAGAAGAGUUCAUCAACAAGGUUCGUAGCAAUGCAGCAUUAGGGGCAAUAUUUGAAGAGGAAAAAGAGUGGAAGACUGCAGUGGAAGCUGUGAACGAUCCAAGGUUCUGGGCUCUAGUGG
KU365778       AUGGUCUCUUCCUGGUUGUGGAAAGAGCUAGGCAAACACAAACGGCCACGAGUCUGUACCAAAGAAGAGUUCAUCAACAAGGUUCGUAGCAAUGCAGCAUUAGGGGCAAUAUUUGAAGAGGAAAAAGAGUGGAAGACUGCAGUGGAAGCUGUGAACGAUCCAAGGUUCUGGGCUCUAGUGG
KU365777       AUGGUCUCUUCCUGGUUGUGGAAAGAGCUAGGCAAACACAAACGGCCACGAGUCUGUACCAAAGAAGAGUUCAUCAACAAGGUUCGUAGCAAUGCAGCAUUAGGGGCAAUAUUUGAAGAGGAAAAAGAGUGGAAGACUGCAGUGGAAGCUGUGAACGAUCCAAGGUUCUGGGCUCUAGUGG
KU312312       AUGGUCUCUUCCUGGUUGUGGAAAGAGCUAGGCAAACACAAACGGCCACGAGUCUGUACCAAAGAAGAGUUCAUCAACAAGGUUCGUAGCAAUGCAGCAUUAGGGGCAAUAUUUGAAGAGGAAAAAGAGUGGAAGACUGCAGUGGAAGCUGUGAACGAUCCAAGGUUCUGGGCUCUAGUGG
KF268950       AUGGUCUCUUCCUGGCUGUGGAAGGAGUUGGGGAAACGCAAGCGGCCACGUGUCUGCACCAAAGAAGAGUUCAUCAACAAGGUGCGCAGCAAUGCAGCACUGGGAGCAAUAUUUGAAGAGGAAAAAGAAUGGAAGACGGCCGUGGAGGCUGUGAAUGAUCCAAGGUUUUGGGCUCUAGUGG
KF268949       AUGGUUUCUUCCUGGCUGUGGAAGGAGCUGGGGAAACGCAAGCGGCCACGUGUCUGCACCAAAGAAGAGUUCAUCAACAAGGUGCGCAGCAAUGCAGCACUGGGAGCAAUAUUUGAAGAGGAAAAAGAAUGGAAGACGGCCGUGGAAGCUGUGAAUGAUCCAAGGUUUUGGGCCCUAGUGG
KF268948       AUGGUCUCUUCCUGGCUGUGGAAGGAGUUGGGGAAACGCAAGCGGCCACGUGUCUGCACCAAAGAAGAGUUCAUCAACAAGGUGCGCAGCAAUGCAGCACUGGGAGCAAUAUUUGAAGAGGAAAAAGAAUGGAAGACGGCCGUGGAGGCUGUGAAUGAUCCAAGGUUUUGGGCUCUAGUGG
KJ776791       AUGGUCUCUUCCUGGUUGUGGAAAGAGCUAGGCAAACACAAACGGCCACGAGUCUGUACCAAAGAAGAGUUCAUCAACAAGGUUCGUAGCAAUGCAGCAUUAGGGGCAAUAUUUGAAGAGGAAAAAGAGUGGAAGACUGCAGUGGAAGCUGUGAACGAUCCAAGGUUCUGGGCUCUAGUGG
KF383119       AUGGUCUCUUCCUGGCUGUGGAAGGAGCUGGGGAAACGCAAGCGGCCACGCGUCUGCACCAAAGAAGAGUUUAUCAACAAGGUGCGCAGCAAUGCAGCACUGGGAGCAAUAUUUGAAGAGGAAAAAGAAUGGAAGACGGCUGUGGAAGCUGUGAAUGAUCCAAGGUUUUGGGCCCUAGUGG
KF383118       AUGGUCUCUUCCUGGCUGUGGAAGGAGCUGGGGAAACGCAAGCGGCCACGCGUCUGCACCAAAGAAGAGUUUAUCAACAAGGUGCGCAGCAAUGCAGCACUGGGAGCAAUAUUUGAAGAGGAAAAAGAAUGGAAGACGGCUGUGGAAGCUGUGAAUGAUCCAAGGUUUUGGGCCCUAGUGG
KF383117       AUAGUUUCGUCUUGGCUAUGGAAGGAGCUAGGAAAACGCAAGCGACCACGUGUCUGCACCAAAGAAGAGUUCAUCAAUAAGGUGCGCAGCAAUGCAGCACUGGGAGCAAUAUUUGAAGAGGAAAAAGAAUGGAAGACAGCUGUAGAAGCUGUGAAUGAUCCAAGAUUUUGGGCUCUAGUGG
KF383116       AUGGUCUCGUCUUGGCUAUGGAAGGAGCUGGGAAAACGCAAGCGGCCACGUGUCUGCACCAAAGAAGAGUUCAUUAAUAAGGUGCGCAGCAAUGCAGCACUGGGAGCAAUAUUUGAAGAGGAAAAAGAAUGGAAGACAGCUGUAGAAGCUGUGAAUGAUCCAAGAUUUUGGGCUCUAGUGG
KF383115       AUGGUCUCGUCCUGGCUGUGGAAGGAGCUGGGAAAACGCAAGCGGCCACGCGUCUGCACCAAAGAAGAGUUCAUCAACAAGGUGCGCAGCAAUGCAGCGCUGGGAGCAAUAUUUGAAGAGGAAAAAGAAUGGAAGACGGCCGUGGAAGCUGUGAAUGAUCCAAGGUUUUGGGCCCUAGUGG
EU545988       AUGGUCUCUUCCUGGUUAUGGAAAGAGCUAGGCAAACACAAACGGCCACGAGUCUGUACCAAAGAAGAGUUCAUCAACAAGGUUCGUAGCAAUGCAGCAUUAGGGGCAAUAUUUGAAGAGGAAAAAGAGUGGAAGACUGCAGUGGAAGCUGUGAACGAUCCAAGGUUCUGGGCUCUAGUGG
KU955589       AUGGUCUCUUCCUGGUUGUGGAAAGAGCUAGGCAAACACAAACGGCCACGAGUCUGUACCAAAGAAGAGUUCAUCAACAAGGUUCGUAGCAAUGCAGCAUUAGGGGCAAUAUUUGAAGAGGAAAAAGAGUGGAAGACUGCAGUGGAAGCUGUGAACGAUCCAAGGUUCUGGGCUCUAGUGG
KU955590       AUGGUCUCUUCCUGGUUGUGGAAAGAGCUAGGCAAACACAAACGGCCACGAGUCUGUACCAAAGAAGAGUUCAUCAACAAGGUUCGUAGCAAUGCAGCAUUAGGGGCAAUAUUUGAAGAGGAAAAAGAGUGGAAGACUGCAGUGGAAGCUGUGAACGAUCCAAGGUUCUGGGCUCUAGUGG
KU940228       AUGGUCUCUUCCUGGUUGUGGAAAGAGCUAGGCAAACACAAACGGCCACGAGUCUGUACCAAAGAAGAGUUCAUCAACAAGGUUCGUAGCAAUGCAGCACUAGGGGCAAUAUUUGAAGAGGAAAAAGAGUGGAAGACUGCAGUGGAAGCUGUGAACGAUCCAAGGUUCUGGGCUCUAGUGG
KU991811       AUGGUCUCUUCCUGGUUGUGGAAAGAGCUAGGCAAACACAAACGGCCACGAGUCUGUACCAAAGAAGAGUUCAUCAACAAGGUUCGUAGCAAUGCAGCAUUAGGGGCAAUAUUUGAAGAGGAAAAAGAGUGGAAGACUGCAGUGGAAGCUGUGAACGAUCCAAGGUUCUGGGCUCUAGUGG
KU509998       AUGGUCUCUUCCUGGUUGUGGAAAGAGCUAGGCAAACACAAACGGCCACGAGUCUGUACCAAAGAAGAGUUCAUCAACAAGGUUCGUAGCAAUGCAGCAUUAGGGGCAAUAUUUGAAGAGGAAAAAGAGUGGAAGACUGCAGUGGAAGCUGUGAACGAUCCAAGGUUCUGGGCUCUAGUGG
KX056898       AUGGUCUCUUCCUGGUUGUGGAAAGAGCUAGGCAAACACAAACGGCCACGAGUCUGUACCAAAGAAGAGUUCAUCAACAAGGUUCGUAGCAAUGCAGCAUUAGGGGCAAUAUUUGAAGAGGAAAAAGAGUGGAAGACUGCAGUGGAAGCUGUGAACGAUCCAAGGUUCUGGGCUCUAGUGG
KX117076       AUGGUCUCUUCCUGGUUGUGGAAAGAGCUAGGCAAACACAAACGGCCACGAGUCUGUACCAAAGAAGAGUUCAUCAACAAGGUUCGUAGCAAUGCAGCAUUAGGGGCAAUAUUUGAAGAGGAAAAAGAGUGGAAGACUGCAGUGGAAGCUGUGAACGAUCCAAGGUUCUGGGCUCUAGUGG
KX185891       AUGGUCUCUUCCUGGUUGUGGAAAGAGCUAGGCAAACACAAACGGCCACGAGUCUGUACCAAAGAAGAGUUCAUCAACAAGGUUCGUAGCAAUGCAGCAUUAGGGGCAAUAUUUGAAGAGGAAAAAGAGUGGAAGACUGCAGUGGAAGCUGUGAACGAUCCAAGGUUCUGGGCUCUAGUGG
KX247646       AUGGUCUCUUCCUGGUUGUGGAAAGAGCUAGGCAAACACAAACGGCCACGAGUCUGUACCAAAGAAGAGUUCAUCAACAAGGUGCGUAGCAAUGCAGCAUUAGGGGCAAUAUUUGAAGAGGAAAAAGAGUGGAAGACUGCAGUGGAAGCUGUGAACGAUCCAAGGUUCUGGGCUCUAGUGG
KX262887       AUGGUCUCUUCCUGGUUGUGGAAAGAGCUAGGCAAACACAAACGGCCACGAGUCUGUACCAAAGAAGAGUUCAUCAACAAGGUUCGUAGCAAUGCAGCAUUAGGGGCAAUAUUUGAAGAGGAAAAAGAGUGGAAGACUGCAGUGGAAGCUGUGAACGAUCCAAGGUUCUGGGCUCUAGUGG
KX280026       AUGGUCUCUUCCUGGUUGUGGAAAGAGCUAGGCAAACACAAACGGCCACGAGUCUGUACCAAAGAAGAGUUUAUCAACAAGGUUCGUAGCAAUGCAGCAUUAGGGGCAAUAUUUGAAGAGGAAAAAGAGUGGAAGACUGCAGUGGAAGCUGUGAACGAUCCAAGGUUCUGGGCUCUAGUGG
KU758877       AUGGUCUCUUCCUGGUUGUGGAAAGAGCUAGGCAAACACAAACGGCCACGAGUCUGUACCAAAGAAGAGUUCAUCAACAAGGUUCGUAGCAAUGCAGCAUUAGGGGCAAUAUUUGAAGAGGAAAAAGAGUGGAAGACUGCAGUGGAAGCUGUGAACGAUCCAAGGUUCUGGGCUCUAGUGG
KU866423       AUGGUCUCUUCCUGGUUGUGGAAAGAGCUAGGCAAACACAAACGGCCACGAGUCUGUACCAAAGAAGAGUUCAUCAACAAGGUUCGUAGCAAUGCAGCAUUAGGGGCAAUAUUUGAAGAGGAAAAAGAGUGGAAGACUGCAGUGGAAGCUGUGAACGAUCCAAGGUUCUGGGCUCUAGUGG
KX377336       AUGGUCUCUUCCUGGCUAUGGAAGGAGCUAGGUAAACACAAACGGCCACGAGUUUGCACCAAAGAAGAGUUCAUCAAUAAGGUUCGCAGCAAUGCAGCACUGGGGGCAAUAUUUGAAGAGGAGAAAGAAUGGAAGACUGCAGUGGAAGCUGUGAACGAUCCAAGGUUCUGGGCCCUAGUGG
KX377337       AUGGUCUCUUCCUGGUUGUGGAAAGAGCUAGGCAAACACAAACGGCCACGAGUCUGCACCAAAGAAGAGUUCAUCAACAAGGUUCGUAGCAAUGCAGCAUUAGGGGCAAUAUUUGAAGAGGAAAAAGAGUGGAAGACUGCAGUGGAAGCUGUGAACGAUCCAAGGUUCUGGGCUCUAGUGG
KX369547       AUGGUCUCUUCCUGGUUGUGGAAAGAGCUAGGCAAACACAAACGGCCACGAGUCUGUACCAAAGAAGAGUUCAUCAACAAGGUUCGUAGCAAUGCAGCAUUAGGGGCAAUAUUUGAAGAGGAAAAAGAGUGGAAGACUGCAGUGGAAGCUGUGAACGAUCCAAGGUUCUGGGCUCUAGUGG
KX548902       AUGGUCUCUUCCUGGUUGUGGAAAGAGCUAGGCAAACACAAACGGCCACGAGUCUGUACCAAAGAAGAGUUCAUCAACAAGGUUCGUAGCAAUGCAGCAUUAGGGGCAAUAUUUGAAGAGGAAAAAGAGUGGAAGACUGCAGUGGAAGCUGUGAACGAUCCAAGGUUCUGGGCUCUAGUGG
KX601166       AUGGUAUCGUCUUGGCUAUGGAAGGAGCUGGGAAAACGCAAGCGACCACGUGUCUGCACCAAAGAAGAGUUCAUUAAUAAGGUGCGCAGCAAUGCAGCACUGGGAGCAAUAUUUGAAGAGGAAAAAGAAUGGAAGACAGCUGUAGAAGCUGUGAAUGAUCCAAGAUUUUGGGCUCUAGUGG
KX601167       AUGGUCUCUUCCUGGCUAUGGAAGGAGCUAGGUAAACACAAACGGCCACGAGUUUGCACCAAAGAAGAGUUCAUCAAUAAGGUUCGCAGCAAUGCAGCACUGGGGGCAAUAUUUGAAGAGGAGAAAGAAUGGAAGACUGCAGUGGAAGCUGUGAACGAUCCAAGGUUCUGGGCCCUAGUGG
KX601168       AUGGUCUCUUCCUGGUUGUGGAAAGAGCUAGGCAAACACAAACGGCCACGAGUCUGCACCAAAGAAGAGUUCAUCAACAAGGUUCGUAGCAAUGCAGCAUUAGGGGCAAUAUUUGAAGAGGAAAAAGAGUGGAAGACUGCAGUGGAAGCUGUGAACGAUCCAAGGUUCUGGGCUCUAGUGG
KX766028       AUGGUCUCUUCCUGGUUGUGGAAAGAGCUAGGCAAACACAAACGGCCACGAGUCUGUACCAAAGAAGAGUUCAUCAACAAGGUUCGUAGCAAUGCAGCAUUAGGGGCAAUAUUUGAAGAGGAAAAAGAGUGGAAGACUGCAGUGGAAGCUGUGAACGAUCCAAGGUUCUGGGCUCUAGUGG
KX766029       AUGGUCUCUUCCUGGUUGUGGAAAGAGCUAGGCAAACACAAACGGCCACGAGUCUGUACCAAAGAAGAGUUCAUCAACAAGGUUCGUAGCAAUGCAGCAUUAGGGGCAAUAUUUGAAGAGGAAAAAGAGUGGAAGACUGCAGUGGAAGCUGUGAACGAUCCAAGGUUCUGGGCUCUAGUGG
KU940224       AUGGUCUCUUCCUGGUUGUGGAAAGAGCUAGGCAAACACAAACGGCCACGAGUCUGUACCAAAGAAGAGUUCAUCAACAAGGUUCGUAGCAAUGCAGCACUAGGGGCAAUAUUUGAAGAGGAAAAAGAGUGGAAGACUGCAGUGGAAGCUGUGAACGAUCCAAGGUUCUGGGCUCUAGUGG


               8888888888888888888888888888888888888888888888888888888888888888888888888888888888888888888888888888888888888888888888888888888888999999999999999999999999999999999999999999999999999
               8888888888888888888888888888889999999999999999999999999999999999999999999999999999999999999999999999999999999999999999999999999999000000000000000000000000000000000000000000000000000
               7777777777888888888899999999990000000000111111111122222222223333333333444444444455555555556666666666777777777788888888889999999999000000000011111111112222222222333333333344444444445
Sequence       0123456789012345678901234567890123456789012345678901234567890123456789012345678901234567890123456789012345678901234567890123456789012345678901234567890123456789012345678901234567890
----------------------------------------------------------------------------------------------------------------------------------------------------------------------------------------------------

NC_012532      AUAGGGAGAGAGAACACCACCUGAGAGGAGAGUGUCACAGCUGUGUGUACAACAUGAUGGGAAAAAGAGAAAAGAAGCAAGGAGAGUUCGGGAAAGCAAAAGGUAGCCGCGCCAUCUGGUACAUGUGGUUGGGAGCCAGAUUCUUGGAGUUUGAAGCCCUUGGAUUCUUGAACGAGGACCA
KU922960       ACAAGGAAAGAGAGCACCACCUGAGAGGAGAGUGCCAGAGUUGUGUGUACAACAUGAUGGGAAAAAGAGAAAAGAAACAAGGGGAAUUUGGAAAGGCCAAGGGCAGCCGCGCCAUCUGGUAUAUGUGGCUAGGGGCUAGAUUUCUAGAGUUCGAAGCCCUUGGAUUCUUGAACGAGGAUCA
KU922923       ACAAGGAAAGAGAGCACCACCUGAGAGGAGAGUGCCAGAGUUGUGUGUACAACAUGAUGGGAAAAAGAGAAAAGAAACAAGGGGAAUUUGGAAAGGCCAAGGGCAGCCGCGCCAUCUGGUAUAUGUGGCUAGGGGCUAGAUUUCUAGAGUUCGAAGCCCUUGGAUUCUUGAACGAGGAUCA
KU820898       ACAAGGAAAGAGAGCACCACCUGAGAGGAGAGUGCCAGAGUUGUGUGUACAACAUGAUGGGAAAAAGAGAAAAGAAACAAGGGGAAUUUGGAAAGGCCAAGGGCAGCCGCGCCAUCUGGUAUAUGUGGCUAGGGGCUAGAUUUCUAGAGUUCGAAGCCCUUGGAUUCUUGAACGAGGAUCA
KU321639       ACAAGGAAAGAGAGCACCACCUGAGAGGAGAGUGCCAGAGUUGUGUGUACAACAUGAUGGGAAAAAGAGAAAAGAAACAAGGGGAAUUUGGAAAGGCCAAGGGCAGCCGCGCCAUCUGGUAUAUGUGGCUAGGGGCUAGAUUUCUAGAGUUCGAAGCCCUUGGAUUCUUGAACGAGGAUCA
KU820899       ACAAGGAAAGAGAGCACCACCUGAGAGGAGAGUGCCAGAGUUGUGUGUACAACAUGAUGGGAAAAAGAGAAAAGAAACAAGGGGAAUUUGGAAAGGCCAAGGGCAGCCGCGCCAUCUGGUAUAUGUGGCUAGGGGCUAGAUUUCUAGAGUUCGAAGCCCUUGGAUUCUUGAACGAGGAUCA
KU853012       ACAAGGAAAGAGAGCACCACCUGAGAGGAGAGUGCCAGAGUUGUGUGUACAACAUGAUGGGAAAAAGAGAAAAGAAACAAGGGGAAUUUGGAAAGGCCAAGGGCAGCCGCGCCAUCUGGUAUAUGUGGCUAGGGGCUAGAUUUCUAGAGUUCGAAGCCCUUGGAUUCUUGAACGAGGAUCA
KU729217       ACAAGGAAAGAGAGCACCACCUGAGAGGAGAGUGCCAGAGUUGUGUGUACAACAUGAUGGGAAAAAGAGAAAAGAAACAAGGGGAAUUUGGAAAGGCCAAGGGCAGCCGCGCCAUCUGGUAUAUGUGGCUAGGGGCUAGAUUUCUAGAGUUCGAAGCCCUUGGAUUCUUGAACGAGGAUCA
KU729218       ACAAGGAAAGAGAGCACCACCUGAGAGGAGAGUGCCAGAGUUGUGUGUACAACAUGAUGGGGAAAAGAGAAAAGAAACAAGGGGAAUUUGGAAAGGCCAAGGGCAGCCGCGCCAUCUGGUAUAUGUGGCUAGGGGCUAGAUUUCUAGAGUUCGAAGCCCUUGGAUUCUUGAACGAGGAUCA
KU761564       ACAAGGAAAGAGAGCACCACCUGAGAGGAGAGUGCCAGAGUUGUGUGUACAACAUGAUGGGAAAAAGAGAAAAGAAACAAGGGGAAUUUGGAAAGGCCAAGGGCAGCCGCGCCAUCUGGUAUAUGUGGCUGGGGGCUAGAUUUCUAGAGUUCGAAGCCCUUGGAUUCUUGAACGAGGAUCA
KU740184       ACAAGGAAAGAGAGCACCACCUGAGAGGAGAGUGCCAGAGUUGUGUGUACAACAUGAUGGGAAAAAGAGAAAAGAAACAAGGGGAAUUUGGAAAGGCCAAGGGCAGCCGCGCCAUCUGGUAUAUGUGGCUGGGGGCUAGAUUUCUAGAGUUCGAAGCCCUUGGAUUCUUGAACGAGGAUCA
KU744693       ACAAGGAAAGAGAGCACCACCUGAGAGGAGAGUGCCAGAGUUGUGUGUACAUCACAAUGGGAAAAAGAGAAAAGAAACAAGGGGAAUUUGGAAAGGCCAAGGGCAGCCGCGCCAUCUGGUAUAUGUGGCUAGGGGCUAGAUUUCUAGAGUUCGAAGCCCUUGGAUUCUUGAACGAGGAUCA
KU497555       ACAAGGAAAGAGAGCACCACCUGAGAGGAGAGUGCCAGAGUUGUGUGUACAACAUGAUGGGAAAAAGAGAAAAGAAACAAGGGGAAUUUGGAAAGGCCAAGGGCAGCCGCGCCAUCUGGUAUAUGUGGCUAGGGGCUAGAUUUCUAGAGUUCGAAGCCCUUGGAUUCUUGAACGAGGAUCA
KU527068       ACAAGGAAAGAGAGCACCACCUGAGAGGAGAGUGCCAGAGUUGUGUGUACAACAUGAUGGGAAAAAGAGAAAAGAAACAAGGGGAAUUUGGAAAGGCCAAGGGCAGCCGCGCCAUCUGGUAUAUGUGGCUAGGGGCUAGAUUUCUAGAGUUCGAAGCCCUUGGAUUCUUGAACGAGGAUCA
KU501217       ACAAGGAAAGAGAGCACCACCUGAGAGGAGAGUGCCAGAGUUGUGUGUACAACAUGAUGGGAAAAAGAGAAAAGAAACAAGGGGAAUUUGGAAAGGCCAAGGGCAGCCGCGCCAUCUGGUAUAUGUGGCUAGGGGCUAGAUUUCUAGAGUUCGAAGCCCUUGGAUUCUUGAACGAGGAUCA
KU501216       ACAAGGAAAGAGAGCACCACCUGAGAGGAGAGUGCCAGAGUUGUGUGUACAACAUGAUGGGAAAAAGAGAAAAGAAACAAGGGGAAUUUGGAAAGGCCAAGGGCAGCCGCGCCAUCUGGUAUAUGUGGCUAGGGGCUAGAUUUCUAGAGUUCGAAGCCCUUGGAUUCUUGAACGAGGAUCA
KU501215       ACAAGGAAAGAGAGCACCACCUGAGAGGAGAGUGCCAGAGCUGUGUGUACAACAUGAUGGGAAAAAGAGAAAAGAAACAAGGGGAAUUUGGAAAGGCCAAGGGCAGCCGCGCCAUCUGGUAUAUGUGGCUAGGGGCUAGAUUUCUAGAGUUCGAAGCCCUUGGAUUCUUGAACGAGGAUCA
KU365780       AUAAGGAAAGAGAGCACCACCUGAGAGGAGAGUGCCAGAGUUGUGUGUACAACAUGAUGGGAAAAAGAGAAAAGAAACAAGGGGAAUUUGGAAAGGCCAAGGGCAGCCGCGCCAUCUGGUAUAUGUGGCUAGGGGCUAGAUUUCUAGAGUUCGAAGCCCUUGGAUUCUUGAACGAGGAUCA
KU365779       AUAAGGAAAGAGAGCACCACCUGAGAGGAGAGUGCCAGAGUUGUGUGUACAACAUGAUGGGAAAAAGAGAAAAGAAACAAGGGGAAUUUGGAAAGGCCAAGGGCAGCCGCGCCAUCUGGUAUAUGUGGCUAGGGGCUAGAUUUCUAGAGUUCGAAGCCCUUGGAUUCUUGAACGAGGAUCA
KU707826       AUAAGGAAAGAGAGCACCACCUGAGAGGAGAGUGCCAGAGUUGUGUGUACAACAUGAUGGGAAAAAGAGAAAAGAAACAAGGGGAAUUUGGAAAGGCCAAGGGCAGCCGCGCCAUCUGGUAUAUGUGGCUAGGGGCUAGAUUUCUAGAGUUCGAAGCCCUUGGAUUCUUGAACGAGGAUCA
KU365778       ACAAGGAAAGAGAGCACCACCUGAGAGGAGAGUGCCAGAGUUGUGUGUAUAACAUGAUGGGAAAAAGAGAAAAGAAACAAGGGGAAUUUGGAAAGGCCAAGGGCAGCCGCGCCAUCUGGUAUAUGUGGCUAGGGGCUAGAUUUCUAGAGUUCGAAGCCCUUGGAUUCUUGAACGAGGAUCA
```

```
KU365777    AUAAGGAAAGAGAGCACCACCUGAGAGGAGAGUGCCAGAGUUGUGUGUACAACAUGAUGGGAAAAAGAGAAAAGAAACAAGGGGAAUUUGGAAAGGCCAAGGGCAGCCGCGCCAUCUGGUAUAUGUGGCUAGGGGCUAGAUUUCUAGAGUUCGAAGCCCUUGGAUUCUUGAACGAGGAUCA
KU312312    ACAAGGAAAGAGAGCACCACCUGAGAGGAGAGUGCCAGAGUUGUGUGUACAACAUGAUGGGAAAAAGAGAAAAGAAACAAGGGGAAUUUGGAAAGGCCAAGGGCAGCCGCGCCAUCUGGUAUAUGUGGCUAGGGGCUAGAUUUCUAGAGUUCGAAGCCCUUGGAUUCUUGAACGAGGAUCA
KF268950    ACAAGGAAAGAGAACAUCACCUGAGAGGAGAGUGCCAUAGCUGUGUGUACAAUAUGAUGGGAAAAAGAGAGAAGAAGCAAGGAGAAUUCGGAAAAGCAAAAGGCAGCCGCGCCAUCUGGUACAUGUGGUUGGGAGCCAGAUUCUUGGAGUUUGAAGCCCUUGGAUUCUUGAAUGAGGACCA
KF268949    ACAAGGAGAGAGAACAUCACCUGAGAGGAGAGUGCCAUAGCUGUGUGUACAAUAUGAUGGGAAAAAGAGAGAAGAAGCAAGGAGAAUUCGGAAAAGCAAAAGGCAGCCGCGCCAUCUGGUACAUGUGGUUGGGAGCCAGAUUCUUGGAGUUUGAAGCCCUUGGAUUCUUGAAUGAGGACCA
KF268948    ACAAGGAAAGAGAACAUCACCUGAGAGGAGAGUGUCAUAGCUGUGUGUACAACAUGAUGGGAAAAAGAGAGAAGAAGCAAGGAGAAUUCGGAAAAGCAAAAGGCAGCCGCGCCAUCUGGUACAUGUGGUUGGGAGCCAGAUUCUUGGAGUUUGAAGCCCUUGGAUUCUUGAAUGAGGACCA
KJ776791    ACAAGGAAAGAGAGCACCACCUGAGAGGAGAGUGCCAGAGUUGUGUGUACAACAUGAUGGGAAAAAGAGAAAAGAAACAAGGGGAAUUUGGAAAGGCCAAGGGCAGCCGCGCCAUCUGGUAUAUGUGGCUAGGGGCUAGAUUUCUAGAGUUCGAAGCCCUUGGAUUCUUGAACGAGGAUCA
KF383119    AUAGGGAGAGAGAACACCACCUGAGAGGAGAGUGUCACAGCUGUGUGUACAACAUGAUGGGAAAAAGAGAAAAGAAGCAAGGAGAGUUCGGGAAAGCAAAAGGUAGCCGCGCCAUCUGGUACAUGUGGUUGGGAGCCAGAUUCUUGGAGUUUGAAGCCCUUGGAUUCUUGAACGAGGACCA
KF383118    AUAGGGAGAGAGAACACCACCUGAGAGGAGAGUGUCACAGCUGUGUGUACAACAUGAUGGGAAAAAGAGAAAAGAAGCAAGGAGAGUUCGGGAAAGCAAAAGGUAGCCGCGCCAUCUGGUACAUGUGGUUGGGAGCCAGGUUUCUGGAGUUUGAAUCACUGGGGUUUCUGAAUGAAGAUCA
KF383117    ACAAGGAAAGAGAACACCACCUGAGAGGAGAGUGUCACAGCUGUGUGUACAACAUGAUGGGUAAAAGAGAAAAGAAGCAAGGAGAAUUCGGGAAAGCAAAAGGCAGCCGCGCAAUCUGGUACAUGUGGUUGGGAGCCAGAUUUUUGGAGUUUGAAGCUCUUGGAUUCUUGAACGAGGAUCA
KF383116    ACAAGGAAAGAGAACACCACCUGAGAGGAGAGUGUCACAGCUGUGUGUACAACAUGAUGGGUAAAAGAGAAAAGAAGCAAGGAGAAUUCGGGAAAGCAAAAGGCAGCCGCGCAAUCUGGUACAUGUGGUUGGGAGCCAGAUUUUUGGAGUUUGAAGCUCUUGGAUUCUUGAACGAGGACCA
KF383115    AUAAGGAGAGAGAGCAUCACCUAAGAGGAGAGUGUCAUAGCUGUGUGUACAACAUGAUGGGAAAAAGAGAGAAGAAG?AAGGAGAAUUCGGGAAAGCAAAAGGCAGCCGCGCCAUCUGGUACAUGUGGUUGGGAGCCAGGUUUCUGGAGUUUGAAUCACUGGGGUUUCUGAAUGAAGAUCA
EU545988    ACAAGGAAAGAGAGCACCACCUGAGAGGAGAGUGUCAGAGCUGUGUGUACAACAUGAUGGGAAAAAGAGAAAAGAAACAAGGGGAAUUUGGAAAGGCCAAGGGCAGCCGCGCCAUCUGGUAUAUGUGGCUAGGGGCUAGAUUCCUGGAGUUCGAAGCCCUUGGAUUCUUGAACGAGGAUCA
KU955589    ACAAGGAAAGAGAGCACCACCUGAGAGGAGAGUGCCAGAGUUGUGUGUACAACAUGAUGGGAAAAAGAGAAAAGAAACAAGGGGAAUUUGGAAAGGCCAAGGGCAGCCGCGCCAUCUGGUAUAUGUGGCUAGGGGCUAGAUUUCUAGAGUUCGAAGCCCUUGGAUUCUUGAACGAGGAUCA
KU955590    ACAAGGAAAGAGAGCACCACCUGAGAGGAGAGUGCCAGAGUUGUGUGUACAACAUGAUGGGAAAAAGAGAAAAGAAACAAGGGGAAUUUGGAAAGGCCAAGGGCAGCCGCGCCAUCUGGUAUAUGUGGCUGGGGGCUAGAUUUCUAGAGUUCGAAGCCCUUGGAUUCUUGAACGAGGAUCA
KU940228    ACAAGGAAAGAGAGCACCACCUGAGAGGAGAGUGCCAGAGUUGUGUGUACAACAUGAUGGGAAAAAGAGAAAAGAAACAAGGGGAAUUUGGAAAGGCCAAGGGCAGCCGCGCCAUCUGGUAUAUGUGGCUAGGGGCUAGAUUUCUAGAGUUCGAAGCCCUUGGAUUCUUGAACGAGGAUCA
KU991811    ACAAGGAAAGAGAGCACCACCUGAGAGGAGAGUGCCAGAGUUGUGUGUACAACAUGAUGGGAAAAAGAGAAAAGAAACAAGGGGAAUUUGGAAAGGCCAAGGGCAGCCGCGCCAUCUGGUAUAUGUGGCUAGGGGCUAGAUUUCUAGAGUUCGAAGCCCUUGGAUUCUUGAACGAGGAUCA
KU509998    ACAAGGAAAGAGAGCACCACCUGAGAGGAGAGUGCCAGAGUUGUGUGUACAACAUGAUGGGAAAAAGAGAAAAGAAACAAGGGGAAUUUGGAAAGGCCAAGGGCAGCCGCGCCAUCUGGUAUAUGUGGCUAGGGGCUAGAUUUCUAGAGUUCGAAGCCCUUGGAUUCUUGAACGAGGAUCA
KX056898    ACAAGGAAAGAGAGCACCACCUGAGAGGAGAGUGCCAGAGUUGUGUGUACAACAUGAUGGGAAAAAGAGAAAAGAAACAAGGGGAAUUUGGAAAGGCCAAGGGCAGCCGCGCCAUCUGGUAUAUGUGGCUGGGGGCUAGAUUUCUAGAGUUCGAAGCCCUUGGAUUCUUGAACGAGGAUCA
KX117076    ACAAGGAAAGAGAGCACCACCUGAGAGGAGAGUGCCAGAGUUGUGUGUACAACAUGAUGGGAAAAAGAGAAAAGAAACAAGGGGAAUUUGGAAAGGCCAAGGGCAGCCGCGCCAUCUGGUAUAUGUGGCUAGGGGCUAGAUUUCUAGAGUUCGAAGCCCUUGGAUUCUUGAACGAGGAUCA
KX185891    ACAAGGAAAGAGAGCACCACCUGAGAGGAGAGUGCCAGAGUUGUGUGUACAACAUGAUGGGAAAAAGAGAAAAGAAACAAGGGGAAUUUGGAAAGGCCAAGGGCAGCCGCGCCAUCUGGUAUAUGUGGCUAGGGGCUAGAUUUCUAGAGUUCGAAGCCCUUGGAUUCUUGAACGAGGAUCA
KX247646    ACAAGGAAAGAGAGCACCACCUGAGAGGAGAGUGCCAGAGUUGUGUGUACAACAUGAUGGGAAAAAGAGAAAAGAAACAAGGGGAAUUUGGAAAGGCCAAGGGCAGCCGCGCCAUCUGGUAUAUGUGGCUAGGGGCUAGAUUUCUAGAGUUCGAAGCCCUUGGAUUCUUGAACGAGGAUCA
KX262887    ACAAGGAAAGAGAGCACCACCUGAGAGGAGAGUGCCAGAGUUGUGUGUACAACAUGAUGGGAAAAAGAGAAAAGAAACAAGGGGAAUUUGGAAAGGCCAAGGGCAGCCGCGCCAUCUGGUAUAUGUGGCUAGGGGCUAGAUUUCUAGAGUUCGAAGCCCUUGGAUUCUUGAACGAGGAUCA
KX280026    ACAAGGAAAGAGAGCACCACCUGAGAGGAGAGUGCCAGAGUUGUGUGUACAACAUGAUGGGAAAAAGAGAAAAGAAACAAGGGGAAUUUGGAAAGGCCAAGGGCAGCCGCGCCAUCUGGUAUAUGUGGCUAGGGGCUAGAUUUCUAGAGUUCGAAGCCCUUGGAUUCUUGAACGAGGAUCA
KU758877    ACAAGGAAAGAGAGCACCACCUGAGAGGAGAGUGCCAGAGUUGUGUGUACAACAUGAUGGGAAAAAGAGAAAAGAAACAAGGGGAAUUUGGAAAGGCCAAGGGCAGCCGCGCCAUCUGGUAUAUGUGGCUAGGGGCUAGAUUUCUAGAGUUCGAAGCCCUUGGAUUCUUGAACGAGGAUCA
KU866423    ACAAGGAAAGAGAGCACCACCUGAGAGGAGAGUGCCAGAGUUGUGUGUACAACAUGAUGGGAAAAAGAGAAAAGAAACAAGGGGAAUUUGGAAAGGCCAAGGGCAGCCGCGCCAUCUGGUAUAUGUGGCUAGGGGCUAGAUUUCUAGAGUUCGAAGCCCUUGGAUUCUUGAACGAGGAUCA
KX377336    ACAAGGAAAGAGAGCACCACUUGAGAGGAGAGUGUCAGAGCUGUGUGUACAACAUGAUGGGAAAAAGAGAAAAGAAGCAAGGGGAAUUUGGAAAGGCCAAGGGCAGCCGCGCCAUUUGGUACAUGUGGCUAGGGGCUAGAUUUCUAGAGUUUGAAGCCCUUGGAUUCUUGAACGAGGAUCA
KX377337    ACAAGGAAAGAGAGCACCACCUGAGAGGAGAGUGCCAGAGCUGUGUGUACAACAUGAUGGGAAAAAGAGAAAAGAAACAAGGGGAAUUUGGAAAGGCCAAGGGCAGCCGCGCCAUCUGGUAUAUGUGGCUAGGGGCUAGAUUUCUAGAGUUCGAAGCCCUUGGAUUCUUGAACGAGGAUCA
KX369547    ACAAGGAAAGAGAGCACCACCUGAGAGGAGAGUGCCAGAGUUGUGUGUACAACAUGAUGGGAAAAAGAGAAAAGAAACAAGGGGAAUUUGGAAAGGCCAAGGGCAGCCGCGCCAUCUGGUAUAUGUGGCUAGGGGCUAGAUUUCUAGAGUUCGAAGCCCUUGGAUUCUUGAACGAGGAUCA
KX548902    ACAAGGAAAGAGAGCACCACCUGAGAGGAGAGUGCCAGAGUUGUGUGUACAACAUGAUGGGAAAAAGAGAAAAGAAACAAGGGGAAUUUGGAAAGGCCAAGGGCAGCCGCGCCAUCUGGUAUAUGUGGCUAGGGGCUAGAUUUCUAGAGUUCGAAGCCCUUGGAUUCUUGAACGAGGAUCA
KX601166    ACAAGGAAAGAGAACACCACCUGAGAGGAGAGUGUCAUAGCUGUGUGUACAACAUGAUGGGCAAAAGAGAAAAGAAGCAAGGAGAAUUCGGGAAAGCAAAGGGCAGCCGCGCAAUCUGGUACAUGUGGUUGGGAGCCAGAUUUUUGGAGUUUGAAGCUCUUGGGUUCUUGAACGAGGACCA
KX601167    ACAAGGAAAGAGAGCACCACUUGAGAGGAGAGUGUCAGAGCUGUGUGUACAACAUGAUGGGAAAAAGAGAAAAGAAGCAAGGGGAAUUUGGAAAGGCCAAGGGCAGCCGCGCCAUUUGGUACAUGUGGCUAGGGGCUAGAUUUCUAGAGUUUGAAGCCCUUGGAUUCUUGAACGAGGAUCA
KX601168    ACAAGGAAAGAGAGCACCACCUGAGAGGAGAGUGCCAGAGCUGUGUGUACAACAUGAUGGGAAAAAGAGAAAAGAAACAAGGGGAAUUUGGAAAGGCCAAGGGCAGCCGCGCCAUCUGGUAUAUGUGGCUAGGGGCUAGAUUUCUAGAGUUCGAAGCCCUUGGAUUCUUGAACGAGGAUCA
KX766028    ACAAGGAAAGAGAGCACCACCUGAGAGGAGAGUGCCAGAGUUGUGUGUACAACAUGAUGGGAAAAAGAGAAAAGAAACAAGGGGAAUUUGGAAAGGCCAAGGGCAGCCGCGCCAUCUGGUAUAUGUGGCUGGGGGCUAGAUUUCUAGAGUUCGAAGCCCUUGGAUUCUUGAACGAGGAUCA
KX766029    ACAAGGAAAGAGAGCACCACCUGAGAGGAGAGUGCCAGAGUUGUGUGUACAACAUGAUGGGAAAAAGAGAAAAGAAACAAGGGGAAUUUGGAAAGGCCAAGGGCAGCCGCGCCAUCUGGUAUAUGUGGCUAGGGGCUAGAUUUCUAGAGUUCGAAGCCCUUGGAUUCUUGAACGAGGAUCA
KU940224    ACAAGGAAAGAGAGCACCACCUGAGAGGAGAGUGCCAGAGUUGUGUGUACAACAUGAUGGGAAAAAGAGAAAAGAAACAAGGGGAAUUUGGAAAGGCCAAGGGCAGCCGCGCCAUCUGGUAUAUGUGGCUAGGGGCUAGAUUUCUAGAGUUCGAAGCCCUUGGAUUCUUGAACGAGGAUCA


            9999999999999999999999999999999999999999999999999999999999999999999999999999999999999999999999999999999999999999999999999999999999999999999999999999999999999999999999999999999
            0000000000000000000000000000000000000000000000000111111111111111111111111111111111111111111111111111111111111111111111111111111111111111111111111111111222222222222222222222222222222222
            5555555556666666666777777777788888888889999999999000000000011111111112222222222333333333344444444445555555555666666666677777777778888888888999999999900000000001111111111222222222233
Sequence    1234567890123456789012345678901234567890123456789012345678901234567890123456789012345678901234567890123456789012345678901234567890123456789012345678901234567890123456789012345678901
-----------------------------------------------------------------------------------------------------------------------------------------------------------------------------------------------------
NC_012532   UUGGAUGGGAAGAGAAAACUCAGGAGGUGGAGUCGAAGGGUUAGGAUUGCAAAGACUUGGAUACAUUCUAGAAGAAAUGAAUCGGGCACCAGGAGGAAAGAUGUACGCAGAUGACACUGCUGGCUGGGACACCCGCAUUAGUAAGUUUGAUCUGGAGAAUGAAGCUCUGAUUACCAACCAA
KU922960    CUGGAUGGGGAGAGAGAACUCAGGAGGUGGUGUUGAAGUGCUGGGAUUACAGAGACUCGGAUAUGUCCUAGAAGAGAUGAGUCGCAUACCAGGAGGAAGGAUGUAUGCAGAUGACACUGCUGGCUGGGACACCCGCAUCAGCAGGUUUGAUCUGGAGAAUGAAGCUCUAAUCACCAACCAA
KU922923    CUGGAUGGGGAGAGAGAACUCAGGAGGUGGUGUUGAAGUGCUGGGAUUACAGAGACUCGGAUAUGUCCUAGAAGAGAUGAGUCGCAUACCAGGAGGAAGGAUGUAUGCAGAUGACACUGCUGGCUGGGACACCCGCAUCAGCAGGUUUGAUCUGGAGAAUGAAGCUCUAAUCACCAACCAA
KU820898    CUGGAUGGGGAGAGAGAACUCAGGAGGUGGUGUUGAAGGGCUGGGAUUACAAAGACUCGGAUAUGUCCUAGAAGAGAUGAGUCGUAUACCAGGAGGAAGGAUGUAUGCAGAUGACACUGCUGGCUGGGACACCCGCAUUAGCAGGUUUGAUCUGGAGAAUGAAGCUCUAAUCACCAACCAA
KU321639    CUGGAUGGGGAGAGAGAACUCAGGAGGUGGUGUUGAAGGGCUGGGAUUACAAAGACUCGGAUAUGUCCUAGAAGAGAUGAGUCGCAUACCAGGAGGAAGGAUGUAUGCAGAUGACACUGCUGGCUGGGACACCCGCAUCAGCAGGUUUGAUCUGGAGAAUGAAGCUCUAAUCACCAACCAA
KU820899    CUGGAUGGGGAGAGAGAACUCAGGAGGUGGUGUUGAAGGGCUGGGAUUACAAAGACUCGGAUAUGUCCUAGAAGAGAUGAGUCGCAUACCAGGAGGAAGGAUGUAUGCAGAUGACACUGCUGGCUGGGACACCCGCAUCAGCAGGUUUGAUCUGGAGAAUGAAGCUCUAAUCACCAACCAA
KU853012    CUGGAUGGGGAGAGAGAACUCAGGAGGUGGUGUUGAAGGGCUGGGAUUACAAAGACUCGGAUAUGUCCUAGAAGAGAUGAGUCGCAUACCAGGAGGAAGGAUGUAUGCAGAUGACACUGCUGGCUGGGAUACCCGCAUCAGCAGGUUUGAUCUAGAGAAUGAAGCUCUAAUCACCAACCAA
KU729217    CUGGAUGGGGAGAGAGAACUCAGGAGGUGGUGUUGAAGGGCUGGGAUUACAAAGACUCGGAUAUGUCCUAGAAGAGAUGAGUCGCAUACCAGGAGGAAGGAUGUAUGCAGAUGACACUGCUGGCUGGGACACCCGCAUCAGCAGGUUUGAUCUGGAGAAUGAAGCUCUAAUCACCAACCAA
KU729218    CUGGAUGGGGAGAGAGAACUCAGGAGGUGGUGUUGAAGGGCUGGGAUUACAAAGACUCGGAUAUGUCCUAGAAGAGAUGAGUCGCAUACCAGGAGGAAGGAUGUAUGCAGAUGACACUGCUGGCUGGGACACCCGCAUCAGCAGGUUUGAUCUGGAGAAUGAAGCUCUAAUCACCAACCAA
KU761564    CUGGAUGGGGAGAGAGAACUCAGGAGGUGGUGUUGAAGGGCUGGGAUUACAAAGACUCGGAUAUGUCCUAGAAGAGAUGAGUCGUAUACCAGGAGGAAGGAUGUAUGCAGAUGACACUGCUGGCUGGGACACCCGCAUUAGCAGGUUUGAUCUGGAGAAUGAAGCUCUAAUCACCAACCAA
KU740184    CUGGAUGGGGAGAGAGAACUCAGGAGGUGGUGUUGAAGGGCUGGGAUUACAAAGACUCGGAUAUGUCCUAGAAGAGAUGAGUCGUAUACCAGGAGGAAGGAUGUAUGCAGAUGACACUGCUGGCUGGGACACCCGCAUUAGCAGGUUUGAUCUGGAGAAUGAAGCUCUAAUCACCAACCAA
KU744693    CUGGAUGGGGAGAGAGAACUCAGGAGGUGGUGUUGAAGGGCUGGGAUUACAAAGACUCGGAUAUGUCCUAGAAGAGAUGAGUCGCAUACCAGGAGGAAGGAUGUAUGCAGAUGACACUGCUGGCUGGGACACCCGCAUCAGCAGGUUUGAUCUGGAGAAUGAAGCUCUAAUCACCAACCAA
KU497555    CUGGAUGGGGAGAGAGAACUCAGGAGGUGGUGUUGAAGGGCUGGGAUUACAAAGACUCGGAUAUGUCCUAGAAGAGAUGAGUCGCAUACCAGGAGGAAGGAUGUAUGCAGAUGACACUGCUGGCUGGGACACCCGCAUCAGCAGGUUUGAUCUGGAGAAUGAAGCUCUAAUCACCAACCAA
KU527068    CUGGAUGGGGAGAGAGAACUCAGGAGGUGGUGUUGAAGGGCUGGGAUUACAAAGACUCGGAUAUGUCCUAGAAGAGAUGAGUCGCAUACCAGGAGGAAGGAUGUAUGCAGAUGACACUGCUGGCUGGGACACCCGCAUCAGCAGGUUCGAUCUGGAGAAUGAAGCUCUAAUCACCAACCAA
KU501217    CUGGAUGGGGAGAGAGAACUCAGGAGGUGGUGUUGAAGGGCUGGGAUUACAAAGACUCGGAUAUGUCCUAGAAGAGAUGAGUUGCAUACCAGGAGGAAGGAUGUAUGCAGAUGACACUGCUGGCUGGGACACCCGCAUCAGCAGGUUUGAUCUGGAGAAUGAAGCUCUAAUCACCAACCAA
KU501216    CUGGAUGGGGAGAGAGAACUCAGGAGGUGGUGUUGAAGGGCUGGGAUUACAAAGACUCGGAUAUGUCCUAGAAGAGAUGAGUUGCAUACCAGGAGGAAGGAUGUAUGCAGAUGACACUGCUGGCUGGGACACCCGCAUCAGCAGGUUUGAUCUGGAGAAUGAAGCUCUAAUCACCAACCAA
KU501215    CUGGAUGGGGAGAGAGAACUCAGGAGGUGGUGUUGAAGGGCUGGGAUUACAAAGACUCGGAUAUGUCCUAGAAGAGAUGAGUCGUAUACCAGGAGGAAGGAUGUAUGCAGAUGACACUGCUGGCUGGGACACCCGCAUUAGCAGGUUUGAUCUGGAGAAUGAAGCUCUAAUCACCAACCAA
KU365780    CUGGAUGGGGAGAGAGAACUCAGGAGGUGGUGUUGAAGGGCUGGGAUUACAAAGACUCGGAUAUGUCCUAGAAGAGAUGAGUCGUAUACCAGGAGGAAGGAUGUAUGCAGAUGACACUGCUGGCUGGGACACCCGCAUCAGCAGGUUUGAUCUGGAGAAUGAAGCUCUAAUCACCAACCAA
KU365779    CUGGAUGGGGAGAGAGAACUCAGGAGGUGGUGUUGAAGGGCUGGGAUUACAAAGACUCGGAUAUGUCCUAGAAGAGAUGAGUCGUAUACCAGGAGGAAGGAUGUAUGCAGAUGACACUGCUGGCUGGGACACCCGCAUCAGCAGGUUUGAUCUGGAGAAUGAAGCUCUAAUCACCAACCAA
KU707826    CUGGAUGGGGAGAGAGAACUCAGGAGGUGGUGUUGAAGGGCUGGGAUUACAAAGACUCGGAUAUGUCCUAGAAGAGAUGAGUCGUAUACCAGGAGGAAGGAUGUAUGCAGAUGACACUGCUGGCUGGGACACCCGCAUCAGCAGGUUUGAUCUGGAGAAUGAAGCUCUAAUCACCAACCAA
KU365778    CUGGAUGGGGAGAGAGAACUCAGGAGGUGGUGUUGAAGGGCUGGGAUUACAAAGACUCGGAUAUGUCCUAGAAGAGAUGAGUCGUAUACCAGGAGGAAGGAUGUAUGCAGAUGACACUGCUGGCUGGGACACCCGCAUUAGCAGGUUUGAUCUGGAGAAUGAAGCUCUAAUCACCAACCAA
KU365777    CUGGAUGGGGAGAGAGAACUCAGGAGGUGGUGUUGAAGGGCUGGGAUUACAAAGACUCGGAUAUGUCCUAGAAGAGAUGAGUCGUAUACCAGGAGGAAGGAUGUAUGCAGAUGACACUGCUGGCUGGGACACCCGCAUCAGCAGGUUUGAUCUGGAGAAUGAAGCUCUAAUCACCAACCAA
KU312312    CUGGAUGGGGAGAGAGAACUCAGGAGGUGGUGUUGAAGGGCUGGGAUUACAAAGACUCGGAUAUGUCCUAGAAGAGAUGAGUCGUAUACCAGGAGGAAGGAUGUAUGCAGAUGACACUGCUGGCUGGGACACCCGCAUUAGCAGGUUUGAUCUGGAGAAUGAAGCUCUAAUCACCAACCAA
KF268950    UUGGAUGGGAAGAGAAAACUCAGGAGGUGGUGUCGAAGGGCUAGGACUGCAAAGACUUGGAUACAUUCUAGAAGAAAUGAAUCGGGCACCAGGAGGAAAGAUGUAUGCAGAUGACACCGCUGGCUGGGACACCCGCAUUAGUAAGUUUGAUCUGGAGAAUGAAGCCUUAAUUACCAACCAA
KF268949    UUGGAUGGGAAGAGAAAACUCAGGAGGUGGUGUCGAAGGGCUAGGACUGCAAAGACUUGGAUACAUUCUAGAAGAAAUGAAUCGAGCACCAGGAGGAAAGAUGUAUGCAGAUGACACCGCUGGCUGGGAUACCCGUAUUAGUAAGUUUGAUCUGGAGAAUGAAGCCUUAAUUACCAACCAA
KF268948    UUGGAUGGGAAGAGAAAACUCAGGAGGUGGUGUCGAAGGGCUAGGACUGCAAAGACUUGGAUACAUUCUAGAAGAAAUGAAUCGGGCACCAGGAGGAAAGAUGUAUGCAGAUGACACCGCUGGCUGGGACACCCGCAUUAGUAAGUUUGAUCUGGAGAAUGAAGCCUUAAUUACCAACCAA
KJ776791    CUGGAUGGGGAGAGAGAACUCAGGAGGUGGUGUUGAAGGGCUGGGAUUACAAAGACUCGGAUAUGUCCUAGAAGAGAUGAGUCGCAUACCAGGAGGAAGGAUGUAUGCAGAUGACACUGCUGGCUGGGACACCCGCAUCAGCAGGUUUGAUCUGGAGAAUGAAGCUCUAAUCACCAACCAA
KF383119    UUGGAUGGGAAGAGAGAACUCUGGAGGCGGAGUUGAAGGACUGGGACUGCAGAGACUGGGCUAUGUCCUUGAGGAGAUGAGCCAGGCACCAGGAGGGAAGAUGUACGCAGAUGACACUGCUGGCUGGGACACCCGCAUUAGUAAGUUUGAUCUGGAGAAUGAAGCUUUGAUUACCAACCAA
KF383118    CUGGAUGGGAAGAGAGAACUCUGGAGGCGGAGUUGAAGGACUGGGACUGCAGAGACUGGGCUAUGUCCUUGAGGAGAUGAGCCAGGCACCAGGAGGGAAGAUGUACGCAGAUGACACUGCUGGCUGGGACACCCGCAUUAGUAAGUUUGAUCUGGAGAAUGAAGCUUUGAUUACCAACCAA
KF383117    CUGGAUGGGAAGAGAAAACUCAGGAGGUGGUGUUGAAGGGCUAGGACUGCAAAGGCUUGGAUAUAUCCUAGAAGAAAUGAACCGGGCACCAGGAGGAAAGAUGUAUGCAGAUGACACCGCUGGCUGGGACACCCGUAUUAGUAAGUUUGAUCUAGAGAAUGAAGCCCUGAUCACUAACCAG
KF383116    CUGGAUGGGAAGAGAAAACUCAGGAGGUGGCGUUGAAGGGCUAGGACUGCAAAGGCUUGGAUAUAUCCUAGAAGAAAUGAACCGGGCACCAGGAGGAAAGAUGUAUGCAGAUGACACCGCUGGCUGGGACACCCGCAUUAGCAAGUUUGAUCUAGAGAAUGAAGCCCUGAUCACUAACCAG
KF383115    CUGGAUGGGAAGAGAGAACUCAGGAGGUGGUGUCGAAGGGCUAGGACUGCAAAGACUUGGAUAUAUUCUAGAAGAAAUGAAUCGGGCACCAGGAGGAAAGAUGUAUGCAGAUGACACCGCUGGUUGGGAUACCCGCAUUAGUAAGUUUGAUCUGGAGAAUGAAGCCUUGAUUACCAACCAA
EU545988    CUGGAUGGGGAGAGAGAAUUCAGGAGGUGGUGUUGAAGGGCUGGGAUUACAAAGACUCGGAUAUGUCCUAGAAGAGAUGAGUCGCAUACCAGGAGGAAGGAUGUAUGCUGAUGACACAGCUGGCUGGGACACCCGCAUCAGCAGGUUUGAUCUGGAGAAUGAAGCUCUAAUCACCAACCAA
KU955589    CUGGAUGGGGAGAGAGAACUCAGGAGGUGGUGUUGAAGGGCUGGGAUUACAAAGACUCGGAUAUGUCCUAGAAGAGAUGAGUCGCAUACCAGGAGGAAGGAUGUAUGCAGAUGACACUGCUGGCUGGGACACCCGCAUCAGCAGGUUUGAUCUGGAGAAUGAAGCUCUAAUCACCAACCAA
KU955590    CUGGAUGGGGAGAGAGAACUCAGGAGGUGGUGUUGAAGGGCUGGGAUUACAAAGACUCGGAUAUGUCCUAGAAGAGAUGAGUCGUAUACCAGGAGGAAGGAUGUAUGCAGAUGACACUGCUGGCUGGGACACCCGCAUUAGCAGGUUUGAUCUGGAGAAUGAAGCUCUAAUCACCAACCAA
KU940228    CUGGAUGGGGAGAGAGAACUCAGGAGGUGGUGUUGAAGGGCUGGGAUUACAAAGACUCGGAUAUGUCCUAGAAGAGAUGAGUCGCAUACCAGGAGGAAGGAUGUAUGCAGAUGACACUGCUGGCUGGGACACCCGCAUCAGCAGGUUUGAUCUGGAGAAUGAAGCUCUAAUCACCAACCAA
```

```
KU991811    CUGGAUGGGGAGAGAGAACUCAGGAGGUGGUGUUGAAGGGCUGGGAUUACAAAGACUCGGAUAUGUCCUAGAAGAGAUGAGUCGCAUACCAGGAGGAAGGAUGUAUGCAGAUGACACUGCUGGCUGGGAUACCCGCAUCAGCAGGUUUGAUCUGGAGAAUGAAGCUCUAAUCACCAACCAA
KU509998    CUGGAUGGGGAGAGAGAACUCAGGAGGUGGUGUUGAAGGGCUGGGAUUACAAAGACUCGGAUAUGUCCUAGAAGAGAUGAGUCGCAUACCAGGAGGAAGGAUGUAUGCAGAUGACACUGCUGGCUGGGACACCCGCAUCAGCAGGUUUGAUCUGGAGAAUGAAGCUCUAAUCACCAACCAA
KX056898    CUGGAUGGGGAGAGAGAACUCAGGAGGUGGUGUUGAAGGGCUGGGAUUACAAAGACUCGGAUAUGUCCUAGAAGAGAUGAGUCGUAUACCAGGAGGAAGGAUGUAUGCAGAUGACACUGCUGGCUGGGACACCCGCAUUAGCAGGUUUGAUCUGGAGAAUGAAGCUCUAAUCACCAACCAA
KX117076    CUGGAUGGGGAGAGAGAACUCAGGAGGUGGUGUUGAAGGGCUGGGAUUACAAAGACUCGGAUAUGUCCUAGAAGAGAUGAGUCGCAUACCAGGAGGAAGGAUGUAUGCAGAUGACACUGCUGGCUGGGACACCCGCAUCAGCAGGUUUGAUCUGGAGAAUGAAGCUCUAAUCACCAACCAA
KX185891    CUGGAUGGGGAGAGAGAACUCAGGAGGUGGUGUUGAAGGGCUGGGAUUACAAAGACUCGGAUAUGUCCUAGAAGAGAUGAGUCGCAUACCAGGAGGAAGGAUGUAUGCAGAUGACACUGCUGGCUGGGACACCCGCAUCAGCAGGUUUGAUCUGGAGAAUGAAGCUCUAAUCACCAACCAA
KX247646    CUGGAUGGGGAGAGAGAACUCAGGAGGUGGUGUUGAAGGGCUGGGAUUACAAAGACUCGGAUAUGUCCUAGAAGAGAUGAGUCGCAUACCAGGAGGAAGGAUGUAUGCAGAUGACACUGCUGGCUGGGACACCCGCAUUAGCAGGUUUGAUCUGGAGAAUGAAGCUCUAAUCACCAACCAA
KX262887    CUGGAUGGGGAGAGAGAACUCAGGAGGUGGUGUUGAAGGGCUGGGAUUACAAAGACUCGGAUAUGUCCUAGAAGAGAUGAGUUGCAUACCAGGAGGAAGGAUGUAUGCAGAUGACACUGCUGGCUGGGACACCCGCAUCAGCAGGUUUGAUCUGGAGAAUGAAGCUCUAAUCACCAACCAA
KX280026    CUGGAUGGGGAGAGAGAACUCAGGAGGUGGUGUUGAAGGGCUGGGAUUACAAAGACUCGGAUAUGUCCUAGAAGAGAUGAGUCGCAUACCAGGAGGAAGGAUGUAUGCAGAUGACACUGCUGGCUGGGACACCCGCAUCAGCAGGUUUGAUCUGGAGAAUGAAGCUCUAAUCACCAACCAA
KU758877    CUGGAUGGGGAGAGAGAACUCAGGAGGUGGUGUUGAAGGGCUGGGAUUACAAAGACUCGGAUAUGUCCUAGAAGAGAUGAGUCGUAUACCAGGAGGAAGGAUGUAUGCAGAUGACACUGCUGGCUGGGACACCCGCAUUAGCAGGUUUGAUCUGGAGAAUGAAGCUCUAAUCACCAACCAA
KU866423    CUGGAUGGGGAGAGAGAACUCAGGAGGUGGUGUUGAAGGGCUGGGAUUACAAAGACUCGGAUAUGUCCUAGAAGAGAUGAGUCGCAUACCAGGAGGAAGGAUGUAUGCAGAUGACACUGCUGGCUGGGACACCCGCAUCAGCAGGUUUGAUCUGGAGAAUGAAGCUCUAAUCACCAACCAA
KX377336    CUGGAUGGGGAGAGAGAAUUCAGGAGGUGGUGUUGAAGGGCUGGGAUUACAAAGACUUGGAUAUGUUCUAGAAGAAAUGAGCCGCACACCAGGAGGAAAGAUGUAUGCAGAUGAUACCGCUGGCUGGGACACCCGCAUCAGUAGGUUUGAUCUGGAGAAUGAAGCUCUGAUCACCAACCAA
KX377337    CUGGAUGGGGAGAGAGAACUCAGGAGGUGGUGUUGAAGGGCUGGGAUUACAAAGACUCGGAUAUGUCCUAGAAGAGAUGAGUCGUAUACCAGGAGGAAGGAUGUAUGCAGAUGACACUGCUGGCUGGGACACCCGCAUUAGCAGGUUUGAUCUGGAGAAUGAAGCUCUAAUCACCAACCAA
KX369547    CUGGAUGGGGAGAGAGAACUCAGGAGGUGGUGUUGAAGGGCUGGGAUUACAAAGACUCGGAUAUGUCCUAGAAGAGAUGAGUCGCAUACCAGGAGGAAGGAUGUAUGCAGAUGACACUGCUGGCUGGGACACCCGCAUCAGCAGGUUUGAUCUGGAGAAUGAAGCUCUAAUCACCAACCAA
KX548902    CUGGAUGGGGAGAGAGAACUCAGGAGGUGGUGUUGAAGGGCUGGGAUUACAAAGACUCGGAUAUGUCCUAGAAGAGAUGAGUCGCAUACCAGGAGGAAGGAUGUAUGCAGAUGACACUGCUGGCUGGGACACCCGCAUCAGCAGGUUUGAUCUGGAGAAUGAAGCUCUAAUCACCAACCAA
KX601166    CUGGAUGGGGAGAGAAAACUCAGGAGGUGGCGUUGAAGGGCUAGGACUGCAAAGGCUUGGAUAUAUCCUAGAAGAAAUGAACCGGGCACCAGGAGGAAAGAUGUAUGCAGAUGACACUGCUGGCUGGGACACCCGCAUUAGCAAGUUUGAUCUAGAGAAUGAAGCCCUGAUCACUAACCAG
KX601167    CUGGAUGGGGAGAGAGAAUUCAGGAGGUGGUGUUGAAGGGCUGGGAUUACAAAGACUUGGAUAUGUUCUAGAAGAAAUGAGCCGCACACCAGGAGGAAAGAUGUAUGCAGAUGAUACCGCUGGCUGGGACACCCGCAUCAGUAGGUUUGAUCUGGAGAAUGAAGCUCUGAUCACCAACCAA
KX601168    CUGGAUGGGGAGAGAGAACUCAGGAGGUGGUGUUGAAGGGCUGGGAUUACAAAGACUCGGAUAUGUCCUAGAAGAGAUGAGUCGUAUACCAGGAGGAAGGAUGUAUGCAGAUGACACUGCUGGCUGGGACACCCGCAUUAGCAGGUUUGAUCUGGAGAAUGAAGCUCUAAUCACCAACCAA
KX766028    CUGGAUGGGGAGAGAGAACUCAGGAGGUGGUGUUGAAGGGCUGGGAUUACAAAGACUCGGAUAUGUCCUAGAAGAGAUGAGUCGUAUACCAGGAGGAAGGAUGUAUGCAGAUGACACUGCUGGCUGGGACACCCGCAUUAGCAGGUUUGAUCUGGAGAAUGAAGCUCUAAUCACCAACCAA
KX766029    CUGGAUGGGGAGAGAGAACUCAGGAGGUGGUGUUGAAGGGCUGGGAUUACAAAGACUCGGAUAUGUCCUAGAAGAGAUGAGUCGCACACCAGGAGGAAGGAUGUAUGCAGAUGACACUGCUGGCUGGGACACCCGCAUCAGCAGGUUUGACCUGGAGAAUGAAGCUCUAAUCACCAACCAA
KU940224    CUGGAUGGGGAGAGAGAACUCAGGAGGUGGUGUUGAAGGGCUGGGAUUACAAAGACUCGGAUAUGUCCUAGAAGAGAUGAGUCGCAUACCAGGAGGAAGGAUGUAUGCAGAUGACACUGCUGGCUGGGACACCCGCAUCAGCAGGUUUGAUCUGGAGAAUGAAGCUCUAAUCACCAACCAA


            9999999999999999999999999999999999999999999999999999999999999999999999999999999999999999999999999999999999999999999999999999999999999999999999999999999999999999999999999999999
            2222222222222222222222222222222222222222222222222222222222222222222233333333333333333333333333333333333333333333333333333333333333333333333333333333333333333333333334444444444444
            3333333344444444445555555555666666666677777777778888888888999999999900000000001111111111222222222233333333334444444444555555555566666666667777777777888888888899999999990000000000111
Sequence    2345678901234567890123456789012345678901234567890123456789012345678901234567890123456789012345678901234567890123456789012345678901234567890123456789012345678901234567890123456789012
-----------------------------------------------------------------------------------------------------------------------------------------------------------------------------------------------------------
NC_012532   AUGGAGGAAGGGCACAGAACUCUGGCGUUGGCCGUGAUUAAAUACACAUACCAAAACAAAGUGGUGAAGGUUCUCAGACCAGCUGAAGGAGGAAAAACAGUUAUGGACAUCAUUUCAAGACAAGACCAGAGAGGGAGUGGACAAGUUGUCACUUAUGCUCUCAACACAUUCACCAACUUGG
KU922960    AUGGAGAAAGGGCACAGGGCCUUGGCAUUGGCCAUAAUCAAGUACACAUACCAAAACAAAGUGGUAAAGGUCCUUAGACCAGCUGAAAAAGGGAAAACAGUUAUGGACAUUAUUUCGAGACAAGACCAAAGGGGGAGCGGACAAGUUGUCACUUACGCUCUUAACACAUUUACCAACCUAG
KU922923    AUGGAGAAAGGGCACAGGGCCUUGGCAUUGGCCAUAAUCAAGUACACAUACCAAAACAAAGUGGUAAAGGUCCUUAGACCAGCUGAAAAAGGGAAAACAGUUAUGGACAUUAUUUCGAGACAAGACCAAAGGGGGAGCGGACAAGUUGUCACUUACGCUCUUAACACAUUUACCAACCUAG
KU820898    AUGGAGAAAGGGCACAGGGCCUUGGCAUUGGCCAUAAUCAAGUACACAUACCAAAACAAAGUGGUAAAGGUCCUUAGACCAGCUGAAAAAGGGAAAACAGUUAUGGACAUUAUUUCGAGACAAGACCAAAGGGGGAGCGGACAAGUUGUCACUUACGCUCUUAACACAUUUACCAACCUAG
KU321639    AUGGAGAAAGGGCACAGGGCCUUGGCAUUGGCCAUAAUCAAGUACACAUACCAAAACAAAGUGGUAAAGGUCCUUAGACCAGCUGAAAAAGGGAAAACAGUUAUGGACAUUAUUUCGAGACAAGACCAAAGGGGGAGCGGACAAGUUGUCACUUACGCUCUUAACACAUUUACCAACCUAG
KU820899    AUGGAGAAAGGGCACAGGGCCUUGGCAUUGGCCAUAAUCAAGUACACAUACCAAAACAAAGUGGUAAAGGUCCUUAGACCAGCUGAAAAAGGGAAGACAGUUAUGGACAUUAUUUCGAGACAAGACCAAAGGGGGAGCGGACAAGUUGUCACUUACGCUCUUAACACAUUUACCAACCUAG
KU853012    AUGGAGAAAGGGCACAGGGCCUUGGCAUUGGCCAUAAUCAAGUACACAUACCAAAACAAAGUGGUAAAGGUCCUUAGACCAGCUGAAAAAGGGAAAACAGUUAUGGACAUUAUUUCGAGACAAGACCAAAGGGGGAGCGGACAAGUUGUCACUUACGCUCUUAACACAUUUACCAACCUAG
KU729217    AUGGAGAAAGGGCACAGGGCCUUGGCACUGGCCAUAAUCAAGUACACAUACCAAAACAAAGUGGUAAAGGUCCUUAGACCAGCUGAAAAAGGGAAAACAGUUAUGGACAUUAUUUCGAGACAAGACCAAAGGGGGAGCGGACAAGUUGUCACUUACGCUCUUAACACAUUUACCAACCUAG
KU729218    AUGGAGAAAGGGCACAGGGCCUUGGCAUUGGCUAUAAUCAAGUACACAUACCAAAACAAAGUGGUAAAGGUCCUUAGACCAGCUGAAAAAGGGAAAACAGUUAUGGACAUUAUUUCGAGACAAGACCAAAGGGGGAGCGGACAAGUUGUCACUUACGCUCUUAACACAUUUACCAACCUAG
KU761564    AUGGAGAAAGGGCACAGGGCCUUGGCAUUGGCCAUAAUCAAGUACACAUACCAAAACAAAGUGGUAAAGGUCCUUAGACCAGCUGAAAAAGGGAAAACAGUUAUGGACAUUAUUUCGAGACAAGACCAAAGGGGGAGCGGACAAGUUGUCACUUACGCUCUUAACACAUUUACCAACCUAG
KU740184    AUGGAGAAAGGGCACAGGGCCUUGGCAUUGGCCAUAAUCAAGUACACAUACCAAAACAAAGUGGUAAAGGUCCUUAGACCAGCUGAAAAAGGGAAAACAGUUAUGGACAUUAUUUCGAGACAAGACCAAAGGGGGAGCGGACAAGUUGUCACUUACGCUCUUAACACAUUUACCAACCUAG
KU744693    AUGGAGAAAGGGCACAGGGCCUUGGCAUUGGCCAUAAUCAAGUACACAUACCAAAACAAAGUGGUAAAGGUCCUUAGACCAGCUGAAAAAGGGAAGACAGUUAUGGACAUUAUUUCGAGACAAGACCAAAGGGGGAGCGGACAAGUUGUCACUUACGCUCUCAACACAUUUACCAACCUAG
KU497555    AUGGAGAAAGGGCACAGGGCCUUGGCAUUGGCCAUAAUCAAGUACACAUACCAAAACAAAGUGGUAAAGGUCCUUAGACCAGCUGAAAAAGGGAAAACAGUUAUGGACAUUAUUUCGAGACAAGACCAAAGGGGGAGCGGACAAGUUGUCACUUACGCUCUUAACACAUUUACCAACCUAG
KU527068    AUGGAGAAAGGGCAUAGGGCCUUGGCAUUGGCCAUAAUCAAGUACACAUACCAAAACAAAGUGGUAAAGGUCCUUAGACCAGCUGAAAAAGGGAAAACAGUUAUGGACAUUAUUUCGAGACAAGACCAAAGGGGGAGCGGACAAGUUGUCACUUACGCUCUUAACACAUUUACCAACCUAG
KU501217    AUGGAGAAAGGGCACAGGGCCUUGGCAUUGGCCAUAAUCAAGUACACAUACCAAAACAAAGUGGUAAAGGUCCUUAGACCAGCUGAAAAAGGGAAAACAGUUAUGGACAUUAUUUCAAGACAAGACCAAAGGGGGAGCGGACAAGUUGUCACUUACGCUCUUAACACAUUUACCAACCUAG
KU501216    AUGGAGAAAGGGCACAGGGCCUUGGCAUUGGCCAUAAUCAAGUACACAUACCAAAACAAAGUGGUAAAGGUCCUUAGACCAGCUGAAAAAGGGAAAACAGUUAUGGACAUUAUUUCAAGACAAGACCAAAGGGGGAGCGGACAAGUUGUCACUUACGCUCUUAACACAUUUACCAACCUAG
KU501215    AUGGAGAAAGGGCACAGGGCCUUGGCAUUGGCCAUAAUCAAGUACACAUACCAAAACAAAGUGGUAAAGGUCCUUAGACCAGCUGAAAAAGGGAAAACAGUUAUGGACAUUAUUUCGAGACAAGACCAAAGGGGGAGCGGACAAGUUGUCACUUACGCUCUUAACACAUUUACCAACCUAG
KU365780    AUGGAAAAAGGGCACAGGGCCUUGGCAUUGGCCAUAAUCAAGUACACAUACCAAAACAAAGUGGUAAAGGUCCUUAGACCAGCUGAAAAAGGGAAAACAGUUAUGGACAUUAUUUCGAGACAAGACCAAAGGGGGAGCGGACAAGUUGUCACUUACGCUCUUAACACAUUUACCAACCUAG
KU365779    AUGGAAAAAGGGCACAGGGCCUUGGCAUUGGCCAUAAUCAAGUACACAUACCAAAACAAAGUGGUAAAGGUCCUUAGACCAGCUGAAAAAGGGAAAACAGUUAUGGACAUUAUUUCGAGACAAGACCAAAGGGGGAGCGGACAAGUUGUCACUUACGCUCUUAACACAUUUACCAACCUAG
KU707826    AUGGAAAAAGGGCACAGGGCCUUGGCAUUGGCCAUAAUCAAGUACACAUACCAAAACAAAGUGGUAAAGGUCCUUAGACCAGCUGAAAAAGGGAAAACAGUUAUGGACAUUAUUUCGAGACAAGACCAAAGGGGGAGCGGACAAGUUGUCACUUACGCUCUUAACACAUUUACCAACCUAG
KU365778    AUGGAGAAAGGGCACAGGGCCUUGGCAUUGGCCAUAAUCAAGUACACAUACCAAAACAAAGUGGUAAAGGUCCUUAGACCAGCUGAAAAAGGGAAAACAGUUAUGGACAUUAUUUCGAGACAAGACCAAAGGGGGAGCGGACAAGUUGUCACUUACGCUCUUAACACAUUUACCAACCUAG
KU365777    AUGGAAAAAGGGCACAGGGCCUUGGCAUUGGCCAUAAUCAAGUACACAUACCAAAACAAAGUGGUAAAGGUCCUUAGACCAGCUGAAAAAGGGAAAACAGUUAUGGACAUUAUUUCGAGACAAGACCAAAGGGGGAGCGGACAAGUUGUCACUUACGCUCUUAACACAUUUACCAACCUAG
KU312312    AUGGAGAAAGGGCACAGGGCCUUGGCAUUGGCCAUAAUCAAGUACACAUACCAAAACAAAGUGGUAAAGGUCCUUAGACCAGCUGAAAAAGGGAAAACAGUUAUGGACAUUAUUUCGAGACAAGACCAAAGGGGGAGCGGACAAGUUGUCACUUACGCUCUUAACACAUUUACCAACCUAG
KF268950    AUGGAGGAAGGGCACAGAGCUCUGGCGUUGGCUGUGAUUAAGUACACA---AAAAACAAAGUGGUGAAGGUCCUCAGACCAGCUGAAGGAGGGAAAACAGUUAUGGACAUCAUUUCAAGACAGGACCAGAGAGGGAGUGGACAAGUUGUCACCUAUGCUCUCAACACAUUUACCAACUUAG
KF268949    AUGGAGGAAGGGCAUAGAGCUCUAGCAUUGGCUGUGAUUAAGUACACAUACCAAAACAAAGUGGUGAAGGUCCUCAGACCAGCUGAAGGAGGGAAAACAGUUAUGGACAUCAUUUCAAGACAAGACCAGAGAGGGAGUGGACAAGUUGUCACUUAUGCUCUCAACACAUUUACCAACUUGG
KF268948    AUGGAGGAAGGGCACAGAGCUCUGGCGUUGGCUGUGAUUAAGUACACA---AAAAACAAAGUGGUGAAGGUCCUCAGACCAGCUGAAGGAGGGAAAACAGUUAUGGACAUCAUUUCAAGACAGGACCAGAGAGGGAGUGGACAAGUUGUCACCUAUGCUCUCAACACAUUUACCAACUUAG
KJ776791    AUGGAGAAAGGGCACAGGGCCUUGGCAUUGGCCAUAAUCAAGUACACAUACCAAAACAAAGUGGUAAAGGUCCUUAGACCAGCUGAAAAAGGGAAGACAGUUAUGGACAUUAUUUCGAGACAAGACCAAAGGGGGAGCGGACAAGUUGUCACUUACGCUCUUAACACAUUUACCAACCUAG
KF383119    AUGGAGGAAGGGCACAGAACUCUGGCGUUGGCCGUGAUUAAAUACACAUACCAAAACAAAGUGGUGAAGGUCCUCAGACCAGCUGAAGGAGGAAAAACAGUUAUGGACAUCAUUUCAAGACAAGACCAGAGAGGGAGUGGACAAGUUGUCACUUAUGCUCUCAACACAUUCACCAACUUGG
KF383118    AUGGAGGAAGGGCACAGAACUCUGGCGUUGGCCGUGAUUAAAUACACAUACCAAAACAAAGUGGUGAAGGUCCUCAGACCAGCUGAAGGAGGAAAAACAGUUAUGGACAUCAUUUCAAGACAAGACCAGAGAGGGAGUGGACAAGUUGUCACUUAUGCUCUCAACACAUUCACCAACUUGG
KF383117    AUGGAAGAAGGGCACAGAGCUCUGGCGUUGGCCGUGAUUAAAUACACAUACCAAAACAAAGUGGUGAAGGUUCUCAGACCAGCUGAAGGAGGGAAAACAGUCAUGGACAUCAUCUCAAGACAAGACCAGAGAGGGAGUGGACAAGUUGUUACUUAUGCCCUCAACACAUUCACCAACCUGG
KF383116    AUGGAAGAAGGGCACAGAGCUCUGGCGUUGGCCGUGAUUAAAUACACAUACCAAAACAAAGUGGUGAAGGUUCUCAGACCAGCUGAAGGAGGGAAAACAGUCAUGGACAUCAUCUCAAGACAAGACCAGAGAGGGAGCGGACAAGUUGUUACUUAUGCUCUCAACACAUUCACCAACCUGG
KF383115    AUGGAGGAAGGGCACAGAGCUCUGGCGUUGGCUGUGAUUAAAUACACAUACCAAAACAAAGUGGUGAAGGUCCUUAGACCAGCUGAAGGAGGAAAAACAGUUAUGGACAUCAUUUCAAGACAAGACCAGAGAGGGAGUGGGCAAGUUGUCACUUAUGCUCUCAACACGUUUACCAACUUAG
EU545988    AUGGAGAAAGGGCACAGGGCCUUGGCAUUGGCCAUAAUCAAGUACACAUACCAAAACAAAGUGGUAAAGGUCCUCAGACCAGCUGAAAAAGGGAAGACAGUUAUGGACAUUAUUUCAAGACAAGACCAAAGGGGGAGCGGACAAGUUGUCACUUACGCUCUUAAUACAUUCACCAACCUGG
KU955589    AUGGAGAAAGGGCACAGGGCCUUGGCAUUGGCCAUAAUCAAGUACACAUACCAAAACAAAGUGGUAAAGGUCCUUAGACCAGCUGAAAAAGGGAAGACAGUUAUGGACAUUAUUUCGAGACAAGACCAAAGGGGGAGCGGACAAGUUGUCACUUACGCUCUUAACACAUUUACCAACCUAG
KU955590    AUGGAGAAAGGGCACAGGGCCUUGGCAUUGGCCAUAAUCAAGUACACAUACCAAAACAAAGUGGUAAAGGUCCUUAGACCAGCUGAAAAAGGGAAAACAGUUAUGGACAUUAUUUCGAGACAAGACCAAAGGGGGAGCGGACAAGUUGUCACUUACGCUCUUAACACAUUUACCAACCUAG
KU940228    AUGGAGAAAGGGCACAGGGCCUUGGCAUUGGCCAUAAUCAAGUACACAUACCAAAACAAAGUGGUAAAGGUCCUUAGACCAGCUGAAAAAGGGAAAACAGUUAUGGACAUUAUUUCGAGACAAGACCAAAGGGGGAGCGGACAAGUUGUCACUUACGCUCUUAACACAUUUACCAACCUAG
KU991811    AUGGAGAAAGGGCACAGGGCCUUGGCAUUGGCCAUAAUCAAGUACACAUACCAAAACAAAGUGGUAAAGGUCCUUAGACCAGCUGAAAAAGGGAAAACAGUUAUGGACAUUAUUUCGAGACAAGACCAAAGGGGGAGCGGACAAGUUGUCACUUACGCUCUUAACACAUUUACCAACCUAG
KU509998    AUGGAGAAAGGGCACAGGGCCUUGGCAUUGGCCAUAAUCAAGUACACAUACCAAAACAAAGUGGUAAAGGUCCUUAGACCAGCUGAAAAAGGGAAGACAGUUAUGGACAUUAUUUCGAGACAAGACCAAAGGGGGAGCGGACAAGUUGUCACUUACGCUCUUAACACAUUUACCAACCUAG
KX056898    AUGGAGAAAGGGCACAGGGCCUUGGCAUUGGCCAUAAUCAAGUACACAUACCAAAACAAAGUGGUAAAGGUCCUUAGACCAGCUGAAAAAGGGAAAACAGUUAUGGACAUUAUUUCGAGACAAGACCAAAGGGGGAGCGGACAAGUUGUCACUUACGCUCUUAACACAUUUACCAACCUAG
KX117076    AUGGAGAAAGGGCACAGGGCCUUGGCAUUGGCCAUAAUCAAGUACACAUACCAAAACAAAGUGGUAAAGGUCCUUAGACCAGCUGAAAAAGGGAAGACAGUUAUGGACAUUAUUUCGAGACAAGACCAAAGGGGGAGCGGACAAGUUGUCACUUACGCUCUUAACACAUUUACCAACCUAG
KX185891    AUGGAGAAAGGGCACAGGGCCUUGGCAUUGGCCAUAAUCAAGUACACAUACCAAAACAAAGUGGUAAAGGUCCUUAGACCAGCUGAAAAAGGGAAGACAGUUAUGGACAUUAUUUCGAGACAAGACCAAAGGGGGAGCGGACAAGUUGUCACUUACGCUCUUAACACAUUUACCAACCUAG
KX247646    AUGGAGAAAGGGCACAGGGCCUUGGCAUUGGCCAUAAUCAAGUACACAUACCAAAACAAAGUGGUAAAGGUCCUUAGACCAGCUGAAAAAGGGAAAACAGUUAUGGACAUUAUUUCGAGACAAGACCAAAGGGGGAGCGGACAAGUUGUCACUUACGCUCUUAACACAUUUACCAACCUAG
KX262887    AUGGAGAAAGGGCACAGGGCCUUGGCAUUGGCCAUAAUCAAGUACACAUACCAAAACAAAGUGGUAAAGGUCCUUAGACCAGCUGAAAAAGGGAAAACAGUUAUGGACAUUAUUUCGAGACAAGACCAAAGGGGGAGCGGACAAGUUGUCACUUACGCUCUUAACACAUUUACCAACCUAG
KX280026    AUGGAGAAAGGGCACAGGGCCUUGGCAUUGGCCAUAAUCAAGUACACAUACCAAAACAAAGUGGUAAAGGUCCUUAGACCAGCUGAAAAAGGGAAAACAGUUAUGGACAUUAUUUCGAGACAAGACCAAAGGGGGAGCGGACAAGUUGUCACUUACGCUCUUAACACAUUUACCAACCUAG
KU758877    AUGGAGAAAGGGCACAGGGCCUUGGCAUUGGCCAUAAUCAAGUACACAUACCAAAACAAAGUGGUAAAGGUCCUUAGACCAGCUGAAAAAGGGAAAACAGUUAUGGACAUUAUUUCGAGACAAGACCAAAGGGGGAGCGGACAAGUUGUCACUUACGCUCUUAACACAUUUACCAACCUAG
KU866423    AUGGAGAAAGGGCACAGGGCCUUGGCAUUGGCCAUAAUCAAGUACACAUACCAAAACAAAGUGGUAAAGGUCCUUAGACCAGCUGAAAAAGGGAAGACAGUUAUGGACAUUAUUUCGAGACAAGACCAAAGGGGGAGCGGACAAGUUGUCACUUACGCUCUUAACACAUUUACCAACCUAG
KX377336    AUGGAGAAAGGGCACAGGGCCUUGGCGUUGGCCAUAAUCAAGUACACAUACCAAAACAAAGUGGUAAAGGUCCUUAGACCAGCUGAAAGAGGGAAGACAGUUAUGGACAUCAUCUCAAGACAAGACCAAAGAGGGAGCGGACAAGUUGUUACUUACGCUCUUAAUACAUUCACCAACCUGG
KX377337    AUGGAGAAAGGGCACAGGGCCUUGGCAUUGGCCAUAAUCAAGUACACAUACCAAAACAAAGUGGUAAAGGUCCUUAGACCAGCUGAAAAAGGGAAAACAGUUAUGGACAUUAUUUCGAGACAAGACCAAAGGGGGAGCGGACAAGUUGUCACUUACGCUCUUAACACAUUUACCAACCUAG
KX369547    AUGGAGAAAGGGCACAGGGCCUUGGCAUUGGCCAUAAUCAAGUACACAUACCAAAACAAAGUGGUAAAGGUCCUUAGACCAGCUGAAAAAGGGAAGACAGUUAUGGACAUUAUUUCGAGACAAGACCAAAGGGGGAGCGGACAAGUUGUCACUUACGCUCUUAACACAUUUACCAACCUAG
KX548902    AUGGAGAAAGGGCACAGGGCCUUGGCAUUGGCCAUAAUCAAGUACACAUACCAAAACAAAGUGGUAAAGGUCCUUAGACCAGCUGAAAAAGGGAAAACAGUUAUGGACAUUAUUUCGAGACAAGACCAAAGGGGGAGCGGACAAGUUGUCACUUACGCUCUUAACACAUUUACCAACCUAG
KX601166    AUGGAAGAAGGGCACAGAGCUCUGGCGUUGGCCGUGAUUAAAUACACAUACCAAAACAAAGUGGUGAAGGUUCUCAGACCAGCUGAAGGAGGGAAAACAGUCAUGGACAUCAUCUCAAGACAAGACCAGAGAGGGAGCGGACAAGUUGUUACUUAUGCUCUCAACACAUUCACCAACCUGG
```

```
KX601167       AUGGAGAAAGGGCACAGGGCCUUGGCGUUGGCCAUAAUCAAGUACACAUACCAAAACAAAGUGGUAAAGGUCCUUAGACCAGCUGAAAGAGGGAAGACAGUUAUGGACAUCAUCUCAAGACAAGACCAAAGAGGGAGCGGACAAGUUGUUACUUACGCUCUUAAUACAUUCACCAACCUGG
KX601168       AUGGAGAAAGGGCACAGGGCCUUGGCAUUGGCCAUAAUCAAGUACACAUACCAAAACAAAGUGGUAAAGGUCCUUAGACCAGCUGAAAAAGGGAAAACAGUUAUGGACAUUAUUUCGAGACAAGACCAAAGGGGGAGCGGACAAGUUGUCACUUACGCUCUUAACACAUUUACCAACCUAG
KX766028       AUGGAGAAAGGGCACAGGGCCUUGGCAUUGGCCAUAAUCAAGUACACAUACCAAAACAAAGUGGUAAAGGUCCUUAGACCAGCUGAAAAAGGGAAAACAGUUAUGGACAUUAUUUCGAGACAAGACCAAAGGGGGAGCGGACAAGUUGUCACUUACGCUCUUAACACAUUUACCAACCUAG
KX766029       AUGGAGAAAGGGCACAGGGCCUUGGCAUUGGCCAUAAUCAAGUACACAUACCAAAACAAAGUGGUAAAGGUCCUUAGACCAGCUGAAAAAGGGAAAACAGUUAUGGACAUUAUUUCGAGACAAGACCAAAGGGGGAGCGGACAAGUUGUCACUUACGCUCUUAACACAUUUACCAACCUAG
KU940224       AUGGAGAAAGGGCACAGGGCCUUGGCAUUGGCCAUAAUCAAGUACACAUACCAAAACAAAGUGGUAAAGGUCCUUAGACCAGCUGAAAAAGGGAAAACAGUUAUGGACAUUAUUUCGAGACAAGACCAAAGGGGGAGCGGACAAGUUGUCACUUACGCUCUUAACACAUUUACCAACCUAG


               9999999999999999999999999999999999999999999999999999999999999999999999999999999999999999999999999999999999999999999999999999999999999999999999999999999999999999999999999999999
               4444444444444444444444444444444444444444444444444444444444444444444444444444444444444445555555555555555555555555555555555555555555555555555555555555555555555555555555555555555555
               1111111222222222233333333334444444444555555555566666666667777777777888888888899999999990000000000111111111122222222223333333333444444444455555555556666666666777777777788888888889999
Sequence       3456789012345678901234567890123456789012345678901234567890123456789012345678901234567890123456789012345678901234567890123456789012345678901234567890123456789012345678901234567890123
---------------------------------------------------------------------------------------------------------------------------------------------------------------------------------------------------------------
NC_012532      UGGUGCAGCUUAUCCGGAACAUGGAAGCUGAGGAAGUGUUAGAGAUGCAAGACUUAUGGUUGUUGAGGAAGCCAGAGAAAGUGACCAGAUGGUUGCAGAGCAAUGGAUGGGAUAGACUCAAACGAAUGGCGGUCAGUGGAGAUGACUGCGUUGUGAAGCCAAUCGAUGAUAGGUUUGCACA
KU922960       UGGUGCAACUCAUUCGGAAUAUGGAGGCUGAGGAAGUUCUAGAGAUGCAAGACUUGUGGCUGCUGCGGAGGUCAGAGAAAGUGACCAACUGGUUGCAGAGCAACGGAUGGGAUAGGCUCAAACGAAUGGCAGUCAGUGGAGAUGAUUGCGUUGUGAAGCCAAUUGAUGAUAGGUUUGCACA
KU922923       UGGUGCAACUCAUUCGGAAUAUGGAGGCUGAGGAAGUUCUAGAGAUGCAAGACUUGUGGCUGCUGCGGAGGUCAGAGAAAGUGACCAACUGGUUGCAGAGCAACGGAUGGGAUAGGCUCAAACGAAUGGCAGUCAGUGGAGAUGAUUGCGUUGUGAAGCCAAUUGAUGAUAGGUUUGCACA
KU820898       UGGUGCAACUCAUUCGGAAUAUGGAGGCUGAGGAAGUUCUAGAGAUGCAAGACUUGUGGCUGCUGCGGAGGUCAGAGAAAGUGACCAACUGGUUGCAGAGCAACGGAUGGGAUAGGCUCAAACGAAUGGCAGUCAGUGGAGAUGAUUGCGUUGUGAAGCCAAUUGAUGAUAGGUUUGCACA
KU321639       UGGUGCAACUCAUUCGGAAUAUGGAGGCUGAGGAAGUCCUAGAGAUGCAAGACUUGUGGCUGCUGCGGAGGUCAGAGAAAGUGACCAACUGGUUGCAGAGCAACGGAUGGGAUAGGCUCAAACGAAUGGCAGUCAGUGGAGAUGAUUGCGUUGUGAAGCCAAUUGAUGAUAGGUUUGCACA
KU820899       UGGUGCAACUCAUUCGGAGUAUGGAGGCUGAGGAAGUUCUAGAGAUGCAAGACUUGUGGCUGCUGCGGAGGUCAGAGAAAGUGACCAACUGGCUGCAGAGCAACGGAUGGGAUAGGCUCAAACGAAUGGCAGUCAGUGGAGAUGAUUGCGUUGUGAGGCCAAUUGAUGAUAGGUUUGCACA
KU853012       UGGUGCAACUCAUUCGGAAUAUGGAGGCUGAGGAAGUUCUAGAGAUGCAAGACUUGUGGCUGCUGCGGAGGUCAGAGAAAGUGACCAACUGGUUGCAGAGCAACGGAUGGGAUAGGCUCAAACGAAUGGCAGUCAGUGGAGAUGAUUGCGUUGUGAAGCCAAUUGAUGAUAGGUUUGCACA
KU729217       UGGUGCAACUCAUUCGGAAUAUGGAGGCUGAGGAAGUUCUAGAGAUGCAAGACUUGUGGCUGCUGCGGAGGUCAGAGAAAGUGACCAACUGGUUGCAGAGCAACGGAUGGGAUAGGCUCAAACGAAUGGCAGUCAGUGGAGAUGAUUGCGUUGUGAAGCCAAUUGAUGAUAGGUUUGCACA
KU729218       UGGUGCAACUCAUUCGGAAUAUGGAGGCUGAGGAAGUUCUAGAGAUGCAAGACUUGUGGCUGCUGCGGAGGUCAGAGAAAGUGACCAACUGGUUGCAGAGCAACGGAUGGGAUAGGCUCAAACGAAUGGCAGUCAGUGGGGAUGAUUGCGUUGUGAAGCCAAUUGAUGAUAGGUUUGCACA
KU761564       UGGUGCAACUCAUUCGGAAUAUGGAGGCUGAGGAAGUUCUAGAGAUGCAAGACUUGUGGCUGCUGCGGAGGUCAGAGAAAGUGACCAACUGGUUGCAGAGCAACGGAUGGGAUAGGCUCAAACGAAUGGCAGUCAGUGGAGAUGAUUGCGUUGUGAAGCCAAUUGAUGAUAGGUUUGCACA
KU740184       UGGUGCAACUCAUUCGGAAUAUGGAGGCUGAGGAAGUUCUAGAGAUGCAAGACUUGUGGCUGCUGCGGAGGUCAGAGAAAGUGACCAACUGGUUGCAGAGCAACGGAUGGGAUAGGCUCAAACGAAUGGCAGUCAGUGGAGAUGAUUGCGUUGUGAAGCCAAUUGAUGAUAGGUUUGCACA
KU744693       UGGUGCAACUCAUUCGGAAUAUGGAGGCUGAGGAAGUUCUAGAGAUGCAAGACUUGUGGCUGCUGCGGAGGUCAGAGAAAGUGACCAACUGGUUGCAGAGCAACGGAUGGGAUAGGCUCAAACGAAUGGCGGUCAGUGGAGAUGAUUGCGUUGUGAAACCAAUUGAUGAUAGGUUUGCACA
KU497555       UGGUGCAACUCAUUCGGAAUAUGGAGGCUGAGGAAGUUCUAGAGAUGCAAGACUUGUGGCUGCUGCGGAGGUCAGAGAAAGUGACCAACUGGUUGCAGAGCAACGGAUGGGAUAGGCUCAAACGAAUGGCAGUCAGUGGAGAUGAUUGCGUUGUGAAGCCAAUUGAUGAUAGGUUUGCACA
KU527068       UGGUGCAACUCAUUCGGAAUAUGGAGGCUGAGGAAGUUCUAGAGAUGCAAGACUUGUGGCUGCUGCGGAGGUCAGAGAAAGUGACCAACUGGUUGCAGAGCAACGGAUGGGAUAGGCUCAAACGAAUGGCAGUCAGUGGAGAUGAUUGCGUUGUGAAGCCAAUUGAUGAUAGGUUUGCACA
KU501217       UGGUGCAACUCAUUCGGAAUAUGGAGGCUGAGGAAGUUCUAGAGAUGCAAGACUUGUGGCUGCUGCGGAGGUCAGAGAAAGUGACCAACUGGUUGCAGAGCAACGGAUGGGAUAGGCUCAAACGAAUGGCAGUCAGUGGAGAUGAUUGCGUUGUGAAGCCAAUUGAUGAUAGGUUUGCACA
KU501216       UGGUGCAACUCAUUCGGAAUAUGGAGGCUGAGGAAGUUCUAGAGAUGCAAGACUUGUGGCUGCUGCGGAGGUCAGAGAAAGUGACCAACUGGUUGCAGAGCAACGGAUGGGAUAGGCUCAAACGAAUGGCAGUCAGUGGAGAUGAUUGCGUUGUGAAGCCAAUUGAUGAUAGGUUUGCACA
KU501215       UGGUGCAACUCAUUCGGAAUAUGGAGGCUGAGGAAGUUCUAGAGAUGCAAGACUUGUGGCUGCUGCGGAGGUCAGAGAAAGUGACCAACUGGUUGCAGAGCAACGGAUGGGAUAGGCUCAAACGAAUGGCAGUCAGUGGAGAUGAUUGCGUUGUGAAGCCAAUUGAUGAUAGGUUUGCACA
KU365780       UGGUGCAACUCAUUCGGAAUAUGGAGGCUGAGGAAGUUCUAGAGAUGCAAGACUUGUGGCUGCUGCGGAGGUCAGAGAAAGUGACCAACUGGUUGCAGAGCAACGGAUGGGAUAGGCUCAAACGAAUGGCAGUCAGUGGAGAUGAUUGCGUUGUGAAGCCAAUUGAUGAUAGGUUUGCACA
KU365779       UGGUGCAACUCAUUCGGAAUAUGGAGGCUGAGGAAGUUCUAGAGAUGCAAGACUUGUGGCUGCUGCGGAGGUCAGAGAAAGUGACCAACUGGUUGCAGAGCAACGGAUGGGAUAGGCUCAAACGAAUGGCAGUCAGUGGAGAUGAUUGCGUUGUGAAGCCAAUUGAUGAUAGGUUUGCACA
KU707826       UGGUGCAACUCAUUCGGAAUAUGGAGGCUGAGGAAGUUCUAGAGAUGCAAGACUUGUGGCUGCUGCGGAGGUCAGAGAAAGUGACCAACUGGUUGCAGAGCAACGGAUGGGAUAGGCUCAAACGAAUGGCAGUCAGUGGAGAUGAUUGCGUUGUGAAGCCAAUUGAUGAUAGGUUUGCACA
KU365778       UGGUGCAACUCAUUCGGAAUAUGGAGGCUGAGGAAGUUCUAGAGAUGCAAGACUUGUGGCUGCUGCGGAGGUCAGAGAAAGUGACCAACUGGUUGCAGAGCAACGGAUGGGAUAGGCUCAAACGAAUGGCAGUCAGUGGAGAUGAUUGCGUUGUGAAGCCAAUUGAUGAUAGGUUUGCACA
KU365777       UGGUGCAACUCAUUCGGAAUAUGGAGGCUGAGGAAGUUCUAGAGAUGCAAGACUUGUGGCUGCUGCGGAGGUCAGAGAAAGUGACCAACUGGUUGCAGAGCAACGGAUGGGAUAGGCUCAAACGAAUGGCAGUCAGUGGAGAUGAUUGCGUUGUGAAGCCAAUUGAUGAUAGGUUUGCACA
KU312312       UGGUGCAACUCAUUCGGAAUAUGGAGGCUGAGGAAGUUCUAGAGAUGCAAGACUUGUGGCUGCUGCGGAGGUCAGAGAAAGUGACUAACUGGUUGCAGAGCAACGGAUGGGAUAGGCUCAAACGAAUGGCAGUCAGUGGAGAUGAUUGCGUUGUGAAGCCAAUUGAUGAUAGGUUUGCACA
KF268950       UGGUGCAGCUUAUCCGGAAUAUGGAGGCUGAGGAAGUGUUAGAGAUGCAAGACUUAUGGCUGUUGAGGAAGCCAGAGAAAGUGACCAGAUGGUUGCAGUGCAAUGGAUGGGACAGACUCAAACGAAUGGCAGUCAGUGGAGAUGACUGCGUUGUGAAGCCAAUUGAUGAUAGGUUUGCGCA
KF268949       UGGUGCAGCUUAUCCGGAACAUGGAGGCUGAGGAAGUGCUAGAGAUGCAAGACUUAUGGCUGCUGAGGAAGCCAGAGAAAGUGACCAGAUGGUUGCAGUGCAAUGGAUGGGACAGACUCAAACGAAUGGCAGUCAGUGGAGAUGACUGCGUUGUGAAGCCAAUUGAUGAUAGGUUUGCACA
KF268948       UGGUGCAGCUUAUCCGGAAUAUGGAGGCUGAGGAAGUGUUAGAGAUGCAAGACUUAUGGCUGUUGAGGAAGCCAGAGAAAGUGACCAGAUGGUUGCAGUGCAAUGGAUGGGACAGACUCAAACGAAUGGCAGUCAGUGGAGAUGACUGCGUUGUGAAGCCAAUUGAUGAUAGGUUUGCGCA
KJ776791       UGGUGCAACUCAUUCGGAAUAUGGAGGCUGAGGAAGUUCUAGAGAUGCAAGACUUGUGGCUGCUGCGGAGGUCAGAGAAAGUGACCAACUGGUUGCAGAGCAACGGAUGGGAUAGGCUCAAACGAAUGGCAGUCAGUGGAGAUGAUUGCGUUGUGAAGCCAAUUGAUGAUAGGUUUGCACA
KF383119       UGGUGCAGCUUAUCCGGAACAUGGAAGCUGAGGAAGUGUUAGAGAUGCAAGACUUAUGGCUGUUGAGGAAGCCAGAGAAAGUGACCAGAUGGUUGCAGAGCAAUGGAUGGGAUGGACUCAAACGAAUGGCGGUCAGUGGAGAUGACUGCGUUGUGAAGCCAAUCGAUGAUAGGUUUGCACA
KF383118       UGGUGCAGCUUAUCCGGAACAUGGAAGCUGAGGAAGUGUUAGAGAUGCAAGACUUAUGGCUGUUGAGGAAGCCAGAGAAAGUGACCAGAUGGUUGCAGAGCAAUGGAUGGGAUAGACUCAAACGAAUGGCGGUCAGUGGAGAUGACUGCGUUGUGAAGCCAAUCGAUGAUAGGUUUGCACA
KF383117       UGGUGCAGCUUAUCCGGAACAUGGAGGCUGAGGAAGUGCUAGAGAUGCAUGAUCUGUGGUUGUUGAGGAAACCAGAGAAAGUGACCAGAUGGUUGCAGAGUAAUGGAUGGGACAGACUCAAACGGAUGGCAGUUAGUGGAGAUGACUGCGUUGUAAAGCCAAUUGAUGAUAGGUUUGCACA
KF383116       UGGUGCAGCUUAUCCGGAACAUGGAGGCUGAGGAAGUGCUAGAGAUGCAUGAUCUAUGGUUGUUGAGGAAGCCAGAGAAAGUGACCAGAUGGUUGCAGAGCAAUGGAUGGGACAGACUCAAACGGAUGGCAGUUAGUGGAGAUGACUGCGUUGUAAAGCCAAUUGAUGAUAGGUUUGCACA
KF383115       UGGUGCAGCUUAUUCGGAACAUGGAGGCUGAGGAAGUGUUAGAGAUGCAAGACUUAUGGCUGUUGAGGAAGCCAGAGAAAGUGACUAGAUGGUUGCAGAGUAAUGGAUGGGACAGACUCAAACGAAUGGCAGUCAGUGGAGAUGACUGUGUUGUGAAGCCAAUUGAUGAUAGGUUUGCAUG
EU545988       UGGUGCAGCUCAUUCGGAAUAUGGAGGCUGAGGAAGUUCUAGAGAUGCAAGACUUGUGGCUGCUGCGGAGGUCAGAGAAAGUGACCAACUGGUUGCAGAGCAACGGAUGGGAUAGGCUCAAACGAAUGGCAGUCAGUGGAGAUGAUUGCGUUGUGAAACCAAUUGAUGAUAGGUUUGCACA
KU955589       UGGUGCAACUCAUUCGGAGUAUGGAGGCUGAGGAAGUUCUAGAGAUGCAAGACUUGUGGCUGCUGCGGAGGUCAGAGAAAGUGACCAACUGGCUGCAGAGCAACGGAUGGGAUAGGCUCAAACGAAUGGCAGUCAGUGGAGAUGAUUGCGUUGUGAGGCCAAUUGAUGAUAGGUUUGCACA
KU955590       UGGUGCAACUCAUUCGGAAUAUGGAGGCUGAGGAAGUUCUAGAGAUGCAAGACUUGUGGCUGCUGCGGAGGUCAGAGAAAGUGACCAACUGGUUGCAGAGCAACGGAUGGGAUAGGCUCAAACGAAUGGCAGUCAGUGGAGAUGAUUGCGUUGUGAAGCCAAUUGAUGAUAGGUUUGCACA
KU940228       UGGUGCAACUCAUUCGGAAUAUGGAGGCUGAGGAAGUUCUCGAGAUGCAAGACUUGUGGCUGCUGCGGAGGCCAGAGAAAGUGACCAACUGGUUGCAGAGCAACGGAUGGGAUAGGCUCAAACGAAUGGCAGUCAGUGGAGAUGAUUGCGUUGUGAAGCCAAUUGAUGAUAGGUUUGCACA
KU991811       UGGUGCAACUCAUUCGGAAUAUGGAGGCUGAGGAAGUUCUAGAGAUGCAAGACUUGUGGCUGCUGCGGAGGUCAGAGAAAGUGACCAACUGGUUGCAGAGCAACGGAUGGGAUAGGCUCAAACGAAUGGCAGUCAGUGGAGAUGAUUGCGUUGUGAAGCCAAUUGAUGAUAGGUUUGCACA
KU509998       UGGUGCAACUCAUUCGGAAUAUGGAGGCUGAGGAAGUUCUAGAGAUGCAAGACUUGUGGCUGCUGCGGAGGUCAGAGAAAGUGACCAACUGGUUGCAGAGCAACGGAUGGGAUAGGCUCAAACGAAUGGCAGUCAGUGGAGAUGAUUGCGUUGUGAAGCCAAUUGAUGAUAGGUUUGCACA
KX056898       UGGUGCAACUCAUUCGGAAUAUGGAGGCUGAGGAAGUUCUAGAGAUGCAAGACUUGUGGCUGCUGCGGAGGUCAGAGAAAGUGACCAACUGGUUGCAGAGCAACGGAUGGGAUAGGCUCAAACGAAUGGCAGUCAGUGGAGAUGAUUGCGUUGUGAAGCCAAUUGAUGAUAGGUUUGCACA
KX117076       UGGUGCAACUCAUUCGGAGUAUGGAGGCUGAGGAAGUUCUAGAGAUGCAAGACUUGUGGCUGCUGCGGAGGUCAGAGAAAGUGACCAACUGGCUGCAGAGCAACGGAUGGGAUAGGCUCAAACGAAUGGCAGUCAGUGGAGAUGAUUGCGUUGUGAGGCCAAUUGAUGAUAGGUUUGCACA
KX185891       UGGUGCAACUCAUUCGGAGUAUGGAGGCUGAGGAAGUUCUAGAGAUGCAAGACUUGUGGCUGCUGCGGAGGUCAGAGAAAGUGACCAACUGGCUGCAGAGCAACGGAUGGGAUAGGCUCAAACGAAUGGCAGUCAGUGGAGAUGAUUGCGUUGUGAGGCCAAUUGAUGAUAGGUUUGCACA
KX247646       UGGUGCAACUCAUUCGGAAUAUGGAGGCUGAGGAAGUUCUAGAGAUGCAAGACUUGUGGCUGCUGCGGAGGUCAGAGAAAGUGACCAACUGGUUGCAGAGCAACGGAUGGGAUAGGCUCAAACGAAUGGCAGUCAGUGGAGAUGAUUGCGUUGUGAAGCCAAUUGAUGAUAGGUUUGCACA
KX262887       UGGUGCAACUCAUUCGGAAUAUGGAGGCUGAGGAAGUUCUAGAGAUGCAAGACUUGUGGCUGCUGCGGAGGUCAGAGAAAGUGACCAACUGGUUGCAGAGCAACGGAUGGGAUAGGCUCAAACGAAUGGCAGUCAGUGGAGAUGAUUGCGUUGUGAAGCCAAUUGAUGAUAGGUUUGCACA
KX280026       UGGUGCAACUCAUUCGGAAUAUGGAGGCUGAGGAAGUUCUGGAGAUGCAAGACUUGUGGCUGCUGCGGAGGUCAGAGAAAGUGACCAACUGGUUGCAGAGCAACGGAUGGGAUAGGCUCAAACGAAUGGCAGUCAGUGGAGAUGAUUGCGUUGUGAAGCCAAUUGAUGAUAGGUUUGCACA
KU758877       UGGUGCAACUCAUUCGGAAUAUGGAGGCUGAGGAAGUUCUAGAGAUGCAAGACUUGUGGCUGCUGCGGAGGUCAGAGAAAGUGACCAACUGGUUGCAGAGCAACGGAUGGGAUAGGCUCAAACGAAUGGCAGUCAGUGGAGAUGAUUGCGUUGUGAAGCCAAUUGAUGAUAGGUUUGCACA
KU866423       UGGUGCAACUCAUUCGGAGUAUGGAGGCUGAGGAAGUUCUAGAGAUGCAAGACUUGUGGCUGCUGCGGAGGUCAGAGAAAGUGACCAACUGGCUGCAGAGCAACGGAUGGGAUAGGCUCAAACGAAUGGCAGUCAGUGGAGAUGAUUGCGUUGUGAGGCCAAUUGAUGAUAGGUUUGCACA
KX377336       UGGUGCAGCUCAUUCGGAACAUGGAGGCUGAGGAAGUUCUAGAGAUGCAAGACUUGUGGCUGUUGAGGAGGCCAGAGAAGGUGACCAGCUGGUUGCAGAGCAACGGAUGGGAUAGGCUCAAACGAAUGGCAGUCAGUGGAGAUGAUUGUGUUGUGAAACCAAUUGAUGAUAGGUUUGCACA
KX377337       UGGUGCAACUCAUUCGGAAUAUGGAGGCUGAGGAAGUUCUAGAGAUGCAAGACUUGUGGCUGCUGCGGAGGUCAGAGAAAGUGACCAACUGGUUGCAGAGCAACGGAUGGGAUAGGCUCAAACGAAUGGCAGUCAGUGGAGAUGAUUGCGUUGUGAAGCCAAUUGAUGAUAGGUUUGCACA
KX369547       UGGUGCAACUCAUUCGGAAUAUGGAGGCUGAGGAAGUUCUAGAGAUGCAAGACUUGUGGCUGCUGCGGAGGUCAGAGAAAGUGACCAACUGGUUGCAGAGCAACGGAUGGGAUAGGCUCAAACGAAUGGCAGUCAGUGGAGAUGAUUGCGUUGUGAAGCCAAUUGAUGAUAGGUUUGCACA
KX548902       UGGUGCAACUCAUUCGGAAUAUGGAGGCUGAGGAAGUUCUAGAGAUGCAAGACUUGUGGCUGCUGCGGAGGUCAGAGAAAGUGACCAACUGGUUGCAGAGCAACGGAUGGGAUAGGCUCAAACGAAUGGCAGUCAGUGGAGAUGAUUGCGUUGUGAAGCCAAUUGAUGAUAGGUUUGCACA
KX601166       UGGUGCAGCUUAUCCGGAACAUGGAGGCUGAGGAAGUGCUAGAGAUGCAUGAUCUGUGGUUGUUGAGGAAGCCAGAGAAAGUGACCAGAUGGUUGCAGAGCAAUGGAUGGGACAGACUCAAACGGAUGGCAGUUAGUGGAGAUGACUGCGUUGUAAAGCCAAUUGAUGAUAGGUUUGCACA
KX601167       UGGUGCAGCUCAUUCGGAACAUGGAGGCUGAGGAAGUUCUAGAGAUGCAAGACUUGUGGCUGUUGAGGAGGCCAGAGAAGGUGACCAGCUGGUUGCAGAGCAACGGAUGGGAUAGGCUCAAACGAAUGGCAGUCAGUGGAGAUGAUUGUGUUGUGAAACCAAUUGAUGAUAGGUUUGCACA
KX601168       UGGUGCAACUCAUUCGGAAUAUGGAGGCUGAGGAAGUUCUAGAGAUGCAAGACUUGUGGCUGCUGCGGAGGUCAGAGAAAGUGACCAACUGGUUGCAGAGCAACGGAUGGGAUAGGCUCAAACGAAUGGCAGUCAGUGGAGAUGAUUGCGUUGUGAAGCCAAUUGAUGAUAGGUUUGCACA
KX766028       UGGUGCAACUCAUUCGGAAUAUGGAGGCUGAGGAAGUUCUAGAGAUGCAAGACUUGUGGCUGCUGCGGAGGUCAGAGAAAGUGACCAACUGGUUGCAGAGCAACGGAUGGGAUAGGCUCAAACGAAUGGCAGUCAGUGGAGAUGAUUGCGUUGUGAAGCCAAUUGAUGAUAGGUUUGCACA
KX766029       UGGUGCAACUCAUUCGGAAUAUGGAGGCUGAGGAAGUUCUAGAGAUGCAAGACUUGUGGCUGCUGCGGAGGUCAGAGAAAGUGACCAACUGGUUGCAGAGCAACGGAUGGGAUAGGCUCAAACGAAUGGCAGUCAGUGGAGAUGAUUGCGUUGUGAAGCCAAUUGAUGAUAGGUUUGCACA
KU940224       UGGUGCAACUCAUUCGGAAUAUGGAGGCUGAGGAAGUUCUCGAGAUGCAAGACUUGUGGCUGCUGCGGAGGCCAGAGAAAGUGACCAACUGGUUGCAGAGCAACGGAUGGGAUAGGCUCAAACGAAUGGCAGUCAGUGGAGAUGAUUGCGUUGUGAAGCCAAUUGAUGAUAGGUUUGCACA


               9999999999999999999999999999999999999999999999999999999999999999999999999999999999999999999999999999999999999999999999999999999999999999999999999999999999999999999999999999999
               5555556666666666666666666666666666666666666666666666666666666666666666666666666666666666666666666666666777777777777777777777777777777777777777777777777777777777777777777777777777
               9999990000000000111111111122222222223333333333444444444455555555556666666666777777777788888888889999999999000000000011111111112222222222333333333344444444445555555555666666666677777
Sequence       4567890123456789012345678901234567890123456789012345678901234567890123456789012345678901234567890123456789012345678901234567890123456789012345678901234567890123456789012345678901234
---------------------------------------------------------------------------------------------------------------------------------------------------------------------------------------------------------------
NC_012532      UGCCCUCAGGUUCUUGAAUGACAUGGGAAAAGUUAGGAAAGACACACAGGAGUGGAAACCCUCGACUGGAUGGAGCAAUUGGGAAGAAGUCCCGUUCUGCUCCCACCACUUCAACAAGCUGUACCUCAAGGAUGGGAGAUCCAUUGUGGUCCCUUGCCGCCACCAAGAUGAACUGAUUGGC
KU922960       UGCCCUCAGGUUCUUGAAUGAUAUGGGAAAAGUUAGGAAGGACACACAAGAGUGGAAACCCUCAACUGGAUGGGACAACUGGGAAGAAGUUCCGUUUUGCUCCCACCACUUCAACAAGCUCCAUCUCAAGGACGGGAGGUCCAUUGUGGUUCCCUGCCGCCACCAAGAUGAACUGAUUGGC
```

```
KU922923    UGCCCUCAGGUUCUUGAAUGAUAUGGGAAAAGUUAGGAAGGACACACAAGAGUGGAAACCCUCAACUGGAUGGGACAACUGGGAAGAAGUUCCGUUUUGCUCCCACCACUUCAACAAGCUCCAUCUCAAGGACGGGAGGUCCAUUGUGGUUCCCUGCCGCCACCAAGAUGAACUGAUUGGC
KU820898    UGCCCUCAGGUUCUUGAAUGAUAUGGGAAAAGUUAGGAAGGACACACAAGAGUGGAAACCCUCAACUGGAUGGGACAACUGGGAAGAAGUUCCGUUUUGCUCCCACCAUUUCAACAAGCUCCAUCUCAAGGACGGGAGGUCCAUUGUGGUUCCCUGCCGCCACCAAGAUGAACUGAUUGGC
KU321639    UGCCCUCAGGUUCUUGAAUGAUAUGGGAAAAGUUAGGAAGGACACACAAGAGUGGAAACCCUCAACUGGAUGGGACAACUGGGAAGAAGUUCCGUUUUGCUCCCACCACUUCAACAAGCUCCAUCUCAAGGACGGGAGGUCCAUUGUGGUUCCCUGCCGCCACCAAGAUGAACUGAUUGGC
KU820899    UGCCCUCAGGUUCUUGAAUGAUAUGGGGAAAGUUAGGAAGGACACACAAGAGUGGAAACCCUCAACUGGAUGGGACAACUGGGAGGAAGUUCCGUUUUGCUCCCACCACUUCAACAAGCUCCAUCUCAAGGACGGGAGGUCCAUUGUGGUUCCCUGCCGCCACCAAGAUGAACUGAUUGGC
KU853012    UGCCCUCAGGUUCUUGAAUGAUAUGGGAAAAGUUAGGAAGGACACACAAGAGUGGAAACCCUCAACUGGAUGGGACAACUGGGAAGAAGUUCCGUUUUGCUCCCACCACUUCAACAAGCUCCAUCUCAAGGACGGGAGGUCCAUUGUGGUUCCCUGCCGCCACCAAGAUGAACUGAUUGGC
KU729217    UGCCCUCAGGUUCUUGAAUGAUAUGGGAAAAGUUAGGAAGGACACACAAGAGUGGAAACCCUCAACUGGAUGGGACAACUGGGAAGAAGUUCCGUUUUGCUCCCACCACUUCAACAAGCUCCAUCUCAAGGACGGGAGGUCCAUUGUGGUUCCCUGCCGCCACCAAGAUGAACUGAUUGGC
KU729218    UGCCCUUAGGUUCUUGAAUGAUAUGGGAAAAGUUAGGAAGGACACACAAGAGUGGAAACCCUCAACUGGAUGGGACAACUGGGAAGAAGUUCCGUUUUGCUCCCACCACUUCAACAAGCUCCAUCUCAAGGACGGGAGGUCCAUUGUGGUUCCCUGCCGCCACCAAGAUGAACUGAUUGGC
KU761564    UGCCCUCAGGUUCUUGAAUGAUAUGGGAAAAGUUAGGAAGGACACACAAGAGUGGAAACCCUCAACUGGAUGGGACAACUGGGAAGAAGUUCCGUUUUGCUCCCACCAUUUCAACAAGCUCCAUCUCAAGGACGGGAGGUCCAUUGUGGUUCCCUGCCGCCACCAAGAUGAACUGAUUGGC
KU740184    UGCCCUCAGGUUCUUGAAUGAUAUGGGAAAAGUUAGGAAGGACACACAAGAGUGGAAACCCUCAACUGGAUGGGACAACUGGGAAGAAGUUCCGUUUUGCUCCCACCAUUUCAACAAGCUCCAUCUCAAGGACGGGAGGUCCAUUGUGGUUCCCUGCCGCCACCAAGAUGAACUGAUUGGC
KU744693    UGCCCUCAGGUUCUUGAAUGAUAUGGGAAAAGUUAGGAAGGACACACAAGAGUGGAAACCCUCAACUGGAUGGGACAACUGGGAAGAAGUUCCCUUCUGCUCCCACCACUUCAACAAGCUCCAUCUCAAGGACGGGAGGUCCAUUGUGGUUCCCUGCCGCCACCAAGAUGAACUGAUUGGC
KU497555    UGCCCUCAGGUUCUUGAAUGAUAUGGGAAAAGUUAGGAAGGACACACAAGAGUGGAAACCCUCAACUGGAUGGGACAACUGGGAAGAAGUUCCGUUUUGCUCCCACCACUUCAACAAGCUCCAUCUCAAGGACGGGAGGUCCAUUGUGGUUCCCUGCCGCCACCAAGAUGAACUGAUUGGC
KU527068    UGCCCUCAGGUUCUUGAAUGAUAUGGGAAAAGUUAGGAAGGACACACAAGAGUGGAAACCCUCAACUGGAUGGGACAACUGGGAAGAAGUUCCGUUUUGCUCCCACCACUUCAACAAGCUCCAUCUCAAGGACGGGAGGUCCAUUGUGGUUCCCUGCCGCCACCAAGAUGAACUGAUUGGC
KU501217    UGCCCUCAGGUUCUUGAAUGAUAUGGGAAAAGUUAGGAAGGACACACAAGAGUGGAAACCCUCAACUGGAUGGGACAACUGGGAAGAAGUUCCGUUUUGCUCCCACCACUUCAACAAGCUCCAUCUCAAGGACGGGAGGUCCAUUGUGGUUCCCUGCCGCCACCAAGAUGAACUGAUUGGC
KU501216    UGCCCUCAGGUUCUUGAAUGAUAUGGGAAAAGUUAGGAAGGACACACAAGAGUGGAAACCCUCAACUGGAUGGGACAACUGGGAAGAAGUUCCGUUUUGCUCCCACCACUUCAACAAGCUCCAUCUCAAGGACGGGAGGUCCAUUGUGGUUCCCUGCCGCCACCAAGAUGAACUGAUUGGC
KU501215    UGCCCUCAGGUUCUUGAAUGAUAUGGGAAAAGUUAGGAAGGACACACAAGAGUGGAAACCCUCAACUGGAUGGGACAACUGGGAAGAAGUUCCGUUUUGCUCCCACCACUUCAACAAGCUCCAUCUCAAGGACGGGAGGUCCAUUGUGGUUCCCUGCCGCCACCAAGAUGAACUGAUUGGC
KU365780    UGCCCUCAGGUUCUUGAAUGAUAUGGGAAAAGUUAGGAAGGACACACAAGAGUGGAAACCCUCAACUGGAUGGGACAACUGGGAAGAAGUUCCGUUUUGCUCCCACCACUUCAACAAGCUCCAUCUCAAGGACGGGAGGUCCAUUGUGGUUCCCUGCCGCCACCAAGAUGAACUGAUUGGC
KU365779    UGCCCUCAGGUUCUUGAAUGAUAUGGGAAAAGUUAGGAAGGACACACAAGAGUGGAAACCCUCAACUGGAUGGGACAACUGGGAAGAAGUUCCGUUUUGCUCCCACCACUUCAACAAGCUCCAUCUCAAGGACGGGAGGUCCAUUGUGGUUCCCUGCCGCCACCAAGAUGAACUGAUUGGC
KU707826    UGCCCUCAGGUUCUUGAAUGAUAUGGGAAAAGUUAGGAAGGACACACAAGAGUGGAAACCCUCAACUGGAUGGGACAACUGGGAAGAAGUUCCGUUUUGCUCCCACCACUUCAACAAGCUCCAUCUCAAGGACGGGAGGUCCAUUGUGGUUCCCUGCCGCCACCAAGAUGAACUGAUUGGC
KU365778    UGCCCUCAGGUUCUUGAAUGAUAUGGGAAAAGUUAGAAAGGACACACAAGAGUGGAAACCCUCAACUGGAUGGGACAACUGGGAAGAAGUUCCGUUUUGCUCCCACCACUUCAACAAGCUCCAUCUCAAGGACGGGAGGUCCAUUGUGGUUCCCUGCCGCCACCAAGAUGAACUGAUUGGC
KU365777    UGCCCUCAGGUUCUUGAAUGAUAUGGGAAAAGUUAGGAAGGACACACAAGAGUGGAAACCCUCAACUGGAUGGGACAACUGGGAAGAAGUUCCGUUUUGCUCCCACCACUUCAACAAGCUCCAUCUCAAGGACGGGAGGUCCAUUGUGGUUCCCUGCCGCCACCAAGAUGAACUGAUUGGC
KU312312    UGCCCUCAGGUUCUUGAAUGAUAUGGGAAAAGUUAGGAAGGACACACAAGAGUGGAAACCCUCAACUGGAUGGGACAACUGGGAAGAAGUUCCGUUUUGCUCCCACCACUUCAACAAGCUCCAUCUCAAGGACGGGAGGUCCAUUGUGGUUCCCUGCCGCCACCAAGAUGAACUGAUUGGC
KF268950    UGCCCUCAGGUUCUUAAAUGACAUGGGAAAAGUUAGGAAAGACACACAGGAAUGGAAACCCUCGACUGGAUGGAGCAAUUGGGAAGAAGUCCCGUUCUGCUCCCACCAUUUCAACAAGCUGCACCUCAAGGAUGGUAGAUCCAUUGUGGUCCCUUGCCGCCACCAAGAUGAAUUGAUUGGC
KF268949    UGCCCUCAGGUUCUUGAAUGACAUGGGAAAAGUUAGGAAAGACACACAGGAAUGGAAACCCUCGACUGGAUGGAGCAAUUGGGAAGAAGUCCCGUUCUGCUCCCACCAUUUCAACAAGCUGCACCUCAAGGAUGGUAGAUCCAUUGUGGUCCCUUGCCGCCACCAAGAUGAAUUGAUUGGC
KF268948    UGCCCUCAGGUUCUUAAAUGACAUGGGAAAAGUUAGGAAAGACACACAGGAAUGGAAACCCUCGACUGGAUGGAGCAAUUGGGAAGAAGUCCCGUUCUGCUCCCACCAUUUCAACAAGCUGCACCUCAAGGAUGGUAGAUCCAUUGUGGUCCCUUGCCGCCACCAAGAUGAAUUGAUUGGC
KJ776791    UGCCCUCAGGUUCUUGAAUGAUAUGGGAAAAGUUAGGAAGGACACACAAGAGUGGAAACCCUCAACUGGAUGGGACAACUGGGAAGAAGUUCCGUUUUGCUCCCACCACUUCAACAAGCUCCAUCUCAAGGACGGGAGGUCCAUUGUGGUUCCCUGCCGCCACCAAGAUGAACUGAUUGGC
KF383119    UGCCCUCAGGUUCUUGAAUGACAUGGGAAAAGUUAGGAAAGACACACAGGAGUGGAAACCCUCGACUGGAUGGAGCAAUUGGGAAGAAGUCCCGUUCUGCUCCCACCACUUCAACAAGCUGUACCUCAAGGAUGGGAGAUCCAUUGUGGUCCCUUGCCGCCACCAAGAUGAACUGAUUGGC
KF383118    UGCCCUCAGGUUCUUGAAUGACAUGGGAAAAGUUAGGAAAGACACACAGGAGUGGAAACCCUCGACUGGAUGGAGCAAUUGGGAAGAAGUCCCGUUCUGCUCCCACCACUUCAACAAGCUGUACCUCAAGGAUGGGAGAUCCAUUGUGGUCCCUUGCCGCCACCAAGAUGAACUGAUUGGC
KF383117    UGCCCUCAGGUUCUUGAAUGACAUGGGAAAGGUUAGGAAAGACACACAGGAAUGGAAGCCCUCGACUGGAUGGAGCAAUUGGGAGGAGGUCCCGUUCUGUUCUCACCACUUCAACAAGCUGCACCUCAAGGACGGGAGAUCCAUUGUGGUCCCCUGCCGCCACCAAGAUGAACUGAUUGGC
KF383116    UGCCCUCAGGUUCUUGAAUGACAUGGGAAAGGUUAGGAAAGACACACAGGAAUGGAAACCCUCGACUGGAUGGAGCAAUUGGGAAGAGGUCCCGUUCUGUUCUCACCACUUCAACAAGCUGCACCUCAAGGACGGGAGAUCCAUUGUGGUCCCCUGCCGCCACCAAGAUGAACUGAUUGGC
KF383115    CUGCCUCAGGUUCCUAAACGACAUGGGGAAAGUUAGGAAAGACACACAGGAAUGGAAACCCUCGACUGGAUGGAGCAAUUGGGAAGAAGUCCCUUUCUGCUCCCACCACUUCAACAAGCUGCACCUCAAGGAUGGUAGAUCCAUUGUGGUCCCUUGCCGCCACCAAGAUGAACUGAUUGGC
EU545988    UGCCCUCAGGUUCUUGAAUGAUAUGGGAAAAGUCAGGAAGGACACACAAGAGUGGAAACCCUCAACUGGAUGGGACAAUUGGGAAGAAGUUCCGUUUUGCUCCCACCACUUCAACAAGCUCCAUCUCAAGGACGGGAGGUCCAUUGUGGUUCCCUGCCGCCACCAAGAUGAACUGAUUGGC
KU955589    UGCCCUCAGGUUCUUGAAUGAUAUGGGGAAAGUUAGGAAGGACACACAAGAGUGGAAACCCUCAACUGGAUGGGACAACUGGGAGGAAGUUCCGUUUUGCUCCCACCACUUCAACAAGCUCCAUCUCAAGGACGGGAGGUCCAUUGUGGUUCCCUGCCGCCACCAAGAUGAACUGAUUGGC
KU955590    UGCCCUCAGGUUCUUGAAUGAUAUGGGAAAAGUUAGGAAGGACACACAAGAGUGGAAACCCUCAACUGGAUGGGACAACUGGGAAGAAGUUCCGUUUUGCUCCCACCAUUUCAACAAGCUCCAUCUCAAGGACGGGAGGUCCAUUGUGGUUCCCUGCCGCCACCAAGAUGAACUGAUUGGC
KU940228    UGCCCUCAGGUUCUUGAAUGAUAUGGGAAAAGUUAGGAAGGACACACAAGAGUGGAAACCCUCAACUGGAUGGGACAACUGGGAAGAAGUUCCGUUUUGCUCCCACCACUUCAACAAGCUCCAUCUCAAGGACGGGAGGUCCAUUGUGGUUCCCUGCCGCCACCAAGAUGAACUGAUUGGC
KU991811    UGCCCUCAGGUUCUUGAAUGAUAUGGGAAAAGUUAGGAAGGACACACAAGAGUGGAAACCCUCAACUGGAUGGGACAACUGGGAAGAAGUUCCGUUUUGCUCCCACCACUUCAACAAGCUCCAUCUCAAGGACGGGAGGUCCAUUGUGGUUCCCUGCCGCCACCAAGAUGAACUGAUUGGC
KU509998    UGCCCUCAGGUUCUUGAAUGAUAUGGGAAAAGUUAGGAAGGACACACAAGAGUGGAAACCCUCAACUGGAUGGGACAACUGGGAAGAAGUUCCGUUUUGCUCCCACCACUUCAACAAGCUCCAUCUCAAGGACGGGAGGUCCAUUGUGGUUCCCUGCCGCCACCAAGAUGAACUGAUUGGC
KX056898    UGCCCUCAGGUUCUUGAAUGAUAUGGGAAAAGUUAGGAAGGACACACAAGAGUGGAAACCCUCAACUGGAUGGGACAACUGGGAAGAAGUUCCGUUUUGCUCCCACCAUUUCAACAAGCUCCAUCUCAAGGACGGGAGGUCCAUUGUGGUUCCCUGCCGCCACCAAGAUGAACUGAUUGGC
KX117076    UGCCCUCAGGUUCUUGAAUGAUAUGGGAAAAGUUAGGAAGGACACACAAGAGUGGAAACCCUCAACUGGAUGGGACAACUGGGAGGAAGUUCCGUUUUGCUCCCACCACUUCAACAAGCUCCAUCUCAAGGACGGGAGGUCCAUUGUGGUUCCCUGCCGCCACCAAGAUGAACUGAUUGGC
KX185891    UGCCCUCAGGUUCUUGAAUGAUAUGGGGAAAGUUAGGAAGGACACACAAGAGUGGAAACCCUCAACUGGAUGGGACAACUGGGAGGAAGUUCCGUUUUGCUCCCACCACUUCAACAAGCUCCAUCUCAAGGACGGGAGGUCCAUUGUGGUUCCCUGCCGCCACCAAGAUGAACUGAUUGGC
KX247646    UGCCCUCAGGUUCUUGAAUGAUAUGGGAAAAGUUAGGAAGGACACACAAGAGUGGAAACCCUCAACUGGAUGGGACAACUGGGAAGAAGUUCCGUUUUGCUCCCACCACUUCAACAAGCUCCAUCUCAAGGACGGGAGGUCCAUUGUGGUUCCCUGCCGCCACCAAGAUGAACUGAUUGGC
KX262887    UGCCCUCAGGUUCUUGAAUGAUAUGGGAAAAGUUAGGAAGGACACACAAGAGUGGAAACCCUCAACUGGAUGGGACAACUGGGAAGAAGUUCCGUUUUGCUCCCACCACUUCAACAAGCUCCAUCUCAAGGACGGGAGGUCCAUUGUGGUUCCCUGCCGCCACCAAGAUGAACUGAUUGGC
KX280026    UGCCCUCAGGUUCUUGAAUGAUAUGGGAAAAGUUAGGAAGGACACACAAGAGUGGAAACCCUCAACUGGAUGGGACAACUGGGAAGAAGUUCCGUUUUGCUCCCACCACUUCAACAAGCUCCAUCUCAAGGACGGGAGGUCCAUUGUGGUUCCCUGCCGCCACCAAGAUGAACUGAUUGGC
KU758877    UGCCCUCAGGUUCUUGAAUGAUAUGGGAAAAGUUAGGAAGGACACACAAGAGUGGAAACCCUCAACUGGAUGGGACAACUGGGAAGAAGUUCCGUUUUGCUCCCACCACUUCAACAAGCUCCAUCUCAAGGACGGGAGGUCCAUUGUGGUUCCCUGCCGCCACCAAGAUGAACUGAUUGGC
KU866423    UGCCCUCAGGUUCUUGAAUGAUAUGGGGAAAGUUAGGAAGGACACACAAGAGUGGAAACCCUCAACUGGAUGGGACAACUGGGAGGAAGUUCCGUUUUGCUCCCACCACUUCAACAAGCUCCAUCUCAAGGACGGGAGGUCCAUUGUGGUUCCCUGCCGCCACCAAGAUGAACUGAUUGGC
KX377336    UGCCCUCAGGUUUUUGAAUGACAUGGGGAAAGUUAGGAAGGACACACAGGAGUGGAAACCCUCAACUGGAUGGAGCAACUGGGAAGAAGUUCCGUUUUGCUCCUAUCACUUCAACAAGCUUUACCUCAAGGACGGGAGGUCCAUUGUGGUCCCCUGUCGCCACCAAGAUGAACUGAUUGGC
KX377337    UGCCCUCAGGUUCUUGAAUGAUAUGGGAAAAGUUAGGAAGGACACACAAGAGUGGAAACCCUCAACUGGAUGGGACAACUGGGAAGAAGUUCCGUUUUGCUCCCACCACUUCAACAAGCUCCAUCUCAAGGACGGGAGGUCCAUUGUGGUUCCCUGCCGCCACCAAGAUGAACUGAUUGGC
KX369547    UGCCCUCAGGUUCUUGAAUGAUAUGGGAAAAGUUAGGAAGGACACACAAGAGUGGAAACCCUCAACUGGAUGGGACAACUGGGAAGAAGUUCCGUUUUGCUCCCACCACUUCAACAAGCUCCAUCUCAAGGACGGGAGGUCCAUUGUGGUUCCCUGCCGCCACCAAGAUGAACUGAUUGGC
KX548902    UGCCCUCAGGUUCUUGAAUGAUAUGGGAAAAGUUAGGAAGGACACACAAGAGUGGAAACCCUCAACUGGAUGGGACAACUGGGAAGAAGUUCCGUUUUGCUCCCACCACUUCAACAAGCUCCAUCUCAAGGACGGGAGGUCCAUUGUGGUUCCCUGCCGCCACCAAGAUGAACUGAUUGGC
KX601166    UGCCCUCAGGUUCUUGAAUGACAUGGGAAAGGUUAGGAAAGACACACAGGAAUGGAAACCCUCGACUGGAUGGAGCAAUUGGGAAGAGGUCCCGUUCUGCUCUCACCACUUCAACAAGCUGCACCUCAAGGACGGGAGAUCCAUUGUGGUCCCCUGCCGCCACCAAGAUGAACUGAUUGGC
KX601167    UGCCCUCAGGUUUUUGAAUGACAUGGGGAAAGUUAGGAAGGACACACAGGAGUGGAAACCCUCAACUGGAUGGAGCAACUGGGAAGAAGUUCCGUUUUGCUCCCAUCACUUCAACAAGCUUUACCUCAAGGACGGGAGGUCCAUUGUGGUCCCCUGUCGCCACCAAGAUGAACUGAUUGGC
KX601168    UGCCCUCAGGUUCUUGAAUGAUAUGGGAAAAGUUAGGAAGGACACACAAGAGUGGAAACCCUCAACUGGAUGGGACAACUGGGAAGAAGUUCCGUUUUGCUCCCACCACUUCAACAAGCUCCAUCUCAAGGACGGGAGGUCCAUUGUGGUUCCCUGCCGCCACCAAGAUGAACUGAUUGGC
KX766028    UGCCCUCAGGUUCUUGAAUGAUAUGGGAAAAGUUAGGAAGGACACACAAGAGUGGAAACCCUCAACUGGAUGGGACAACUGGGAAGAAGUUCCGUUUUGCUCCCACCAUUUCAACAAGCUCCAUCUCAAGGACGGGAGGUCCAUUGUGGUUCCCUGCCGCCACCAAGAUGAACUGAUUGGC
KX766029    UGCCCUCAGGUUCUUGAAUGAUAUGGGAAAAGUUAGGAAGGACACACAAGAGUGGAAACCCUCAACUGGAUGGGACAACUGGGAAGAAGUUCCGUUUUGCUCCCACCACUUCAACAAGCUCCAUCUCAAGGACGGGAGGUCCAUUGUGGUUCCCUGCCGCCACCAAGAUGAACUGAUUGGC
KU940224    UGCCCUCAGGUUCUUGAAUGAUAUGGGAAAAGUUAGGAAGGACACACAAGAGUGGAAACCCUCAACUGGAUGGGACAACUGGGAAGAAGUUCCGUUUUGCUCCCACCACUUCAACAAGCUCCAUCUCAAGGACGGGAGGUCCAUUGUGGUUCCCUGCCGCCACCAAGAUGAACUGAUUGGC


            9999999999999999999999999999999999999999999999999999999999999999999999999999999999999999999999999999999999999999999999999999999999999999999999999999999999999999999999999999999
            7777777777777777777777777888888888888888888888888888888888888888888888888888888888888888888888888888888888888888888888888999999999999999999999999999999999999999999999999999999
            7777788888888889999999999000000000011111111112222222222333333333344444444445555555555666666666677777777778888888888999999999900000000001111111111222222222233333333334444444444555555
Sequence    5678901234567890123456789012345678901234567890123456789012345678901234567890123456789012345678901234567890123456789012345678901234567890123456789012345678901234567890123456789012345
-----------------------------------------------------------------------------------------------------------------------------------------------------------------------------------------------------
NC_012532   CGAGCUCGCGUCUCACCAGGGGCAGGAUGGAGCAUCCGGGAGACUGCCUGUCUUGCAAAAUCAUAUGCGCAGAUGUGGCAGCUCCUUUAUUUCCACAGAAGAGACCUUCGACUGAUGGCUAAUGCCAUUUGCUCGGCUGUGCCAGUUGACUGGGUACCAACUGGGAGAACCACCUGGUCAA
KU922960    CGGGCCCGCGUCUCUCCAGGGGCGGGAUGGAGCAUCCGGGAGACUGCUUGCCUAGCAAAAUCAUAUGCGCAAAUGUGGCAGCUCCUUUAUUUCCACAGAAGGGACCUCCGACUGAUGGCCAAUGCCAUUUGUUCAUCUGUGCCAGUUGACUGGGUUCCAACUGGGAGAACUACCUGGUCAA
KU922923    CGGGCCCGCGUCUCUCCAGGGGCGGGAUGGAGCAUCCGGGAGACUGCUUGCCUAGCAAAAUCAUAUGCGCAAAUGUGGCAGCUCCUUUAUUUCCACAGAAGGGACCUCCGACUGAUGGCCAAUGCCAUUUGUUCAUCUGUGCCAGUUGACUGGGUUCCAACUGGGAGAACUACCUGGUCAA
KU820898    CGGGCCCGCGUCUCUCCAGGGGCGGGAUGGAGCAUCCGGGAGACUGCUUGCCUAGCAAAAUCAUAUGCGCAAAUGUGGCAGCUCCUUUAUUUCCACAGAAGGGACCUCCGACUGAUGGCCAAUGCCAUUUGUUCAUCUGUGCCAGUUGACUGGGUUCCAACUGGGAGAACUACCUGGUCAA
KU321639    CGGGCCCGCGUCUCUCCAGGGGCGGGAUGGAGCAUCCGGGAGACUGCUUGCCUAGCAAAAUCAUAUGCGCAAAUGUGGCAGCUCCUUUAUUUCCACAGAAGGGACCUCCGACUGAUGGCCAAUGCCAUUUGUUCAUCUGUGCCAGUUGACUGGGUUCCAACUGGGAGAACUACCUGGUCAA
KU820899    CGGGCCCGCGUCUCUCCAGGGGCGGGAUGGAGCAUCCGGGAGACUGCUUGCCUAGCAAAAUCAUAUGCGCAAAUGUGGCAGCUCCUUUAUUUCCACAGAAGGGACCUCCGACUGAUGGCCAAUGCCAUUUGUUCAUCUGUGCCAGUUGACUGGGUUCCAACUGGGAGAACUACCUGGUCAA
KU853012    CGGGCCCGCGUCUCUCCAGGGGCGGGAUGGAGCAUCCGGGAGACUGCUUGCCUAGCAAAAUCAUAUGCGCAAAUGUGGCAGCUCCUUUAUUUCCACAGAAGGGACCUCCGACUGAUGGCCAAUGCCAUUUGUUCAUCUGUGCCAGUUGACUGGGUUCCAACUGGGAGAACUACCUGGUCAA
KU729217    CGGGCCCGCGUCUCUCCAGGGGCGGGAUGGAGCAUCCGGGAGACUGCUUGCCUAGCAAAAUCAUAUGCGCAAAUGUGGCAGCUCCUUUAUUUCCACAGAAGGGACCUCCGACUGAUGGCCAAUGCCAUUUGUUCAUCUGUGCCAGUUGACUGGGUUCCAACUGGGAGAACUACCUGGUCAA
KU729218    CGGGCCCGCGUCUCUCCAGGGGCGGGAUGGAGCAUCCGGGAGACUGCUUGCCUAGCAAAAUCAUAUGCGCAAAUGUGGCAGCUCCUUUAUUUCCACAGAAGGGACCUCCGACUGAUGGCCAAUGCCAUUUGUUCAUCUGUGCCAGUUGACUGGGUUCCAACUGGGAGAACUACCUGGUCAA
KU761564    CGGGCCCGCGUCUCUCCAGGGGCGGGAUGGAGCAUCCGGGAGACUGCUUGCCUAGCAAAAUCAUAUGCGCAAAUGUGGCAGCUCCUUUAUUUCCACAGAAGGGACCUCCGACUGAUGGCCAAUGCCAUUUGUUCAUCUGUGCCAGUUGACUGGGUUCCAACUGGGAGAACUACCUGGUCAA
KU740184    CGGGCCCGCGUCUCUCCAGGGGCGGGAUGGAGCAUCCGGGAGACUGCUUGCCUAGCAAAAUCAUAUGCGCAAAUGUGGCAGCUCCUUUAUUUCCACAGAAGGGACCUCCGACUGAUGGCCAAUGCCAUUUGUUCAUCUGUGCCAGUUGACUGGGUUCCAACUGGGAGAACUACCUGGUCAA
KU744693    CGGGCCCGCGUCUCUCCAGGGGCGGGAUGGAGCAUCCGGGAGACUGCUUGCCUAGCAAAAUCAUAUGCGCAAAUGUGGCAGCUCCUUUAUUUCCACAGAAGGGACCUCCGACUGAUGGCCAAUGCCAUUUGUUCAUCUGUGCCAGUUGACUGGGUUCCAACUGGGAGAACUACCUGGUCAA
KU497555    CGGGCCCGCGUCUCUCCAGGGGCGGGAUGGAGCAUCCGGGAGACUGCUUGCCUAGCAAAAUCAUAUGCGCAAAUGUGGCAGCUCCUUUAUUUCCACAGAAGGGACCUCCGACUGAUGGCCAAUGCCAUUUGUUCAUCUGUGCCAGUUGACUGGGUUCCAACUGGGAGAACUACCUGGUCAA
KU527068    CGGGCCCGCGUCUCUCCAGGGGCGGGAUGGAGCAUCCGGGAGACUGCUUGCCUAGCAAAAUCAUAUGCGCAAAUGUGGCAGCUCCUUUAUUUCCACAGAAGGGACCUCCGACUGAUGGCCAAUGCCAUUUGUUCAUCUGUGCCAGUUGACUGGGUUCCAACUGGGAGAACUACCUGGUCAA
KU501217    CGGGCCCGCGUCUCUCCAGGGGCGGGAUGGAGCAUCCGGGAGACUGCUUGCCUAGCAAAAUCAUAUGCGCAAAUGUGGCAGCUCCUUUAUUUCCACAGAAGGGACCUCCGACUGAUGGCCAAUGCCAUUUGUUCAUCUGUGCCAGUUGACUGGGUUCCAACUGGGAGAACUACCUGGUCAA
KU501216    CGGGCCCGCGUCUCUCCAGGGGCGGGAUGGAGCAUCCGGGAGACUGCUUGCCUAGCAAAAUCAUAUGCGCAAAUGUGGCAGCUCCUUUAUUUCCACAGAAGGGACCUCCGACUGAUGGCCAAUGCCAUUUGUUCAUCUGUGCCAGUUGACUGGGUUCCAACUGGGAGAACUACCUGGUCAA
KU501215    CGGGCCCGCGUCUCUCCAGGGGCGGGAUGGAGCAUCCGGGAGACUGCUUGCCUAGCAAAAUCAUAUGCGCAAAUGUGGCAGCUCCUUUAUUUCCACAGAAGGGACCUCCGACUGAUGGCCAAUGCCAUUUGUUCAUCUGUGCCAGUUGACUGGGUUCCAACUGGGAGAACUACCUGGUCAA
```

```
KU365780    CGGGCCCGCGUCUCUCCAGGGGCGGGAUGGAGCAUCCGGGAGACUGCUUGCCUAGCAAAAUCAUAUGCGCAAAUGUGGCAGCUCCUUUAUUUCCACAGAAGGGACCUCCGACUGAUGGCCAAUGCCAUUUGUUCAUCUGUGCCAGUUGACUGGGUUCCAACUGGGAGAACUACCUGGUCAA
KU365779    CGGGCCCGCGUCUCUCCAGGGGCGGGAUGGAGCAUCCGGGAGACUGCUUGCCUAGCAAAAUCAUAUGCGCAAAUGUGGCAGCUCCUUUAUUUCCACAGAAGGGACCUCCGACUGAUGGCCAAUGCCAUUUGUUCAUCUGUGCCAGUUGACUGGGUUCCAACUGGGAGAACUACCUGGUCAA
KU707826    CGGGCCCGCGUCUCUCCAGGGGCGGGAUGGAGCAUCCGGGAGACUGCUUGCCUAGCAAAAUCAUAUGCGCAAAUGUGGCAGCUCCUUUAUUUCCACAGAAGGGACCUCCGACUGAUGGCCAAUGCCAUUUGUUCAUCUGUGCCAGUUGACUGGGUUCCAACUGGGAGAACUACCUGGUCAA
KU365778    CGGGCCCGCGUCUCUCCAGGGGCGGGAUGGAGCAUCCGGGAGACUGCUUGCCUAGCAAAAUCAUAUGCGCAGAUGUGGCAGCUCCUUUAUUUCCACAGAAGGGACCUCCGACUGAUGGCCAAUGCCAUUUGUUCAUCUGUGCCAGUUGACUGGGUUCCAACUGGGAGAACUACCUGGUCAA
KU365777    CGGGCCCGCGUCUCUCCAGGGGCGGGAUGGAGCAUCCGGGAGACUGCUUGCCUAGCAAAAUCAUAUGCGCAAAUGUGGCAGCUCCUUUAUUUCCACAGAAGGGACCUCCGACUGAUGGCCAAUGCCAUUUGUUCAUCUGUGCCAGUUGACUGGGUUCCAACUGGGAGAACUACCUGGUCAA
KU312312    CGGGCCCGCGUCUCUCCAGGGGCGGGAUGGAGCAUCCGGGAGACUGCUUGCCUAGCAAAAUCAUAUGCGCAAAUGUGGCAGCUCCUUUAUUUCCACAGAAGGGACCUCCGACUGAUGGCCAAUGCCAUUUGUUCAUCUGUGCCAGUUGACUGGGUUCCAACUGGGAGAACUACCUGGUCAA
KF268950    CGAGCCCGUGUAUCACCAGGGGCAGGAUGGAGCAUUCGGGAGACUGCCUGUCUAGCAAAAUCAUAUGCACAGAUGUGGCAGCUUCUUUACUUCCACAGAAGAGACCUUCGACUGAUGGCCAAUGCUAUUUGUUCGGCUGUGCCAGUUGACUGGGUACCAACCGGGAGAACCACCUGGUCAA
KF268949    CGAGCCCGUGUAUCACCAGGGGCAGGAUGGAGCAUCCGGGAGACUGCCUGUCUAGCAAAAUCAUAUGCACAGAUGUGGCAGCUUCUUUACUUCCACAGAAGAGACCUUCGACUGAUGGCCAAUGCUAUUUGUUCGGCUGUGCCAGUUGACUGGGUGCCAACCGGGAGAACCACCUGGUCAA
KF268948    CGAGCCCGUGUAUCACCAGGGGCAGGAUGGAGCAUUCGGGAGACUGCCUGUCUAGCAAAAUCAUAUGCACAGAUGUGGCAGCUUCUUUACUUCCACAGAAGAGACCUUCGACUGAUGGCCAAUGCUAUUUGUUCGGCUGUGCCAGUUGACUGGGUACCAACCGGGAGAACCACCUGGUCAA
KJ776791    CGGGCCCGCGUCUCUCCAGGGGCGGGAUGGAGCAUCCGGGAGACUGCUUGCCUAGCAAAAUCAUAUGCGCAAAUGUGGCAGCUCCUUUAUUUCCACAGAAGGGACCUCCGACUGAUGGCCAAUGCCAUUUGUUCAUCUGUGCCAGUUGACUGGGUUCCAACUGGGAGAACUACCUGGUCAA
KF383119    CGAGCUCGCGUUUCACCAGGGGCAGGAUGGAGCAUCCGGGAGACUGCCUGUCUUGCAAAAUCAUAUGCGCAGAUGUGGCAGCUCCUUUAUUUCCACAGAAGAGACCUUCGACUGAUGGCUAAUGCCAUUUGCUCGGCUGUACCAGUUGACUGGGUACCAACUGGGAGAACCACCUGGUCAA
KF383118    CGAGCUCGCGUUUCACCAGGGGCAGGAUGGAGCAUCCGGGAGACUGCCUGUCUUGCAAAAUCAUAUGCGCAGAUGUGGCAGCUCCUUUAUUUCCACAGAAGAGACCUUCGACUGAUGGCUAAUGCCAUUUGCUCGGCUGUACCAGUUGACUGGGUACCAACUGGGAGAACCACCUGGUCAA
KF383117    CGAGCCCGUGUCUCACCAGGGGCAGGAUGGAGCAUCCGUGAGACUGCUUGCCUUGCAAAAUCAUAUGCACAGAUGUGGCAGCUUCUUUAUUUCCACAGAAGAGAUCUCCGACUGAUGGCCAAUGCCAUUUGUUCGGCUGUGCCAGUUGAUUGGGUACCAACUGGGAGAACCACCUGGUCAA
KF383116    CGAGCCCGUGUCUCACCAGGGGCAGGAUGGAGCAUCCGGGAGACUGCUUGUCUUGCAAAAUCAUAUGCACAGAUGUGGCAGCUUCUUUAUUUCCACAAAAGAGACCUCCGACUGAUGGCCAAUGCCAUUUGUUCGGCUGUGCCAGUUGACUGGGUACCAACUGGGAGAACCACCUGGUCAA
KF383115    CGAGCCCGUGUAUCACCAGGGGCAGGAUGGAGCAUCCGGGAGACUGCCUGUCUUGCAAAAUCAUAUGCACAGAUGUGGCAGCUUCUUUACUUCCACAGAAGAGACCUUCGACUGAUGGCCAACGCCAUUUGUUCGGCUGUGCCAGUCGACUGGGUACCAACCGGGAGAACCACCUGGUCAA
EU545988    CGAGCCCGCGUCUCACCAGGGGCGGGAUGGAGCAUCCGGGAGACUGCUUGCCUAGCAAAAUCAUAUGCGCAAAUGUGGCAGCUCCUUUAUUUCCACAGAAGGGACCUCCGACUGAUGGCCAAUGCCAUUUGUUCAUCUGUGCCAGUUGACUGGGUUCCAACUGGGAGAACUACCUGGUCAA
KU955589    CGGGCCCGCGUCUCUCCAGGGGCGGGAUGGAGCAUCCGGGAGACUGCUUGCCUAGCAAAAUCAUAUGCGCAAAUGUGGCAGCUCCUUUAUUUCCACAGAAGGGACCUCCGACUGAUGGCCAAUGCCAUUUGUUCAUCUGUGCCAGUUGACUGGGUUCCAACUGGGAGAACUACCUGGUCAA
KU955590    CGGGCCCGCGUCUCUCCAGGGGCGGGAUGGAGCAUCCGGGAGACUGCUUGCCUAGCAAAAUCAUAUGCGCAAAUGUGGCAGCUCCUUUAUUUCCACAGAAGGGACCUCCGACUGAUGGCCAAUGCCAUUUGUUCAUCUGUGCCAGUUGACUGGGUUCCAACUGGGAGAACUACCUGGUCAA
KU940228    CGGGCCCGCGUCUCUCCAGGGGCGGGAUGGAGCAUCCGGGAGACUGCUUGCCUAGCAAAAUCAUAUGCGCAAAUGUGGCAGCUCCUUUAUUUCCACAGAAGGGACCUCCGACUGAUGGCCAAUGCCAUUUGCUCAUCUGUGCCAGUUGACUGGGUUCCAACUGGGAGAACUACCUGGUCAA
KU991811    CGGGCCCGCGUCUCUCCAGGGGCGGGAUGGAGCAUCCGGGAGACUGCUUGCCUAGCAAAAUCAUAUGCGCAGAUGUGGCAGCUCCUUUAUUUCCACAGAAGGGACCUCCGACUGAUGGCCAAUGCCAUUUGUUCAUCUGUGCCAGUUGACUGGGUUCCAACUGGGAGAACUACCUGGUCAA
KU509998    CGGGCCCGCGUCUCUCCAGGGGCGGGAUGGAGCAUCCGGGAGACUGCUUGCCUAGCAAAAUCAUAUGCGCAAAUGUGGCAGCUCCUUUAUUUCCACAGAAGGGACCUCCGACUGAUGGCCAAUGCCAUUUGUUCAUCUGUGCCAGUUGACUGGGUUCCAACUGGGAGAACUACCUGGUCAA
KX056898    CGGGCCCGCGUCUCUCCAGGGGCGGGAUGGAGCAUCCGGGAGACUGCUUGCCUAGCAAAAUCAUAUGCGCAAAUGUGGCAGCUCCUUUAUUUCCACAGAAGGGACCUCCGACUGAUGGCCAAUGCCAUUUGUUCAUCUGUGCCAGUUGACUGGGUUCCAACUGGGAGAACUACCUGGUCAA
KX117076    CGGGCCCGCGUCUCUCCAGGGGCGGGAUGGAGCAUCCGGGAGACUGCUUGCCUAGCAAAAUCAUAUGCGCAAAUGUGGCAGCUCCUUUAUUUCCACAGAAGGGACCUCCGACUGAUGGCCAAUGCCAUUUGUUCAUCUGUGCCAGUUGACUGGGUUCCAACUGGGAGAACUACCUGGUCAA
KX185891    CGGGCCCGCGUCUCUCCAGGGGCGGGAUGGAGCAUCCGGGAGACUGCUUGCCUAGCAAAAUCAUAUGCGCAAAUGUGGCAGCUCCUUUAUUUCCACAGAAGGGACCUCCGACUGAUGGCCAAUGCCAUUUGUUCAUCUGUGCCAGUUGACUGGGUUCCAACUGGGAGAACUACCUGGUCAA
KX247646    CGGGCCCGCGUCUCUCCAGGGGCGGGAUGGAGCAUCCGGGAGACUGCUUGCCUAGCAAAAUCAUAUGCGCAAAUGUGGCAGCUCCUUUAUUUCCACAGAAGGGACCUCCGACUGAUGGCCAAUGCCAUUUGUUCAUCUGUGCCAGUUGACUGGGUUCCAACUGGGAGAACUACCUGGUCAA
KX262887    CGGGCCCGCGUCUCUCCAGGGGCGGGAUGGAGCAUCCGGGAGACUGCUUGCCUAGCAAAAUCAUAUGCGCAAAUGUGGCAGCUCCUUUAUUUCCACAGAAGGGACCUCCGACUGAUGGCCAAUGCCAUUUGUUCAUCUGUGCCAGUUGACUGGGUUCCAACUGGGAGAACUACCUGGUCAA
KX280026    CGGGCCCGCGUCUCUCCAGGGGCGGGAUGGAGCAUCCGGGAGACUGCUUGCCUAGCAAAAUCAUAUGCGCAAAUGUGGCAGCUCCUUUAUUUCCACAGAAGGGACCUCCGACUGAUGGCCAAUGCCAUUUGUUCAUCUGUGCCAGUUGACUGGGUUCCAACUGGGAGAACUACCUGGUCAA
KU758877    CGGGCCCGCGUCUCUCCAGGGGCGGGAUGGAGCAUCCGGGAGACUGCUUGCCUAGCAAAAUCAUAUGCGCAAAUGUGGCAGCUCCUUUAUUUCCACAGAAGGGACCUCCGACUGAUGGCCAAUGCCAUUUGUUCAUCUGUGCCAGUUGACUGGGUUCCAACUGGGAGAACUACCUGGUCAA
KU866423    CGGGCCCGCGUCUCUCCAGGGGCGGGAUGGAGCAUCCGGGAGACUGCUUGCCUAGCAAAAUCAUAUGCGCAAAUGUGGCAGCUCCUUUAUUUCCACAGAAGGGACCUCCGACUGAUGGCCAAUGCCAUUUGUUCAUCUGUGCCAGUUGACUGGGUUCCAACUGGGAGAACUACCUGGUCAA
KX377336    CGAGCCCGCGUCUCACCAGGGGCGGGAUGGAGCAUCCGGGAGACUGCUUGCCUAGCAAAAUCAUAUGCACAAAUGUGGCAGCUUCUUUAUUUCCACAGAAGGGACCUCCGACUGAUGGCCAACGCCAUUUGUUCAUCUGUGCCAGUUGACUGGGUUCCAACUGGGAGAACCACCUGGUCAA
KX377337    CGGGCCCGCGUCUCUCCAGGGGCGGGAUGGAGCAUCCGGGAGACUGCUUGCCUAGCAAAAUCAUAUGCGCAAAUGUGGCAGCUCCUUUAUUUCCACAGAAGGGACCUCCGACUGAUGGCCAAUGCCAUUUGUUCAUCUGUGCCAGUUGACUGGGUUCCAACUGGGAGAACUACCUGGUCAA
KX369547    CGGGCCCGCGUCUCUCCAGGGGCGGGAUGGAGCAUCCGGGAGACUGCUUGCCUAGCAAAAUCAUAUGCGCAAAUGUGGCAGCUCCUUUAUUUCCACAGAAGGGACCUCCGACUGAUGGCCAAUGCCAUUUGUUCAUCUGUGCCAGUUGACUGGGUUCCAACUGGGAGAACUACCUGGUCAA
KX548902    CGGGCCCGCGUCUCUCCAGGGGCGGGAUGGAGCAUCCGGGAGACUGCUUGCCUAGCAAAAUCAUAUGCGCAAAUGUGGCAGCUCCUUUAUUUCCACAGAAGGGACCUCCGACUGAUGGCCAAUGCCAUUUGUUCAUCUGUGCCAGUUGACUGGGUUCCAACUGGGAGAACUACCUGGUCAA
KX601166    CGAGCCCGUGUCUCACCAGGGGCAGGAUGGAGCAUCCGGGAGACUGCUUGUCUUGCAAAAUCAUAUGCACAGAUGUGGCAGCUUCUUUAUUUCCACAGAAGAGACCUCCGACUGAUGGCCAAUGCCAUUUGUUCGGCUGUGCCAAUUGACUGGGUACCAACUGGGAGAACCACCUGGUCAA
KX601167    CGAGCCCGCGUCUCACCAGGGGCGGGAUGGAGCAUCCGGGAGACUGCUUGCCUAGCAAAAUCAUAUGCACAAAUGUGGCAGCUUCUUUAUUUCCACAGAAGGGACCUCCGACUGAUGGCCAACGCCAUUUGUUCAUCUGUGCCAGUUGACUGGGUUCCAACUGGGAGAACCACCUGGUCAA
KX601168    CGGGCCCGCGUCUCUCCAGGGGCGGGAUGGAGCAUCCGGGAGACUGCUUGCCUAGCAAAAUCAUAUGCGCAAAUGUGGCAGCUCCUUUAUUUCCACAGAAGGGACCUCCGACUGAUGGCCAAUGCCAUUUGUUCAUCUGUGCCAGUUGACUGGGUUCCAACUGGGAGAACUACCUGGUCAA
KX766028    CGGGCCCGCGUCUCUCCAGGGGCGGGAUGGAGCAUCCGGGAGACUGCUUGCCUAGCAAAAUCAUAUGCGCAAAUGUGGCAGCUCCUUUAUUUCCACAGAAGGGACCUCCGACUGAUGGCCAAUGCCAUUUGUUCAUCUGUGCCAGUUGACUGGGUUCCAACUGGGAGAACUACCUGGUCAA
KX766029    CGGGCCCGCGUCUCUCCAGGGGCGGGAUGGAGCAUCCGGGAGACUGCUUGCCUAGCAAAAUCAUAUGCGCAAAUGUGGCAGCUCCUUUAUUUCCACAGAAGGGACCUCCGACUGAUGGCCAAUGCCAUUUGUUCAUCUGUGCCAGUUGACUGGGUUCCAACUGGGAGAACUACCUGGUCAA
KU940224    CGGGCCCGCGUCUCUCCAGGGGCGGGAUGGAGCAUCCGGGAGACUGCUUGCCUAGCAAAAUCAUAUGCGCAAAUGUGGCAGCUCCUUUAUUUCCACAGAAGGGACCUCCGACUGAUGGCCAAUGCCAUUUGCUCAUCUGUGCCAGUUGACUGGGUUCCAACUGGGAGAACUACCUGGUCAA


                                                        11111111111111111111111111111111111111111111111111111111111111111111111111111111111111111111111111111111111111111111111111111111111111111
            9999999999999999999999999999999999999999999900000000000000000000000000000000000000000000000000000000000000000000000000000000000000000000000000000
            9999999999999999999999999999999999999999999900000000000000000000000000000000000000000000000000000000000000000000000000000000000000000000001111111111111111111111111111111111111
            5555666666666677777777778888888888999999999900000000001111111111222222222233333333334444444444555555555566666666667777777777888888888899999999990000000000111111111122222222223333333
Sequence    6789012345678901234567890123456789012345678901234567890123456789012345678901234567890123456789012345678901234567890123456789012345678901234567890123456789012345678901234567890123456
-------------------------------------------------------------------------------------------------------------------------------------------------------------------------------------------------
NC_012532   UCCAUGGAAAGGGAGAAUGGAUGACCACUGAGGACAUGCUCAUGGUGUGGAAUAGAGUGUGGAUUGAGGAGAACGACCAUAUGGAGGACAAGACUCCUGUAACAAAAUGGACAGACAUUCCCUAUCUAGGAAAAAGGGAGGACUUAUGGUGUGGAUCCCUUAUAGGGCACAGACCCCGCAC
KU922960    UCCAUGGAAAGGGAGAAUGGAUGACCACUGAAGACAUGCUUGUGGUGUGGAACAGAGUGUGGAUUGAGGAGAACGACCACAUGGAAGACAAGACCCCAGUUGCGAAAUGGACAGACAUUCCCUAUUUGGGAAAAAGGGAAGACUUGUGGUGUGGAUCUCUCAUAGGGCACAGACCGCGCAC
KU922923    UCCAUGGAAAGGGAGAAUGGAUGACCACUGAAGACAUGCUUGUGGUGUGGAACAGAGUGUGGAUUGAGGAGAACGACCACAUGGAAGACAAGACCCCAGUUGCGAAAUGGACAGACAUUCCCUAUUUGGGAAAAAGGGAAGACUUGUGGUGUGGAUCUCUCAUAGGGCACAGACCGCGCAC
KU820898    UCCAUGGAAAGGGAGAAUGGAUGACCACUGAAGACAUGCUUGUGGUGUGGAACAGAGUGUGGAUUGAGGAGAACGACCACAUGGAAGACAAGACCCCAGUUACGAAAUGGACAGACAUUCCCUAUUUGGGAAAAAGGGAAGACUUGUGGUGUGGAUCUCUCAUAGGGCACAGACCGCGCAC
KU321639    UCCAUGGAAAGGGAGAAUGGAUGACCACUGAAGACAUGCUUGUGGUGUGGAACAGAGUGUGGAUUGAGGAGAACGACCACAUGGAAGACAAGACCCCAGUUACGAAAUGGACAGACAUUCCCUAUUUGGGAAAAAGGGAAGACUUGUGGUGUGGAUCUCUCAUAGGGCACAGACCGCGCAC
KU820899    UCCAUGGAAAGGGAGAAUGGAUGACCACUGAAGACAUGCUUGUGGUGUGGAACAGAGUGUGGAUUGAGGAGAACGACCACAUGGAAGACAAGACCCCAGUUACGAAAUGGACAGACAUUCCCUAUUUGGGAAAAAGGGAAGACUUGUGGUGUGGAUCUCUCAUAGGGCACAGACCGCGCAC
KU853012    UCCAUGGAAAGGGAGAAUGGAUGACCACUGAAGACAUGCUUGUGGUGUGGAACAGAGUGUGGAUUGAGGAGAACGACCACAUGGAAGACAAGACCCCAGUUACGAAAUGGACAGACAUUCCCUAUUUGGGGAAAAGGGAAGACUUGUGGUGUGGAUCUCUCAUAGGGCACAGACCGCGCAC
KU729217    UCCAUGGAAAGGGAGAAUGGAUGACCACUGAAGACAUGCUUGUGGUGUGGAACAGAGUGUGGAUUGAGGAGAACGACCACAUGGAAGACAAGACCCCAGUUACGAAAUGGACAGACAUUCCCUAUUUGGGAAAAAGGGAAGACUUGUGGUGUGGAUCUCUCAUAGGGCACAGACCGCGCAC
KU729218    UCCAUGGAAAGGGAGAAUGGAUGACCACUGAAGACAUGCUUGUGGUGUGGAACAGAGUGUGGAUUGAGGAGAACGACCACAUGGAAGACAAGACCCCAGUUACGAAAUGGACAGACAUUCCCUAUUUGGGAAAAAGGGAAGACUUGUGGUGUGGAUCUCUCAUAGGGCACAGACCGCGCAC
KU761564    UCCAUGGAAAGGGAGAAUGGAUGACCACUGAAGACAUGCUUGUGGUGUGGAACAGAGUGUGGAUUGAGGAGAACGACCACAUGGAAGACAAGACCCCAGUUACGAAAUGGACAGACAUUCCCUAUUUGGGAAAAAGGGAAGACUUGUGGUGUGGAUCUCUCAUAGGGCACAGACCGCGCAC
KU740184    UCCAUGGAAAGGGAGAAUGGAUGACCACUGAAGACAUGCUUGUGGUGUGGAACAGAGUGUGGAUUGAGGAGAACGACCACAUGGAAGACAAGACCCCAGUUACGAAAUGGACAGACAUUCCCUAUUUGGGAAAAAGGGAAGACUUGUGGUGUGGAUCUCUCAUAGGGCACAGACCGCGCAC
KU744693    UCCAUGGAAAGGGAGAAUGGAUGACCACUGAAGACAUGCUUGUGGCGUGGAACAGAGUGUGGAUUGAGGAGAACGACCACAUGGAAGACAAGACCCCAGUCACGAAAUGGACAGACAUUCCCUAUUUGGGAAAAAGGGAAGACUUGUGGUGUGGAUCUCUCAUAGGGCACAGACCGCGCAC
KU497555    UCCAUGGAAAGGGAGAAUGGAUGACCACUGAAGACAUGCUUGUGGUGUGGAACAGAGUGUGGAUUGAGGAGAACGACCACAUGGAAGACAAGACCCCAGUUACGAAAUGGACAGACAUUCCCUAUUUGGGAAAAAGGGAAGACUUGUGGUGUGGAUCUCUCAUAGGGCACAGACCGCGCAC
KU527068    UCCAUGGAAAGGGAGAAUGGAUGACCACUGAAGACAUGCUUGUGGUGUGGAACAGAGUGUGGAUUGAGGAGAACGACCACAUGGAAGACAAGACCCCAGUUACGAAAUGGACAGACAUUCCCUAUUUGGGAAAAAGGGAAGACUUGUGGUGUGGAUCUCUCAUAGGGCACAGACCGCGCAC
KU501217    UCCAUGGAAAGGGAGAAUGGAUGACCACUGAAGACAUGCUUGUGGUGUGGAACAGAGUGUGGAUUGAGGAGAACGACCACAUGGAAGACAAGACCCCAGUUACGAAAUGGACAGACAUUCCCUAUUUGGGAAAAAGGGAAGACUUGUGGUGUGGAUCUCUCAUAGGGCACAGACCGCGCAC
KU501216    UCCAUGGAAAGGGAGAAUGGAUGACCACUGAAGACAUGCUUGUGGUGUGGAACAGAGUGUGGAUUGAGGAGAACGACCACAUGGAAGACAAGACCCCAGUUACGAAAUGGACAGACAUUCCCUAUUUGGGAAAAAGGGAAGACUUGUGGUGUGGAUCUCUCAUAGGGCACAGACCGCGCAC
KU501215    UCCAUGGAAAGGGAGAAUGGAUGACCACUGAAGACAUGCUUGUGGUGUGGAACAGAGUGUGGAUUGAGGAGAACGACCACAUGGAAGACAAGACCCCAGUUACGAAAUGGACAGACAUUCCCUAUUUGGGAAAAAGGGAAGACUUGUGGUGUGGAUCUCUCAUAGGGCACAGACCGCGCAC
KU365780    UCCAUGGAAAGGGAGAAUGGAUGACCACUGAAGACAUGCUUGUGGUGUGGAACAGAGUGUGGAUUGAGGAGAACGACCACAUGGAAGACAAGACCCCAGUUACGAAAUGGACAGACAUUCCCUAUUUGGGAAAAAGGGAAGACUUGUGGUGUGGAUCUCUCAUAGGGCACAGACCGCGCAC
KU365779    UCCAUGGAAAGGGAGAAUGGAUGACCACUGAAGACAUGCUUGUGGUGUGGAACAGAGUGUGGAUUGAGGAGAACGACCACAUGGAAGACAAGACCCCAGUUACGAAAUGGACAGACAUCCCCUAUUUGGGAAAAAGGGAAGACUUGUGGUGUGGAUCUCUCAUAGGGCACAGACCGCGCAC
KU707826    UCCAUGGAAAGGGAGAAUGGAUGACCACUGAAGACAUGCUUGUGGUGUGGAACAGAGUGUGGAUUGAGGAGAACGACCACAUGGAAGACAAGACCCCAGUUACGAAAUGGACAGACAUCCCCUAUUUGGGAAAAAGGGAAGACUUGUGGUGUGGAUCUCUCAUAGGGCACAGACCGCGCAC
KU365778    UCCAUGGAAAGGGAGAAUGGAUGACCACUGAAGACAUGCUUGUGGUGUGGAACAGAGUGUGGAUUGAGGAGAACGACCACAUGGAAGACAAGACCCCAGUUACGAAAUGGACAGACAUUCCCUAUUUGGGAAAAAGGGAAGACUUGUGGUGUGGAUCUCUCAUAGGGCACAGACCGCGCAC
KU365777    UCCAUGGAAAGGGAGAAUGGAUGACCACUGAAGACAUGCUUGUGGUGUGGAACAGAGUGUGGAUUGAGGAGAACGACCACAUGGAAGACAAGACCCCAGUUACGAAAUGGACAGACAUCCCCUAUUUGGGAAAAAGGGAAGACUUGUGGUGUGGAUCUCUCAUAGGGCACAGACCGCGCAC
KU312312    UCCAUGGAAAGGGAGAAUGGAUGACCACUGAAGACAUGCUUGUGGUGUGGAACAGAGUGUGGAUUGAGGAGAACGACCACAUGGAAGACAAGACCCCAGUUACGAAAUGGACAGACAUUCCCUAUUUGGGAAAAAGGGAAGACUUGUGGUGUGGAUCUCUCAUAGGGCACAGACCGCGCAC
KF268950    UCCACGGAAAGGGAGAAUGGAUGACUACUGAGGACAUGCUCAUGGUGUGGAAUAGAGUGUGGAUUGAGGAGAACGACCAUAUGGAGGACAAGACUCCUGUGACAAAGUGGACAGACAUCCCCUAUCUGGGAAAAAGGGAGGACUUAUGGUGUGGAUCCCUUAUAGGGCACAGGCCCCGCAC
KF268949    UCCACGGAAAGGGAGAAUGGAUGACUACUGAGGACAUGCUCAUGGUGUGGAAUAGAGUGUGGAUUGAGGAGAACGACCACAUGGAGGACAAGACUCCUGUGACAAAGUGGACAGACAUCCCCUAUCUGGGAAAAAGGGAGGACUUAUGGUGUGGAUCCCUUAUAGGGCACAGGCCCCGCAC
KF268948    UCCACGGAAAGGGAGAAUGGAUGACUACUGAGGACAUGCUCAUGGUGUGGAAUAGAGUGUGGAUUGAGGAGAACGACCAUAUGGAGGACAAGACUCCUGUGACAAAGUGGACAGACAUCCCCUAUCUGGGAAAAAGGGAGGACUUAUGGUGUGGAUCCCUUAUAGGGCACAGGCCCCGCAC
KJ776791    UCCAUGGAAAGGGAGAAUGGAUGACCACUGAAGACAUGCUUGUGGUGUGGAACAGAGUGUGGAUUGAGGAGAACGACCACAUGGAAGACAAGACCCCAGUUACGAAAUGGACAGACAUUCCCUAUUUGGGAAAAAGGGAAGACUUGUGGUGUGGAUCUCUCAUAGGGCACAGACCGCGCAC
KF383119    UCCAUGGAAAGGGAGAAUGGAUGACCACUGAGGACAUGCUCAUGGUGUGGAAUAGAGUGUGGAUUGAGGAGAACGACCAUAUGGAGGACAAGACUCCUGUAACAAAAUGGACAGACAUUCCCUAUCUAGGAAAAAGGGAGGACUUAUGGUGUGGAUCCCUUAUAGGGCACAGACCCCGCAC
KF383118    UCCAUGGAAAGGGAGAAUGGAUGACCACUGAGGACAUGCUCAUGGUGUGGAAUAGAGUGUGGAUUGAGGAGAACGACCAUAUGGAGGACAAGACUCCUGUAACAAAAUGGACAGACAUUCCCUAUCUAGGAAAAAGGGAGGACUUAUGGUGUGGAUCCCUUAUAGGGCACAGACCCCGCAC
KF383117    UCCAUGGAAAGGGAGAAUGGAUGACUACUGAGGACAUGCUCAUGGUGUGGAAUAGAGUGUGGAUUGAGGAGAACGACCACAUGGAGGACAAGACCCCUGUUCCAAAAUGGACAGAAAUUCCCUACUUGGGGAAAAGGGAGUCCCUAUGGUGUGGAUACAUUAUAGGGCACAGACCUCGCAC
KF383116    UCCAUGGAAAGGGAGAAUGGAUGACUACUGAGGACAUGCUCAUGGUGUGGAAUAGAGUGUGGAUUGAGGAGAACGACCACAUGGAGGACAAGACCCCUGUAACAAAAUGGACAGACAUUCCCUAUUUGGGGAAAAGGGAGGACUUAUGGUGUGGAUCCCUUAUAGGGCACAGACCUCGCAC
```

```
KF383115    UCCACGGGAAGGGAGAAUGGAUGACCACUGAGGACAUGCUCAUGGUGUGGAAUAGAGUGUGGAUUGAGGAGAACGACCAUAUGGAGGACAAGACUCCUGUAACAAAGUGGACUGACAUUCCCUACCUGGGAAAAAGGGAGGACUUAUGGUGUGGAUCCCUUAUAGGGCACAGACCCCGCAC
EU545988    UCCAUGGAAAGGGAGAAUGGAUGACCACUGAAGACAUGCUUGUGGUGUGGAACAGAGUGUGGAUUGAGGAGAACGACCACAUGGAAGACAAGACCCCAGUUACGAAAUGGACAGACAUUCCCUAUUUGGGAAAAAGGGAAGACUUGUGGUGUGGAUCUCUCAUAGGGCACAGACCGCGCAC
KU955589    UCCAUGGAAAGGGAGAAUGGAUGACCACUGAAGACAUGCUUGUGGUGUGGAACAGAGUGUGGAUUGAGGAGAACGACCACAUGGAAGACAAGACCCCAGUUACGAAAUGGACAGACAUUCCCUAUUUGGGAAAAAGGGAAGACUUGUGGUGUGGAUCUCUCAUAGGGCACAGACCGCGCAC
KU955590    UCCAUGGAAAGGGAGAAUGGAUGACCACUGAGGACAUGCUUGUGGUGUGGAACAGAGUGUGGAUUGAGGAGAACGACCACAUGGAAGACAAGACCCCAGUUACGAAAUGGACAGACAUUCCCUAUUUGGGAAAAAGGGAAGACUUGUGGUGUGGAUCUCUCAUAGGGCACAGACCGCGCAC
KU940228    UCCAUGGAAAGGGAGAAUGGAUGACCACUGAAGACAUGCUUGUGGUGUGGAACAGAGUGUGGAUUGAGGAGAACGACCACAUGGAAGACAAGACCCCAGUUACGAAAUGGACAGACAUUCCCUAUUUGGGAAAAAGGGAAGACUUGUGGUGUGGAUCUCUCAUAGGGCACAGACCGCGCAC
KU991811    UCCAUGGAAAGGGAGAAUGGAUGACCACUGAAGACAUGCUUGUGGUGUGGAACAGAGUGUGGAUUGAGGAGAACGACCACAUGGAAGACAAGACCCCAGUUACGAAAUGGACAGACAUUCCCUAUUUGGGAAAAAGGGAAGACUUGUGGUGUGGAUCUCUCAUAGGGCACAGACCGCGCAC
KU509998    UCCAUGGAAAGGGAGAAUGGAUGACCACUGAAGACAUGCUUGUGGUGUGGAACAGAGUGUGGAUUGAGGAGAACGACCACAUGGAAGACAAGACCCCAGUUACGAAAUGGACAGACAUUCCCUAUUUGGGAAAAAGGGAAGACUUGUGGUGUGGAUCUCUCAUAGGGCACAGACCGCGCAC
KX056898    UCCAUGGAAAGGGAGAAUGGAUGACCACUGAAGACAUGCUUGUGGUGUGGAACAGAGUGUGGAUUGAGGAGAACGACCACAUGGAAGACAAGACCCCAGUUACGAAAUGGACAGACAUUCCCUAUUUGGGAAAAAGGGAAGACUUGUGGUGUGGAUCUCUCAUAGGGCACAGACCGCGCAC
KX117076    UCCAUGGAAAGGGAGAAUGGAUGACCACUGAAGACAUGCUUGUGGUGUGGAACAGAGUGUGGAUUGAGGAGAACGACCACAUGGAAGACAAGACCCCAGUUACGAAAUGGACAGACAUUCCCUAUUUGGGAAAAAGGGAAGACUUGUGGUGUGGAUCUCUCAUAGGGCACAGACCGCGCAC
KX185891    UCCAUGGAAAGGGAGAAUGGAUGACCACUGAAGACAUGCUUGUGGUGUGGAACAGAGUGUGGAUUGAGGAGAACGACCACAUGGAAGACAAGACCCCAGUUACGAAAUGGACAGACAUUCCCUAUUUGGGAAAAAGGGAAGACUUGUGGUGUGGAUCUCUCAUAGGGCACAGACCGCGCAC
KX247646    UCCAUGGAAAGGGAGAAUGGAUGACCACUGAAGACAUGCUUGUGGUGUGGAACAGAGUGUGGAUUGAGGAGAACGACCACAUGGAAGACAAGACCCCAGUUACGAAAUGGACAGACAUUCCCUAUUUGGGAAAAAGGGAAGACUUGUGGUGUGGAUCUCUCAUAGGGCACAGACCGCGCAC
KX262887    UCCAUGGAAAGGGAGAAUGGAUGACCACUGAAGACAUGCUUGUGGUGUGGAACAGAGUGUGGAUUGAGGAGAACGACCACAUGGAAGACAAGACCCCAGUUACGAAAUGGACAGACAUUCCCUAUUUGGGAAAAAGGGAAGACUUGUGGUGUGGAUCUCUCAUAGGGCACAGACCGCGCAC
KX280026    UCCAUGGAAAGGGAGAAUGGAUGACCACUGAAGACAUGCUUGUGGUGUGGAACAGAGUGUGGAUUGAGGAGAACGACCACAUGGAAGACAAGACCCCAGUUACGAAAUGGACAGACAUUCCCUAUUUGGGAAAAAGGGAAGACUUGUGGUGUGGAUCUCUCAUAGGGCACAGACCGCGCAC
KU758877    UCCAUGGAAAGGGAGAAUGGAUGACCACUGAAGACAUGCUUGUGGUGUGGAACAGAGUGUGGAUUGAGGAAAACGACCACAUGGAAGACAAGACCCCAGUUACGAAAUGGACAGACAUUCCCUAUUUGGGAAAAAGGGAAGACUUGUGGUGUGGAUCUCUCAUAGGGCACAGACCGCGCAC
KU866423    UCCAUGGAAAGGGAGAAUGGAUGACCACUGAAGACAUGCUUGUGGUGUGGAACAGAGUGUGGAUUGAGGAGAACGACCACAUGGAAGACAAGACCCCAGUUACGAAAUGGACAGACAUUCCCUAUUUGGGAAAAAGGGAAGACUUGUGGUGUGGAUCUCUCAUAGGGCACAGACCGCGCAC
KX377336    UCCAUGGAAAGGGAGAAUGGAUGACCACUGAGGACAUGCUUGUGGUGUGGAACAGAGUGUGGAUUGAGGAGAACGACCACAUGGAGGACAAGACCCCAGUCACGAAAUGGACAGACAUUCCCUAUUUGGGAAAAAGGGAAGACUUAUGGUGUGGAUCUCUUAUAGGGCACAGACCACGCAC
KX377337    UCCAUGGAAAGGGAGAAUGGAUGACCACUGAAGACAUGCUUGUGGUGUGGAACAGAGUGUGGAUUGAGGAGAACGACCACAUGGAAGACAAGACCCCAGUUACGAAAUGGACAGACAUUCCCUAUUUGGGAAAAAGGGAAGACUUGUGGUGUGGAUCUCUCAUAGGGCACAGACCGCGCAC
KX369547    UCCAUGGAAAGGGAGAAUGGAUGACCACUGAAGACAUGCUUGUGGUGUGGAACAGAGUGUGGAUUGAGGAGAACGACCACAUGGAAGACAAGACCCCAGUUACGAAAUGGACAGACAUUCCCUAUUUGGGAAAAAGGGAAGACUUGUGGUGUGGAUCUCUCAUAGGGCACAGACCGCGCAC
KX548902    UCCAUGGAAAGGGAGAAUGGAUGACCACUGAAGACAUGCUUGUGGUGUGGAACAGAGUGUGGAUUGAGGAGAACGACCACAUGGAAGACAAGACCCCAGUUGCGAAAUGGACAGACAUUCCCUAUUUGGAAAAAAGGGAAGACUUGUGGUGUGGAUCUCUCAUAGGGCACAGACCGCGCAC
KX601166    UCCAUGGAAAGGGAGAAUGGAUGACUACUGAGGACAUGCUCAUGGUGUGGAAUAGAGUGUGGAUUGAGGAGAACGACCACAUGGAGGACAAGACCCCUGUAACAAAAUGGACAGACAUUCCCUAUUUGGGAAAAAGGGAGGACUUAUGGUGUGGAUCCCUUAUAGGGCAUAGACCUCGCAC
KX601167    UCCAUGGAAAGGGAGAAUGGAUGACCACUGAGGACAUGCUUGUGGUGUGGAACAGAGUGUGGAUUGAGGAGAACGACCACAUGGAGGACAAGACCCCAGUCACGAAAUGGACAGACAUUCCCUAUUUGGGAAAAAGGGAAGACUUAUGGUGUGGAUCUCUUAUAGGGCACAGACCACGCAC
KX601168    UCCAUGGAAAGGGAGAAUGGAUGACCACUGAAGACAUGCUUGUGGUGUGGAACAGAGUGUGGAUUGAGGAGAACGACCACAUGGAAGACAAGACCCCAGUUACGAAAUGGACAGACAUUCCCUAUUUGGGAAAAAGGGAAGACUUGUGGUGUGGAUCUCUCAUAGGGCACAGACCGCGCAC
KX766028    UCCAUGGAAAGGGAGAAUGGAUGACCACUGAAGACAUGCUUGUGGUGUGGAACAGAGUGUGGAUUGAGGAGAACGACCACAUGGAAGACAAGACCCCAGUUACGAAAUGGACAGACAUUCCCUAUUUGGGAAAAAGGGAAGACUUGUGGUGUGGAUCUCUCAUAGGGCACAGACCGCGCAC
KX766029    UCCAUGGAAAGGGAGAAUGGAUGACCACUGAAGACAUGCUUGUGGUGUGGAACAGAGUGUGGAUUGAGGAGAACGACCACAUGGAAGACAAGACCCCAGUUACGAAAUGGACAGACAUUCCCUAUUUGGGAAAAAGGGAAGACUUGUGGUGUGGAUCUCUCAUAGGGCACAGACCGCGCAC
KU940224    UCCAUGGAAAGGGAGAAUGGAUGACCACUGAAGACAUGCUUGUGGUGUGGAACAGAGUGUGGAUUGAGGAGAACGACCACAUGGAAGACAAGACCCCAGUUACGAAAUGGACAGACAUUCCCUAUUUGGGAAAAAGGGAAGACUUGUGGUGUGGAUCUCUCAUAGGGCACAGACCGCGCAC


            1111111111111111111111111111111111111111111111111111111111111111111111111111111111111111111111111111111111111111111111111111111111111111
            0000000000000000000000000000000000000000000000000000000000000000000000000000000000000000000000000000000000000000000000000000000000000000
            1111111111111111111111111111111111111111111111111111111111111112222222222222222222222222222222222222222222222222222222222222222222222222
            3334444444444555555555566666666667777777777888888888899999999990000000000111111111122222222223333333333444444444455555555556666666666777
Sequence    7890123456789012345678901234567890123456789012345678901234567890123456789012345678901234567890123456789012345678901234567890123456789012
----------------------------------------------------------------------------------------------------------------------------------------------------
NC_012532   CACUUGGGCUGAAAACAUCAAAGACACAGUCAACAUGGUGCGCAGGAUCAUAGGUGAUGAAGAAAAGUACAUGGACUAUCUAUCCACCCAAGUCCGCUACUUGGGUGAGGAAGGGUCCACACCCGGAGUGUUGUAA
KU922960    CACCUGGGCUGAGAACAUUAAAAACACAGUCAACAUGGUGCGCAGGAUCAUAGGUGAUGAAGAAAAGUACAUGGACUACCUAUCCACCCAAGUUCGCUACUUGGGUGAAGAAGGGUCUACACCUGGAGUGCUGUAA
KU922923    CACCUGGGCUGAGAACAUUAAAAACACAGUCAACAUGGUGCGCAGGAUCAUAGGUGAUGAAGAAAAGUACAUGGACUACCUAUCCACCCAAGUUCGCUACUUGGGUGAAGAAGGGUCUACACCUGGAGUGCUGUAA
KU820898    CACCUGGGCUGAGAACAUUAAAAACACAGUCAACAUGGUGCGCAGGAUCAUAGGUGAUGAAGAAAAGUACAUGGACUACCUAUCCACCCAAGUUCGCUACUUGGGUGAAGAAGGGUCUACACCUGGAGUGCUGUAA
KU321639    CACCUGGGCUGAGAACAUUAAAAACACAGUCAACAUGGUGCGCAGGAUCAUAGGUGAUGAAGAAAAGUACAUGGACUACCUAUCCACCCAAGUUCGCUACUUGGGUGAAGAAGGGUCUACACCUGGAGUGCUGUAA
KU820899    CACCUGGGCUGAGAACAUUAAAAACACAGUCAACAUGGUGCGCAGGAUCAUAGGUGAUGAAGAAAAGUACAUGGACUACCUAUCCACCCAAGUUCGCUACUUGGGUGAAGAAGGGUCUACACCUGGAGUGCUGUAA
KU853012    CACCUGGGCUGAGAACAUUAAAAACACAGUCAACAUGGUGCGCAGGAUCAUAGGUGAGGAAGAAAAGUACAUGGACUACCUAUCCACCCAAGUUCGCUACUUGGGUGAAGAAGGGUCUACACCUGGARUGCUGUAA
KU729217    CACCUGGGCUGAGAACAUUAAAAACACAGUCAACAUGGUGCGCAGGAUCAUAGGUGAUGAAGAAAAGUACAUGGACUACCUAUCCACCCAAGUUCGCUACUUGGGUGAAGAAGGGUCUACACCUGGAGUGCUGUAA
KU729218    CACCUGGGCUGAGAACAUUAAAAACACAGUCAACAUGGUGCGCAGGAUCAUAGGUGAUGAAGAAAAGUACAUGGACUACCUAUCCACCCAAGUUCGCUACUUGGGUGAAGAAGGGUCUACACCUGGAGUGCUGUAA
KU761564    CACCUGGGCUGAGAACAUUAAAAACACAGUCAACAUGGUGCGCAGGAUCAUAGGUGAUGAAGAAAAGUACAUGGAUUACCUAUCCACCCAAGUUCGCUACUUGGGUGAAGAAGGGUCUACACCUGGAGUGCUGUAA
KU740184    CACCUGGGCUGAGAACAUUAAAAACACAGUCAACAUGGUGCGCAGGAUCAUAGGUGAUGAAGAAAAGUACAUGGAUUACCUAUCCACCCAAGUUCGCUACUUGGGUGAAGAAGGGUCUACACCUGGAGUGCUGUAA
KU744693    CACCUGGGCUGAGAACAUUAAAAACACAGUCAACAUGGUGCGCAGGAUCAUAGGUGAUGAAGAAAAGUACAUGGACUACCUAUCCACCCAAGUUCGCUACUUGGGUGAAGAAGGGUCUACACCUGGAGUGCUGUAA
KU497555    CACCUGGGCUGAGAACAUUAAAAAUACAGUCAACAUGGUGCGCAGGAUCAUAGGUGAUGAAGAAAAGUACAUGGACUACCUAUCCACCCAAGUUCGCUACUUGGGUGAAGAAGGGUCUACACCUGGAGUGCUGUGA
KU527068    CACCUGGGCUGAGAACAUUAAAAACACAGUCAACAUGGUGCGCAGGAUCAUAGGUGAUGAAGAAAAGUACAUGGACUACCUAUCCACCCAAGUUCGCUACUUGGGUGAAGAAGGGUCUACACCUGGAGUGCUGUAA
KU501217    CACCUGGGCUGAGAACAUUAAAAACACAGUCAACAUGGUGCGCAGGAUCAUAGGUGAUGAAGAAAAGUACAUGGACUACCUAUCCACCCAAGUUCGCUACUUGGGUGAAGAAGGGUCUACACCUGGAGUGCUGUAA
KU501216    CACCUGGGCUGAGAACAUUAAAAACACAGUCAACAUGGUGCGCAGGAUCAUAGGUGAUGAAGAAAAGUACAUGGACUACCUAUCCACCCAAGUUCGCUACUUGGGUGAAGAAGGGUCUACACCUGGAGUGCUGUAA
KU501215    CACCUGGGCUGAGAACAUUAAAAACACAGUCAACAUGGUGCGCAGGAUCAUAGGUGAUGAAGAAAAGUACAUGGACUACCUAUCCACCCAAGUUCGCUACUUGGGUGAAGAAGGGUCUACACCUGGAGUGCUGUAA
KU365780    CACCUGGGCUGAGAACAUUAAAAACACAGUCAACAUGGUGCGCAGGAUCAUAGGUGAUGAAGAAAAGUACAUGGACUACCUAUCCACCCAAGUUCGCUACUUGGGUGAAGAAGGGUCUACACCUGGAGUGCUGUAA
KU365779    CACCUGGGCUGAGAACAUUAAAAACACAGUCAACAUGGUGCGCAGGAUCAUAGGUGAUGAAGAAAAGUACAUGGACUACCUAUCCACCCAAGUUCGCUACUUGGGUGAAGAAGGGUCUACACCUGGAGUGCUGUAA
KU707826    CACCUGGGCUGAGAACAUUAAAAACACAGUCAACAUGGUGCGCAGGAUCAUAGGUGAUGAAGAAAAGUACAUGGACUACCUAUCCACCCAAGUUCGCUACUUGGGUGAAGAAGGGUCUACACCUGGAGUGCUGUAA
KU365778    CACCUGGGCUGAGAACAUUAAAAACACAGUCAACAUGGUGCGCAGGAUCAUAGGUGAUGAAGAAAAGUACAUGGACUACCUAUCCACCCAAGUUCGCUACUUGGGUGAAGAAGGGUCUACACCUGGAGUGCUGUAA
KU365777    CACCUGGGCUGAGAACAUUAAAAACACAGUCAACAUGGUGCGCAGGAUCAUAGGUGAUGAAGAAAAGUACAUGGACUACCUAUCCACCCAAGUUCGCUACUUGGGUGAAGAAGGGUCUACACCUGGAGUGCUGUAA
KU312312    CACCUGGGCUGAGAACAUUAAAAACACAGUCAACAUGGUGCGCAGGAUCAUAGGUGAUGAAGAAAAGUACAUGGACUACCUAUCCACCCAAGUUCGCUACUUGGGUGAAGAAGGGUCUACACCUGGAGUGCUGUAA
KF268950    CACUUGGGCUGAGAACAUCAAAGACACAGUCAACAUGGUGCGCAGGAUCAUAGGUGAUGAAGAAAAAUACAUGGACUACUUAUCCACCCAGGUCCGCUACUUGGGUGAGGAAGGGUCCACACCUGGAGUGUUGUAA
KF268949    CACUUGGGCUGAGAACAUCAAAGACACAGUCAACAUGGUGCGCAGGAUCAUAGGUGAUGAAGAAAAAUACAUGGACUACUUAUCCACCCAGGUCCGCUACUUGGGUGAGGAAGGGUCCACACCUGGAGUGUUGUAA
KF268948    CACUUGGGCUGAGAACAUCAAAGACACAGUCAACAUGGUGCGCAGGAUCAUAGGUGAUGAAGAAAAAUACAUGGACUACUUAUCCACCCAGGUCCGCUACUUGGGUGAGGAAGGGUCCACACCUGGAGUGUUGUAA
KJ776791    CACCUGGGCUGAGAACAUUAAAAACACAGUCAACAUGGUGCGCAGGAUCAUAGGUGAUGAAGAAAAGUACAUGGACUACCUAUCCACCCAAGUUCGCUACUUGGGUGAAGAAGGGUCUACACCUGGAGUGCUGUAA
KF383119    CACUUGGGCUGAAAACAUCAAAGACACAGUCAACAUGGUGCGCAGGAUCAUAGGUGAUGAAGAAAAGUACAUGGACUAUCUAUCCACCCAAGUCCGCUACUUGGGUGAGGAAGGGUCCACACCCGGAGUGUUGUAA
KF383118    CACUUGGGCUGAAAACAUCAAAGACACAGUCAACAUGGUGCGCAGGAUCAUAGGUGAUGAAGAAAAGUACAUGGACUAUCUAUCCACCCAAGUCCGCUACUUGGGUGAGGAAGGGUCCACACCCGGAGUGUUGUAA
KF383117    CACUUGGGCUGAGAACAUCAAAGACACAGUCAACAUGGUGCUUAGGCUCAUAGGUGAUGAAGAAAAGUACAUGGACUACCUAUCCACCCAAGUACGCUACUUGGGUGAGGAAGGGUCCACACCUGGAGUGCUGUAA
KF383116    CACUUGGGCUGAGAACAUCAAAGACACAGUCAACAUGGUGCGUAGGAUCAUAGGUGAUGAAGAAAAGUACAUGGACUACCUAUCCACCCAAGUACGCUACUUGGGUGAGGAAGGGUCCACACCUGGAGUGCUGUAA
KF383115    CACUUGGGCUGAGAACAUCAAAGACACAGUCAACAUGGUGCGCAGGAUCAUAGGUGAUGAAGAAAAAUACAUGGACUACCUAUCCACCCAAGUCCGCUACUUGGGUGAGGAAGGGUCCACACCUGGAGUGUUGUAA
EU545988    UACCUGGGCUGAGAACAUCAAAAACACAGUCAACAUGAUGCGCAGGAUCAUAGGUGAUGAAGAAAAGUACAUGGACUACCUAUCCACCCAAGUUCGCUACUUGGGUGAAGAAGGGUCCACACCCGGAGUGUUGUAA
KU955589    CACCUGGGCUGAGAACAUUAAAAACACAGUCAACAUGGUGCGCAGGAUCAUAGGUGAUGAAGAAAAGUACAUGGACUACCUAUCCACCCAAGUUCGCUACUUGGGUGAAGAAGGGUCUACACCUGGAGUGCUGUAA
KU955590    CACCUGGGCUGAGAACAUUAAAAACACAGUCAACAUGGUGCGCAGGAUCAUAGGUGAUGAAGAAAAGUACAUGGAUUACCUAUCCACCCAAGUUCGCUACUUGGGUGAAGAAGGGUCUACACCUGGAGUGCUGUAA
KU940228    CACCUGGGCUGAGAACAUUAAAAACACAGUCAACAUGGUGCGCAGGAUCAUAGGUGAUGAAGAAAAGUACAUGGACUACCUAUCCACCCAAGUUCGCUACUUGGGUGAAGAAGGGUCUACACCUGGAGUGCUGUAA
KU991811    CACCUGGGCUGAGAACAUUAAAAACACAGUCAACAUGGUGCGCAGGAUCAUAGGUGAUGAAGAAAAGUACAUGGACUACCUAUCCACCCAAGUUCGCUACUUGGGUGAAGAAGGGUCUACACCUGGAGUGCUGUAA
KU509998    CACCUGGGCUGAGAACAUUAAAAACACAGUCAACAUGGUGCGCAGGAUCAUAGGUGAUGAAGAAAAGUACAUGGACUACCUAUCCACCCAAGUUCGCUACUUGGGUGAAGAAGGGUCUACACCUGGAGUGCUGUAA
KX056898    CACCUGGGCUGAGAACAUUAAAAACACAGUCAACAUGGUGCGCAGGAUCAUAGGUGAUGAAGAAAAGUACAUGGAUUACCUAUCCACCCAAGUUCGCUACUUGGGUGAAGAAGGGUCUACACCUGGAGUGCUGUAA
KX117076    CACCUGGGCUGAGAACAUUAAAAACACAGUCAACAUGGUGCGCAGGAUCAUAGGUGAUGAAGAAAAGUACAUGGACUACCUAUCCACCCAAGUUCGCUACUUGGGUGAAGAAGGGUCUACACCUGGAGUGCUGUAA
KX185891    CACCUGGGCUGAGAACAUUAAAAACACAGUCAACAUGGUGCGCAGGAUCAUAGGUGAUGAAGAAAAGUACAUGGACUACCUAUCCACCCAAGUUCGCUACUUGGGUGAAGAAGGGUCUACACCUGGAGUGCUGUAA
KX247646    CACCUGGGCUGAGAACAUUAAAAACACAGUCAACAUGGUGCGCAGGAUCAUAGGUGAUGAAGAAAAGUACAUGGACUACCUAUCCACCCAAGUUCGCUACUUGGGUGAAGAAGGGUCUACACCUGGAGUGCUGUAA
KX262887    CACCUGGGCUGAGAACAUUAAAAACACAGUCAACAUGGUGCGCAGGAUCAUAGGUGAUGAAGAAAAGUACAUGGACUACCUAUCCACCCAAGUUCGCUACUUGGGUGAAGAAGGGUCUACACCUGGAGUGCUGUAA
KX280026    CACCUGGGCUGAGAACAUUAAAAACACAGUCAACAUGGUGCGCAGGAUCAUAGGUGAUGAAGAAAAGUACAUGGACUACCUAUCCACCCAAGUUCGCUACUUGGGUGAAGAAGGGUCUACACCUGGAGUGCUGUAA
KU758877    CACCUGGGCUGAGAACAUUAAAAACACAGUCAACAUGGUGCGCAGGAUCAUAGGUGAUGAAGAAAAGUACAUGGACUACCUAUCCACCCAAGUUCGCUACUUGGGUGAAGAAGGGUCUACACCUGGAGUGCUGUAA
```

KU866423	CACCUGGGCUGAGAACAUUAAAAACACAGUCAACAUGGUGCGCAGGAUCAUAGGUGAUGAAGAAAAGUACAUGGACUACCUAUCCACCCAAGUUCGCUACUUGGGUGAAGAAGGGUCUACACCUGGAGUGCUGUAA
KX377336	UACUUGGGCUGAGAACAUUAAAGACACAGUCAACAUGGUGCGCAGGAUCAUAGGUGAUGAAGAAAAGUACAUGGACUACCUAUCCACUCAAGUUCGCUACUUGGGUGAAGAAGGGUCCACACCUGGAGUGUUAUAA
KX377337	CACCUGGGCUGAGAACAUUAAAAACACAGUCAACAUGGUGCGCAGGAUCAUAGGUGAUGAAGAAAAGUACAUGGACUACCUAUCCACCCAAGUUCGCUACUUGGGUGAAGAAGGGUCUACACCUGGAGUGCUGUAA
KX369547	CACCUGGGCUGAGAACAUUAAAAACACAGUCAACAUGGUGCGCAGGAUCAUAGGUGAUGAAGAAAAGUACAUGGACUACCUAUCCACCCAAGUUCGCUACUUGGGUGAAGAAGGGUCUACACCUGGAGUGCUGUAA
KX548902	CACCUGGGCUGAGAACAUUAAAAACACAGUCAACAUGGUGCGCAGGAUCAUAGGUGAUGAAGAAAAGUACAUGGACUACCUAUCCACCCAAGUUCGCUACUUGGGUGAAGAAGGGUCUACACCUGGAGUGCUGUAA
KX601166	CACUUGGGCUGAGAACAUCAAAGACACAGUCAACAUGGUGCGUAGGAUCAUAGGUGAUGAAGAAAAGUUCAUGGACUACCUAUCCACCCAAGUACGCUACUUGGGUGAGGAAGGGUCCACACCUGGAGUGCUGUAA
KX601167	UACUUGGGCUGAGAACAUUAAAGACACAGUCAACAUGGUGCGCAGGAUCAUAGGUGAUGAAGAAAAGUACAUGGACUACCUAUCCACUCAAGUUCGCUACUUGGGUGAAGAAGGGUCCACACCUGGAGUGUUAUAA
KX601168	CACCUGGGCUGAGAACAUUAAAAACACAGUCAACAUGGUGCGCAGGAUCAUAGGUGAUGAAGAAAAGUACAUGGACUACCUAUCCACCCAAGUUCGCUACUUGGGUGAAGAAGGGUCUACACCUGGAGUGCUGUAA
KX766028	CACCUGGGCUGAGAACAUUAAAAACACAGUCAACAUGGUGCGCAGGAUCAUAGGUGAUGAAGAAAAGUACAUGGAUUACCUAUCCACCCAAGUUCGCUACUUGGGUGAAGAAGGGUCUACACCUGGAGUGCUGUAA
KX766029	CACCUGGGCUGAGAACAUUAAAAACACAGUCAACAUGGUGCGCAGGAUCAUAGGUGAUGAAGAAAAGUACAUGGACUACCUAUCCACCCAAGUUCGCUACUUGGGUGAAGAAGGGUCUACACCUGGAGUGCUGUAA
KU940224	CACCUGGGCUGAGAACAUUAAAAACACAGUCAACAUGGUGCGCAGGAUCAUAGGUGAUGAAGAAAAGUACAUGGACUACCUAUCCACCCAAGUUCGCUACUUGGGUGAAGAAGGGUCUACACCUGGAGUGCUGUAA
